# Using Speech Features and Machine Learning Models to Predict Emotional and Behavioral Problems in Chinese Adolescents

**DOI:** 10.1155/da/5734107

**Published:** 2025-06-16

**Authors:** Jinyu Li, Yang Wang, Fei Wang, Ran Zhang, Ning Wang, Yue Zhu, Taihong Zhao

**Affiliations:** ^1^The Affiliated Brain Hospital of Nanjing Medical University, Nanjing, China; ^2^Nanjing Medical University, Nanjing, China; ^3^Xinxiang Medical University, Xinxiang, Henan Province, China; ^4^China Medical University, Shenyang, Liaoning, China

**Keywords:** adolescent mental health, emotional and behavioral problems, gender differences, machine learning, speech features

## Abstract

**Background:** Current assessments of adolescent emotional and behavioral problems rely heavily on subjective reports, which are prone to biases.

**Aim:** This study is the first to explore the potential of speech signals as objective markers for predicting emotional and behavioral problems (hyperactivity, emotional symptoms, conduct problems, and peer problems) in adolescents using machine learning techniques.

**Materials and Methods:** We analyzed speech data from 8215 adolescents aged 12–18 years, extracting four categories of speech features: mel-frequency cepstral coefficients (MFCC), mel energy spectrum (MELS), prosodic features (PROS), and formant features (FORM). Machine learning models—logistic regression (LR), support vector machine (SVM), and gradient boosting decision trees (GBDT)—were employed to classify hyperactivity, emotional symptoms, conduct problems, and peer problems as defined by the Strengths and Difficulties Questionnaire (SDQ). Model performance was assessed using area under the curve (AUC), F1-score, and Shapley additive explanations (SHAP) values.

**Results:** The GBDT model achieved the highest accuracy for predicting hyperactivity (AUC = 0.78) and emotional symptoms (AUC = 0.74 for males and 0.66 for females), while performance was weaker for conduct and peer problems. SHAP analysis revealed gender-specific feature importance patterns, with certain speech features being more critical for males than females.

**Conclusion:** These findings demonstrate the feasibility of using speech features to objectively predict emotional and behavioral problems in adolescents and identify gender-specific markers. This study lays the foundation for developing speech-based assessment tools for early identification and intervention, offering an objective alternative to traditional subjective evaluation methods.

## 1. Introduction

Emotional and behavioral problems (e.g., emotional symptoms, hyperactivity, conduct problems, and peer relationship issues) are widespread among adolescents, significantly impacting their development and long-term mental health. These problems can lead to enduring psychological disorders and impair social functioning [1]. Current assessments primarily rely on subjective reports from parents, teachers, or the adolescents themselves, which are often influenced by personal biases and memory errors, compromising the accuracy of the evaluations [2]. Thus, there is an urgent need for objective assessment measures to overcome the limitations of existing methods [3].

Speech signals represent a potential objective measure, rich in information on cognitive and emotional states [4]. Research has shown that prosodic features (e.g., pitch and energy variation), spectral features and formant features can effectively reflect an individual's mental state [5, 6]. These speech features offer new possibilities for objectively evaluating behavioral problems and mental health conditions.

Previous studies have found potential links between speech features and emotional or behavioral problems. For example, Lima and Behlau [7] found that adolescents with vocal issues scored higher in behavioral assessments, while Strand et al. [8] highlighted the influence of cultural and language factors on emotional understanding and behavior. Glogowska et al. [9] and Westrupp et al. [10] revealed long-term associations between language development and behavioral problems. Additionally, Aro et al. [11] suggested that emotional and behavioral symptoms may differ by gender. However, these studies are mostly descriptive and research directly using speech features to predict emotional and behavioral problems remains scarce.

This study aims to systematically explore the value and feasibility of speech features in predicting adolescent emotional and behavioral problems. First, previous research has explored the potential of speech features to assess emotional and behavioral problems, indicating that these features may contain key information. Second, emotional and behavioral problems are highly comorbid with other mental health issues (e.g., anxiety and depression) and speech features have shown promising results in predicting these conditions [12–14]. Therefore, it is reasonable to infer that speech features play a crucial role in predicting emotional and behavioral problems.

Nevertheless, existing research has some limitations. For example, most studies focus on only a few types of speech features without considering the diversity and interrelationships between features; the results are often inconsistent, potentially due to differences in data processing methods, sample characteristics, or cultural contexts [15]. Small sample sizes may also limit the generalizability of the results, leading to overestimated predictive effects [16]. To address these gaps, this study is the first to systematically utilize speech features to aid in predicting and diagnosing various emotional and behavioral problems assessed by the Strengths and Difficulties Questionnaire (SDQ).

This study aims to address the following key scientific questions:

1. Can prediction models for emotional and behavioral problems be established for both genders using large-sample speech features?
2. What are the optimal speech feature categories or combinations for predicting each emotional and behavioral problem for males and females?
3. Are there gender differences in predicting the same emotional and behavioral problems using different speech feature categories?

## 2. Materials and Methods

**Figure 1:**
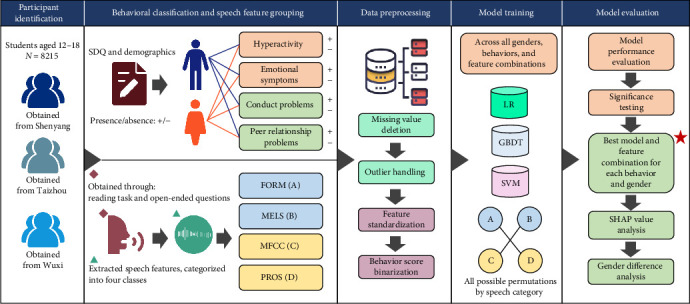
Analytical workflow of the study.

The detailed analysis workflow is shown in Figure 1, which presents the steps from data preprocessing to model evaluation and interpretation.

### 2.1. Participants

The sample was drawn from Phase 1 of the “SEARCH” database [17], which collected longitudinal data on child and adolescent health. The original dataset included 11,427 voice recordings and complete responses to the Student SDQ. Based on the study objective, we established the following screening criteria for the database: (a) participants had to be between 12 and 18 years old, (b) complete a fixed-content reading task and an open-ended question voice recording task, and (c) provide complete data from the SDQ. After applying these criteria and completing the data cleaning process, a total of 8215 eligible adolescents were included in the study, with 4370 males and 3845 females. The study was approved by the ethics committee of the Affiliated Brain Hospital of Nanjing Medical University (Ethics Approval No.: 2022-KY095-02).

### 2.2. Sample Size Estimation

The sample size was estimated based on the binary classification task for each emotional and behavioral problem dimension, considering 378 independent variables included in the study. The significance level was set at *α* = 0.05, with a power of 0.80, assuming a medium effect size [18]. The minimum required sample size for each gender group was estimated using G*⁣*^*∗*^Power software, taking into account the possible proportion of invalid questionnaires [19].

### 2.3. Instruments

Emotional and behavioral problems were assessed using the self-report version of the SDQ [22] (25 items across five subscales) with three-point Likert-scale responses (0 = “Not true,” 1 = “Somewhat true,” and 2 = “Certainly true”).The SDQ was selected as the reference standard for the following reasons: the scale has been extensively validated in Chinese adolescents, with recent studies demonstrating acceptable reliability and validity of the Chinese version, particularly showing good internal consistency (*α* = 0.75) on the total difficulties scale [20]. Furthermore, this instrument provides comprehensive assessment across multiple emotional and behavioral dimensions, while maintaining practical feasibility for large-scale data collection [21]. Threshold scores were dichotomized based on Goodman's [22] early research and population characteristics: hyperactivity threshold = 5 (score >5 considered problematic), emotional symptoms threshold = 3 (score >3 considered problematic), peer problems threshold = 2 (score >2 considered problematic), and conduct problems threshold = 2 (score >2 considered problematic). Demographic information such as gender and age was collected using a self-designed questionnaire.

Voice samples were recorded using an Android device, including a fixed-content reading task (Mandarin version of “The North Wind and the Sun,” ~3 min) [17] and four open-ended question tasks (see Supporting Information 1: Appendix A for questionnaire details and reading tasks). Audio data were converted to WAV format using FFmpeg, with noise reduction and silent segment removal. Prosodic, voice quality, energy, spectral, and formant features were extracted using the openSMILE toolkit [23]. Four key categories of features were selected: mel-frequency cepstral coefficients (MFCC), mel energy spectrum (MELS), prosodic features (PROS), and formant features (FORM) [24–27], the composition and number of features in each of the four speech feature categories are presented in Table 1. The reference standard (SDQ) and index test (speech recording) were administered concurrently and no clinical interventions occurred between the two assessments.

### 2.4. Data Preprocessing and Statistical Analysis

Data preprocessing involved handling complete datasets with no missing values, outlier detection (1%–99% quantile method) and *Z*-score normalization [28, 29]. All combinations of the four speech feature categories were generated, resulting in 15 feature combinations.  Table 2 presents a detailed list of all possible feature category combinations. In this study, we selected three machine learning algorithms—logistic regression (LR), support vector machine (SVM), and gradient boosting decision tree (GBDT)—to construct emotion and behavior prediction models. First, LR represents a machine learning algorithm extensively applied in classification tasks, particularly suitable for binary classification problems. For instance, in sentiment analysis applications, it can effectively predict textual sentiment polarity (e.g., positive or negative emotions) [30]. SVM demonstrates superior performance in handling complex nonlinear classification problems within high-dimensional spaces. In domains such as sentiment analysis, cognitive diagnosis, and mental health assessment, SVM achieves effective category differentiation and continuous variable prediction (e.g., emotional states or mental health risks) through the construction of optimal hyperplanes [31]. GBDT, as a robust ensemble learning method, progressively enhances prediction accuracy by sequentially building decision trees to minimize prediction errors. Recognized for its exceptional performance in processing large-scale complex datasets and improving model accuracy, GBDT is particularly effective for analyzing multidimensional feature relationships in emotion and behavior prediction tasks [32].The selection criteria for these algorithms were based not only on their theoretical foundations and general applicability in categorical prediction but also referenced their documented success in emotion recognition and behavior prediction applications. The hyperparameter optimization process employed randomized grid search with 50 iterations to balance computational efficiency and parameter space exploration. For each algorithm, we defined a comprehensive candidate parameter space based on empirical evidence from previous emotion prediction studies in Table 3. The randomized search strategy was systematically applied across all model configurations under different feature combinations. The SMOTE technique was applied to address class imbalance and SelectKBest was used to select the most relevant features [33, 34]. Model performance was evaluated through stratified fivefold cross-validation [35]. Evaluation metrics included area under the curve (AUC), F1-score, sensitivity, specificity, and precision. The best model was selected based on the average AUC and paired *t*-tests were used to compare the performance differences between feature combinations and models. Shapley additive explanations (SHAPs) was used to interpret the best prediction model for each emotional and behavioral problem, assessing the marginal contribution of each feature to the model output and feature importance plots were generated [36]. In this study, we set the machine learning classification threshold at 0.5, categorizing participants with predicted probabilities above 0.5 as having emotional or behavioral problems and those at or below as nonproblematic. This threshold was chosen to balance sensitivity and specificity based on receiver operating characteristic (ROC) curve analysis, ensuring optimal performance across different symptom dimensions and genders. The performers of the index test were blinded to the participants' SDQ results. Conversely, the assessors of the reference standard were unaware of the index test outcomes.

## 3. Results

### 3.1. Sample Description

According to the G*⁣*^*∗*^Power software calculations, the final sample size requirement for each gender group was at least 2064 participants. In this study, the actual sample size met this requirement, with 4370 males and 3845 females, totaling 8215 participants. Among male participants, 16.32% exhibited hyperactivity problems, 23.00% had emotional symptoms, 60.69% had peer relationship issues, and 42.29% showed conduct problems. Among female participants, these percentages were 17.89%, 35.66%, 53.71%, and 40.00%, respectively. Participants without emotional or behavioral problems did not have alternative diagnoses recorded.

### 3.2. Associations Between Emotional and Behavioral Problems and Speech Features

Exploratory analysis of the 378 speech features revealed significant differences between problematic and nonproblematic groups across different dimensions and genders (Table 4, complete results in Supporting Information 1: Appendix B). For example, in hyperactivity symptoms, males in the problematic group had significantly lower mean F1 values compared to the nonproblematic group (993.39 ± 388.15 vs. 1072.80 ± 465.41, *p*  < 0.001, Cohen's *d* = −0.19), while females in the problematic group had significantly higher MELS 5 standard deviation values compared to the nonproblematic group (52.01 ± 7.26 vs. 50.54 ± 7.72, *p*  < 0.001, *d* = 0.20). In emotional symptoms, males in the problematic group had significantly higher maximum second derivative values for F3 compared to the nonproblematic group (6348.35 ± 1884.51 vs. 6018.80 ± 2029.42, *p*  < 0.001, *d* = 0.17), while females also showed significantly higher MELS 5 std (*p*  < 0.001, *d* = 0.20). For conduct problems, the mean F1 values were significantly lower in the problematic group for both males (*d* = −0.18) and females (*d* = −0.18). For peer relationship problems, males in the problematic group had significantly higher kurtosis for MELS 3 (*p*  < 0.001, *d* = 0.11), while females had significantly higher standard deviation for MFCC 6 (*p*  < 0.001, *d* = 0.15).

### 3.3. Significance Testing Results

Significance testing showed the GBDT model performed best across all symptom-gender combinations, with optimal feature sets varying by gender (e.g., MFCC + PROS for male emotional symptoms, MFCC alone for female hyperactivity, all *p*  < 0.05). Even in nonsignificant cases like female peer problems (AUC = 0.5707), GBDT maintained superior performance over LR and SVM models. Full significance testing results are provided in the Supporting Information 1: Appendix C (intramodel variability test) and Supporting Information 1: Appendix D (optimal feature difference test across multiple models).

### 3.4. Best Model and Feature Combinations

**Figure 2:**
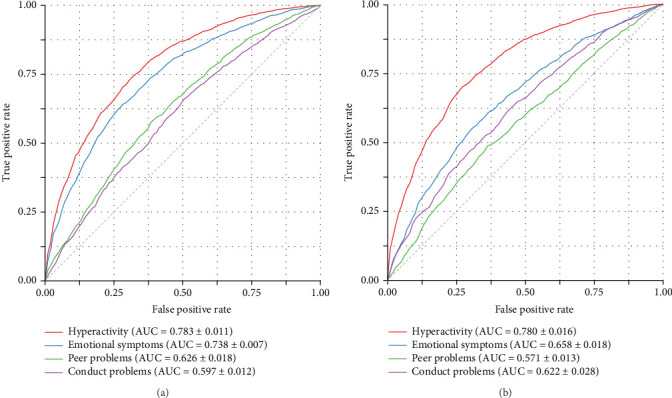
(a) ROC curves for different symptoms (male) and (b) ROC curves for different symptoms (female).

**Figure 3:**
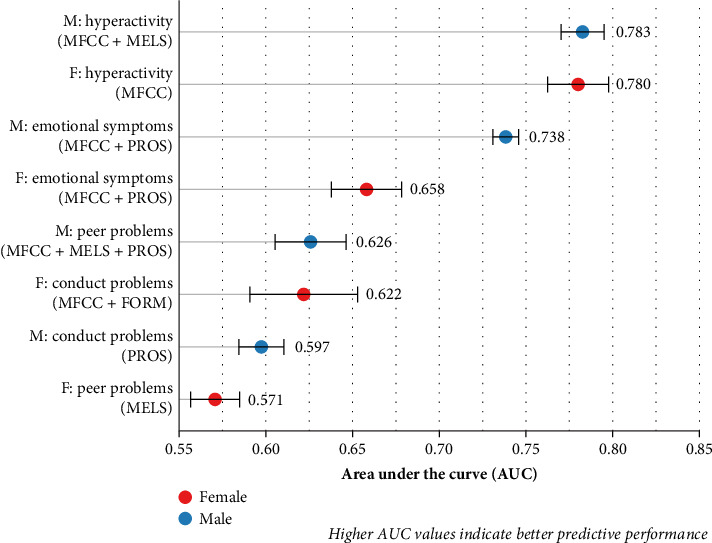
AUC values distribution by gender for different symptoms. This figure shows the AUC values for males and females across different behavioral problems, representing the predictive performance of the best feature combinations for each. Blue represents males, red represents females, and the error bars indicate ±1 standard deviation.

Table 5 presents the optimal feature combinations and performance metrics for the GBDT model across emotional and behavioral problem dimensions and gender combinations. Detailed performance data are provided in Supporting Information 1: Appendix E. Based on the data in the table, the results showed that for hyperactivity, the best feature combination for males was MFCC + MELS (AUC = 0.783 ± 0.011, F1 = 0.725 ± 0.010), while for females it was MFCC (AUC = 0.780 ± 0.016, F1 = 0.722 ± 0.019). For emotional symptoms, MFCC + PROS was the best combination for both genders, but the predictive performance was higher for males (AUC = 0.738 ± 0.007, F1 = 0.686 ± 0.010) compared to females (AUC = 0.658 ± 0.018, F1 = 0.632 ± 0.014). For conduct problems, the best feature combination for males was PROS (AUC = 0.597 ± 0.012, F1 = 0.600 ± 0.011), while for females it was MFCC + FORM (AUC = 0.622 ± 0.028, F1 = 0.599 ± 0.020). For peer problems, the best combination for males was MFCC + MELS + PROS (AUC = 0.626 ± 0.015, F1 = 0.562 ± 0.017), while for females it was MELS (AUC = 0.571 ± 0.013, F1 = 0.534 ± 0.019). Overall, the prediction performance for conduct problems and peer problems was relatively weaker. The ROC curves are shown in Figures 2, with AUC box plots in Figure 3. Clinically actionable thresholds determined by Youden's index (*J* = sensitivity + specificity − 1) for dimension-specific optimal models are provided in Table 6.

### 3.5. Feature Importance

**Figure 4:**
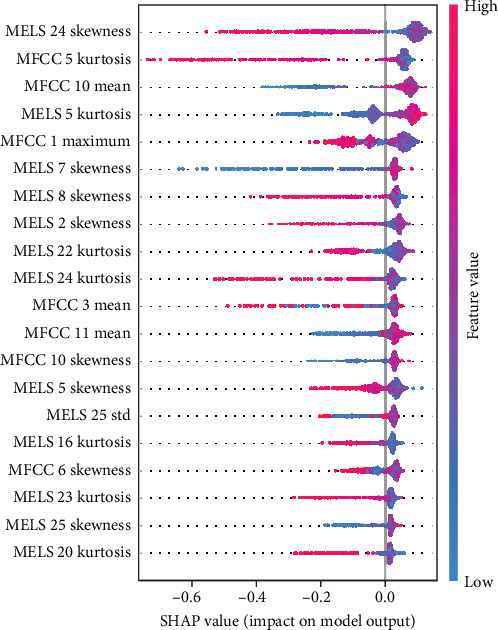
SHAP summary plot for hyperactivity (male). This figure illustrates the SHAP values for the top features contributing to the prediction of hyperactivity. Each dot represents a feature's impact on the model output, with higher SHAP values indicating a greater contribution. Feature values are color-coded, with red indicating high feature values and blue indicating low feature values. The features are ordered by their importance, with those at the top having a greater influence on the model's predictions.

**Figure 5:**
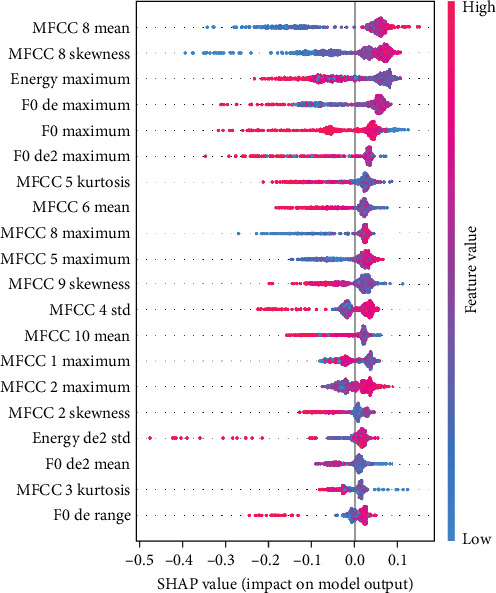
SHAP summary plot for emotional symptoms (female). This figure illustrates the SHAP values for the top features contributing to the prediction of emotional symptoms. Each dot represents a feature's impact on the model output, with higher SHAP values indicating a greater contribution. Feature values are color-coded, with red indicating high feature values and blue indicating low feature values. The features are ordered by their importance, with those at the top having a greater influence on the model's predictions.

**Figure 6:**
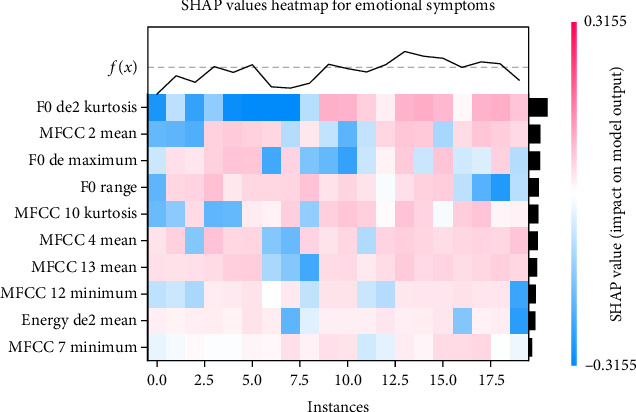
This heatmap (male) shows SHAP values for the key features predicting emotional symptoms. Red indicates a positive impact on the model output and blue a negative impact. Features are ranked by importance and the color gradient visualizes their influence across instances. The line plot above displays the predicted output *f*(*x*) for each instance.

**Figure 7:**
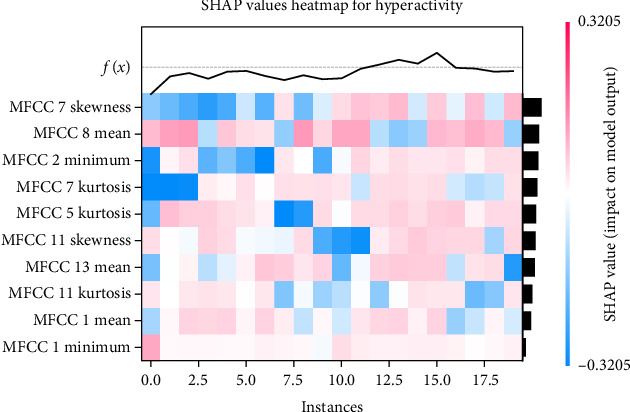
This heatmap (female) shows SHAP values for the key features predicting hyperactivity. Red indicates a positive impact on the model output and blue a negative impact. Features are ranked by importance and the color gradient visualizes their influence across instances. The line plot above displays the predicted output *f*(*x*) for each instance.

Figures 4 and 5 show the feature importance for predicting male hyperactivity and female emotional symptoms, respectively, using the best prediction models. SHAP analyses for all dimensions and gender groups and feature importance are presented in Supporting Information 1: Appendix F. Significant differences were observed in the influence of speech features on model predictions across genders and symptoms: for hyperactivity symptoms, male predictors included MELS 24 skewness and MFCC 5 kurtosis (negative direction), and MFCC 10 mean (positive direction), while female predictors included MFCC 8 mean and MFCC 7 skewness (both positive direction). For peer problems, male predictors included MELS 16 and MELS 21 kurtosis (positive direction), while female predictors included MELS 2 skewness (negative direction) and MELS 3 skewness (positive direction). For conduct problems, male predictors included energy maximum (strong positive direction) and energy skewness (strong negative direction), while female predictors included MFCC 10 mean and MFCC 4 maximum (positive direction). For emotional symptoms, male predictors included F0 second derivative kurtosis and F0 first derivative maximum (both negative direction), while female predictors included MFCC 8 mean (positive direction) and skewness (negative direction). SHAP case analysis (Figures 6 and 7, all dimensions and gender groups are presented in Supporting Information 1: Appendix G) showed opposing effects between certain features; for example, F0 second derivative kurtosis and F0 first derivative maximum had opposing effects in predicting male emotional symptoms, while MFCC 7 skewness (negative) and MFCC 8 mean (positive) had opposing effects in female hyperactivity symptoms, especially when prediction values were close to 0.5.

## 4. Discussion

### 4.1. Main Findings and Interpretation

This study explored the predictive value of speech features for hyperactivity, emotional symptoms, conduct problems, and peer problems in a large sample of adolescents. The prediction models constructed using the GBDT algorithm performed well in hyperactivity (male AUC = 0.783 and female AUC = 0.780) and emotional symptoms (male AUC = 0.738 and female AUC = 0.658). This suggests that speech features alone can effectively predict emotional and behavioral problems in adolescents, expanding the application of speech features from previous systematic reviews indicating their ability to detect depression and schizophrenia [15].

Overall, the optimal feature combinations differed significantly by symptom and gender. For hyperactivity, the optimal feature combinations were MFCC + MELS for males and MFCC for females, while for emotional problems, MFCC + PROS was the best combination for both genders. For conduct problems, the best feature combination was PROS for males and MFCC + FORM for females, while for peer problems, MFCC + MELS + PROS was best for males and MELS for females. This indicates that appropriate feature combinations should be selected based on the specific symptom and gender when developing speech assessment tools [37].

MFCC emerged as one of the best feature combinations across all symptoms and genders, likely because it contains rich speech information such as articulation and speech rate [24]. However, the optimal combination of MFCC with other feature categories (e.g., MELS and PROS) varied by symptom and gender. For example, males' optimal combinations for hyperactivity and peer problems included both MFCC and MELS, which may be related to greater spectral energy variation in males for these problems [38], possibly leading to difficulties in attention allocation and self-regulation [39]. In contrast, females relied only on MFCC for hyperactivity and only on MELS for peer problems, possibly due to higher stability in female speech features [40].

For emotional symptoms, MFCC + PROS was the optimal combination for both genders, consistent with previous findings that combining spectral (MFCC) and prosodic (PROS) features captures speech changes associated with emotional states [41]. Depression and anxiety, for instance, may lead to slower speech rate, lower volume, and flattened pitch [42, 43].

For conduct problems, the gender difference in the best feature combination was the most pronounced (PROS for males and MFCC + FORM for females). This gender difference may stem from differences in the clinical manifestation of conduct problems, with males more likely to exhibit direct physical aggression and females more likely to exhibit relational aggression [44]. This results in anomalies in prosodic features (e.g., dramatic pitch variation) for males, while females show anomalies in spectral and formant features [45].

Notably, the relatively lower AUC values observed for conduct problems (0.62) and peer problems (0.58) in this study may stem from the interplay between clinical complexity and methodological constraints. On one hand, the context-dependent nature of conduct problems (e.g., transient vocal changes during aggressive outbursts that escape detection by SDQ's low-frequency event sensitivity) [46] and the implicit characteristics of peer problems (requiring longitudinal social dynamics assessment, with SDQ AUC as low as 0.49 in cross-cultural validations) [47] interact with inherent limitations of static measurement scales (particularly SDQ's inadequate cross-cultural sensitivity for peer relationship evaluation) [48], collectively contributing to ambiguous vocal-label mapping for these behavioral dimensions.

On the other hand, the ecological validity mismatch between laboratory-controlled vocal tasks and SDQ's 6-month behavioral recall framework might underlie the insufficient characterization of externalizing behaviors (e.g., abrupt vocal interruptions) and social cognition (e.g., conversational turn-taking patterns) through conventional acoustic features (MFCC/fundamental frequency) [49]. This temporal and contextual discontinuity potentially introduces systematic bias in feature-behavior correlations.

This study also found that the GBDT model outperformed others in all symptom dimensions and required the least feature combination for optimal prediction. This may be because GBDT builds decision trees iteratively, automatically selecting the optimal features and identifying key predictive factors [50].

### 4.2. Clinical Implications

This study leverages SHAP analysis to identify abnormal speech feature patterns across different symptoms and genders, leading to the development of a novel and user-friendly speech-based assessment tool. By utilizing this predictive tool, clinicians can accurately evaluate emotional and behavioral problems by selecting appropriate speech features tailored to the patient's gender and specific symptoms. For hyperactivity, the tool identifies male symptoms primarily through MELS and MFCC features, such as significant volume fluctuations and rapid speech rate, aligning with clinical observations of males exhibiting “fast and loud” speech patterns [24, 51]. In contrast, female hyperactivity is detected mainly through MFCC features, characterized by increased speech rate and higher pitch [52], underscoring the need for gender-specific evaluation criteria. Regarding emotional symptoms, the tool associates male emotional issues with abnormalities in fundamental frequency (F0) features, indicating a general decline in pitch during low emotional states [53]. For females, emotional problems are more accurately reflected through changes in MFCC features, such as alterations in vocal timbre and clarity [54]. This differentiation facilitates the creation of targeted intervention strategies based on gender-specific vocal indicators. Behavioral problems also exhibit significant gender differences in speech features; males are primarily identified through prosodic features (PROS) abnormalities, marked by dramatic volume variations that reflect internal impulsiveness and emotional instability [45]. Conversely, female behavioral issues are linked to anomalies in MFCC and FORM features, potentially associated with a tendency towards indirect aggressive behaviors [44]. The predictive performance for peer relationship problems was relatively weak; however, differences in feature performance between males and females suggest that abnormalities in vocal energy distribution during social interactions may play a role [55]. This indicates the necessity for more nuanced assessments that consider gender differences to enhance evaluation accuracy. Implementing this structured speech feature assessment tool allows clinicians to more precisely identify emotional and behavioral problems across different genders and symptoms, thereby optimizing intervention strategies and resource allocation. Additionally, SHAP case heatmap analysis revealed opposing effects of certain features within the predictive model. For instance, in predicting hyperactivity, the skewness of MELS 24 in males had a negative effect, while the mean of MFCC 10 had a positive effect, potentially canceling each other out and reducing prediction accuracy. A similar pattern was observed for emotional symptoms, where the kurtosis of F0's second derivative had a negative impact, and the maximum of F0's first derivative had a positive effect. These complex interactions highlight the importance of using complementary assessment tools, such as behavioral observations and psychological scales, to mitigate the risk of misjudgments that may arise from relying solely on speech features. Future research should focus on externally validating this speech-based assessment tool in larger and more diverse cohorts to ensure its effectiveness across different populations. Additionally, determining the most cost- and resource-effective cutoffs in various clinical settings will be essential for the practical implementation of this tool. By addressing these areas, the tool can significantly enhance the reliability and effectiveness of clinical assessments for emotional and behavioral problems.

For Chinese medical students who face unique mental health challenges, this speech-based assessment tool offers several distinctive advantages. First, it addresses the prevalent issue of mental health stigma in this population [56] by providing an objective assessment method that circumvents the reluctance to self-report symptoms. Second, the tool's ability to detect subclinical symptoms through subtle speech changes [57] is particularly valuable for identifying at-risk students during the preclinical phase, when traditional screening often fails. Third, the gender-specific feature analysis accounts for cultural differences in emotional expression among Chinese medical students, where males may exhibit more somatization and females tend toward internalizing symptoms [58]. Implementation could leverage China's advanced telemedicine infrastructure by integrating the tool into: (1) routine health checkups at medical universities, (2) student psychological service platforms, and (3) mobile apps for continuous monitoring during high-stress periods like national licensing exam preparation. This approach would overcome barriers posed by heavy academic workloads and limited mental health resources in Chinese medical education settings.

### 4.3. Contributions, Limitations, and Future Directions

This study, the first to systematically use speech features to predict and diagnose SDQ-assessed emotional and behavioral problems, presents the following innovations: (1) It investigated the predictive utility of speech features for multidimensional emotional and behavioral problems in a large sample of adolescents, expanding the application of speech technology in mental health assessment. (2) It compared differences in speech prediction patterns across different symptoms and genders, revealing the complexity and specificity of speech-emotion associations. (3) It innovatively combined machine learning and SHAP interpretation methods to achieve efficient prediction and interpretation.

However, this study has some limitations. First, the cross-sectional design cannot establish causal relationships between speech abnormalities and emotional or behavioral problems. Second, the classification of emotional and behavioral problems is broad, which may affect prediction accuracy, especially for conduct and peer relationship problems, which require further analysis to improve differentiation. Third, while we adopted a hierarchical analytic strategy by partitioning 378 speech features into four acoustic categories (PROS, MFCC, MELS, and FORM) and evaluating 15 combinatorial subsets (ranging from 42 to 378 features), the inherent tradeoff between exploratory discovery and statistical rigor warrants attention. Traditional Bonferroni correction (e.g., *α* = 0.05/378 ≈ 1.32 × 10^−4^ for the full combinatorial set) would retain only 0–3 significant features, potentially obscuring clinically relevant biomarkers. This methodological approach reflects established practices in speech emotion analytics—as exemplified by Dibben et al. [59] in Nature Human Behaviour when handling high-dimensional speech features with substantial inter-correlation (Pearson's *r* = 0.65–0.89)—we explicitly report all effect sizes (Cohen's *d*) and raw *p*-value distributions to facilitate future replication in Supporting Information 1: Appendix B. Additionally, the cultural background of the sample may limit the generalizability of the results, requiring validation in different cultural contexts.

Future research could combine technologies such as brain imaging to further reveal the relationship between speech abnormalities and brain activity and evaluate the effectiveness of combining speech technology with traditional methods in real-world settings. Multidimensional validation strategies should prioritize: (1) longitudinal designs to track biomarker stability, (2) multimodal fusion with physiological signals (e.g., heart rate and EEG) for enhanced specificity, and (3) cross-cultural adaptation of acoustic thresholds based on linguistic prosody patterns, and (4) systematic integration of textual and semantic features to capture paralinguistic-behavioral synergies.

## 5. Conclusion

This study is the first to use speech features and machine learning to predict emotional and behavioral problems in adolescents, demonstrating speech's potential as a biomarker. Analyzing data from 8215 adolescents, the GBDT model accurately identified hyperactivity and emotional symptoms, revealing gender-specific feature patterns. While the current model focuses on these dimensions, future work could further enhance the prediction of peer problems and conduct problems by integrating semantic and contextual speech analysis. The developed model provides an objective tool for early detection, enabling targeted interventions by healthcare systems. Future research should validate this speech-based assessment tool with larger and more diverse populations to improve its effectiveness and applicability across broader behavioral domains.

## Figures and Tables

**Table 1:** Classification, feature distribution, and mental health relevance of key speech categories.

Main category	Subcategory	Number of features (function)	Significance in mental health assessment
MFCC	MFCC (1–13)	91 (Min/Max, range, mean, Std Dev, skewness, kurtosis)	Reflects changes in the speech spectral envelope, associated with emotional states and cognitive load

PROS	F0	21 (Min/Max, range, mean, Std Dev, skewness, kurtosis)	Represents pitch variation, indicative of emotional arousal and stress levels
	Energy	21 (Min/Max, range, mean, Std Dev, skewness, kurtosis)	Describes speech loudness, reflecting emotional intensity and patterns of social interaction

FORM	F1	21 (Min/Max, range, mean, Std Dev, skewness, kurtosis, delta/delta–delta)	Related to vowel openness, indicating emotional expression and speech clarity
	F2	21 (Min/Max, range, mean, Std Dev, skewness, kurtosis, delta/delta–delta)	Reflects the anterior–posterior movement of the tongue, revealing speech fluency and emotional expression capabilities
	F3	21 (Min/Max, range, mean, Std Dev, skewness, kurtosis, delta/delta–delta)	Associated with vocal tract shape, reflecting voice quality and subtle emotional changes

MELS	MELS (1–26)	182 (Min/Max, range, mean, Std Dev, skewness, kurtosis)	Simulates human auditory perception, capturing subtle differences in emotional changes and psychological states

Total	—	378	—

**Table 2:** All possible combinations of speech feature categories for model training and evaluation.

No.	Combination	No.	Combination	No.	Combination
1	PROS	2	MFCC	3	FORM
4	MELS	5	MFCC + PROS	6	MFCC + FORM
7	MFCC + MELS	8	PROS + FORM	9	PROS + MELS
10	FORM + MELS	11	MFCC + PROS + FORM	12	MFCC + PROS + MESL
13	MFCC + FORM + MESL	14	PROS + FORM + MELS	15	MFCC + PROS + FORM + MELS

**Table 3:** Optimized parameters of the predictive models.

Models	Hyperparameter	Candidate range
LR	Regularization (*C*)	[0.001, 0.01, 0.1, 1, 10, 100]
	Penalty type	[‘l2', ‘l1', ‘elasticnet']
	Solver	[‘lbfgs', ‘liblinear', ‘sag']
	Max iterations	[100, 500, 1000]

SVM	*C*	[0.1, 1, 10, 100]
	Kernel	[‘linear', ‘rbf']
	Gamma	[‘scale', 0.01, 0.1, 1]
	Shrinking	[True, False]

GBDT	n_estimators	[50, 100, 200, 500]
	learning_rate	[0.005, 0.05, 0.1, 0.2]
	max_depth	[3, 5, 7, 10]
	min_samples_split	[20, 50, 100]
	min_samples_leaf	[10, 20, 30]
	subsample	[0.6, 0.8, 1.0]

**Table 4:** Representative speech feature differences across behavioral problem dimensions in male and female groups.

Dimension	Gender	Feature	Problematic *M* (SD)	Nonproblematic *M* (SD)	*p*-Value	Cohen's *d*
Hyperactivity	Male	F1 mean	993.39 (388.15)	1072.80 (465.41)	<0.001	−0.19
		F3 Std	1217.86 (192.35)	1183.97 (216.10)	<0.001	0.17
	Female	MELS 5 Std	52.01 (7.26)	50.54 (7.72)	<0.001	0.2
		MELS 18 Std	71.71 (6.84)	70.49 (6.98)	<0.001	0.18

Emotional symptoms	Male	F3 2nd derivative max	6348.35 (1884.51)	6018.80 (2029.42)	<0.001	0.17
		F1 mean	1007.62 (397.85)	1075.44 (469.16)	<0.001	−0.16
	Female	MELS 5 Std	52.01 (7.26)	50.54 (7.72)	<0.001	0.2
		MELS 18 Std	71.71 (6.84)	70.49 (6.98)	<0.001	0.18

Conduct problems	Male	F1 mean	1012.84 (416.84)	1094.29 (477.56)	<0.001	−0.18
		F1 Std	880.59 (245.69)	919.95 (260.90)	<0.001	−0.16
	Female	F1 mean	1020.42 (426.41)	1099.80 (478.65)	<0.001	−0.18
		F3 1st derivative min	−2034.35 (462.71)	−1949.29 (504.65)	<0.001	−0.18

Peer problems	Male	MELS 3 kurtosis	0.47 (0.44)	0.42 (0.39)	<0.001	0.11
		MELS 20 mean	0.04 (1.14)	−0.06 (1.22)	0.0061	0.08
	Female	MFCC 6 Std	143.40 (22.16)	140.11 (23.17)	<0.001	0.15
		MELS 6 minimum	−184.41 (42.27)	−178.26 (41.44)	<0.001	−0.15

**Table 5:** Detailed performance of the best feature subspace for GBDT models across different symptoms and genders.

Dimension	Gender	Best feature combination	AUC (Std)	F1 score (Std)	Sensitivity (Std)	Specificity (Std)
Hyperactivity	Male	MFCC + MELS	0.783 ± 0.011	0.725 ± 0.010	0.763 ± 0.020	0.659 ± 0.021
	Female	MFCC	0.780 ± 0.016	0.722 ± 0.019	0.764 ± 0.027	0.648 ± 0.027

Emotional symptoms	Male	MFCC + PROS	0.738 ± 0.007	0.686 ± 0.010	0.710 ± 0.017	0.642 ± 0.012
	Female	MFCC + PROS	0.658 ± 0.018	0.632 ± 0.014	0.662 ± 0.017	0.567 ± 0.023

Conduct problems	Male	PROS	0.597 ± 0.012	0.600 ± 0.011	0.635 ± 0.015	0.517 ± 0.022
	Female	MFCC + FORM	0.622 ± 0.028	0.599 ± 0.020	0.621 ± 0.024	0.549 ± 0.028

Peer problems	Male	MFCC + MELS + PROS	0.626 ± 0.015	0.562 ± 0.017	0.534 ± 0.030	0.635 ± 0.025
	Female	MELS	0.571 ± 0.013	0.534 ± 0.019	0.510 ± 0.027	0.602 ± 0.030

**Table 6:** Maximized Youden's index values across feature-specific optimal models.

Dimension	Gender	Best feature combination	Youden's index
Hyperactivity	Male	MFCC + MELS	0.409
	Female	MFCC	0.412

Emotional symptoms	Male	MFCC + PROS	0.352
	Female	MFCC + PROS	0.201

Conduct problems	Male	PROS	0.152
	Female	MFCC + FORM	0.170

Peer problems	Male	MFCC + MELS + PROS	0.186
	Female	MELS	0.112

## Data Availability

The data that support the findings of this study are available upon request from the corresponding author. The data are not publicly available due to privacy or ethical restrictions.

## Supporting Information

Supporting Information 1 The supporting information file includes all additional figures and tables referenced in the main text as Appendix A–G.

Supporting Information 2 “STARD-2015-Checklist.docx” is provided to confirm adherence to diagnostic reporting standards.

## References

[1] Memmott-Elison M. K., Toseeb U. (2023). Prosocial Behavior and Psychopathology: An 11-Year Longitudinal Study of Inter- and Intraindividual Reciprocal Relations Across Childhood and Adolescence. *Development and Psychopathology*.

[2] Osman S., Khalaf S., Omar M., Ismail T. A. (2019). Behavioral and Emotional Problems Among Adolescent Students. *Journal of High Institute of Public Health*.

[3] Georgoulas N. Behavioral Disorders in Children.

[4] Palo H., Chandra M., Mohanty M., Satapathy S. C., Das S. Recognition of Human Speech Emotion Using Variants of Mel-Frequency Cepstral Coefficients.

[5] Pakyurek M., Atmis M., Kulaç S., Uludağ U. (2020). Extraction of Novel Features Based on Histograms of MFCCs Used in Emotion Classification From Generated Original Speech Dataset. *Elektronika ir Elektrotechnika*.

[6] Lalitha S., Tripathi S., Gupta D. (2019). Enhanced Speech Emotion Detection Using Deep Neural Networks. *International Journal of Speech Technology*.

[7] Lima L., Behlau M. (2021). Emotional/Behavioral Indicators in Children and Adolescents With and Without Vocal Problems: Self-Evaluation and Parental Evaluation. *Journal of Voice*.

[8] Strand P. S., Barbosa-Leiker C., Piedra M. A., Downs A. M. (2015). Exploring the Bidirectionality of Emotion Understanding and Classroom Behavior With Spanish- and English-Speaking Preschoolers Attending Head Start. *Social Development*.

[9] Glogowska M., Roulstone S., Peters T. J., Enderby P. (2006). Early Speech- and Language-Impaired Children: Linguistic, Literacy, and Social Outcomes. *Developmental Medicine & Child Neurology*.

[10] Westrupp E. M., Reilly S., McKean C., Law J., Mensah F., Nicholson J. M. (2020). Vocabulary Development and Trajectories of Behavioral and Emotional Difficulties via Academic Ability and Peer Problems. *Child Development*.

[11] Aro T., Eklund K., Eloranta A.-K., Ahonen T., Rescorla L. (2022). Learning Disabilities Elevate Children’s Risk for Behavioral-Emotional Problems: Differences Between LD Types, Genders, and Contexts. *Journal of Learning Disabilities*.

[12] Konac D., Young K. S., Lau J., Barker E. D. (2021). Comorbidity Between Depression and Anxiety in Adolescents: Bridge Symptoms and Relevance of Risk and Protective Factors. *Journal of Psychopathology and Behavioral Assessment*.

[13] Ghandour R. M., Sherman L. J., Vladutiu C. J. (2019). Prevalence and Treatment of Depression, Anxiety, and Conduct Problems in US Children. *The Journal of Pediatrics*.

[14] Weller B. E., Blanford K. L., Butler A. M. (2018). Estimated Prevalence of Psychiatric Comorbidities in U.S. Adolescents With Depression by Race/Ethnicity, 2011–2012. *Journal of Adolescent Health*.

[15] Low D. M., Bentley K. H., Ghosh S. S. (2020). Automated Assessment of Psychiatric Disorders Using Speech: A Systematic Review. *Laryngoscope Investigative Otolaryngology*.

[16] Vabalas A., Gowen E., Poliakoff E., Casson A. J. (2019). Machine Learning Algorithm Validation With a Limited Sample Size. *PLoS ONE*.

[17] Zhang R., Wang Y., Womer F. (2023). School-Based Evaluation Advancing Response for Child Health (SEARCH): A Mixed Longitudinal Cohort Study From Multifaceted Perspectives in Jiangsu, China. *BMJ Mental Health*.

[18] Mascha E. J., Vetter T. R. (2018). Significance, Errors, Power, and Sample Size: The Blocking and Tackling of Statistics. *Anesthesia & Analgesia*.

[19] Kang H. (2021). Sample Size Determination and Power Analysis Using the G^∗^Power Software. *Journal of Educational Evaluation for Health Professions*.

[20] Zhao L., Li Y., Wang Z., Wu J. (2024). Validation of the Chinese Version of the Adverse Life Experiences Scale. *Frontiers in Pediatrics*.

[21] Yao S., Zhang C., Zhu X., Jing X., McWhinnie C. M., Abela J. R. Z. (2009). Measuring Adolescent Psychopathology: Psychometric Properties of the Self-Report Strengths and Difficulties Questionnaire in a Sample of Chinese Adolescents. *Journal of Adolescent Health*.

[22] Goodman R. (1997). The Strengths and Difficulties Questionnaire: A Research Note. *Journal of Child Psychology and Psychiatry*.

[23] Eyben F., Weninger F., Groß F., Schuller B. Recent Developments in openSMILE, the Munich Open-Source Multimedia Feature Extractor.

[24] Bhimavarapu J. P., Sarvana K., Achanta V., Kadiyala C., Yadhavkareti C. Modelling of Emotion Recognition System from Speech Using MFCC Features.

[25] Skuratovskii R., Bazarna A., Osadchy E. (2021). Analysis of Speech MEL Scale and Its Classification as Big Data by Parameterized KNN. *Journal of Automatic Information Science*.

[26] Fishman B., Opher I. Prosodic Feature Criterion for Hebrew Using Different Feature Sets.

[27] Bhanja C. C., Laskar M. A., Laskar R., Karpov A., Potapova R., Mporas I. (2019). Formants and Prosody-Based Automatic Tonal and Non-Tonal Language Classification of North East Indian Languages. *Speech and Computer*.

[28] Modarres R. (2022). Nonparametric Tests for Detection of High Dimensional Outliers. *Journal of Nonparametric Statistics*.

[29] Singh D., Singh B. (2020). Investigating the Impact of Data Normalization on Classification Performance. *Applied Soft Computing*.

[30] Abia V. M., Johnson E. H. (2024). Sentiment Analysis Techniques: A Comparative Study of Logistic Regression, Random Forest, and Naive Bayes on General English and Nigerian Texts. *Journal of Engineering Research and Reports*.

[31] Vezzoli M., Zogmaister C. (2023). An Introductory Guide for Conducting Psychological Research With Big Data. *Psychological Methods*.

[32] Chi M. Optimization of Prediction Model Based on Gradient Boosting Decision Tree.

[33] Fernández A., García S., Herrera F., Chawla N. V. (2018). SMOTE for Learning from Imbalanced Data: Progress and Challenges, Marking the 15-Year Anniversary. *Journal of Artificial Intelligence Research*.

[34] Tislenko M. D., Gaidel A. V., Kupriyanov A. V. Comparison of Feature Selection Algorithms for Data Classification Problems.

[35] Egaji O. A., Evans G., Griffiths M. G., Islas G. (2021). Real-Time Machine Learning-Based Approach for Pothole Detection. *Expert Systems with Applications*.

[36] Nohara Y., Matsumoto K., Soejima H., Nakashima N. Explanation of Machine Learning Models Using Improved Shapley Additive Explanation.

[37] Berisha V., Sandoval S., Utianski R., Liss J., Spanias A. Selecting Disorder-Specific Features for Speech Pathology Fingerprinting.

[38] Hanson D. M., Jackson A. W., Hagerman R. J., Hagerman P. J., Opitz J. M., Reynolds J. F. (1986). Speech Disturbances (Cluttering) in Mildly Impaired Males with the Martin-Bell/Fragile X Syndrome. *American Journal of Medical Genetics*.

[39] Murray-Close D., Hoza B., Hinshaw S. P. (2010). Developmental Processes in Peer Problems of Children With Attention-Deficit/Hyperactivity Disorder in the Multimodal Treatment Study of Children With ADHD: Developmental Cascades and Vicious Cycles. *Development and Psychopathology*.

[40] Li L., Zheng T. Gender-Dependent Feature Extraction for Speaker Recognition.

[41] Koo H., Jeong S., Yoon S., Kim W. Development of Speech Emotion Recognition Algorithm Using MFCC and Prosody.

[42] Cummins N., Sethu V., Epps J., Schnieder S., Krajewski J. (2015). Analysis of Acoustic Space Variability in Speech Affected by Depression. *Speech Communication*.

[43] Afshan A., Guo J., Park S., Ravi V., Flint J., Alwan A. Effectiveness of Voice Quality Features in Detecting Depression.

[44] Crick N. R., Grotpeter J. K. (1995). Relational Aggression, Gender, and Social-Psychological Adjustment. *Child Development*.

[45] Kawai T., Suzuki Y., Hatanaka C., Konakawa H., Tanaka Y., Uchida A. (2020). Gender Differences in Psychological Symptoms and Psychotherapeutic Processes in Japanese Children. *International Journal of Environmental Research and Public Health*.

[46] Goodman R., Ford T., Simmons H., Gatward R., Meltzer H. (2003). Using the Strengths and Difficulties Questionnaire (SDQ) to Screen for Child Psychiatric Disorders in a Community Sample. *International Review of Psychiatry*.

[47] Stone L. L., Otten R., Engels R. C. M. E., Vermulst A. A., Janssens J. M. A. M. (2010). Psychometric Properties of the Parent and Teacher Versions of the Strengths and Difficulties Questionnaire for 4- to 12-Year-Olds: A Review. *Clinical Child and Family Psychology Review*.

[48] Rimfeld Kaili, Malanchini Margherita, Arathimos Ryan (2022). The Consequences of a Year of the COVID-19 Pandemic for the Mental Health of Young Adult Twins in England and Wales. *BJPsych Open*.

[49] Goodman A., Lamping D. L., Ploubidis G. B. (2010). When to Use Broader Internalising and Externalising Subscales Instead of the Hypothesised Five Subscales on the Strengths and Difficulties Questionnaire (SDQ): Data From British Parents, Teachers and Children. *Journal of Abnormal Child Psychology*.

[50] Zhang Z., Jung C. (2021). GBDT-MO: Gradient-Boosted Decision Trees for Multiple Outputs. *IEEE Transactions on Neural Networks and Learning Systems*.

[51] Jayawardena G., Michalek A., Duchowski A. T., Jayarathna S. Pilot Study of Audiovisual Speech-in-Noise (SIN) Performance of Young Adults with ADHD.

[52] Suárez I., Aragón C., Grandjean A. (2021). Two Sides of the Same Coin: ADHD Affects Reactive but not Proactive Inhibition in Children. *Cognitive Neuropsychology*.

[53] Fraas M., Solloway M. (2004). The Effects of Complementary and Alternative Medicine on the Speech of Patients With Depression. *Journal of the Acoustical Society of America*.

[54] Lausen A., Schacht A. (2018). Gender Differences in the Recognition of Vocal Emotions. *Frontiers in Psychology*.

[55] Almaghrabi S. A., Thewlis D., Thwaites S. (2022). The Reproducibility of Bio-Acoustic Features is Associated With Sample Duration, Speech Task, and Gender. *IEEE Transactions on Neural Systems and Rehabilitation Engineering*.

[56] Ryder Andrew G., Sun Jiahong, Zhu Xiongzhao, Yao Shuqiao, Chentsova-Dutton Yulia E. (2012). Depression in China: Integrating Developmental Psychopathology and Cultural-Clinical Psychology. *Journal of Clinical Child & Adolescent Psychology*.

[57] Ryder Andrew G., Zhao Yue, Chentsova-Dutton Yulia E., DeRubeis Robert J., Strunk Daniel R. (2017). Disordered Mood in Cultural-Historical Context. *The Oxford Handbook of Mood Disorders*.

[58] Zhang N., Ren X., Xu Z., Zhang K. (2024). Gender Differences in the Relationship between Medical Students’ Emotional Intelligence and Stress Coping: A Cross-Sectional Study. *BMC Medical Education*.

[59] Dibben N., Coutinho E., Vilar Jé A., Estévez-Pérez G. (2018). Do Individual Differences Influence Moment-by-Moment Reports of Emotion Perceived in Music and Speech Prosody?. *Frontiers in Behavioral Neuroscience*.

