# Supplementary material for: Using Speech Features and Machine Learning Models to Predict Emotional and Behavioral Problems in Chinese Adolescents
Source: Depress Anxiety. 2025 Jun 16;2025:5734107. doi: 10.1155/da/5734107 (PMC12185205; doi:10.1155/da/5734107)
Supplement: Supporting Information 1 — The supporting information file includes all additional figures and tables referenced in the main text as Appendix A–G. [file 5734107.f1.zip › Appendix A.pdf]

Appendix A

English Version:

Section 1: Questionnaires

Table 1-1 General Information (Student Version)

A. Basic Information

No.	Survey Item	Options
A01	Ethnicity	1.Han 2.Hui 3.Miao<br>4. Tujia<br>5. Other (specify____)

B. Family and Living Situation

No.	Survey Item	Options
B01	Do you live on campus?	1. Yes 2. No
B02	In the past six months, how many family members have you been living with?	____ people
B03	They include (multiple choices)	1. Grandparents<br>2. Father<br>3. Mother<br>4. Stepfather<br>5. Stepmother<br>6. Siblings<br>7. Other
B04	Are you an only child?	1. Yes (skip to B06) 2. No

**B05**	**If not an only child, do you feel your parents have any favoritism?**	**1. Favor siblings<br>2. No favoritism<br>3. Favor me**
**B06**	**Frequency of parental arguments**	**1.Never 2. Sometimes 3. Often**

C. Academic Performance

No.	Survey Item	Options
**C01**	**In the most recent major exam (mid-term or final), your class rank is approximately**	**1. Top 10%<br>2. 11-25%<br>3. 26-50%<br>4. 51-75%<br>5. Bottom 25%<br>6. School does not release rankings**
**C02**	**Compared to last semester, changes in your academic performance this semester**	**1. Improved 2. Declined 3. Stable**
**C03**	**Compared to last semester, changes in your mood this semester**	**1.Better 2. Worse 3. No change**
**C04**	**Have you ever served as a student leader in your class or school?**	**1. No 2. Yes**

Table 1-2 Mood Questionnaire (Depression Anxiety Stress Scales)

[Instructions]: Please read each statement carefully and select the option that best describes your experience over the past week. Please answer every item, there are no right or wrong answers. Now let's begin!

D. Mood

No.	In the past week	Did not apply to me at all	Applied to me to some degree, or some of the time	Applied to me a considerable degree, or a good part of time	Applied to me very much, or most of the time
D01	I found it hard to wind down.	0	1	2	3
D02	I was aware of dryness of my mouth.	0	1	2	3
D03	I couldn't seem to experience any positive feeling at all.	0	1	2	3
D04	I experienced breathing difficulty (e.g., excessively rapid breathing, breathlessness in the absence of physical exertion).	0	1	2	3
D05	I found it difficult to work up the initiative to do things.	0	1	2	3

**D06**	**I tended to over-react to situations.**	**0**	**1**	**2**	**3**
**D07**	**I experienced trembling (e.g., in the hands).**	**0**	**1**	**2**	**3**
**D08**	**I felt that I was using a lot of nervous energy.**	**0**	**1**	**2**	**3**
**D09**	**I was worried about situations in which I might panic and make a fool of myself.**	**0**	**1**	**2**	**3**
**D10**	**I felt that I had nothing to look forward to.**	**0**	**1**	**2**	**3**
**D11**	**I found myself getting agitated.**	**0**	**1**	**2**	**3**
**D12**	**I found it difficult to relax.**	**0**	**1**	**2**	**3**
**D13**	**I felt down-hearted and blue.**	**0**	**1**	**2**	**3**
**D14**	**I was intolerant of anything that kept me from getting on with what I was doing.**	**0**	**1**	**2**	**3**
**D15**	**I felt I was close to panic.**	**0**	**1**	**2**	**3**
**D16**	**I was unable to become enthusiastic about anything.**	**0**	**1**	**2**	**3**
**D17**	**I felt I wasn't worth much as a person.**	**0**	**1**	**2**	**3**
**D18**	**I felt that I was rather touchy.**	**0**	**1**	**2**	**3**
**D19**	**I was aware of the action of my heart**	**0**	**1**	**2**	**3**

	**in the absence of physical exertion (e.g., sense of heart rate increase, heart missing a beat).**				
**D20**	**I felt scared without any good reason.**	**0**	**1**	**2**	**3**
**D21**	**I felt that life was meaningless.**	**0**	**1**	**2**	**3**

Table 1-3 Sleep Questionnaire (Student Version)

[Instructions]: The following questions assess your sleep situation. For each question, choose the answer that best describes you. Now let's begin!

No.	Survey Item	None	Mild	Moderate	Severe	Very Severe
H01	Please rate the severity of your sleep problem(s) in the past two weeks:1）Difficulty falling asleep	0	1	2	3	4
H02	2）Difficulty staying asleep	0	1	2	3	4
H03	3）Waking up too early	0	1	2	3	4
No.	Survey Item	(0 indicates very satisfied, 4 indicates very dissatisfied)				
H04	How satisfied/dissatisfied are you with your sleep pattern over the past two weeks?	0	1	2	3	4
No.	Survey Item	Not at all interfering	A little interfering	Somewhat interfering	Much interfering	Very much interfering
H05	In the past two weeks, to what extent do you consider your sleep problem(s) to interfere with your daily functioning (e.g., energy, concentration, memory,	0	1	2	3	4

	**mood)?**					
**No.**	**Survey Item**	**Not at all noticeable**	**Barely noticeable**	**Somewhat noticeable**	**Much noticeable**	**Very much noticeable**
**H06**	**Over the past two weeks, how noticeable to others do you think your sleep problem has been in terms of impairing the quality of your life?**	**0**	**1**	**2**	**3**	**4**
**No.**	**Survey Item**	**Not at all worried**	**A little worried**	**Somewhat worried**	**Much worried**	**Very much worried**
**H07**	**How worried/distressed are you about your current sleep problem over the past two weeks?**	**0**	**1**	**2**	**3**	**4**

Table 1-4 Strengths and Difficulties Questionnaire (Student Version)

[Instructions]: Please answer the following questions based on your experience and reality over the past six months. Select the appropriate answer from the three options for each item: "Not True", "Somewhat True", "Certainly True". Please do not skip any questions, now let's begin!

No.	In the past 6 months	Not	Somewhat	Certainly

		True	True	True
E01	I try to be nice to other people. I care about their feelings	0	1	2
E02	I am restless, I cannot stay still for long	0	1	2
E03	I get a lot of headaches, stomach-aches or sickness	0	1	2
E04	I usually share with others (food, games, pens etc.)	0	1	2
E05	I get very angry and often lose my temper	0	1	2
E06	I am usually on my own. I generally play alone	0	1	2
E07	I usually do as I am told	2	1	0
E08	I worry a lot	0	1	2
E09	I am helpful if someone is hurt, upset or feeling ill	0	1	2
E10	I am constantly fidgeting or squirming	0	1	2
E11	I have one good friend or more	2	1	0
E12	I fight a lot. I can make other people do what I want	0	1	2
E13	I am often unhappy, down-hearted or tearful	0	1	2
E14	Other people my age generally like me	2	1	0
E15	I am easily distracted, I find it difficult to concentrate	0	1	2
E16	I am nervous in new situations. I easily lose confidence	0	1	2
E17	I am kind to younger children	0	1	2
E18	I am often accused of lying or cheating	0	1	2
E19	Other children or young people pick on me or bully me	0	1	2
E20	I often volunteer to help others (parents, teachers, children)	0	1	2
E21	I think before I do things	2	1	0
E22	I take things that are not mine from home, school or elsewhere	0	1	2
E23	I get on better with adults than with people my own age	0	1	2
E24	I have many fears, I am easily scared	0	1	2
E25	I finish the work I'm doing. My attention is good	2	1	0

[Instructions]: Next, do you have any other opinions or thoughts?

Additional Questions

No.	Survey Item	Options
F01	Overall, do you think you have difficulties in your emotions, concentration, behavior or being able to get on with others?	1. No (skip to G01)<br>2. Yes (minor difficulties)<br>3. Yes (definite difficulties)<br>4. Yes (severe difficulties)
F02	How long have these difficulties been present?	1. Less than a month<br>2. 1-5 months<br>3. 6-11 months<br>4. A year or more
F03	Do the difficulties upset or distress you?	1. Not at all<br>2. A little<br>3. Quite a lot<br>4. A great deal
F04	Do the difficulties interfere with your peer relationships?	1. Not at all<br>2. A little<br>3. Quite a lot<br>4. A great deal
F05	Do the difficulties interfere with your classroom learning?	1. Not at all<br>2. A little<br>3. Quite a lot<br>4. A great deal
F06	Do the difficulties put a burden on your	1. Not at all

	**family, friends or teachers?**	**2. A little**<br>**3. Quite a lot**<br>**4. A great deal**

Section 2: Survey Content Explanation

1.Table 1-1 General Information (Student Version)

A.Basic Information: Item A01

A01 Ethnicity: Should match the information on Resident ID Card/Household Registration Book.

B.Family and Living Situation: Items B01-B06

B01 Living on Campus: Refers to living in school dormitory for at least 3 days per week this semester.

B02～B03 Family Members: Reflects family structure - extended family, nuclear family, single-parent family, blended family.

Father and mother refer to biological parents and adoptive parents.

Nuclear family: Only select father and mother.

Extended family: Select grandparents, father and mother.

Single-parent family: Only select either father or mother.

Blended family: Select father and stepmother; or mother and stepfather.

Skipped-generation family: Select grandparents, without father/mother/stepfather/stepmother.

B04 Whether the student is an only child.

B05 Reflects whether non-only children feel parental favoritism at home.

B06 Reflects frequency of parental arguments (never, sometimes, often). **C.Academic Performance: Items C01-C04**

C01 Reflects the student's class ranking in the most recent major exam (mid-term or

final) - top 10%, 11-25%, 26-50%, 51-75%, bottom 25%, school does not release rankings.

C02 Reflects changes in the student's academic performance compared to last semester (improved, declined, stable).

C03 Reflects changes in the student's mood compared to last semester (better, worse, no change).

C04 Reflects whether the student has served as a student leader in class or school.

2.Table 1-2 Mood Questionnaire: Items D01-D21

(DASS-21) was developed by Lovibond et al. in 1995. It is a self-report scale used to assess depression, anxiety and stress. Originally used for normal adults, it was later revised by Antony et al. to create the simplified version DASS-21. Researchers began trying to expand its research subjects to children, adolescents and even the elderly, and use it in clinical settings.

The scale contains 21 items, with 7 items each for the three subscales of depression, anxiety and stress. All use a 4-point scale from "0" (did not apply to me at all) to "3" (applied to me very much, or most of the time). Multiply the score of each subscale by 2 to get the subscale score. Higher scores indicate higher levels of that emotion.

Factors and corresponding items:

Stress subscale items: D01, D06, D08, D11, D12, D14, D18

Anxiety subscale items: D02, D04, D07, D09, D15, D19, D20

Depression subscale items: D03, D05, D10, D13, D16, D17, D21

Cut-off criteria:

Depression subscale: ≤9 normal, 10-13 mild, 14-20 moderate, 21-27 severe, ≥28 extremely severe

Anxiety subscale: ≤7 normal, 8-9 mild, 10-14 moderate, 15-19 severe, ≥20 extremely

severe

Stress subscale: ≤14 normal, 15-18 mild, 19-25 moderate, 26-33 severe, ≥34 extremely severe

3.Table 1-4 Strengths and Difficulties Questionnaire: Items E01-F06

The Strengths and Difficulties Questionnaire (SDQ) is a measurement tool for assessing emotional and behavioral problems in children and adolescents. It was developed by British psychologist Robert Goodman based on the ICD-10, DSM-IV and Rutter's child behavior questionnaire. As some SDQ items are consistent with the diagnostic criteria for emotional disorders, conduct disorders, attention deficit and hyperactivity in ICD-10 and DSM-IV, the SDQ has diagnostic features in screening for child mental disorders and evaluating intervention effects. In addition, the strengths section of the SDQ focuses on the positive behaviors of children and adolescents, changing the previous psychological health assessment tools that only focused on negative behaviors, making the assessment more comprehensive. Its two-dimensional structure also facilitates horizontal comparison with patients with emotional and behavioral disorders, and pre-post tests can be used to evaluate intervention effects. The SDQ includes student, parent and teacher versions, with basically the same item content across the three versions, only with slight wording differences. All three versions have good reliability and validity.

The scale contains 25 items and 6 additional questions. Add the scores according to the item numbers to calculate the score for each factor. The total difficulties score is the sum of the scores of 4 factors (emotional symptoms, conduct problems, hyperactivity and peer problems), reflecting the overall situation of the child's behavioral problems. The total strengths score is the prosocial factor, reflecting the overall situation of the child's behavioral strengths. The impact factor is derived by adding the scores in the last table of the questionnaire (impact supplement).

Factors and corresponding items:

Emotional Symptoms: E03, E08, E13, E16, E24

Conduct Problems: E05, E07*, E12, E18, E22

Hyperactivity/Inattention: E02, E10, E15, E21*, E25*

Peer Problems: E06, E11*, E14*, E19, E23

Prosocial Behavior: E01, E04, E09, E17, E20

Additional Impact Supplement: F01, F04, F05

Total Difficulties Score = Emotional Symptoms + Conduct Problems + Hyperactivity/Inattention + Peer Problems

Total Strengths Score = Prosocial Behavior

(Note: Items with * are reverse scored; F02, F03, F06 are not scored; If F01 is "No", the impact score is directly recorded as 0.)

Positive scoring: Not True = 0; Somewhat True = 1; Certainly True = 2

Reverse scoring: Not True = 2; Somewhat True = 1; Certainly True = 0

Additional questions: Choosing A or B scores 0, choosing C scores 1, choosing D scores 2

Cut-off criteria:

Factor	Normal	Borderline	Abnormal
Emotional Symptoms (E03,E08,E13,E16,E24)	0～5	6	7～10
Conduct Problems (E05,E07*,E12,E18,E22)	0～3	4	5～10
Hyperactivity/Inattention (E02,E10,E15,E21*,E25*)	0～5	6	7～10
Peer Problems (E06,E11*,E14*,E19,E23)	0～3	4～5	6～10
Prosocial Behavior (E01,E04,E09,E17,E20)	10～6	5	4～0
Impact Supplement (F01,F04,F05)	0	1	2 or above
Total	0～15	16～19	20～40

Chineses Version:

一、调查问卷

表 1-1　一般资料（学生版）

A、 基本信息

序号	调查内容	选项
**A01**	民族	**1.**汉族　**2.**回族　**3.**苗族<br>**4.**土家族<br>**5.**其他（注明__族）

B、家庭及居住情况

序号	调查内容	选项
**B01**	是否住校	**1.** 是　　**2.** 否
**B02**	近半年，和你一起生活的家庭成员人数	____人
**B03**	他们分别包括（可多选）	**1.** 爷爷奶奶或外公外婆<br>**2.** 父亲<br>**3.** 母亲<br>**4.** 继父（后爸）<br>**5.** 继母（后妈）<br>**6.** 兄弟姐妹<br>**7.** 其他
**B04**	是否独生子女	**1.** 是（跳转至 **B06**）　　**2.** 否
**B05**	如非独生子女，是否感受到父母有所偏爱？	**4.** 偏爱兄弟姐妹<br>**5.** 无偏爱

		6. 偏爱自己
B06	父母争吵的频率	1. 从未 2. 有时 3.经常

C、 学业情况

序号	调查内容	选项
C01	最近一次大考（期中或期末）中，你在班级的排名大约为	7. 前 10%<br>8. 11-25%<br>9. 26-50%<br>10. 51-75%<br>11. 后 25%<br>12. 学校不公布排名
C02	本学期相比于上个学期，你的学习成绩变动情况	1. 进步 2. 退步 3. 稳定
C03	本学期相比于上个学期，你的情绪变动情况	1. 变好 2.变差 3. 不变
C04	是否担任过班级或学校的学生干部	1. 否 2. 是

表 1-2　情绪情况问卷（抑郁-焦虑-压力量表）

【指导语】：请仔细阅读以下每个条目，并根据过去一周的情况，在每个条目中选择适用于你情况的程度选项。请回答每个条目，选择没有对错之分，现在开始吧！

D、 情绪情况

序号	过去一周	不符合	有时符合	常常符合	总是符合
**D01**	我觉得很难让自己安静下来。	**0**	**1**	**2**	**3**
**D02**	我感到口干舌燥。	**0**	**1**	**2**	**3**
**D03**	我好像一点都没有感觉到任何愉快、舒畅。	**0**	**1**	**2**	**3**
**D04**	我感到呼吸困难（例如：气喘或透不过气来）。	**0**	**1**	**2**	**3**
**D05**	我感到很难主动去开始学习。	**0**	**1**	**2**	**3**
**D06**	我对事情往往做出过度反应。	**0**	**1**	**2**	**3**
**D07**	我感到颤抖（例如：手抖）。	**0**	**1**	**2**	**3**
**D08**	我觉得自己消耗了很多精力。	**0**	**1**	**2**	**3**
**D09**	我担心一些可能让自己恐慌或出丑的场合。	**0**	**1**	**2**	**3**
**D10**	我觉得自己对不久的将来没有什么可期盼的。	**0**	**1**	**2**	**3**
**D11**	我感到忐忑不安。	**0**	**1**	**2**	**3**
**D12**	我感到很难放松自己。	**0**	**1**	**2**	**3**
**D13**	我感到忧郁沮丧。	**0**	**1**	**2**	**3**
**D14**	我无法容忍任何阻碍我继续学习的事情。	**0**	**1**	**2**	**3**

**D15**	我感到快要崩溃了。	**0**	**1**	**2**	**3**
**D16**	我对任何事情都不能产生热情。	**0**	**1**	**2**	**3**
**D17**	我觉得我作为一个人没有很大的价值。	**0**	**1**	**2**	**3**
**D18**	我发觉自己很容易被激怒。	**0**	**1**	**2**	**3**
**D19**	即使在没有明显的体力活动时，我也感到心律不正常。	**0**	**1**	**2**	**3**
**D20**	我无缘无故地感到害怕。	**0**	**1**	**2**	**3**
**D21**	我感到生命毫无意义。	**0**	**1**	**2**	**3**

表 1-3　睡眠情况问卷（学生版）

【指导语】：以下问题是用于评估你的睡眠情况，对下面每一个问题，选择最符合自己的答案，现在开始吧！

序号	调查内容	没有	轻微	普通	严重	非常严重
**H01**	请评估你过去两周睡眠问题的严重程度：<br>1）入睡困难	**0**	**1**	**2**	**3**	**4**
**H02**	2）难以维持睡眠	**0**	**1**	**2**	**3**	**4**
**H03**	3）太早就醒了	**0**	**1**	**2**	**3**	**4**
序号	调查内容	（0 表示非常满意，4 表示非常不满意）				
**H04**	你对过去两周的睡眠状况满意度如何?	**0**	**1**	**2**	**3**	**4**
序号	调查内容	完全不妨碍	少许妨碍	颇为妨碍	非常妨碍	极妨碍
**H05**	在过去两周中，你认为你的睡眠问题妨碍你日常学习和生活 (例如：精力、集中力、记忆、情绪等) 到哪一个程度?	**0**	**1**	**2**	**3**	**4**
序号	调查内容	完全不明显	少许明显	颇为明显	非常明显	极明显

**H06**	在过去两周中，你的睡眠问题在降低生活质量方面，在其他人眼中有多明显?	**0**	**1**	**2**	**3**	**4**
序号	调查内容	完全不苦恼	少许苦恼	颇为苦恼	非常苦恼	极苦恼
**H07**	在过去两周中，你对你现有的睡眠问题有多忧虑/苦恼?	**0**	**1**	**2**	**3**	**4**

表 1-4　长处和困难问卷（学生版）

【指导语】：请根据你过去六个月内的经验与事实，回答以下问题，从题目的三个选项："不符合""有点符合""完全符合"中选出你觉得合适的答案，请不要遗漏任何一题，现在开始吧！

序号	过去 6 个月内	不符合	有点符合	完全符合
**E01**	我尝试对别人友善，我关心别人的感受	**0**	**1**	**2**
**E02**	我不能安定，不能长时间保持安静	**0**	**1**	**2**
**E03**	我经常头痛、肚子痛或身体不舒服	**0**	**1**	**2**
**E04**	我常与他人分享东西（例如：食物、玩具、笔）	**0**	**1**	**2**
**E05**	我觉得非常愤怒及常发脾气	**0**	**1**	**2**
**E06**	我经常独处，我通常自己玩耍	**0**	**1**	**2**
**E07**	我通常按照别人的吩咐做事	**2**	**1**	**0**
**E08**	我经常担忧，心事重重	**0**	**1**	**2**

**E09**	如果有人受伤、难过或不适，我都乐意帮忙	**0**	**1**	**2**
**E10**	我经常坐立不安或感到不耐烦	**0**	**1**	**2**
**E11**	我有一个或几个好朋友	**2**	**1**	**0**
**E12**	我经常与别人争执，我能够使别人依我的想法行事	**0**	**1**	**2**
**E13**	我经常不快乐、心情沉重或流泪	**0**	**1**	**2**
**E14**	一般来说，其他与我年纪相近的人都喜欢我	**2**	**1**	**0**
**E15**	我容易分心，我觉得难以集中精神	**0**	**1**	**2**
**E16**	我在新的环境中会感到紧张，我很容易失去自信	**0**	**1**	**2**
**E17**	我会友善的对待比我小的孩子	**0**	**1**	**2**
**E18**	我常被指责撒谎或不老实	**0**	**1**	**2**
**E19**	其他小孩或青少年常作弄或欺负我	**0**	**1**	**2**
**E20**	我常自愿帮助别人（例如：家人、老师、同学）	**0**	**1**	**2**
**E21**	我做事前会先想清楚	**2**	**1**	**0**
**E22**	我会从家里、学校或别处拿取不属于我的东西	**0**	**1**	**2**
**E23**	我与大人相处比与同学/朋友相处融洽	**0**	**1**	**2**
**E24**	我心中有很多恐惧，我容易受到惊吓	**0**	**1**	**2**
**E25**	我总能把手头上的事情办妥，我的注意力良好	**2**	**1**	**0**

【指导语】：接下来，你是否有其他意见或想法？

附加题

序号	调查内容	选项
**F01**	总的来说，你认为自己在情绪方面、注意力方面、行为方面、与别人相处方面	**5.** 否（跳转至 **G01**）<br>**6.** 是（有少许困难）

	是否有困难？	7. 是（有困难）<br>8. 是（有很大困难）
**F02**	上述这些困难出现了多久？	5. 少于 1 个月<br>6. 1-5 个月<br>7. 6-11 个月<br>8. 1 年及以上
**F03**	这些困难是否给你带来困扰？	5. 没有<br>6. 轻微<br>7. 经常<br>8. 总是
**F04**	这些困难是否对你与同龄人的关系造成干扰？	5. 没有<br>6. 轻微<br>7. 经常<br>8. 总是
**F05**	这些困难是否对你上课学习造成干扰？	5. 没有<br>6. 轻微<br>7. 经常<br>8. 总是
**F06**	这些困难是否成为你身边其他人（家人、朋友、老师）的负担？	5. 没有<br>6. 轻微<br>7. 经常<br>8. 总是

二、调查内容

1、表 1-1　一般资料（学生版）

A、基本情况：条目 A01

A01 民族：应与居民身份证/户口簿一致。

B、家庭及居住情况：条目 B01～B06

B01 住校：指本学期，每周至少有 3 天时间在学校住宿。

B02～B03 家庭成员：反映家庭结构，分为大家庭、核心家庭、单亲家庭、重组家庭。

父亲和母亲，指生物学父母和养父母。

核心家庭：只选择父亲和母亲。

大家庭：选择爷爷奶奶或外公外婆、父亲和母亲。

单亲家庭：只选择父亲或母亲。

重组家庭：选择了父亲和继母；或者母亲和继父。

隔代家庭：选择了爷爷奶奶或外公外婆，而无父亲/母亲/继父/继母。

B04 学生是否为独生子女。

B05 反映非独生子女在家庭中是否感受到父母的偏爱。

B06 反映父母吵架频率（从未、有时、经常）。

C、学业情况：条目 C01～C04

C01 反映学生最近一次大考（期中或期末）中在班级的学习成绩排名情况（前 10%、11-25%、26-50%、51-75%、后 25%、学校不公布排名）。

C02 反映学生本学期相比于上个学期，学习成绩变动情况（进步、退步、稳定）。

C03 反映学生本学期相比于上个学期，情绪变动情况（变好、变差、不变）。

C04 反映学生担任过班级或学校的学生干部的情况。

2、表 1-2　情绪情况问卷：条目 D01-D21

抑郁-焦虑-压力量表（DASS-21）是由 Lovibond 等人于 1995 年编制，该量

表是一套用于评定抑郁、焦虑和压力状况的自评量表，最初用于正常成年人，随后由 Antony 等对其进行修订，编制了 DASS 的精简版 DASS-21，研究者们开始试图将其研究对象的范围扩展到儿童、青少年甚至老年人，并在临床中使用。

量表包含 21 个条目，抑郁、焦虑和压力三个分量表各含 7 个条目，均采用从“0”（不符）到“3”（总是符合）的 4 级计分，将各分量表得分乘以 2，即为该分量表的分值，分值越高代表越具有这种情绪。

因子及对应题目：

压力量表条目：D01、D06、D08、D11、D12、D14、D18

焦虑量表条目：D02、D04、D07、D09、D15、D19、D20

抑郁量表条目：D03、D05、D10、D13、D16、D17、D21

划界标准：

抑郁量表≤9 分为正常，l0～l3 分为轻度，14～20 分为中度，21～27 分为重度，≥28 分为非常严重；

焦虑量表≤7 分为正常，8～9 分为轻度，10～14 分为中度，15～l9 分为重度，≥20 分为非常严重；

压力量表≤14 分为正常，15～l8 分为轻度，l9～25 分为中度，26～33 分为重度，≥34 分为非常严重。

3、表 1-3　长处和困难问卷：条目 E01-F06

长处和困难问卷(Strengths and Difficulties Questionnaire, SDQ)作为评估儿童青少年情绪和行为问题的测量工具，由英国心理学家 Robert Goodman 根据 ICD-10 和 DSM-IV 在 Rutter 儿童行为问卷的基础上编制的。因 SDQ 的部分题目与 ICD-10 和 DSM-IV 中情绪障碍、品行障碍、注意缺陷和多动症的诊断标准一致，所以 SDQ 在筛查儿童精神障碍、评估干预的效果方面具有诊断特征。另外，SDQ 设置的长处部分关注儿童青少年的积极表现，改变了既往的心理健康评估工具只关注负面表现，评估更加全面，它的二维结构也便于与情绪行为障碍患者进行横向比较，也可通过前后测来评估干预的效果。SDQ 包括学生版、父母版和教师版，三个版本测验的条目内容基本一致，只在措辞上存在细微差别，且三个版本都具有良好的信效度。

量表包含 25 个条目和 6 个附加题目，按照题目序号将得分相加，算出各因

子得分；困难总分为 4 因子得分之和（情绪症状、品行问题、多动和同伴交往问题），反映儿童行为问题的总体情况，长处总分即亲社会因子，反映儿童行为长处的总体情况；影响因子由问卷最后一个表格（影响因子）内的得分相加得出。

因子及对应题目：

情绪症状：E03、E08、E13、E16、E24

品行问题：E05、E07*、E12、E18、E22

多动注意不能：E02、E10、E15、E21*、E25*

同伴交往问题：E06、E11*、E14*、E19、E23

亲社会行为：E01、E04、E09、E17、E20

附加影响因子得分：F01、F04、F05

困难总分=情绪症状+品行问题+多动注意不能+同伴交往问题

长处总分=亲社会行为

（注：加*号的为反向计分条目；F02，F03，F06不计分；若F01为“否”，影响因子得分直接记0分。）

正向计分方式：不符合=0分；有点符合=1分；完全符合=2分

反向计分方式：不符合=2分；有点符合=1分；完全符合=0分

附加题目：选A、B计0分，选C计1分，选D计2分

划界标准：

因子	正常	边缘水平	异常
情绪症状(E03、E08、E13、E16、E24)	0～5	6	7～10
品行问题(E05、E07*、E12、E18、E22)	0～3	4	5～10
多动(E02、E10、E15、E21*、E25*)	0～5	6	7～10
同伴交往问题(E06、E11*、E14*、E19、E23)	0～3	4～5	6～10
亲社会行为(E01、E04、E09、E17、E20)	10～6	5	4～0
附加影响因子(F01、F04、F05)	0	1	2 或以上
困难总分	0～15	16～19	20～40

Reading Tasks

English Version:

Fixed Reading Material

- *The North Wind and the Sun*

The wind and the sun were disputing which was the stronger. Suddenly they saw a traveler coming down the road, and the sun said: "I see a way to decide our dispute. Whichever of us can cause that traveler to take off his cloak shall be regarded as the stronger. You begin." So the sun retired behind a cloud, and the wind began to blow as hard as it could upon the traveler. But the harder it blew, the more closely did the traveler wrap his cloak round him, till at last the wind had to give up in despair. Then the sun came out and shone in all its glory upon the traveler, who soon found it too hot to walk with his cloak on.

Open-ended Question Texts

I. Baseline Questions:
Please describe how your day was yesterday?
How are you feeling right now?
What is your happiest memory from the past week?
What is your saddest memory from the past week?
II. Self-Evaluation:
What is the most creative thing you have done?
III. Coping with Adversity:
When you are sure you are right but others disagree with you, what would you do?
IV. Achievement Motivation:
How would you strive to achieve your goals?
I have structured it with clear section headings and numbered lists for clarity. All the content matches what you originally provided, but presented in English per your request for an English psychology journal submission.

中文版本：

固定材料阅读

- *《北风和太阳》*

有一回,北风跟太阳在那儿争论谁的本领大。说着说着,来了一个过路的,身上穿了一件厚袍子。他们俩就商量好了,说谁能先叫这个过路的把他的袍子脱下来,就算是他的本领大。北风就使劲吹起来,拼命地吹。可是,他吹得越厉害,那个人就把他的袍子裹得越紧。到末了儿,北风没辙了,只好就算了。一会儿,太阳出来一晒,那个人马上就把袍子脱了下来。所以,北风不得不承认,还是太阳比他的本领大。

开放性问题文本

一、*基线问题:*
请描述一下,你昨天过的怎么样?
请描述一下,你的感受如何呢?
请描述一下,现在回想最近一周最开心的记忆?
请描述一下,现在回想最近一周最悲伤的记忆?
二、*自我评价:*
在你所做过的事情中,最有创造性的是什么?
三、*挫折应对:*
当你确信自己是正确的,但是其他人却不赞同你时,你会怎样做?
四、*成就动机:*
为了实现自己的目标你会怎样努力?
