# Supplementary material for: Using Speech Features and Machine Learning Models to Predict Emotional and Behavioral Problems in Chinese Adolescents
Source: Depress Anxiety. 2025 Jun 16;2025:5734107. doi: 10.1155/da/5734107 (PMC12185205; doi:10.1155/da/5734107)
Supplement: Supporting Information 1 — The supporting information file includes all additional figures and tables referenced in the main text as Appendix A–G. [file 5734107.f1.zip › Appendix B.pdf]

Dimension	Gender	Feature	Problematic M(SD)	Non-Problematic M(SD)	p-value	Cohen's d
**Hyperactivity**	Male	F0 Minimum	59.18 (11.40)	58.90 (12.76)	0.5938	0.02
**Hyperactivity**	Male	F0 Maximum	483.15 (2.88)	483.08 (5.09)	0.7350	0.02
**Hyperactivity**	Male	F0 Range	423.97 (12.53)	424.18 (15.39)	0.7349	-0.01
**Hyperactivity**	Male	F0 Mean	280.98 (15.87)	281.76 (16.69)	0.2511	-0.05
**Hyperactivity**	Male	F0 Std	113.21 (7.01)	112.94 (7.60)	0.3885	0.04
**Hyperactivity**	Male	F0 Skewness	0.13 (0.17)	0.13 (0.17)	0.5189	0.03
**Hyperactivity**	Male	F0 Kurtosis	-1.15 (0.23)	-1.13 (0.26)	0.0407	-0.09
**Hyperactivity**	Male	F0 de Minimum	-177.33 (16.79)	-177.57 (18.80)	0.7516	0.01
**Hyperactivity**	Male	F0 de Maximum	187.15 (12.93)	187.06 (14.77)	0.8777	0.01
**Hyperactivity**	Male	F0 de Range	364.48 (25.23)	364.63 (28.99)	0.8985	-0.01
**Hyperactivity**	Male	F0 de Mean	0.17 (0.67)	0.18 (0.66)	0.6450	-0.02
**Hyperactivity**	Male	F0 de Std	76.85 (12.67)	76.29 (13.39)	0.3076	0.04
**Hyperactivity**	Male	F0 de Skewness	0.32 (0.17)	0.32 (0.18)	0.8126	0.01
**Hyperactivity**	Male	F0 de Kurtosis	-0.49 (0.93)	-0.43 (1.03)	0.1258	-0.06
**Hyperactivity**	Male	F0 de2 Minimum	-651.51 (60.40)	-648.94 (61.34)	0.3059	-0.04
**Hyperactivity**	Male	F0 de2 Maximum	614.91 (78.60)	610.85 (78.22)	0.2063	0.05
**Hyperactivity**	Male	F0 de2 Range	1266.41 (127.32)	1259.80 (129.23)	0.2101	0.05
**Hyperactivity**	Male	F0 de2 Mean	0.05 (1.61)	0.10 (1.48)	0.3717	-0.04
**Hyperactivity**	Male	F0 de2 Std	298.99 (24.01)	298.61 (25.15)	0.7118	0.02
**Hyperactivity**	Male	F0 de2 Skewness	-0.06 (0.05)	-0.06 (0.05)	0.9604	0.00
**Hyperactivity**	Male	F0 de2 Kurtosis	-1.09 (0.26)	-1.08 (0.27)	0.3844	-0.04
**Hyperactivity**	Male	Energy Minimum	-11.66 (6.69)	-11.48 (6.19)	0.4811	-0.03
**Hyperactivity**	Male	Energy Maximum	3.73 (1.26)	3.89 (1.26)	0.0027	-0.12
**Hyperactivity**	Male	Energy Range	15.39 (7.13)	15.37 (6.60)	0.9238	0.00
**Hyperactivity**	Male	Energy Mean	-2.05 (1.05)	-2.06 (1.13)	0.8503	0.01
**Hyperactivity**	Male	Energy Std	2.62 (0.63)	2.65 (0.59)	0.3062	-0.04
**Hyperactivity**	Male	Energy Skewness	-0.79 (0.63)	-0.73 (0.68)	0.0182	-0.10
**Hyperactivity**	Male	Energy Kurtosis	1.66 (5.09)	1.56 (5.24)	0.6376	0.02
**Hyperactivity**	Male	Energy de Minimum	-3.55 (1.95)	-3.45 (1.76)	0.1449	-0.06
**Hyperactivity**	Male	Energy de Maximum	4.56 (2.32)	4.51 (2.15)	0.6060	0.02
**Hyperactivity**	Male	Energy de Range	8.11 (3.98)	7.96 (3.61)	0.3093	0.04
**Hyperactivity**	Male	Energy de Mean	0.01 (0.03)	0.01 (0.03)	0.3828	-0.04
**Hyperactivity**	Male	Energy de Std	1.11 (0.23)	1.11 (0.22)	0.7665	0.01
**Hyperactivity**	Male	Energy de Skewness	0.37 (0.47)	0.42 (0.53)	0.0460	-0.08
**Hyperactivity**	Male	Energy de Kurtosis	1.74 (3.76)	1.79 (3.67)	0.7263	-0.01
**Hyperactivity**	Male	Energy de2 Minimum	-7.18 (4.27)	-6.99 (3.89)	0.2296	-0.05
**Hyperactivity**	Male	Energy de2 Maximum	8.59 (6.69)	8.34 (6.17)	0.3247	0.04
**Hyperactivity**	Male	Energy de2 Range	15.77 (10.66)	15.33 (9.76)	0.2711	0.04
**Hyperactivity**	Male	Energy de2 Mean	-0.01 (0.02)	-0.01 (0.02)	0.6068	0.02

**Hyperactivity**	Male	Energy de2 Std	1.56 (0.30)	1.55 (0.32)	0.5496	0.02
**Hyperactivity**	Male	Energy de2 Skewness	0.71 (0.72)	0.71 (0.70)	0.9429	0.00
**Hyperactivity**	Male	Energy de2 Kurtosis	6.83 (22.15)	6.16 (19.40)	0.4120	0.03
**Hyperactivity**	Male	MFCC 1 Minimum	-11.66 (6.69)	-11.48 (6.19)	0.4811	-0.03
**Hyperactivity**	Male	MFCC 1 Maximum	3.73 (1.26)	3.89 (1.26)	0.0027	-0.12
**Hyperactivity**	Male	MFCC 1 Range	15.39 (7.13)	15.37 (6.60)	0.9238	0.00
**Hyperactivity**	Male	MFCC 1 Mean	-2.05 (1.05)	-2.06 (1.13)	0.8503	0.01
**Hyperactivity**	Male	MFCC 1 Std	2.62 (0.63)	2.65 (0.59)	0.3062	-0.04
**Hyperactivity**	Male	MFCC 1 Skewness	-0.79 (0.63)	-0.73 (0.68)	0.0182	-0.10
**Hyperactivity**	Male	MFCC 1 Kurtosis	1.66 (5.09)	1.56 (5.24)	0.6376	0.02
**Hyperactivity**	Male	MFCC 2 Minimum	-479.68 (101.45)	-475.01 (104.29)	0.2727	-0.05
**Hyperactivity**	Male	MFCC 2 Maximum	356.76 (59.02)	351.21 (62.10)	0.0279	0.09
**Hyperactivity**	Male	MFCC 2 Range	836.44 (148.04)	826.23 (154.53)	0.1042	0.07
**Hyperactivity**	Male	MFCC 2 Mean	42.49 (22.60)	40.87 (23.87)	0.0954	0.07
**Hyperactivity**	Male	MFCC 2 Std	157.89 (25.47)	155.42 (27.40)	0.0256	0.09
**Hyperactivity**	Male	MFCC 2 Skewness	-0.69 (0.26)	-0.69 (0.29)	0.6872	-0.02
**Hyperactivity**	Male	MFCC 2 Kurtosis	0.27 (0.48)	0.31 (0.49)	0.0365	-0.09
**Hyperactivity**	Male	MFCC 3 Minimum	-395.30 (111.34)	-388.99 (113.44)	0.1731	-0.06
**Hyperactivity**	Male	MFCC 3 Maximum	410.28 (74.87)	408.84 (76.44)	0.6446	0.02
**Hyperactivity**	Male	MFCC 3 Range	805.58 (171.86)	797.83 (173.67)	0.2751	0.04
**Hyperactivity**	Male	MFCC 3 Mean	28.03 (26.76)	30.26 (29.52)	0.0605	-0.08
**Hyperactivity**	Male	MFCC 3 Std	148.64 (27.09)	146.93 (27.89)	0.1326	0.06
**Hyperactivity**	Male	MFCC 3 Skewness	-0.20 (0.24)	-0.20 (0.24)	0.5690	0.02
**Hyperactivity**	Male	MFCC 3 Kurtosis	-0.32 (0.33)	-0.30 (0.36)	0.1380	-0.06
**Hyperactivity**	Male	MFCC 4 Minimum	-465.15 (112.89)	-455.14 (115.64)	0.0338	-0.09
**Hyperactivity**	Male	MFCC 4 Maximum	549.89 (111.73)	543.29 (113.03)	0.1530	0.06
**Hyperactivity**	Male	MFCC 4 Range	1015.05 (203.33)	998.43 (207.50)	0.0498	0.08
**Hyperactivity**	Male	MFCC 4 Mean	19.50 (31.08)	21.93 (31.91)	0.0617	-0.08
**Hyperactivity**	Male	MFCC 4 Std	188.60 (30.78)	186.01 (32.66)	0.0506	0.08
**Hyperactivity**	Male	MFCC 4 Skewness	0.15 (0.25)	0.16 (0.26)	0.5257	-0.03
**Hyperactivity**	Male	MFCC 4 Kurtosis	-0.29 (0.34)	-0.27 (0.37)	0.2711	-0.05
**Hyperactivity**	Male	MFCC 5 Minimum	-458.38 (129.67)	-450.35 (128.39)	0.1270	-0.06
**Hyperactivity**	Male	MFCC 5 Maximum	400.96 (90.88)	396.30 (91.96)	0.2155	0.05
**Hyperactivity**	Male	MFCC 5 Range	859.34 (198.50)	846.65 (196.72)	0.1157	0.06
**Hyperactivity**	Male	MFCC 5 Mean	-6.57 (30.37)	-6.91 (30.42)	0.7799	0.01
**Hyperactivity**	Male	MFCC 5 Std	144.08 (26.93)	141.10 (26.79)	0.0066	0.11
**Hyperactivity**	Male	MFCC 5 Skewness	-0.14 (0.24)	-0.15 (0.25)	0.4286	0.03
**Hyperactivity**	Male	MFCC 5 Kurtosis	-0.04 (0.38)	0.01 (0.43)	0.0065	-0.12
**Hyperactivity**	Male	MFCC 6 Minimum	-451.88 (109.36)	-446.84 (114.21)	0.2775	-0.05
**Hyperactivity**	Male	MFCC 6 Maximum	406.90 (95.65)	402.66 (96.90)	0.2847	0.04

**Hyperactivity**	Male	MFCC 6 Range	858.78 (175.50)	849.50 (182.69)	0.2119	0.05
**Hyperactivity**	Male	MFCC 6 Mean	-13.89 (31.81)	-12.74 (32.04)	0.3832	-0.04
**Hyperactivity**	Male	MFCC 6 Std	141.98 (21.53)	140.73 (22.40)	0.1721	0.06
**Hyperactivity**	Male	MFCC 6 Skewness	-0.15 (0.25)	-0.14 (0.25)	0.6144	-0.02
**Hyperactivity**	Male	MFCC 6 Kurtosis	0.03 (0.39)	0.02 (0.40)	0.4865	0.03
**Hyperactivity**	Male	MFCC 7 Minimum	-521.31 (123.48)	-519.82 (126.14)	0.7722	-0.01
**Hyperactivity**	Male	MFCC 7 Maximum	374.81 (90.31)	365.77 (93.35)	0.0175	0.10
**Hyperactivity**	Male	MFCC 7 Range	896.12 (183.72)	885.59 (190.64)	0.1749	0.06
**Hyperactivity**	Male	MFCC 7 Mean	-50.88 (35.37)	-53.63 (34.73)	0.0539	0.08
**Hyperactivity**	Male	MFCC 7 Std	149.65 (23.57)	148.54 (24.44)	0.2634	0.05
**Hyperactivity**	Male	MFCC 7 Skewness	-0.19 (0.22)	-0.20 (0.23)	0.2261	0.05
**Hyperactivity**	Male	MFCC 7 Kurtosis	-0.03 (0.37)	-0.02 (0.39)	0.6863	-0.02
**Hyperactivity**	Male	MFCC 8 Minimum	-516.70 (124.85)	-511.79 (128.97)	0.3504	-0.04
**Hyperactivity**	Male	MFCC 8 Maximum	357.72 (89.50)	354.79 (90.08)	0.4251	0.03
**Hyperactivity**	Male	MFCC 8 Range	874.42 (183.54)	866.58 (186.56)	0.3032	0.04
**Hyperactivity**	Male	MFCC 8 Mean	-61.34 (32.67)	-60.66 (35.00)	0.6339	-0.02
**Hyperactivity**	Male	MFCC 8 Std	143.76 (21.21)	142.81 (22.27)	0.2920	0.04
**Hyperactivity**	Male	MFCC 8 Skewness	-0.16 (0.26)	-0.16 (0.26)	0.9350	0.00
**Hyperactivity**	Male	MFCC 8 Kurtosis	0.05 (0.42)	0.05 (0.40)	0.7653	0.01
**Hyperactivity**	Male	MFCC 9 Minimum	-434.15 (102.33)	-429.72 (98.17)	0.2740	-0.04
**Hyperactivity**	Male	MFCC 9 Maximum	371.63 (86.33)	367.60 (92.20)	0.2814	0.05
**Hyperactivity**	Male	MFCC 9 Range	805.78 (158.86)	797.33 (160.92)	0.1986	0.05
**Hyperactivity**	Male	MFCC 9 Mean	-30.94 (27.68)	-30.77 (29.18)	0.8874	-0.01
**Hyperactivity**	Male	MFCC 9 Std	133.21 (18.27)	132.87 (19.17)	0.6585	0.02
**Hyperactivity**	Male	MFCC 9 Skewness	-0.01 (0.24)	-0.02 (0.24)	0.3112	0.04
**Hyperactivity**	Male	MFCC 9 Kurtosis	-0.02 (0.37)	-0.03 (0.39)	0.3362	0.04
**Hyperactivity**	Male	MFCC 10 Minimum	-388.92 (90.70)	-388.15 (92.80)	0.8383	-0.01
**Hyperactivity**	Male	MFCC 10 Maximum	353.50 (94.70)	345.22 (92.90)	0.0302	0.09
**Hyperactivity**	Male	MFCC 10 Range	742.42 (161.02)	733.37 (160.82)	0.1696	0.06
**Hyperactivity**	Male	MFCC 10 Mean	-22.13 (24.53)	-24.01 (25.59)	0.0706	0.08
**Hyperactivity**	Male	MFCC 10 Std	120.96 (20.49)	120.13 (21.02)	0.3342	0.04
**Hyperactivity**	Male	MFCC 10 Skewness	0.02 (0.23)	-0.00 (0.24)	0.0461	0.08
**Hyperactivity**	Male	MFCC 10 Kurtosis	0.03 (0.38)	0.03 (0.40)	0.7534	-0.01
**Hyperactivity**	Male	MFCC 11 Minimum	-349.27 (84.01)	-349.04 (83.24)	0.9469	0.00
**Hyperactivity**	Male	MFCC 11 Maximum	298.11 (83.22)	290.38 (81.99)	0.0217	0.09
**Hyperactivity**	Male	MFCC 11 Range	647.37 (141.33)	639.41 (139.07)	0.1634	0.06
**Hyperactivity**	Male	MFCC 11 Mean	-22.96 (26.24)	-24.33 (25.49)	0.1909	0.05
**Hyperactivity**	Male	MFCC 11 Std	102.31 (15.82)	101.45 (15.90)	0.1865	0.05
**Hyperactivity**	Male	MFCC 11 Skewness	-0.03 (0.25)	-0.03 (0.25)	0.9126	0.00
**Hyperactivity**	Male	MFCC 11 Kurtosis	0.15 (0.44)	0.14 (0.41)	0.6684	0.02

**Hyperactivity**	Male	MFCC 12 Minimum	-307.16 (68.66)	-305.55 (76.20)	0.6009	-0.02
**Hyperactivity**	Male	MFCC 12 Maximum	269.56 (67.10)	265.95 (68.67)	0.1976	0.05
**Hyperactivity**	Male	MFCC 12 Range	576.72 (112.39)	571.50 (121.98)	0.2903	0.04
**Hyperactivity**	Male	MFCC 12 Mean	-15.87 (21.44)	-15.93 (21.35)	0.9528	0.00
**Hyperactivity**	Male	MFCC 12 Std	89.29 (11.70)	88.55 (13.22)	0.1607	0.06
**Hyperactivity**	Male	MFCC 12 Skewness	-0.05 (0.26)	-0.05 (0.25)	0.9937	0.00
**Hyperactivity**	Male	MFCC 12 Kurtosis	0.24 (0.42)	0.24 (0.44)	0.7562	0.01
**Hyperactivity**	Male	MFCC 13 Minimum	-258.92 (66.84)	-249.42 (65.88)	0.0004	-0.14
**Hyperactivity**	Male	MFCC 13 Maximum	256.68 (61.61)	254.78 (64.75)	0.4679	0.03
**Hyperactivity**	Male	MFCC 13 Range	515.60 (105.58)	504.19 (108.58)	0.0100	0.11
**Hyperactivity**	Male	MFCC 13 Mean	-0.75 (18.89)	0.63 (18.49)	0.0699	-0.07
**Hyperactivity**	Male	MFCC 13 Std	77.80 (11.37)	77.05 (11.46)	0.1092	0.07
**Hyperactivity**	Male	MFCC 13 Skewness	0.02 (0.27)	0.04 (0.26)	0.1810	-0.05
**Hyperactivity**	Male	MFCC 13 Kurtosis	0.36 (0.44)	0.31 (0.45)	0.0167	0.10
**Hyperactivity**	Male	MELS 1 Minimum	-3.55 (1.95)	-3.45 (1.76)	0.1449	-0.06
**Hyperactivity**	Male	MELS 1 Maximum	4.56 (2.32)	4.51 (2.15)	0.6060	0.02
**Hyperactivity**	Male	MELS 1 Range	8.11 (3.98)	7.96 (3.61)	0.3093	0.04
**Hyperactivity**	Male	MELS 1 Mean	0.01 (0.03)	0.01 (0.03)	0.3828	-0.04
**Hyperactivity**	Male	MELS 1 Std	1.11 (0.23)	1.11 (0.22)	0.7665	0.01
**Hyperactivity**	Male	MELS 1 Skewness	0.37 (0.47)	0.42 (0.53)	0.0460	-0.08
**Hyperactivity**	Male	MELS 1 Kurtosis	1.74 (3.76)	1.79 (3.67)	0.7263	-0.01
**Hyperactivity**	Male	MELS 2 Minimum	-210.54 (45.10)	-207.84 (48.00)	0.1660	-0.06
**Hyperactivity**	Male	MELS 2 Maximum	194.12 (42.33)	193.44 (43.93)	0.7052	0.02
**Hyperactivity**	Male	MELS 2 Range	404.65 (82.99)	401.28 (87.45)	0.3422	0.04
**Hyperactivity**	Male	MELS 2 Mean	0.11 (0.79)	0.14 (0.87)	0.2999	-0.04
**Hyperactivity**	Male	MELS 2 Std	58.43 (11.22)	57.79 (11.81)	0.1825	0.06
**Hyperactivity**	Male	MELS 2 Skewness	-0.12 (0.24)	-0.09 (0.25)	0.0039	-0.12
**Hyperactivity**	Male	MELS 2 Kurtosis	1.10 (0.60)	1.14 (0.66)	0.1484	-0.06
**Hyperactivity**	Male	MELS 3 Minimum	-175.40 (42.40)	-173.00 (42.34)	0.1673	-0.06
**Hyperactivity**	Male	MELS 3 Maximum	167.16 (41.39)	164.30 (42.07)	0.0950	0.07
**Hyperactivity**	Male	MELS 3 Range	342.56 (78.19)	337.30 (78.85)	0.1026	0.07
**Hyperactivity**	Male	MELS 3 Mean	0.09 (0.90)	0.06 (0.77)	0.3148	0.04
**Hyperactivity**	Male	MELS 3 Std	49.96 (8.64)	49.64 (8.83)	0.3757	0.04
**Hyperactivity**	Male	MELS 3 Skewness	-0.12 (0.18)	-0.13 (0.19)	0.4208	0.03
**Hyperactivity**	Male	MELS 3 Kurtosis	0.44 (0.42)	0.45 (0.42)	0.5458	-0.02
**Hyperactivity**	Male	MELS 4 Minimum	-208.85 (45.11)	-207.63 (47.50)	0.5261	-0.03
**Hyperactivity**	Male	MELS 4 Maximum	205.13 (48.15)	202.28 (47.81)	0.1449	0.06
**Hyperactivity**	Male	MELS 4 Range	413.98 (83.38)	409.90 (86.36)	0.2459	0.05
**Hyperactivity**	Male	MELS 4 Mean	0.06 (0.89)	0.06 (1.01)	0.9484	0.00
**Hyperactivity**	Male	MELS 4 Std	60.48 (8.48)	59.92 (8.89)	0.1198	0.06

**Hyperactivity**	Male	MELS 4 Skewness	-0.08 (0.19)	-0.07 (0.19)	0.4853	-0.03
**Hyperactivity**	Male	MELS 4 Kurtosis	0.42 (0.42)	0.43 (0.49)	0.8404	-0.01
**Hyperactivity**	Male	MELS 5 Minimum	-183.77 (44.51)	-178.30 (43.71)	0.0023	-0.12
**Hyperactivity**	Male	MELS 5 Maximum	173.51 (39.68)	169.66 (39.38)	0.0170	0.10
**Hyperactivity**	Male	MELS 5 Range	357.28 (76.96)	347.96 (76.04)	0.0028	0.12
**Hyperactivity**	Male	MELS 5 Mean	0.05 (0.67)	0.06 (0.82)	0.7863	-0.01
**Hyperactivity**	Male	MELS 5 Std	51.95 (7.57)	50.99 (7.70)	0.0025	0.12
**Hyperactivity**	Male	MELS 5 Skewness	-0.07 (0.17)	-0.05 (0.17)	0.0198	-0.10
**Hyperactivity**	Male	MELS 5 Kurtosis	0.37 (0.38)	0.35 (0.41)	0.2621	0.05
**Hyperactivity**	Male	MELS 6 Minimum	-183.44 (39.76)	-180.14 (41.32)	0.0497	-0.08
**Hyperactivity**	Male	MELS 6 Maximum	181.10 (40.48)	178.59 (40.55)	0.1313	0.06
**Hyperactivity**	Male	MELS 6 Range	364.54 (71.86)	358.73 (74.43)	0.0554	0.08
**Hyperactivity**	Male	MELS 6 Mean	0.03 (0.87)	0.08 (0.78)	0.1880	-0.05
**Hyperactivity**	Male	MELS 6 Std	52.21 (6.25)	51.77 (6.80)	0.1039	0.07
**Hyperactivity**	Male	MELS 6 Skewness	-0.01 (0.19)	-0.01 (0.17)	0.8002	-0.01
**Hyperactivity**	Male	MELS 6 Kurtosis	0.44 (0.48)	0.42 (0.39)	0.2188	0.05
**Hyperactivity**	Male	MELS 7 Minimum	-198.06 (45.86)	-198.07 (46.03)	0.9962	0.00
**Hyperactivity**	Male	MELS 7 Maximum	191.04 (44.10)	188.67 (44.00)	0.1882	0.05
**Hyperactivity**	Male	MELS 7 Range	389.10 (81.67)	386.74 (81.92)	0.4810	0.03
**Hyperactivity**	Male	MELS 7 Mean	0.02 (0.70)	0.03 (0.69)	0.7516	-0.01
**Hyperactivity**	Male	MELS 7 Std	55.49 (7.06)	55.28 (7.46)	0.4948	0.03
**Hyperactivity**	Male	MELS 7 Skewness	-0.06 (0.18)	-0.07 (0.18)	0.0257	0.09
**Hyperactivity**	Male	MELS 7 Kurtosis	0.46 (0.41)	0.49 (0.42)	0.1206	-0.06
**Hyperactivity**	Male	MELS 8 Minimum	-193.86 (43.52)	-191.23 (46.00)	0.1581	-0.06
**Hyperactivity**	Male	MELS 8 Maximum	186.63 (42.71)	186.46 (44.31)	0.9252	0.00
**Hyperactivity**	Male	MELS 8 Range	380.49 (77.76)	377.68 (82.54)	0.4022	0.03
**Hyperactivity**	Male	MELS 8 Mean	-0.00 (0.58)	-0.04 (0.85)	0.2712	0.05
**Hyperactivity**	Male	MELS 8 Std	53.81 (6.54)	53.49 (7.14)	0.2798	0.05
**Hyperactivity**	Male	MELS 8 Skewness	-0.06 (0.20)	-0.04 (0.17)	0.0142	-0.10
**Hyperactivity**	Male	MELS 8 Kurtosis	0.51 (0.45)	0.50 (0.42)	0.5431	0.02
**Hyperactivity**	Male	MELS 9 Minimum	-179.33 (39.53)	-176.34 (39.74)	0.0662	-0.08
**Hyperactivity**	Male	MELS 9 Maximum	178.09 (37.02)	175.75 (39.33)	0.1419	0.06
**Hyperactivity**	Male	MELS 9 Range	357.42 (67.92)	352.09 (71.44)	0.0663	0.08
**Hyperactivity**	Male	MELS 9 Mean	0.03 (0.89)	-0.03 (0.69)	0.0701	0.07
**Hyperactivity**	Male	MELS 9 Std	50.94 (5.56)	50.55 (5.98)	0.1139	0.07
**Hyperactivity**	Male	MELS 9 Skewness	0.00 (0.17)	0.01 (0.17)	0.5908	-0.02
**Hyperactivity**	Male	MELS 9 Kurtosis	0.43 (0.37)	0.43 (0.42)	0.8984	-0.01
**Hyperactivity**	Male	MELS 10 Minimum	-165.08 (37.27)	-163.63 (38.58)	0.3537	-0.04
**Hyperactivity**	Male	MELS 10 Maximum	161.36 (35.16)	159.79 (36.19)	0.2890	0.04
**Hyperactivity**	Male	MELS 10 Range	326.44 (64.55)	323.42 (67.62)	0.2718	0.05

**Hyperactivity**	Male	MELS 10 Mean	0.02 (0.71)	0.01 (0.80)	0.6981	0.02
**Hyperactivity**	Male	MELS 10 Std	46.43 (5.20)	46.21 (5.60)	0.3319	0.04
**Hyperactivity**	Male	MELS 10 Skewness	-0.01 (0.16)	-0.02 (0.16)	0.3828	0.04
**Hyperactivity**	Male	MELS 10 Kurtosis	0.41 (0.38)	0.42 (0.39)	0.9040	0.00
**Hyperactivity**	Male	MELS 11 Minimum	-146.15 (32.88)	-144.85 (33.29)	0.3401	-0.04
**Hyperactivity**	Male	MELS 11 Maximum	143.49 (30.67)	142.21 (31.56)	0.3188	0.04
**Hyperactivity**	Male	MELS 11 Range	289.64 (56.89)	287.06 (58.37)	0.2784	0.04
**Hyperactivity**	Male	MELS 11 Mean	-0.02 (0.73)	0.00 (0.62)	0.4386	-0.03
**Hyperactivity**	Male	MELS 11 Std	41.45 (4.36)	41.26 (4.71)	0.3182	0.04
**Hyperactivity**	Male	MELS 11 Skewness	-0.01 (0.15)	-0.01 (0.16)	0.7201	-0.02
**Hyperactivity**	Male	MELS 11 Kurtosis	0.37 (0.37)	0.38 (0.39)	0.5764	-0.02
**Hyperactivity**	Male	MELS 12 Minimum	-130.13 (26.68)	-128.93 (27.97)	0.2908	-0.04
**Hyperactivity**	Male	MELS 12 Maximum	131.26 (27.62)	129.58 (28.85)	0.1521	0.06
**Hyperactivity**	Male	MELS 12 Range	261.39 (47.73)	258.51 (51.14)	0.1643	0.06
**Hyperactivity**	Male	MELS 12 Mean	-0.01 (0.59)	-0.02 (0.51)	0.5152	0.03
**Hyperactivity**	Male	MELS 12 Std	37.30 (3.66)	37.08 (4.16)	0.1937	0.06
**Hyperactivity**	Male	MELS 12 Skewness	0.01 (0.16)	-0.00 (0.17)	0.1379	0.06
**Hyperactivity**	Male	MELS 12 Kurtosis	0.40 (0.40)	0.40 (0.41)	0.6841	0.02
**Hyperactivity**	Male	MELS 13 Minimum	-115.34 (23.01)	-114.74 (24.97)	0.5542	-0.02
**Hyperactivity**	Male	MELS 13 Maximum	116.88 (23.66)	115.74 (25.78)	0.2724	0.05
**Hyperactivity**	Male	MELS 13 Range	232.22 (41.32)	230.48 (45.61)	0.3441	0.04
**Hyperactivity**	Male	MELS 13 Mean	-0.00 (0.45)	0.00 (0.50)	0.8297	-0.01
**Hyperactivity**	Male	MELS 13 Std	33.29 (3.40)	33.05 (3.64)	0.1144	0.07
**Hyperactivity**	Male	MELS 13 Skewness	0.01 (0.16)	0.01 (0.16)	0.7313	-0.01
**Hyperactivity**	Male	MELS 13 Kurtosis	0.39 (0.35)	0.39 (0.36)	0.6928	0.02
**Hyperactivity**	Male	MELS 14 Minimum	-7.18 (4.27)	-6.99 (3.89)	0.2296	-0.05
**Hyperactivity**	Male	MELS 14 Maximum	8.59 (6.69)	8.34 (6.17)	0.3247	0.04
**Hyperactivity**	Male	MELS 14 Range	15.77 (10.66)	15.33 (9.76)	0.2711	0.04
**Hyperactivity**	Male	MELS 14 Mean	-0.01 (0.02)	-0.01 (0.02)	0.6068	0.02
**Hyperactivity**	Male	MELS 14 Std	1.56 (0.30)	1.55 (0.32)	0.5496	0.02
**Hyperactivity**	Male	MELS 14 Skewness	0.71 (0.72)	0.71 (0.70)	0.9429	0.00
**Hyperactivity**	Male	MELS 14 Kurtosis	6.83 (22.15)	6.16 (19.40)	0.4120	0.03
**Hyperactivity**	Male	MELS 15 Minimum	-227.69 (60.85)	-226.95 (64.98)	0.7802	-0.01
**Hyperactivity**	Male	MELS 15 Maximum	336.86 (89.59)	333.35 (92.16)	0.3494	0.04
**Hyperactivity**	Male	MELS 15 Range	564.55 (137.35)	560.30 (144.51)	0.4690	0.03
**Hyperactivity**	Male	MELS 15 Mean	-0.01 (0.72)	0.01 (0.89)	0.6233	-0.02
**Hyperactivity**	Male	MELS 15 Std	63.46 (10.59)	63.10 (11.51)	0.4403	0.03
**Hyperactivity**	Male	MELS 15 Skewness	0.85 (0.35)	0.84 (0.35)	0.6039	0.02
**Hyperactivity**	Male	MELS 15 Kurtosis	3.32 (1.57)	3.33 (1.59)	0.9551	0.00
**Hyperactivity**	Male	MELS 16 Minimum	-252.08 (65.97)	-251.01 (68.87)	0.7019	-0.02

**Hyperactivity**	Male	MELS 16 Maximum	234.10 (63.67)	234.51 (62.66)	0.8734	-0.01
**Hyperactivity**	Male	MELS 16 Range	486.18 (117.40)	485.52 (117.74)	0.8907	0.01
**Hyperactivity**	Male	MELS 16 Mean	-0.15 (0.97)	-0.14 (1.20)	0.7936	-0.01
**Hyperactivity**	Male	MELS 16 Std	60.63 (7.53)	60.43 (8.02)	0.5409	0.03
**Hyperactivity**	Male	MELS 16 Skewness	-0.15 (0.21)	-0.14 (0.23)	0.3475	-0.04
**Hyperactivity**	Male	MELS 16 Kurtosis	1.09 (0.83)	1.15 (0.93)	0.0958	-0.07
**Hyperactivity**	Male	MELS 17 Minimum	-292.55 (68.25)	-289.72 (74.53)	0.3469	-0.04
**Hyperactivity**	Male	MELS 17 Maximum	285.16 (68.15)	284.10 (74.66)	0.7267	0.01
**Hyperactivity**	Male	MELS 17 Range	577.71 (122.80)	573.83 (135.10)	0.4760	0.03
**Hyperactivity**	Male	MELS 17 Mean	-0.07 (1.42)	-0.15 (1.31)	0.1157	0.06
**Hyperactivity**	Male	MELS 17 Std	73.13 (7.98)	72.24 (8.79)	0.0126	0.11
**Hyperactivity**	Male	MELS 17 Skewness	-0.09 (0.18)	-0.08 (0.19)	0.0947	-0.07
**Hyperactivity**	Male	MELS 17 Kurtosis	1.00 (0.64)	1.05 (0.75)	0.1235	-0.07
**Hyperactivity**	Male	MELS 18 Minimum	-270.32 (61.87)	-266.68 (64.10)	0.1624	-0.06
**Hyperactivity**	Male	MELS 18 Maximum	269.44 (64.31)	265.09 (64.43)	0.0991	0.07
**Hyperactivity**	Male	MELS 18 Range	539.76 (113.89)	531.76 (114.14)	0.0870	0.07
**Hyperactivity**	Male	MELS 18 Mean	-0.06 (1.13)	0.00 (1.36)	0.2790	-0.05
**Hyperactivity**	Male	MELS 18 Std	71.56 (6.65)	70.79 (7.14)	0.0083	0.11
**Hyperactivity**	Male	MELS 18 Skewness	-0.04 (0.16)	-0.03 (0.16)	0.2886	-0.04
**Hyperactivity**	Male	MELS 18 Kurtosis	0.69 (0.67)	0.67 (0.59)	0.4815	0.03
**Hyperactivity**	Male	MELS 19 Minimum	-284.19 (71.46)	-278.62 (68.23)	0.0476	-0.08
**Hyperactivity**	Male	MELS 19 Maximum	279.28 (63.97)	277.42 (63.57)	0.4747	0.03
**Hyperactivity**	Male	MELS 19 Range	563.47 (117.43)	556.03 (116.41)	0.1191	0.06
**Hyperactivity**	Male	MELS 19 Mean	-0.07 (1.08)	-0.05 (1.33)	0.6964	-0.02
**Hyperactivity**	Male	MELS 19 Std	75.17 (6.62)	74.66 (7.19)	0.0798	0.07
**Hyperactivity**	Male	MELS 19 Skewness	0.00 (0.15)	0.01 (0.17)	0.4441	-0.03
**Hyperactivity**	Male	MELS 19 Kurtosis	0.64 (0.56)	0.64 (0.59)	0.7681	-0.01
**Hyperactivity**	Male	MELS 20 Minimum	-298.03 (67.80)	-298.09 (68.07)	0.9831	0.00
**Hyperactivity**	Male	MELS 20 Maximum	316.24 (75.60)	314.71 (76.71)	0.6251	0.02
**Hyperactivity**	Male	MELS 20 Range	614.27 (126.40)	612.80 (129.12)	0.7799	0.01
**Hyperactivity**	Male	MELS 20 Mean	0.00 (1.05)	0.01 (1.20)	0.9809	0.00
**Hyperactivity**	Male	MELS 20 Std	81.54 (6.98)	81.23 (7.52)	0.3098	0.04
**Hyperactivity**	Male	MELS 20 Skewness	0.06 (0.15)	0.06 (0.16)	0.5743	-0.02
**Hyperactivity**	Male	MELS 20 Kurtosis	0.68 (0.54)	0.72 (0.62)	0.1315	-0.06
**Hyperactivity**	Male	MELS 21 Minimum	-304.43 (67.74)	-300.16 (67.97)	0.1244	-0.06
**Hyperactivity**	Male	MELS 21 Maximum	308.92 (67.91)	305.19 (71.77)	0.2003	0.05
**Hyperactivity**	Male	MELS 21 Range	613.36 (120.72)	605.35 (124.88)	0.1155	0.07
**Hyperactivity**	Male	MELS 21 Mean	-0.06 (1.34)	0.07 (1.34)	0.0187	-0.10
**Hyperactivity**	Male	MELS 21 Std	82.05 (6.60)	81.67 (7.27)	0.1982	0.05
**Hyperactivity**	Male	MELS 21 Skewness	0.02 (0.13)	0.02 (0.14)	0.7686	0.01

**Hyperactivity**	Male	MELS 21 Kurtosis	0.62 (0.49)	0.62 (0.53)	0.8399	0.01
**Hyperactivity**	Male	MELS 22 Minimum	-295.85 (60.45)	-289.97 (62.18)	0.0204	-0.10
**Hyperactivity**	Male	MELS 22 Maximum	287.03 (55.65)	282.47 (58.89)	0.0563	0.08
**Hyperactivity**	Male	MELS 22 Range	582.88 (100.08)	572.44 (105.79)	0.0150	0.10
**Hyperactivity**	Male	MELS 22 Mean	-0.01 (1.22)	0.05 (1.27)	0.2436	-0.05
**Hyperactivity**	Male	MELS 22 Std	80.08 (6.07)	79.36 (6.29)	0.0049	0.12
**Hyperactivity**	Male	MELS 22 Skewness	-0.02 (0.13)	-0.02 (0.15)	0.9708	0.00
**Hyperactivity**	Male	MELS 22 Kurtosis	0.50 (0.42)	0.49 (0.44)	0.8940	0.01
**Hyperactivity**	Male	MELS 23 Minimum	-273.74 (53.83)	-272.58 (54.33)	0.6013	-0.02
**Hyperactivity**	Male	MELS 23 Maximum	275.89 (56.28)	271.18 (55.07)	0.0375	0.08
**Hyperactivity**	Male	MELS 23 Range	549.63 (95.65)	543.76 (95.92)	0.1349	0.06
**Hyperactivity**	Male	MELS 23 Mean	-0.01 (0.73)	0.04 (1.41)	0.3570	-0.04
**Hyperactivity**	Male	MELS 23 Std	76.89 (5.46)	76.42 (5.77)	0.0452	0.08
**Hyperactivity**	Male	MELS 23 Skewness	-0.00 (0.12)	-0.01 (0.13)	0.1194	0.07
**Hyperactivity**	Male	MELS 23 Kurtosis	0.42 (0.38)	0.43 (0.42)	0.4941	-0.03
**Hyperactivity**	Male	MELS 24 Minimum	-255.43 (46.94)	-254.04 (50.24)	0.4969	-0.03
**Hyperactivity**	Male	MELS 24 Maximum	257.25 (47.06)	256.43 (50.89)	0.6919	0.02
**Hyperactivity**	Male	MELS 24 Range	512.67 (83.12)	510.47 (88.82)	0.5413	0.03
**Hyperactivity**	Male	MELS 24 Mean	-0.01 (0.81)	0.05 (1.33)	0.2350	-0.06
**Hyperactivity**	Male	MELS 24 Std	72.77 (5.07)	72.23 (5.54)	0.0150	0.10
**Hyperactivity**	Male	MELS 24 Skewness	0.01 (0.12)	0.01 (0.13)	0.1973	-0.05
**Hyperactivity**	Male	MELS 24 Kurtosis	0.37 (0.39)	0.40 (0.40)	0.0292	-0.09
**Hyperactivity**	Male	MELS 25 Minimum	-241.49 (45.80)	-239.85 (47.22)	0.3958	-0.04
**Hyperactivity**	Male	MELS 25 Maximum	239.58 (44.38)	236.81 (46.44)	0.1435	0.06
**Hyperactivity**	Male	MELS 25 Range	481.06 (78.56)	476.67 (81.98)	0.1874	0.05
**Hyperactivity**	Male	MELS 25 Mean	0.02 (0.98)	0.07 (1.07)	0.2441	-0.05
**Hyperactivity**	Male	MELS 25 Std	68.20 (4.77)	67.60 (5.12)	0.0036	0.12
**Hyperactivity**	Male	MELS 25 Skewness	0.01 (0.13)	-0.01 (0.13)	0.0244	0.09
**Hyperactivity**	Male	MELS 25 Kurtosis	0.38 (0.38)	0.39 (0.42)	0.4518	-0.03
**Hyperactivity**	Male	MELS 26 Minimum	-220.79 (41.44)	-218.91 (42.25)	0.2765	-0.04
**Hyperactivity**	Male	MELS 26 Maximum	220.44 (39.90)	218.41 (41.77)	0.2309	0.05
**Hyperactivity**	Male	MELS 26 Range	441.23 (70.66)	437.32 (73.65)	0.1918	0.05
**Hyperactivity**	Male	MELS 26 Mean	-0.04 (1.06)	0.03 (0.97)	0.1113	-0.06
**Hyperactivity**	Male	MELS 26 Std	62.96 (4.85)	62.56 (4.85)	0.0421	0.08
**Hyperactivity**	Male	MELS 26 Skewness	-0.00 (0.11)	-0.01 (0.12)	0.3676	0.04
**Hyperactivity**	Male	MELS 26 Kurtosis	0.36 (0.38)	0.36 (0.38)	0.6443	-0.02
**Hyperactivity**	Male	F1 Minimum	213.87 (38.04)	220.74 (75.99)	0.0184	-0.11
**Hyperactivity**	Male	F1 Maximum	4551.87 (875.50)	4600.38 (916.74)	0.1930	-0.05
**Hyperactivity**	Male	F1 Range	4338.00 (881.40)	4379.64 (922.85)	0.2671	-0.05
**Hyperactivity**	Male	F1 Mean	993.39 (388.15)	1072.80 (465.41)	0.0000	-0.19

**Hyperactivity**	Male	F1 Std	876.22 (231.53)	908.58 (259.37)	0.0019	-0.13
**Hyperactivity**	Male	F1 Skewness	2.05 (0.97)	1.94 (1.07)	0.0070	0.11
**Hyperactivity**	Male	F1 Kurtosis	5.00 (5.00)	4.73 (5.43)	0.2173	0.05
**Hyperactivity**	Male	F2 Minimum	586.49 (404.72)	618.87 (446.54)	0.0723	-0.08
**Hyperactivity**	Male	F2 Maximum	6303.87 (581.62)	6301.35 (566.44)	0.9141	0.00
**Hyperactivity**	Male	F2 Range	5717.38 (787.59)	5682.48 (792.95)	0.2820	0.04
**Hyperactivity**	Male	F2 Mean	2489.14 (577.98)	2586.08 (664.99)	0.0003	-0.16
**Hyperactivity**	Male	F2 Std	1221.57 (195.35)	1247.01 (227.35)	0.0052	-0.12
**Hyperactivity**	Male	F2 Skewness	0.90 (0.54)	0.83 (0.62)	0.0046	0.12
**Hyperactivity**	Male	F2 Kurtosis	0.51 (1.25)	0.46 (1.32)	0.3385	0.04
**Hyperactivity**	Male	F3 Minimum	1834.33 (671.95)	1927.45 (769.71)	0.0026	-0.13
**Hyperactivity**	Male	F3 Maximum	7003.53 (402.06)	6972.48 (413.87)	0.0656	0.08
**Hyperactivity**	Male	F3 Range	5169.21 (934.85)	5045.02 (1026.31)	0.0027	0.13
**Hyperactivity**	Male	F3 Mean	4348.22 (465.10)	4392.38 (521.25)	0.0354	-0.09
**Hyperactivity**	Male	F3 Std	1217.86 (192.35)	1183.97 (216.10)	0.0001	0.17
**Hyperactivity**	Male	F3 Skewness	0.11 (0.54)	0.08 (0.61)	0.2614	0.05
**Hyperactivity**	Male	F3 Kurtosis	-0.61 (0.96)	-0.47 (1.11)	0.0024	-0.13
**Hyperactivity**	Male	F1 1st Derivative Minim	-1891.43 (466.55)	-1873.32 (468.12)	0.3445	-0.04
**Hyperactivity**	Male	F1 1st Derivative Maxin	1893.42 (469.64)	1871.70 (483.63)	0.2705	0.05
**Hyperactivity**	Male	F1 1st Derivative Range	3784.85 (884.83)	3745.02 (896.94)	0.2771	0.04
**Hyperactivity**	Male	F1 1st Derivative Mean	-2.41 (23.71)	-5.71 (24.67)	0.0010	0.14
**Hyperactivity**	Male	F1 1st Derivative Std	489.79 (119.56)	494.38 (127.79)	0.3752	-0.04
**Hyperactivity**	Male	F1 1st Derivative Skewı	0.05 (0.46)	0.04 (0.47)	0.9159	0.00
**Hyperactivity**	Male	F1 1st Derivative Kurtos	5.60 (3.78)	5.61 (4.10)	0.9467	0.00
**Hyperactivity**	Male	F2 1st Derivative Minim	-2259.30 (456.84)	-2201.23 (471.28)	0.0025	-0.13
**Hyperactivity**	Male	F2 1st Derivative Maxin	2233.67 (492.26)	2179.22 (504.96)	0.0082	0.11
**Hyperactivity**	Male	F2 1st Derivative Range	4492.96 (877.80)	4380.45 (901.09)	0.0022	0.13
**Hyperactivity**	Male	F2 1st Derivative Mean	-5.52 (31.45)	-8.78 (32.46)	0.0137	0.10
**Hyperactivity**	Male	F2 1st Derivative Std	667.97 (108.35)	664.39 (110.49)	0.4283	0.03
**Hyperactivity**	Male	F2 1st Derivative Skewı	0.01 (0.31)	-0.01 (0.35)	0.1628	0.06
**Hyperactivity**	Male	F2 1st Derivative Kurtos	2.20 (1.35)	2.21 (1.45)	0.8746	-0.01
**Hyperactivity**	Male	F3 1st Derivative Minim	-2035.11 (460.93)	-1960.32 (492.60)	0.0002	-0.16
**Hyperactivity**	Male	F3 1st Derivative Maxin	2022.22 (491.70)	1958.06 (532.00)	0.0029	0.13
**Hyperactivity**	Male	F3 1st Derivative Range	4057.33 (899.46)	3918.38 (970.86)	0.0004	0.15
**Hyperactivity**	Male	F3 1st Derivative Mean	-4.61 (33.40)	-6.99 (39.45)	0.1309	0.07
**Hyperactivity**	Male	F3 1st Derivative Std	698.03 (126.20)	677.00 (137.71)	0.0002	0.16
**Hyperactivity**	Male	F3 1st Derivative Skewı	-0.01 (0.32)	-0.02 (0.36)	0.5725	0.02
**Hyperactivity**	Male	F3 1st Derivative Kurtos	1.00 (0.84)	1.06 (0.97)	0.0885	-0.07
**Hyperactivity**	Male	F1 2nd Derivative Minin	-6583.62 (2041.13)	-6435.47 (2063.62)	0.0790	-0.07
**Hyperactivity**	Male	F1 2nd Derivative Maxiı	4711.49 (1364.09)	4712.89 (1453.97)	0.9810	0.00

**Hyperactivity**	Male	F1 2nd Derivative Rang	11295.11 (3096.57)	11148.36 (3177.57)	0.2574	0.05
**Hyperactivity**	Male	F1 2nd Derivative Mear	-0.40 (43.20)	-0.26 (43.00)	0.9373	0.00
**Hyperactivity**	Male	F1 2nd Derivative Std	1245.85 (337.64)	1265.30 (380.72)	0.2041	-0.05
**Hyperactivity**	Male	F1 2nd Derivative Skew	-0.79 (0.62)	-0.76 (0.65)	0.2469	-0.05
**Hyperactivity**	Male	F1 2nd Derivative Kurtc	8.59 (6.18)	8.51 (6.28)	0.7603	0.01
**Hyperactivity**	Male	F2 2nd Derivative Minin	-7701.82 (1988.78)	-7389.80 (2083.61)	0.0002	-0.15
**Hyperactivity**	Male	F2 2nd Derivative Maxii	6139.03 (1678.97)	5954.61 (1724.48)	0.0087	0.11
**Hyperactivity**	Male	F2 2nd Derivative Rang	13840.85 (3313.11)	13344.41 (3438.53)	0.0004	0.15
**Hyperactivity**	Male	F2 2nd Derivative Mear	-0.36 (53.56)	1.50 (55.73)	0.4126	-0.03
**Hyperactivity**	Male	F2 2nd Derivative Std	1731.50 (319.29)	1718.57 (348.15)	0.3580	0.04
**Hyperactivity**	Male	F2 2nd Derivative Skew	-0.54 (0.40)	-0.51 (0.44)	0.0794	-0.07
**Hyperactivity**	Male	F2 2nd Derivative Kurtc	4.20 (2.47)	4.06 (2.54)	0.1825	0.06
**Hyperactivity**	Male	F3 2nd Derivative Minin	-6544.60 (1871.28)	-6284.41 (2004.33)	0.0014	-0.13
**Hyperactivity**	Male	F3 2nd Derivative Maxii	6354.67 (1899.32)	6043.88 (2017.31)	0.0001	0.16
**Hyperactivity**	Male	F3 2nd Derivative Rang	12899.27 (3499.32)	12328.29 (3758.27)	0.0002	0.16
**Hyperactivity**	Male	F3 2nd Derivative Mear	3.00 (69.18)	2.13 (70.74)	0.7616	0.01
**Hyperactivity**	Male	F3 2nd Derivative Std	1805.85 (357.24)	1746.03 (387.18)	0.0001	0.16
**Hyperactivity**	Male	F3 2nd Derivative Skew	-0.16 (0.38)	-0.16 (0.42)	0.9352	0.00
**Hyperactivity**	Male	F3 2nd Derivative Kurtc	2.14 (1.28)	2.25 (1.49)	0.0677	-0.08
**Emotional Symptoms**	Male	F0 Minimum	58.98 (10.95)	58.94 (12.99)	0.9273	0.00
**Emotional Symptoms**	Male	F0 Maximum	483.33 (2.25)	483.02 (5.33)	0.0726	0.08
**Emotional Symptoms**	Male	F0 Range	424.35 (11.64)	424.08 (15.82)	0.6174	0.02
**Emotional Symptoms**	Male	F0 Mean	281.90 (15.55)	281.55 (16.85)	0.5581	0.02
**Emotional Symptoms**	Male	F0 Std	113.02 (6.66)	112.98 (7.74)	0.8730	0.01
**Emotional Symptoms**	Male	F0 Skewness	0.13 (0.17)	0.13 (0.17)	0.8872	-0.01
**Emotional Symptoms**	Male	F0 Kurtosis	-1.15 (0.22)	-1.13 (0.27)	0.1228	-0.06
**Emotional Symptoms**	Male	F0 de Minimum	-177.85 (17.08)	-177.44 (18.89)	0.5334	-0.02
**Emotional Symptoms**	Male	F0 de Maximum	187.74 (12.68)	186.88 (14.98)	0.1001	0.06
**Emotional Symptoms**	Male	F0 de Range	365.59 (25.22)	364.32 (29.29)	0.2136	0.05
**Emotional Symptoms**	Male	F0 de Mean	0.17 (0.62)	0.18 (0.68)	0.5619	-0.02
**Emotional Symptoms**	Male	F0 de Std	76.58 (12.46)	76.32 (13.51)	0.5940	0.02
**Emotional Symptoms**	Male	F0 de Skewness	0.32 (0.17)	0.32 (0.18)	0.5547	0.02
**Emotional Symptoms**	Male	F0 de Kurtosis	-0.49 (0.92)	-0.43 (1.05)	0.1219	-0.06
**Emotional Symptoms**	Male	F0 de2 Minimum	-651.08 (55.96)	-648.85 (62.66)	0.3097	-0.04
**Emotional Symptoms**	Male	F0 de2 Maximum	613.62 (75.49)	610.89 (79.11)	0.3305	0.04
**Emotional Symptoms**	Male	F0 de2 Range	1264.70 (120.65)	1259.73 (131.30)	0.2833	0.04
**Emotional Symptoms**	Male	F0 de2 Mean	0.06 (1.26)	0.11 (1.56)	0.3903	-0.03
**Emotional Symptoms**	Male	F0 de2 Std	298.97 (23.76)	298.58 (25.32)	0.6658	0.02
**Emotional Symptoms**	Male	F0 de2 Skewness	-0.05 (0.05)	-0.06 (0.05)	0.2630	0.04
**Emotional Symptoms**	Male	F0 de2 Kurtosis	-1.10 (0.25)	-1.08 (0.28)	0.1184	-0.06

**Emotional Symptoms**	Male	Energy Minimum	-11.85 (6.73)	-11.41 (6.13)	0.0493	-0.07
**Emotional Symptoms**	Male	Energy Maximum	3.83 (1.26)	3.87 (1.26)	0.3929	-0.03
**Emotional Symptoms**	Male	Energy Range	15.68 (7.20)	15.28 (6.53)	0.0923	0.06
**Emotional Symptoms**	Male	Energy Mean	-2.07 (1.02)	-2.06 (1.14)	0.8028	-0.01
**Emotional Symptoms**	Male	Energy Std	2.66 (0.62)	2.64 (0.60)	0.4619	0.03
**Emotional Symptoms**	Male	Energy Skewness	-0.77 (0.62)	-0.73 (0.68)	0.0687	-0.07
**Emotional Symptoms**	Male	Energy Kurtosis	1.59 (4.78)	1.57 (5.33)	0.9391	0.00
**Emotional Symptoms**	Male	Energy de Minimum	-3.58 (1.99)	-3.43 (1.73)	0.0162	-0.08
**Emotional Symptoms**	Male	Energy de Maximum	4.62 (2.32)	4.49 (2.13)	0.0814	0.06
**Emotional Symptoms**	Male	Energy de Range	8.21 (4.03)	7.92 (3.55)	0.0274	0.08
**Emotional Symptoms**	Male	Energy de Mean	0.01 (0.03)	0.01 (0.03)	0.2576	-0.04
**Emotional Symptoms**	Male	Energy de Std	1.12 (0.22)	1.11 (0.23)	0.0659	0.07
**Emotional Symptoms**	Male	Energy de Skewness	0.38 (0.46)	0.42 (0.54)	0.0553	-0.07
**Emotional Symptoms**	Male	Energy de Kurtosis	1.74 (3.55)	1.79 (3.73)	0.7279	-0.01
**Emotional Symptoms**	Male	Energy de2 Minimum	-7.25 (4.31)	-6.95 (3.84)	0.0400	-0.07
**Emotional Symptoms**	Male	Energy de2 Maximum	8.79 (7.08)	8.26 (5.99)	0.0176	0.08
**Emotional Symptoms**	Male	Energy de2 Range	16.04 (11.09)	15.21 (9.53)	0.0205	0.08
**Emotional Symptoms**	Male	Energy de2 Mean	-0.01 (0.02)	-0.01 (0.03)	0.0800	0.07
**Emotional Symptoms**	Male	Energy de2 Std	1.57 (0.30)	1.54 (0.32)	0.0737	0.07
**Emotional Symptoms**	Male	Energy de2 Skewness	0.73 (0.77)	0.70 (0.68)	0.2504	0.04
**Emotional Symptoms**	Male	Energy de2 Kurtosis	7.32 (23.58)	5.96 (18.61)	0.0564	0.06
**Emotional Symptoms**	Male	MFCC 1 Minimum	-11.85 (6.73)	-11.41 (6.13)	0.0493	-0.07
**Emotional Symptoms**	Male	MFCC 1 Maximum	3.83 (1.26)	3.87 (1.26)	0.3929	-0.03
**Emotional Symptoms**	Male	MFCC 1 Range	15.68 (7.20)	15.28 (6.53)	0.0923	0.06
**Emotional Symptoms**	Male	MFCC 1 Mean	-2.07 (1.02)	-2.06 (1.14)	0.8028	-0.01
**Emotional Symptoms**	Male	MFCC 1 Std	2.66 (0.62)	2.64 (0.60)	0.4619	0.03
**Emotional Symptoms**	Male	MFCC 1 Skewness	-0.77 (0.62)	-0.73 (0.68)	0.0687	-0.07
**Emotional Symptoms**	Male	MFCC 1 Kurtosis	1.59 (4.78)	1.57 (5.33)	0.9391	0.00
**Emotional Symptoms**	Male	MFCC 2 Minimum	-482.13 (98.72)	-473.88 (105.26)	0.0270	-0.08
**Emotional Symptoms**	Male	MFCC 2 Maximum	357.57 (57.57)	350.49 (62.72)	0.0014	0.12
**Emotional Symptoms**	Male	MFCC 2 Range	839.70 (143.57)	824.37 (156.22)	0.0055	0.10
**Emotional Symptoms**	Male	MFCC 2 Mean	42.94 (22.22)	40.60 (24.07)	0.0059	0.10
**Emotional Symptoms**	Male	MFCC 2 Std	158.05 (25.11)	155.15 (27.64)	0.0030	0.11
**Emotional Symptoms**	Male	MFCC 2 Skewness	-0.70 (0.26)	-0.68 (0.29)	0.0977	-0.06
**Emotional Symptoms**	Male	MFCC 2 Kurtosis	0.29 (0.46)	0.30 (0.50)	0.5720	-0.02
**Emotional Symptoms**	Male	MFCC 3 Minimum	-396.30 (107.92)	-388.14 (114.57)	0.0448	-0.07
**Emotional Symptoms**	Male	MFCC 3 Maximum	413.09 (72.12)	407.88 (77.32)	0.0569	0.07
**Emotional Symptoms**	Male	MFCC 3 Range	809.39 (164.37)	796.02 (175.89)	0.0319	0.08
**Emotional Symptoms**	Male	MFCC 3 Mean	28.69 (27.43)	30.26 (29.57)	0.1357	-0.05
**Emotional Symptoms**	Male	MFCC 3 Std	148.41 (26.22)	146.85 (28.21)	0.1185	0.06

**Emotional Symptoms**	Male	MFCC 3 Skewness	-0.19 (0.24)	-0.21 (0.24)	0.1113	0.06
**Emotional Symptoms**	Male	MFCC 3 Kurtosis	-0.30 (0.33)	-0.30 (0.36)	0.9510	0.00
**Emotional Symptoms**	Male	MFCC 4 Minimum	-464.60 (107.83)	-454.44 (117.28)	0.0142	-0.09
**Emotional Symptoms**	Male	MFCC 4 Maximum	551.28 (113.04)	542.31 (112.70)	0.0269	0.08
**Emotional Symptoms**	Male	MFCC 4 Range	1015.88 (199.17)	996.74 (208.97)	0.0101	0.09
**Emotional Symptoms**	Male	MFCC 4 Mean	19.30 (30.05)	22.20 (32.26)	0.0110	-0.09
**Emotional Symptoms**	Male	MFCC 4 Std	188.51 (31.03)	185.82 (32.74)	0.0210	0.08
**Emotional Symptoms**	Male	MFCC 4 Skewness	0.16 (0.26)	0.16 (0.25)	0.7121	0.01
**Emotional Symptoms**	Male	MFCC 4 Kurtosis	-0.27 (0.34)	-0.28 (0.37)	0.5587	0.02
**Emotional Symptoms**	Male	MFCC 5 Minimum	-463.88 (126.73)	-448.01 (128.97)	0.0006	-0.12
**Emotional Symptoms**	Male	MFCC 5 Maximum	403.63 (88.84)	395.10 (92.58)	0.0097	0.09
**Emotional Symptoms**	Male	MFCC 5 Range	867.51 (192.22)	843.11 (198.14)	0.0006	0.13
**Emotional Symptoms**	Male	MFCC 5 Mean	-7.83 (30.23)	-6.57 (30.46)	0.2455	-0.04
**Emotional Symptoms**	Male	MFCC 5 Std	144.01 (25.86)	140.86 (27.07)	0.0011	0.12
**Emotional Symptoms**	Male	MFCC 5 Skewness	-0.15 (0.24)	-0.15 (0.25)	0.8089	-0.01
**Emotional Symptoms**	Male	MFCC 5 Kurtosis	0.01 (0.41)	-0.00 (0.42)	0.3667	0.03
**Emotional Symptoms**	Male	MFCC 6 Minimum	-450.99 (106.32)	-446.67 (115.48)	0.2894	-0.04
**Emotional Symptoms**	Male	MFCC 6 Maximum	408.63 (92.38)	401.78 (97.90)	0.0485	0.07
**Emotional Symptoms**	Male	MFCC 6 Range	859.62 (169.25)	848.44 (185.01)	0.0868	0.06
**Emotional Symptoms**	Male	MFCC 6 Mean	-13.24 (31.84)	-12.84 (32.05)	0.7265	-0.01
**Emotional Symptoms**	Male	MFCC 6 Std	141.83 (20.70)	140.67 (22.70)	0.1468	0.05
**Emotional Symptoms**	Male	MFCC 6 Skewness	-0.14 (0.24)	-0.15 (0.25)	0.4258	0.03
**Emotional Symptoms**	Male	MFCC 6 Kurtosis	0.04 (0.40)	0.02 (0.39)	0.2802	0.04
**Emotional Symptoms**	Male	MFCC 7 Minimum	-529.30 (122.00)	-517.31 (126.67)	0.0079	-0.10
**Emotional Symptoms**	Male	MFCC 7 Maximum	374.44 (90.97)	365.09 (93.39)	0.0051	0.10
**Emotional Symptoms**	Male	MFCC 7 Range	903.74 (180.77)	882.40 (191.84)	0.0017	0.11
**Emotional Symptoms**	Male	MFCC 7 Mean	-53.42 (34.49)	-53.11 (34.96)	0.8052	-0.01
**Emotional Symptoms**	Male	MFCC 7 Std	150.01 (23.19)	148.34 (24.62)	0.0552	0.07
**Emotional Symptoms**	Male	MFCC 7 Skewness	-0.20 (0.23)	-0.20 (0.23)	0.7306	0.01
**Emotional Symptoms**	Male	MFCC 7 Kurtosis	-0.01 (0.37)	-0.03 (0.39)	0.2630	0.04
**Emotional Symptoms**	Male	MFCC 8 Minimum	-521.46 (126.76)	-509.94 (128.67)	0.0125	-0.09
**Emotional Symptoms**	Male	MFCC 8 Maximum	361.07 (90.12)	353.53 (89.88)	0.0198	0.08
**Emotional Symptoms**	Male	MFCC 8 Range	882.52 (184.02)	863.48 (186.49)	0.0044	0.10
**Emotional Symptoms**	Male	MFCC 8 Mean	-60.93 (33.27)	-60.73 (35.03)	0.8712	-0.01
**Emotional Symptoms**	Male	MFCC 8 Std	143.82 (21.43)	142.71 (22.29)	0.1634	0.05
**Emotional Symptoms**	Male	MFCC 8 Skewness	-0.16 (0.25)	-0.16 (0.26)	0.4648	-0.03
**Emotional Symptoms**	Male	MFCC 8 Kurtosis	0.06 (0.41)	0.04 (0.40)	0.1799	0.05
**Emotional Symptoms**	Male	MFCC 9 Minimum	-438.17 (100.76)	-428.14 (98.19)	0.0047	-0.10
**Emotional Symptoms**	Male	MFCC 9 Maximum	375.20 (91.33)	366.19 (91.16)	0.0060	0.10
**Emotional Symptoms**	Male	MFCC 9 Range	813.37 (160.69)	794.33 (160.33)	0.0010	0.12

**Emotional Symptoms**	Male	MFCC 9 Mean	-31.83 (28.37)	-30.49 (29.11)	0.1970	-0.05
**Emotional Symptoms**	Male	MFCC 9 Std	133.77 (18.44)	132.67 (19.19)	0.1081	0.06
**Emotional Symptoms**	Male	MFCC 9 Skewness	-0.02 (0.24)	-0.02 (0.24)	0.7382	0.01
**Emotional Symptoms**	Male	MFCC 9 Kurtosis	-0.01 (0.40)	-0.04 (0.38)	0.0409	0.07
**Emotional Symptoms**	Male	MFCC 10 Minimum	-396.48 (88.61)	-385.82 (93.44)	0.0013	-0.12
**Emotional Symptoms**	Male	MFCC 10 Maximum	354.03 (91.02)	344.35 (93.79)	0.0039	0.10
**Emotional Symptoms**	Male	MFCC 10 Range	750.51 (154.88)	730.17 (162.34)	0.0004	0.13
**Emotional Symptoms**	Male	MFCC 10 Mean	-23.43 (24.60)	-23.79 (25.68)	0.6975	0.01
**Emotional Symptoms**	Male	MFCC 10 Std	121.90 (19.91)	119.78 (21.21)	0.0048	0.10
**Emotional Symptoms**	Male	MFCC 10 Skewness	0.01 (0.23)	0.00 (0.24)	0.3426	0.03
**Emotional Symptoms**	Male	MFCC 10 Kurtosis	0.04 (0.37)	0.03 (0.40)	0.3745	0.03
**Emotional Symptoms**	Male	MFCC 11 Minimum	-352.94 (81.72)	-347.92 (83.82)	0.0938	-0.06
**Emotional Symptoms**	Male	MFCC 11 Maximum	300.10 (82.00)	289.11 (82.15)	0.0002	0.13
**Emotional Symptoms**	Male	MFCC 11 Range	653.04 (136.99)	637.03 (139.99)	0.0014	0.12
**Emotional Symptoms**	Male	MFCC 11 Mean	-23.03 (25.13)	-24.43 (25.76)	0.1299	0.05
**Emotional Symptoms**	Male	MFCC 11 Std	102.85 (15.35)	101.21 (16.03)	0.0040	0.10
**Emotional Symptoms**	Male	MFCC 11 Skewness	-0.03 (0.25)	-0.03 (0.24)	0.5920	0.02
**Emotional Symptoms**	Male	MFCC 11 Kurtosis	0.15 (0.40)	0.14 (0.42)	0.3895	0.03
**Emotional Symptoms**	Male	MFCC 12 Minimum	-310.36 (69.82)	-304.46 (76.46)	0.0285	-0.08
**Emotional Symptoms**	Male	MFCC 12 Maximum	269.48 (66.01)	265.66 (69.11)	0.1210	0.06
**Emotional Symptoms**	Male	MFCC 12 Range	579.84 (112.16)	570.12 (122.77)	0.0248	0.08
**Emotional Symptoms**	Male	MFCC 12 Mean	-16.41 (21.35)	-15.77 (21.36)	0.4067	-0.03
**Emotional Symptoms**	Male	MFCC 12 Std	89.48 (11.91)	88.43 (13.29)	0.0247	0.08
**Emotional Symptoms**	Male	MFCC 12 Skewness	-0.06 (0.24)	-0.05 (0.25)	0.5398	-0.02
**Emotional Symptoms**	Male	MFCC 12 Kurtosis	0.24 (0.42)	0.24 (0.44)	0.8980	0.00
**Emotional Symptoms**	Male	MFCC 13 Minimum	-256.81 (64.15)	-249.22 (66.61)	0.0014	-0.12
**Emotional Symptoms**	Male	MFCC 13 Maximum	256.85 (62.51)	254.56 (64.75)	0.3220	0.04
**Emotional Symptoms**	Male	MFCC 13 Range	513.65 (104.20)	503.78 (109.23)	0.0111	0.09
**Emotional Symptoms**	Male	MFCC 13 Mean	-0.89 (18.11)	0.79 (18.68)	0.0118	-0.09
**Emotional Symptoms**	Male	MFCC 13 Std	77.64 (11.03)	77.03 (11.57)	0.1357	0.05
**Emotional Symptoms**	Male	MFCC 13 Skewness	0.03 (0.25)	0.04 (0.26)	0.4354	-0.03
**Emotional Symptoms**	Male	MFCC 13 Kurtosis	0.33 (0.43)	0.32 (0.46)	0.5614	0.02
**Emotional Symptoms**	Male	MELS 1 Minimum	-3.58 (1.99)	-3.43 (1.73)	0.0162	-0.08
**Emotional Symptoms**	Male	MELS 1 Maximum	4.62 (2.32)	4.49 (2.13)	0.0814	0.06
**Emotional Symptoms**	Male	MELS 1 Range	8.21 (4.03)	7.92 (3.55)	0.0274	0.08
**Emotional Symptoms**	Male	MELS 1 Mean	0.01 (0.03)	0.01 (0.03)	0.2576	-0.04
**Emotional Symptoms**	Male	MELS 1 Std	1.12 (0.22)	1.11 (0.23)	0.0659	0.07
**Emotional Symptoms**	Male	MELS 1 Skewness	0.38 (0.46)	0.42 (0.54)	0.0553	-0.07
**Emotional Symptoms**	Male	MELS 1 Kurtosis	1.74 (3.55)	1.79 (3.73)	0.7279	-0.01
**Emotional Symptoms**	Male	MELS 2 Minimum	-211.04 (44.96)	-207.46 (48.27)	0.0362	-0.08

**Emotional Symptoms**	Male	MELS 2 Maximum	195.87 (40.77)	192.86 (44.48)	0.0546	0.07
**Emotional Symptoms**	Male	MELS 2 Range	406.91 (81.45)	400.31 (88.21)	0.0343	0.08
**Emotional Symptoms**	Male	MELS 2 Mean	0.13 (0.79)	0.14 (0.88)	0.8159	-0.01
**Emotional Symptoms**	Male	MELS 2 Std	58.55 (11.11)	57.69 (11.89)	0.0411	0.07
**Emotional Symptoms**	Male	MELS 2 Skewness	-0.11 (0.21)	-0.09 (0.26)	0.0375	-0.08
**Emotional Symptoms**	Male	MELS 2 Kurtosis	1.10 (0.57)	1.14 (0.67)	0.0819	-0.07
**Emotional Symptoms**	Male	MELS 3 Minimum	-175.46 (40.54)	-172.78 (42.86)	0.0783	-0.06
**Emotional Symptoms**	Male	MELS 3 Maximum	167.05 (39.84)	164.08 (42.57)	0.0489	0.07
**Emotional Symptoms**	Male	MELS 3 Range	342.51 (74.24)	336.86 (80.02)	0.0459	0.07
**Emotional Symptoms**	Male	MELS 3 Mean	0.09 (0.75)	0.05 (0.81)	0.2543	0.04
**Emotional Symptoms**	Male	MELS 3 Std	49.95 (8.27)	49.62 (8.95)	0.2987	0.04
**Emotional Symptoms**	Male	MELS 3 Skewness	-0.12 (0.18)	-0.13 (0.19)	0.1965	0.05
**Emotional Symptoms**	Male	MELS 3 Kurtosis	0.46 (0.41)	0.45 (0.42)	0.4728	0.03
**Emotional Symptoms**	Male	MELS 4 Minimum	-211.73 (46.02)	-206.66 (47.38)	0.0028	-0.11
**Emotional Symptoms**	Male	MELS 4 Maximum	206.77 (47.19)	201.54 (48.01)	0.0023	0.11
**Emotional Symptoms**	Male	MELS 4 Range	418.50 (82.73)	408.20 (86.67)	0.0008	0.12
**Emotional Symptoms**	Male	MELS 4 Mean	0.06 (0.87)	0.06 (1.02)	0.8645	-0.01
**Emotional Symptoms**	Male	MELS 4 Std	60.52 (8.50)	59.86 (8.91)	0.0352	0.08
**Emotional Symptoms**	Male	MELS 4 Skewness	-0.07 (0.20)	-0.07 (0.19)	0.9476	0.00
**Emotional Symptoms**	Male	MELS 4 Kurtosis	0.45 (0.50)	0.42 (0.47)	0.0984	0.06
**Emotional Symptoms**	Male	MELS 5 Minimum	-183.76 (43.13)	-177.83 (44.02)	0.0002	-0.14
**Emotional Symptoms**	Male	MELS 5 Maximum	173.79 (38.16)	169.24 (39.78)	0.0013	0.12
**Emotional Symptoms**	Male	MELS 5 Range	357.55 (73.87)	347.07 (76.80)	0.0001	0.14
**Emotional Symptoms**	Male	MELS 5 Mean	0.07 (0.67)	0.05 (0.83)	0.4450	0.03
**Emotional Symptoms**	Male	MELS 5 Std	51.80 (7.42)	50.96 (7.76)	0.0022	0.11
**Emotional Symptoms**	Male	MELS 5 Skewness	-0.06 (0.16)	-0.06 (0.17)	0.5320	-0.02
**Emotional Symptoms**	Male	MELS 5 Kurtosis	0.38 (0.36)	0.35 (0.42)	0.0693	0.07
**Emotional Symptoms**	Male	MELS 6 Minimum	-183.14 (39.35)	-179.94 (41.56)	0.0302	-0.08
**Emotional Symptoms**	Male	MELS 6 Maximum	181.45 (39.64)	178.27 (40.79)	0.0293	0.08
**Emotional Symptoms**	Male	MELS 6 Range	364.59 (70.76)	358.21 (74.94)	0.0165	0.09
**Emotional Symptoms**	Male	MELS 6 Mean	0.05 (0.79)	0.07 (0.80)	0.4330	-0.03
**Emotional Symptoms**	Male	MELS 6 Std	52.22 (6.17)	51.73 (6.86)	0.0401	0.08
**Emotional Symptoms**	Male	MELS 6 Skewness	-0.01 (0.17)	-0.01 (0.18)	0.8069	0.01
**Emotional Symptoms**	Male	MELS 6 Kurtosis	0.42 (0.44)	0.42 (0.39)	0.7081	-0.01
**Emotional Symptoms**	Male	MELS 7 Minimum	-201.12 (44.70)	-197.16 (46.34)	0.0166	-0.09
**Emotional Symptoms**	Male	MELS 7 Maximum	193.37 (43.77)	187.77 (44.02)	0.0004	0.13
**Emotional Symptoms**	Male	MELS 7 Range	394.49 (79.78)	384.92 (82.37)	0.0011	0.12
**Emotional Symptoms**	Male	MELS 7 Mean	-0.00 (0.58)	0.04 (0.72)	0.1045	-0.06
**Emotional Symptoms**	Male	MELS 7 Std	55.73 (6.99)	55.19 (7.51)	0.0444	0.07
**Emotional Symptoms**	Male	MELS 7 Skewness	-0.06 (0.17)	-0.08 (0.18)	0.0031	0.11

**Emotional Symptoms**	Male	MELS 7 Kurtosis	0.50 (0.40)	0.48 (0.42)	0.3280	0.04
**Emotional Symptoms**	Male	MELS 8 Minimum	-194.22 (43.39)	-190.89 (46.23)	0.0427	-0.07
**Emotional Symptoms**	Male	MELS 8 Maximum	189.23 (43.18)	185.66 (44.27)	0.0240	0.08
**Emotional Symptoms**	Male	MELS 8 Range	383.45 (78.77)	376.55 (82.59)	0.0190	0.09
**Emotional Symptoms**	Male	MELS 8 Mean	-0.05 (0.58)	-0.03 (0.87)	0.6483	-0.02
**Emotional Symptoms**	Male	MELS 8 Std	53.94 (6.73)	53.43 (7.13)	0.0447	0.07
**Emotional Symptoms**	Male	MELS 8 Skewness	-0.04 (0.17)	-0.04 (0.18)	0.9033	0.00
**Emotional Symptoms**	Male	MELS 8 Kurtosis	0.51 (0.41)	0.50 (0.43)	0.6610	0.02
**Emotional Symptoms**	Male	MELS 9 Minimum	-180.92 (38.93)	-175.60 (39.87)	0.0002	-0.14
**Emotional Symptoms**	Male	MELS 9 Maximum	178.83 (36.92)	175.33 (39.52)	0.0125	0.09
**Emotional Symptoms**	Male	MELS 9 Range	359.75 (67.67)	350.93 (71.72)	0.0005	0.13
**Emotional Symptoms**	Male	MELS 9 Mean	0.02 (0.86)	-0.03 (0.68)	0.0593	0.06
**Emotional Symptoms**	Male	MELS 9 Std	51.13 (5.68)	50.46 (5.97)	0.0015	0.12
**Emotional Symptoms**	Male	MELS 9 Skewness	0.00 (0.16)	0.01 (0.17)	0.4071	-0.03
**Emotional Symptoms**	Male	MELS 9 Kurtosis	0.44 (0.39)	0.42 (0.42)	0.2663	0.04
**Emotional Symptoms**	Male	MELS 10 Minimum	-167.25 (36.76)	-162.85 (38.78)	0.0014	-0.12
**Emotional Symptoms**	Male	MELS 10 Maximum	163.04 (34.32)	159.15 (36.47)	0.0027	0.11
**Emotional Symptoms**	Male	MELS 10 Range	330.29 (63.34)	322.01 (68.11)	0.0006	0.13
**Emotional Symptoms**	Male	MELS 10 Mean	0.01 (0.72)	0.02 (0.81)	0.7226	-0.01
**Emotional Symptoms**	Male	MELS 10 Std	46.71 (5.13)	46.11 (5.65)	0.0028	0.11
**Emotional Symptoms**	Male	MELS 10 Skewness	-0.01 (0.15)	-0.02 (0.17)	0.4911	0.03
**Emotional Symptoms**	Male	MELS 10 Kurtosis	0.43 (0.37)	0.41 (0.39)	0.1283	0.06
**Emotional Symptoms**	Male	MELS 11 Minimum	-146.97 (32.13)	-144.49 (33.53)	0.0379	-0.08
**Emotional Symptoms**	Male	MELS 11 Maximum	145.45 (30.43)	141.51 (31.66)	0.0005	0.13
**Emotional Symptoms**	Male	MELS 11 Range	292.42 (55.63)	286.00 (58.79)	0.0021	0.11
**Emotional Symptoms**	Male	MELS 11 Mean	-0.02 (0.64)	0.00 (0.64)	0.3188	-0.04
**Emotional Symptoms**	Male	MELS 11 Std	41.69 (4.36)	41.18 (4.74)	0.0021	0.11
**Emotional Symptoms**	Male	MELS 11 Skewness	-0.01 (0.15)	-0.01 (0.16)	0.9774	0.00
**Emotional Symptoms**	Male	MELS 11 Kurtosis	0.39 (0.35)	0.38 (0.39)	0.2313	0.04
**Emotional Symptoms**	Male	MELS 12 Minimum	-130.19 (25.81)	-128.81 (28.31)	0.1673	-0.05
**Emotional Symptoms**	Male	MELS 12 Maximum	132.43 (26.79)	129.09 (29.15)	0.0012	0.12
**Emotional Symptoms**	Male	MELS 12 Range	262.61 (46.66)	257.90 (51.68)	0.0095	0.10
**Emotional Symptoms**	Male	MELS 12 Mean	-0.02 (0.49)	-0.02 (0.53)	0.8254	0.01
**Emotional Symptoms**	Male	MELS 12 Std	37.42 (3.68)	37.03 (4.19)	0.0075	0.10
**Emotional Symptoms**	Male	MELS 12 Skewness	0.00 (0.16)	-0.00 (0.17)	0.3249	0.04
**Emotional Symptoms**	Male	MELS 12 Kurtosis	0.41 (0.41)	0.39 (0.41)	0.2005	0.05
**Emotional Symptoms**	Male	MELS 13 Minimum	-116.19 (23.98)	-114.44 (24.85)	0.0477	-0.07
**Emotional Symptoms**	Male	MELS 13 Maximum	116.91 (24.11)	115.63 (25.82)	0.1611	0.05
**Emotional Symptoms**	Male	MELS 13 Range	233.10 (42.72)	230.07 (45.56)	0.0601	0.07
**Emotional Symptoms**	Male	MELS 13 Mean	0.00 (0.38)	0.00 (0.51)	0.9493	0.00

**Emotional Symptoms**	Male	MELS 13 Std	33.22 (3.37)	33.05 (3.67)	0.1813	0.05
**Emotional Symptoms**	Male	MELS 13 Skewness	0.01 (0.15)	0.02 (0.16)	0.2860	-0.04
**Emotional Symptoms**	Male	MELS 13 Kurtosis	0.39 (0.35)	0.39 (0.36)	0.7807	0.01
**Emotional Symptoms**	Male	MELS 14 Minimum	-7.25 (4.31)	-6.95 (3.84)	0.0400	-0.07
**Emotional Symptoms**	Male	MELS 14 Maximum	8.79 (7.08)	8.26 (5.99)	0.0176	0.08
**Emotional Symptoms**	Male	MELS 14 Range	16.04 (11.09)	15.21 (9.53)	0.0205	0.08
**Emotional Symptoms**	Male	MELS 14 Mean	-0.01 (0.02)	-0.01 (0.03)	0.0800	0.07
**Emotional Symptoms**	Male	MELS 14 Std	1.57 (0.30)	1.54 (0.32)	0.0737	0.07
**Emotional Symptoms**	Male	MELS 14 Skewness	0.73 (0.77)	0.70 (0.68)	0.2504	0.04
**Emotional Symptoms**	Male	MELS 14 Kurtosis	7.32 (23.58)	5.96 (18.61)	0.0564	0.06
**Emotional Symptoms**	Male	MELS 15 Minimum	-231.91 (62.40)	-225.63 (64.82)	0.0065	-0.10
**Emotional Symptoms**	Male	MELS 15 Maximum	341.36 (88.82)	331.70 (92.50)	0.0034	0.11
**Emotional Symptoms**	Male	MELS 15 Range	573.27 (138.06)	557.32 (144.72)	0.0020	0.11
**Emotional Symptoms**	Male	MELS 15 Mean	-0.02 (0.64)	0.01 (0.92)	0.3247	-0.04
**Emotional Symptoms**	Male	MELS 15 Std	63.57 (10.86)	63.04 (11.52)	0.1959	0.05
**Emotional Symptoms**	Male	MELS 15 Skewness	0.86 (0.34)	0.84 (0.35)	0.2128	0.05
**Emotional Symptoms**	Male	MELS 15 Kurtosis	3.40 (1.56)	3.31 (1.60)	0.1222	0.06
**Emotional Symptoms**	Male	MELS 16 Minimum	-254.96 (66.45)	-250.05 (68.94)	0.0461	-0.07
**Emotional Symptoms**	Male	MELS 16 Maximum	235.02 (61.82)	234.27 (63.13)	0.7386	0.01
**Emotional Symptoms**	Male	MELS 16 Range	489.98 (115.49)	484.32 (118.30)	0.1811	0.05
**Emotional Symptoms**	Male	MELS 16 Mean	-0.11 (0.88)	-0.15 (1.24)	0.3054	0.04
**Emotional Symptoms**	Male	MELS 16 Std	60.66 (7.42)	60.41 (8.10)	0.3839	0.03
**Emotional Symptoms**	Male	MELS 16 Skewness	-0.15 (0.20)	-0.14 (0.23)	0.0947	-0.06
**Emotional Symptoms**	Male	MELS 16 Kurtosis	1.12 (0.82)	1.14 (0.94)	0.4654	-0.03
**Emotional Symptoms**	Male	MELS 17 Minimum	-294.25 (71.47)	-288.97 (74.12)	0.0460	-0.07
**Emotional Symptoms**	Male	MELS 17 Maximum	287.79 (70.77)	283.23 (74.44)	0.0844	0.06
**Emotional Symptoms**	Male	MELS 17 Range	582.04 (127.62)	572.20 (134.71)	0.0398	0.08
**Emotional Symptoms**	Male	MELS 17 Mean	-0.08 (1.18)	-0.15 (1.37)	0.1267	0.06
**Emotional Symptoms**	Male	MELS 17 Std	72.86 (8.31)	72.25 (8.77)	0.0509	0.07
**Emotional Symptoms**	Male	MELS 17 Skewness	-0.08 (0.17)	-0.08 (0.19)	0.9244	0.00
**Emotional Symptoms**	Male	MELS 17 Kurtosis	1.03 (0.68)	1.04 (0.75)	0.7329	-0.01
**Emotional Symptoms**	Male	MELS 18 Minimum	-269.70 (60.11)	-266.55 (64.79)	0.1684	-0.05
**Emotional Symptoms**	Male	MELS 18 Maximum	269.88 (65.73)	264.58 (63.99)	0.0220	0.08
**Emotional Symptoms**	Male	MELS 18 Range	539.58 (113.03)	531.12 (114.39)	0.0392	0.07
**Emotional Symptoms**	Male	MELS 18 Mean	0.01 (0.96)	-0.02 (1.42)	0.6117	0.02
**Emotional Symptoms**	Male	MELS 18 Std	71.37 (6.80)	70.78 (7.14)	0.0212	0.08
**Emotional Symptoms**	Male	MELS 18 Skewness	-0.03 (0.16)	-0.03 (0.16)	0.4339	-0.03
**Emotional Symptoms**	Male	MELS 18 Kurtosis	0.68 (0.55)	0.68 (0.62)	0.9999	0.00
**Emotional Symptoms**	Male	MELS 19 Minimum	-282.07 (68.26)	-278.77 (68.94)	0.1824	-0.05
**Emotional Symptoms**	Male	MELS 19 Maximum	279.00 (60.85)	277.34 (64.44)	0.4674	0.03

**Emotional Symptoms**	Male	MELS 19 Range	561.07 (111.70)	556.11 (118.01)	0.2367	0.04
**Emotional Symptoms**	Male	MELS 19 Mean	-0.06 (0.94)	-0.06 (1.38)	0.9898	0.00
**Emotional Symptoms**	Male	MELS 19 Std	75.05 (6.72)	74.65 (7.21)	0.1222	0.06
**Emotional Symptoms**	Male	MELS 19 Skewness	0.00 (0.15)	0.01 (0.17)	0.2615	-0.04
**Emotional Symptoms**	Male	MELS 19 Kurtosis	0.63 (0.55)	0.64 (0.60)	0.4637	-0.03
**Emotional Symptoms**	Male	MELS 20 Minimum	-300.67 (65.99)	-297.30 (68.60)	0.1681	-0.05
**Emotional Symptoms**	Male	MELS 20 Maximum	316.19 (74.65)	314.59 (77.08)	0.5632	0.02
**Emotional Symptoms**	Male	MELS 20 Range	616.86 (123.38)	611.90 (130.20)	0.2835	0.04
**Emotional Symptoms**	Male	MELS 20 Mean	-0.04 (1.06)	0.02 (1.21)	0.1536	-0.05
**Emotional Symptoms**	Male	MELS 20 Std	81.59 (6.92)	81.19 (7.58)	0.1389	0.05
**Emotional Symptoms**	Male	MELS 20 Skewness	0.06 (0.14)	0.07 (0.16)	0.0474	-0.07
**Emotional Symptoms**	Male	MELS 20 Kurtosis	0.70 (0.53)	0.72 (0.63)	0.3298	-0.04
**Emotional Symptoms**	Male	MELS 21 Minimum	-304.28 (65.98)	-299.83 (68.49)	0.0686	-0.07
**Emotional Symptoms**	Male	MELS 21 Maximum	310.62 (69.79)	304.36 (71.51)	0.0144	0.09
**Emotional Symptoms**	Male	MELS 21 Range	614.90 (121.07)	604.20 (125.07)	0.0165	0.09
**Emotional Symptoms**	Male	MELS 21 Mean	-0.03 (1.32)	0.07 (1.35)	0.0396	-0.07
**Emotional Symptoms**	Male	MELS 21 Std	82.03 (6.52)	81.64 (7.35)	0.1254	0.06
**Emotional Symptoms**	Male	MELS 21 Skewness	0.02 (0.13)	0.02 (0.14)	0.7637	0.01
**Emotional Symptoms**	Male	MELS 21 Kurtosis	0.63 (0.50)	0.62 (0.53)	0.6789	0.02
**Emotional Symptoms**	Male	MELS 22 Minimum	-295.86 (59.40)	-289.46 (62.60)	0.0040	-0.11
**Emotional Symptoms**	Male	MELS 22 Maximum	286.73 (55.50)	282.16 (59.20)	0.0297	0.08
**Emotional Symptoms**	Male	MELS 22 Range	582.59 (98.20)	571.62 (106.75)	0.0036	0.11
**Emotional Symptoms**	Male	MELS 22 Mean	0.03 (1.01)	0.04 (1.33)	0.6833	-0.02
**Emotional Symptoms**	Male	MELS 22 Std	79.98 (5.81)	79.32 (6.38)	0.0037	0.11
**Emotional Symptoms**	Male	MELS 22 Skewness	-0.02 (0.14)	-0.02 (0.15)	0.4258	-0.03
**Emotional Symptoms**	Male	MELS 22 Kurtosis	0.50 (0.42)	0.49 (0.44)	0.3716	0.03
**Emotional Symptoms**	Male	MELS 23 Minimum	-274.46 (51.59)	-272.27 (55.01)	0.2609	-0.04
**Emotional Symptoms**	Male	MELS 23 Maximum	277.21 (53.48)	270.38 (55.74)	0.0006	0.13
**Emotional Symptoms**	Male	MELS 23 Range	551.67 (90.02)	542.65 (97.49)	0.0088	0.10
**Emotional Symptoms**	Male	MELS 23 Mean	0.01 (0.91)	0.03 (1.42)	0.6805	-0.02
**Emotional Symptoms**	Male	MELS 23 Std	76.94 (5.26)	76.36 (5.85)	0.0045	0.11
**Emotional Symptoms**	Male	MELS 23 Skewness	0.01 (0.13)	-0.01 (0.13)	0.0001	0.14
**Emotional Symptoms**	Male	MELS 23 Kurtosis	0.44 (0.40)	0.43 (0.42)	0.4483	0.03
**Emotional Symptoms**	Male	MELS 24 Minimum	-257.63 (47.20)	-253.26 (50.41)	0.0145	-0.09
**Emotional Symptoms**	Male	MELS 24 Maximum	257.89 (47.01)	256.17 (51.21)	0.3405	0.04
**Emotional Symptoms**	Male	MELS 24 Range	515.52 (82.43)	509.43 (89.44)	0.0538	0.07
**Emotional Symptoms**	Male	MELS 24 Mean	-0.02 (0.70)	0.05 (1.38)	0.1283	-0.06
**Emotional Symptoms**	Male	MELS 24 Std	72.50 (5.13)	72.26 (5.56)	0.2346	0.04
**Emotional Symptoms**	Male	MELS 24 Skewness	0.01 (0.12)	0.01 (0.13)	0.1474	-0.05
**Emotional Symptoms**	Male	MELS 24 Kurtosis	0.40 (0.36)	0.39 (0.41)	0.6783	0.02

**Emotional Symptoms**	Male	MELS 25 Minimum	-243.39 (45.30)	-239.14 (47.45)	0.0119	-0.09
**Emotional Symptoms**	Male	MELS 25 Maximum	239.86 (44.51)	236.49 (46.57)	0.0421	0.07
**Emotional Symptoms**	Male	MELS 25 Range	483.25 (77.47)	475.63 (82.52)	0.0092	0.10
**Emotional Symptoms**	Male	MELS 25 Mean	0.01 (0.83)	0.07 (1.11)	0.1424	-0.06
**Emotional Symptoms**	Male	MELS 25 Std	67.98 (4.71)	67.61 (5.17)	0.0445	0.07
**Emotional Symptoms**	Male	MELS 25 Skewness	0.00 (0.12)	-0.01 (0.13)	0.0518	0.07
**Emotional Symptoms**	Male	MELS 25 Kurtosis	0.40 (0.38)	0.38 (0.42)	0.3399	0.04
**Emotional Symptoms**	Male	MELS 26 Minimum	-221.04 (40.71)	-218.67 (42.52)	0.1170	-0.06
**Emotional Symptoms**	Male	MELS 26 Maximum	220.53 (41.05)	218.21 (41.59)	0.1194	0.06
**Emotional Symptoms**	Male	MELS 26 Range	441.57 (70.56)	436.88 (73.92)	0.0743	0.06
**Emotional Symptoms**	Male	MELS 26 Mean	-0.02 (0.73)	0.03 (1.05)	0.1302	-0.06
**Emotional Symptoms**	Male	MELS 26 Std	62.60 (4.69)	62.63 (4.90)	0.8901	-0.01
**Emotional Symptoms**	Male	MELS 26 Skewness	-0.01 (0.11)	-0.01 (0.12)	0.6413	-0.02
**Emotional Symptoms**	Male	MELS 26 Kurtosis	0.36 (0.36)	0.36 (0.38)	0.7211	-0.01
**Emotional Symptoms**	Male	F1 Minimum	214.51 (41.79)	221.14 (77.84)	0.0096	-0.11
**Emotional Symptoms**	Male	F1 Maximum	4578.86 (888.75)	4596.52 (916.63)	0.5895	-0.02
**Emotional Symptoms**	Male	F1 Range	4364.35 (894.34)	4375.38 (922.80)	0.7378	-0.01
**Emotional Symptoms**	Male	F1 Mean	1007.62 (397.85)	1075.44 (469.16)	0.0000	-0.16
**Emotional Symptoms**	Male	F1 Std	886.22 (242.72)	908.41 (258.75)	0.0156	-0.09
**Emotional Symptoms**	Male	F1 Skewness	2.04 (1.02)	1.93 (1.06)	0.0029	0.11
**Emotional Symptoms**	Male	F1 Kurtosis	5.07 (5.58)	4.69 (5.29)	0.0494	0.07
**Emotional Symptoms**	Male	F2 Minimum	575.04 (393.05)	625.10 (452.63)	0.0015	-0.12
**Emotional Symptoms**	Male	F2 Maximum	6308.00 (580.83)	6299.90 (565.33)	0.6923	0.01
**Emotional Symptoms**	Male	F2 Range	5732.96 (765.48)	5674.80 (799.49)	0.0411	0.07
**Emotional Symptoms**	Male	F2 Mean	2491.81 (587.04)	2593.70 (669.12)	0.0000	-0.16
**Emotional Symptoms**	Male	F2 Std	1227.21 (203.58)	1247.54 (227.82)	0.0111	-0.09
**Emotional Symptoms**	Male	F2 Skewness	0.89 (0.56)	0.82 (0.62)	0.0026	0.11
**Emotional Symptoms**	Male	F2 Kurtosis	0.52 (1.27)	0.45 (1.32)	0.1362	0.05
**Emotional Symptoms**	Male	F3 Minimum	1840.68 (714.85)	1933.63 (765.82)	0.0006	-0.13
**Emotional Symptoms**	Male	F3 Maximum	7002.47 (392.82)	6970.10 (417.43)	0.0289	0.08
**Emotional Symptoms**	Male	F3 Range	5161.79 (962.61)	5036.46 (1025.81)	0.0006	0.13
**Emotional Symptoms**	Male	F3 Mean	4352.05 (482.59)	4395.07 (521.04)	0.0196	-0.09
**Emotional Symptoms**	Male	F3 Std	1205.76 (204.76)	1184.64 (214.87)	0.0057	0.10
**Emotional Symptoms**	Male	F3 Skewness	0.12 (0.55)	0.08 (0.62)	0.0807	0.06
**Emotional Symptoms**	Male	F3 Kurtosis	-0.54 (1.05)	-0.48 (1.10)	0.1164	-0.06
**Emotional Symptoms**	Male	F1 1st Derivative Minim	-1890.98 (453.69)	-1871.89 (471.99)	0.2564	-0.04
**Emotional Symptoms**	Male	F1 1st Derivative Maxin	1897.00 (461.64)	1868.74 (487.01)	0.1025	0.06
**Emotional Symptoms**	Male	F1 1st Derivative Range	3787.98 (864.37)	3740.63 (903.79)	0.1411	0.05
**Emotional Symptoms**	Male	F1 1st Derivative Mean	-3.15 (21.34)	-5.78 (25.40)	0.0029	0.11
**Emotional Symptoms**	Male	F1 1st Derivative Std	491.67 (121.44)	494.21 (127.96)	0.5758	-0.02

**Emotional Symptoms**	Male	F1 1st Derivative Skewı	0.05 (0.44)	0.04 (0.47)	0.8937	0.00
**Emotional Symptoms**	Male	F1 1st Derivative Kurtos	5.68 (4.06)	5.59 (4.05)	0.5212	0.02
**Emotional Symptoms**	Male	F2 1st Derivative Minim	-2247.18 (449.20)	-2199.81 (474.78)	0.0050	-0.10
**Emotional Symptoms**	Male	F2 1st Derivative Maxin	2232.40 (477.75)	2174.87 (509.95)	0.0015	0.12
**Emotional Symptoms**	Male	F2 1st Derivative Range	4479.58 (852.90)	4374.68 (910.02)	0.0012	0.12
**Emotional Symptoms**	Male	F2 1st Derivative Mean	-5.94 (29.19)	-8.93 (33.17)	0.0100	0.10
**Emotional Symptoms**	Male	F2 1st Derivative Std	667.32 (107.35)	664.28 (110.96)	0.4418	0.03
**Emotional Symptoms**	Male	F2 1st Derivative Skewı	0.01 (0.32)	-0.01 (0.35)	0.0497	0.07
**Emotional Symptoms**	Male	F2 1st Derivative Kurtos	2.24 (1.41)	2.20 (1.44)	0.4108	0.03
**Emotional Symptoms**	Male	F3 1st Derivative Minim	-2016.44 (465.89)	-1959.41 (494.12)	0.0012	-0.12
**Emotional Symptoms**	Male	F3 1st Derivative Maxin	2020.61 (494.79)	1952.97 (534.21)	0.0003	0.13
**Emotional Symptoms**	Male	F3 1st Derivative Range	4037.05 (910.51)	3912.38 (973.67)	0.0003	0.13
**Emotional Symptoms**	Male	F3 1st Derivative Mean	-4.10 (29.31)	-7.35 (40.86)	0.0188	0.09
**Emotional Symptoms**	Male	F3 1st Derivative Std	691.64 (129.89)	677.08 (137.75)	0.0029	0.11
**Emotional Symptoms**	Male	F3 1st Derivative Skewı	-0.00 (0.30)	-0.02 (0.37)	0.1860	0.05
**Emotional Symptoms**	Male	F3 1st Derivative Kurtos	1.02 (0.89)	1.06 (0.97)	0.2982	-0.04
**Emotional Symptoms**	Male	F1 2nd Derivative Minin	-6570.54 (2022.63)	-6426.52 (2070.77)	0.0518	-0.07
**Emotional Symptoms**	Male	F1 2nd Derivative Maxi	4774.89 (1425.42)	4694.08 (1443.41)	0.1184	0.06
**Emotional Symptoms**	Male	F1 2nd Derivative Rang	11345.43 (3120.92)	11120.60 (3176.18)	0.0481	0.07
**Emotional Symptoms**	Male	F1 2nd Derivative Mear	0.73 (38.63)	-0.59 (44.26)	0.3933	0.03
**Emotional Symptoms**	Male	F1 2nd Derivative Std	1250.71 (352.03)	1265.53 (380.37)	0.2704	-0.04
**Emotional Symptoms**	Male	F1 2nd Derivative Skew	-0.78 (0.63)	-0.76 (0.65)	0.5476	-0.02
**Emotional Symptoms**	Male	F1 2nd Derivative Kurtc	8.68 (6.33)	8.47 (6.25)	0.3604	0.03
**Emotional Symptoms**	Male	F2 2nd Derivative Minin	-7596.95 (1947.88)	-7394.05 (2104.95)	0.0064	-0.10
**Emotional Symptoms**	Male	F2 2nd Derivative Maxi	6091.31 (1673.84)	5952.86 (1730.33)	0.0250	0.08
**Emotional Symptoms**	Male	F2 2nd Derivative Rang	13688.26 (3253.22)	13346.91 (3468.61)	0.0055	0.10
**Emotional Symptoms**	Male	F2 2nd Derivative Mear	3.06 (53.76)	0.64 (55.85)	0.2228	0.04
**Emotional Symptoms**	Male	F2 2nd Derivative Std	1720.38 (319.24)	1720.77 (350.60)	0.9749	0.00
**Emotional Symptoms**	Male	F2 2nd Derivative Skew	-0.54 (0.40)	-0.51 (0.44)	0.0588	-0.07
**Emotional Symptoms**	Male	F2 2nd Derivative Kurtc	4.19 (2.51)	4.05 (2.53)	0.1297	0.05
**Emotional Symptoms**	Male	F3 2nd Derivative Minin	-6506.77 (1869.53)	-6273.13 (2015.82)	0.0011	-0.12
**Emotional Symptoms**	Male	F3 2nd Derivative Maxi	6348.35 (1884.51)	6018.80 (2029.42)	0.0000	0.17
**Emotional Symptoms**	Male	F3 2nd Derivative Rang	12855.12 (3472.16)	12291.93 (3785.38)	0.0000	0.16
**Emotional Symptoms**	Male	F3 2nd Derivative Mear	5.31 (63.83)	1.36 (72.33)	0.1192	0.06
**Emotional Symptoms**	Male	F3 2nd Derivative Std	1792.51 (361.16)	1744.82 (388.73)	0.0005	0.13
**Emotional Symptoms**	Male	F3 2nd Derivative Skew	-0.15 (0.39)	-0.17 (0.42)	0.2939	0.04
**Emotional Symptoms**	Male	F3 2nd Derivative Kurtc	2.24 (1.41)	2.23 (1.48)	0.8518	0.01
**Conduct Problems**	Male	F0 Minimum	58.92 (11.84)	58.96 (13.04)	0.9209	0.00
**Conduct Problems**	Male	F0 Maximum	483.16 (3.11)	483.04 (5.73)	0.4048	0.03
**Conduct Problems**	Male	F0 Range	424.24 (13.25)	424.08 (16.10)	0.7259	0.01

**Conduct Problems**	Male	F0 Mean	281.74 (16.18)	281.55 (16.84)	0.7035	0.01
**Conduct Problems**	Male	F0 Std	113.05 (6.98)	112.94 (7.87)	0.6358	0.01
**Conduct Problems**	Male	F0 Skewness	0.13 (0.17)	0.13 (0.18)	0.9997	0.00
**Conduct Problems**	Male	F0 Kurtosis	-1.14 (0.23)	-1.13 (0.27)	0.1095	-0.05
**Conduct Problems**	Male	F0 de Minimum	-177.64 (17.65)	-177.45 (19.08)	0.7437	-0.01
**Conduct Problems**	Male	F0 de Maximum	187.14 (13.60)	187.03 (15.10)	0.8045	0.01
**Conduct Problems**	Male	F0 de Range	364.78 (26.83)	364.48 (29.52)	0.7346	0.01
**Conduct Problems**	Male	F0 de Mean	0.16 (0.68)	0.19 (0.65)	0.0983	-0.05
**Conduct Problems**	Male	F0 de Std	76.47 (13.04)	76.32 (13.45)	0.7108	0.01
**Conduct Problems**	Male	F0 de Skewness	0.32 (0.17)	0.32 (0.18)	0.5543	0.02
**Conduct Problems**	Male	F0 de Kurtosis	-0.46 (0.98)	-0.43 (1.04)	0.3552	-0.03
**Conduct Problems**	Male	F0 de2 Minimum	-651.39 (58.77)	-647.87 (62.87)	0.0603	-0.06
**Conduct Problems**	Male	F0 de2 Maximum	614.09 (77.16)	609.63 (79.07)	0.0627	0.06
**Conduct Problems**	Male	F0 de2 Range	1265.48 (125.28)	1257.50 (131.46)	0.0432	0.06
**Conduct Problems**	Male	F0 de2 Mean	0.07 (1.69)	0.11 (1.34)	0.4327	-0.02
**Conduct Problems**	Male	F0 de2 Std	298.74 (23.83)	298.62 (25.77)	0.8741	0.00
**Conduct Problems**	Male	F0 de2 Skewness	-0.06 (0.05)	-0.06 (0.05)	0.6666	0.01
**Conduct Problems**	Male	F0 de2 Kurtosis	-1.09 (0.26)	-1.08 (0.28)	0.4717	-0.02
**Conduct Problems**	Male	Energy Minimum	-11.63 (6.46)	-11.42 (6.13)	0.2777	-0.03
**Conduct Problems**	Male	Energy Maximum	3.79 (1.27)	3.92 (1.25)	0.0005	-0.11
**Conduct Problems**	Male	Energy Range	15.41 (6.93)	15.34 (6.50)	0.7191	0.01
**Conduct Problems**	Male	Energy Mean	-2.07 (1.05)	-2.05 (1.16)	0.5776	-0.02
**Conduct Problems**	Male	Energy Std	2.63 (0.61)	2.65 (0.59)	0.2973	-0.03
**Conduct Problems**	Male	Energy Skewness	-0.76 (0.64)	-0.72 (0.69)	0.0423	-0.06
**Conduct Problems**	Male	Energy Kurtosis	1.57 (4.96)	1.59 (5.39)	0.9054	0.00
**Conduct Problems**	Male	Energy de Minimum	-3.54 (1.92)	-3.41 (1.69)	0.0150	-0.07
**Conduct Problems**	Male	Energy de Maximum	4.55 (2.24)	4.49 (2.13)	0.3627	0.03
**Conduct Problems**	Male	Energy de Range	8.09 (3.89)	7.90 (3.50)	0.0843	0.05
**Conduct Problems**	Male	Energy de Mean	0.01 (0.03)	0.01 (0.03)	0.2142	-0.04
**Conduct Problems**	Male	Energy de Std	1.11 (0.22)	1.11 (0.22)	0.3842	0.03
**Conduct Problems**	Male	Energy de Skewness	0.38 (0.49)	0.43 (0.55)	0.0025	-0.09
**Conduct Problems**	Male	Energy de Kurtosis	1.76 (3.74)	1.80 (3.65)	0.7217	-0.01
**Conduct Problems**	Male	Energy de2 Minimum	-7.18 (4.13)	-6.91 (3.81)	0.0265	-0.07
**Conduct Problems**	Male	Energy de2 Maximum	8.57 (6.68)	8.24 (5.92)	0.0856	0.05
**Conduct Problems**	Male	Energy de2 Range	15.74 (10.50)	15.15 (9.45)	0.0489	0.06
**Conduct Problems**	Male	Energy de2 Mean	-0.01 (0.02)	-0.01 (0.02)	0.2506	0.04
**Conduct Problems**	Male	Energy de2 Std	1.56 (0.31)	1.54 (0.32)	0.0594	0.06
**Conduct Problems**	Male	Energy de2 Skewness	0.72 (0.76)	0.70 (0.66)	0.2477	0.04
**Conduct Problems**	Male	Energy de2 Kurtosis	6.89 (22.20)	5.82 (17.96)	0.0790	0.05
**Conduct Problems**	Male	MFCC 1 Minimum	-11.63 (6.46)	-11.42 (6.13)	0.2777	-0.03

**Conduct Problems**	Male	MFCC 1 Maximum	3.79 (1.27)	3.92 (1.25)	0.0005	-0.11
**Conduct Problems**	Male	MFCC 1 Range	15.41 (6.93)	15.34 (6.50)	0.7191	0.01
**Conduct Problems**	Male	MFCC 1 Mean	-2.07 (1.05)	-2.05 (1.16)	0.5776	-0.02
**Conduct Problems**	Male	MFCC 1 Std	2.63 (0.61)	2.65 (0.59)	0.2973	-0.03
**Conduct Problems**	Male	MFCC 1 Skewness	-0.76 (0.64)	-0.72 (0.69)	0.0423	-0.06
**Conduct Problems**	Male	MFCC 1 Kurtosis	1.57 (4.96)	1.59 (5.39)	0.9054	0.00
**Conduct Problems**	Male	MFCC 2 Minimum	-478.19 (101.21)	-474.00 (105.70)	0.1879	-0.04
**Conduct Problems**	Male	MFCC 2 Maximum	354.15 (60.58)	350.63 (62.37)	0.0624	0.06
**Conduct Problems**	Male	MFCC 2 Range	832.34 (149.62)	824.64 (156.27)	0.1012	0.05
**Conduct Problems**	Male	MFCC 2 Mean	41.71 (22.91)	40.72 (24.22)	0.1701	0.04
**Conduct Problems**	Male	MFCC 2 Std	156.60 (26.47)	155.25 (27.55)	0.1048	0.05
**Conduct Problems**	Male	MFCC 2 Skewness	-0.69 (0.26)	-0.68 (0.29)	0.4236	-0.02
**Conduct Problems**	Male	MFCC 2 Kurtosis	0.29 (0.47)	0.31 (0.50)	0.3821	-0.03
**Conduct Problems**	Male	MFCC 3 Minimum	-391.25 (111.34)	-389.11 (114.41)	0.5381	-0.02
**Conduct Problems**	Male	MFCC 3 Maximum	410.16 (74.64)	408.28 (77.28)	0.4201	0.02
**Conduct Problems**	Male	MFCC 3 Range	801.41 (170.51)	797.40 (175.47)	0.4497	0.02
**Conduct Problems**	Male	MFCC 3 Mean	29.86 (28.85)	29.92 (29.29)	0.9465	0.00
**Conduct Problems**	Male	MFCC 3 Std	147.74 (27.53)	146.81 (27.94)	0.2756	0.03
**Conduct Problems**	Male	MFCC 3 Skewness	-0.20 (0.23)	-0.20 (0.25)	0.8862	0.00
**Conduct Problems**	Male	MFCC 3 Kurtosis	-0.31 (0.35)	-0.30 (0.36)	0.1415	-0.05
**Conduct Problems**	Male	MFCC 4 Minimum	-461.92 (111.22)	-453.00 (117.99)	0.0114	-0.08
**Conduct Problems**	Male	MFCC 4 Maximum	547.06 (112.43)	542.40 (113.11)	0.1778	0.04
**Conduct Problems**	Male	MFCC 4 Range	1008.98 (202.06)	995.40 (210.22)	0.0320	0.07
**Conduct Problems**	Male	MFCC 4 Mean	20.89 (30.61)	22.01 (32.61)	0.2478	-0.04
**Conduct Problems**	Male	MFCC 4 Std	187.22 (31.49)	185.87 (32.99)	0.1729	0.04
**Conduct Problems**	Male	MFCC 4 Skewness	0.15 (0.25)	0.16 (0.26)	0.0295	-0.07
**Conduct Problems**	Male	MFCC 4 Kurtosis	-0.27 (0.35)	-0.27 (0.38)	0.9960	0.00
**Conduct Problems**	Male	MFCC 5 Minimum	-455.70 (129.25)	-448.70 (128.11)	0.0752	-0.05
**Conduct Problems**	Male	MFCC 5 Maximum	400.10 (91.55)	394.84 (91.93)	0.0614	0.06
**Conduct Problems**	Male	MFCC 5 Range	855.80 (196.52)	843.54 (197.30)	0.0421	0.06
**Conduct Problems**	Male	MFCC 5 Mean	-7.29 (30.97)	-6.54 (29.99)	0.4222	-0.02
**Conduct Problems**	Male	MFCC 5 Std	143.17 (27.10)	140.43 (26.58)	0.0008	0.10
**Conduct Problems**	Male	MFCC 5 Skewness	-0.14 (0.24)	-0.15 (0.25)	0.1113	0.05
**Conduct Problems**	Male	MFCC 5 Kurtosis	-0.01 (0.41)	0.01 (0.43)	0.0521	-0.06
**Conduct Problems**	Male	MFCC 6 Minimum	-450.50 (110.80)	-445.58 (115.31)	0.1571	-0.04
**Conduct Problems**	Male	MFCC 6 Maximum	407.32 (96.81)	400.45 (96.53)	0.0203	0.07
**Conduct Problems**	Male	MFCC 6 Range	857.82 (178.80)	846.03 (183.41)	0.0339	0.07
**Conduct Problems**	Male	MFCC 6 Mean	-14.06 (31.69)	-12.10 (32.21)	0.0461	-0.06
**Conduct Problems**	Male	MFCC 6 Std	141.59 (22.05)	140.45 (22.40)	0.0951	0.05
**Conduct Problems**	Male	MFCC 6 Skewness	-0.14 (0.24)	-0.15 (0.25)	0.0190	0.07

**Conduct Problems**	Male	MFCC 6 Kurtosis	0.02 (0.39)	0.03 (0.40)	0.9442	0.00
**Conduct Problems**	Male	MFCC 7 Minimum	-527.95 (123.11)	-514.29 (127.28)	0.0004	-0.11
**Conduct Problems**	Male	MFCC 7 Maximum	372.83 (92.73)	363.15 (92.85)	0.0007	0.10
**Conduct Problems**	Male	MFCC 7 Range	900.77 (185.31)	877.45 (192.03)	0.0001	0.12
**Conduct Problems**	Male	MFCC 7 Mean	-54.10 (34.09)	-52.50 (35.38)	0.1340	-0.05
**Conduct Problems**	Male	MFCC 7 Std	150.30 (23.81)	147.57 (24.60)	0.0002	0.11
**Conduct Problems**	Male	MFCC 7 Skewness	-0.20 (0.23)	-0.20 (0.23)	0.6527	0.01
**Conduct Problems**	Male	MFCC 7 Kurtosis	-0.02 (0.38)	-0.03 (0.38)	0.2467	0.04
**Conduct Problems**	Male	MFCC 8 Minimum	-516.22 (127.00)	-509.93 (129.22)	0.1098	-0.05
**Conduct Problems**	Male	MFCC 8 Maximum	359.71 (91.90)	352.01 (88.42)	0.0052	0.09
**Conduct Problems**	Male	MFCC 8 Range	875.92 (186.75)	861.95 (185.39)	0.0142	0.08
**Conduct Problems**	Male	MFCC 8 Mean	-61.00 (33.47)	-60.60 (35.46)	0.7062	-0.01
**Conduct Problems**	Male	MFCC 8 Std	143.47 (22.17)	142.59 (22.04)	0.1934	0.04
**Conduct Problems**	Male	MFCC 8 Skewness	-0.16 (0.26)	-0.16 (0.26)	0.8864	0.00
**Conduct Problems**	Male	MFCC 8 Kurtosis	0.06 (0.40)	0.04 (0.40)	0.0941	0.05
**Conduct Problems**	Male	MFCC 9 Minimum	-434.36 (98.93)	-427.58 (98.73)	0.0252	-0.07
**Conduct Problems**	Male	MFCC 9 Maximum	370.57 (90.73)	366.57 (91.64)	0.1529	0.04
**Conduct Problems**	Male	MFCC 9 Range	804.92 (159.38)	794.15 (161.36)	0.0285	0.07
**Conduct Problems**	Male	MFCC 9 Mean	-31.50 (28.72)	-30.29 (29.09)	0.1716	-0.04
**Conduct Problems**	Male	MFCC 9 Std	133.44 (18.71)	132.54 (19.25)	0.1237	0.05
**Conduct Problems**	Male	MFCC 9 Skewness	-0.02 (0.24)	-0.02 (0.24)	0.7361	-0.01
**Conduct Problems**	Male	MFCC 9 Kurtosis	-0.03 (0.38)	-0.04 (0.39)	0.4032	0.03
**Conduct Problems**	Male	MFCC 10 Minimum	-391.95 (90.73)	-385.58 (93.62)	0.0245	-0.07
**Conduct Problems**	Male	MFCC 10 Maximum	351.65 (92.53)	342.85 (93.60)	0.0020	0.09
**Conduct Problems**	Male	MFCC 10 Range	743.60 (158.59)	728.43 (162.25)	0.0021	0.09
**Conduct Problems**	Male	MFCC 10 Mean	-23.60 (24.86)	-23.78 (25.84)	0.8207	0.01
**Conduct Problems**	Male	MFCC 10 Std	121.25 (20.60)	119.54 (21.15)	0.0076	0.08
**Conduct Problems**	Male	MFCC 10 Skewness	0.01 (0.24)	-0.00 (0.24)	0.1495	0.04
**Conduct Problems**	Male	MFCC 10 Kurtosis	0.04 (0.38)	0.03 (0.41)	0.5070	0.02
**Conduct Problems**	Male	MFCC 11 Minimum	-353.31 (82.63)	-345.97 (83.77)	0.0041	-0.09
**Conduct Problems**	Male	MFCC 11 Maximum	295.92 (83.75)	288.50 (80.98)	0.0032	0.09
**Conduct Problems**	Male	MFCC 11 Range	649.22 (140.34)	634.48 (138.50)	0.0005	0.11
**Conduct Problems**	Male	MFCC 11 Mean	-24.38 (25.54)	-23.91 (25.68)	0.5533	-0.02
**Conduct Problems**	Male	MFCC 11 Std	102.51 (16.06)	100.91 (15.73)	0.0010	0.10
**Conduct Problems**	Male	MFCC 11 Skewness	-0.03 (0.25)	-0.03 (0.25)	0.9538	0.00
**Conduct Problems**	Male	MFCC 11 Kurtosis	0.15 (0.42)	0.13 (0.41)	0.1110	0.05
**Conduct Problems**	Male	MFCC 12 Minimum	-308.71 (73.99)	-303.70 (75.70)	0.0290	-0.07
**Conduct Problems**	Male	MFCC 12 Maximum	269.76 (69.15)	264.18 (67.80)	0.0078	0.08
**Conduct Problems**	Male	MFCC 12 Range	578.47 (119.32)	567.88 (121.13)	0.0041	0.09
**Conduct Problems**	Male	MFCC 12 Mean	-16.11 (21.49)	-15.77 (21.27)	0.6044	-0.02

**Conduct Problems**	Male	MFCC 12 Std	89.43 (12.98)	88.11 (12.97)	0.0009	0.10
**Conduct Problems**	Male	MFCC 12 Skewness	-0.05 (0.25)	-0.05 (0.25)	0.9471	0.00
**Conduct Problems**	Male	MFCC 12 Kurtosis	0.25 (0.42)	0.24 (0.44)	0.5139	0.02
**Conduct Problems**	Male	MFCC 13 Minimum	-254.49 (66.40)	-248.38 (65.81)	0.0025	-0.09
**Conduct Problems**	Male	MFCC 13 Maximum	257.32 (63.85)	253.45 (64.49)	0.0495	0.06
**Conduct Problems**	Male	MFCC 13 Range	511.81 (107.27)	501.83 (108.65)	0.0026	0.09
**Conduct Problems**	Male	MFCC 13 Mean	0.23 (18.33)	0.52 (18.73)	0.6098	-0.02
**Conduct Problems**	Male	MFCC 13 Std	77.70 (11.34)	76.78 (11.52)	0.0084	0.08
**Conduct Problems**	Male	MFCC 13 Skewness	0.03 (0.26)	0.04 (0.26)	0.5119	-0.02
**Conduct Problems**	Male	MFCC 13 Kurtosis	0.33 (0.45)	0.31 (0.45)	0.1113	0.05
**Conduct Problems**	Male	MELS 1 Minimum	-3.54 (1.92)	-3.41 (1.69)	0.0150	-0.07
**Conduct Problems**	Male	MELS 1 Maximum	4.55 (2.24)	4.49 (2.13)	0.3627	0.03
**Conduct Problems**	Male	MELS 1 Range	8.09 (3.89)	7.90 (3.50)	0.0843	0.05
**Conduct Problems**	Male	MELS 1 Mean	0.01 (0.03)	0.01 (0.03)	0.2142	-0.04
**Conduct Problems**	Male	MELS 1 Std	1.11 (0.22)	1.11 (0.22)	0.3842	0.03
**Conduct Problems**	Male	MELS 1 Skewness	0.38 (0.49)	0.43 (0.55)	0.0025	-0.09
**Conduct Problems**	Male	MELS 1 Kurtosis	1.76 (3.74)	1.80 (3.65)	0.7217	-0.01
**Conduct Problems**	Male	MELS 2 Minimum	-209.62 (46.44)	-207.30 (48.33)	0.1112	-0.05
**Conduct Problems**	Male	MELS 2 Maximum	194.21 (42.83)	193.07 (44.29)	0.3944	0.03
**Conduct Problems**	Male	MELS 2 Range	403.83 (84.76)	400.37 (88.14)	0.1929	0.04
**Conduct Problems**	Male	MELS 2 Mean	0.12 (0.85)	0.15 (0.87)	0.3618	-0.03
**Conduct Problems**	Male	MELS 2 Std	57.98 (11.48)	57.83 (11.89)	0.6886	0.01
**Conduct Problems**	Male	MELS 2 Skewness	-0.10 (0.24)	-0.09 (0.25)	0.0678	-0.06
**Conduct Problems**	Male	MELS 2 Kurtosis	1.12 (0.60)	1.14 (0.68)	0.4163	-0.03
**Conduct Problems**	Male	MELS 3 Minimum	-173.66 (41.95)	-173.20 (42.65)	0.7256	-0.01
**Conduct Problems**	Male	MELS 3 Maximum	165.69 (41.34)	164.09 (42.43)	0.2134	0.04
**Conduct Problems**	Male	MELS 3 Range	339.34 (77.56)	337.29 (79.63)	0.3943	0.03
**Conduct Problems**	Male	MELS 3 Mean	0.08 (0.92)	0.05 (0.69)	0.1194	0.05
**Conduct Problems**	Male	MELS 3 Std	49.78 (8.72)	49.63 (8.86)	0.5617	0.02
**Conduct Problems**	Male	MELS 3 Skewness	-0.12 (0.18)	-0.13 (0.19)	0.0805	0.05
**Conduct Problems**	Male	MELS 3 Kurtosis	0.45 (0.41)	0.45 (0.43)	0.5162	-0.02
**Conduct Problems**	Male	MELS 4 Minimum	-208.88 (46.34)	-207.05 (47.67)	0.2061	-0.04
**Conduct Problems**	Male	MELS 4 Maximum	204.74 (47.56)	201.28 (48.05)	0.0183	0.07
**Conduct Problems**	Male	MELS 4 Range	413.61 (84.02)	408.33 (87.17)	0.0446	0.06
**Conduct Problems**	Male	MELS 4 Mean	0.07 (1.05)	0.05 (0.94)	0.5996	0.02
**Conduct Problems**	Male	MELS 4 Std	60.21 (8.63)	59.86 (8.96)	0.1960	0.04
**Conduct Problems**	Male	MELS 4 Skewness	-0.07 (0.19)	-0.08 (0.19)	0.7328	0.01
**Conduct Problems**	Male	MELS 4 Kurtosis	0.43 (0.47)	0.42 (0.48)	0.6391	0.01
**Conduct Problems**	Male	MELS 5 Minimum	-182.11 (44.52)	-177.06 (43.30)	0.0002	-0.12
**Conduct Problems**	Male	MELS 5 Maximum	172.54 (40.05)	168.63 (38.93)	0.0012	0.10

**Conduct Problems**	Male	MELS 5 Range	354.65 (77.28)	345.69 (75.29)	0.0001	0.12
**Conduct Problems**	Male	MELS 5 Mean	0.07 (0.80)	0.05 (0.80)	0.3912	0.03
**Conduct Problems**	Male	MELS 5 Std	51.61 (7.73)	50.81 (7.65)	0.0006	0.10
**Conduct Problems**	Male	MELS 5 Skewness	-0.06 (0.17)	-0.06 (0.17)	0.7864	0.01
**Conduct Problems**	Male	MELS 5 Kurtosis	0.37 (0.42)	0.35 (0.40)	0.1270	0.05
**Conduct Problems**	Male	MELS 6 Minimum	-182.82 (40.59)	-179.11 (41.37)	0.0031	-0.09
**Conduct Problems**	Male	MELS 6 Maximum	179.66 (40.15)	178.52 (40.83)	0.3571	0.03
**Conduct Problems**	Male	MELS 6 Range	362.48 (72.60)	357.62 (75.02)	0.0321	0.07
**Conduct Problems**	Male	MELS 6 Mean	0.06 (0.82)	0.08 (0.78)	0.3816	-0.03
**Conduct Problems**	Male	MELS 6 Std	52.10 (6.58)	51.65 (6.81)	0.0289	0.07
**Conduct Problems**	Male	MELS 6 Skewness	-0.02 (0.19)	-0.01 (0.17)	0.0036	-0.09
**Conduct Problems**	Male	MELS 6 Kurtosis	0.43 (0.43)	0.42 (0.39)	0.2345	0.04
**Conduct Problems**	Male	MELS 7 Minimum	-200.93 (45.67)	-195.97 (46.13)	0.0004	-0.11
**Conduct Problems**	Male	MELS 7 Maximum	190.74 (43.34)	187.82 (44.48)	0.0306	0.07
**Conduct Problems**	Male	MELS 7 Range	391.67 (80.74)	383.80 (82.56)	0.0017	0.10
**Conduct Problems**	Male	MELS 7 Mean	0.03 (0.71)	0.03 (0.67)	0.8493	0.01
**Conduct Problems**	Male	MELS 7 Std	55.76 (7.28)	54.99 (7.47)	0.0008	0.10
**Conduct Problems**	Male	MELS 7 Skewness	-0.07 (0.18)	-0.07 (0.18)	0.6769	-0.01
**Conduct Problems**	Male	MELS 7 Kurtosis	0.50 (0.43)	0.47 (0.41)	0.0278	0.07
**Conduct Problems**	Male	MELS 8 Minimum	-192.85 (44.38)	-190.78 (46.47)	0.1389	-0.05
**Conduct Problems**	Male	MELS 8 Maximum	188.25 (43.60)	185.19 (44.33)	0.0234	0.07
**Conduct Problems**	Male	MELS 8 Range	381.10 (79.98)	375.97 (83.01)	0.0407	0.06
**Conduct Problems**	Male	MELS 8 Mean	-0.04 (0.85)	-0.03 (0.78)	0.7236	-0.01
**Conduct Problems**	Male	MELS 8 Std	53.75 (6.78)	53.40 (7.23)	0.1022	0.05
**Conduct Problems**	Male	MELS 8 Skewness	-0.04 (0.18)	-0.04 (0.17)	0.5802	-0.02
**Conduct Problems**	Male	MELS 8 Kurtosis	0.50 (0.43)	0.50 (0.42)	0.7696	0.01
**Conduct Problems**	Male	MELS 9 Minimum	-179.00 (39.89)	-175.23 (39.51)	0.0019	-0.09
**Conduct Problems**	Male	MELS 9 Maximum	177.86 (38.29)	174.87 (39.41)	0.0124	0.08
**Conduct Problems**	Male	MELS 9 Range	356.86 (70.00)	350.10 (71.42)	0.0019	0.10
**Conduct Problems**	Male	MELS 9 Mean	-0.00 (0.77)	-0.03 (0.70)	0.1551	0.04
**Conduct Problems**	Male	MELS 9 Std	50.88 (5.90)	50.43 (5.92)	0.0131	0.08
**Conduct Problems**	Male	MELS 9 Skewness	0.01 (0.17)	0.01 (0.17)	0.6077	-0.02
**Conduct Problems**	Male	MELS 9 Kurtosis	0.44 (0.40)	0.42 (0.41)	0.2074	0.04
**Conduct Problems**	Male	MELS 10 Minimum	-165.45 (37.81)	-162.70 (38.73)	0.0192	-0.07
**Conduct Problems**	Male	MELS 10 Maximum	162.27 (35.66)	158.42 (36.21)	0.0005	0.11
**Conduct Problems**	Male	MELS 10 Range	327.72 (65.98)	321.12 (67.84)	0.0013	0.10
**Conduct Problems**	Male	MELS 10 Mean	0.03 (0.95)	-0.00 (0.65)	0.2017	0.04
**Conduct Problems**	Male	MELS 10 Std	46.56 (5.30)	46.02 (5.69)	0.0014	0.10
**Conduct Problems**	Male	MELS 10 Skewness	-0.01 (0.16)	-0.02 (0.17)	0.0133	0.08
**Conduct Problems**	Male	MELS 10 Kurtosis	0.43 (0.39)	0.41 (0.39)	0.0862	0.05

**Conduct Problems**	Male	MELS 11 Minimum	-146.46 (32.97)	-144.04 (33.38)	0.0175	-0.07
**Conduct Problems**	Male	MELS 11 Maximum	144.08 (31.43)	141.19 (31.36)	0.0027	0.09
**Conduct Problems**	Male	MELS 11 Range	290.54 (57.80)	285.24 (58.29)	0.0029	0.09
**Conduct Problems**	Male	MELS 11 Mean	-0.03 (0.66)	0.02 (0.62)	0.0307	-0.07
**Conduct Problems**	Male	MELS 11 Std	41.58 (4.64)	41.08 (4.66)	0.0004	0.11
**Conduct Problems**	Male	MELS 11 Skewness	-0.01 (0.16)	-0.01 (0.16)	0.8567	-0.01
**Conduct Problems**	Male	MELS 11 Kurtosis	0.39 (0.37)	0.37 (0.39)	0.1519	0.04
**Conduct Problems**	Male	MELS 12 Minimum	-130.38 (27.20)	-128.20 (28.13)	0.0103	-0.08
**Conduct Problems**	Male	MELS 12 Maximum	131.70 (28.70)	128.51 (28.56)	0.0003	0.11
**Conduct Problems**	Male	MELS 12 Range	262.08 (49.83)	256.71 (51.05)	0.0005	0.11
**Conduct Problems**	Male	MELS 12 Mean	-0.02 (0.51)	-0.02 (0.53)	0.7395	-0.01
**Conduct Problems**	Male	MELS 12 Std	37.36 (4.04)	36.93 (4.11)	0.0006	0.11
**Conduct Problems**	Male	MELS 12 Skewness	-0.00 (0.17)	-0.00 (0.17)	0.8004	0.01
**Conduct Problems**	Male	MELS 12 Kurtosis	0.42 (0.42)	0.39 (0.40)	0.0207	0.07
**Conduct Problems**	Male	MELS 13 Minimum	-115.86 (24.24)	-114.09 (24.94)	0.0194	-0.07
**Conduct Problems**	Male	MELS 13 Maximum	116.57 (25.05)	115.45 (25.72)	0.1526	0.04
**Conduct Problems**	Male	MELS 13 Range	232.43 (43.93)	229.55 (45.63)	0.0364	0.06
**Conduct Problems**	Male	MELS 13 Mean	0.00 (0.50)	0.00 (0.48)	0.8978	0.00
**Conduct Problems**	Male	MELS 13 Std	33.25 (3.60)	32.98 (3.60)	0.0150	0.07
**Conduct Problems**	Male	MELS 13 Skewness	0.01 (0.16)	0.02 (0.15)	0.5963	-0.02
**Conduct Problems**	Male	MELS 13 Kurtosis	0.39 (0.36)	0.38 (0.36)	0.5240	0.02
**Conduct Problems**	Male	MELS 14 Minimum	-7.18 (4.13)	-6.91 (3.81)	0.0265	-0.07
**Conduct Problems**	Male	MELS 14 Maximum	8.57 (6.68)	8.24 (5.92)	0.0856	0.05
**Conduct Problems**	Male	MELS 14 Range	15.74 (10.50)	15.15 (9.45)	0.0489	0.06
**Conduct Problems**	Male	MELS 14 Mean	-0.01 (0.02)	-0.01 (0.02)	0.2506	0.04
**Conduct Problems**	Male	MELS 14 Std	1.56 (0.31)	1.54 (0.32)	0.0594	0.06
**Conduct Problems**	Male	MELS 14 Skewness	0.72 (0.76)	0.70 (0.66)	0.2477	0.04
**Conduct Problems**	Male	MELS 14 Kurtosis	6.89 (22.20)	5.82 (17.96)	0.0790	0.05
**Conduct Problems**	Male	MELS 15 Minimum	-227.58 (63.13)	-226.70 (65.18)	0.6537	-0.01
**Conduct Problems**	Male	MELS 15 Maximum	336.68 (90.87)	331.89 (92.35)	0.0884	0.05
**Conduct Problems**	Male	MELS 15 Range	564.26 (141.08)	558.59 (144.99)	0.1964	0.04
**Conduct Problems**	Male	MELS 15 Mean	0.00 (0.83)	0.01 (0.89)	0.6538	-0.01
**Conduct Problems**	Male	MELS 15 Std	63.32 (11.14)	63.04 (11.53)	0.4261	0.02
**Conduct Problems**	Male	MELS 15 Skewness	0.85 (0.35)	0.84 (0.34)	0.6446	0.01
**Conduct Problems**	Male	MELS 15 Kurtosis	3.35 (1.56)	3.31 (1.61)	0.3703	0.03
**Conduct Problems**	Male	MELS 16 Minimum	-251.59 (66.19)	-250.88 (69.99)	0.7363	-0.01
**Conduct Problems**	Male	MELS 16 Maximum	234.80 (61.83)	234.18 (63.55)	0.7468	0.01
**Conduct Problems**	Male	MELS 16 Range	486.39 (115.03)	485.06 (119.59)	0.7127	0.01
**Conduct Problems**	Male	MELS 16 Mean	-0.14 (0.99)	-0.14 (1.28)	0.9384	0.00
**Conduct Problems**	Male	MELS 16 Std	60.62 (7.69)	60.36 (8.13)	0.2813	0.03

**Conduct Problems**	Male	MELS 16 Skewness	-0.14 (0.21)	-0.14 (0.23)	0.9841	0.00
**Conduct Problems**	Male	MELS 16 Kurtosis	1.12 (0.85)	1.15 (0.95)	0.2014	-0.04
**Conduct Problems**	Male	MELS 17 Minimum	-291.57 (71.87)	-289.17 (74.74)	0.2867	-0.03
**Conduct Problems**	Male	MELS 17 Maximum	286.21 (70.68)	282.86 (75.70)	0.1379	0.05
**Conduct Problems**	Male	MELS 17 Range	577.78 (128.21)	572.03 (136.65)	0.1589	0.04
**Conduct Problems**	Male	MELS 17 Mean	-0.11 (1.32)	-0.16 (1.34)	0.1947	0.04
**Conduct Problems**	Male	MELS 17 Std	72.72 (8.25)	72.14 (8.97)	0.0278	0.07
**Conduct Problems**	Male	MELS 17 Skewness	-0.08 (0.18)	-0.08 (0.19)	0.1644	0.04
**Conduct Problems**	Male	MELS 17 Kurtosis	1.03 (0.69)	1.05 (0.76)	0.5899	-0.02
**Conduct Problems**	Male	MELS 18 Minimum	-269.00 (63.91)	-266.01 (63.61)	0.1259	-0.05
**Conduct Problems**	Male	MELS 18 Maximum	268.93 (65.93)	263.50 (63.21)	0.0059	0.08
**Conduct Problems**	Male	MELS 18 Range	537.93 (115.46)	529.51 (113.03)	0.0160	0.07
**Conduct Problems**	Male	MELS 18 Mean	0.00 (1.24)	-0.02 (1.38)	0.6700	0.01
**Conduct Problems**	Male	MELS 18 Std	71.28 (6.97)	70.65 (7.12)	0.0035	0.09
**Conduct Problems**	Male	MELS 18 Skewness	-0.03 (0.16)	-0.03 (0.17)	0.2758	0.03
**Conduct Problems**	Male	MELS 18 Kurtosis	0.69 (0.63)	0.67 (0.59)	0.3565	0.03
**Conduct Problems**	Male	MELS 19 Minimum	-282.51 (67.01)	-277.34 (70.00)	0.0140	-0.08
**Conduct Problems**	Male	MELS 19 Maximum	279.39 (62.35)	276.49 (64.54)	0.1367	0.05
**Conduct Problems**	Male	MELS 19 Range	561.91 (112.64)	553.83 (119.31)	0.0237	0.07
**Conduct Problems**	Male	MELS 19 Mean	-0.07 (1.61)	-0.05 (0.99)	0.5271	-0.02
**Conduct Problems**	Male	MELS 19 Std	75.21 (7.00)	74.40 (7.15)	0.0002	0.12
**Conduct Problems**	Male	MELS 19 Skewness	0.00 (0.15)	0.01 (0.17)	0.2964	-0.03
**Conduct Problems**	Male	MELS 19 Kurtosis	0.64 (0.55)	0.64 (0.61)	0.8009	-0.01
**Conduct Problems**	Male	MELS 20 Minimum	-301.15 (66.88)	-295.83 (68.76)	0.0107	-0.08
**Conduct Problems**	Male	MELS 20 Maximum	315.54 (75.47)	314.54 (77.31)	0.6706	0.01
**Conduct Problems**	Male	MELS 20 Range	616.68 (126.55)	610.37 (130.15)	0.1093	0.05
**Conduct Problems**	Male	MELS 20 Mean	0.00 (1.17)	0.01 (1.18)	0.8135	-0.01
**Conduct Problems**	Male	MELS 20 Std	81.66 (7.34)	81.01 (7.50)	0.0041	0.09
**Conduct Problems**	Male	MELS 20 Skewness	0.06 (0.15)	0.07 (0.16)	0.0540	-0.06
**Conduct Problems**	Male	MELS 20 Kurtosis	0.71 (0.62)	0.71 (0.60)	0.8876	0.00
**Conduct Problems**	Male	MELS 21 Minimum	-302.98 (67.70)	-299.30 (68.09)	0.0762	-0.05
**Conduct Problems**	Male	MELS 21 Maximum	308.90 (72.22)	303.53 (70.30)	0.0138	0.08
**Conduct Problems**	Male	MELS 21 Range	611.88 (124.69)	602.83 (123.78)	0.0173	0.07
**Conduct Problems**	Male	MELS 21 Mean	0.05 (1.42)	0.05 (1.28)	0.9866	0.00
**Conduct Problems**	Male	MELS 21 Std	82.00 (7.15)	81.53 (7.17)	0.0354	0.06
**Conduct Problems**	Male	MELS 21 Skewness	0.02 (0.14)	0.02 (0.14)	0.6812	0.01
**Conduct Problems**	Male	MELS 21 Kurtosis	0.62 (0.52)	0.62 (0.52)	0.6706	0.01
**Conduct Problems**	Male	MELS 22 Minimum	-292.93 (59.94)	-289.46 (63.33)	0.0674	-0.06
**Conduct Problems**	Male	MELS 22 Maximum	286.31 (57.81)	280.94 (58.73)	0.0027	0.09
**Conduct Problems**	Male	MELS 22 Range	579.24 (102.08)	570.41 (106.85)	0.0060	0.08

**Conduct Problems**	Male	MELS 22 Mean	0.07 (1.24)	0.02 (1.28)	0.2186	0.04
**Conduct Problems**	Male	MELS 22 Std	79.74 (6.18)	79.28 (6.31)	0.0158	0.07
**Conduct Problems**	Male	MELS 22 Skewness	-0.02 (0.14)	-0.02 (0.15)	0.2743	0.03
**Conduct Problems**	Male	MELS 22 Kurtosis	0.50 (0.43)	0.49 (0.44)	0.2821	0.03
**Conduct Problems**	Male	MELS 23 Minimum	-274.53 (53.41)	-271.48 (54.83)	0.0666	-0.06
**Conduct Problems**	Male	MELS 23 Maximum	274.53 (54.82)	270.07 (55.57)	0.0084	0.08
**Conduct Problems**	Male	MELS 23 Range	549.06 (94.17)	541.55 (97.03)	0.0105	0.08
**Conduct Problems**	Male	MELS 23 Mean	0.03 (1.34)	0.03 (1.31)	0.9830	0.00
**Conduct Problems**	Male	MELS 23 Std	76.77 (5.63)	76.30 (5.79)	0.0071	0.08
**Conduct Problems**	Male	MELS 23 Skewness	-0.01 (0.13)	-0.01 (0.13)	0.3895	0.03
**Conduct Problems**	Male	MELS 23 Kurtosis	0.44 (0.40)	0.43 (0.42)	0.3595	0.03
**Conduct Problems**	Male	MELS 24 Minimum	-256.33 (48.71)	-252.76 (50.40)	0.0190	-0.07
**Conduct Problems**	Male	MELS 24 Maximum	259.13 (49.88)	254.68 (50.50)	0.0039	0.09
**Conduct Problems**	Male	MELS 24 Range	515.46 (86.83)	507.44 (88.55)	0.0029	0.09
**Conduct Problems**	Male	MELS 24 Mean	0.02 (1.12)	0.05 (1.35)	0.3972	-0.03
**Conduct Problems**	Male	MELS 24 Std	72.54 (5.36)	72.15 (5.54)	0.0186	0.07
**Conduct Problems**	Male	MELS 24 Skewness	0.01 (0.13)	0.01 (0.13)	0.3368	-0.03
**Conduct Problems**	Male	MELS 24 Kurtosis	0.40 (0.38)	0.39 (0.41)	0.2254	0.04
**Conduct Problems**	Male	MELS 25 Minimum	-241.88 (46.63)	-238.83 (47.22)	0.0341	-0.06
**Conduct Problems**	Male	MELS 25 Maximum	239.71 (46.62)	235.47 (45.67)	0.0027	0.09
**Conduct Problems**	Male	MELS 25 Range	481.59 (81.30)	474.31 (81.42)	0.0035	0.09
**Conduct Problems**	Male	MELS 25 Mean	0.04 (1.10)	0.07 (1.01)	0.2992	-0.03
**Conduct Problems**	Male	MELS 25 Std	67.90 (5.14)	67.55 (5.01)	0.0256	0.07
**Conduct Problems**	Male	MELS 25 Skewness	-0.00 (0.12)	-0.01 (0.13)	0.1594	0.04
**Conduct Problems**	Male	MELS 25 Kurtosis	0.40 (0.42)	0.38 (0.41)	0.0391	0.06
**Conduct Problems**	Male	MELS 26 Minimum	-220.43 (41.44)	-218.33 (42.59)	0.1039	-0.05
**Conduct Problems**	Male	MELS 26 Maximum	219.40 (41.56)	218.26 (41.41)	0.3656	0.03
**Conduct Problems**	Male	MELS 26 Range	439.83 (72.03)	436.58 (74.00)	0.1475	0.04
**Conduct Problems**	Male	MELS 26 Mean	-0.01 (1.04)	0.04 (0.94)	0.1634	-0.04
**Conduct Problems**	Male	MELS 26 Std	62.68 (4.95)	62.58 (4.78)	0.5023	0.02
**Conduct Problems**	Male	MELS 26 Skewness	-0.01 (0.12)	-0.01 (0.12)	0.8979	0.00
**Conduct Problems**	Male	MELS 26 Kurtosis	0.37 (0.38)	0.36 (0.38)	0.4478	0.02
**Conduct Problems**	Male	F1 Minimum	215.75 (58.82)	222.46 (79.00)	0.0021	-0.10
**Conduct Problems**	Male	F1 Maximum	4551.91 (890.43)	4622.18 (923.50)	0.0117	-0.08
**Conduct Problems**	Male	F1 Range	4336.16 (896.94)	4399.72 (929.39)	0.0235	-0.07
**Conduct Problems**	Male	F1 Mean	1012.84 (416.84)	1094.29 (477.56)	0.0000	-0.18
**Conduct Problems**	Male	F1 Std	880.59 (245.69)	919.95 (260.90)	0.0000	-0.16
**Conduct Problems**	Male	F1 Skewness	2.03 (1.03)	1.90 (1.06)	0.0000	0.13
**Conduct Problems**	Male	F1 Kurtosis	5.08 (5.50)	4.56 (5.25)	0.0014	0.10
**Conduct Problems**	Male	F2 Minimum	582.66 (401.32)	636.25 (465.26)	0.0001	-0.12

**Conduct Problems**	Male	F2 Maximum	6289.57 (581.13)	6310.69 (559.67)	0.2254	-0.04
**Conduct Problems**	Male	F2 Range	5706.91 (779.75)	5674.45 (800.89)	0.1807	0.04
**Conduct Problems**	Male	F2 Mean	2509.52 (603.31)	2614.78 (683.02)	0.0000	-0.16
**Conduct Problems**	Male	F2 Std	1225.31 (204.73)	1255.72 (234.07)	0.0000	-0.14
**Conduct Problems**	Male	F2 Skewness	0.87 (0.58)	0.81 (0.64)	0.0017	0.10
**Conduct Problems**	Male	F2 Kurtosis	0.50 (1.25)	0.44 (1.35)	0.1034	0.05
**Conduct Problems**	Male	F3 Minimum	1867.49 (712.76)	1945.06 (783.59)	0.0008	-0.10
**Conduct Problems**	Male	F3 Maximum	6996.88 (400.94)	6963.38 (419.58)	0.0079	0.08
**Conduct Problems**	Male	F3 Range	5129.39 (966.55)	5018.31 (1043.23)	0.0003	0.11
**Conduct Problems**	Male	F3 Mean	4366.06 (486.87)	4399.18 (530.52)	0.0349	-0.07
**Conduct Problems**	Male	F3 Std	1195.11 (197.23)	1185.38 (223.39)	0.1354	0.05
**Conduct Problems**	Male	F3 Skewness	0.10 (0.57)	0.08 (0.62)	0.1755	0.04
**Conduct Problems**	Male	F3 Kurtosis	-0.53 (1.02)	-0.47 (1.14)	0.1097	-0.05
**Conduct Problems**	Male	F1 1st Derivative Minim	-1875.15 (466.48)	-1877.10 (468.96)	0.8914	0.00
**Conduct Problems**	Male	F1 1st Derivative Maxin	1885.90 (468.46)	1867.43 (490.58)	0.2101	0.04
**Conduct Problems**	Male	F1 1st Derivative Range	3761.05 (887.19)	3744.53 (900.79)	0.5467	0.02
**Conduct Problems**	Male	F1 1st Derivative Mean	-3.74 (22.60)	-6.23 (25.83)	0.0009	0.10
**Conduct Problems**	Male	F1 1st Derivative Std	487.81 (124.47)	497.89 (127.80)	0.0092	-0.08
**Conduct Problems**	Male	F1 1st Derivative Skewi	0.06 (0.43)	0.03 (0.49)	0.0324	0.07
**Conduct Problems**	Male	F1 1st Derivative Kurtos	5.75 (4.13)	5.50 (3.99)	0.0457	0.06
**Conduct Problems**	Male	F2 1st Derivative Minim	-2226.25 (456.01)	-2199.31 (478.73)	0.0609	-0.06
**Conduct Problems**	Male	F2 1st Derivative Maxin	2216.72 (490.78)	2167.13 (511.28)	0.0013	0.10
**Conduct Problems**	Male	F2 1st Derivative Range	4442.97 (873.95)	4366.44 (914.37)	0.0054	0.09
**Conduct Problems**	Male	F2 1st Derivative Mean	-6.10 (28.60)	-9.82 (34.71)	0.0002	0.12
**Conduct Problems**	Male	F2 1st Derivative Std	664.61 (107.98)	665.25 (111.71)	0.8499	-0.01
**Conduct Problems**	Male	F2 1st Derivative Skewi	0.00 (0.33)	-0.01 (0.36)	0.1786	0.04
**Conduct Problems**	Male	F2 1st Derivative Kurtos	2.23 (1.38)	2.20 (1.47)	0.4581	0.02
**Conduct Problems**	Male	F3 1st Derivative Minim	-1996.60 (469.24)	-1954.88 (501.18)	0.0052	-0.09
**Conduct Problems**	Male	F3 1st Derivative Maxin	2000.10 (504.74)	1945.39 (540.18)	0.0007	0.10
**Conduct Problems**	Male	F3 1st Derivative Range	3996.71 (920.67)	3900.27 (987.44)	0.0010	0.10
**Conduct Problems**	Male	F3 1st Derivative Mean	-4.45 (31.05)	-8.18 (43.14)	0.0016	0.10
**Conduct Problems**	Male	F3 1st Derivative Std	684.18 (129.62)	677.68 (140.63)	0.1188	0.05
**Conduct Problems**	Male	F3 1st Derivative Skewi	-0.00 (0.33)	-0.02 (0.38)	0.0781	0.05
**Conduct Problems**	Male	F3 1st Derivative Kurtos	1.05 (0.90)	1.05 (0.99)	0.8275	-0.01
**Conduct Problems**	Male	F1 2nd Derivative Minin	-6476.35 (2034.99)	-6447.40 (2079.24)	0.6464	-0.01
**Conduct Problems**	Male	F1 2nd Derivative Maxi	4729.98 (1433.13)	4699.97 (1444.36)	0.4961	0.02
**Conduct Problems**	Male	F1 2nd Derivative Rang	11206.33 (3130.80)	11147.37 (3189.56)	0.5429	0.02
**Conduct Problems**	Male	F1 2nd Derivative Mear	-0.38 (44.69)	-0.21 (41.78)	0.8985	0.00
**Conduct Problems**	Male	F1 2nd Derivative Std	1243.53 (354.82)	1275.75 (387.05)	0.0049	-0.09
**Conduct Problems**	Male	F1 2nd Derivative Skew	-0.77 (0.63)	-0.76 (0.65)	0.8020	-0.01

**Conduct Problems**	Male	F1 2nd Derivative Kurtc	8.70 (6.36)	8.39 (6.20)	0.1068	0.05
**Conduct Problems**	Male	F2 2nd Derivative Minin	-7536.21 (2022.31)	-7370.73 (2104.34)	0.0091	-0.08
**Conduct Problems**	Male	F2 2nd Derivative Maxi	6071.53 (1702.46)	5921.08 (1727.38)	0.0042	0.09
**Conduct Problems**	Male	F2 2nd Derivative Rang	13607.75 (3352.52)	13291.81 (3468.19)	0.0026	0.09
**Conduct Problems**	Male	F2 2nd Derivative Mear	0.80 (58.27)	1.49 (53.17)	0.6839	-0.01
**Conduct Problems**	Male	F2 2nd Derivative Std	1721.89 (338.97)	1719.79 (347.04)	0.8425	0.01
**Conduct Problems**	Male	F2 2nd Derivative Skew	-0.52 (0.42)	-0.51 (0.44)	0.5220	-0.02
**Conduct Problems**	Male	F2 2nd Derivative Kurtc	4.16 (2.50)	4.03 (2.54)	0.1087	0.05
**Conduct Problems**	Male	F3 2nd Derivative Minin	-6440.49 (1905.96)	-6243.60 (2037.93)	0.0012	-0.10
**Conduct Problems**	Male	F3 2nd Derivative Maxi	6240.95 (1915.54)	5987.35 (2056.19)	0.0000	0.13
**Conduct Problems**	Male	F3 2nd Derivative Rang	12681.43 (3554.95)	12230.95 (3830.73)	0.0001	0.12
**Conduct Problems**	Male	F3 2nd Derivative Mear	1.28 (64.89)	3.00 (74.32)	0.4244	-0.02
**Conduct Problems**	Male	F3 2nd Derivative Std	1773.05 (367.76)	1743.14 (393.48)	0.0108	0.08
**Conduct Problems**	Male	F3 2nd Derivative Skew	-0.16 (0.39)	-0.17 (0.43)	0.3955	0.03
**Conduct Problems**	Male	F3 2nd Derivative Kurtc	2.24 (1.37)	2.23 (1.52)	0.8758	0.00
**Peer Problems**	Male	F0 Minimum	58.80 (11.75)	59.17 (13.69)	0.3494	-0.03
**Peer Problems**	Male	F0 Maximum	483.13 (3.06)	483.03 (6.65)	0.4881	0.02
**Peer Problems**	Male	F0 Range	424.33 (13.05)	423.86 (17.50)	0.3137	0.03
**Peer Problems**	Male	F0 Mean	281.58 (16.65)	281.72 (16.42)	0.7815	-0.01
**Peer Problems**	Male	F0 Std	112.91 (7.21)	113.11 (7.95)	0.3794	-0.03
**Peer Problems**	Male	F0 Skewness	0.13 (0.17)	0.12 (0.18)	0.3237	0.03
**Peer Problems**	Male	F0 Kurtosis	-1.14 (0.25)	-1.14 (0.26)	0.9401	0.00
**Peer Problems**	Male	F0 de Minimum	-177.27 (18.20)	-177.93 (18.93)	0.2533	0.04
**Peer Problems**	Male	F0 de Maximum	187.16 (14.44)	186.96 (14.56)	0.6584	0.01
**Peer Problems**	Male	F0 de Range	364.43 (27.94)	364.89 (29.13)	0.6044	-0.02
**Peer Problems**	Male	F0 de Mean	0.18 (0.68)	0.17 (0.63)	0.5740	0.02
**Peer Problems**	Male	F0 de Std	76.25 (13.05)	76.60 (13.63)	0.3951	-0.03
**Peer Problems**	Male	F0 de Skewness	0.32 (0.17)	0.32 (0.19)	0.9434	0.00
**Peer Problems**	Male	F0 de Kurtosis	-0.45 (0.98)	-0.43 (1.07)	0.6964	-0.01
**Peer Problems**	Male	F0 de2 Minimum	-649.42 (59.49)	-649.27 (63.74)	0.9363	0.00
**Peer Problems**	Male	F0 de2 Maximum	611.56 (77.13)	611.44 (80.08)	0.9603	0.00
**Peer Problems**	Male	F0 de2 Range	1260.98 (125.85)	1260.71 (133.58)	0.9456	0.00
**Peer Problems**	Male	F0 de2 Mean	0.06 (1.37)	0.14 (1.68)	0.0881	-0.05
**Peer Problems**	Male	F0 de2 Std	298.65 (24.72)	298.70 (25.35)	0.9431	0.00
**Peer Problems**	Male	F0 de2 Skewness	-0.06 (0.05)	-0.06 (0.05)	0.5053	0.02
**Peer Problems**	Male	F0 de2 Kurtosis	-1.09 (0.27)	-1.08 (0.28)	0.4691	-0.02
**Peer Problems**	Male	Energy Minimum	-11.50 (6.30)	-11.51 (6.23)	0.9618	0.00
**Peer Problems**	Male	Energy Maximum	3.85 (1.27)	3.88 (1.24)	0.5250	-0.02
**Peer Problems**	Male	Energy Range	15.36 (6.74)	15.39 (6.61)	0.8693	-0.01
**Peer Problems**	Male	Energy Mean	-2.06 (1.11)	-2.06 (1.13)	0.9902	0.00

**Peer Problems**	Male	Energy Std	2.64 (0.61)	2.65 (0.59)	0.2995	-0.03
**Peer Problems**	Male	Energy Skewness	-0.74 (0.66)	-0.72 (0.69)	0.3577	-0.03
**Peer Problems**	Male	Energy Kurtosis	1.58 (5.10)	1.58 (5.38)	0.9868	0.00
**Peer Problems**	Male	Energy de Minimum	-3.46 (1.79)	-3.47 (1.80)	0.7729	0.01
**Peer Problems**	Male	Energy de Maximum	4.53 (2.18)	4.51 (2.17)	0.7978	0.01
**Peer Problems**	Male	Energy de Range	7.98 (3.66)	7.98 (3.69)	0.9912	0.00
**Peer Problems**	Male	Energy de Mean	0.01 (0.03)	0.01 (0.03)	0.7327	0.01
**Peer Problems**	Male	Energy de Std	1.11 (0.22)	1.11 (0.23)	0.2770	-0.03
**Peer Problems**	Male	Energy de Skewness	0.41 (0.53)	0.41 (0.51)	0.7320	0.01
**Peer Problems**	Male	Energy de Kurtosis	1.80 (3.74)	1.75 (3.60)	0.7135	0.01
**Peer Problems**	Male	Energy de2 Minimum	-7.02 (3.93)	-7.02 (3.99)	0.9538	0.00
**Peer Problems**	Male	Energy de2 Maximum	8.36 (6.16)	8.41 (6.40)	0.7979	-0.01
**Peer Problems**	Male	Energy de2 Range	15.38 (9.78)	15.43 (10.12)	0.8899	0.00
**Peer Problems**	Male	Energy de2 Mean	-0.01 (0.02)	-0.01 (0.02)	0.6497	-0.01
**Peer Problems**	Male	Energy de2 Std	1.55 (0.31)	1.55 (0.33)	0.6101	-0.02
**Peer Problems**	Male	Energy de2 Skewness	0.70 (0.70)	0.72 (0.71)	0.4590	-0.02
**Peer Problems**	Male	Energy de2 Kurtosis	6.27 (19.95)	6.28 (19.75)	0.9809	0.00
**Peer Problems**	Male	MFCC 1 Minimum	-11.50 (6.30)	-11.51 (6.23)	0.9618	0.00
**Peer Problems**	Male	MFCC 1 Maximum	3.85 (1.27)	3.88 (1.24)	0.5250	-0.02
**Peer Problems**	Male	MFCC 1 Range	15.36 (6.74)	15.39 (6.61)	0.8693	-0.01
**Peer Problems**	Male	MFCC 1 Mean	-2.06 (1.11)	-2.06 (1.13)	0.9902	0.00
**Peer Problems**	Male	MFCC 1 Std	2.64 (0.61)	2.65 (0.59)	0.2995	-0.03
**Peer Problems**	Male	MFCC 1 Skewness	-0.74 (0.66)	-0.72 (0.69)	0.3577	-0.03
**Peer Problems**	Male	MFCC 1 Kurtosis	1.58 (5.10)	1.58 (5.38)	0.9868	0.00
**Peer Problems**	Male	MFCC 2 Minimum	-476.28 (102.57)	-475.00 (105.79)	0.6896	-0.01
**Peer Problems**	Male	MFCC 2 Maximum	352.03 (62.11)	352.25 (60.92)	0.9086	0.00
**Peer Problems**	Male	MFCC 2 Range	828.31 (152.27)	827.25 (155.47)	0.8227	0.01
**Peer Problems**	Male	MFCC 2 Mean	40.91 (24.47)	41.49 (22.40)	0.4254	-0.02
**Peer Problems**	Male	MFCC 2 Std	155.84 (26.92)	155.79 (27.40)	0.9524	0.00
**Peer Problems**	Male	MFCC 2 Skewness	-0.69 (0.28)	-0.68 (0.28)	0.7053	-0.01
**Peer Problems**	Male	MFCC 2 Kurtosis	0.31 (0.49)	0.29 (0.49)	0.4515	0.02
**Peer Problems**	Male	MFCC 3 Minimum	-391.51 (113.54)	-387.72 (112.45)	0.2792	-0.03
**Peer Problems**	Male	MFCC 3 Maximum	409.98 (75.76)	407.68 (76.82)	0.3287	0.03
**Peer Problems**	Male	MFCC 3 Range	801.49 (173.50)	795.39 (173.18)	0.2563	0.04
**Peer Problems**	Male	MFCC 3 Mean	30.14 (28.93)	29.53 (29.36)	0.4969	0.02
**Peer Problems**	Male	MFCC 3 Std	147.49 (27.67)	146.76 (27.92)	0.3965	0.03
**Peer Problems**	Male	MFCC 3 Skewness	-0.20 (0.24)	-0.20 (0.24)	0.4734	-0.02
**Peer Problems**	Male	MFCC 3 Kurtosis	-0.30 (0.37)	-0.31 (0.33)	0.1657	0.04
**Peer Problems**	Male	MFCC 4 Minimum	-458.00 (115.91)	-454.87 (114.21)	0.3806	-0.03
**Peer Problems**	Male	MFCC 4 Maximum	543.52 (112.11)	545.68 (113.96)	0.5360	-0.02

**Peer Problems**	Male	MFCC 4 Range	1001.52 (206.86)	1000.55 (207.01)	0.8801	0.00
**Peer Problems**	Male	MFCC 4 Mean	21.52 (31.98)	21.56 (31.48)	0.9716	0.00
**Peer Problems**	Male	MFCC 4 Std	186.79 (32.28)	185.89 (32.51)	0.3644	0.03
**Peer Problems**	Male	MFCC 4 Skewness	0.15 (0.26)	0.16 (0.25)	0.0994	-0.05
**Peer Problems**	Male	MFCC 4 Kurtosis	-0.28 (0.36)	-0.26 (0.36)	0.1182	-0.05
**Peer Problems**	Male	MFCC 5 Minimum	-451.99 (128.57)	-451.15 (128.73)	0.8329	-0.01
**Peer Problems**	Male	MFCC 5 Maximum	396.98 (91.30)	397.20 (92.58)	0.9375	0.00
**Peer Problems**	Male	MFCC 5 Range	848.97 (196.21)	848.35 (198.38)	0.9194	0.00
**Peer Problems**	Male	MFCC 5 Mean	-7.15 (30.53)	-6.40 (30.22)	0.4234	-0.02
**Peer Problems**	Male	MFCC 5 Std	141.83 (26.62)	141.20 (27.16)	0.4485	0.02
**Peer Problems**	Male	MFCC 5 Skewness	-0.15 (0.26)	-0.15 (0.23)	0.9498	0.00
**Peer Problems**	Male	MFCC 5 Kurtosis	0.01 (0.43)	-0.00 (0.41)	0.3259	0.03
**Peer Problems**	Male	MFCC 6 Minimum	-448.59 (113.18)	-446.22 (113.85)	0.4992	-0.02
**Peer Problems**	Male	MFCC 6 Maximum	403.72 (97.18)	402.79 (95.97)	0.7575	0.01
**Peer Problems**	Male	MFCC 6 Range	852.31 (181.91)	849.01 (181.02)	0.5574	0.02
**Peer Problems**	Male	MFCC 6 Mean	-13.17 (31.82)	-12.56 (32.29)	0.5393	-0.02
**Peer Problems**	Male	MFCC 6 Std	141.22 (22.35)	140.49 (22.12)	0.2884	0.03
**Peer Problems**	Male	MFCC 6 Skewness	-0.14 (0.24)	-0.15 (0.26)	0.4492	0.02
**Peer Problems**	Male	MFCC 6 Kurtosis	0.02 (0.40)	0.03 (0.39)	0.5246	-0.02
**Peer Problems**	Male	MFCC 7 Minimum	-521.69 (125.93)	-517.56 (125.33)	0.2891	-0.03
**Peer Problems**	Male	MFCC 7 Maximum	366.85 (93.55)	367.85 (91.93)	0.7303	-0.01
**Peer Problems**	Male	MFCC 7 Range	888.54 (189.86)	885.41 (189.10)	0.5933	0.02
**Peer Problems**	Male	MFCC 7 Mean	-53.27 (35.03)	-53.05 (34.57)	0.8375	-0.01
**Peer Problems**	Male	MFCC 7 Std	148.70 (24.51)	148.75 (23.99)	0.9532	0.00
**Peer Problems**	Male	MFCC 7 Skewness	-0.20 (0.24)	-0.19 (0.22)	0.5203	-0.02
**Peer Problems**	Male	MFCC 7 Kurtosis	-0.01 (0.38)	-0.04 (0.39)	0.0171	0.07
**Peer Problems**	Male	MFCC 8 Minimum	-512.67 (128.33)	-512.48 (128.32)	0.9619	0.00
**Peer Problems**	Male	MFCC 8 Maximum	355.89 (91.68)	354.30 (87.30)	0.5699	0.02
**Peer Problems**	Male	MFCC 8 Range	868.55 (187.96)	866.78 (183.17)	0.7583	0.01
**Peer Problems**	Male	MFCC 8 Mean	-60.54 (33.88)	-61.14 (35.76)	0.5733	0.02
**Peer Problems**	Male	MFCC 8 Std	142.73 (22.16)	143.32 (22.02)	0.3899	-0.03
**Peer Problems**	Male	MFCC 8 Skewness	-0.16 (0.26)	-0.16 (0.25)	0.9963	0.00
**Peer Problems**	Male	MFCC 8 Kurtosis	0.06 (0.40)	0.03 (0.40)	0.0466	0.06
**Peer Problems**	Male	MFCC 9 Minimum	-429.26 (99.80)	-432.28 (97.40)	0.3225	0.03
**Peer Problems**	Male	MFCC 9 Maximum	370.66 (92.69)	364.56 (88.93)	0.0307	0.07
**Peer Problems**	Male	MFCC 9 Range	799.92 (163.86)	796.84 (155.46)	0.5363	0.02
**Peer Problems**	Male	MFCC 9 Mean	-29.99 (28.61)	-32.05 (29.41)	0.0212	0.07
**Peer Problems**	Male	MFCC 9 Std	132.84 (19.47)	133.05 (18.33)	0.7139	-0.01
**Peer Problems**	Male	MFCC 9 Skewness	-0.01 (0.23)	-0.03 (0.25)	0.0682	0.06
**Peer Problems**	Male	MFCC 9 Kurtosis	-0.03 (0.38)	-0.03 (0.40)	0.8294	0.01

**Peer Problems**	Male	MFCC 10 Minimum	-388.46 (92.16)	-387.99 (92.92)	0.8693	-0.01
**Peer Problems**	Male	MFCC 10 Maximum	346.77 (94.69)	346.26 (90.97)	0.8596	0.01
**Peer Problems**	Male	MFCC 10 Range	735.23 (162.21)	734.25 (158.82)	0.8437	0.01
**Peer Problems**	Male	MFCC 10 Mean	-24.34 (25.37)	-22.73 (25.49)	0.0405	-0.06
**Peer Problems**	Male	MFCC 10 Std	120.14 (21.01)	120.46 (20.82)	0.6170	-0.02
**Peer Problems**	Male	MFCC 10 Skewness	0.01 (0.24)	-0.00 (0.24)	0.2097	0.04
**Peer Problems**	Male	MFCC 10 Kurtosis	0.03 (0.39)	0.03 (0.40)	0.5237	0.02
**Peer Problems**	Male	MFCC 11 Minimum	-350.28 (83.96)	-347.22 (82.41)	0.2372	-0.04
**Peer Problems**	Male	MFCC 11 Maximum	291.35 (82.27)	292.08 (82.21)	0.7754	-0.01
**Peer Problems**	Male	MFCC 11 Range	641.63 (140.19)	639.30 (138.34)	0.5904	0.02
**Peer Problems**	Male	MFCC 11 Mean	-24.32 (25.38)	-23.78 (25.98)	0.5013	-0.02
**Peer Problems**	Male	MFCC 11 Std	101.58 (15.94)	101.60 (15.81)	0.9712	0.00
**Peer Problems**	Male	MFCC 11 Skewness	-0.03 (0.25)	-0.03 (0.24)	0.5793	-0.02
**Peer Problems**	Male	MFCC 11 Kurtosis	0.15 (0.42)	0.13 (0.40)	0.1557	0.04
**Peer Problems**	Male	MFCC 12 Minimum	-305.41 (75.01)	-306.44 (75.03)	0.6576	0.01
**Peer Problems**	Male	MFCC 12 Maximum	267.53 (68.72)	265.00 (67.95)	0.2331	0.04
**Peer Problems**	Male	MFCC 12 Range	572.94 (120.99)	571.45 (119.69)	0.6882	0.01
**Peer Problems**	Male	MFCC 12 Mean	-15.46 (21.48)	-16.62 (21.15)	0.0809	0.05
**Peer Problems**	Male	MFCC 12 Std	88.80 (13.11)	88.47 (12.80)	0.4136	0.03
**Peer Problems**	Male	MFCC 12 Skewness	-0.05 (0.25)	-0.06 (0.26)	0.3596	0.03
**Peer Problems**	Male	MFCC 12 Kurtosis	0.23 (0.43)	0.25 (0.44)	0.2227	-0.04
**Peer Problems**	Male	MFCC 13 Minimum	-251.28 (65.55)	-250.48 (67.01)	0.6976	-0.01
**Peer Problems**	Male	MFCC 13 Maximum	255.17 (63.72)	254.96 (65.06)	0.9151	0.00
**Peer Problems**	Male	MFCC 13 Range	506.45 (107.34)	505.44 (109.46)	0.7635	0.01
**Peer Problems**	Male	MFCC 13 Mean	0.29 (18.35)	0.57 (18.89)	0.6228	-0.02
**Peer Problems**	Male	MFCC 13 Std	77.20 (11.37)	77.13 (11.58)	0.8436	0.01
**Peer Problems**	Male	MFCC 13 Skewness	0.03 (0.26)	0.04 (0.26)	0.6606	-0.01
**Peer Problems**	Male	MFCC 13 Kurtosis	0.33 (0.46)	0.31 (0.44)	0.2654	0.03
**Peer Problems**	Male	MELS 1 Minimum	-3.46 (1.79)	-3.47 (1.80)	0.7729	0.01
**Peer Problems**	Male	MELS 1 Maximum	4.53 (2.18)	4.51 (2.17)	0.7978	0.01
**Peer Problems**	Male	MELS 1 Range	7.98 (3.66)	7.98 (3.69)	0.9912	0.00
**Peer Problems**	Male	MELS 1 Mean	0.01 (0.03)	0.01 (0.03)	0.7327	0.01
**Peer Problems**	Male	MELS 1 Std	1.11 (0.22)	1.11 (0.23)	0.2770	-0.03
**Peer Problems**	Male	MELS 1 Skewness	0.41 (0.53)	0.41 (0.51)	0.7320	0.01
**Peer Problems**	Male	MELS 1 Kurtosis	1.80 (3.74)	1.75 (3.60)	0.7135	0.01
**Peer Problems**	Male	MELS 2 Minimum	-208.04 (47.39)	-208.64 (47.81)	0.6844	0.01
**Peer Problems**	Male	MELS 2 Maximum	192.75 (43.56)	194.79 (43.82)	0.1310	-0.05
**Peer Problems**	Male	MELS 2 Range	400.79 (86.59)	403.43 (86.96)	0.3255	-0.03
**Peer Problems**	Male	MELS 2 Mean	0.13 (0.88)	0.14 (0.82)	0.7400	-0.01
**Peer Problems**	Male	MELS 2 Std	57.70 (11.64)	58.19 (11.84)	0.1760	-0.04

**Peer Problems**	Male	MELS 2 Skewness	-0.10 (0.25)	-0.09 (0.24)	0.0242	-0.07
**Peer Problems**	Male	MELS 2 Kurtosis	1.14 (0.64)	1.12 (0.65)	0.4317	0.02
**Peer Problems**	Male	MELS 3 Minimum	-174.05 (42.41)	-172.38 (42.26)	0.2043	-0.04
**Peer Problems**	Male	MELS 3 Maximum	165.21 (41.78)	164.08 (42.27)	0.3868	0.03
**Peer Problems**	Male	MELS 3 Range	339.25 (78.57)	336.46 (79.03)	0.2527	0.04
**Peer Problems**	Male	MELS 3 Mean	0.05 (0.74)	0.08 (0.87)	0.1914	-0.04
**Peer Problems**	Male	MELS 3 Std	49.74 (8.76)	49.62 (8.86)	0.6443	0.01
**Peer Problems**	Male	MELS 3 Skewness	-0.13 (0.19)	-0.12 (0.18)	0.7431	-0.01
**Peer Problems**	Male	MELS 3 Kurtosis	0.47 (0.44)	0.42 (0.39)	0.0006	0.11
**Peer Problems**	Male	MELS 4 Minimum	-208.16 (47.18)	-207.30 (47.02)	0.5535	-0.02
**Peer Problems**	Male	MELS 4 Maximum	202.68 (47.84)	202.83 (47.93)	0.9210	0.00
**Peer Problems**	Male	MELS 4 Range	410.85 (85.60)	410.13 (86.34)	0.7873	0.01
**Peer Problems**	Male	MELS 4 Mean	0.05 (1.03)	0.08 (0.92)	0.3161	-0.03
**Peer Problems**	Male	MELS 4 Std	59.98 (8.84)	60.06 (8.80)	0.7623	-0.01
**Peer Problems**	Male	MELS 4 Skewness	-0.08 (0.19)	-0.07 (0.18)	0.7119	-0.01
**Peer Problems**	Male	MELS 4 Kurtosis	0.43 (0.49)	0.41 (0.46)	0.1959	0.04
**Peer Problems**	Male	MELS 5 Minimum	-179.67 (44.44)	-178.46 (43.03)	0.3754	-0.03
**Peer Problems**	Male	MELS 5 Maximum	170.37 (39.10)	170.15 (40.01)	0.8600	0.01
**Peer Problems**	Male	MELS 5 Range	350.04 (76.17)	348.62 (76.41)	0.5476	0.02
**Peer Problems**	Male	MELS 5 Mean	0.05 (0.80)	0.07 (0.79)	0.4285	-0.02
**Peer Problems**	Male	MELS 5 Std	51.23 (7.62)	51.02 (7.80)	0.3648	0.03
**Peer Problems**	Male	MELS 5 Skewness	-0.06 (0.17)	-0.06 (0.16)	0.7628	-0.01
**Peer Problems**	Male	MELS 5 Kurtosis	0.36 (0.39)	0.35 (0.43)	0.1355	0.05
**Peer Problems**	Male	MELS 6 Minimum	-180.91 (41.16)	-180.32 (40.97)	0.6398	-0.01
**Peer Problems**	Male	MELS 6 Maximum	178.96 (40.58)	179.06 (40.50)	0.9375	0.00
**Peer Problems**	Male	MELS 6 Range	359.87 (74.17)	359.38 (73.86)	0.8284	0.01
**Peer Problems**	Male	MELS 6 Mean	0.07 (0.74)	0.07 (0.88)	0.9021	0.00
**Peer Problems**	Male	MELS 6 Std	51.90 (6.72)	51.74 (6.70)	0.4462	0.02
**Peer Problems**	Male	MELS 6 Skewness	-0.01 (0.17)	-0.01 (0.18)	0.5703	-0.02
**Peer Problems**	Male	MELS 6 Kurtosis	0.42 (0.39)	0.43 (0.43)	0.2791	-0.03
**Peer Problems**	Male	MELS 7 Minimum	-198.18 (46.22)	-197.90 (45.67)	0.8463	-0.01
**Peer Problems**	Male	MELS 7 Maximum	189.28 (44.43)	188.72 (43.39)	0.6810	0.01
**Peer Problems**	Male	MELS 7 Range	387.45 (82.23)	386.62 (81.35)	0.7415	0.01
**Peer Problems**	Male	MELS 7 Mean	0.03 (0.66)	0.03 (0.74)	0.9572	0.00
**Peer Problems**	Male	MELS 7 Std	55.30 (7.43)	55.34 (7.34)	0.8490	-0.01
**Peer Problems**	Male	MELS 7 Skewness	-0.07 (0.18)	-0.07 (0.18)	0.7262	0.01
**Peer Problems**	Male	MELS 7 Kurtosis	0.49 (0.42)	0.48 (0.42)	0.7343	0.01
**Peer Problems**	Male	MELS 8 Minimum	-191.97 (45.67)	-191.17 (45.52)	0.5722	-0.02
**Peer Problems**	Male	MELS 8 Maximum	186.48 (44.20)	186.49 (43.82)	0.9981	0.00
**Peer Problems**	Male	MELS 8 Range	378.45 (82.15)	377.66 (81.21)	0.7537	0.01

**Peer Problems**	Male	MELS 8 Mean	-0.05 (0.91)	-0.01 (0.63)	0.1348	-0.05
**Peer Problems**	Male	MELS 8 Std	53.46 (7.05)	53.68 (7.03)	0.3161	-0.03
**Peer Problems**	Male	MELS 8 Skewness	-0.04 (0.17)	-0.04 (0.18)	0.7674	0.01
**Peer Problems**	Male	MELS 8 Kurtosis	0.51 (0.42)	0.49 (0.44)	0.1410	0.05
**Peer Problems**	Male	MELS 9 Minimum	-176.65 (39.81)	-177.10 (39.57)	0.7182	0.01
**Peer Problems**	Male	MELS 9 Maximum	175.86 (39.02)	176.56 (38.88)	0.5635	-0.02
**Peer Problems**	Male	MELS 9 Range	352.51 (71.10)	353.65 (70.59)	0.6033	-0.02
**Peer Problems**	Male	MELS 9 Mean	-0.03 (0.69)	-0.01 (0.79)	0.3207	-0.03
**Peer Problems**	Male	MELS 9 Std	50.52 (5.98)	50.76 (5.80)	0.1881	-0.04
**Peer Problems**	Male	MELS 9 Skewness	0.01 (0.16)	0.01 (0.17)	0.8596	0.01
**Peer Problems**	Male	MELS 9 Kurtosis	0.43 (0.41)	0.42 (0.41)	0.6038	0.02
**Peer Problems**	Male	MELS 10 Minimum	-164.00 (38.47)	-163.65 (38.21)	0.7639	-0.01
**Peer Problems**	Male	MELS 10 Maximum	160.29 (36.27)	159.67 (35.64)	0.5763	0.02
**Peer Problems**	Male	MELS 10 Range	324.30 (67.21)	323.32 (67.02)	0.6373	0.01
**Peer Problems**	Male	MELS 10 Mean	0.02 (0.82)	0.00 (0.73)	0.4981	0.02
**Peer Problems**	Male	MELS 10 Std	46.24 (5.54)	46.27 (5.53)	0.8427	-0.01
**Peer Problems**	Male	MELS 10 Skewness	-0.02 (0.16)	-0.01 (0.16)	0.4764	-0.02
**Peer Problems**	Male	MELS 10 Kurtosis	0.42 (0.40)	0.40 (0.37)	0.1679	0.04
**Peer Problems**	Male	MELS 11 Minimum	-145.19 (33.11)	-144.87 (33.41)	0.7607	-0.01
**Peer Problems**	Male	MELS 11 Maximum	142.46 (31.39)	142.35 (31.48)	0.9121	0.00
**Peer Problems**	Male	MELS 11 Range	287.64 (57.88)	287.22 (58.54)	0.8152	0.01
**Peer Problems**	Male	MELS 11 Mean	-0.00 (0.59)	-0.00 (0.70)	0.9219	0.00
**Peer Problems**	Male	MELS 11 Std	41.27 (4.59)	41.33 (4.75)	0.7132	-0.01
**Peer Problems**	Male	MELS 11 Skewness	-0.01 (0.16)	-0.01 (0.16)	0.2392	-0.04
**Peer Problems**	Male	MELS 11 Kurtosis	0.39 (0.38)	0.38 (0.39)	0.3509	0.03
**Peer Problems**	Male	MELS 12 Minimum	-128.93 (27.93)	-129.43 (27.50)	0.5615	0.02
**Peer Problems**	Male	MELS 12 Maximum	130.04 (28.83)	129.58 (28.40)	0.6030	0.02
**Peer Problems**	Male	MELS 12 Range	258.97 (50.96)	259.00 (50.06)	0.9809	0.00
**Peer Problems**	Male	MELS 12 Mean	-0.02 (0.52)	-0.02 (0.53)	0.8391	-0.01
**Peer Problems**	Male	MELS 12 Std	37.12 (4.09)	37.10 (4.08)	0.8823	0.00
**Peer Problems**	Male	MELS 12 Skewness	0.00 (0.17)	-0.01 (0.16)	0.1351	0.05
**Peer Problems**	Male	MELS 12 Kurtosis	0.39 (0.42)	0.41 (0.40)	0.2935	-0.03
**Peer Problems**	Male	MELS 13 Minimum	-115.00 (24.63)	-114.60 (24.71)	0.6011	-0.02
**Peer Problems**	Male	MELS 13 Maximum	115.92 (25.15)	115.93 (25.89)	0.9883	0.00
**Peer Problems**	Male	MELS 13 Range	230.92 (44.57)	230.53 (45.50)	0.7806	0.01
**Peer Problems**	Male	MELS 13 Mean	0.01 (0.48)	-0.01 (0.50)	0.2064	0.04
**Peer Problems**	Male	MELS 13 Std	33.07 (3.57)	33.12 (3.66)	0.6661	-0.01
**Peer Problems**	Male	MELS 13 Skewness	0.01 (0.16)	0.01 (0.16)	0.9061	0.00
**Peer Problems**	Male	MELS 13 Kurtosis	0.39 (0.37)	0.38 (0.35)	0.3812	0.03
**Peer Problems**	Male	MELS 14 Minimum	-7.02 (3.93)	-7.02 (3.99)	0.9538	0.00

**Peer Problems**	Male	MELS 14 Maximum	8.36 (6.16)	8.41 (6.40)	0.7979	-0.01
**Peer Problems**	Male	MELS 14 Range	15.38 (9.78)	15.43 (10.12)	0.8899	0.00
**Peer Problems**	Male	MELS 14 Mean	-0.01 (0.02)	-0.01 (0.02)	0.6497	-0.01
**Peer Problems**	Male	MELS 14 Std	1.55 (0.31)	1.55 (0.33)	0.6101	-0.02
**Peer Problems**	Male	MELS 14 Skewness	0.70 (0.70)	0.72 (0.71)	0.4590	-0.02
**Peer Problems**	Male	MELS 14 Kurtosis	6.27 (19.95)	6.28 (19.75)	0.9809	0.00
**Peer Problems**	Male	MELS 15 Minimum	-227.44 (64.68)	-226.51 (63.77)	0.6399	-0.01
**Peer Problems**	Male	MELS 15 Maximum	333.74 (91.56)	334.20 (92.06)	0.8720	0.00
**Peer Problems**	Male	MELS 15 Range	561.18 (143.59)	560.70 (143.04)	0.9150	0.00
**Peer Problems**	Male	MELS 15 Mean	-0.00 (0.86)	0.02 (0.88)	0.3174	-0.03
**Peer Problems**	Male	MELS 15 Std	63.02 (11.28)	63.37 (11.51)	0.3153	-0.03
**Peer Problems**	Male	MELS 15 Skewness	0.84 (0.35)	0.85 (0.34)	0.1945	-0.04
**Peer Problems**	Male	MELS 15 Kurtosis	3.33 (1.58)	3.32 (1.61)	0.7310	0.01
**Peer Problems**	Male	MELS 16 Minimum	-251.70 (67.45)	-250.38 (69.84)	0.5336	-0.02
**Peer Problems**	Male	MELS 16 Maximum	235.79 (62.57)	232.36 (63.17)	0.0774	0.05
**Peer Problems**	Male	MELS 16 Range	487.49 (116.20)	482.74 (119.88)	0.1920	0.04
**Peer Problems**	Male	MELS 16 Mean	-0.15 (1.29)	-0.12 (0.93)	0.2842	-0.03
**Peer Problems**	Male	MELS 16 Std	60.60 (7.94)	60.26 (7.95)	0.1715	0.04
**Peer Problems**	Male	MELS 16 Skewness	-0.14 (0.23)	-0.15 (0.22)	0.1099	0.05
**Peer Problems**	Male	MELS 16 Kurtosis	1.15 (0.86)	1.12 (0.98)	0.3052	0.03
**Peer Problems**	Male	MELS 17 Minimum	-290.25 (72.46)	-290.08 (75.20)	0.9420	0.00
**Peer Problems**	Male	MELS 17 Maximum	285.84 (73.26)	281.85 (74.15)	0.0802	0.05
**Peer Problems**	Male	MELS 17 Range	576.09 (131.67)	571.94 (135.43)	0.3137	0.03
**Peer Problems**	Male	MELS 17 Mean	-0.15 (1.38)	-0.11 (1.26)	0.3451	-0.03
**Peer Problems**	Male	MELS 17 Std	72.49 (8.54)	72.23 (8.86)	0.3246	0.03
**Peer Problems**	Male	MELS 17 Skewness	-0.08 (0.19)	-0.09 (0.18)	0.1412	0.05
**Peer Problems**	Male	MELS 17 Kurtosis	1.05 (0.72)	1.02 (0.76)	0.1737	0.04
**Peer Problems**	Male	MELS 18 Minimum	-267.76 (63.81)	-266.52 (63.66)	0.5317	-0.02
**Peer Problems**	Male	MELS 18 Maximum	267.08 (65.60)	263.82 (62.53)	0.1026	0.05
**Peer Problems**	Male	MELS 18 Range	534.83 (115.50)	530.34 (111.95)	0.2038	0.04
**Peer Problems**	Male	MELS 18 Mean	-0.02 (1.31)	0.01 (1.35)	0.3479	-0.03
**Peer Problems**	Male	MELS 18 Std	70.98 (6.94)	70.82 (7.26)	0.4585	0.02
**Peer Problems**	Male	MELS 18 Skewness	-0.03 (0.17)	-0.03 (0.16)	0.8896	0.00
**Peer Problems**	Male	MELS 18 Kurtosis	0.68 (0.59)	0.67 (0.63)	0.4452	0.02
**Peer Problems**	Male	MELS 19 Minimum	-279.74 (67.88)	-279.20 (70.19)	0.7992	-0.01
**Peer Problems**	Male	MELS 19 Maximum	277.73 (62.19)	277.71 (65.80)	0.9953	0.00
**Peer Problems**	Male	MELS 19 Range	557.46 (114.17)	556.91 (120.27)	0.8781	0.00
**Peer Problems**	Male	MELS 19 Mean	-0.03 (1.30)	-0.09 (1.28)	0.1848	0.04
**Peer Problems**	Male	MELS 19 Std	74.88 (7.09)	74.54 (7.11)	0.1260	0.05
**Peer Problems**	Male	MELS 19 Skewness	0.01 (0.16)	0.00 (0.17)	0.0766	0.05

**Peer Problems**	Male	MELS 19 Kurtosis	0.64 (0.57)	0.65 (0.61)	0.5749	-0.02
**Peer Problems**	Male	MELS 20 Minimum	-297.71 (68.23)	-298.65 (67.69)	0.6576	0.01
**Peer Problems**	Male	MELS 20 Maximum	315.62 (75.78)	313.94 (77.68)	0.4761	0.02
**Peer Problems**	Male	MELS 20 Range	613.34 (127.64)	612.58 (130.27)	0.8497	0.01
**Peer Problems**	Male	MELS 20 Mean	0.04 (1.14)	-0.06 (1.22)	0.0061	0.08
**Peer Problems**	Male	MELS 20 Std	81.31 (7.36)	81.25 (7.55)	0.8091	0.01
**Peer Problems**	Male	MELS 20 Skewness	0.07 (0.15)	0.06 (0.16)	0.2773	0.03
**Peer Problems**	Male	MELS 20 Kurtosis	0.72 (0.61)	0.71 (0.60)	0.5813	0.02
**Peer Problems**	Male	MELS 21 Minimum	-302.04 (68.91)	-299.03 (66.40)	0.1535	-0.04
**Peer Problems**	Male	MELS 21 Maximum	306.77 (71.40)	304.31 (70.78)	0.2637	0.03
**Peer Problems**	Male	MELS 21 Range	608.81 (125.25)	603.34 (122.59)	0.1553	0.04
**Peer Problems**	Male	MELS 21 Mean	0.06 (1.17)	0.02 (1.57)	0.2867	0.03
**Peer Problems**	Male	MELS 21 Std	81.76 (7.19)	81.69 (7.13)	0.7593	0.01
**Peer Problems**	Male	MELS 21 Skewness	0.02 (0.14)	0.02 (0.14)	0.9504	0.00
**Peer Problems**	Male	MELS 21 Kurtosis	0.63 (0.53)	0.61 (0.51)	0.2192	0.04
**Peer Problems**	Male	MELS 22 Minimum	-290.67 (61.55)	-291.33 (62.54)	0.7289	0.01
**Peer Problems**	Male	MELS 22 Maximum	282.60 (58.16)	284.16 (58.75)	0.3864	-0.03
**Peer Problems**	Male	MELS 22 Range	573.26 (104.57)	575.49 (105.52)	0.4923	-0.02
**Peer Problems**	Male	MELS 22 Mean	0.03 (1.16)	0.05 (1.40)	0.7538	-0.01
**Peer Problems**	Male	MELS 22 Std	79.51 (6.24)	79.43 (6.29)	0.6935	0.01
**Peer Problems**	Male	MELS 22 Skewness	-0.02 (0.14)	-0.02 (0.15)	0.2715	-0.03
**Peer Problems**	Male	MELS 22 Kurtosis	0.49 (0.44)	0.50 (0.44)	0.5064	-0.02
**Peer Problems**	Male	MELS 23 Minimum	-273.36 (54.28)	-271.86 (54.20)	0.3735	-0.03
**Peer Problems**	Male	MELS 23 Maximum	272.38 (55.15)	271.28 (55.53)	0.5198	0.02
**Peer Problems**	Male	MELS 23 Range	545.74 (95.69)	543.15 (96.20)	0.3818	0.03
**Peer Problems**	Male	MELS 23 Mean	0.04 (1.14)	0.02 (1.55)	0.7120	0.01
**Peer Problems**	Male	MELS 23 Std	76.53 (5.74)	76.44 (5.70)	0.6344	0.01
**Peer Problems**	Male	MELS 23 Skewness	-0.01 (0.14)	-0.01 (0.13)	0.4887	0.02
**Peer Problems**	Male	MELS 23 Kurtosis	0.44 (0.42)	0.42 (0.40)	0.2662	0.03
**Peer Problems**	Male	MELS 24 Minimum	-254.96 (49.80)	-253.21 (49.59)	0.2556	-0.04
**Peer Problems**	Male	MELS 24 Maximum	256.38 (50.02)	256.85 (50.69)	0.7584	-0.01
**Peer Problems**	Male	MELS 24 Range	511.33 (87.83)	510.06 (88.04)	0.6405	0.01
**Peer Problems**	Male	MELS 24 Mean	0.02 (1.16)	0.06 (1.40)	0.4349	-0.02
**Peer Problems**	Male	MELS 24 Std	72.35 (5.50)	72.26 (5.41)	0.5749	0.02
**Peer Problems**	Male	MELS 24 Skewness	0.01 (0.13)	0.02 (0.13)	0.1315	-0.05
**Peer Problems**	Male	MELS 24 Kurtosis	0.40 (0.40)	0.39 (0.39)	0.4837	0.02
**Peer Problems**	Male	MELS 25 Minimum	-240.39 (46.79)	-239.70 (47.31)	0.6366	-0.01
**Peer Problems**	Male	MELS 25 Maximum	237.16 (45.85)	237.43 (46.54)	0.8472	-0.01
**Peer Problems**	Male	MELS 25 Range	477.55 (81.29)	477.13 (81.69)	0.8701	0.01
**Peer Problems**	Male	MELS 25 Mean	0.04 (0.88)	0.09 (1.27)	0.0968	-0.05

**Peer Problems**	Male	MELS 25 Std	67.70 (5.19)	67.70 (4.87)	0.9925	0.00
**Peer Problems**	Male	MELS 25 Skewness	-0.01 (0.13)	-0.00 (0.13)	0.2365	-0.04
**Peer Problems**	Male	MELS 25 Kurtosis	0.39 (0.42)	0.38 (0.41)	0.5281	0.02
**Peer Problems**	Male	MELS 26 Minimum	-219.05 (42.18)	-219.47 (42.03)	0.7495	0.01
**Peer Problems**	Male	MELS 26 Maximum	218.77 (41.36)	218.69 (41.66)	0.9479	0.00
**Peer Problems**	Male	MELS 26 Range	437.83 (73.22)	438.16 (73.15)	0.8834	0.00
**Peer Problems**	Male	MELS 26 Mean	0.01 (0.97)	0.02 (1.02)	0.7333	-0.01
**Peer Problems**	Male	MELS 26 Std	62.62 (4.81)	62.63 (4.90)	0.9064	0.00
**Peer Problems**	Male	MELS 26 Skewness	-0.01 (0.12)	-0.00 (0.12)	0.2178	-0.04
**Peer Problems**	Male	MELS 26 Kurtosis	0.36 (0.37)	0.37 (0.38)	0.4483	-0.02
**Peer Problems**	Male	F1 Minimum	219.11 (65.92)	220.40 (78.76)	0.5604	-0.02
**Peer Problems**	Male	F1 Maximum	4593.54 (906.80)	4590.80 (915.74)	0.9225	0.00
**Peer Problems**	Male	F1 Range	4374.43 (913.25)	4370.40 (921.09)	0.8872	0.00
**Peer Problems**	Male	F1 Mean	1051.85 (450.37)	1072.19 (460.94)	0.1485	-0.04
**Peer Problems**	Male	F1 Std	900.11 (254.07)	908.23 (257.17)	0.3048	-0.03
**Peer Problems**	Male	F1 Skewness	1.97 (1.06)	1.94 (1.05)	0.3258	0.03
**Peer Problems**	Male	F1 Kurtosis	4.84 (5.43)	4.68 (5.25)	0.3148	0.03
**Peer Problems**	Male	F2 Minimum	609.72 (429.09)	619.56 (456.65)	0.4703	-0.02
**Peer Problems**	Male	F2 Maximum	6305.07 (570.20)	6296.66 (566.95)	0.6331	0.01
**Peer Problems**	Male	F2 Range	5695.35 (781.74)	5677.10 (807.91)	0.4569	0.02
**Peer Problems**	Male	F2 Mean	2562.08 (647.29)	2582.91 (660.48)	0.3026	-0.03
**Peer Problems**	Male	F2 Std	1238.96 (218.53)	1248.88 (228.73)	0.1502	-0.04
**Peer Problems**	Male	F2 Skewness	0.84 (0.61)	0.84 (0.61)	0.9294	0.00
**Peer Problems**	Male	F2 Kurtosis	0.48 (1.31)	0.44 (1.30)	0.3570	0.03
**Peer Problems**	Male	F3 Minimum	1911.32 (750.79)	1913.70 (762.51)	0.9192	0.00
**Peer Problems**	Male	F3 Maximum	6985.55 (405.25)	6965.18 (422.23)	0.1105	0.05
**Peer Problems**	Male	F3 Range	5074.23 (1007.76)	5051.48 (1020.90)	0.4685	0.02
**Peer Problems**	Male	F3 Mean	4384.86 (508.55)	4385.67 (519.24)	0.9594	0.00
**Peer Problems**	Male	F3 Std	1189.29 (209.55)	1189.82 (217.67)	0.9356	0.00
**Peer Problems**	Male	F3 Skewness	0.10 (0.59)	0.07 (0.62)	0.2142	0.04
**Peer Problems**	Male	F3 Kurtosis	-0.51 (1.03)	-0.48 (1.18)	0.4374	-0.02
**Peer Problems**	Male	F1 1st Derivative Minim	-1884.23 (469.96)	-1864.00 (464.48)	0.1626	-0.04
**Peer Problems**	Male	F1 1st Derivative Maxin	1879.17 (485.61)	1869.18 (474.88)	0.5029	0.02
**Peer Problems**	Male	F1 1st Derivative Range	3763.40 (900.76)	3733.18 (885.98)	0.2756	0.03
**Peer Problems**	Male	F1 1st Derivative Mean	-5.02 (25.71)	-5.42 (22.63)	0.5999	0.02
**Peer Problems**	Male	F1 1st Derivative Std	493.67 (126.53)	493.57 (126.45)	0.9816	0.00
**Peer Problems**	Male	F1 1st Derivative Skewı	0.04 (0.47)	0.05 (0.46)	0.4472	-0.02
**Peer Problems**	Male	F1 1st Derivative Kurtos	5.62 (4.04)	5.59 (4.08)	0.8154	0.01
**Peer Problems**	Male	F2 1st Derivative Minim	-2214.18 (464.75)	-2205.33 (476.55)	0.5428	-0.02
**Peer Problems**	Male	F2 1st Derivative Maxin	2194.63 (502.48)	2178.03 (504.42)	0.2870	0.03

**Peer Problems**	Male	F2 1st Derivative Range	4408.81 (891.40)	4383.36 (908.64)	0.3604	0.03
**Peer Problems**	Male	F2 1st Derivative Mean	-7.55 (33.62)	-9.31 (30.18)	0.0796	0.05
**Peer Problems**	Male	F2 1st Derivative Std	665.97 (110.45)	663.44 (109.68)	0.4591	0.02
**Peer Problems**	Male	F2 1st Derivative Skewı	-0.00 (0.35)	-0.01 (0.34)	0.7952	0.01
**Peer Problems**	Male	F2 1st Derivative Kurtos	2.22 (1.45)	2.20 (1.40)	0.6440	0.01
**Peer Problems**	Male	F3 1st Derivative Minim	-1970.03 (491.92)	-1976.37 (482.79)	0.6752	0.01
**Peer Problems**	Male	F3 1st Derivative Maxin	1970.88 (523.06)	1964.89 (530.95)	0.7132	0.01
**Peer Problems**	Male	F3 1st Derivative Range	3940.91 (960.72)	3941.26 (961.32)	0.9906	0.00
**Peer Problems**	Male	F3 1st Derivative Mean	-5.83 (39.02)	-7.80 (37.75)	0.0972	0.05
**Peer Problems**	Male	F3 1st Derivative Std	680.96 (135.29)	679.62 (137.39)	0.7507	0.01
**Peer Problems**	Male	F3 1st Derivative Skewı	-0.01 (0.34)	-0.02 (0.38)	0.2549	0.03
**Peer Problems**	Male	F3 1st Derivative Kurtos	1.03 (0.90)	1.09 (1.02)	0.0531	-0.06
**Peer Problems**	Male	F1 2nd Derivative Minin	-6485.55 (2057.61)	-6419.65 (2064.81)	0.3018	-0.03
**Peer Problems**	Male	F1 2nd Derivative Maxii	4734.89 (1444.35)	4678.36 (1431.81)	0.2049	0.04
**Peer Problems**	Male	F1 2nd Derivative Rang	11220.44 (3153.94)	11098.01 (3180.50)	0.2116	0.04
**Peer Problems**	Male	F1 2nd Derivative Mear	-0.41 (45.69)	-0.09 (38.58)	0.8082	-0.01
**Peer Problems**	Male	F1 2nd Derivative Std	1265.43 (378.71)	1257.02 (366.81)	0.4680	0.02
**Peer Problems**	Male	F1 2nd Derivative Skew	-0.77 (0.64)	-0.76 (0.65)	0.8826	0.00
**Peer Problems**	Male	F1 2nd Derivative Kurtc	8.53 (6.15)	8.51 (6.45)	0.9014	0.00
**Peer Problems**	Male	F2 2nd Derivative Minin	-7446.84 (2058.79)	-7431.25 (2091.35)	0.8080	-0.01
**Peer Problems**	Male	F2 2nd Derivative Maxii	5992.58 (1713.59)	5972.54 (1725.97)	0.7065	0.01
**Peer Problems**	Male	F2 2nd Derivative Rang	13439.42 (3408.89)	13403.78 (3445.38)	0.7368	0.01
**Peer Problems**	Male	F2 2nd Derivative Mear	0.56 (58.87)	2.18 (49.50)	0.3436	-0.03
**Peer Problems**	Male	F2 2nd Derivative Std	1721.40 (341.04)	1719.56 (347.64)	0.8627	0.01
**Peer Problems**	Male	F2 2nd Derivative Skew	-0.52 (0.43)	-0.52 (0.43)	0.7764	-0.01
**Peer Problems**	Male	F2 2nd Derivative Kurtc	4.08 (2.53)	4.09 (2.52)	0.9469	0.00
**Peer Problems**	Male	F3 2nd Derivative Minin	-6323.98 (1975.30)	-6331.31 (2001.36)	0.9052	0.00
**Peer Problems**	Male	F3 2nd Derivative Maxii	6111.93 (2009.91)	6067.83 (1989.05)	0.4769	0.02
**Peer Problems**	Male	F3 2nd Derivative Rang	12435.91 (3724.24)	12399.14 (3721.67)	0.7498	0.01
**Peer Problems**	Male	F3 2nd Derivative Mear	1.40 (71.45)	3.62 (68.96)	0.3083	-0.03
**Peer Problems**	Male	F3 2nd Derivative Std	1754.85 (385.76)	1757.24 (378.95)	0.8401	-0.01
**Peer Problems**	Male	F3 2nd Derivative Skew	-0.16 (0.41)	-0.17 (0.42)	0.4750	0.02
**Peer Problems**	Male	F3 2nd Derivative Kurtc	2.22 (1.45)	2.25 (1.48)	0.4583	-0.02
**Hyperactivity**	Female	F0 Minimum	58.79 (11.39)	58.53 (12.72)	0.6262	0.02
**Hyperactivity**	Female	F0 Maximum	483.26 (2.66)	482.94 (5.83)	0.1531	0.07
**Hyperactivity**	Female	F0 Range	424.47 (12.37)	424.41 (15.70)	0.9139	0.00
**Hyperactivity**	Female	F0 Mean	282.34 (16.18)	282.91 (17.20)	0.4266	-0.03
**Hyperactivity**	Female	F0 Std	113.30 (6.63)	112.60 (7.65)	0.0264	0.10
**Hyperactivity**	Female	F0 Skewness	0.12 (0.16)	0.12 (0.18)	0.9055	-0.01
**Hyperactivity**	Female	F0 Kurtosis	-1.15 (0.22)	-1.13 (0.35)	0.1577	-0.07

**Hyperactivity**	Female	F0 de Minimum	-178.16 (16.48)	-176.92 (19.13)	0.1138	-0.07
**Hyperactivity**	Female	F0 de Maximum	187.98 (12.83)	187.57 (14.99)	0.5035	0.03
**Hyperactivity**	Female	F0 de Range	366.15 (25.06)	364.49 (29.77)	0.1747	0.06
**Hyperactivity**	Female	F0 de Mean	0.15 (0.40)	0.21 (0.72)	0.0283	-0.11
**Hyperactivity**	Female	F0 de Std	76.41 (12.69)	75.16 (13.71)	0.0287	0.09
**Hyperactivity**	Female	F0 de Skewness	0.31 (0.17)	0.32 (0.19)	0.0618	-0.08
**Hyperactivity**	Female	F0 de Kurtosis	-0.48 (0.92)	-0.35 (1.06)	0.0035	-0.13
**Hyperactivity**	Female	F0 de2 Minimum	-652.19 (58.02)	-646.69 (61.91)	0.0329	-0.09
**Hyperactivity**	Female	F0 de2 Maximum	613.57 (78.77)	608.48 (78.09)	0.1221	0.06
**Hyperactivity**	Female	F0 de2 Range	1265.76 (125.06)	1255.18 (129.20)	0.0503	0.08
**Hyperactivity**	Female	F0 de2 Mean	0.01 (0.85)	0.12 (1.47)	0.0418	-0.10
**Hyperactivity**	Female	F0 de2 Std	297.93 (22.52)	300.07 (25.41)	0.0410	-0.09
**Hyperactivity**	Female	F0 de2 Skewness	-0.06 (0.04)	-0.06 (0.07)	0.3409	0.05
**Hyperactivity**	Female	F0 de2 Kurtosis	-1.08 (0.26)	-1.09 (0.32)	0.2515	0.05
**Hyperactivity**	Female	Energy Minimum	-11.55 (6.09)	-11.48 (6.24)	0.7840	-0.01
**Hyperactivity**	Female	Energy Maximum	3.74 (1.24)	3.89 (1.24)	0.0033	-0.12
**Hyperactivity**	Female	Energy Range	15.29 (6.54)	15.37 (6.62)	0.7684	-0.01
**Hyperactivity**	Female	Energy Mean	-2.12 (0.94)	-2.03 (1.13)	0.0511	-0.09
**Hyperactivity**	Female	Energy Std	2.61 (0.55)	2.63 (0.59)	0.3807	-0.04
**Hyperactivity**	Female	Energy Skewness	-0.76 (0.65)	-0.73 (0.68)	0.2869	-0.05
**Hyperactivity**	Female	Energy Kurtosis	1.65 (5.44)	1.69 (5.51)	0.8639	-0.01
**Hyperactivity**	Female	Energy de Minimum	-3.48 (1.67)	-3.40 (1.64)	0.2627	-0.05
**Hyperactivity**	Female	Energy de Maximum	4.50 (2.13)	4.50 (2.17)	0.9783	0.00
**Hyperactivity**	Female	Energy de Range	7.98 (3.50)	7.90 (3.48)	0.5854	0.02
**Hyperactivity**	Female	Energy de Mean	0.01 (0.02)	0.01 (0.03)	0.0031	-0.14
**Hyperactivity**	Female	Energy de Std	1.11 (0.21)	1.10 (0.22)	0.4797	0.03
**Hyperactivity**	Female	Energy de Skewness	0.36 (0.45)	0.42 (0.53)	0.0120	-0.11
**Hyperactivity**	Female	Energy de Kurtosis	1.67 (3.61)	1.81 (3.82)	0.3893	-0.04
**Hyperactivity**	Female	Energy de2 Minimum	-7.05 (3.82)	-6.93 (3.79)	0.4373	-0.03
**Hyperactivity**	Female	Energy de2 Maximum	8.30 (5.45)	8.23 (5.76)	0.7718	0.01
**Hyperactivity**	Female	Energy de2 Range	15.36 (8.94)	15.16 (9.27)	0.6171	0.02
**Hyperactivity**	Female	Energy de2 Mean	-0.01 (0.01)	-0.01 (0.02)	0.0383	0.10
**Hyperactivity**	Female	Energy de2 Std	1.56 (0.28)	1.54 (0.32)	0.1424	0.06
**Hyperactivity**	Female	Energy de2 Skewness	0.66 (0.57)	0.69 (0.63)	0.2139	-0.05
**Hyperactivity**	Female	Energy de2 Kurtosis	5.52 (15.30)	5.70 (17.37)	0.7967	-0.01
**Hyperactivity**	Female	MFCC 1 Minimum	-11.55 (6.09)	-11.48 (6.24)	0.7840	-0.01
**Hyperactivity**	Female	MFCC 1 Maximum	3.74 (1.24)	3.89 (1.24)	0.0033	-0.12
**Hyperactivity**	Female	MFCC 1 Range	15.29 (6.54)	15.37 (6.62)	0.7684	-0.01
**Hyperactivity**	Female	MFCC 1 Mean	-2.12 (0.94)	-2.03 (1.13)	0.0511	-0.09
**Hyperactivity**	Female	MFCC 1 Std	2.61 (0.55)	2.63 (0.59)	0.3807	-0.04

**Hyperactivity**	Female	MFCC 1 Skewness	-0.76 (0.65)	-0.73 (0.68)	0.2869	-0.05
**Hyperactivity**	Female	MFCC 1 Kurtosis	1.65 (5.44)	1.69 (5.51)	0.8639	-0.01
**Hyperactivity**	Female	MFCC 2 Minimum	-486.82 (84.26)	-472.16 (107.81)	0.0008	-0.15
**Hyperactivity**	Female	MFCC 2 Maximum	359.55 (53.03)	351.27 (63.22)	0.0014	0.14
**Hyperactivity**	Female	MFCC 2 Range	846.37 (123.46)	823.43 (159.40)	0.0004	0.16
**Hyperactivity**	Female	MFCC 2 Mean	41.78 (21.12)	41.59 (22.63)	0.8414	0.01
**Hyperactivity**	Female	MFCC 2 Std	159.69 (22.46)	155.48 (27.56)	0.0002	0.17
**Hyperactivity**	Female	MFCC 2 Skewness	-0.70 (0.25)	-0.68 (0.30)	0.1123	-0.07
**Hyperactivity**	Female	MFCC 2 Kurtosis	0.29 (0.47)	0.28 (0.51)	0.7582	0.01
**Hyperactivity**	Female	MFCC 3 Minimum	-397.51 (99.88)	-392.17 (116.57)	0.2646	-0.05
**Hyperactivity**	Female	MFCC 3 Maximum	415.98 (70.52)	408.67 (78.46)	0.0243	0.10
**Hyperactivity**	Female	MFCC 3 Range	813.49 (152.66)	800.84 (179.52)	0.0859	0.08
**Hyperactivity**	Female	MFCC 3 Mean	28.27 (28.26)	29.58 (29.25)	0.2830	-0.05
**Hyperactivity**	Female	MFCC 3 Std	149.88 (24.84)	147.19 (28.69)	0.0225	0.10
**Hyperactivity**	Female	MFCC 3 Skewness	-0.19 (0.21)	-0.20 (0.25)	0.5036	0.03
**Hyperactivity**	Female	MFCC 3 Kurtosis	-0.33 (0.31)	-0.28 (0.37)	0.0022	-0.14
**Hyperactivity**	Female	MFCC 4 Minimum	-467.87 (102.18)	-455.34 (117.19)	0.0095	-0.11
**Hyperactivity**	Female	MFCC 4 Maximum	553.24 (101.44)	538.37 (112.46)	0.0014	0.14
**Hyperactivity**	Female	MFCC 4 Range	1021.11 (179.78)	993.72 (208.09)	0.0014	0.14
**Hyperactivity**	Female	MFCC 4 Mean	20.87 (31.51)	19.37 (31.86)	0.2609	0.05
**Hyperactivity**	Female	MFCC 4 Std	190.21 (27.87)	185.51 (32.56)	0.0004	0.16
**Hyperactivity**	Female	MFCC 4 Skewness	0.15 (0.23)	0.16 (0.25)	0.4743	-0.03
**Hyperactivity**	Female	MFCC 4 Kurtosis	-0.28 (0.34)	-0.28 (0.37)	0.9543	0.00
**Hyperactivity**	Female	MFCC 5 Minimum	-469.68 (122.56)	-449.38 (131.10)	0.0002	-0.16
**Hyperactivity**	Female	MFCC 5 Maximum	406.74 (84.85)	392.96 (93.86)	0.0004	0.15
**Hyperactivity**	Female	MFCC 5 Range	876.42 (182.95)	842.33 (201.39)	0.0000	0.18
**Hyperactivity**	Female	MFCC 5 Mean	-9.09 (29.08)	-7.49 (29.99)	0.2020	-0.05
**Hyperactivity**	Female	MFCC 5 Std	146.29 (25.02)	139.84 (26.96)	0.0000	0.25
**Hyperactivity**	Female	MFCC 5 Skewness	-0.14 (0.23)	-0.15 (0.25)	0.8151	0.01
**Hyperactivity**	Female	MFCC 5 Kurtosis	-0.02 (0.37)	0.02 (0.44)	0.0255	-0.10
**Hyperactivity**	Female	MFCC 6 Minimum	-454.27 (104.26)	-450.01 (116.41)	0.3756	-0.04
**Hyperactivity**	Female	MFCC 6 Maximum	408.10 (88.67)	401.81 (97.18)	0.1184	0.07
**Hyperactivity**	Female	MFCC 6 Range	862.37 (160.03)	851.82 (185.04)	0.1655	0.06
**Hyperactivity**	Female	MFCC 6 Mean	-14.50 (31.13)	-14.26 (32.27)	0.8547	-0.01
**Hyperactivity**	Female	MFCC 6 Std	142.38 (19.59)	141.77 (23.32)	0.5182	0.03
**Hyperactivity**	Female	MFCC 6 Skewness	-0.14 (0.23)	-0.15 (0.24)	0.1248	0.07
**Hyperactivity**	Female	MFCC 6 Kurtosis	0.03 (0.36)	0.02 (0.39)	0.6088	0.02
**Hyperactivity**	Female	MFCC 7 Minimum	-523.36 (114.66)	-517.86 (128.15)	0.2989	-0.05
**Hyperactivity**	Female	MFCC 7 Maximum	376.58 (85.75)	361.91 (92.70)	0.0001	0.16
**Hyperactivity**	Female	MFCC 7 Range	899.93 (168.34)	879.76 (192.04)	0.0108	0.11

**Hyperactivity**	Female	MFCC 7 Mean	-51.51 (32.19)	-53.68 (35.00)	0.1346	0.06
**Hyperactivity**	Female	MFCC 7 Std	149.96 (21.03)	147.42 (24.41)	0.0115	0.11
**Hyperactivity**	Female	MFCC 7 Skewness	-0.19 (0.21)	-0.20 (0.24)	0.0910	0.07
**Hyperactivity**	Female	MFCC 7 Kurtosis	-0.03 (0.33)	-0.02 (0.39)	0.7294	-0.02
**Hyperactivity**	Female	MFCC 8 Minimum	-517.71 (122.68)	-505.06 (129.94)	0.0195	-0.10
**Hyperactivity**	Female	MFCC 8 Maximum	363.73 (81.64)	355.28 (91.72)	0.0257	0.10
**Hyperactivity**	Female	MFCC 8 Range	881.44 (170.23)	860.34 (190.76)	0.0074	0.12
**Hyperactivity**	Female	MFCC 8 Mean	-59.03 (33.54)	-59.78 (34.83)	0.6096	0.02
**Hyperactivity**	Female	MFCC 8 Std	144.33 (20.53)	141.61 (22.85)	0.0041	0.13
**Hyperactivity**	Female	MFCC 8 Skewness	-0.15 (0.24)	-0.14 (0.26)	0.5921	-0.02
**Hyperactivity**	Female	MFCC 8 Kurtosis	0.05 (0.36)	0.04 (0.43)	0.7452	0.01
**Hyperactivity**	Female	MFCC 9 Minimum	-442.04 (92.61)	-428.57 (102.18)	0.0015	-0.14
**Hyperactivity**	Female	MFCC 9 Maximum	377.18 (80.54)	366.93 (91.41)	0.0066	0.12
**Hyperactivity**	Female	MFCC 9 Range	819.22 (142.38)	795.50 (165.20)	0.0005	0.15
**Hyperactivity**	Female	MFCC 9 Mean	-30.57 (28.36)	-31.20 (28.66)	0.5976	0.02
**Hyperactivity**	Female	MFCC 9 Std	135.17 (16.08)	132.36 (19.54)	0.0004	0.16
**Hyperactivity**	Female	MFCC 9 Skewness	-0.03 (0.24)	-0.02 (0.23)	0.5435	-0.03
**Hyperactivity**	Female	MFCC 9 Kurtosis	-0.00 (0.37)	-0.05 (0.39)	0.0094	0.11
**Hyperactivity**	Female	MFCC 10 Minimum	-392.03 (84.10)	-380.09 (93.77)	0.0021	-0.13
**Hyperactivity**	Female	MFCC 10 Maximum	350.57 (87.87)	343.82 (93.84)	0.0838	0.07
**Hyperactivity**	Female	MFCC 10 Range	742.59 (148.27)	723.90 (163.63)	0.0058	0.12
**Hyperactivity**	Female	MFCC 10 Mean	-23.91 (24.51)	-22.38 (25.42)	0.1497	-0.06
**Hyperactivity**	Female	MFCC 10 Std	121.08 (18.37)	118.41 (20.96)	0.0020	0.14
**Hyperactivity**	Female	MFCC 10 Skewness	0.01 (0.23)	0.02 (0.24)	0.2534	-0.05
**Hyperactivity**	Female	MFCC 10 Kurtosis	0.04 (0.39)	0.04 (0.42)	0.8686	-0.01
**Hyperactivity**	Female	MFCC 11 Minimum	-355.78 (77.00)	-349.71 (86.55)	0.0891	-0.07
**Hyperactivity**	Female	MFCC 11 Maximum	298.94 (80.43)	289.15 (82.89)	0.0048	0.12
**Hyperactivity**	Female	MFCC 11 Range	654.72 (128.00)	638.86 (141.53)	0.0068	0.12
**Hyperactivity**	Female	MFCC 11 Mean	-25.28 (25.98)	-24.81 (25.98)	0.6684	-0.02
**Hyperactivity**	Female	MFCC 11 Std	103.83 (14.55)	101.39 (16.11)	0.0003	0.16
**Hyperactivity**	Female	MFCC 11 Skewness	-0.03 (0.24)	-0.05 (0.26)	0.1203	0.07
**Hyperactivity**	Female	MFCC 11 Kurtosis	0.14 (0.41)	0.15 (0.44)	0.4849	-0.03
**Hyperactivity**	Female	MFCC 12 Minimum	-311.82 (69.08)	-301.26 (76.58)	0.0009	-0.14
**Hyperactivity**	Female	MFCC 12 Maximum	273.05 (65.34)	264.81 (68.81)	0.0041	0.12
**Hyperactivity**	Female	MFCC 12 Range	584.87 (111.92)	566.08 (123.30)	0.0002	0.16
**Hyperactivity**	Female	MFCC 12 Mean	-15.36 (18.89)	-14.88 (20.41)	0.5697	-0.02
**Hyperactivity**	Female	MFCC 12 Std	89.44 (11.08)	87.77 (13.08)	0.0018	0.14
**Hyperactivity**	Female	MFCC 12 Skewness	-0.05 (0.23)	-0.05 (0.25)	0.7977	-0.01
**Hyperactivity**	Female	MFCC 12 Kurtosis	0.26 (0.40)	0.24 (0.45)	0.4136	0.04
**Hyperactivity**	Female	MFCC 13 Minimum	-256.25 (61.21)	-249.46 (67.83)	0.0156	-0.11

**Hyperactivity**	Female	MFCC 13 Maximum	256.67 (60.34)	254.20 (64.50)	0.3582	0.04
**Hyperactivity**	Female	MFCC 13 Range	512.91 (100.66)	503.66 (111.16)	0.0443	0.09
**Hyperactivity**	Female	MFCC 13 Mean	-1.15 (16.97)	0.90 (18.74)	0.0084	-0.11
**Hyperactivity**	Female	MFCC 13 Std	77.77 (10.65)	76.79 (11.84)	0.0448	0.09
**Hyperactivity**	Female	MFCC 13 Skewness	0.03 (0.24)	0.03 (0.25)	0.5836	-0.02
**Hyperactivity**	Female	MFCC 13 Kurtosis	0.32 (0.42)	0.31 (0.46)	0.7594	0.01
**Hyperactivity**	Female	MELS 1 Minimum	-3.48 (1.67)	-3.40 (1.64)	0.2627	-0.05
**Hyperactivity**	Female	MELS 1 Maximum	4.50 (2.13)	4.50 (2.17)	0.9783	0.00
**Hyperactivity**	Female	MELS 1 Range	7.98 (3.50)	7.90 (3.48)	0.5854	0.02
**Hyperactivity**	Female	MELS 1 Mean	0.01 (0.02)	0.01 (0.03)	0.0031	-0.14
**Hyperactivity**	Female	MELS 1 Std	1.11 (0.21)	1.10 (0.22)	0.4797	0.03
**Hyperactivity**	Female	MELS 1 Skewness	0.36 (0.45)	0.42 (0.53)	0.0120	-0.11
**Hyperactivity**	Female	MELS 1 Kurtosis	1.67 (3.61)	1.81 (3.82)	0.3893	-0.04
**Hyperactivity**	Female	MELS 2 Minimum	-214.39 (41.37)	-207.13 (49.50)	0.0003	-0.16
**Hyperactivity**	Female	MELS 2 Maximum	197.14 (39.18)	193.18 (44.65)	0.0313	0.09
**Hyperactivity**	Female	MELS 2 Range	411.53 (76.11)	400.32 (90.25)	0.0024	0.13
**Hyperactivity**	Female	MELS 2 Mean	0.11 (0.84)	0.18 (0.81)	0.0396	-0.09
**Hyperactivity**	Female	MELS 2 Std	59.09 (10.49)	58.01 (12.05)	0.0295	0.10
**Hyperactivity**	Female	MELS 2 Skewness	-0.12 (0.22)	-0.09 (0.23)	0.0254	-0.10
**Hyperactivity**	Female	MELS 2 Kurtosis	1.12 (0.61)	1.08 (0.60)	0.1074	0.07
**Hyperactivity**	Female	MELS 3 Minimum	-176.22 (37.88)	-173.24 (43.61)	0.0964	-0.07
**Hyperactivity**	Female	MELS 3 Maximum	168.87 (39.60)	165.67 (43.04)	0.0726	0.08
**Hyperactivity**	Female	MELS 3 Range	345.10 (70.41)	338.91 (81.28)	0.0642	0.08
**Hyperactivity**	Female	MELS 3 Mean	0.06 (0.53)	0.07 (0.72)	0.6833	-0.02
**Hyperactivity**	Female	MELS 3 Std	50.65 (8.00)	49.94 (9.10)	0.0575	0.08
**Hyperactivity**	Female	MELS 3 Skewness	-0.12 (0.19)	-0.12 (0.19)	0.9995	0.00
**Hyperactivity**	Female	MELS 3 Kurtosis	0.45 (0.41)	0.45 (0.46)	0.8607	0.01
**Hyperactivity**	Female	MELS 4 Minimum	-213.31 (43.37)	-207.01 (47.56)	0.0014	-0.14
**Hyperactivity**	Female	MELS 4 Maximum	205.48 (45.87)	201.91 (48.75)	0.0793	0.08
**Hyperactivity**	Female	MELS 4 Range	418.79 (79.03)	408.92 (87.57)	0.0065	0.12
**Hyperactivity**	Female	MELS 4 Mean	-0.01 (0.82)	0.05 (0.78)	0.0784	-0.07
**Hyperactivity**	Female	MELS 4 Std	61.00 (7.70)	59.80 (8.76)	0.0009	0.15
**Hyperactivity**	Female	MELS 4 Skewness	-0.08 (0.17)	-0.07 (0.20)	0.1429	-0.06
**Hyperactivity**	Female	MELS 4 Kurtosis	0.41 (0.44)	0.43 (0.48)	0.4532	-0.03
**Hyperactivity**	Female	MELS 5 Minimum	-182.89 (40.66)	-176.73 (43.60)	0.0007	-0.15
**Hyperactivity**	Female	MELS 5 Maximum	176.18 (38.19)	168.63 (39.69)	0.0000	0.19
**Hyperactivity**	Female	MELS 5 Range	359.07 (71.82)	345.36 (76.30)	0.0000	0.19
**Hyperactivity**	Female	MELS 5 Mean	0.06 (0.53)	0.06 (0.62)	0.9306	0.00
**Hyperactivity**	Female	MELS 5 Std	52.41 (7.17)	50.77 (7.65)	0.0000	0.22
**Hyperactivity**	Female	MELS 5 Skewness	-0.05 (0.15)	-0.06 (0.18)	0.8479	0.01

**Hyperactivity**	Female	MELS 5 Kurtosis	0.32 (0.33)	0.35 (0.43)	0.0533	-0.09
**Hyperactivity**	Female	MELS 6 Minimum	-183.41 (38.34)	-181.16 (42.74)	0.2031	-0.06
**Hyperactivity**	Female	MELS 6 Maximum	180.69 (37.02)	178.94 (41.64)	0.3066	0.04
**Hyperactivity**	Female	MELS 6 Range	364.10 (65.73)	360.10 (76.66)	0.2032	0.06
**Hyperactivity**	Female	MELS 6 Mean	0.02 (0.66)	0.06 (0.77)	0.2162	-0.05
**Hyperactivity**	Female	MELS 6 Std	52.28 (6.03)	51.99 (6.98)	0.3090	0.04
**Hyperactivity**	Female	MELS 6 Skewness	-0.02 (0.16)	-0.01 (0.17)	0.2028	-0.06
**Hyperactivity**	Female	MELS 6 Kurtosis	0.42 (0.41)	0.41 (0.43)	0.8324	0.01
**Hyperactivity**	Female	MELS 7 Minimum	-198.99 (41.07)	-196.62 (46.43)	0.2168	-0.05
**Hyperactivity**	Female	MELS 7 Maximum	189.90 (39.85)	186.78 (44.27)	0.0890	0.07
**Hyperactivity**	Female	MELS 7 Range	388.88 (72.80)	383.40 (83.46)	0.1108	0.07
**Hyperactivity**	Female	MELS 7 Mean	0.03 (0.57)	-0.01 (0.84)	0.2340	0.06
**Hyperactivity**	Female	MELS 7 Std	55.67 (6.63)	54.99 (7.63)	0.0303	0.10
**Hyperactivity**	Female	MELS 7 Skewness	-0.07 (0.16)	-0.07 (0.17)	0.8906	0.01
**Hyperactivity**	Female	MELS 7 Kurtosis	0.45 (0.35)	0.46 (0.42)	0.5959	-0.02
**Hyperactivity**	Female	MELS 8 Minimum	-193.51 (43.05)	-189.86 (46.00)	0.0564	-0.08
**Hyperactivity**	Female	MELS 8 Maximum	189.01 (40.89)	185.04 (44.15)	0.0306	0.09
**Hyperactivity**	Female	MELS 8 Range	382.52 (74.62)	374.90 (82.53)	0.0257	0.10
**Hyperactivity**	Female	MELS 8 Mean	-0.01 (0.56)	-0.04 (0.69)	0.1927	0.06
**Hyperactivity**	Female	MELS 8 Std	54.11 (6.34)	53.24 (7.32)	0.0039	0.13
**Hyperactivity**	Female	MELS 8 Skewness	-0.04 (0.16)	-0.04 (0.18)	0.7528	0.01
**Hyperactivity**	Female	MELS 8 Kurtosis	0.48 (0.41)	0.49 (0.44)	0.7391	-0.01
**Hyperactivity**	Female	MELS 9 Minimum	-179.28 (35.78)	-176.22 (40.25)	0.0659	-0.08
**Hyperactivity**	Female	MELS 9 Maximum	178.55 (35.68)	175.13 (40.01)	0.0383	0.09
**Hyperactivity**	Female	MELS 9 Range	357.83 (62.97)	351.35 (72.45)	0.0298	0.10
**Hyperactivity**	Female	MELS 9 Mean	-0.04 (0.55)	-0.02 (0.72)	0.4663	-0.03
**Hyperactivity**	Female	MELS 9 Std	51.41 (5.12)	50.41 (6.07)	0.0001	0.18
**Hyperactivity**	Female	MELS 9 Skewness	0.01 (0.15)	0.01 (0.17)	0.4076	0.04
**Hyperactivity**	Female	MELS 9 Kurtosis	0.43 (0.36)	0.43 (0.43)	0.8153	0.01
**Hyperactivity**	Female	MELS 10 Minimum	-164.82 (35.46)	-161.32 (38.42)	0.0285	-0.09
**Hyperactivity**	Female	MELS 10 Maximum	161.34 (33.59)	158.83 (36.63)	0.0986	0.07
**Hyperactivity**	Female	MELS 10 Range	326.16 (61.60)	320.16 (68.10)	0.0332	0.09
**Hyperactivity**	Female	MELS 10 Mean	0.00 (0.59)	0.01 (0.63)	0.7705	-0.01
**Hyperactivity**	Female	MELS 10 Std	46.61 (4.91)	45.82 (5.73)	0.0008	0.15
**Hyperactivity**	Female	MELS 10 Skewness	-0.01 (0.16)	-0.01 (0.17)	0.8741	-0.01
**Hyperactivity**	Female	MELS 10 Kurtosis	0.42 (0.36)	0.41 (0.40)	0.8995	0.01
**Hyperactivity**	Female	MELS 11 Minimum	-147.14 (30.80)	-144.49 (33.24)	0.0549	-0.08
**Hyperactivity**	Female	MELS 11 Maximum	146.42 (29.59)	142.42 (32.49)	0.0030	0.13
**Hyperactivity**	Female	MELS 11 Range	293.56 (53.33)	286.91 (59.48)	0.0069	0.12
**Hyperactivity**	Female	MELS 11 Mean	-0.03 (0.38)	-0.03 (0.67)	0.9453	0.00

**Hyperactivity**	Female	MELS 11 Std	41.93 (4.14)	41.31 (4.85)	0.0018	0.14
**Hyperactivity**	Female	MELS 11 Skewness	-0.01 (0.15)	-0.01 (0.16)	0.4851	0.03
**Hyperactivity**	Female	MELS 11 Kurtosis	0.40 (0.38)	0.38 (0.40)	0.3088	0.04
**Hyperactivity**	Female	MELS 12 Minimum	-130.12 (25.32)	-127.89 (28.46)	0.0577	-0.08
**Hyperactivity**	Female	MELS 12 Maximum	130.90 (27.63)	128.53 (29.94)	0.0559	0.08
**Hyperactivity**	Female	MELS 12 Range	261.03 (46.55)	256.42 (52.72)	0.0341	0.09
**Hyperactivity**	Female	MELS 12 Mean	-0.01 (0.36)	-0.02 (0.54)	0.7752	0.01
**Hyperactivity**	Female	MELS 12 Std	37.46 (3.69)	36.87 (4.19)	0.0007	0.15
**Hyperactivity**	Female	MELS 12 Skewness	-0.00 (0.15)	-0.00 (0.16)	0.6647	0.02
**Hyperactivity**	Female	MELS 12 Kurtosis	0.38 (0.35)	0.37 (0.38)	0.9124	0.00
**Hyperactivity**	Female	MELS 13 Minimum	-115.03 (22.73)	-114.03 (24.64)	0.3284	-0.04
**Hyperactivity**	Female	MELS 13 Maximum	118.20 (24.25)	115.73 (25.99)	0.0222	0.10
**Hyperactivity**	Female	MELS 13 Range	233.23 (41.46)	229.75 (45.49)	0.0654	0.08
**Hyperactivity**	Female	MELS 13 Mean	0.00 (0.30)	-0.00 (0.46)	0.7152	0.02
**Hyperactivity**	Female	MELS 13 Std	33.32 (3.43)	33.05 (3.72)	0.0766	0.08
**Hyperactivity**	Female	MELS 13 Skewness	0.02 (0.14)	0.01 (0.16)	0.5616	0.03
**Hyperactivity**	Female	MELS 13 Kurtosis	0.37 (0.34)	0.39 (0.39)	0.4930	-0.03
**Hyperactivity**	Female	MELS 14 Minimum	-7.05 (3.82)	-6.93 (3.79)	0.4373	-0.03
**Hyperactivity**	Female	MELS 14 Maximum	8.30 (5.45)	8.23 (5.76)	0.7718	0.01
**Hyperactivity**	Female	MELS 14 Range	15.36 (8.94)	15.16 (9.27)	0.6171	0.02
**Hyperactivity**	Female	MELS 14 Mean	-0.01 (0.01)	-0.01 (0.02)	0.0383	0.10
**Hyperactivity**	Female	MELS 14 Std	1.56 (0.28)	1.54 (0.32)	0.1424	0.06
**Hyperactivity**	Female	MELS 14 Skewness	0.66 (0.57)	0.69 (0.63)	0.2139	-0.05
**Hyperactivity**	Female	MELS 14 Kurtosis	5.52 (15.30)	5.70 (17.37)	0.7967	-0.01
**Hyperactivity**	Female	MELS 15 Minimum	-230.26 (61.18)	-224.97 (63.40)	0.0463	-0.08
**Hyperactivity**	Female	MELS 15 Maximum	341.66 (85.85)	331.47 (91.92)	0.0077	0.11
**Hyperactivity**	Female	MELS 15 Range	571.92 (133.93)	556.45 (143.70)	0.0097	0.11
**Hyperactivity**	Female	MELS 15 Mean	-0.01 (0.57)	0.01 (0.81)	0.4662	-0.03
**Hyperactivity**	Female	MELS 15 Std	64.09 (10.86)	63.27 (11.55)	0.0877	0.07
**Hyperactivity**	Female	MELS 15 Skewness	0.87 (0.32)	0.84 (0.35)	0.0414	0.09
**Hyperactivity**	Female	MELS 15 Kurtosis	3.37 (1.57)	3.26 (1.66)	0.0863	0.07
**Hyperactivity**	Female	MELS 16 Minimum	-256.26 (68.51)	-251.38 (68.79)	0.0916	-0.07
**Hyperactivity**	Female	MELS 16 Maximum	236.45 (58.53)	232.20 (61.56)	0.0982	0.07
**Hyperactivity**	Female	MELS 16 Range	492.71 (112.62)	483.58 (117.07)	0.0622	0.08
**Hyperactivity**	Female	MELS 16 Mean	-0.12 (0.68)	-0.13 (1.08)	0.7818	0.01
**Hyperactivity**	Female	MELS 16 Std	61.10 (7.12)	60.61 (7.93)	0.1376	0.06
**Hyperactivity**	Female	MELS 16 Skewness	-0.15 (0.23)	-0.14 (0.23)	0.8925	-0.01
**Hyperactivity**	Female	MELS 16 Kurtosis	1.13 (0.85)	1.10 (0.91)	0.4771	0.03
**Hyperactivity**	Female	MELS 17 Minimum	-293.75 (69.27)	-287.33 (73.55)	0.0361	-0.09
**Hyperactivity**	Female	MELS 17 Maximum	288.57 (69.12)	279.92 (71.91)	0.0040	0.12

**Hyperactivity**	Female	MELS 17 Range	582.32 (123.13)	567.25 (130.90)	0.0057	0.12
**Hyperactivity**	Female	MELS 17 Mean	-0.10 (0.60)	-0.15 (1.27)	0.3159	0.05
**Hyperactivity**	Female	MELS 17 Std	73.18 (7.98)	72.23 (8.49)	0.0070	0.12
**Hyperactivity**	Female	MELS 17 Skewness	-0.07 (0.17)	-0.08 (0.19)	0.1459	0.06
**Hyperactivity**	Female	MELS 17 Kurtosis	1.02 (0.61)	1.00 (0.73)	0.3943	0.04
**Hyperactivity**	Female	MELS 18 Minimum	-272.91 (60.37)	-266.30 (64.31)	0.0136	-0.11
**Hyperactivity**	Female	MELS 18 Maximum	268.98 (61.97)	262.97 (64.74)	0.0262	0.09
**Hyperactivity**	Female	MELS 18 Range	541.89 (105.61)	529.26 (113.86)	0.0077	0.11
**Hyperactivity**	Female	MELS 18 Mean	-0.03 (0.85)	0.00 (1.10)	0.5560	-0.03
**Hyperactivity**	Female	MELS 18 Std	71.94 (6.87)	70.71 (6.96)	0.0000	0.18
**Hyperactivity**	Female	MELS 18 Skewness	-0.04 (0.15)	-0.03 (0.16)	0.4270	-0.03
**Hyperactivity**	Female	MELS 18 Kurtosis	0.66 (0.53)	0.66 (0.56)	0.8273	0.01
**Hyperactivity**	Female	MELS 19 Minimum	-284.68 (63.50)	-279.96 (67.58)	0.0937	-0.07
**Hyperactivity**	Female	MELS 19 Maximum	281.74 (60.51)	278.87 (65.04)	0.2875	0.05
**Hyperactivity**	Female	MELS 19 Range	566.42 (106.70)	558.83 (117.57)	0.1189	0.07
**Hyperactivity**	Female	MELS 19 Mean	-0.00 (0.89)	-0.00 (1.19)	0.9398	0.00
**Hyperactivity**	Female	MELS 19 Std	75.42 (6.35)	75.04 (7.10)	0.1961	0.06
**Hyperactivity**	Female	MELS 19 Skewness	-0.00 (0.15)	0.00 (0.16)	0.2390	-0.05
**Hyperactivity**	Female	MELS 19 Kurtosis	0.63 (0.55)	0.63 (0.62)	0.9159	0.00
**Hyperactivity**	Female	MELS 20 Minimum	-299.48 (65.16)	-295.79 (69.22)	0.2004	-0.05
**Hyperactivity**	Female	MELS 20 Maximum	318.96 (70.69)	313.45 (77.33)	0.0859	0.07
**Hyperactivity**	Female	MELS 20 Range	618.44 (120.81)	609.24 (131.21)	0.0912	0.07
**Hyperactivity**	Female	MELS 20 Mean	-0.02 (0.68)	0.01 (1.12)	0.3857	-0.04
**Hyperactivity**	Female	MELS 20 Std	81.88 (6.85)	81.22 (7.57)	0.0351	0.09
**Hyperactivity**	Female	MELS 20 Skewness	0.07 (0.15)	0.07 (0.15)	0.8547	-0.01
**Hyperactivity**	Female	MELS 20 Kurtosis	0.70 (0.60)	0.69 (0.72)	0.6912	0.02
**Hyperactivity**	Female	MELS 21 Minimum	-305.94 (67.08)	-299.26 (68.62)	0.0203	-0.10
**Hyperactivity**	Female	MELS 21 Maximum	311.17 (71.34)	303.41 (71.26)	0.0097	0.11
**Hyperactivity**	Female	MELS 21 Range	617.11 (121.35)	602.67 (124.97)	0.0058	0.12
**Hyperactivity**	Female	MELS 21 Mean	0.07 (0.86)	0.05 (1.14)	0.6895	0.02
**Hyperactivity**	Female	MELS 21 Std	82.45 (6.67)	81.65 (7.34)	0.0084	0.11
**Hyperactivity**	Female	MELS 21 Skewness	0.02 (0.14)	0.02 (0.15)	0.4518	0.03
**Hyperactivity**	Female	MELS 21 Kurtosis	0.64 (0.50)	0.61 (0.69)	0.2897	0.05
**Hyperactivity**	Female	MELS 22 Minimum	-297.56 (61.34)	-288.65 (63.26)	0.0008	-0.14
**Hyperactivity**	Female	MELS 22 Maximum	287.65 (54.89)	280.85 (56.61)	0.0041	0.12
**Hyperactivity**	Female	MELS 22 Range	585.21 (101.19)	569.51 (105.70)	0.0004	0.15
**Hyperactivity**	Female	MELS 22 Mean	0.03 (0.72)	0.06 (1.12)	0.4560	-0.04
**Hyperactivity**	Female	MELS 22 Std	80.43 (5.57)	79.52 (6.17)	0.0004	0.15
**Hyperactivity**	Female	MELS 22 Skewness	-0.02 (0.13)	-0.02 (0.13)	0.9095	0.00
**Hyperactivity**	Female	MELS 22 Kurtosis	0.49 (0.43)	0.46 (0.44)	0.1258	0.06

**Hyperactivity**	Female	MELS 23 Minimum	-280.05 (55.24)	-271.89 (55.68)	0.0005	-0.15
**Hyperactivity**	Female	MELS 23 Maximum	276.96 (54.42)	271.43 (55.40)	0.0173	0.10
**Hyperactivity**	Female	MELS 23 Range	557.01 (95.10)	543.32 (97.50)	0.0008	0.14
**Hyperactivity**	Female	MELS 23 Mean	0.05 (0.69)	0.05 (1.15)	0.9876	0.00
**Hyperactivity**	Female	MELS 23 Std	77.14 (5.30)	76.55 (5.83)	0.0137	0.11
**Hyperactivity**	Female	MELS 23 Skewness	-0.00 (0.12)	0.00 (0.14)	0.3071	-0.04
**Hyperactivity**	Female	MELS 23 Kurtosis	0.46 (0.46)	0.42 (0.44)	0.0588	0.08
**Hyperactivity**	Female	MELS 24 Minimum	-258.95 (48.30)	-255.87 (52.40)	0.1563	-0.06
**Hyperactivity**	Female	MELS 24 Maximum	260.59 (49.21)	256.79 (52.57)	0.0819	0.07
**Hyperactivity**	Female	MELS 24 Range	519.54 (84.05)	512.65 (92.92)	0.0732	0.08
**Hyperactivity**	Female	MELS 24 Mean	0.09 (0.65)	0.03 (1.21)	0.2681	0.05
**Hyperactivity**	Female	MELS 24 Std	72.99 (4.94)	72.58 (5.61)	0.0766	0.08
**Hyperactivity**	Female	MELS 24 Skewness	0.01 (0.12)	0.01 (0.12)	0.9702	0.00
**Hyperactivity**	Female	MELS 24 Kurtosis	0.42 (0.40)	0.40 (0.53)	0.4663	0.03
**Hyperactivity**	Female	MELS 25 Minimum	-242.14 (42.66)	-238.72 (48.52)	0.0880	-0.07
**Hyperactivity**	Female	MELS 25 Maximum	240.80 (42.96)	236.71 (46.73)	0.0350	0.09
**Hyperactivity**	Female	MELS 25 Range	482.94 (73.21)	475.44 (84.04)	0.0302	0.10
**Hyperactivity**	Female	MELS 25 Mean	0.05 (0.53)	0.04 (0.96)	0.7983	0.01
**Hyperactivity**	Female	MELS 25 Std	68.29 (4.56)	67.68 (5.20)	0.0050	0.12
**Hyperactivity**	Female	MELS 25 Skewness	0.00 (0.11)	-0.01 (0.12)	0.1818	0.06
**Hyperactivity**	Female	MELS 25 Kurtosis	0.39 (0.38)	0.38 (0.42)	0.3100	0.04
**Hyperactivity**	Female	MELS 26 Minimum	-223.74 (42.47)	-220.44 (43.50)	0.0702	-0.08
**Hyperactivity**	Female	MELS 26 Maximum	220.90 (40.62)	219.04 (42.28)	0.2911	0.05
**Hyperactivity**	Female	MELS 26 Range	444.64 (72.55)	439.48 (75.45)	0.1014	0.07
**Hyperactivity**	Female	MELS 26 Mean	-0.02 (0.50)	0.01 (1.03)	0.4508	-0.04
**Hyperactivity**	Female	MELS 26 Std	62.93 (4.49)	62.82 (4.91)	0.5804	0.02
**Hyperactivity**	Female	MELS 26 Skewness	-0.01 (0.11)	-0.01 (0.13)	0.2352	-0.05
**Hyperactivity**	Female	MELS 26 Kurtosis	0.35 (0.36)	0.37 (0.40)	0.3307	-0.04
**Hyperactivity**	Female	F1 Minimum	211.12 (27.94)	223.04 (89.58)	0.0006	-0.18
**Hyperactivity**	Female	F1 Maximum	4635.26 (831.02)	4631.98 (909.78)	0.9307	0.00
**Hyperactivity**	Female	F1 Range	4424.14 (835.93)	4408.94 (917.08)	0.6891	0.02
**Hyperactivity**	Female	F1 Mean	994.71 (384.46)	1084.03 (473.49)	0.0000	-0.21
**Hyperactivity**	Female	F1 Std	889.45 (227.67)	909.38 (259.55)	0.0624	-0.08
**Hyperactivity**	Female	F1 Skewness	2.08 (1.00)	1.94 (1.10)	0.0020	0.13
**Hyperactivity**	Female	F1 Kurtosis	5.23 (5.57)	4.87 (5.85)	0.1392	0.06
**Hyperactivity**	Female	F2 Minimum	564.19 (354.97)	636.07 (475.49)	0.0002	-0.17
**Hyperactivity**	Female	F2 Maximum	6329.54 (545.44)	6319.18 (547.93)	0.6529	0.02
**Hyperactivity**	Female	F2 Range	5765.35 (702.74)	5683.11 (788.20)	0.0116	0.11
**Hyperactivity**	Female	F2 Mean	2487.21 (561.59)	2600.04 (679.07)	0.0000	-0.18
**Hyperactivity**	Female	F2 Std	1236.98 (204.02)	1246.24 (226.20)	0.3223	-0.04

**Hyperactivity**	Female	F2 Skewness	0.90 (0.56)	0.83 (0.63)	0.0154	0.11
**Hyperactivity**	Female	F2 Kurtosis	0.52 (1.24)	0.48 (1.39)	0.4446	0.03
**Hyperactivity**	Female	F3 Minimum	1788.91 (646.46)	1925.11 (793.41)	0.0000	-0.19
**Hyperactivity**	Female	F3 Maximum	6984.68 (384.24)	6970.12 (402.69)	0.3864	0.04
**Hyperactivity**	Female	F3 Range	5195.77 (879.15)	5045.01 (1041.10)	0.0004	0.16
**Hyperactivity**	Female	F3 Mean	4335.57 (453.68)	4404.48 (532.33)	0.0016	-0.14
**Hyperactivity**	Female	F3 Std	1216.40 (181.93)	1192.29 (217.90)	0.0069	0.12
**Hyperactivity**	Female	F3 Skewness	0.10 (0.53)	0.06 (0.62)	0.1294	0.07
**Hyperactivity**	Female	F3 Kurtosis	-0.60 (0.82)	-0.50 (1.16)	0.0378	-0.10
**Hyperactivity**	Female	F1 1st Derivative Minim	-1939.49 (433.25)	-1886.89 (474.45)	0.0075	-0.12
**Hyperactivity**	Female	F1 1st Derivative Maxin	1948.51 (458.92)	1877.53 (489.70)	0.0005	0.15
**Hyperactivity**	Female	F1 1st Derivative Range	3887.99 (840.15)	3764.42 (912.08)	0.0011	0.14
**Hyperactivity**	Female	F1 1st Derivative Mean	-3.88 (20.68)	-5.83 (26.88)	0.0729	0.08
**Hyperactivity**	Female	F1 1st Derivative Std	497.16 (117.22)	493.55 (132.00)	0.5075	0.03
**Hyperactivity**	Female	F1 1st Derivative Skewı	0.04 (0.42)	0.03 (0.48)	0.3494	0.04
**Hyperactivity**	Female	F1 1st Derivative Kurtos	5.80 (4.03)	5.72 (4.30)	0.6448	0.02
**Hyperactivity**	Female	F2 1st Derivative Minim	-2250.87 (449.69)	-2205.12 (476.81)	0.0213	-0.10
**Hyperactivity**	Female	F2 1st Derivative Maxin	2234.16 (468.52)	2177.56 (503.36)	0.0069	0.12
**Hyperactivity**	Female	F2 1st Derivative Range	4485.03 (846.91)	4382.69 (906.80)	0.0067	0.12
**Hyperactivity**	Female	F2 1st Derivative Mean	-5.60 (28.43)	-8.21 (32.94)	0.0539	0.08
**Hyperactivity**	Female	F2 1st Derivative Std	669.46 (100.44)	660.66 (110.13)	0.0541	0.08
**Hyperactivity**	Female	F2 1st Derivative Skewı	0.02 (0.32)	-0.02 (0.34)	0.0124	0.11
**Hyperactivity**	Female	F2 1st Derivative Kurtos	2.19 (1.27)	2.26 (1.47)	0.2032	-0.06
**Hyperactivity**	Female	F3 1st Derivative Minim	-2035.20 (443.39)	-1972.00 (498.95)	0.0022	-0.13
**Hyperactivity**	Female	F3 1st Derivative Maxin	2056.40 (462.65)	1955.87 (539.92)	0.0000	0.20
**Hyperactivity**	Female	F3 1st Derivative Range	4091.60 (843.19)	3927.87 (985.24)	0.0001	0.18
**Hyperactivity**	Female	F3 1st Derivative Mean	-3.03 (28.99)	-7.77 (38.69)	0.0025	0.14
**Hyperactivity**	Female	F3 1st Derivative Std	699.92 (118.74)	679.82 (139.11)	0.0004	0.16
**Hyperactivity**	Female	F3 1st Derivative Skewı	0.01 (0.35)	-0.02 (0.39)	0.0246	0.10
**Hyperactivity**	Female	F3 1st Derivative Kurtos	1.05 (0.91)	1.07 (1.04)	0.5627	-0.03
**Hyperactivity**	Female	F1 2nd Derivative Minin	-6686.47 (1998.75)	-6470.14 (2108.33)	0.0139	-0.11
**Hyperactivity**	Female	F1 2nd Derivative Maxi	4893.23 (1447.37)	4691.66 (1471.98)	0.0011	0.14
**Hyperactivity**	Female	F1 2nd Derivative Rang	11579.70 (3092.44)	11161.80 (3260.40)	0.0021	0.13
**Hyperactivity**	Female	F1 2nd Derivative Mear	-0.47 (41.48)	-0.25 (44.93)	0.9088	0.00
**Hyperactivity**	Female	F1 2nd Derivative Std	1274.65 (360.00)	1261.29 (390.01)	0.4095	0.04
**Hyperactivity**	Female	F1 2nd Derivative Skew	-0.81 (0.61)	-0.78 (0.66)	0.3866	-0.04
**Hyperactivity**	Female	F1 2nd Derivative Kurtc	8.75 (5.77)	8.65 (6.49)	0.7214	0.02
**Hyperactivity**	Female	F2 2nd Derivative Minin	-7602.29 (1956.19)	-7399.65 (2039.67)	0.0174	-0.10
**Hyperactivity**	Female	F2 2nd Derivative Maxi	6095.96 (1642.18)	5930.54 (1711.46)	0.0207	0.10
**Hyperactivity**	Female	F2 2nd Derivative Rang	13698.25 (3198.27)	13330.19 (3397.56)	0.0093	0.11

**Hyperactivity**	Female	F2 2nd Derivative Mear	2.82 (67.70)	2.15 (60.73)	0.7981	0.01
**Hyperactivity**	Female	F2 2nd Derivative Std	1722.19 (312.29)	1710.37 (336.46)	0.3982	0.04
**Hyperactivity**	Female	F2 2nd Derivative Skew	-0.53 (0.40)	-0.54 (0.42)	0.9105	0.00
**Hyperactivity**	Female	F2 2nd Derivative Kurtc	4.13 (2.27)	4.15 (2.55)	0.8092	-0.01
**Hyperactivity**	Female	F3 2nd Derivative Minin	-6456.34 (1768.93)	-6303.00 (2013.76)	0.0647	-0.08
**Hyperactivity**	Female	F3 2nd Derivative Maxi	6293.41 (1894.76)	6110.16 (2083.98)	0.0338	0.09
**Hyperactivity**	Female	F3 2nd Derivative Rang	12749.76 (3397.39)	12413.17 (3844.44)	0.0338	0.09
**Hyperactivity**	Female	F3 2nd Derivative Mear	1.84 (57.03)	4.78 (68.72)	0.2962	-0.05
**Hyperactivity**	Female	F3 2nd Derivative Std	1785.40 (336.84)	1756.66 (394.11)	0.0757	0.08
**Hyperactivity**	Female	F3 2nd Derivative Skew	-0.17 (0.36)	-0.16 (0.41)	0.7467	-0.01
**Hyperactivity**	Female	F3 2nd Derivative Kurtc	2.19 (1.37)	2.25 (1.52)	0.3610	-0.04
**Emotional Symptoms**	Female	F0 Minimum	58.88 (11.31)	58.41 (13.10)	0.2662	0.04
**Emotional Symptoms**	Female	F0 Maximum	483.20 (2.76)	482.88 (6.42)	0.0770	0.07
**Emotional Symptoms**	Female	F0 Range	424.32 (12.45)	424.47 (16.47)	0.7752	-0.01
**Emotional Symptoms**	Female	F0 Mean	282.79 (16.93)	282.82 (17.07)	0.9551	0.00
**Emotional Symptoms**	Female	F0 Std	113.04 (6.87)	112.55 (7.80)	0.0552	0.07
**Emotional Symptoms**	Female	F0 Skewness	0.12 (0.16)	0.12 (0.19)	0.8549	0.01
**Emotional Symptoms**	Female	F0 Kurtosis	-1.15 (0.25)	-1.13 (0.37)	0.1691	-0.05
**Emotional Symptoms**	Female	F0 de Minimum	-177.56 (17.51)	-176.91 (19.31)	0.3029	-0.04
**Emotional Symptoms**	Female	F0 de Maximum	188.25 (13.17)	187.31 (15.37)	0.0579	0.07
**Emotional Symptoms**	Female	F0 de Range	365.80 (26.32)	364.22 (30.35)	0.1050	0.06
**Emotional Symptoms**	Female	F0 de Mean	0.17 (0.62)	0.21 (0.70)	0.1266	-0.05
**Emotional Symptoms**	Female	F0 de Std	75.86 (12.90)	75.12 (13.87)	0.1054	0.06
**Emotional Symptoms**	Female	F0 de Skewness	0.32 (0.17)	0.32 (0.19)	0.4705	-0.02
**Emotional Symptoms**	Female	F0 de Kurtosis	-0.42 (0.96)	-0.35 (1.07)	0.0304	-0.07
**Emotional Symptoms**	Female	F0 de2 Minimum	-649.57 (57.98)	-646.62 (62.99)	0.1525	-0.05
**Emotional Symptoms**	Female	F0 de2 Maximum	610.95 (76.78)	608.53 (79.02)	0.3579	0.03
**Emotional Symptoms**	Female	F0 de2 Range	1260.53 (123.36)	1255.15 (131.27)	0.2143	0.04
**Emotional Symptoms**	Female	F0 de2 Mean	0.05 (1.24)	0.13 (1.46)	0.0626	-0.06
**Emotional Symptoms**	Female	F0 de2 Std	299.56 (23.53)	299.76 (25.68)	0.8100	-0.01
**Emotional Symptoms**	Female	F0 de2 Skewness	-0.06 (0.05)	-0.06 (0.07)	0.9226	0.00
**Emotional Symptoms**	Female	F0 de2 Kurtosis	-1.09 (0.27)	-1.09 (0.33)	0.7707	-0.01
**Emotional Symptoms**	Female	Energy Minimum	-11.57 (6.30)	-11.45 (6.17)	0.5539	-0.02
**Emotional Symptoms**	Female	Energy Maximum	3.77 (1.22)	3.92 (1.25)	0.0006	-0.12
**Emotional Symptoms**	Female	Energy Range	15.35 (6.69)	15.37 (6.55)	0.9285	0.00
**Emotional Symptoms**	Female	Energy Mean	-2.07 (1.04)	-2.04 (1.13)	0.3836	-0.03
**Emotional Symptoms**	Female	Energy Std	2.62 (0.58)	2.63 (0.58)	0.4367	-0.03
**Emotional Symptoms**	Female	Energy Skewness	-0.77 (0.67)	-0.72 (0.68)	0.0176	-0.08
**Emotional Symptoms**	Female	Energy Kurtosis	1.74 (5.47)	1.65 (5.51)	0.6308	0.02
**Emotional Symptoms**	Female	Energy de Minimum	-3.48 (1.72)	-3.38 (1.60)	0.0776	-0.06

**Emotional Symptoms**	Female	Energy de Maximum	4.52 (2.19)	4.49 (2.14)	0.6944	0.01
**Emotional Symptoms**	Female	Energy de Range	8.00 (3.60)	7.87 (3.41)	0.2816	0.04
**Emotional Symptoms**	Female	Energy de Mean	0.01 (0.03)	0.01 (0.02)	0.3887	-0.03
**Emotional Symptoms**	Female	Energy de Std	1.11 (0.22)	1.10 (0.22)	0.0914	0.06
**Emotional Symptoms**	Female	Energy de Skewness	0.38 (0.48)	0.43 (0.54)	0.0132	-0.08
**Emotional Symptoms**	Female	Energy de Kurtosis	1.73 (3.68)	1.81 (3.84)	0.5089	-0.02
**Emotional Symptoms**	Female	Energy de2 Minimum	-7.01 (3.84)	-6.92 (3.77)	0.4373	-0.03
**Emotional Symptoms**	Female	Energy de2 Maximum	8.36 (5.71)	8.18 (5.71)	0.3550	0.03
**Emotional Symptoms**	Female	Energy de2 Range	15.38 (9.24)	15.10 (9.20)	0.3717	0.03
**Emotional Symptoms**	Female	Energy de2 Mean	-0.01 (0.02)	-0.01 (0.02)	0.1138	0.06
**Emotional Symptoms**	Female	Energy de2 Std	1.56 (0.29)	1.54 (0.32)	0.0095	0.09
**Emotional Symptoms**	Female	Energy de2 Skewness	0.68 (0.62)	0.68 (0.62)	0.9879	0.00
**Emotional Symptoms**	Female	Energy de2 Kurtosis	5.67 (16.49)	5.67 (17.30)	0.9915	0.00
**Emotional Symptoms**	Female	MFCC 1 Minimum	-11.57 (6.30)	-11.45 (6.17)	0.5539	-0.02
**Emotional Symptoms**	Female	MFCC 1 Maximum	3.77 (1.22)	3.92 (1.25)	0.0006	-0.12
**Emotional Symptoms**	Female	MFCC 1 Range	15.35 (6.69)	15.37 (6.55)	0.9285	0.00
**Emotional Symptoms**	Female	MFCC 1 Mean	-2.07 (1.04)	-2.04 (1.13)	0.3836	-0.03
**Emotional Symptoms**	Female	MFCC 1 Std	2.62 (0.58)	2.63 (0.58)	0.4367	-0.03
**Emotional Symptoms**	Female	MFCC 1 Skewness	-0.77 (0.67)	-0.72 (0.68)	0.0176	-0.08
**Emotional Symptoms**	Female	MFCC 1 Kurtosis	1.74 (5.47)	1.65 (5.51)	0.6308	0.02
**Emotional Symptoms**	Female	MFCC 2 Minimum	-482.35 (95.06)	-470.58 (108.62)	0.0008	-0.12
**Emotional Symptoms**	Female	MFCC 2 Maximum	357.46 (57.60)	350.15 (63.57)	0.0004	0.12
**Emotional Symptoms**	Female	MFCC 2 Range	839.81 (140.75)	820.73 (160.23)	0.0002	0.13
**Emotional Symptoms**	Female	MFCC 2 Mean	42.14 (21.70)	41.33 (22.73)	0.2833	0.04
**Emotional Symptoms**	Female	MFCC 2 Std	158.76 (24.90)	154.84 (27.66)	0.0000	0.15
**Emotional Symptoms**	Female	MFCC 2 Skewness	-0.70 (0.26)	-0.68 (0.30)	0.0989	-0.06
**Emotional Symptoms**	Female	MFCC 2 Kurtosis	0.28 (0.47)	0.29 (0.53)	0.4839	-0.02
**Emotional Symptoms**	Female	MFCC 3 Minimum	-396.09 (107.20)	-391.48 (117.24)	0.2288	-0.04
**Emotional Symptoms**	Female	MFCC 3 Maximum	413.04 (73.37)	408.28 (79.12)	0.0669	0.06
**Emotional Symptoms**	Female	MFCC 3 Range	809.13 (163.48)	799.76 (181.12)	0.1119	0.05
**Emotional Symptoms**	Female	MFCC 3 Mean	28.70 (28.36)	29.70 (29.46)	0.3090	-0.03
**Emotional Symptoms**	Female	MFCC 3 Std	148.78 (26.22)	147.06 (29.01)	0.0692	0.06
**Emotional Symptoms**	Female	MFCC 3 Skewness	-0.19 (0.24)	-0.20 (0.25)	0.3195	0.03
**Emotional Symptoms**	Female	MFCC 3 Kurtosis	-0.31 (0.36)	-0.28 (0.36)	0.0296	-0.07
**Emotional Symptoms**	Female	MFCC 4 Minimum	-463.49 (111.31)	-454.31 (116.48)	0.0175	-0.08
**Emotional Symptoms**	Female	MFCC 4 Maximum	549.59 (102.96)	536.29 (114.52)	0.0004	0.12
**Emotional Symptoms**	Female	MFCC 4 Range	1013.08 (190.77)	990.61 (209.92)	0.0010	0.11
**Emotional Symptoms**	Female	MFCC 4 Mean	20.64 (32.81)	19.08 (31.22)	0.1469	0.05
**Emotional Symptoms**	Female	MFCC 4 Std	189.00 (29.30)	184.88 (33.04)	0.0001	0.13
**Emotional Symptoms**	Female	MFCC 4 Skewness	0.17 (0.24)	0.15 (0.25)	0.1628	0.05

**Emotional Symptoms**	Female	MFCC 4 Kurtosis	-0.29 (0.35)	-0.28 (0.37)	0.4316	-0.03
**Emotional Symptoms**	Female	MFCC 5 Minimum	-461.77 (124.82)	-448.16 (132.30)	0.0018	-0.11
**Emotional Symptoms**	Female	MFCC 5 Maximum	402.57 (89.97)	391.46 (93.59)	0.0004	0.12
**Emotional Symptoms**	Female	MFCC 5 Range	864.34 (191.26)	839.62 (202.09)	0.0002	0.13
**Emotional Symptoms**	Female	MFCC 5 Mean	-8.47 (28.53)	-7.39 (30.53)	0.2845	-0.04
**Emotional Symptoms**	Female	MFCC 5 Std	143.88 (25.88)	139.40 (27.07)	0.0000	0.17
**Emotional Symptoms**	Female	MFCC 5 Skewness	-0.15 (0.24)	-0.15 (0.26)	0.9443	0.00
**Emotional Symptoms**	Female	MFCC 5 Kurtosis	-0.00 (0.39)	0.03 (0.45)	0.0354	-0.07
**Emotional Symptoms**	Female	MFCC 6 Minimum	-453.13 (107.92)	-449.47 (117.73)	0.3419	-0.03
**Emotional Symptoms**	Female	MFCC 6 Maximum	404.56 (93.02)	402.03 (97.21)	0.4326	0.03
**Emotional Symptoms**	Female	MFCC 6 Range	857.69 (170.10)	851.50 (186.52)	0.3095	0.03
**Emotional Symptoms**	Female	MFCC 6 Mean	-15.58 (31.23)	-13.59 (32.50)	0.0647	-0.06
**Emotional Symptoms**	Female	MFCC 6 Std	142.05 (21.34)	141.78 (23.41)	0.7287	0.01
**Emotional Symptoms**	Female	MFCC 6 Skewness	-0.14 (0.24)	-0.15 (0.24)	0.2388	0.04
**Emotional Symptoms**	Female	MFCC 6 Kurtosis	0.02 (0.36)	0.02 (0.40)	0.9655	0.00
**Emotional Symptoms**	Female	MFCC 7 Minimum	-522.68 (121.63)	-516.71 (128.09)	0.1591	-0.05
**Emotional Symptoms**	Female	MFCC 7 Maximum	369.83 (90.33)	361.60 (92.27)	0.0076	0.09
**Emotional Symptoms**	Female	MFCC 7 Range	892.51 (181.51)	878.31 (191.58)	0.0250	0.08
**Emotional Symptoms**	Female	MFCC 7 Mean	-52.77 (33.06)	-53.59 (35.30)	0.4802	0.02
**Emotional Symptoms**	Female	MFCC 7 Std	148.74 (22.48)	147.40 (24.57)	0.0949	0.06
**Emotional Symptoms**	Female	MFCC 7 Skewness	-0.20 (0.22)	-0.20 (0.24)	0.3249	0.03
**Emotional Symptoms**	Female	MFCC 7 Kurtosis	-0.03 (0.35)	-0.02 (0.40)	0.5171	-0.02
**Emotional Symptoms**	Female	MFCC 8 Minimum	-508.54 (124.15)	-506.65 (131.24)	0.6623	-0.01
**Emotional Symptoms**	Female	MFCC 8 Maximum	363.38 (85.37)	353.14 (92.36)	0.0007	0.12
**Emotional Symptoms**	Female	MFCC 8 Range	871.92 (178.03)	859.79 (192.30)	0.0545	0.07
**Emotional Symptoms**	Female	MFCC 8 Mean	-58.24 (34.17)	-60.42 (34.82)	0.0610	0.06
**Emotional Symptoms**	Female	MFCC 8 Std	142.95 (20.88)	141.62 (23.29)	0.0782	0.06
**Emotional Symptoms**	Female	MFCC 8 Skewness	-0.13 (0.25)	-0.15 (0.26)	0.0415	0.07
**Emotional Symptoms**	Female	MFCC 8 Kurtosis	0.05 (0.39)	0.04 (0.44)	0.7412	0.01
**Emotional Symptoms**	Female	MFCC 9 Minimum	-439.55 (95.41)	-426.23 (103.15)	0.0001	-0.13
**Emotional Symptoms**	Female	MFCC 9 Maximum	370.67 (85.92)	367.71 (91.64)	0.3281	0.03
**Emotional Symptoms**	Female	MFCC 9 Range	810.22 (151.15)	793.94 (166.85)	0.0028	0.10
**Emotional Symptoms**	Female	MFCC 9 Mean	-32.11 (28.24)	-30.52 (28.79)	0.0977	-0.06
**Emotional Symptoms**	Female	MFCC 9 Std	133.96 (17.35)	132.26 (19.83)	0.0079	0.09
**Emotional Symptoms**	Female	MFCC 9 Skewness	-0.03 (0.23)	-0.02 (0.23)	0.0232	-0.08
**Emotional Symptoms**	Female	MFCC 9 Kurtosis	-0.02 (0.38)	-0.05 (0.39)	0.0501	0.07
**Emotional Symptoms**	Female	MFCC 10 Minimum	-386.83 (88.68)	-379.67 (94.04)	0.0211	-0.08
**Emotional Symptoms**	Female	MFCC 10 Maximum	348.22 (88.72)	343.25 (94.99)	0.1117	0.05
**Emotional Symptoms**	Female	MFCC 10 Range	735.05 (152.19)	722.92 (165.75)	0.0253	0.08
**Emotional Symptoms**	Female	MFCC 10 Mean	-23.46 (25.21)	-22.20 (25.29)	0.1390	-0.05

**Emotional Symptoms**	Female	MFCC 10 Std	119.77 (19.16)	118.40 (21.26)	0.0483	0.07
**Emotional Symptoms**	Female	MFCC 10 Skewness	0.02 (0.23)	0.01 (0.25)	0.5678	0.02
**Emotional Symptoms**	Female	MFCC 10 Kurtosis	0.04 (0.39)	0.04 (0.42)	0.6192	0.02
**Emotional Symptoms**	Female	MFCC 11 Minimum	-354.09 (81.05)	-348.97 (86.98)	0.0732	-0.06
**Emotional Symptoms**	Female	MFCC 11 Maximum	294.25 (83.02)	289.04 (82.21)	0.0611	0.06
**Emotional Symptoms**	Female	MFCC 11 Range	648.34 (134.87)	638.01 (141.62)	0.0277	0.07
**Emotional Symptoms**	Female	MFCC 11 Mean	-25.36 (26.65)	-24.64 (25.60)	0.4137	-0.03
**Emotional Symptoms**	Female	MFCC 11 Std	102.82 (15.20)	101.28 (16.21)	0.0041	0.10
**Emotional Symptoms**	Female	MFCC 11 Skewness	-0.04 (0.24)	-0.04 (0.26)	0.5922	0.02
**Emotional Symptoms**	Female	MFCC 11 Kurtosis	0.14 (0.41)	0.15 (0.44)	0.4502	-0.03
**Emotional Symptoms**	Female	MFCC 12 Minimum	-306.52 (72.70)	-301.29 (76.80)	0.0393	-0.07
**Emotional Symptoms**	Female	MFCC 12 Maximum	269.54 (65.88)	264.48 (69.50)	0.0276	0.07
**Emotional Symptoms**	Female	MFCC 12 Range	576.06 (115.89)	565.77 (124.43)	0.0119	0.09
**Emotional Symptoms**	Female	MFCC 12 Mean	-15.50 (19.08)	-14.66 (20.71)	0.2169	-0.04
**Emotional Symptoms**	Female	MFCC 12 Std	88.62 (11.90)	87.76 (13.20)	0.0464	0.07
**Emotional Symptoms**	Female	MFCC 12 Skewness	-0.04 (0.24)	-0.05 (0.25)	0.7431	0.01
**Emotional Symptoms**	Female	MFCC 12 Kurtosis	0.25 (0.42)	0.24 (0.46)	0.5135	0.02
**Emotional Symptoms**	Female	MFCC 13 Minimum	-254.13 (65.06)	-248.76 (67.59)	0.0169	-0.08
**Emotional Symptoms**	Female	MFCC 13 Maximum	254.30 (62.42)	254.83 (64.52)	0.8060	-0.01
**Emotional Symptoms**	Female	MFCC 13 Range	508.43 (105.87)	503.59 (111.29)	0.1888	0.04
**Emotional Symptoms**	Female	MFCC 13 Mean	-0.39 (17.67)	1.04 (18.85)	0.0206	-0.08
**Emotional Symptoms**	Female	MFCC 13 Std	77.20 (11.16)	76.84 (11.90)	0.3528	0.03
**Emotional Symptoms**	Female	MFCC 13 Skewness	0.02 (0.25)	0.04 (0.25)	0.0468	-0.07
**Emotional Symptoms**	Female	MFCC 13 Kurtosis	0.32 (0.44)	0.31 (0.47)	0.5084	0.02
**Emotional Symptoms**	Female	MELS 1 Minimum	-3.48 (1.72)	-3.38 (1.60)	0.0776	-0.06
**Emotional Symptoms**	Female	MELS 1 Maximum	4.52 (2.19)	4.49 (2.14)	0.6944	0.01
**Emotional Symptoms**	Female	MELS 1 Range	8.00 (3.60)	7.87 (3.41)	0.2816	0.04
**Emotional Symptoms**	Female	MELS 1 Mean	0.01 (0.03)	0.01 (0.02)	0.3887	-0.03
**Emotional Symptoms**	Female	MELS 1 Std	1.11 (0.22)	1.10 (0.22)	0.0914	0.06
**Emotional Symptoms**	Female	MELS 1 Skewness	0.38 (0.48)	0.43 (0.54)	0.0132	-0.08
**Emotional Symptoms**	Female	MELS 1 Kurtosis	1.73 (3.68)	1.81 (3.84)	0.5089	-0.02
**Emotional Symptoms**	Female	MELS 2 Minimum	-212.84 (46.08)	-205.99 (49.21)	0.0000	-0.14
**Emotional Symptoms**	Female	MELS 2 Maximum	197.02 (41.99)	192.16 (44.59)	0.0009	0.11
**Emotional Symptoms**	Female	MELS 2 Range	409.87 (84.11)	398.14 (89.81)	0.0001	0.13
**Emotional Symptoms**	Female	MELS 2 Mean	0.14 (0.88)	0.18 (0.78)	0.1727	-0.05
**Emotional Symptoms**	Female	MELS 2 Std	59.14 (11.23)	57.68 (12.07)	0.0002	0.13
**Emotional Symptoms**	Female	MELS 2 Skewness	-0.11 (0.22)	-0.09 (0.24)	0.1171	-0.05
**Emotional Symptoms**	Female	MELS 2 Kurtosis	1.08 (0.58)	1.09 (0.62)	0.5913	-0.02
**Emotional Symptoms**	Female	MELS 3 Minimum	-175.34 (40.19)	-172.90 (43.94)	0.0895	-0.06
**Emotional Symptoms**	Female	MELS 3 Maximum	166.94 (41.20)	165.85 (43.13)	0.4450	0.03

**Emotional Symptoms**	Female	MELS 3 Range	342.29 (75.23)	338.76 (81.71)	0.1871	0.04
**Emotional Symptoms**	Female	MELS 3 Mean	0.08 (0.66)	0.06 (0.71)	0.5946	0.02
**Emotional Symptoms**	Female	MELS 3 Std	50.45 (8.47)	49.85 (9.15)	0.0460	0.07
**Emotional Symptoms**	Female	MELS 3 Skewness	-0.13 (0.19)	-0.11 (0.19)	0.0146	-0.08
**Emotional Symptoms**	Female	MELS 3 Kurtosis	0.44 (0.42)	0.45 (0.47)	0.6246	-0.02
**Emotional Symptoms**	Female	MELS 4 Minimum	-211.35 (45.03)	-206.36 (47.81)	0.0016	-0.11
**Emotional Symptoms**	Female	MELS 4 Maximum	205.87 (46.58)	200.71 (49.08)	0.0015	0.11
**Emotional Symptoms**	Female	MELS 4 Range	417.21 (82.03)	407.07 (88.20)	0.0005	0.12
**Emotional Symptoms**	Female	MELS 4 Mean	0.01 (0.76)	0.06 (0.80)	0.0352	-0.07
**Emotional Symptoms**	Female	MELS 4 Std	60.84 (7.91)	59.56 (8.92)	0.0000	0.15
**Emotional Symptoms**	Female	MELS 4 Skewness	-0.08 (0.18)	-0.07 (0.20)	0.2289	-0.04
**Emotional Symptoms**	Female	MELS 4 Kurtosis	0.42 (0.47)	0.43 (0.48)	0.4946	-0.02
**Emotional Symptoms**	Female	MELS 5 Minimum	-181.14 (41.90)	-176.00 (43.72)	0.0004	-0.12
**Emotional Symptoms**	Female	MELS 5 Maximum	173.02 (38.39)	168.30 (40.05)	0.0004	0.12
**Emotional Symptoms**	Female	MELS 5 Range	354.16 (73.11)	344.30 (76.88)	0.0001	0.13
**Emotional Symptoms**	Female	MELS 5 Mean	0.04 (0.61)	0.06 (0.61)	0.2742	-0.04
**Emotional Symptoms**	Female	MELS 5 Std	52.01 (7.26)	50.54 (7.72)	0.0000	0.20
**Emotional Symptoms**	Female	MELS 5 Skewness	-0.06 (0.17)	-0.05 (0.18)	0.4989	-0.02
**Emotional Symptoms**	Female	MELS 5 Kurtosis	0.33 (0.39)	0.36 (0.43)	0.0271	-0.08
**Emotional Symptoms**	Female	MELS 6 Minimum	-183.19 (40.03)	-180.66 (43.02)	0.0732	-0.06
**Emotional Symptoms**	Female	MELS 6 Maximum	179.50 (38.81)	179.11 (41.95)	0.7819	0.01
**Emotional Symptoms**	Female	MELS 6 Range	362.69 (70.53)	359.78 (77.10)	0.2474	0.04
**Emotional Symptoms**	Female	MELS 6 Mean	0.02 (0.77)	0.07 (0.75)	0.0455	-0.07
**Emotional Symptoms**	Female	MELS 6 Std	52.28 (6.28)	51.91 (7.10)	0.1054	0.06
**Emotional Symptoms**	Female	MELS 6 Skewness	-0.02 (0.16)	-0.01 (0.18)	0.2440	-0.04
**Emotional Symptoms**	Female	MELS 6 Kurtosis	0.41 (0.39)	0.42 (0.45)	0.3907	-0.03
**Emotional Symptoms**	Female	MELS 7 Minimum	-198.04 (42.58)	-196.49 (47.07)	0.3114	-0.03
**Emotional Symptoms**	Female	MELS 7 Maximum	188.69 (40.95)	186.59 (44.87)	0.1526	0.05
**Emotional Symptoms**	Female	MELS 7 Range	386.73 (76.13)	383.08 (84.58)	0.1847	0.05
**Emotional Symptoms**	Female	MELS 7 Mean	0.04 (0.61)	-0.02 (0.89)	0.0428	0.07
**Emotional Symptoms**	Female	MELS 7 Std	55.63 (7.00)	54.83 (7.69)	0.0014	0.11
**Emotional Symptoms**	Female	MELS 7 Skewness	-0.07 (0.16)	-0.07 (0.17)	0.7888	0.01
**Emotional Symptoms**	Female	MELS 7 Kurtosis	0.45 (0.37)	0.47 (0.42)	0.0878	-0.06
**Emotional Symptoms**	Female	MELS 8 Minimum	-191.59 (43.28)	-189.92 (46.69)	0.2757	-0.04
**Emotional Symptoms**	Female	MELS 8 Maximum	187.35 (41.83)	184.87 (44.54)	0.0906	0.06
**Emotional Symptoms**	Female	MELS 8 Range	378.94 (76.50)	374.78 (83.69)	0.1287	0.05
**Emotional Symptoms**	Female	MELS 8 Mean	-0.01 (0.60)	-0.05 (0.70)	0.0438	0.07
**Emotional Symptoms**	Female	MELS 8 Std	53.74 (6.45)	53.21 (7.52)	0.0282	0.08
**Emotional Symptoms**	Female	MELS 8 Skewness	-0.03 (0.17)	-0.04 (0.18)	0.1473	0.05
**Emotional Symptoms**	Female	MELS 8 Kurtosis	0.48 (0.40)	0.49 (0.45)	0.2815	-0.04

**Emotional Symptoms**	Female	MELS 9 Minimum	-177.94 (38.06)	-176.12 (40.27)	0.1716	-0.05
**Emotional Symptoms**	Female	MELS 9 Maximum	177.92 (37.61)	174.54 (40.14)	0.0105	0.09
**Emotional Symptoms**	Female	MELS 9 Range	355.86 (67.44)	350.65 (72.67)	0.0292	0.07
**Emotional Symptoms**	Female	MELS 9 Mean	-0.03 (0.61)	-0.02 (0.74)	0.6970	-0.01
**Emotional Symptoms**	Female	MELS 9 Std	51.08 (5.43)	50.32 (6.16)	0.0002	0.13
**Emotional Symptoms**	Female	MELS 9 Skewness	0.02 (0.16)	0.01 (0.17)	0.0808	0.06
**Emotional Symptoms**	Female	MELS 9 Kurtosis	0.42 (0.38)	0.43 (0.43)	0.1952	-0.04
**Emotional Symptoms**	Female	MELS 10 Minimum	-163.50 (36.85)	-161.09 (38.49)	0.0584	-0.06
**Emotional Symptoms**	Female	MELS 10 Maximum	160.69 (35.12)	158.50 (36.63)	0.0721	0.06
**Emotional Symptoms**	Female	MELS 10 Range	324.19 (64.60)	319.59 (68.27)	0.0413	0.07
**Emotional Symptoms**	Female	MELS 10 Mean	0.01 (0.68)	0.01 (0.60)	0.8161	0.01
**Emotional Symptoms**	Female	MELS 10 Std	46.26 (5.18)	45.80 (5.81)	0.0144	0.08
**Emotional Symptoms**	Female	MELS 10 Skewness	-0.02 (0.17)	-0.01 (0.17)	0.5049	-0.02
**Emotional Symptoms**	Female	MELS 10 Kurtosis	0.42 (0.38)	0.41 (0.41)	0.4665	0.02
**Emotional Symptoms**	Female	MELS 11 Minimum	-146.94 (31.75)	-143.87 (33.36)	0.0055	-0.09
**Emotional Symptoms**	Female	MELS 11 Maximum	144.99 (30.46)	142.11 (32.82)	0.0075	0.09
**Emotional Symptoms**	Female	MELS 11 Range	291.93 (55.43)	285.98 (60.00)	0.0025	0.10
**Emotional Symptoms**	Female	MELS 11 Mean	-0.02 (0.58)	-0.03 (0.65)	0.5046	0.02
**Emotional Symptoms**	Female	MELS 11 Std	41.72 (4.38)	41.26 (4.91)	0.0039	0.10
**Emotional Symptoms**	Female	MELS 11 Skewness	-0.01 (0.15)	-0.01 (0.16)	0.4550	0.03
**Emotional Symptoms**	Female	MELS 11 Kurtosis	0.40 (0.38)	0.37 (0.40)	0.1134	0.05
**Emotional Symptoms**	Female	MELS 12 Minimum	-129.29 (26.43)	-127.74 (28.72)	0.0999	-0.06
**Emotional Symptoms**	Female	MELS 12 Maximum	130.28 (28.37)	128.22 (30.16)	0.0387	0.07
**Emotional Symptoms**	Female	MELS 12 Range	259.56 (48.87)	255.96 (53.16)	0.0384	0.07
**Emotional Symptoms**	Female	MELS 12 Mean	-0.03 (0.48)	-0.01 (0.53)	0.2815	-0.04
**Emotional Symptoms**	Female	MELS 12 Std	37.23 (3.81)	36.84 (4.26)	0.0047	0.10
**Emotional Symptoms**	Female	MELS 12 Skewness	-0.00 (0.15)	-0.00 (0.16)	0.7898	-0.01
**Emotional Symptoms**	Female	MELS 12 Kurtosis	0.38 (0.35)	0.37 (0.39)	0.7214	0.01
**Emotional Symptoms**	Female	MELS 13 Minimum	-114.84 (23.33)	-113.85 (24.84)	0.2309	-0.04
**Emotional Symptoms**	Female	MELS 13 Maximum	116.43 (24.88)	116.02 (26.15)	0.6363	0.02
**Emotional Symptoms**	Female	MELS 13 Range	231.27 (42.93)	229.88 (45.82)	0.3570	0.03
**Emotional Symptoms**	Female	MELS 13 Mean	-0.00 (0.45)	-0.00 (0.42)	0.9956	0.00
**Emotional Symptoms**	Female	MELS 13 Std	33.24 (3.55)	33.02 (3.73)	0.0795	0.06
**Emotional Symptoms**	Female	MELS 13 Skewness	0.02 (0.15)	0.02 (0.16)	0.9564	0.00
**Emotional Symptoms**	Female	MELS 13 Kurtosis	0.37 (0.35)	0.39 (0.39)	0.2594	-0.04
**Emotional Symptoms**	Female	MELS 14 Minimum	-7.01 (3.84)	-6.92 (3.77)	0.4373	-0.03
**Emotional Symptoms**	Female	MELS 14 Maximum	8.36 (5.71)	8.18 (5.71)	0.3550	0.03
**Emotional Symptoms**	Female	MELS 14 Range	15.38 (9.24)	15.10 (9.20)	0.3717	0.03
**Emotional Symptoms**	Female	MELS 14 Mean	-0.01 (0.02)	-0.01 (0.02)	0.1138	0.06
**Emotional Symptoms**	Female	MELS 14 Std	1.56 (0.29)	1.54 (0.32)	0.0095	0.09

**Emotional Symptoms**	Female	MELS 14 Skewness	0.68 (0.62)	0.68 (0.62)	0.9879	0.00
**Emotional Symptoms**	Female	MELS 14 Kurtosis	5.67 (16.49)	5.67 (17.30)	0.9915	0.00
**Emotional Symptoms**	Female	MELS 15 Minimum	-229.01 (61.11)	-224.21 (64.02)	0.0237	-0.08
**Emotional Symptoms**	Female	MELS 15 Maximum	337.84 (88.54)	330.78 (92.16)	0.0211	0.08
**Emotional Symptoms**	Female	MELS 15 Range	566.85 (137.61)	554.99 (144.39)	0.0132	0.08
**Emotional Symptoms**	Female	MELS 15 Mean	-0.01 (0.61)	0.02 (0.85)	0.1751	-0.05
**Emotional Symptoms**	Female	MELS 15 Std	64.22 (11.12)	62.97 (11.58)	0.0011	0.11
**Emotional Symptoms**	Female	MELS 15 Skewness	0.86 (0.34)	0.84 (0.34)	0.0728	0.06
**Emotional Symptoms**	Female	MELS 15 Kurtosis	3.29 (1.60)	3.27 (1.67)	0.6534	0.02
**Emotional Symptoms**	Female	MELS 16 Minimum	-254.96 (69.38)	-250.75 (68.38)	0.0686	-0.06
**Emotional Symptoms**	Female	MELS 16 Maximum	234.83 (60.19)	231.93 (61.50)	0.1573	0.05
**Emotional Symptoms**	Female	MELS 16 Range	489.80 (116.00)	482.68 (116.45)	0.0690	0.06
**Emotional Symptoms**	Female	MELS 16 Mean	-0.13 (0.86)	-0.13 (1.09)	0.9049	0.00
**Emotional Symptoms**	Female	MELS 16 Std	61.11 (7.53)	60.47 (7.93)	0.0149	0.08
**Emotional Symptoms**	Female	MELS 16 Skewness	-0.14 (0.22)	-0.14 (0.24)	0.8024	0.01
**Emotional Symptoms**	Female	MELS 16 Kurtosis	1.09 (0.84)	1.12 (0.93)	0.2483	-0.04
**Emotional Symptoms**	Female	MELS 17 Minimum	-292.85 (70.33)	-286.06 (74.09)	0.0056	-0.09
**Emotional Symptoms**	Female	MELS 17 Maximum	285.89 (69.52)	279.01 (72.46)	0.0042	0.10
**Emotional Symptoms**	Female	MELS 17 Range	578.75 (125.11)	565.07 (131.88)	0.0017	0.11
**Emotional Symptoms**	Female	MELS 17 Mean	-0.16 (1.14)	-0.13 (1.20)	0.5233	-0.02
**Emotional Symptoms**	Female	MELS 17 Std	73.22 (8.05)	71.94 (8.57)	0.0000	0.15
**Emotional Symptoms**	Female	MELS 17 Skewness	-0.09 (0.18)	-0.08 (0.19)	0.4359	-0.03
**Emotional Symptoms**	Female	MELS 17 Kurtosis	1.01 (0.67)	1.00 (0.74)	0.8096	0.01
**Emotional Symptoms**	Female	MELS 18 Minimum	-272.55 (62.19)	-264.67 (64.31)	0.0002	-0.12
**Emotional Symptoms**	Female	MELS 18 Maximum	265.91 (63.10)	263.01 (64.93)	0.1802	0.05
**Emotional Symptoms**	Female	MELS 18 Range	538.46 (109.85)	527.68 (113.81)	0.0044	0.10
**Emotional Symptoms**	Female	MELS 18 Mean	-0.00 (0.90)	-0.01 (1.14)	0.9541	0.00
**Emotional Symptoms**	Female	MELS 18 Std	71.71 (6.84)	70.49 (6.98)	0.0000	0.18
**Emotional Symptoms**	Female	MELS 18 Skewness	-0.04 (0.16)	-0.03 (0.16)	0.0749	-0.06
**Emotional Symptoms**	Female	MELS 18 Kurtosis	0.65 (0.53)	0.66 (0.57)	0.6971	-0.01
**Emotional Symptoms**	Female	MELS 19 Minimum	-284.66 (66.44)	-278.67 (67.05)	0.0078	-0.09
**Emotional Symptoms**	Female	MELS 19 Maximum	281.99 (62.03)	277.94 (65.43)	0.0614	0.06
**Emotional Symptoms**	Female	MELS 19 Range	566.64 (112.68)	556.61 (117.24)	0.0100	0.09
**Emotional Symptoms**	Female	MELS 19 Mean	0.01 (0.91)	-0.01 (1.25)	0.7544	0.01
**Emotional Symptoms**	Female	MELS 19 Std	75.49 (6.66)	74.89 (7.12)	0.0107	0.09
**Emotional Symptoms**	Female	MELS 19 Skewness	-0.00 (0.15)	0.00 (0.16)	0.3316	-0.03
**Emotional Symptoms**	Female	MELS 19 Kurtosis	0.63 (0.61)	0.62 (0.61)	0.6075	0.02
**Emotional Symptoms**	Female	MELS 20 Minimum	-299.57 (65.56)	-294.72 (70.05)	0.0354	-0.07
**Emotional Symptoms**	Female	MELS 20 Maximum	318.48 (73.81)	312.20 (77.43)	0.0144	0.08
**Emotional Symptoms**	Female	MELS 20 Range	618.04 (123.75)	606.91 (132.35)	0.0106	0.09

**Emotional Symptoms**	Female	MELS 20 Mean	-0.00 (0.95)	0.01 (1.10)	0.6361	-0.02
**Emotional Symptoms**	Female	MELS 20 Std	81.93 (7.01)	81.01 (7.66)	0.0002	0.13
**Emotional Symptoms**	Female	MELS 20 Skewness	0.07 (0.15)	0.07 (0.16)	0.3855	-0.03
**Emotional Symptoms**	Female	MELS 20 Kurtosis	0.70 (0.57)	0.69 (0.77)	0.5863	0.02
**Emotional Symptoms**	Female	MELS 21 Minimum	-302.90 (66.34)	-299.10 (69.48)	0.0989	-0.06
**Emotional Symptoms**	Female	MELS 21 Maximum	307.70 (71.08)	303.19 (71.43)	0.0606	0.06
**Emotional Symptoms**	Female	MELS 21 Range	610.60 (120.92)	602.29 (126.27)	0.0474	0.07
**Emotional Symptoms**	Female	MELS 21 Mean	0.06 (1.15)	0.06 (1.06)	0.9723	0.00
**Emotional Symptoms**	Female	MELS 21 Std	82.18 (6.60)	81.58 (7.55)	0.0139	0.08
**Emotional Symptoms**	Female	MELS 21 Skewness	0.02 (0.14)	0.02 (0.16)	0.4380	-0.03
**Emotional Symptoms**	Female	MELS 21 Kurtosis	0.62 (0.51)	0.61 (0.73)	0.7776	0.01
**Emotional Symptoms**	Female	MELS 22 Minimum	-293.36 (61.90)	-288.52 (63.55)	0.0227	-0.08
**Emotional Symptoms**	Female	MELS 22 Maximum	285.47 (52.83)	280.18 (58.15)	0.0053	0.10
**Emotional Symptoms**	Female	MELS 22 Range	578.83 (99.23)	568.71 (108.02)	0.0042	0.10
**Emotional Symptoms**	Female	MELS 22 Mean	0.04 (0.90)	0.07 (1.14)	0.3395	-0.03
**Emotional Symptoms**	Female	MELS 22 Std	80.18 (5.66)	79.41 (6.28)	0.0001	0.13
**Emotional Symptoms**	Female	MELS 22 Skewness	-0.01 (0.13)	-0.02 (0.13)	0.2437	0.04
**Emotional Symptoms**	Female	MELS 22 Kurtosis	0.48 (0.41)	0.46 (0.45)	0.2445	0.04
**Emotional Symptoms**	Female	MELS 23 Minimum	-276.96 (55.17)	-271.35 (55.87)	0.0028	-0.10
**Emotional Symptoms**	Female	MELS 23 Maximum	273.68 (54.40)	271.72 (55.72)	0.2908	0.04
**Emotional Symptoms**	Female	MELS 23 Range	550.64 (94.65)	543.07 (98.51)	0.0207	0.08
**Emotional Symptoms**	Female	MELS 23 Mean	0.05 (1.00)	0.05 (1.12)	0.9299	0.00
**Emotional Symptoms**	Female	MELS 23 Std	76.96 (5.41)	76.48 (5.92)	0.0119	0.09
**Emotional Symptoms**	Female	MELS 23 Skewness	-0.00 (0.13)	0.00 (0.14)	0.0895	-0.06
**Emotional Symptoms**	Female	MELS 23 Kurtosis	0.44 (0.43)	0.43 (0.45)	0.6174	0.02
**Emotional Symptoms**	Female	MELS 24 Minimum	-258.47 (52.73)	-255.28 (51.10)	0.0669	-0.06
**Emotional Symptoms**	Female	MELS 24 Maximum	258.78 (49.50)	256.74 (53.33)	0.2426	0.04
**Emotional Symptoms**	Female	MELS 24 Range	517.25 (89.40)	512.02 (92.49)	0.0890	0.06
**Emotional Symptoms**	Female	MELS 24 Mean	0.06 (1.04)	0.04 (1.18)	0.5997	0.02
**Emotional Symptoms**	Female	MELS 24 Std	72.89 (5.25)	72.53 (5.63)	0.0506	0.07
**Emotional Symptoms**	Female	MELS 24 Skewness	0.01 (0.12)	0.01 (0.12)	0.7793	0.01
**Emotional Symptoms**	Female	MELS 24 Kurtosis	0.41 (0.40)	0.40 (0.56)	0.8145	0.01
**Emotional Symptoms**	Female	MELS 25 Minimum	-241.62 (45.67)	-238.07 (48.50)	0.0265	-0.08
**Emotional Symptoms**	Female	MELS 25 Maximum	238.73 (43.48)	236.73 (47.48)	0.1964	0.04
**Emotional Symptoms**	Female	MELS 25 Range	480.35 (77.21)	474.80 (84.86)	0.0448	0.07
**Emotional Symptoms**	Female	MELS 25 Mean	0.01 (0.81)	0.06 (0.95)	0.1010	-0.06
**Emotional Symptoms**	Female	MELS 25 Std	68.02 (4.91)	67.66 (5.19)	0.0379	0.07
**Emotional Symptoms**	Female	MELS 25 Skewness	-0.00 (0.12)	-0.01 (0.12)	0.7136	0.01
**Emotional Symptoms**	Female	MELS 25 Kurtosis	0.39 (0.37)	0.37 (0.43)	0.1782	0.05
**Emotional Symptoms**	Female	MELS 26 Minimum	-222.70 (43.51)	-220.11 (43.21)	0.0757	-0.06

**Emotional Symptoms**	Female	MELS 26 Maximum	220.07 (41.74)	218.98 (42.14)	0.4424	0.03
**Emotional Symptoms**	Female	MELS 26 Range	442.77 (75.23)	439.09 (74.79)	0.1451	0.05
**Emotional Symptoms**	Female	MELS 26 Mean	-0.02 (0.65)	0.02 (1.09)	0.3138	-0.04
**Emotional Symptoms**	Female	MELS 26 Std	62.94 (4.73)	62.78 (4.89)	0.2999	0.04
**Emotional Symptoms**	Female	MELS 26 Skewness	-0.01 (0.11)	-0.01 (0.13)	0.9245	0.00
**Emotional Symptoms**	Female	MELS 26 Kurtosis	0.35 (0.37)	0.37 (0.41)	0.3844	-0.03
**Emotional Symptoms**	Female	F1 Minimum	217.25 (73.30)	222.93 (86.61)	0.0401	-0.07
**Emotional Symptoms**	Female	F1 Maximum	4626.69 (856.77)	4635.82 (917.31)	0.7620	-0.01
**Emotional Symptoms**	Female	F1 Range	4409.43 (863.18)	4412.89 (924.52)	0.9094	0.00
**Emotional Symptoms**	Female	F1 Mean	1021.37 (422.24)	1093.91 (477.86)	0.0000	-0.16
**Emotional Symptoms**	Female	F1 Std	889.58 (237.48)	914.80 (262.66)	0.0032	-0.10
**Emotional Symptoms**	Female	F1 Skewness	2.04 (1.02)	1.92 (1.11)	0.0014	0.11
**Emotional Symptoms**	Female	F1 Kurtosis	5.13 (5.58)	4.83 (5.91)	0.1243	0.05
**Emotional Symptoms**	Female	F2 Minimum	588.67 (431.52)	642.35 (469.61)	0.0005	-0.12
**Emotional Symptoms**	Female	F2 Maximum	6337.32 (534.87)	6312.01 (554.17)	0.1698	0.05
**Emotional Symptoms**	Female	F2 Range	5748.65 (737.10)	5669.66 (792.70)	0.0024	0.10
**Emotional Symptoms**	Female	F2 Mean	2513.48 (611.72)	2616.64 (684.05)	0.0000	-0.16
**Emotional Symptoms**	Female	F2 Std	1232.14 (206.87)	1251.49 (230.31)	0.0098	-0.09
**Emotional Symptoms**	Female	F2 Skewness	0.90 (0.58)	0.82 (0.64)	0.0002	0.13
**Emotional Symptoms**	Female	F2 Kurtosis	0.57 (1.34)	0.44 (1.37)	0.0061	0.09
**Emotional Symptoms**	Female	F3 Minimum	1829.82 (724.57)	1940.04 (792.80)	0.0000	-0.15
**Emotional Symptoms**	Female	F3 Maximum	6997.37 (385.63)	6959.07 (406.33)	0.0044	0.10
**Emotional Symptoms**	Female	F3 Range	5167.55 (948.46)	5019.03 (1047.35)	0.0000	0.15
**Emotional Symptoms**	Female	F3 Mean	4356.50 (491.13)	4411.91 (534.03)	0.0015	-0.11
**Emotional Symptoms**	Female	F3 Std	1208.50 (197.59)	1190.01 (219.48)	0.0096	0.09
**Emotional Symptoms**	Female	F3 Skewness	0.09 (0.55)	0.06 (0.63)	0.0867	0.06
**Emotional Symptoms**	Female	F3 Kurtosis	-0.57 (0.85)	-0.49 (1.23)	0.0299	-0.08
**Emotional Symptoms**	Female	F1 1st Derivative Minim	-1921.28 (455.34)	-1882.46 (473.97)	0.0137	-0.08
**Emotional Symptoms**	Female	F1 1st Derivative Maxin	1914.32 (469.02)	1876.87 (493.28)	0.0218	0.08
**Emotional Symptoms**	Female	F1 1st Derivative Range	3835.60 (872.84)	3759.34 (914.92)	0.0119	0.09
**Emotional Symptoms**	Female	F1 1st Derivative Mean	-4.54 (21.81)	-6.00 (27.88)	0.0950	0.06
**Emotional Symptoms**	Female	F1 1st Derivative Std	492.15 (120.98)	495.33 (133.95)	0.4661	-0.02
**Emotional Symptoms**	Female	F1 1st Derivative Skewı	0.04 (0.45)	0.02 (0.48)	0.4058	0.03
**Emotional Symptoms**	Female	F1 1st Derivative Kurtos	5.77 (4.02)	5.71 (4.38)	0.6577	0.02
**Emotional Symptoms**	Female	F2 1st Derivative Minim	-2255.00 (450.77)	-2190.20 (482.42)	0.0000	-0.14
**Emotional Symptoms**	Female	F2 1st Derivative Maxin	2235.39 (476.83)	2161.26 (507.09)	0.0000	0.15
**Emotional Symptoms**	Female	F2 1st Derivative Range	4490.40 (853.59)	4351.46 (916.79)	0.0000	0.16
**Emotional Symptoms**	Female	F2 1st Derivative Mean	-6.45 (28.26)	-8.46 (34.17)	0.0642	0.06
**Emotional Symptoms**	Female	F2 1st Derivative Std	666.90 (101.88)	659.65 (111.94)	0.0472	0.07
**Emotional Symptoms**	Female	F2 1st Derivative Skewı	-0.00 (0.33)	-0.02 (0.34)	0.2179	0.04

**Emotional Symptoms**	Female	F2 1st Derivative Kurtos	2.26 (1.35)	2.24 (1.48)	0.6780	0.01
**Emotional Symptoms**	Female	F3 1st Derivative Minim	-2029.02 (468.08)	-1957.98 (500.06)	0.0000	-0.15
**Emotional Symptoms**	Female	F3 1st Derivative Maxin	2017.70 (496.26)	1949.56 (543.80)	0.0001	0.13
**Emotional Symptoms**	Female	F3 1st Derivative Range	4046.72 (907.70)	3907.54 (989.48)	0.0000	0.15
**Emotional Symptoms**	Female	F3 1st Derivative Mean	-4.60 (34.22)	-8.21 (38.67)	0.0039	0.10
**Emotional Symptoms**	Female	F3 1st Derivative Std	693.39 (127.12)	677.88 (140.23)	0.0007	0.12
**Emotional Symptoms**	Female	F3 1st Derivative Skewı	-0.01 (0.34)	-0.02 (0.40)	0.1856	0.05
**Emotional Symptoms**	Female	F3 1st Derivative Kurtos	1.02 (0.90)	1.09 (1.07)	0.0451	-0.07
**Emotional Symptoms**	Female	F1 2nd Derivative Minin	-6579.61 (2054.16)	-6469.64 (2109.82)	0.1182	-0.05
**Emotional Symptoms**	Female	F1 2nd Derivative Maxiı	4784.39 (1441.82)	4696.33 (1483.90)	0.0751	0.06
**Emotional Symptoms**	Female	F1 2nd Derivative Rang	11363.99 (3162.61)	11165.97 (3272.25)	0.0690	0.06
**Emotional Symptoms**	Female	F1 2nd Derivative Mear	1.46 (39.15)	-1.26 (46.93)	0.0677	0.06
**Emotional Symptoms**	Female	F1 2nd Derivative Std	1250.30 (347.84)	1271.09 (403.71)	0.1086	-0.06
**Emotional Symptoms**	Female	F1 2nd Derivative Skew	-0.80 (0.63)	-0.78 (0.66)	0.4428	-0.03
**Emotional Symptoms**	Female	F1 2nd Derivative Kurtc	8.76 (6.09)	8.62 (6.51)	0.4920	0.02
**Emotional Symptoms**	Female	F2 2nd Derivative Minin	-7638.29 (1975.85)	-7323.76 (2045.39)	0.0000	-0.16
**Emotional Symptoms**	Female	F2 2nd Derivative Maxiı	6091.01 (1665.35)	5887.61 (1715.31)	0.0004	0.12
**Emotional Symptoms**	Female	F2 2nd Derivative Rang	13729.30 (3258.04)	13211.38 (3409.98)	0.0000	0.16
**Emotional Symptoms**	Female	F2 2nd Derivative Mear	2.43 (62.24)	2.19 (61.92)	0.9090	0.00
**Emotional Symptoms**	Female	F2 2nd Derivative Std	1721.46 (316.11)	1707.52 (340.84)	0.2128	0.04
**Emotional Symptoms**	Female	F2 2nd Derivative Skew	-0.55 (0.41)	-0.52 (0.42)	0.0326	-0.07
**Emotional Symptoms**	Female	F2 2nd Derivative Kurtc	4.28 (2.40)	4.08 (2.56)	0.0151	0.08
**Emotional Symptoms**	Female	F3 2nd Derivative Minin	-6473.51 (1886.46)	-6251.16 (2015.12)	0.0008	-0.11
**Emotional Symptoms**	Female	F3 2nd Derivative Maxiı	6273.61 (1994.52)	6070.55 (2080.61)	0.0033	0.10
**Emotional Symptoms**	Female	F3 2nd Derivative Rang	12747.12 (3624.80)	12321.71 (3840.65)	0.0008	0.11
**Emotional Symptoms**	Female	F3 2nd Derivative Mear	2.42 (69.17)	5.27 (65.41)	0.2035	-0.04
**Emotional Symptoms**	Female	F3 2nd Derivative Std	1779.91 (364.93)	1751.76 (394.80)	0.0297	0.07
**Emotional Symptoms**	Female	F3 2nd Derivative Skew	-0.17 (0.37)	-0.16 (0.42)	0.5139	-0.02
**Emotional Symptoms**	Female	F3 2nd Derivative Kurtc	2.22 (1.42)	2.25 (1.54)	0.5572	-0.02
**Conduct Problems**	Female	F0 Minimum	58.91 (12.52)	58.36 (12.47)	0.1809	0.04
**Conduct Problems**	Female	F0 Maximum	483.07 (5.46)	482.95 (5.37)	0.4881	0.02
**Conduct Problems**	Female	F0 Range	424.16 (15.51)	424.59 (14.92)	0.3925	-0.03
**Conduct Problems**	Female	F0 Mean	283.80 (16.65)	282.15 (17.23)	0.0032	0.10
**Conduct Problems**	Female	F0 Std	112.63 (7.31)	112.79 (7.60)	0.5220	-0.02
**Conduct Problems**	Female	F0 Skewness	0.12 (0.17)	0.12 (0.19)	0.5283	-0.02
**Conduct Problems**	Female	F0 Kurtosis	-1.15 (0.24)	-1.13 (0.38)	0.0374	-0.07
**Conduct Problems**	Female	F0 de Minimum	-177.20 (18.36)	-177.10 (18.91)	0.8666	-0.01
**Conduct Problems**	Female	F0 de Maximum	187.77 (13.93)	187.56 (15.07)	0.6600	0.01
**Conduct Problems**	Female	F0 de Range	364.98 (28.01)	364.66 (29.62)	0.7412	0.01
**Conduct Problems**	Female	F0 de Mean	0.18 (0.68)	0.21 (0.66)	0.1841	-0.04

**Conduct Problems**	Female	F0 de Std	75.01 (13.18)	75.63 (13.77)	0.1641	-0.05
**Conduct Problems**	Female	F0 de Skewness	0.33 (0.17)	0.32 (0.19)	0.2065	0.04
**Conduct Problems**	Female	F0 de Kurtosis	-0.37 (0.98)	-0.37 (1.07)	0.9467	0.00
**Conduct Problems**	Female	F0 de2 Minimum	-648.31 (60.84)	-647.26 (61.55)	0.6026	-0.02
**Conduct Problems**	Female	F0 de2 Maximum	609.24 (77.56)	609.50 (78.68)	0.9186	0.00
**Conduct Problems**	Female	F0 de2 Range	1257.54 (127.21)	1256.76 (129.41)	0.8525	0.01
**Conduct Problems**	Female	F0 de2 Mean	0.12 (1.47)	0.09 (1.32)	0.5157	0.02
**Conduct Problems**	Female	F0 de2 Std	299.77 (24.02)	299.64 (25.52)	0.8681	0.01
**Conduct Problems**	Female	F0 de2 Skewness	-0.06 (0.05)	-0.06 (0.08)	0.2340	0.04
**Conduct Problems**	Female	F0 de2 Kurtosis	-1.10 (0.26)	-1.08 (0.33)	0.1191	-0.05
**Conduct Problems**	Female	Energy Minimum	-11.52 (6.14)	-11.48 (6.27)	0.8290	-0.01
**Conduct Problems**	Female	Energy Maximum	3.77 (1.26)	3.92 (1.23)	0.0002	-0.12
**Conduct Problems**	Female	Energy Range	15.30 (6.56)	15.40 (6.63)	0.6253	-0.02
**Conduct Problems**	Female	Energy Mean	-2.07 (1.05)	-2.03 (1.14)	0.3467	-0.03
**Conduct Problems**	Female	Energy Std	2.62 (0.57)	2.63 (0.58)	0.4272	-0.03
**Conduct Problems**	Female	Energy Skewness	-0.77 (0.66)	-0.72 (0.69)	0.0279	-0.07
**Conduct Problems**	Female	Energy Kurtosis	1.68 (5.43)	1.68 (5.54)	0.9998	0.00
**Conduct Problems**	Female	Energy de Minimum	-3.43 (1.54)	-3.40 (1.71)	0.7018	-0.01
**Conduct Problems**	Female	Energy de Maximum	4.51 (2.14)	4.50 (2.17)	0.9336	0.00
**Conduct Problems**	Female	Energy de Range	7.93 (3.34)	7.91 (3.58)	0.8162	0.01
**Conduct Problems**	Female	Energy de Mean	0.01 (0.03)	0.01 (0.02)	0.0220	-0.07
**Conduct Problems**	Female	Energy de Std	1.11 (0.21)	1.10 (0.23)	0.2911	0.03
**Conduct Problems**	Female	Energy de Skewness	0.39 (0.50)	0.43 (0.54)	0.0251	-0.07
**Conduct Problems**	Female	Energy de Kurtosis	1.69 (3.60)	1.85 (3.90)	0.1927	-0.04
**Conduct Problems**	Female	Energy de2 Minimum	-6.96 (3.73)	-6.94 (3.84)	0.8647	-0.01
**Conduct Problems**	Female	Energy de2 Maximum	8.29 (5.36)	8.22 (5.93)	0.6744	0.01
**Conduct Problems**	Female	Energy de2 Range	15.26 (8.80)	15.16 (9.48)	0.7410	0.01
**Conduct Problems**	Female	Energy de2 Mean	-0.01 (0.02)	-0.01 (0.02)	0.0690	0.06
**Conduct Problems**	Female	Energy de2 Std	1.55 (0.29)	1.54 (0.33)	0.2821	0.04
**Conduct Problems**	Female	Energy de2 Skewness	0.68 (0.55)	0.69 (0.67)	0.8829	0.00
**Conduct Problems**	Female	Energy de2 Kurtosis	5.35 (15.07)	5.88 (18.20)	0.3493	-0.03
**Conduct Problems**	Female	MFCC 1 Minimum	-11.52 (6.14)	-11.48 (6.27)	0.8290	-0.01
**Conduct Problems**	Female	MFCC 1 Maximum	3.77 (1.26)	3.92 (1.23)	0.0002	-0.12
**Conduct Problems**	Female	MFCC 1 Range	15.30 (6.56)	15.40 (6.63)	0.6253	-0.02
**Conduct Problems**	Female	MFCC 1 Mean	-2.07 (1.05)	-2.03 (1.14)	0.3467	-0.03
**Conduct Problems**	Female	MFCC 1 Std	2.62 (0.57)	2.63 (0.58)	0.4272	-0.03
**Conduct Problems**	Female	MFCC 1 Skewness	-0.77 (0.66)	-0.72 (0.69)	0.0279	-0.07
**Conduct Problems**	Female	MFCC 1 Kurtosis	1.68 (5.43)	1.68 (5.54)	0.9998	0.00
**Conduct Problems**	Female	MFCC 2 Minimum	-481.79 (96.24)	-470.10 (108.84)	0.0006	-0.11
**Conduct Problems**	Female	MFCC 2 Maximum	355.52 (59.39)	350.91 (62.97)	0.0231	0.08

**Conduct Problems**	Female	MFCC 2 Range	837.31 (143.63)	821.01 (159.96)	0.0013	0.11
**Conduct Problems**	Female	MFCC 2 Mean	41.37 (21.71)	41.79 (22.80)	0.5768	-0.02
**Conduct Problems**	Female	MFCC 2 Std	157.55 (25.49)	155.36 (27.56)	0.0129	0.08
**Conduct Problems**	Female	MFCC 2 Skewness	-0.70 (0.26)	-0.68 (0.30)	0.0677	-0.06
**Conduct Problems**	Female	MFCC 2 Kurtosis	0.29 (0.48)	0.28 (0.53)	0.8279	0.01
**Conduct Problems**	Female	MFCC 3 Minimum	-399.26 (107.67)	-389.03 (117.50)	0.0063	-0.09
**Conduct Problems**	Female	MFCC 3 Maximum	415.26 (74.57)	406.45 (78.63)	0.0005	0.12
**Conduct Problems**	Female	MFCC 3 Range	814.53 (165.81)	795.48 (180.61)	0.0009	0.11
**Conduct Problems**	Female	MFCC 3 Mean	28.29 (28.55)	30.05 (29.41)	0.0670	-0.06
**Conduct Problems**	Female	MFCC 3 Std	149.51 (27.00)	146.45 (28.67)	0.0009	0.11
**Conduct Problems**	Female	MFCC 3 Skewness	-0.20 (0.22)	-0.20 (0.26)	0.4337	-0.03
**Conduct Problems**	Female	MFCC 3 Kurtosis	-0.30 (0.33)	-0.28 (0.38)	0.0654	-0.06
**Conduct Problems**	Female	MFCC 4 Minimum	-463.23 (111.59)	-453.82 (116.66)	0.0127	-0.08
**Conduct Problems**	Female	MFCC 4 Maximum	549.15 (105.59)	535.62 (113.69)	0.0002	0.12
**Conduct Problems**	Female	MFCC 4 Range	1012.38 (194.14)	989.44 (209.14)	0.0006	0.11
**Conduct Problems**	Female	MFCC 4 Mean	19.79 (32.49)	19.53 (31.34)	0.8069	0.01
**Conduct Problems**	Female	MFCC 4 Std	188.38 (30.02)	185.00 (32.90)	0.0013	0.11
**Conduct Problems**	Female	MFCC 4 Skewness	0.17 (0.24)	0.15 (0.25)	0.0284	0.07
**Conduct Problems**	Female	MFCC 4 Kurtosis	-0.29 (0.36)	-0.28 (0.37)	0.7235	-0.01
**Conduct Problems**	Female	MFCC 5 Minimum	-462.66 (126.92)	-446.58 (131.37)	0.0002	-0.12
**Conduct Problems**	Female	MFCC 5 Maximum	402.21 (90.16)	390.90 (93.70)	0.0002	0.12
**Conduct Problems**	Female	MFCC 5 Range	864.87 (193.26)	837.47 (201.42)	0.0000	0.14
**Conduct Problems**	Female	MFCC 5 Mean	-8.76 (29.60)	-7.12 (29.98)	0.0939	-0.06
**Conduct Problems**	Female	MFCC 5 Std	142.94 (25.95)	139.70 (27.18)	0.0002	0.12
**Conduct Problems**	Female	MFCC 5 Skewness	-0.14 (0.23)	-0.15 (0.26)	0.4938	0.02
**Conduct Problems**	Female	MFCC 5 Kurtosis	0.01 (0.41)	0.02 (0.44)	0.8274	-0.01
**Conduct Problems**	Female	MFCC 6 Minimum	-457.09 (109.80)	-446.56 (117.08)	0.0051	-0.09
**Conduct Problems**	Female	MFCC 6 Maximum	409.80 (93.01)	398.36 (97.26)	0.0003	0.12
**Conduct Problems**	Female	MFCC 6 Range	866.89 (172.22)	844.92 (185.89)	0.0002	0.12
**Conduct Problems**	Female	MFCC 6 Mean	-15.59 (32.17)	-13.44 (31.97)	0.0413	-0.07
**Conduct Problems**	Female	MFCC 6 Std	143.00 (21.58)	141.13 (23.38)	0.0119	0.08
**Conduct Problems**	Female	MFCC 6 Skewness	-0.14 (0.23)	-0.16 (0.25)	0.0436	0.07
**Conduct Problems**	Female	MFCC 6 Kurtosis	0.02 (0.37)	0.02 (0.40)	0.6349	0.02
**Conduct Problems**	Female	MFCC 7 Minimum	-526.03 (120.96)	-514.05 (128.80)	0.0038	-0.10
**Conduct Problems**	Female	MFCC 7 Maximum	368.99 (89.43)	361.56 (93.01)	0.0138	0.08
**Conduct Problems**	Female	MFCC 7 Range	895.02 (180.45)	875.61 (192.77)	0.0017	0.10
**Conduct Problems**	Female	MFCC 7 Mean	-54.97 (33.02)	-52.18 (35.45)	0.0140	-0.08
**Conduct Problems**	Female	MFCC 7 Std	149.18 (23.00)	147.01 (24.37)	0.0059	0.09
**Conduct Problems**	Female	MFCC 7 Skewness	-0.20 (0.23)	-0.20 (0.24)	0.2972	0.03
**Conduct Problems**	Female	MFCC 7 Kurtosis	-0.02 (0.35)	-0.02 (0.40)	0.8145	0.01

**Conduct Problems**	Female	MFCC 8 Minimum	-512.01 (124.20)	-504.19 (131.62)	0.0651	-0.06
**Conduct Problems**	Female	MFCC 8 Maximum	362.69 (87.56)	352.86 (91.48)	0.0009	0.11
**Conduct Problems**	Female	MFCC 8 Range	874.70 (180.01)	857.05 (191.89)	0.0042	0.09
**Conduct Problems**	Female	MFCC 8 Mean	-58.83 (33.32)	-60.19 (35.42)	0.2331	0.04
**Conduct Problems**	Female	MFCC 8 Std	143.02 (21.46)	141.48 (23.10)	0.0371	0.07
**Conduct Problems**	Female	MFCC 8 Skewness	-0.14 (0.24)	-0.15 (0.27)	0.7960	0.01
**Conduct Problems**	Female	MFCC 8 Kurtosis	0.05 (0.40)	0.04 (0.44)	0.4882	0.02
**Conduct Problems**	Female	MFCC 9 Minimum	-439.78 (97.79)	-425.11 (102.12)	0.0000	-0.15
**Conduct Problems**	Female	MFCC 9 Maximum	374.74 (88.66)	364.79 (90.09)	0.0007	0.11
**Conduct Problems**	Female	MFCC 9 Range	814.51 (156.55)	789.90 (164.17)	0.0000	0.15
**Conduct Problems**	Female	MFCC 9 Mean	-31.19 (28.82)	-31.02 (28.47)	0.8545	-0.01
**Conduct Problems**	Female	MFCC 9 Std	134.41 (17.96)	131.84 (19.59)	0.0000	0.14
**Conduct Problems**	Female	MFCC 9 Skewness	-0.03 (0.23)	-0.02 (0.23)	0.1113	-0.05
**Conduct Problems**	Female	MFCC 9 Kurtosis	-0.02 (0.38)	-0.05 (0.39)	0.0970	0.05
**Conduct Problems**	Female	MFCC 10 Minimum	-389.91 (88.36)	-377.10 (94.37)	0.0000	-0.14
**Conduct Problems**	Female	MFCC 10 Maximum	349.38 (91.43)	342.12 (93.65)	0.0174	0.08
**Conduct Problems**	Female	MFCC 10 Range	739.30 (155.58)	719.21 (164.27)	0.0002	0.13
**Conduct Problems**	Female	MFCC 10 Mean	-24.43 (25.00)	-21.46 (25.38)	0.0004	-0.12
**Conduct Problems**	Female	MFCC 10 Std	120.29 (19.86)	117.95 (20.94)	0.0005	0.11
**Conduct Problems**	Female	MFCC 10 Skewness	0.01 (0.24)	0.02 (0.24)	0.6203	-0.02
**Conduct Problems**	Female	MFCC 10 Kurtosis	0.05 (0.39)	0.04 (0.42)	0.4822	0.02
**Conduct Problems**	Female	MFCC 11 Minimum	-356.28 (81.18)	-347.14 (87.18)	0.0011	-0.11
**Conduct Problems**	Female	MFCC 11 Maximum	296.23 (82.64)	287.35 (82.28)	0.0011	0.11
**Conduct Problems**	Female	MFCC 11 Range	652.52 (132.93)	634.48 (143.00)	0.0001	0.13
**Conduct Problems**	Female	MFCC 11 Mean	-25.39 (26.50)	-24.57 (25.62)	0.3334	-0.03
**Conduct Problems**	Female	MFCC 11 Std	103.19 (15.24)	100.92 (16.22)	0.0000	0.14
**Conduct Problems**	Female	MFCC 11 Skewness	-0.04 (0.25)	-0.04 (0.25)	0.6619	0.01
**Conduct Problems**	Female	MFCC 11 Kurtosis	0.15 (0.42)	0.14 (0.44)	0.4366	0.03
**Conduct Problems**	Female	MFCC 12 Minimum	-307.42 (72.56)	-300.31 (77.11)	0.0042	-0.09
**Conduct Problems**	Female	MFCC 12 Maximum	269.86 (66.62)	263.91 (69.25)	0.0081	0.09
**Conduct Problems**	Female	MFCC 12 Range	577.27 (116.98)	564.22 (124.24)	0.0011	0.11
**Conduct Problems**	Female	MFCC 12 Mean	-15.39 (19.66)	-14.68 (20.46)	0.2892	-0.04
**Conduct Problems**	Female	MFCC 12 Std	88.59 (12.07)	87.72 (13.20)	0.0389	0.07
**Conduct Problems**	Female	MFCC 12 Skewness	-0.04 (0.23)	-0.05 (0.25)	0.6885	0.01
**Conduct Problems**	Female	MFCC 12 Kurtosis	0.25 (0.42)	0.24 (0.46)	0.4316	0.03
**Conduct Problems**	Female	MFCC 13 Minimum	-254.63 (65.04)	-248.03 (67.73)	0.0026	-0.10
**Conduct Problems**	Female	MFCC 13 Maximum	257.58 (62.31)	252.68 (64.67)	0.0196	0.08
**Conduct Problems**	Female	MFCC 13 Range	512.22 (106.40)	500.71 (111.14)	0.0014	0.11
**Conduct Problems**	Female	MFCC 13 Mean	-0.09 (18.12)	0.95 (18.66)	0.0876	-0.06
**Conduct Problems**	Female	MFCC 13 Std	77.65 (11.45)	76.51 (11.75)	0.0030	0.10

**Conduct Problems**	Female	MFCC 13 Skewness	0.02 (0.24)	0.04 (0.26)	0.1307	-0.05
**Conduct Problems**	Female	MFCC 13 Kurtosis	0.31 (0.43)	0.31 (0.47)	0.6833	0.01
**Conduct Problems**	Female	MELS 1 Minimum	-3.43 (1.54)	-3.40 (1.71)	0.7018	-0.01
**Conduct Problems**	Female	MELS 1 Maximum	4.51 (2.14)	4.50 (2.17)	0.9336	0.00
**Conduct Problems**	Female	MELS 1 Range	7.93 (3.34)	7.91 (3.58)	0.8162	0.01
**Conduct Problems**	Female	MELS 1 Mean	0.01 (0.03)	0.01 (0.02)	0.0220	-0.07
**Conduct Problems**	Female	MELS 1 Std	1.11 (0.21)	1.10 (0.23)	0.2911	0.03
**Conduct Problems**	Female	MELS 1 Skewness	0.39 (0.50)	0.43 (0.54)	0.0251	-0.07
**Conduct Problems**	Female	MELS 1 Kurtosis	1.69 (3.60)	1.85 (3.90)	0.1927	-0.04
**Conduct Problems**	Female	MELS 2 Minimum	-210.99 (45.90)	-206.73 (49.65)	0.0072	-0.09
**Conduct Problems**	Female	MELS 2 Maximum	195.75 (41.42)	192.65 (45.19)	0.0317	0.07
**Conduct Problems**	Female	MELS 2 Range	406.74 (83.42)	399.38 (90.80)	0.0111	0.08
**Conduct Problems**	Female	MELS 2 Mean	0.13 (0.76)	0.19 (0.85)	0.0267	-0.07
**Conduct Problems**	Female	MELS 2 Std	58.55 (11.37)	57.97 (12.06)	0.1377	0.05
**Conduct Problems**	Female	MELS 2 Skewness	-0.10 (0.22)	-0.10 (0.24)	0.4708	-0.02
**Conduct Problems**	Female	MELS 2 Kurtosis	1.10 (0.61)	1.08 (0.60)	0.5430	0.02
**Conduct Problems**	Female	MELS 3 Minimum	-176.45 (40.74)	-171.99 (43.79)	0.0015	-0.11
**Conduct Problems**	Female	MELS 3 Maximum	168.48 (41.19)	164.75 (43.22)	0.0076	0.09
**Conduct Problems**	Female	MELS 3 Range	344.93 (75.70)	336.74 (81.74)	0.0017	0.10
**Conduct Problems**	Female	MELS 3 Mean	0.05 (0.66)	0.08 (0.71)	0.2484	-0.04
**Conduct Problems**	Female	MELS 3 Std	50.54 (8.54)	49.75 (9.15)	0.0076	0.09
**Conduct Problems**	Female	MELS 3 Skewness	-0.12 (0.19)	-0.11 (0.19)	0.1217	-0.05
**Conduct Problems**	Female	MELS 3 Kurtosis	0.45 (0.42)	0.44 (0.48)	0.5346	0.02
**Conduct Problems**	Female	MELS 4 Minimum	-210.58 (45.32)	-206.51 (47.86)	0.0084	-0.09
**Conduct Problems**	Female	MELS 4 Maximum	205.45 (46.75)	200.62 (49.16)	0.0024	0.10
**Conduct Problems**	Female	MELS 4 Range	416.02 (82.69)	407.13 (88.26)	0.0017	0.10
**Conduct Problems**	Female	MELS 4 Mean	0.01 (0.76)	0.06 (0.80)	0.0591	-0.06
**Conduct Problems**	Female	MELS 4 Std	60.52 (8.00)	59.68 (8.96)	0.0028	0.10
**Conduct Problems**	Female	MELS 4 Skewness	-0.07 (0.19)	-0.08 (0.20)	0.1633	0.05
**Conduct Problems**	Female	MELS 4 Kurtosis	0.42 (0.48)	0.43 (0.47)	0.7647	-0.01
**Conduct Problems**	Female	MELS 5 Minimum	-181.32 (42.57)	-175.51 (43.38)	0.0000	-0.14
**Conduct Problems**	Female	MELS 5 Maximum	172.46 (38.14)	168.33 (40.35)	0.0015	0.11
**Conduct Problems**	Female	MELS 5 Range	353.78 (73.25)	343.84 (77.04)	0.0001	0.13
**Conduct Problems**	Female	MELS 5 Mean	0.05 (0.56)	0.06 (0.63)	0.6806	-0.01
**Conduct Problems**	Female	MELS 5 Std	51.67 (7.18)	50.66 (7.83)	0.0000	0.14
**Conduct Problems**	Female	MELS 5 Skewness	-0.06 (0.18)	-0.05 (0.18)	0.1582	-0.05
**Conduct Problems**	Female	MELS 5 Kurtosis	0.35 (0.40)	0.35 (0.42)	0.9886	0.00
**Conduct Problems**	Female	MELS 6 Minimum	-184.03 (40.81)	-179.92 (42.69)	0.0029	-0.10
**Conduct Problems**	Female	MELS 6 Maximum	181.48 (39.45)	177.76 (41.70)	0.0057	0.09
**Conduct Problems**	Female	MELS 6 Range	365.51 (71.76)	357.68 (76.66)	0.0015	0.11

**Conduct Problems**	Female	MELS 6 Mean	0.05 (0.64)	0.06 (0.82)	0.6724	-0.01
**Conduct Problems**	Female	MELS 6 Std	52.48 (6.39)	51.75 (7.08)	0.0011	0.11
**Conduct Problems**	Female	MELS 6 Skewness	-0.01 (0.16)	-0.01 (0.18)	0.8553	-0.01
**Conduct Problems**	Female	MELS 6 Kurtosis	0.41 (0.42)	0.42 (0.43)	0.5491	-0.02
**Conduct Problems**	Female	MELS 7 Minimum	-199.24 (43.02)	-195.58 (47.06)	0.0146	-0.08
**Conduct Problems**	Female	MELS 7 Maximum	189.54 (41.70)	185.87 (44.64)	0.0105	0.08
**Conduct Problems**	Female	MELS 7 Range	388.78 (76.97)	381.45 (84.55)	0.0064	0.09
**Conduct Problems**	Female	MELS 7 Mean	-0.02 (0.91)	0.02 (0.72)	0.1803	-0.04
**Conduct Problems**	Female	MELS 7 Std	55.65 (7.03)	54.76 (7.71)	0.0003	0.12
**Conduct Problems**	Female	MELS 7 Skewness	-0.08 (0.15)	-0.07 (0.18)	0.3987	-0.03
**Conduct Problems**	Female	MELS 7 Kurtosis	0.46 (0.37)	0.47 (0.43)	0.3834	-0.03
**Conduct Problems**	Female	MELS 8 Minimum	-192.06 (44.07)	-189.48 (46.42)	0.0846	-0.06
**Conduct Problems**	Female	MELS 8 Maximum	187.05 (41.92)	184.89 (44.69)	0.1325	0.05
**Conduct Problems**	Female	MELS 8 Range	379.11 (77.58)	374.37 (83.51)	0.0760	0.06
**Conduct Problems**	Female	MELS 8 Mean	-0.02 (0.56)	-0.05 (0.73)	0.1463	0.05
**Conduct Problems**	Female	MELS 8 Std	53.73 (6.69)	53.17 (7.45)	0.0175	0.08
**Conduct Problems**	Female	MELS 8 Skewness	-0.03 (0.17)	-0.04 (0.19)	0.3692	0.03
**Conduct Problems**	Female	MELS 8 Kurtosis	0.48 (0.41)	0.49 (0.45)	0.2570	-0.04
**Conduct Problems**	Female	MELS 9 Minimum	-179.13 (38.78)	-175.19 (39.90)	0.0025	-0.10
**Conduct Problems**	Female	MELS 9 Maximum	178.91 (38.13)	173.63 (39.91)	0.0000	0.14
**Conduct Problems**	Female	MELS 9 Range	358.04 (68.18)	348.82 (72.41)	0.0001	0.13
**Conduct Problems**	Female	MELS 9 Mean	-0.02 (0.66)	-0.02 (0.72)	0.9399	0.00
**Conduct Problems**	Female	MELS 9 Std	51.11 (5.52)	50.25 (6.15)	0.0000	0.15
**Conduct Problems**	Female	MELS 9 Skewness	0.01 (0.17)	0.01 (0.16)	0.1043	0.05
**Conduct Problems**	Female	MELS 9 Kurtosis	0.44 (0.43)	0.42 (0.40)	0.2424	0.04
**Conduct Problems**	Female	MELS 10 Minimum	-163.62 (36.95)	-160.83 (38.53)	0.0254	-0.07
**Conduct Problems**	Female	MELS 10 Maximum	161.46 (34.62)	157.83 (37.01)	0.0023	0.10
**Conduct Problems**	Female	MELS 10 Range	325.08 (64.04)	318.67 (68.82)	0.0036	0.10
**Conduct Problems**	Female	MELS 10 Mean	0.03 (0.66)	-0.01 (0.60)	0.0538	0.06
**Conduct Problems**	Female	MELS 10 Std	46.34 (5.28)	45.71 (5.79)	0.0006	0.11
**Conduct Problems**	Female	MELS 10 Skewness	-0.01 (0.17)	-0.02 (0.17)	0.4928	0.02
**Conduct Problems**	Female	MELS 10 Kurtosis	0.42 (0.36)	0.41 (0.42)	0.8370	0.01
**Conduct Problems**	Female	MELS 11 Minimum	-146.98 (32.12)	-143.62 (33.23)	0.0019	-0.10
**Conduct Problems**	Female	MELS 11 Maximum	145.29 (30.15)	141.70 (33.14)	0.0007	0.11
**Conduct Problems**	Female	MELS 11 Range	292.27 (55.35)	285.32 (60.32)	0.0003	0.12
**Conduct Problems**	Female	MELS 11 Mean	-0.04 (0.55)	-0.02 (0.67)	0.4224	-0.03
**Conduct Problems**	Female	MELS 11 Std	41.87 (4.40)	41.13 (4.93)	0.0000	0.16
**Conduct Problems**	Female	MELS 11 Skewness	-0.01 (0.15)	-0.01 (0.16)	0.4544	0.02
**Conduct Problems**	Female	MELS 11 Kurtosis	0.39 (0.39)	0.38 (0.39)	0.5091	0.02
**Conduct Problems**	Female	MELS 12 Minimum	-129.39 (26.60)	-127.56 (28.78)	0.0460	-0.07

**Conduct Problems**	Female	MELS 12 Maximum	130.05 (28.88)	128.22 (29.96)	0.0590	0.06
**Conduct Problems**	Female	MELS 12 Range	259.45 (49.34)	255.78 (53.16)	0.0309	0.07
**Conduct Problems**	Female	MELS 12 Mean	-0.04 (0.55)	-0.01 (0.48)	0.0830	-0.06
**Conduct Problems**	Female	MELS 12 Std	37.19 (3.91)	36.84 (4.23)	0.0094	0.09
**Conduct Problems**	Female	MELS 12 Skewness	-0.01 (0.15)	-0.00 (0.16)	0.5058	-0.02
**Conduct Problems**	Female	MELS 12 Kurtosis	0.38 (0.35)	0.37 (0.39)	0.7186	0.01
**Conduct Problems**	Female	MELS 13 Minimum	-115.70 (23.12)	-113.21 (25.03)	0.0019	-0.10
**Conduct Problems**	Female	MELS 13 Maximum	117.07 (24.87)	115.57 (26.23)	0.0766	0.06
**Conduct Problems**	Female	MELS 13 Range	232.77 (42.77)	228.78 (46.07)	0.0069	0.09
**Conduct Problems**	Female	MELS 13 Mean	0.01 (0.39)	-0.00 (0.46)	0.5063	0.02
**Conduct Problems**	Female	MELS 13 Std	33.32 (3.56)	32.95 (3.74)	0.0021	0.10
**Conduct Problems**	Female	MELS 13 Skewness	0.01 (0.14)	0.02 (0.16)	0.1415	-0.05
**Conduct Problems**	Female	MELS 13 Kurtosis	0.38 (0.35)	0.39 (0.40)	0.3629	-0.03
**Conduct Problems**	Female	MELS 14 Minimum	-6.96 (3.73)	-6.94 (3.84)	0.8647	-0.01
**Conduct Problems**	Female	MELS 14 Maximum	8.29 (5.36)	8.22 (5.93)	0.6744	0.01
**Conduct Problems**	Female	MELS 14 Range	15.26 (8.80)	15.16 (9.48)	0.7410	0.01
**Conduct Problems**	Female	MELS 14 Mean	-0.01 (0.02)	-0.01 (0.02)	0.0690	0.06
**Conduct Problems**	Female	MELS 14 Std	1.55 (0.29)	1.54 (0.33)	0.2821	0.04
**Conduct Problems**	Female	MELS 14 Skewness	0.68 (0.55)	0.69 (0.67)	0.8829	0.00
**Conduct Problems**	Female	MELS 14 Kurtosis	5.35 (15.07)	5.88 (18.20)	0.3493	-0.03
**Conduct Problems**	Female	MELS 15 Minimum	-228.35 (61.42)	-224.29 (64.05)	0.0503	-0.06
**Conduct Problems**	Female	MELS 15 Maximum	338.60 (87.81)	329.76 (92.82)	0.0032	0.10
**Conduct Problems**	Female	MELS 15 Range	566.95 (136.49)	554.06 (145.53)	0.0058	0.09
**Conduct Problems**	Female	MELS 15 Mean	-0.02 (0.75)	0.03 (0.79)	0.0683	-0.06
**Conduct Problems**	Female	MELS 15 Std	63.79 (11.11)	63.17 (11.63)	0.0978	0.05
**Conduct Problems**	Female	MELS 15 Skewness	0.85 (0.34)	0.84 (0.34)	0.1565	0.05
**Conduct Problems**	Female	MELS 15 Kurtosis	3.32 (1.65)	3.25 (1.64)	0.2124	0.04
**Conduct Problems**	Female	MELS 16 Minimum	-255.66 (67.92)	-249.98 (69.24)	0.0120	-0.08
**Conduct Problems**	Female	MELS 16 Maximum	234.77 (58.60)	231.76 (62.60)	0.1348	0.05
**Conduct Problems**	Female	MELS 16 Range	490.43 (113.08)	481.74 (118.33)	0.0233	0.08
**Conduct Problems**	Female	MELS 16 Mean	-0.12 (0.97)	-0.14 (1.05)	0.6419	0.02
**Conduct Problems**	Female	MELS 16 Std	61.09 (7.54)	60.44 (7.95)	0.0110	0.08
**Conduct Problems**	Female	MELS 16 Skewness	-0.14 (0.21)	-0.14 (0.25)	0.8606	0.01
**Conduct Problems**	Female	MELS 16 Kurtosis	1.08 (0.75)	1.13 (0.98)	0.0940	-0.06
**Conduct Problems**	Female	MELS 17 Minimum	-292.67 (70.69)	-285.69 (74.11)	0.0036	-0.10
**Conduct Problems**	Female	MELS 17 Maximum	283.72 (69.01)	279.96 (73.08)	0.1097	0.05
**Conduct Problems**	Female	MELS 17 Range	576.39 (124.90)	565.65 (132.58)	0.0118	0.08
**Conduct Problems**	Female	MELS 17 Mean	-0.14 (1.25)	-0.14 (1.13)	0.8409	-0.01
**Conduct Problems**	Female	MELS 17 Std	72.93 (8.13)	72.04 (8.57)	0.0013	0.11
**Conduct Problems**	Female	MELS 17 Skewness	-0.08 (0.18)	-0.08 (0.19)	0.7748	-0.01

**Conduct Problems**	Female	MELS 17 Kurtosis	1.01 (0.72)	1.00 (0.71)	0.5237	0.02
**Conduct Problems**	Female	MELS 18 Minimum	-270.55 (62.59)	-265.43 (64.30)	0.0146	-0.08
**Conduct Problems**	Female	MELS 18 Maximum	266.69 (63.44)	262.28 (64.80)	0.0369	0.07
**Conduct Problems**	Female	MELS 18 Range	537.24 (110.36)	527.71 (113.81)	0.0100	0.09
**Conduct Problems**	Female	MELS 18 Mean	-0.01 (0.90)	-0.00 (1.16)	0.7953	-0.01
**Conduct Problems**	Female	MELS 18 Std	71.37 (6.89)	70.63 (6.99)	0.0011	0.11
**Conduct Problems**	Female	MELS 18 Skewness	-0.03 (0.15)	-0.03 (0.16)	0.6834	-0.01
**Conduct Problems**	Female	MELS 18 Kurtosis	0.65 (0.54)	0.66 (0.57)	0.3231	-0.03
**Conduct Problems**	Female	MELS 19 Minimum	-283.99 (64.88)	-278.68 (68.12)	0.0159	-0.08
**Conduct Problems**	Female	MELS 19 Maximum	282.79 (63.00)	277.11 (65.00)	0.0073	0.09
**Conduct Problems**	Female	MELS 19 Range	566.78 (112.00)	555.79 (117.95)	0.0039	0.10
**Conduct Problems**	Female	MELS 19 Mean	-0.01 (1.09)	0.01 (1.18)	0.6214	-0.02
**Conduct Problems**	Female	MELS 19 Std	75.59 (6.63)	74.78 (7.16)	0.0004	0.12
**Conduct Problems**	Female	MELS 19 Skewness	0.00 (0.15)	0.00 (0.16)	0.7081	-0.01
**Conduct Problems**	Female	MELS 19 Kurtosis	0.62 (0.55)	0.63 (0.65)	0.7093	-0.01
**Conduct Problems**	Female	MELS 20 Minimum	-300.84 (67.58)	-293.52 (68.99)	0.0012	-0.11
**Conduct Problems**	Female	MELS 20 Maximum	318.81 (73.50)	311.52 (77.84)	0.0036	0.10
**Conduct Problems**	Female	MELS 20 Range	619.65 (124.72)	605.04 (132.21)	0.0006	0.11
**Conduct Problems**	Female	MELS 20 Mean	0.01 (0.90)	0.01 (1.14)	0.9139	0.00
**Conduct Problems**	Female	MELS 20 Std	81.92 (7.13)	80.95 (7.62)	0.0001	0.13
**Conduct Problems**	Female	MELS 20 Skewness	0.07 (0.15)	0.07 (0.16)	0.4573	-0.02
**Conduct Problems**	Female	MELS 20 Kurtosis	0.70 (0.59)	0.69 (0.77)	0.7537	0.01
**Conduct Problems**	Female	MELS 21 Minimum	-302.29 (66.47)	-299.24 (69.63)	0.1753	-0.04
**Conduct Problems**	Female	MELS 21 Maximum	307.36 (70.12)	303.09 (72.08)	0.0685	0.06
**Conduct Problems**	Female	MELS 21 Range	609.65 (120.42)	602.32 (126.99)	0.0736	0.06
**Conduct Problems**	Female	MELS 21 Mean	0.06 (1.15)	0.06 (1.05)	0.9264	0.00
**Conduct Problems**	Female	MELS 21 Std	82.20 (6.91)	81.53 (7.43)	0.0050	0.09
**Conduct Problems**	Female	MELS 21 Skewness	0.02 (0.14)	0.02 (0.16)	0.5401	-0.02
**Conduct Problems**	Female	MELS 21 Kurtosis	0.60 (0.52)	0.62 (0.74)	0.3387	-0.03
**Conduct Problems**	Female	MELS 22 Minimum	-294.28 (62.12)	-287.56 (63.45)	0.0012	-0.11
**Conduct Problems**	Female	MELS 22 Maximum	284.30 (54.58)	280.58 (57.48)	0.0452	0.07
**Conduct Problems**	Female	MELS 22 Range	578.58 (101.45)	568.14 (107.24)	0.0026	0.10
**Conduct Problems**	Female	MELS 22 Mean	0.06 (1.08)	0.06 (1.05)	0.9241	0.00
**Conduct Problems**	Female	MELS 22 Std	80.20 (5.73)	79.34 (6.28)	0.0000	0.14
**Conduct Problems**	Female	MELS 22 Skewness	-0.02 (0.13)	-0.02 (0.13)	0.7964	-0.01
**Conduct Problems**	Female	MELS 22 Kurtosis	0.47 (0.42)	0.47 (0.45)	0.8849	0.00
**Conduct Problems**	Female	MELS 23 Minimum	-277.78 (54.55)	-270.40 (56.24)	0.0001	-0.13
**Conduct Problems**	Female	MELS 23 Maximum	275.14 (54.16)	270.60 (55.91)	0.0126	0.08
**Conduct Problems**	Female	MELS 23 Range	552.92 (94.80)	541.00 (98.51)	0.0002	0.12
**Conduct Problems**	Female	MELS 23 Mean	0.08 (1.15)	0.03 (1.03)	0.1706	0.04

**Conduct Problems**	Female	MELS 23 Std	77.07 (5.50)	76.37 (5.89)	0.0002	0.12
**Conduct Problems**	Female	MELS 23 Skewness	-0.00 (0.13)	0.00 (0.14)	0.4344	-0.03
**Conduct Problems**	Female	MELS 23 Kurtosis	0.44 (0.44)	0.42 (0.44)	0.2070	0.04
**Conduct Problems**	Female	MELS 24 Minimum	-259.94 (52.58)	-254.07 (50.99)	0.0006	-0.11
**Conduct Problems**	Female	MELS 24 Maximum	259.38 (50.47)	256.19 (52.96)	0.0628	0.06
**Conduct Problems**	Female	MELS 24 Range	519.32 (90.21)	510.26 (92.06)	0.0026	0.10
**Conduct Problems**	Female	MELS 24 Mean	0.04 (1.08)	0.05 (1.16)	0.7887	-0.01
**Conduct Problems**	Female	MELS 24 Std	73.07 (5.24)	72.38 (5.65)	0.0001	0.13
**Conduct Problems**	Female	MELS 24 Skewness	0.01 (0.12)	0.01 (0.13)	0.6607	-0.01
**Conduct Problems**	Female	MELS 24 Kurtosis	0.41 (0.63)	0.40 (0.41)	0.6357	0.01
**Conduct Problems**	Female	MELS 25 Minimum	-241.38 (45.57)	-237.97 (48.76)	0.0295	-0.07
**Conduct Problems**	Female	MELS 25 Maximum	239.03 (44.00)	236.39 (47.43)	0.0822	0.06
**Conduct Problems**	Female	MELS 25 Range	480.40 (77.88)	474.36 (84.97)	0.0256	0.07
**Conduct Problems**	Female	MELS 25 Mean	0.00 (0.65)	0.06 (1.04)	0.0419	-0.07
**Conduct Problems**	Female	MELS 25 Std	68.05 (4.98)	67.62 (5.16)	0.0099	0.09
**Conduct Problems**	Female	MELS 25 Skewness	-0.00 (0.12)	-0.01 (0.12)	0.6010	0.02
**Conduct Problems**	Female	MELS 25 Kurtosis	0.38 (0.39)	0.37 (0.42)	0.4442	0.03
**Conduct Problems**	Female	MELS 26 Minimum	-223.96 (43.28)	-219.08 (43.26)	0.0006	-0.11
**Conduct Problems**	Female	MELS 26 Maximum	220.47 (40.89)	218.64 (42.70)	0.1840	0.04
**Conduct Problems**	Female	MELS 26 Range	444.43 (73.50)	437.72 (75.81)	0.0065	0.09
**Conduct Problems**	Female	MELS 26 Mean	-0.03 (0.85)	0.03 (1.02)	0.0893	-0.06
**Conduct Problems**	Female	MELS 26 Std	63.07 (4.68)	62.68 (4.93)	0.0148	0.08
**Conduct Problems**	Female	MELS 26 Skewness	-0.01 (0.12)	-0.01 (0.13)	0.2450	-0.04
**Conduct Problems**	Female	MELS 26 Kurtosis	0.36 (0.37)	0.37 (0.41)	0.5520	-0.02
**Conduct Problems**	Female	F1 Minimum	217.68 (75.13)	223.06 (86.47)	0.0468	-0.07
**Conduct Problems**	Female	F1 Maximum	4645.03 (851.36)	4624.26 (924.80)	0.4815	0.02
**Conduct Problems**	Female	F1 Range	4427.35 (857.22)	4401.20 (932.33)	0.3792	0.03
**Conduct Problems**	Female	F1 Mean	1020.42 (426.41)	1099.80 (478.65)	0.0000	-0.18
**Conduct Problems**	Female	F1 Std	885.59 (240.63)	919.29 (262.09)	0.0001	-0.13
**Conduct Problems**	Female	F1 Skewness	2.06 (1.05)	1.90 (1.09)	0.0000	0.16
**Conduct Problems**	Female	F1 Kurtosis	5.39 (6.27)	4.63 (5.44)	0.0001	0.13
**Conduct Problems**	Female	F2 Minimum	588.69 (437.19)	646.23 (468.53)	0.0001	-0.13
**Conduct Problems**	Female	F2 Maximum	6336.77 (530.88)	6310.55 (558.06)	0.1456	0.05
**Conduct Problems**	Female	F2 Range	5748.08 (739.87)	5664.32 (794.60)	0.0010	0.11
**Conduct Problems**	Female	F2 Mean	2520.17 (615.10)	2619.64 (687.07)	0.0000	-0.15
**Conduct Problems**	Female	F2 Std	1228.49 (206.71)	1255.32 (231.70)	0.0002	-0.12
**Conduct Problems**	Female	F2 Skewness	0.88 (0.59)	0.82 (0.64)	0.0013	0.11
**Conduct Problems**	Female	F2 Kurtosis	0.55 (1.33)	0.45 (1.39)	0.0226	0.08
**Conduct Problems**	Female	F3 Minimum	1840.04 (712.88)	1941.21 (804.85)	0.0001	-0.13
**Conduct Problems**	Female	F3 Maximum	7003.86 (387.58)	6951.97 (405.92)	0.0001	0.13

**Conduct Problems**	Female	F3 Range	5163.83 (942.67)	5010.76 (1057.16)	0.0000	0.15
**Conduct Problems**	Female	F3 Mean	4362.38 (491.29)	4411.99 (537.08)	0.0037	-0.10
**Conduct Problems**	Female	F3 Std	1204.72 (196.03)	1191.19 (222.03)	0.0527	0.06
**Conduct Problems**	Female	F3 Skewness	0.09 (0.56)	0.06 (0.63)	0.1412	0.05
**Conduct Problems**	Female	F3 Kurtosis	-0.54 (0.91)	-0.50 (1.22)	0.2462	-0.04
**Conduct Problems**	Female	F1 1st Derivative Minim	-1922.40 (447.03)	-1878.91 (480.34)	0.0047	-0.09
**Conduct Problems**	Female	F1 1st Derivative Maxin	1929.14 (467.20)	1864.29 (494.98)	0.0000	0.13
**Conduct Problems**	Female	F1 1st Derivative Range	3851.54 (861.47)	3743.19 (923.69)	0.0003	0.12
**Conduct Problems**	Female	F1 1st Derivative Mean	-4.68 (22.33)	-6.01 (28.00)	0.1178	0.05
**Conduct Problems**	Female	F1 1st Derivative Std	487.95 (120.01)	498.36 (135.27)	0.0146	-0.08
**Conduct Problems**	Female	F1 1st Derivative Skewı	0.05 (0.47)	0.02 (0.47)	0.0680	0.06
**Conduct Problems**	Female	F1 1st Derivative Kurtos	6.05 (4.54)	5.52 (4.04)	0.0001	0.12
**Conduct Problems**	Female	F2 1st Derivative Minim	-2243.93 (456.68)	-2192.89 (481.52)	0.0010	-0.11
**Conduct Problems**	Female	F2 1st Derivative Maxin	2225.37 (487.85)	2162.58 (502.73)	0.0001	0.13
**Conduct Problems**	Female	F2 1st Derivative Range	4469.30 (873.54)	4355.47 (909.86)	0.0001	0.13
**Conduct Problems**	Female	F2 1st Derivative Mean	-6.60 (28.06)	-8.51 (34.66)	0.0714	0.06
**Conduct Problems**	Female	F2 1st Derivative Std	660.74 (102.76)	663.24 (112.18)	0.4842	-0.02
**Conduct Problems**	Female	F2 1st Derivative Skewı	-0.01 (0.33)	-0.02 (0.34)	0.4157	0.03
**Conduct Problems**	Female	F2 1st Derivative Kurtos	2.30 (1.36)	2.22 (1.49)	0.1088	0.05
**Conduct Problems**	Female	F3 1st Derivative Minim	-2034.35 (462.71)	-1949.29 (504.65)	0.0000	-0.18
**Conduct Problems**	Female	F3 1st Derivative Maxin	2017.39 (500.94)	1944.84 (543.93)	0.0000	0.14
**Conduct Problems**	Female	F3 1st Derivative Range	4051.74 (904.52)	3894.12 (995.81)	0.0000	0.17
**Conduct Problems**	Female	F3 1st Derivative Mean	-5.65 (37.35)	-7.77 (37.06)	0.0837	0.06
**Conduct Problems**	Female	F3 1st Derivative Std	690.69 (124.97)	678.56 (142.53)	0.0067	0.09
**Conduct Problems**	Female	F3 1st Derivative Skewı	-0.02 (0.36)	-0.02 (0.39)	0.7013	-0.01
**Conduct Problems**	Female	F3 1st Derivative Kurtos	1.08 (1.02)	1.06 (1.02)	0.4960	0.02
**Conduct Problems**	Female	F1 2nd Derivative Minin	-6637.64 (2021.23)	-6422.98 (2131.61)	0.0018	-0.10
**Conduct Problems**	Female	F1 2nd Derivative Maxi	4782.92 (1415.11)	4690.93 (1503.76)	0.0572	0.06
**Conduct Problems**	Female	F1 2nd Derivative Rang	11420.56 (3092.16)	11113.92 (3321.12)	0.0040	0.10
**Conduct Problems**	Female	F1 2nd Derivative Mear	0.46 (42.08)	-0.79 (45.77)	0.3929	0.03
**Conduct Problems**	Female	F1 2nd Derivative Std	1246.85 (354.57)	1274.90 (403.38)	0.0268	-0.07
**Conduct Problems**	Female	F1 2nd Derivative Skew	-0.84 (0.65)	-0.75 (0.64)	0.0001	-0.13
**Conduct Problems**	Female	F1 2nd Derivative Kurtc	9.16 (6.67)	8.34 (6.14)	0.0001	0.13
**Conduct Problems**	Female	F2 2nd Derivative Minin	-7569.65 (2010.24)	-7346.75 (2032.36)	0.0008	-0.11
**Conduct Problems**	Female	F2 2nd Derivative Maxi	6038.71 (1687.13)	5907.75 (1707.29)	0.0193	0.08
**Conduct Problems**	Female	F2 2nd Derivative Rang	13608.37 (3324.75)	13254.51 (3385.43)	0.0014	0.11
**Conduct Problems**	Female	F2 2nd Derivative Mear	3.48 (64.31)	1.47 (60.46)	0.3233	0.03
**Conduct Problems**	Female	F2 2nd Derivative Std	1699.36 (307.80)	1721.24 (347.40)	0.0455	-0.07
**Conduct Problems**	Female	F2 2nd Derivative Skew	-0.55 (0.41)	-0.53 (0.42)	0.0691	-0.06
**Conduct Problems**	Female	F2 2nd Derivative Kurtc	4.27 (2.45)	4.07 (2.53)	0.0127	0.08

**Conduct Problems**	Female	F3 2nd Derivative Minin	-6456.20 (1882.68)	-6246.60 (2026.80)	0.0012	-0.11
**Conduct Problems**	Female	F3 2nd Derivative Maxi	6255.36 (1973.92)	6068.01 (2100.13)	0.0055	0.09
**Conduct Problems**	Female	F3 2nd Derivative Rang	12711.56 (3590.33)	12314.61 (3878.04)	0.0014	0.11
**Conduct Problems**	Female	F3 2nd Derivative Mear	3.72 (67.72)	4.61 (66.16)	0.6859	-0.01
**Conduct Problems**	Female	F3 2nd Derivative Std	1767.16 (350.14)	1758.23 (406.00)	0.4805	0.02
**Conduct Problems**	Female	F3 2nd Derivative Skew	-0.17 (0.37)	-0.16 (0.43)	0.3467	-0.03
**Conduct Problems**	Female	F3 2nd Derivative Kurtc	2.26 (1.43)	2.23 (1.54)	0.5414	0.02
**Peer Problems**	Female	F0 Minimum	58.33 (12.53)	58.87 (12.44)	0.1839	-0.04
**Peer Problems**	Female	F0 Maximum	483.07 (5.28)	482.91 (5.55)	0.3426	0.03
**Peer Problems**	Female	F0 Range	424.74 (15.26)	424.04 (15.03)	0.1518	0.05
**Peer Problems**	Female	F0 Mean	283.23 (16.86)	282.32 (17.19)	0.0955	0.05
**Peer Problems**	Female	F0 Std	112.63 (7.54)	112.84 (7.42)	0.4018	-0.03
**Peer Problems**	Female	F0 Skewness	0.12 (0.17)	0.12 (0.19)	0.5849	0.02
**Peer Problems**	Female	F0 Kurtosis	-1.14 (0.28)	-1.13 (0.39)	0.5859	-0.02
**Peer Problems**	Female	F0 de Minimum	-177.44 (18.34)	-176.79 (19.09)	0.2812	-0.03
**Peer Problems**	Female	F0 de Maximum	188.18 (13.89)	187.02 (15.42)	0.0147	0.08
**Peer Problems**	Female	F0 de Range	365.62 (27.59)	363.82 (30.51)	0.0542	0.06
**Peer Problems**	Female	F0 de Mean	0.17 (0.57)	0.23 (0.77)	0.0084	-0.08
**Peer Problems**	Female	F0 de Std	75.08 (13.44)	75.74 (13.64)	0.1331	-0.05
**Peer Problems**	Female	F0 de Skewness	0.32 (0.18)	0.32 (0.19)	0.2803	0.03
**Peer Problems**	Female	F0 de Kurtosis	-0.35 (1.03)	-0.40 (1.04)	0.1861	0.04
**Peer Problems**	Female	F0 de2 Minimum	-649.41 (60.18)	-645.66 (62.44)	0.0584	-0.06
**Peer Problems**	Female	F0 de2 Maximum	609.81 (77.85)	608.91 (78.68)	0.7243	0.01
**Peer Problems**	Female	F0 de2 Range	1259.22 (127.16)	1254.58 (130.06)	0.2641	0.04
**Peer Problems**	Female	F0 de2 Mean	0.08 (1.43)	0.13 (1.32)	0.2857	-0.03
**Peer Problems**	Female	F0 de2 Std	299.74 (24.45)	299.63 (25.48)	0.8905	0.00
**Peer Problems**	Female	F0 de2 Skewness	-0.06 (0.05)	-0.06 (0.08)	0.9411	0.00
**Peer Problems**	Female	F0 de2 Kurtosis	-1.09 (0.28)	-1.09 (0.33)	0.5532	-0.02
**Peer Problems**	Female	Energy Minimum	-11.42 (6.01)	-11.58 (6.45)	0.4140	0.03
**Peer Problems**	Female	Energy Maximum	3.82 (1.24)	3.92 (1.24)	0.0132	-0.08
**Peer Problems**	Female	Energy Range	15.24 (6.38)	15.50 (6.85)	0.2165	-0.04
**Peer Problems**	Female	Energy Mean	-2.09 (1.04)	-2.00 (1.16)	0.0168	-0.08
**Peer Problems**	Female	Energy Std	2.61 (0.56)	2.64 (0.60)	0.1569	-0.05
**Peer Problems**	Female	Energy Skewness	-0.74 (0.67)	-0.74 (0.69)	0.9090	0.00
**Peer Problems**	Female	Energy Kurtosis	1.64 (5.47)	1.74 (5.54)	0.5743	-0.02
**Peer Problems**	Female	Energy de Minimum	-3.43 (1.61)	-3.39 (1.68)	0.4277	-0.03
**Peer Problems**	Female	Energy de Maximum	4.49 (2.10)	4.52 (2.23)	0.6257	-0.02
**Peer Problems**	Female	Energy de Range	7.92 (3.42)	7.91 (3.56)	0.9429	0.00
**Peer Problems**	Female	Energy de Mean	0.01 (0.02)	0.01 (0.03)	0.0343	-0.07
**Peer Problems**	Female	Energy de Std	1.11 (0.22)	1.10 (0.23)	0.5902	0.02

**Peer Problems**	Female	Energy de Skewness	0.39 (0.50)	0.43 (0.55)	0.0104	-0.08
**Peer Problems**	Female	Energy de Kurtosis	1.70 (3.63)	1.88 (3.95)	0.1323	-0.05
**Peer Problems**	Female	Energy de2 Minimum	-6.96 (3.69)	-6.94 (3.92)	0.8984	0.00
**Peer Problems**	Female	Energy de2 Maximum	8.22 (5.49)	8.28 (5.96)	0.7705	-0.01
**Peer Problems**	Female	Energy de2 Range	15.18 (8.89)	15.22 (9.58)	0.8980	0.00
**Peer Problems**	Female	Energy de2 Mean	-0.01 (0.02)	-0.01 (0.02)	0.0729	0.06
**Peer Problems**	Female	Energy de2 Std	1.55 (0.30)	1.54 (0.33)	0.5179	0.02
**Peer Problems**	Female	Energy de2 Skewness	0.67 (0.59)	0.70 (0.65)	0.2479	-0.04
**Peer Problems**	Female	Energy de2 Kurtosis	5.50 (16.66)	5.86 (17.42)	0.5136	-0.02
**Peer Problems**	Female	MFCC 1 Minimum	-11.42 (6.01)	-11.58 (6.45)	0.4140	0.03
**Peer Problems**	Female	MFCC 1 Maximum	3.82 (1.24)	3.92 (1.24)	0.0132	-0.08
**Peer Problems**	Female	MFCC 1 Range	15.24 (6.38)	15.50 (6.85)	0.2165	-0.04
**Peer Problems**	Female	MFCC 1 Mean	-2.09 (1.04)	-2.00 (1.16)	0.0168	-0.08
**Peer Problems**	Female	MFCC 1 Std	2.61 (0.56)	2.64 (0.60)	0.1569	-0.05
**Peer Problems**	Female	MFCC 1 Skewness	-0.74 (0.67)	-0.74 (0.69)	0.9090	0.00
**Peer Problems**	Female	MFCC 1 Kurtosis	1.64 (5.47)	1.74 (5.54)	0.5743	-0.02
**Peer Problems**	Female	MFCC 2 Minimum	-477.33 (99.40)	-471.82 (109.31)	0.1022	-0.05
**Peer Problems**	Female	MFCC 2 Maximum	354.02 (58.15)	351.29 (65.35)	0.1714	0.04
**Peer Problems**	Female	MFCC 2 Range	831.34 (145.77)	823.11 (162.60)	0.0981	0.05
**Peer Problems**	Female	MFCC 2 Mean	42.00 (21.74)	41.18 (23.08)	0.2582	0.04
**Peer Problems**	Female	MFCC 2 Std	156.56 (25.71)	155.86 (27.95)	0.4230	0.03
**Peer Problems**	Female	MFCC 2 Skewness	-0.69 (0.28)	-0.68 (0.30)	0.0503	-0.06
**Peer Problems**	Female	MFCC 2 Kurtosis	0.30 (0.49)	0.26 (0.53)	0.0272	0.07
**Peer Problems**	Female	MFCC 3 Minimum	-397.32 (110.79)	-388.26 (116.97)	0.0138	-0.08
**Peer Problems**	Female	MFCC 3 Maximum	412.18 (74.71)	407.42 (79.82)	0.0568	0.06
**Peer Problems**	Female	MFCC 3 Range	809.50 (169.19)	795.68 (181.41)	0.0147	0.08
**Peer Problems**	Female	MFCC 3 Mean	29.17 (29.09)	29.54 (29.07)	0.6936	-0.01
**Peer Problems**	Female	MFCC 3 Std	148.57 (27.48)	146.63 (28.67)	0.0328	0.07
**Peer Problems**	Female	MFCC 3 Skewness	-0.21 (0.24)	-0.19 (0.25)	0.0832	-0.06
**Peer Problems**	Female	MFCC 3 Kurtosis	-0.29 (0.36)	-0.29 (0.37)	0.6147	0.02
**Peer Problems**	Female	MFCC 4 Minimum	-461.19 (111.30)	-453.40 (118.50)	0.0358	-0.07
**Peer Problems**	Female	MFCC 4 Maximum	543.01 (105.52)	538.75 (116.42)	0.2346	0.04
**Peer Problems**	Female	MFCC 4 Range	1004.20 (194.93)	992.15 (213.01)	0.0673	0.06
**Peer Problems**	Female	MFCC 4 Mean	19.44 (31.92)	19.87 (31.66)	0.6791	-0.01
**Peer Problems**	Female	MFCC 4 Std	187.62 (30.49)	184.87 (33.23)	0.0076	0.09
**Peer Problems**	Female	MFCC 4 Skewness	0.15 (0.23)	0.16 (0.26)	0.2993	-0.03
**Peer Problems**	Female	MFCC 4 Kurtosis	-0.30 (0.34)	-0.27 (0.39)	0.0101	-0.08
**Peer Problems**	Female	MFCC 5 Minimum	-456.85 (125.14)	-448.55 (134.98)	0.0481	-0.06
**Peer Problems**	Female	MFCC 5 Maximum	395.97 (90.59)	394.78 (94.60)	0.6910	0.01
**Peer Problems**	Female	MFCC 5 Range	852.82 (190.94)	843.34 (207.11)	0.1397	0.05

**Peer Problems**	Female	MFCC 5 Mean	-9.39 (29.52)	-5.91 (30.09)	0.0003	-0.12
**Peer Problems**	Female	MFCC 5 Std	141.21 (25.65)	140.75 (27.94)	0.5980	0.02
**Peer Problems**	Female	MFCC 5 Skewness	-0.15 (0.25)	-0.15 (0.25)	0.8415	-0.01
**Peer Problems**	Female	MFCC 5 Kurtosis	0.02 (0.43)	0.01 (0.43)	0.3665	0.03
**Peer Problems**	Female	MFCC 6 Minimum	-456.65 (111.80)	-443.95 (116.85)	0.0006	-0.11
**Peer Problems**	Female	MFCC 6 Maximum	406.85 (93.76)	398.39 (97.80)	0.0062	0.09
**Peer Problems**	Female	MFCC 6 Range	863.51 (175.32)	842.34 (186.45)	0.0003	0.12
**Peer Problems**	Female	MFCC 6 Mean	-15.18 (32.26)	-13.28 (31.81)	0.0677	-0.06
**Peer Problems**	Female	MFCC 6 Std	143.40 (22.16)	140.11 (23.17)	0.0000	0.15
**Peer Problems**	Female	MFCC 6 Skewness	-0.15 (0.23)	-0.15 (0.25)	0.6573	-0.01
**Peer Problems**	Female	MFCC 6 Kurtosis	0.02 (0.37)	0.02 (0.40)	0.4905	-0.02
**Peer Problems**	Female	MFCC 7 Minimum	-524.77 (123.99)	-511.96 (127.65)	0.0016	-0.10
**Peer Problems**	Female	MFCC 7 Maximum	367.97 (88.82)	360.55 (94.71)	0.0123	0.08
**Peer Problems**	Female	MFCC 7 Range	892.74 (181.50)	872.51 (195.08)	0.0009	0.11
**Peer Problems**	Female	MFCC 7 Mean	-54.91 (34.02)	-51.42 (35.00)	0.0017	-0.10
**Peer Problems**	Female	MFCC 7 Std	148.96 (22.68)	146.62 (25.10)	0.0024	0.10
**Peer Problems**	Female	MFCC 7 Skewness	-0.20 (0.23)	-0.21 (0.24)	0.1138	0.05
**Peer Problems**	Female	MFCC 7 Kurtosis	-0.03 (0.37)	-0.02 (0.40)	0.4129	-0.03
**Peer Problems**	Female	MFCC 8 Minimum	-508.83 (123.80)	-505.57 (134.26)	0.4329	-0.03
**Peer Problems**	Female	MFCC 8 Maximum	358.97 (88.56)	354.26 (91.71)	0.1055	0.05
**Peer Problems**	Female	MFCC 8 Range	867.81 (179.23)	859.83 (196.43)	0.1880	0.04
**Peer Problems**	Female	MFCC 8 Mean	-59.23 (34.08)	-60.13 (35.20)	0.4232	0.03
**Peer Problems**	Female	MFCC 8 Std	142.24 (21.48)	141.93 (23.57)	0.6704	0.01
**Peer Problems**	Female	MFCC 8 Skewness	-0.14 (0.25)	-0.15 (0.26)	0.4137	0.03
**Peer Problems**	Female	MFCC 8 Kurtosis	0.06 (0.41)	0.03 (0.44)	0.0651	0.06
**Peer Problems**	Female	MFCC 9 Minimum	-433.74 (97.10)	-427.77 (104.56)	0.0670	-0.06
**Peer Problems**	Female	MFCC 9 Maximum	372.80 (88.98)	364.08 (90.20)	0.0026	0.10
**Peer Problems**	Female	MFCC 9 Range	806.54 (156.15)	791.86 (167.39)	0.0050	0.09
**Peer Problems**	Female	MFCC 9 Mean	-31.21 (28.42)	-30.95 (28.83)	0.7733	-0.01
**Peer Problems**	Female	MFCC 9 Std	133.77 (18.10)	131.81 (19.93)	0.0014	0.10
**Peer Problems**	Female	MFCC 9 Skewness	-0.02 (0.22)	-0.03 (0.24)	0.1367	0.05
**Peer Problems**	Female	MFCC 9 Kurtosis	-0.04 (0.38)	-0.03 (0.40)	0.2885	-0.03
**Peer Problems**	Female	MFCC 10 Minimum	-386.03 (89.18)	-377.80 (95.45)	0.0058	-0.09
**Peer Problems**	Female	MFCC 10 Maximum	347.43 (89.87)	342.23 (96.09)	0.0836	0.06
**Peer Problems**	Female	MFCC 10 Range	733.46 (154.73)	720.04 (168.01)	0.0100	0.08
**Peer Problems**	Female	MFCC 10 Mean	-23.22 (25.47)	-21.99 (25.01)	0.1351	-0.05
**Peer Problems**	Female	MFCC 10 Std	119.53 (19.87)	118.15 (21.28)	0.0381	0.07
**Peer Problems**	Female	MFCC 10 Skewness	0.02 (0.23)	0.02 (0.25)	0.9842	0.00
**Peer Problems**	Female	MFCC 10 Kurtosis	0.04 (0.38)	0.04 (0.44)	0.5088	0.02
**Peer Problems**	Female	MFCC 11 Minimum	-353.19 (82.26)	-348.02 (87.88)	0.0602	-0.06

**Peer Problems**	Female	MFCC 11 Maximum	291.77 (80.69)	289.90 (84.63)	0.4836	0.02
**Peer Problems**	Female	MFCC 11 Range	644.95 (134.70)	637.92 (144.44)	0.1185	0.05
**Peer Problems**	Female	MFCC 11 Mean	-25.42 (25.73)	-24.29 (26.25)	0.1806	-0.04
**Peer Problems**	Female	MFCC 11 Std	102.24 (15.51)	101.36 (16.27)	0.0856	0.06
**Peer Problems**	Female	MFCC 11 Skewness	-0.04 (0.25)	-0.04 (0.26)	0.4965	-0.02
**Peer Problems**	Female	MFCC 11 Kurtosis	0.15 (0.42)	0.15 (0.44)	0.8450	-0.01
**Peer Problems**	Female	MFCC 12 Minimum	-305.83 (73.54)	-300.04 (77.39)	0.0175	-0.08
**Peer Problems**	Female	MFCC 12 Maximum	269.53 (68.05)	262.52 (68.34)	0.0015	0.10
**Peer Problems**	Female	MFCC 12 Range	575.37 (118.69)	562.56 (124.45)	0.0011	0.11
**Peer Problems**	Female	MFCC 12 Mean	-15.24 (20.00)	-14.64 (20.31)	0.3605	-0.03
**Peer Problems**	Female	MFCC 12 Std	88.70 (12.31)	87.34 (13.23)	0.0010	0.11
**Peer Problems**	Female	MFCC 12 Skewness	-0.04 (0.24)	-0.05 (0.25)	0.4357	0.03
**Peer Problems**	Female	MFCC 12 Kurtosis	0.25 (0.42)	0.24 (0.47)	0.7343	0.01
**Peer Problems**	Female	MFCC 13 Minimum	-252.26 (65.36)	-248.83 (68.27)	0.1122	-0.05
**Peer Problems**	Female	MFCC 13 Maximum	257.47 (63.31)	251.36 (64.16)	0.0030	0.10
**Peer Problems**	Female	MFCC 13 Range	509.73 (107.15)	500.19 (111.77)	0.0070	0.09
**Peer Problems**	Female	MFCC 13 Mean	1.01 (18.24)	-0.02 (18.68)	0.0848	0.06
**Peer Problems**	Female	MFCC 13 Std	77.45 (11.48)	76.41 (11.80)	0.0058	0.09
**Peer Problems**	Female	MFCC 13 Skewness	0.04 (0.25)	0.02 (0.25)	0.0731	0.06
**Peer Problems**	Female	MFCC 13 Kurtosis	0.33 (0.46)	0.29 (0.45)	0.0399	0.07
**Peer Problems**	Female	MELS 1 Minimum	-3.43 (1.61)	-3.39 (1.68)	0.4277	-0.03
**Peer Problems**	Female	MELS 1 Maximum	4.49 (2.10)	4.52 (2.23)	0.6257	-0.02
**Peer Problems**	Female	MELS 1 Range	7.92 (3.42)	7.91 (3.56)	0.9429	0.00
**Peer Problems**	Female	MELS 1 Mean	0.01 (0.02)	0.01 (0.03)	0.0343	-0.07
**Peer Problems**	Female	MELS 1 Std	1.11 (0.22)	1.10 (0.23)	0.5902	0.02
**Peer Problems**	Female	MELS 1 Skewness	0.39 (0.50)	0.43 (0.55)	0.0104	-0.08
**Peer Problems**	Female	MELS 1 Kurtosis	1.70 (3.63)	1.88 (3.95)	0.1323	-0.05
**Peer Problems**	Female	MELS 2 Minimum	-210.49 (46.59)	-206.04 (49.96)	0.0044	-0.09
**Peer Problems**	Female	MELS 2 Maximum	194.89 (42.32)	192.73 (45.31)	0.1272	0.05
**Peer Problems**	Female	MELS 2 Range	405.38 (85.00)	398.78 (91.22)	0.0203	0.07
**Peer Problems**	Female	MELS 2 Mean	0.14 (0.69)	0.20 (0.94)	0.0187	-0.08
**Peer Problems**	Female	MELS 2 Std	58.36 (11.42)	58.02 (12.22)	0.3692	0.03
**Peer Problems**	Female	MELS 2 Skewness	-0.11 (0.22)	-0.08 (0.24)	0.0001	-0.12
**Peer Problems**	Female	MELS 2 Kurtosis	1.11 (0.60)	1.07 (0.61)	0.0672	0.06
**Peer Problems**	Female	MELS 3 Minimum	-175.94 (41.72)	-171.26 (43.58)	0.0007	-0.11
**Peer Problems**	Female	MELS 3 Maximum	167.86 (41.83)	164.36 (43.10)	0.0107	0.08
**Peer Problems**	Female	MELS 3 Range	343.80 (77.49)	335.62 (81.51)	0.0015	0.10
**Peer Problems**	Female	MELS 3 Mean	0.08 (0.66)	0.05 (0.72)	0.3132	0.03
**Peer Problems**	Female	MELS 3 Std	50.36 (8.72)	49.73 (9.13)	0.0281	0.07
**Peer Problems**	Female	MELS 3 Skewness	-0.12 (0.19)	-0.11 (0.19)	0.0060	-0.09

**Peer Problems**	Female	MELS 3 Kurtosis	0.46 (0.45)	0.43 (0.45)	0.0102	0.08
**Peer Problems**	Female	MELS 4 Minimum	-208.93 (45.10)	-207.21 (48.90)	0.2574	-0.04
**Peer Problems**	Female	MELS 4 Maximum	204.07 (47.61)	200.79 (48.96)	0.0351	0.07
**Peer Problems**	Female	MELS 4 Range	413.01 (82.95)	408.00 (89.73)	0.0724	0.06
**Peer Problems**	Female	MELS 4 Mean	0.02 (0.77)	0.07 (0.80)	0.0506	-0.06
**Peer Problems**	Female	MELS 4 Std	60.30 (8.30)	59.69 (8.91)	0.0273	0.07
**Peer Problems**	Female	MELS 4 Skewness	-0.08 (0.19)	-0.07 (0.19)	0.5540	-0.02
**Peer Problems**	Female	MELS 4 Kurtosis	0.42 (0.47)	0.43 (0.49)	0.8809	0.00
**Peer Problems**	Female	MELS 5 Minimum	-179.37 (42.02)	-176.05 (44.36)	0.0172	-0.08
**Peer Problems**	Female	MELS 5 Maximum	171.02 (38.42)	168.78 (40.75)	0.0791	0.06
**Peer Problems**	Female	MELS 5 Range	350.39 (73.13)	344.83 (78.48)	0.0229	0.07
**Peer Problems**	Female	MELS 5 Mean	0.05 (0.59)	0.06 (0.62)	0.5488	-0.02
**Peer Problems**	Female	MELS 5 Std	51.16 (7.29)	50.95 (7.92)	0.4085	0.03
**Peer Problems**	Female	MELS 5 Skewness	-0.06 (0.17)	-0.06 (0.19)	0.9420	0.00
**Peer Problems**	Female	MELS 5 Kurtosis	0.36 (0.39)	0.34 (0.44)	0.2029	0.04
**Peer Problems**	Female	MELS 6 Minimum	-184.41 (42.27)	-178.26 (41.44)	0.0000	-0.15
**Peer Problems**	Female	MELS 6 Maximum	181.51 (40.15)	176.63 (41.52)	0.0002	0.12
**Peer Problems**	Female	MELS 6 Range	365.92 (73.96)	354.90 (75.41)	0.0000	0.15
**Peer Problems**	Female	MELS 6 Mean	0.05 (0.64)	0.06 (0.87)	0.6704	-0.01
**Peer Problems**	Female	MELS 6 Std	52.50 (6.66)	51.51 (6.96)	0.0000	0.14
**Peer Problems**	Female	MELS 6 Skewness	-0.01 (0.16)	-0.01 (0.18)	0.8170	-0.01
**Peer Problems**	Female	MELS 6 Kurtosis	0.42 (0.39)	0.41 (0.47)	0.2972	0.03
**Peer Problems**	Female	MELS 7 Minimum	-198.32 (44.64)	-195.57 (46.49)	0.0618	-0.06
**Peer Problems**	Female	MELS 7 Maximum	188.69 (42.12)	185.77 (45.05)	0.0378	0.07
**Peer Problems**	Female	MELS 7 Range	387.01 (79.22)	381.33 (84.35)	0.0317	0.07
**Peer Problems**	Female	MELS 7 Mean	-0.01 (0.88)	0.01 (0.70)	0.5741	-0.02
**Peer Problems**	Female	MELS 7 Std	55.51 (7.10)	54.65 (7.83)	0.0004	0.11
**Peer Problems**	Female	MELS 7 Skewness	-0.08 (0.16)	-0.07 (0.18)	0.5612	-0.02
**Peer Problems**	Female	MELS 7 Kurtosis	0.46 (0.37)	0.47 (0.44)	0.3651	-0.03
**Peer Problems**	Female	MELS 8 Minimum	-190.92 (44.35)	-190.03 (46.82)	0.5440	-0.02
**Peer Problems**	Female	MELS 8 Maximum	185.85 (41.68)	185.63 (45.75)	0.8743	0.01
**Peer Problems**	Female	MELS 8 Range	376.78 (77.85)	375.66 (84.97)	0.6709	0.01
**Peer Problems**	Female	MELS 8 Mean	-0.04 (0.63)	-0.04 (0.71)	0.8459	0.01
**Peer Problems**	Female	MELS 8 Std	53.46 (6.76)	53.33 (7.60)	0.5694	0.02
**Peer Problems**	Female	MELS 8 Skewness	-0.04 (0.17)	-0.04 (0.19)	0.4545	0.02
**Peer Problems**	Female	MELS 8 Kurtosis	0.48 (0.42)	0.50 (0.45)	0.2208	-0.04
**Peer Problems**	Female	MELS 9 Minimum	-178.32 (39.10)	-174.96 (39.89)	0.0086	-0.08
**Peer Problems**	Female	MELS 9 Maximum	177.44 (39.15)	173.78 (39.36)	0.0040	0.09
**Peer Problems**	Female	MELS 9 Range	355.76 (69.71)	348.74 (72.05)	0.0022	0.10
**Peer Problems**	Female	MELS 9 Mean	-0.02 (0.63)	-0.03 (0.76)	0.7257	0.01

**Peer Problems**	Female	MELS 9 Std	50.86 (5.61)	50.29 (6.25)	0.0029	0.10
**Peer Problems**	Female	MELS 9 Skewness	0.01 (0.17)	0.01 (0.17)	0.8319	-0.01
**Peer Problems**	Female	MELS 9 Kurtosis	0.43 (0.39)	0.42 (0.44)	0.6014	0.02
**Peer Problems**	Female	MELS 10 Minimum	-163.11 (37.39)	-160.61 (38.50)	0.0418	-0.07
**Peer Problems**	Female	MELS 10 Maximum	160.19 (34.56)	158.23 (37.81)	0.0930	0.05
**Peer Problems**	Female	MELS 10 Range	323.30 (65.05)	318.84 (69.16)	0.0397	0.07
**Peer Problems**	Female	MELS 10 Mean	0.01 (0.62)	0.01 (0.63)	0.8435	0.01
**Peer Problems**	Female	MELS 10 Std	46.13 (5.25)	45.76 (5.97)	0.0437	0.06
**Peer Problems**	Female	MELS 10 Skewness	-0.01 (0.16)	-0.02 (0.17)	0.3364	0.03
**Peer Problems**	Female	MELS 10 Kurtosis	0.42 (0.39)	0.41 (0.41)	0.7466	0.01
**Peer Problems**	Female	MELS 11 Minimum	-145.82 (32.12)	-143.97 (33.61)	0.0805	-0.06
**Peer Problems**	Female	MELS 11 Maximum	144.13 (31.10)	141.99 (33.02)	0.0384	0.07
**Peer Problems**	Female	MELS 11 Range	289.95 (56.74)	285.95 (60.37)	0.0344	0.07
**Peer Problems**	Female	MELS 11 Mean	-0.04 (0.56)	-0.01 (0.70)	0.0870	-0.05
**Peer Problems**	Female	MELS 11 Std	41.63 (4.50)	41.18 (4.98)	0.0032	0.10
**Peer Problems**	Female	MELS 11 Skewness	-0.01 (0.15)	-0.01 (0.16)	0.2499	0.04
**Peer Problems**	Female	MELS 11 Kurtosis	0.38 (0.39)	0.39 (0.40)	0.2761	-0.04
**Peer Problems**	Female	MELS 12 Minimum	-129.18 (27.18)	-127.27 (28.76)	0.0347	-0.07
**Peer Problems**	Female	MELS 12 Maximum	130.03 (28.97)	127.70 (30.17)	0.0147	0.08
**Peer Problems**	Female	MELS 12 Range	259.21 (50.40)	254.97 (53.08)	0.0112	0.08
**Peer Problems**	Female	MELS 12 Mean	-0.03 (0.48)	-0.01 (0.54)	0.0928	-0.05
**Peer Problems**	Female	MELS 12 Std	37.21 (3.93)	36.71 (4.29)	0.0002	0.12
**Peer Problems**	Female	MELS 12 Skewness	-0.00 (0.15)	-0.01 (0.16)	0.2118	0.04
**Peer Problems**	Female	MELS 12 Kurtosis	0.37 (0.35)	0.38 (0.40)	0.2499	-0.04
**Peer Problems**	Female	MELS 13 Minimum	-115.15 (23.99)	-113.11 (24.64)	0.0093	-0.08
**Peer Problems**	Female	MELS 13 Maximum	117.40 (25.53)	114.74 (25.84)	0.0013	0.10
**Peer Problems**	Female	MELS 13 Range	232.55 (44.13)	227.84 (45.47)	0.0011	0.11
**Peer Problems**	Female	MELS 13 Mean	0.01 (0.42)	-0.02 (0.45)	0.0295	0.07
**Peer Problems**	Female	MELS 13 Std	33.28 (3.59)	32.89 (3.75)	0.0010	0.11
**Peer Problems**	Female	MELS 13 Skewness	0.02 (0.15)	0.01 (0.16)	0.2455	0.04
**Peer Problems**	Female	MELS 13 Kurtosis	0.39 (0.37)	0.38 (0.39)	0.5341	0.02
**Peer Problems**	Female	MELS 14 Minimum	-6.96 (3.69)	-6.94 (3.92)	0.8984	0.00
**Peer Problems**	Female	MELS 14 Maximum	8.22 (5.49)	8.28 (5.96)	0.7705	-0.01
**Peer Problems**	Female	MELS 14 Range	15.18 (8.89)	15.22 (9.58)	0.8980	0.00
**Peer Problems**	Female	MELS 14 Mean	-0.01 (0.02)	-0.01 (0.02)	0.0729	0.06
**Peer Problems**	Female	MELS 14 Std	1.55 (0.30)	1.54 (0.33)	0.5179	0.02
**Peer Problems**	Female	MELS 14 Skewness	0.67 (0.59)	0.70 (0.65)	0.2479	-0.04
**Peer Problems**	Female	MELS 14 Kurtosis	5.50 (16.66)	5.86 (17.42)	0.5136	-0.02
**Peer Problems**	Female	MELS 15 Minimum	-227.01 (62.32)	-224.65 (63.84)	0.2477	-0.04
**Peer Problems**	Female	MELS 15 Maximum	335.71 (88.17)	330.50 (94.00)	0.0769	0.06

**Peer Problems**	Female	MELS 15 Range	562.71 (138.21)	555.15 (146.43)	0.1000	0.05
**Peer Problems**	Female	MELS 15 Mean	-0.02 (0.76)	0.04 (0.79)	0.0088	-0.08
**Peer Problems**	Female	MELS 15 Std	63.60 (11.26)	63.20 (11.62)	0.2698	0.04
**Peer Problems**	Female	MELS 15 Skewness	0.85 (0.34)	0.84 (0.35)	0.3117	0.03
**Peer Problems**	Female	MELS 15 Kurtosis	3.30 (1.63)	3.25 (1.66)	0.3446	0.03
**Peer Problems**	Female	MELS 16 Minimum	-254.49 (69.33)	-249.65 (68.02)	0.0295	-0.07
**Peer Problems**	Female	MELS 16 Maximum	235.03 (60.02)	230.57 (62.14)	0.0240	0.07
**Peer Problems**	Female	MELS 16 Range	489.52 (115.21)	480.22 (117.44)	0.0135	0.08
**Peer Problems**	Female	MELS 16 Mean	-0.13 (0.96)	-0.14 (1.08)	0.7252	0.01
**Peer Problems**	Female	MELS 16 Std	60.90 (7.71)	60.47 (7.89)	0.0860	0.06
**Peer Problems**	Female	MELS 16 Skewness	-0.14 (0.23)	-0.14 (0.24)	0.8607	-0.01
**Peer Problems**	Female	MELS 16 Kurtosis	1.12 (0.91)	1.09 (0.89)	0.2686	0.04
**Peer Problems**	Female	MELS 17 Minimum	-288.82 (70.29)	-288.08 (75.69)	0.7524	-0.01
**Peer Problems**	Female	MELS 17 Maximum	282.51 (68.75)	280.25 (74.55)	0.3281	0.03
**Peer Problems**	Female	MELS 17 Range	571.34 (124.18)	568.33 (135.74)	0.4737	0.02
**Peer Problems**	Female	MELS 17 Mean	-0.12 (1.24)	-0.16 (1.10)	0.2234	0.04
**Peer Problems**	Female	MELS 17 Std	72.57 (8.19)	72.19 (8.65)	0.1605	0.05
**Peer Problems**	Female	MELS 17 Skewness	-0.08 (0.18)	-0.08 (0.20)	0.7700	0.01
**Peer Problems**	Female	MELS 17 Kurtosis	0.99 (0.68)	1.02 (0.75)	0.1154	-0.05
**Peer Problems**	Female	MELS 18 Minimum	-268.30 (61.80)	-266.53 (65.76)	0.3902	-0.03
**Peer Problems**	Female	MELS 18 Maximum	265.53 (62.70)	262.31 (66.06)	0.1216	0.05
**Peer Problems**	Female	MELS 18 Range	533.83 (108.36)	528.84 (117.14)	0.1705	0.04
**Peer Problems**	Female	MELS 18 Mean	0.01 (0.93)	-0.03 (1.19)	0.2227	0.04
**Peer Problems**	Female	MELS 18 Std	71.03 (6.63)	70.80 (7.32)	0.2969	0.03
**Peer Problems**	Female	MELS 18 Skewness	-0.03 (0.15)	-0.03 (0.16)	0.8448	-0.01
**Peer Problems**	Female	MELS 18 Kurtosis	0.65 (0.55)	0.66 (0.56)	0.7414	-0.01
**Peer Problems**	Female	MELS 19 Minimum	-284.58 (66.08)	-276.42 (67.56)	0.0002	-0.12
**Peer Problems**	Female	MELS 19 Maximum	282.34 (64.83)	275.95 (63.44)	0.0021	0.10
**Peer Problems**	Female	MELS 19 Range	566.92 (114.63)	552.37 (116.51)	0.0001	0.13
**Peer Problems**	Female	MELS 19 Mean	-0.03 (0.94)	0.04 (1.34)	0.0551	-0.06
**Peer Problems**	Female	MELS 19 Std	75.46 (7.01)	74.69 (6.90)	0.0007	0.11
**Peer Problems**	Female	MELS 19 Skewness	0.00 (0.16)	0.01 (0.16)	0.2881	-0.03
**Peer Problems**	Female	MELS 19 Kurtosis	0.65 (0.61)	0.61 (0.61)	0.0403	0.07
**Peer Problems**	Female	MELS 20 Minimum	-298.79 (67.77)	-293.73 (69.29)	0.0224	-0.07
**Peer Problems**	Female	MELS 20 Maximum	317.23 (74.22)	311.20 (78.35)	0.0144	0.08
**Peer Problems**	Female	MELS 20 Range	616.02 (126.17)	604.93 (132.93)	0.0081	0.09
**Peer Problems**	Female	MELS 20 Mean	-0.03 (1.10)	0.05 (0.99)	0.0256	-0.07
**Peer Problems**	Female	MELS 20 Std	81.67 (7.32)	80.95 (7.57)	0.0028	0.10
**Peer Problems**	Female	MELS 20 Skewness	0.07 (0.15)	0.07 (0.16)	0.6148	0.02
**Peer Problems**	Female	MELS 20 Kurtosis	0.70 (0.58)	0.68 (0.82)	0.4477	0.02

**Peer Problems**	Female	MELS 21 Minimum	-301.44 (65.11)	-299.32 (72.01)	0.3372	-0.03
**Peer Problems**	Female	MELS 21 Maximum	305.93 (68.12)	303.48 (74.88)	0.2887	0.03
**Peer Problems**	Female	MELS 21 Range	607.37 (117.78)	602.80 (131.73)	0.2561	0.04
**Peer Problems**	Female	MELS 21 Mean	0.07 (0.92)	0.05 (1.27)	0.5899	0.02
**Peer Problems**	Female	MELS 21 Std	81.92 (6.98)	81.66 (7.51)	0.2621	0.04
**Peer Problems**	Female	MELS 21 Skewness	0.02 (0.15)	0.02 (0.16)	0.7556	0.01
**Peer Problems**	Female	MELS 21 Kurtosis	0.61 (0.49)	0.62 (0.81)	0.4338	-0.02
**Peer Problems**	Female	MELS 22 Minimum	-292.90 (62.11)	-287.17 (63.90)	0.0049	-0.09
**Peer Problems**	Female	MELS 22 Maximum	283.97 (54.95)	279.87 (57.89)	0.0244	0.07
**Peer Problems**	Female	MELS 22 Range	576.87 (102.03)	567.03 (108.28)	0.0038	0.09
**Peer Problems**	Female	MELS 22 Mean	0.08 (1.05)	0.03 (1.07)	0.0987	0.05
**Peer Problems**	Female	MELS 22 Std	79.90 (5.89)	79.43 (6.28)	0.0182	0.08
**Peer Problems**	Female	MELS 22 Skewness	-0.02 (0.13)	-0.01 (0.13)	0.3879	-0.03
**Peer Problems**	Female	MELS 22 Kurtosis	0.48 (0.43)	0.46 (0.44)	0.1521	0.05
**Peer Problems**	Female	MELS 23 Minimum	-275.00 (54.48)	-271.44 (57.00)	0.0477	-0.06
**Peer Problems**	Female	MELS 23 Maximum	274.14 (54.29)	270.43 (56.31)	0.0378	0.07
**Peer Problems**	Female	MELS 23 Range	549.14 (94.45)	541.86 (100.19)	0.0206	0.07
**Peer Problems**	Female	MELS 23 Mean	0.05 (1.04)	0.04 (1.13)	0.6600	0.01
**Peer Problems**	Female	MELS 23 Std	76.85 (5.55)	76.42 (5.95)	0.0216	0.07
**Peer Problems**	Female	MELS 23 Skewness	0.00 (0.13)	0.00 (0.15)	0.8177	-0.01
**Peer Problems**	Female	MELS 23 Kurtosis	0.43 (0.42)	0.43 (0.47)	0.6741	0.01
**Peer Problems**	Female	MELS 24 Minimum	-258.05 (50.65)	-254.52 (52.85)	0.0350	-0.07
**Peer Problems**	Female	MELS 24 Maximum	259.02 (50.69)	255.67 (53.43)	0.0465	0.06
**Peer Problems**	Female	MELS 24 Range	517.07 (88.98)	510.19 (94.07)	0.0201	0.08
**Peer Problems**	Female	MELS 24 Mean	0.03 (0.92)	0.05 (1.34)	0.5685	-0.02
**Peer Problems**	Female	MELS 24 Std	72.80 (5.34)	72.49 (5.68)	0.0762	0.06
**Peer Problems**	Female	MELS 24 Skewness	0.01 (0.12)	0.01 (0.13)	0.7885	-0.01
**Peer Problems**	Female	MELS 24 Kurtosis	0.40 (0.40)	0.41 (0.61)	0.7981	-0.01
**Peer Problems**	Female	MELS 25 Minimum	-240.55 (46.45)	-237.93 (48.73)	0.0881	-0.06
**Peer Problems**	Female	MELS 25 Maximum	237.66 (44.93)	237.19 (47.43)	0.7528	0.01
**Peer Problems**	Female	MELS 25 Range	478.21 (79.81)	475.12 (84.98)	0.2452	0.04
**Peer Problems**	Female	MELS 25 Mean	0.02 (0.75)	0.06 (1.05)	0.1304	-0.05
**Peer Problems**	Female	MELS 25 Std	67.92 (5.03)	67.65 (5.17)	0.1033	0.05
**Peer Problems**	Female	MELS 25 Skewness	-0.01 (0.12)	-0.00 (0.12)	0.0409	-0.07
**Peer Problems**	Female	MELS 25 Kurtosis	0.37 (0.37)	0.39 (0.45)	0.2741	-0.04
**Peer Problems**	Female	MELS 26 Minimum	-222.43 (42.97)	-219.41 (43.70)	0.0311	-0.07
**Peer Problems**	Female	MELS 26 Maximum	220.58 (41.24)	217.96 (42.81)	0.0537	0.06
**Peer Problems**	Female	MELS 26 Range	443.01 (73.41)	437.37 (76.63)	0.0200	0.08
**Peer Problems**	Female	MELS 26 Mean	-0.00 (0.94)	0.01 (0.97)	0.6961	-0.01
**Peer Problems**	Female	MELS 26 Std	62.94 (4.70)	62.72 (4.98)	0.1555	0.05

**Peer Problems**	Female	MELS 26 Skewness	-0.01 (0.12)	-0.01 (0.13)	0.4725	0.02
**Peer Problems**	Female	MELS 26 Kurtosis	0.36 (0.36)	0.36 (0.43)	0.6673	0.01
**Peer Problems**	Female	F1 Minimum	217.35 (62.47)	225.03 (100.11)	0.0039	-0.09
**Peer Problems**	Female	F1 Maximum	4637.80 (895.28)	4626.49 (897.25)	0.6964	0.01
**Peer Problems**	Female	F1 Range	4420.45 (902.50)	4401.46 (903.76)	0.5158	0.02
**Peer Problems**	Female	F1 Mean	1044.60 (439.74)	1095.26 (481.26)	0.0007	-0.11
**Peer Problems**	Female	F1 Std	897.27 (250.97)	915.71 (257.67)	0.0249	-0.07
**Peer Problems**	Female	F1 Skewness	2.03 (1.09)	1.89 (1.06)	0.0000	0.14
**Peer Problems**	Female	F1 Kurtosis	5.30 (6.26)	4.51 (5.18)	0.0000	0.14
**Peer Problems**	Female	F2 Minimum	594.08 (400.68)	657.00 (512.85)	0.0000	-0.14
**Peer Problems**	Female	F2 Maximum	6323.26 (554.77)	6318.45 (538.93)	0.7856	0.01
**Peer Problems**	Female	F2 Range	5729.18 (742.15)	5661.44 (808.40)	0.0068	0.09
**Peer Problems**	Female	F2 Mean	2543.98 (628.68)	2621.47 (694.35)	0.0003	-0.12
**Peer Problems**	Female	F2 Std	1239.17 (219.71)	1250.87 (225.38)	0.1037	-0.05
**Peer Problems**	Female	F2 Skewness	0.87 (0.62)	0.81 (0.62)	0.0024	0.10
**Peer Problems**	Female	F2 Kurtosis	0.56 (1.42)	0.40 (1.30)	0.0003	0.12
**Peer Problems**	Female	F3 Minimum	1870.07 (709.74)	1936.33 (835.02)	0.0079	-0.09
**Peer Problems**	Female	F3 Maximum	6976.66 (395.53)	6968.17 (404.00)	0.5116	0.02
**Peer Problems**	Female	F3 Range	5106.59 (952.40)	5031.85 (1083.14)	0.0229	0.07
**Peer Problems**	Female	F3 Mean	4369.98 (491.80)	4417.87 (549.43)	0.0044	-0.09
**Peer Problems**	Female	F3 Std	1200.67 (197.87)	1191.88 (227.44)	0.2001	0.04
**Peer Problems**	Female	F3 Skewness	0.09 (0.57)	0.05 (0.64)	0.0396	0.07
**Peer Problems**	Female	F3 Kurtosis	-0.54 (0.94)	-0.49 (1.28)	0.1267	-0.05
**Peer Problems**	Female	F1 1st Derivative Minim	-1908.91 (464.34)	-1881.68 (471.34)	0.0719	-0.06
**Peer Problems**	Female	F1 1st Derivative Maxin	1904.74 (480.36)	1873.39 (490.01)	0.0457	0.06
**Peer Problems**	Female	F1 1st Derivative Range	3813.65 (894.50)	3755.08 (907.22)	0.0444	0.07
**Peer Problems**	Female	F1 1st Derivative Mean	-5.68 (23.62)	-5.24 (28.29)	0.6000	-0.02
**Peer Problems**	Female	F1 1st Derivative Std	491.20 (129.03)	497.67 (129.93)	0.1221	-0.05
**Peer Problems**	Female	F1 1st Derivative Skewı	0.04 (0.46)	0.02 (0.48)	0.4028	0.03
**Peer Problems**	Female	F1 1st Derivative Kurtos	5.97 (4.57)	5.46 (3.83)	0.0002	0.12
**Peer Problems**	Female	F2 1st Derivative Minim	-2225.53 (466.65)	-2199.13 (478.61)	0.0839	-0.06
**Peer Problems**	Female	F2 1st Derivative Maxin	2205.08 (491.73)	2167.52 (503.97)	0.0196	0.08
**Peer Problems**	Female	F2 1st Derivative Range	4430.62 (883.22)	4366.64 (912.05)	0.0275	0.07
**Peer Problems**	Female	F2 1st Derivative Mean	-7.41 (29.66)	-8.13 (34.91)	0.4934	0.02
**Peer Problems**	Female	F2 1st Derivative Std	661.09 (107.87)	663.57 (109.25)	0.4801	-0.02
**Peer Problems**	Female	F2 1st Derivative Skewı	-0.01 (0.34)	-0.02 (0.33)	0.3870	0.03
**Peer Problems**	Female	F2 1st Derivative Kurtos	2.31 (1.46)	2.18 (1.40)	0.0060	0.09
**Peer Problems**	Female	F3 1st Derivative Minim	-2003.59 (472.18)	-1959.79 (509.05)	0.0057	-0.09
**Peer Problems**	Female	F3 1st Derivative Maxin	1992.50 (506.88)	1952.24 (551.43)	0.0184	0.08
**Peer Problems**	Female	F3 1st Derivative Range	3996.08 (924.31)	3912.02 (1005.05)	0.0070	0.09

**Peer Problems**	Female	F3 1st Derivative Mean	-6.74 (32.65)	-7.13 (41.84)	0.7489	0.01
**Peer Problems**	Female	F3 1st Derivative Std	685.48 (129.58)	681.01 (142.87)	0.3090	0.03
**Peer Problems**	Female	F3 1st Derivative Skewı	-0.01 (0.34)	-0.03 (0.42)	0.2056	0.04
**Peer Problems**	Female	F3 1st Derivative Kurtos	1.06 (0.93)	1.07 (1.11)	0.8859	0.00
**Peer Problems**	Female	F1 2nd Derivative Minin	-6549.62 (2094.02)	-6461.55 (2086.08)	0.1928	-0.04
**Peer Problems**	Female	F1 2nd Derivative Maxi	4755.65 (1460.70)	4695.33 (1479.28)	0.2044	0.04
**Peer Problems**	Female	F1 2nd Derivative Rang	11305.27 (3217.87)	11156.88 (3252.91)	0.1561	0.05
**Peer Problems**	Female	F1 2nd Derivative Mear	-0.32 (38.57)	-0.26 (50.20)	0.9719	0.00
**Peer Problems**	Female	F1 2nd Derivative Std	1256.12 (380.39)	1272.45 (389.78)	0.1897	-0.04
**Peer Problems**	Female	F1 2nd Derivative Skew	-0.81 (0.67)	-0.76 (0.62)	0.0203	-0.08
**Peer Problems**	Female	F1 2nd Derivative Kurtc	8.98 (6.70)	8.30 (5.93)	0.0009	0.11
**Peer Problems**	Female	F2 2nd Derivative Minin	-7471.46 (2005.74)	-7394.68 (2049.52)	0.2414	-0.04
**Peer Problems**	Female	F2 2nd Derivative Maxi	6006.23 (1690.16)	5906.67 (1710.79)	0.0702	0.06
**Peer Problems**	Female	F2 2nd Derivative Rang	13477.68 (3330.70)	13301.34 (3403.52)	0.1052	0.05
**Peer Problems**	Female	F2 2nd Derivative Mear	3.01 (53.15)	1.42 (70.96)	0.4284	0.03
**Peer Problems**	Female	F2 2nd Derivative Std	1707.67 (329.96)	1718.07 (334.92)	0.3331	-0.03
**Peer Problems**	Female	F2 2nd Derivative Skew	-0.54 (0.42)	-0.53 (0.42)	0.3458	-0.03
**Peer Problems**	Female	F2 2nd Derivative Kurtc	4.23 (2.53)	4.05 (2.47)	0.0251	0.07
**Peer Problems**	Female	F3 2nd Derivative Minin	-6409.45 (1920.43)	-6238.77 (2028.63)	0.0075	-0.09
**Peer Problems**	Female	F3 2nd Derivative Maxi	6182.17 (1981.56)	6097.46 (2131.23)	0.2019	0.04
**Peer Problems**	Female	F3 2nd Derivative Rang	12591.62 (3636.77)	12336.23 (3915.74)	0.0362	0.07
**Peer Problems**	Female	F3 2nd Derivative Mear	3.85 (57.86)	4.72 (75.84)	0.6873	-0.01
**Peer Problems**	Female	F3 2nd Derivative Std	1768.78 (363.16)	1753.70 (408.03)	0.2255	0.04
**Peer Problems**	Female	F3 2nd Derivative Skew	-0.18 (0.39)	-0.15 (0.42)	0.0580	-0.06
**Peer Problems**	Female	F3 2nd Derivative Kurtc	2.27 (1.49)	2.21 (1.50)	0.2204	0.04
