# Supplementary material for: Using Speech Features and Machine Learning Models to Predict Emotional and Behavioral Problems in Chinese Adolescents
Source: Depress Anxiety. 2025 Jun 16;2025:5734107. doi: 10.1155/da/5734107 (PMC12185205; doi:10.1155/da/5734107)
Supplement: Supporting Information 1 — The supporting information file includes all additional figures and tables referenced in the main text as Appendix A–G. [file 5734107.f1.zip › Appendix C.pdf]

Male

Symptom	Best Subspace	Best Subspace AUC	Compared Subspace
Emotional Symptoms	MFCC+PROS	0.7383 ± 0.0067	FORM
Emotional Symptoms	MFCC+PROS	0.7383 ± 0.0067	MELS
Emotional Symptoms	MFCC+PROS	0.7383 ± 0.0067	MELS+FORM
Emotional Symptoms	MFCC+PROS	0.7383 ± 0.0067	MELS+PROS
Emotional Symptoms	MFCC+PROS	0.7383 ± 0.0067	MELS+PROS+FORM
Emotional Symptoms	MFCC+PROS	0.7383 ± 0.0067	MFCC
Emotional Symptoms	MFCC+PROS	0.7383 ± 0.0067	MFCC+FORM
Emotional Symptoms	MFCC+PROS	0.7383 ± 0.0067	MFCC+MELS
Emotional Symptoms	MFCC+PROS	0.7383 ± 0.0067	MFCC+MELS+FORM
Emotional Symptoms	MFCC+PROS	0.7383 ± 0.0067	MFCC+MELS+PROS
Emotional Symptoms	MFCC+PROS	0.7383 ± 0.0067	MFCC+MELS+PROS+FORM
Emotional Symptoms	MFCC+PROS	0.7383 ± 0.0067	MFCC+PROS+FORM
Emotional Symptoms	MFCC+PROS	0.7383 ± 0.0067	PROS
Emotional Symptoms	MFCC+PROS	0.7383 ± 0.0067	PROS+FORM
Hyperactivity	MFCC+MELS	0.7826 ± 0.0110	FORM
Hyperactivity	MFCC+MELS	0.7826 ± 0.0110	MELS
Hyperactivity	MFCC+MELS	0.7826 ± 0.0110	MELS+FORM
Hyperactivity	MFCC+MELS	0.7826 ± 0.0110	MELS+PROS
Hyperactivity	MFCC+MELS	0.7826 ± 0.0110	MELS+PROS+FORM
Hyperactivity	MFCC+MELS	0.7826 ± 0.0110	MFCC
Hyperactivity	MFCC+MELS	0.7826 ± 0.0110	MFCC+FORM
Hyperactivity	MFCC+MELS	0.7826 ± 0.0110	MFCC+MELS+FORM
Hyperactivity	MFCC+MELS	0.7826 ± 0.0110	MFCC+MELS+PROS
Hyperactivity	MFCC+MELS	0.7826 ± 0.0110	MFCC+MELS+PROS+FORM
Hyperactivity	MFCC+MELS	0.7826 ± 0.0110	MFCC+PROS
Hyperactivity	MFCC+MELS	0.7826 ± 0.0110	MFCC+PROS+FORM
Hyperactivity	MFCC+MELS	0.7826 ± 0.0110	PROS
Hyperactivity	MFCC+MELS	0.7826 ± 0.0110	PROS+FORM
Conduct Problems	PROS	0.5974 ± 0.0116	FORM
Conduct Problems	PROS	0.5974 ± 0.0116	MELS
Conduct Problems	PROS	0.5974 ± 0.0116	MELS+FORM
Conduct Problems	PROS	0.5974 ± 0.0116	MELS+PROS
Conduct Problems	PROS	0.5974 ± 0.0116	MELS+PROS+FORM
Conduct Problems	PROS	0.5974 ± 0.0116	MFCC
Conduct Problems	PROS	0.5974 ± 0.0116	MFCC+FORM
Conduct Problems	PROS	0.5974 ± 0.0116	MFCC+MELS
Conduct Problems	PROS	0.5974 ± 0.0116	MFCC+MELS+FORM
Conduct Problems	PROS	0.5974 ± 0.0116	MFCC+MELS+PROS
Conduct Problems	PROS	0.5974 ± 0.0116	MFCC+MELS+PROS+FORM
Conduct Problems	PROS	0.5974 ± 0.0116	MFCC+PROS
Conduct Problems	PROS	0.5974 ± 0.0116	MFCC+PROS+FORM

Conduct Problems	PROS	0.5974 ± 0.0116	PROS+FORM
Peer Problems	MFCC+MELS+PROS	0.6258 ± 0.0183	FORM
Peer Problems	MFCC+MELS+PROS	0.6258 ± 0.0183	MELS
Peer Problems	MFCC+MELS+PROS	0.6258 ± 0.0183	MELS+FORM
Peer Problems	MFCC+MELS+PROS	0.6258 ± 0.0183	MELS+PROS
Peer Problems	MFCC+MELS+PROS	0.6258 ± 0.0183	MELS+PROS+FORM
Peer Problems	MFCC+MELS+PROS	0.6258 ± 0.0183	MFCC
Peer Problems	MFCC+MELS+PROS	0.6258 ± 0.0183	MFCC+FORM
Peer Problems	MFCC+MELS+PROS	0.6258 ± 0.0183	MFCC+MELS
Peer Problems	MFCC+MELS+PROS	0.6258 ± 0.0183	MFCC+MELS+FORM
Peer Problems	MFCC+MELS+PROS	0.6258 ± 0.0183	MFCC+MELS+PROS+FORM
Peer Problems	MFCC+MELS+PROS	0.6258 ± 0.0183	MFCC+PROS
Peer Problems	MFCC+MELS+PROS	0.6258 ± 0.0183	MFCC+PROS+FORM
Peer Problems	MFCC+MELS+PROS	0.6258 ± 0.0183	PROS
Peer Problems	MFCC+MELS+PROS	0.6258 ± 0.0183	PROS+FORM

Female

Symptom	Best Subspace	Best Subspace AUC	Compared Subspace
Emotional Symptoms	MFCC+PROS	0.6581 ± 0.0181	FORM
Emotional Symptoms	MFCC+PROS	0.6581 ± 0.0181	MELS
Emotional Symptoms	MFCC+PROS	0.6581 ± 0.0181	MELS+FORM
Emotional Symptoms	MFCC+PROS	0.6581 ± 0.0181	MELS+PROS
Emotional Symptoms	MFCC+PROS	0.6581 ± 0.0181	MELS+PROS+FORM
Emotional Symptoms	MFCC+PROS	0.6581 ± 0.0181	MFCC
Emotional Symptoms	MFCC+PROS	0.6581 ± 0.0181	MFCC+FORM
Emotional Symptoms	MFCC+PROS	0.6581 ± 0.0181	MFCC+MELS
Emotional Symptoms	MFCC+PROS	0.6581 ± 0.0181	MFCC+MELS+FORM
Emotional Symptoms	MFCC+PROS	0.6581 ± 0.0181	MFCC+MELS+PROS
Emotional Symptoms	MFCC+PROS	0.6581 ± 0.0181	MFCC+MELS+PROS+FORM
Emotional Symptoms	MFCC+PROS	0.6581 ± 0.0181	MFCC+PROS+FORM
Emotional Symptoms	MFCC+PROS	0.6581 ± 0.0181	PROS
Emotional Symptoms	MFCC+PROS	0.6581 ± 0.0181	PROS+FORM
Hyperactivity	MFCC	0.7801 ± 0.0157	FORM
Hyperactivity	MFCC	0.7801 ± 0.0157	MELS
Hyperactivity	MFCC	0.7801 ± 0.0157	MELS+FORM
Hyperactivity	MFCC	0.7801 ± 0.0157	MELS+PROS
Hyperactivity	MFCC	0.7801 ± 0.0157	MELS+PROS+FORM
Hyperactivity	MFCC	0.7801 ± 0.0157	MFCC+FORM
Hyperactivity	MFCC	0.7801 ± 0.0157	MFCC+MELS
Hyperactivity	MFCC	0.7801 ± 0.0157	MFCC+MELS+FORM
Hyperactivity	MFCC	0.7801 ± 0.0157	MFCC+MELS+PROS
Hyperactivity	MFCC	0.7801 ± 0.0157	MFCC+MELS+PROS+FORM
Hyperactivity	MFCC	0.7801 ± 0.0157	MFCC+PROS
Hyperactivity	MFCC	0.7801 ± 0.0157	MFCC+PROS+FORM

Hyperactivity	MFCC	0.7801 ± 0.0157	PROS
Hyperactivity	MFCC	0.7801 ± 0.0157	PROS+FORM
Conduct Problems	MFCC+FORM	0.6218 ± 0.0278	FORM
Conduct Problems	MFCC+FORM	0.6218 ± 0.0278	MELS
Conduct Problems	MFCC+FORM	0.6218 ± 0.0278	MELS+FORM
Conduct Problems	MFCC+FORM	0.6218 ± 0.0278	MELS+PROS
Conduct Problems	MFCC+FORM	0.6218 ± 0.0278	MELS+PROS+FORM
Conduct Problems	MFCC+FORM	0.6218 ± 0.0278	MFCC
Conduct Problems	MFCC+FORM	0.6218 ± 0.0278	MFCC+MELS
Conduct Problems	MFCC+FORM	0.6218 ± 0.0278	MFCC+MELS+FORM
Conduct Problems	MFCC+FORM	0.6218 ± 0.0278	MFCC+MELS+PROS
Conduct Problems	MFCC+FORM	0.6218 ± 0.0278	MFCC+MELS+PROS+FORM
Conduct Problems	MFCC+FORM	0.6218 ± 0.0278	MFCC+PROS
Conduct Problems	MFCC+FORM	0.6218 ± 0.0278	MFCC+PROS+FORM
Conduct Problems	MFCC+FORM	0.6218 ± 0.0278	PROS
Conduct Problems	MFCC+FORM	0.6218 ± 0.0278	PROS+FORM
Peer Problems	MELS	0.5707 ± 0.0126	FORM
Peer Problems	MELS	0.5707 ± 0.0126	MELS+FORM
Peer Problems	MELS	0.5707 ± 0.0126	MELS+PROS
Peer Problems	MELS	0.5707 ± 0.0126	MELS+PROS+FORM
Peer Problems	MELS	0.5707 ± 0.0126	MFCC
Peer Problems	MELS	0.5707 ± 0.0126	MFCC+FORM
Peer Problems	MELS	0.5707 ± 0.0126	MFCC+MELS
Peer Problems	MELS	0.5707 ± 0.0126	MFCC+MELS+FORM
Peer Problems	MELS	0.5707 ± 0.0126	MFCC+MELS+PROS
Peer Problems	MELS	0.5707 ± 0.0126	MFCC+MELS+PROS+FORM
Peer Problems	MELS	0.5707 ± 0.0126	MFCC+PROS
Peer Problems	MELS	0.5707 ± 0.0126	MFCC+PROS+FORM
Peer Problems	MELS	0.5707 ± 0.0126	PROS
Peer Problems	MELS	0.5707 ± 0.0126	PROS+FORM

Compared Subspace AUC	t_stat	p_value	Significance
0.7116 ± 0.0124	4.746713548	0.008992625	TRUE
0.7241 ± 0.0161	2.14456465	0.098581157	FALSE
0.7084 ± 0.0258	2.15514981	0.097417282	FALSE
0.7321 ± 0.0146	1.242944576	0.281769238	FALSE
0.7109 ± 0.0247	2.096216187	0.104097076	FALSE
0.7361 ± 0.0065	0.546420002	0.613826787	FALSE
0.7078 ± 0.0271	2.325867926	0.080616288	FALSE
0.7141 ± 0.0092	4.487381037	0.010927747	TRUE
0.7003 ± 0.0196	3.48545407	0.025226706	TRUE
0.7216 ± 0.0194	1.815682655	0.143595627	FALSE
0.7014 ± 0.0246	2.760645642	0.050815949	FALSE
0.7121 ± 0.0199	2.515407043	0.065677492	FALSE
0.7187 ± 0.0160	3.107742771	0.035950373	TRUE
0.7320 ± 0.0265	0.509814374	0.637022695	FALSE
0.7618 ± 0.0082	3.149221726	0.034539857	TRUE
0.7649 ± 0.0117	10.97226936	0.000392004	TRUE
0.7715 ± 0.0085	2.539961962	0.063983493	FALSE
0.7733 ± 0.0060	3.339111143	0.028859641	TRUE
0.7720 ± 0.0101	2.063501798	0.108022748	FALSE
0.7791 ± 0.0143	0.494760964	0.646711839	FALSE
0.7733 ± 0.0096	1.40071371	0.233903187	FALSE
0.7652 ± 0.0126	3.961125599	0.016661935	TRUE
0.7805 ± 0.0122	0.738914609	0.500970169	FALSE
0.7625 ± 0.0103	4.337894345	0.012273241	TRUE
0.7810 ± 0.0128	0.196724451	0.853634285	FALSE
0.7770 ± 0.0063	0.924074795	0.40774649	FALSE
0.7725 ± 0.0159	0.836929914	0.449719933	FALSE
0.7743 ± 0.0084	1.253003653	0.278453471	FALSE
0.5897 ± 0.0111	0.902578951	0.417787182	FALSE
0.5632 ± 0.0142	3.663993635	0.021504525	TRUE
0.5905 ± 0.0174	0.563118941	0.603421211	FALSE
0.5696 ± 0.0124	3.577342891	0.02322275	TRUE
0.5906 ± 0.0175	0.561829851	0.604220485	FALSE
0.5722 ± 0.0105	2.864773617	0.045708806	TRUE
0.5877 ± 0.0135	0.920727676	0.409296533	FALSE
0.5665 ± 0.0066	7.65703563	0.001563407	TRUE
0.5918 ± 0.0169	0.593278367	0.584914692	FALSE
0.5690 ± 0.0190	2.191984802	0.093484745	FALSE
0.5895 ± 0.0203	0.703731804	0.520406623	FALSE
0.5819 ± 0.0122	2.216979397	0.090917172	FALSE
0.5865 ± 0.0111	1.182537587	0.302481917	FALSE

0.5876 ± 0.0144	1.013063282	0.36832914	FALSE
0.6037 ± 0.0084	1.87738657	0.133684407	FALSE
0.6222 ± 0.0224	0.400090165	0.709535955	FALSE
0.6199 ± 0.0065	0.868719379	0.434017723	FALSE
0.6192 ± 0.0168	1.084497126	0.339138156	FALSE
0.6150 ± 0.0146	1.943702669	0.123849446	FALSE
0.6035 ± 0.0185	5.572100183	0.005083207	TRUE
0.6153 ± 0.0143	0.878368936	0.42934038	FALSE
0.6094 ± 0.0154	2.094235362	0.104330237	FALSE
0.6257 ± 0.0150	0.040582713	0.969573404	FALSE
0.6187 ± 0.0158	2.929421093	0.04283855	TRUE
0.6084 ± 0.0166	2.563535434	0.062404075	FALSE
0.6180 ± 0.0127	0.861938968	0.437329102	FALSE
0.5915 ± 0.0097	4.022975427	0.015825352	TRUE
0.6044 ± 0.0160	1.655507572	0.173163504	FALSE

Compared Subspace AUC	t_stat	p_value	Significance
0.6187 ± 0.0282	2.610376896	0.059396459	FALSE
0.6234 ± 0.0231	2.013292379	0.114368747	FALSE
0.6226 ± 0.0169	2.642134666	0.057451992	FALSE
0.6378 ± 0.0155	1.486507735	0.211342825	FALSE
0.6326 ± 0.0186	2.731599279	0.052356586	FALSE
0.6579 ± 0.0183	0.03206259	0.975958206	FALSE
0.6426 ± 0.0197	1.343019931	0.25040713	FALSE
0.6532 ± 0.0217	0.638600096	0.557814701	FALSE
0.6258 ± 0.0169	2.680437931	0.055203456	FALSE
0.6563 ± 0.0187	0.284562308	0.790103774	FALSE
0.6331 ± 0.0137	2.35072802	0.078452932	FALSE
0.6410 ± 0.0230	1.793182414	0.14740341	FALSE
0.6462 ± 0.0210	1.391112147	0.236573259	FALSE
0.6384 ± 0.0211	2.785360159	0.049546293	TRUE
0.7463 ± 0.0097	5.662668198	0.004794775	TRUE
0.7624 ± 0.0140	1.969818466	0.120194507	FALSE
0.7507 ± 0.0096	4.463697178	0.01112855	TRUE
0.7740 ± 0.0140	1.619436813	0.180666652	FALSE
0.7550 ± 0.0191	5.434302971	0.005563984	TRUE
0.7574 ± 0.0123	9.10007833	0.00080871	TRUE
0.7628 ± 0.0187	2.797142027	0.048953988	TRUE
0.7547 ± 0.0058	3.783586057	0.019375746	TRUE
0.7590 ± 0.0232	3.949711316	0.01682208	TRUE
0.7553 ± 0.0197	3.73706156	0.02017254	TRUE
0.7763 ± 0.0209	1.22915293	0.286376535	FALSE
0.7676 ± 0.0196	3.008170371	0.039621673	TRUE

0.7731 ± 0.0216	0.931392193	0.404374987	FALSE
0.7703 ± 0.0262	1.252842552	0.278506279	FALSE
0.6117 ± 0.0250	1.685897272	0.167094925	FALSE
0.6023 ± 0.0228	1.559737413	0.193834568	FALSE
0.6066 ± 0.0244	2.92779409	0.042908161	TRUE
0.5963 ± 0.0130	2.234539289	0.089160468	FALSE
0.6117 ± 0.0272	2.173514841	0.09543406	FALSE
0.6154 ± 0.0244	0.604231712	0.578286235	FALSE
0.6026 ± 0.0184	2.271918423	0.085546199	FALSE
0.6189 ± 0.0240	0.613445482	0.572749238	FALSE
0.5981 ± 0.0219	3.448974261	0.026078889	TRUE
0.6097 ± 0.0252	2.082320544	0.105744954	FALSE
0.6123 ± 0.0186	1.014439811	0.367746265	FALSE
0.6147 ± 0.0252	2.307555526	0.082252905	FALSE
0.5898 ± 0.0187	3.789942535	0.019269832	TRUE
0.5994 ± 0.0266	4.512934415	0.010715995	TRUE
0.5461 ± 0.0228	2.252873837	0.087366706	FALSE
0.5650 ± 0.0133	4.121140023	0.014598868	TRUE
0.5697 ± 0.0218	0.142985116	0.89321549	FALSE
0.5706 ± 0.0234	0.013571257	0.989821948	FALSE
0.5511 ± 0.0074	2.904726049	0.043909461	TRUE
0.5696 ± 0.0159	0.110276396	0.917501577	FALSE
0.5602 ± 0.0104	1.951463739	0.12275079	FALSE
0.5611 ± 0.0182	1.616580707	0.181275034	FALSE
0.5616 ± 0.0201	1.008000064	0.370480062	FALSE
0.5627 ± 0.0157	1.878761191	0.133472173	FALSE
0.5571 ± 0.0103	2.120518033	0.101282966	FALSE
0.5679 ± 0.0066	0.641715055	0.555984006	FALSE
0.5188 ± 0.0258	3.663946746	0.021505413	TRUE
0.5462 ± 0.0152	3.189735349	0.033224619	TRUE
