# Supplementary material for: Using Speech Features and Machine Learning Models to Predict Emotional and Behavioral Problems in Chinese Adolescents
Source: Depress Anxiety. 2025 Jun 16;2025:5734107. doi: 10.1155/da/5734107 (PMC12185205; doi:10.1155/da/5734107)
Supplement: Supporting Information 1 — The supporting information file includes all additional figures and tables referenced in the main text as Appendix A–G. [file 5734107.f1.zip › Appendix D.pdf]

Gender	Symptom	Friedman Test (χ², p-value)	Significant Difference Models	AUC (95% CI) - LR	AUC (95% CI) - SVM	AUC (95% CI) - GBDT	Cohen's d (LR vs GBDT)	Cohen's d (SVM vs GBDT)
Female	Emotional Symptoms	$\chi^2(2) = 7.6$, $p = 0.0224$	GBDT > LR, GBDT > SVM	0.6012 [0.5908, 0.6155]	0.6024 [0.5972, 0.6114]	0.6581 [0.6410, 0.6724]	-3.0635	-3.4882
Female	Peer Problems	$\chi^2(2) = 3.6$, $p = 0.1653$	No significant difference	0.5464 [0.5257, 0.5707]	0.5440 [0.5215, 0.5697]	0.5707 [0.5607, 0.5826]	N/A	N/A
Female	Hyperactivity	$\chi^2(2) = 8.4$, $p = 0.0150$	GBDT > LR, GBDT > SVM, SVM > LR	0.7068 [0.6946, 0.7171]	0.7080 [0.6977, 0.7166]	0.7801 [0.7671, 0.7928]	-4.5079	-4.7871
Female	Conduct Problems	$\chi^2(2) = 2.8$, $p = 0.2466$	No significant difference	0.5915 [0.5609, 0.6222]	0.5930 [0.5603, 0.6257]	0.6218 [0.5988, 0.6463]	N/A	N/A
Male	Emotional Symptoms	$\chi^2(2) = 7.6$, $p = 0.0224$	GBDT > LR, GBDT > SVM	0.6365 [0.6248, 0.6468]	0.6358 [0.6246, 0.6470]	0.7383 [0.7343, 0.7450]	-9.1510	-8.9087
Male	Hyperactivity	$\chi^2(2) = 8.4$, $p = 0.0150$	GBDT > LR, GBDT > SVM, SVM > LR	0.7202 [0.7034, 0.7371]	0.7214 [0.7059, 0.7370]	0.7826 [0.7731, 0.7927]	-3.4987	-3.6151
Male	Conduct Problems	$\chi^2(2) = 7.6$, $p = 0.0224$	GBDT > LR, GBDT > SVM	0.5459 [0.5275, 0.5584]	0.5416 [0.5329, 0.5528]	0.5974 [0.5878, 0.6081]	-3.0195	-4.3649
Male	Peer Problems	$\chi^2(2) = 10.0$, $p = 0.0067$	GBDT > LR, GBDT > SVM, SVM > LR	0.5654 [0.5496, 0.5822]	0.5632 [0.5478, 0.5791]	0.6258 [0.6077, 0.6393]	-2.9028	-3.0426
