# Supplementary material for: Using Speech Features and Machine Learning Models to Predict Emotional and Behavioral Problems in Chinese Adolescents
Source: Depress Anxiety. 2025 Jun 16;2025:5734107. doi: 10.1155/da/5734107 (PMC12185205; doi:10.1155/da/5734107)
Supplement: Supporting Information 1 — The supporting information file includes all additional figures and tables referenced in the main text as Appendix A–G. [file 5734107.f1.zip › Appendix E.pdf]

GBDT_Female_detailed_performance_results							
**Symptom**	**Subspace**	**Fold**	**AUC**	**F1**	**Sensitivity**	**Specificity**	**Precision**
**Emotional Symptoms**	MFCC	Mean ± Std	0.6579 ± 0.0183	0.6310 ± 0.0079	0.6690 ± 0.0255	0.5488 ± 0.0384	0.5978 ± 0.0129
**Emotional Symptoms**	MFCC	1	0.6235	0.6165	0.6414	0.5606	0.5935
**Emotional Symptoms**	MFCC	2	0.6700	0.6389	0.6768	0.5581	0.6050
**Emotional Symptoms**	MFCC	3	0.6761	0.6326	0.6717	0.5480	0.5978
**Emotional Symptoms**	MFCC	4	0.6597	0.6296	0.6439	0.5975	0.6159
**Emotional Symptoms**	MFCC	5	0.6600	0.6372	0.7114	0.4798	0.5770
**Emotional Symptoms**	MELS	Mean ± Std	0.6234 ± 0.0231	0.6055 ± 0.0144	0.6382 ± 0.0125	0.5295 ± 0.0458	0.5766 ± 0.0231
**Emotional Symptoms**	MELS	1	0.6437	0.6192	0.6364	0.5808	0.6029
**Emotional Symptoms**	MELS	2	0.5956	0.5968	0.6540	0.4621	0.5487
**Emotional Symptoms**	MELS	3	0.6374	0.6073	0.6288	0.5581	0.5873
**Emotional Symptoms**	MELS	4	0.5951	0.5829	0.6212	0.4886	0.5491
**Emotional Symptoms**	MELS	5	0.6453	0.6215	0.6506	0.5581	0.5949
**Emotional Symptoms**	PROS	Mean ± Std	0.6462 ± 0.0210	0.6187 ± 0.0189	0.6428 ± 0.0233	0.5650 ± 0.0335	0.5968 ± 0.0215
**Emotional Symptoms**	PROS	1	0.6195	0.5854	0.6061	0.5354	0.5660
**Emotional Symptoms**	PROS	2	0.6303	0.6268	0.6616	0.5505	0.5955
**Emotional Symptoms**	PROS	3	0.6580	0.6139	0.6263	0.5859	0.6019
**Emotional Symptoms**	PROS	4	0.6792	0.6418	0.6515	0.6203	0.6324
**Emotional Symptoms**	PROS	5	0.6441	0.6256	0.6684	0.5328	0.5880
**Emotional Symptoms**	FORM	Mean ± Std	0.6187 ± 0.0282	0.6087 ± 0.0154	0.6448 ± 0.0178	0.5260 ± 0.0303	0.5766 ± 0.0175
**Emotional Symptoms**	FORM	1	0.6032	0.6012	0.6263	0.5429	0.5781
**Emotional Symptoms**	FORM	2	0.5863	0.5940	0.6465	0.4697	0.5494
**Emotional Symptoms**	FORM	3	0.6434	0.6201	0.6616	0.5278	0.5835
**Emotional Symptoms**	FORM	4	0.6604	0.6331	0.6667	0.5595	0.6027
**Emotional Symptoms**	FORM	5	0.6004	0.5949	0.6228	0.5303	0.5694
**Emotional Symptoms**	MFCC+MELS	Mean ± Std	0.6532 ± 0.0217	0.6255 ± 0.0156	0.6564 ± 0.0206	0.5579 ± 0.0255	0.5977 ± 0.0158
**Emotional Symptoms**	MFCC+MELS	1	0.6155	0.5947	0.6187	0.5379	0.5724
**Emotional Symptoms**	MFCC+MELS	2	0.6535	0.6301	0.6818	0.5177	0.5857
**Emotional Symptoms**	MFCC+MELS	3	0.6744	0.6359	0.6616	0.5808	0.6121
**Emotional Symptoms**	MFCC+MELS	4	0.6476	0.6327	0.6591	0.5747	0.6084
**Emotional Symptoms**	MFCC+MELS	5	0.6748	0.6343	0.6608	0.5783	0.6098
**Emotional Symptoms**	MFCC+PROS	Mean ± Std	0.6581 ± 0.0181	0.6319 ± 0.0137	0.6620 ± 0.0165	0.5670 ± 0.0228	0.6047 ± 0.0152
**Emotional Symptoms**	MFCC+PROS	1	0.6264	0.6163	0.6490	0.5429	0.5868
**Emotional Symptoms**	MFCC+PROS	2	0.6612	0.6245	0.6490	0.5707	0.6019
**Emotional Symptoms**	MFCC+PROS	3	0.6525	0.6230	0.6490	0.5657	0.5991
**Emotional Symptoms**	MFCC+PROS	4	0.6732	0.6528	0.6742	0.6076	0.6327
**Emotional Symptoms**	MFCC+PROS	5	0.6772	0.6430	0.6886	0.5480	0.6031
**Emotional Symptoms**	MFCC+FORM	Mean ± Std	0.6426 ± 0.0197	0.6084 ± 0.0073	0.6331 ± 0.0129	0.5518 ± 0.0284	0.5859 ± 0.0127
**Emotional Symptoms**	MFCC+FORM	1	0.6271	0.6017	0.6313	0.5328	0.5747
**Emotional Symptoms**	MFCC+FORM	2	0.6155	0.6010	0.6389	0.5126	0.5673
**Emotional Symptoms**	MFCC+FORM	3	0.6716	0.6146	0.6364	0.5657	0.5943
**Emotional Symptoms**	MFCC+FORM	4	0.6540	0.6193	0.6490	0.5519	0.5922
**Emotional Symptoms**	MFCC+FORM	5	0.6446	0.6055	0.6101	0.5960	0.6010
**Emotional Symptoms**	MELS+PROS	Mean ± Std	0.6378 ± 0.0155	0.6153 ± 0.0089	0.6392 ± 0.0142	0.5614 ± 0.0332	0.5936 ± 0.0158
**Emotional Symptoms**	MELS+PROS	1	0.6466	0.6248	0.6414	0.5884	0.6091
**Emotional Symptoms**	MELS+PROS	2	0.6113	0.6033	0.6414	0.5152	0.5695
**Emotional Symptoms**	MELS+PROS	3	0.6562	0.6138	0.6162	0.6086	0.6115
**Emotional Symptoms**	MELS+PROS	4	0.6311	0.6087	0.6364	0.5443	0.5833
**Emotional Symptoms**	MELS+PROS	5	0.6436	0.6259	0.6608	0.5505	0.5945
**Emotional Symptoms**	MELS+FORM	Mean ± Std	0.6226 ± 0.0169	0.5905 ± 0.0129	0.6044 ± 0.0160	0.5574 ± 0.0288	0.5776 ± 0.0158
**Emotional Symptoms**	MELS+FORM	1	0.6331	0.5928	0.6010	0.5732	0.5848
**Emotional Symptoms**	MELS+FORM	2	0.5933	0.5772	0.6086	0.5000	0.5490
**Emotional Symptoms**	MELS+FORM	3	0.6324	0.5857	0.5909	0.5732	0.5806
**Emotional Symptoms**	MELS+FORM	4	0.6144	0.5825	0.5884	0.5671	0.5767
**Emotional Symptoms**	MELS+FORM	5	0.6397	0.6143	0.6329	0.5732	0.5967
**Emotional Symptoms**	PROS+FORM	Mean ± Std	0.6384 ± 0.0211	0.6023 ± 0.0093	0.6235 ± 0.0217	0.5533 ± 0.0401	0.5834 ± 0.0164

**Emotional Symptoms**	PROS+FORM	1	0.6053	0.5877	0.6136	0.5253	0.5638
**Emotional Symptoms**	PROS+FORM	2	0.6236	0.6132	0.6667	0.4924	0.5677
**Emotional Symptoms**	PROS+FORM	3	0.6540	0.6002	0.6162	0.5631	0.5851
**Emotional Symptoms**	PROS+FORM	4	0.6632	0.6113	0.6136	0.6051	0.6090
**Emotional Symptoms**	PROS+FORM	5	0.6459	0.5993	0.6076	0.5808	0.5911
**Emotional Symptoms**	MFCC+MELS+PROS	Mean ± Std	0.6563 ± 0.0187	0.6201 ± 0.0163	0.6423 ± 0.0288	0.5715 ± 0.0302	0.6001 ± 0.0138
**Emotional Symptoms**	MFCC+MELS+PROS	1	0.6351	0.6100	0.6338	0.5556	0.5878
**Emotional Symptoms**	MFCC+MELS+PROS	2	0.6513	0.6228	0.6692	0.5202	0.5824
**Emotional Symptoms**	MFCC+MELS+PROS	3	0.6513	0.5964	0.5934	0.6035	0.5995
**Emotional Symptoms**	MFCC+MELS+PROS	4	0.6523	0.6264	0.6414	0.5924	0.6120
**Emotional Symptoms**	MFCC+MELS+PROS	5	0.6915	0.6448	0.6734	0.5859	0.6186
**Emotional Symptoms**	MFCC+MELS+FORM	Mean ± Std	0.6258 ± 0.0169	0.5879 ± 0.0186	0.5973 ± 0.0240	0.5659 ± 0.0193	0.5791 ± 0.0158
**Emotional Symptoms**	MFCC+MELS+FORM	1	0.6269	0.5909	0.6035	0.5606	0.5787
**Emotional Symptoms**	MFCC+MELS+FORM	2	0.6005	0.5819	0.6010	0.5354	0.5640
**Emotional Symptoms**	MFCC+MELS+FORM	3	0.6383	0.5775	0.5833	0.5631	0.5718
**Emotional Symptoms**	MFCC+MELS+FORM	4	0.6148	0.5674	0.5631	0.5772	0.5718
**Emotional Symptoms**	MFCC+MELS+FORM	5	0.6485	0.6221	0.6354	0.5934	0.6092
**Emotional Symptoms**	MFCC+PROS+FORM	Mean ± Std	0.6410 ± 0.0230	0.6048 ± 0.0161	0.6195 ± 0.0219	0.5710 ± 0.0263	0.5910 ± 0.0163
**Emotional Symptoms**	MFCC+PROS+FORM	1	0.6123	0.5831	0.5934	0.5581	0.5732
**Emotional Symptoms**	MFCC+PROS+FORM	2	0.6147	0.5942	0.6212	0.5303	0.5694
**Emotional Symptoms**	MFCC+PROS+FORM	3	0.6635	0.6078	0.6086	0.6061	0.6071
**Emotional Symptoms**	MFCC+PROS+FORM	4	0.6641	0.6312	0.6591	0.5696	0.6056
**Emotional Symptoms**	MFCC+PROS+FORM	5	0.6503	0.6075	0.6152	0.5909	0.6000
**Emotional Symptoms**	MELS+PROS+FORM	Mean ± Std	0.6326 ± 0.0186	0.6059 ± 0.0120	0.6190 ± 0.0139	0.5756 ± 0.0308	0.5937 ± 0.0170
**Emotional Symptoms**	MELS+PROS+FORM	1	0.6223	0.6091	0.6237	0.5758	0.5952
**Emotional Symptoms**	MELS+PROS+FORM	2	0.6109	0.5930	0.6237	0.5202	0.5652
**Emotional Symptoms**	MELS+PROS+FORM	3	0.6356	0.6049	0.6187	0.5732	0.5918
**Emotional Symptoms**	MELS+PROS+FORM	4	0.6282	0.5957	0.5934	0.6000	0.5980
**Emotional Symptoms**	MELS+PROS+FORM	5	0.6662	0.6267	0.6354	0.6086	0.6182
**Emotional Symptoms**	MFCC+MELS+PROS+	Mean ± Std	0.6331 ± 0.0137	0.5972 ± 0.0049	0.6099 ± 0.0050	0.5675 ± 0.0127	0.5851 ± 0.0074
**Emotional Symptoms**	MFCC+MELS+PROS+	1	0.6276	0.6005	0.6111	0.5758	0.5902
**Emotional Symptoms**	MFCC+MELS+PROS+	2	0.6148	0.5900	0.6086	0.5455	0.5724
**Emotional Symptoms**	MFCC+MELS+PROS+	3	0.6511	0.6042	0.6187	0.5707	0.5904
**Emotional Symptoms**	MFCC+MELS+PROS+	4	0.6252	0.5975	0.6035	0.5823	0.5916
**Emotional Symptoms**	MFCC+MELS+PROS+	5	0.6469	0.5941	0.6076	0.5631	0.5811
**Hyperactivity**	MFCC	Mean ± Std	0.7801 ± 0.0157	0.7218 ± 0.0189	0.7637 ± 0.0267	0.6480 ± 0.0268	0.6847 ± 0.0185
**Hyperactivity**	MFCC	1	0.7973	0.7373	0.7767	0.6693	0.7018
**Hyperactivity**	MFCC	2	0.7961	0.7407	0.7723	0.6877	0.7117
**Hyperactivity**	MFCC	3	0.7748	0.7284	0.7941	0.6139	0.6728
**Hyperactivity**	MFCC	4	0.7773	0.7138	0.7604	0.6297	0.6725
**Hyperactivity**	MFCC	5	0.7548	0.6889	0.7149	0.6396	0.6648
**Hyperactivity**	MELS	Mean ± Std	0.7624 ± 0.0140	0.7170 ± 0.0192	0.7613 ± 0.0301	0.6382 ± 0.0252	0.6780 ± 0.0167
**Hyperactivity**	MELS	1	0.7858	0.7458	0.7885	0.6733	0.7074
**Hyperactivity**	MELS	2	0.7464	0.6994	0.7465	0.6126	0.6579
**Hyperactivity**	MELS	3	0.7702	0.7337	0.8020	0.6158	0.6761
**Hyperactivity**	MELS	4	0.7537	0.7064	0.7505	0.6257	0.6673
**Hyperactivity**	MELS	5	0.7557	0.6994	0.7188	0.6634	0.6811
**Hyperactivity**	PROS	Mean ± Std	0.7731 ± 0.0216	0.7227 ± 0.0198	0.7708 ± 0.0236	0.6378 ± 0.0234	0.6804 ± 0.0188
**Hyperactivity**	PROS	1	0.7673	0.7126	0.7569	0.6317	0.6731
**Hyperactivity**	PROS	2	0.8003	0.7448	0.7861	0.6759	0.7077
**Hyperactivity**	PROS	3	0.7734	0.7274	0.7901	0.6178	0.6740
**Hyperactivity**	PROS	4	0.7879	0.7389	0.7901	0.6515	0.6939
**Hyperactivity**	PROS	5	0.7365	0.6897	0.7307	0.6119	0.6531
**Hyperactivity**	FORM	Mean ± Std	0.7463 ± 0.0097	0.7017 ± 0.0104	0.7522 ± 0.0210	0.6085 ± 0.0185	0.6578 ± 0.0087
**Hyperactivity**	FORM	1	0.7413	0.7017	0.7391	0.6317	0.6679
**Hyperactivity**	FORM	2	0.7627	0.7151	0.7703	0.6166	0.6672
**Hyperactivity**	FORM	3	0.7487	0.7110	0.7842	0.5782	0.6502

**Hyperactivity**	FORM	4	0.7455	0.6929	0.7327	0.6178	0.6572
**Hyperactivity**	FORM	5	0.7334	0.6877	0.7347	0.5980	0.6463
**Hyperactivity**	MFCC+MELS	Mean ± Std	0.7628 ± 0.0187	0.7021 ± 0.0065	0.7356 ± 0.0176	0.6401 ± 0.0313	0.6721 ± 0.0154
**Hyperactivity**	MFCC+MELS	1	0.7880	0.6997	0.7115	0.6772	0.6883
**Hyperactivity**	MFCC+MELS	2	0.7788	0.7122	0.7327	0.6759	0.6929
**Hyperactivity**	MFCC+MELS	3	0.7356	0.7035	0.7564	0.6059	0.6575
**Hyperactivity**	MFCC+MELS	4	0.7592	0.7030	0.7545	0.6079	0.6580
**Hyperactivity**	MFCC+MELS	5	0.7523	0.6919	0.7228	0.6337	0.6636
**Hyperactivity**	MFCC+PROS	Mean ± Std	0.7763 ± 0.0209	0.7188 ± 0.0184	0.7625 ± 0.0176	0.6405 ± 0.0311	0.6800 ± 0.0217
**Hyperactivity**	MFCC+PROS	1	0.8040	0.7319	0.7688	0.6673	0.6984
**Hyperactivity**	MFCC+PROS	2	0.7939	0.7425	0.7822	0.6759	0.7066
**Hyperactivity**	MFCC+PROS	3	0.7643	0.7104	0.7723	0.5980	0.6577
**Hyperactivity**	MFCC+PROS	4	0.7738	0.7199	0.7584	0.6515	0.6852
**Hyperactivity**	MFCC+PROS	5	0.7454	0.6891	0.7307	0.6099	0.6519
**Hyperactivity**	MFCC+FORM	Mean ± Std	0.7574 ± 0.0123	0.7022 ± 0.0114	0.7435 ± 0.0108	0.6255 ± 0.0304	0.6655 ± 0.0181
**Hyperactivity**	MFCC+FORM	1	0.7753	0.7212	0.7490	0.6713	0.6954
**Hyperactivity**	MFCC+FORM	2	0.7647	0.7049	0.7426	0.6364	0.6708
**Hyperactivity**	MFCC+FORM	3	0.7552	0.6982	0.7604	0.5822	0.6454
**Hyperactivity**	MFCC+FORM	4	0.7537	0.7006	0.7366	0.6337	0.6679
**Hyperactivity**	MFCC+FORM	5	0.7382	0.6859	0.7287	0.6040	0.6479
**Hyperactivity**	MELS+PROS	Mean ± Std	0.7740 ± 0.0140	0.7218 ± 0.0173	0.7652 ± 0.0257	0.6453 ± 0.0171	0.6832 ± 0.0137
**Hyperactivity**	MELS+PROS	1	0.7895	0.7369	0.7806	0.6614	0.6979
**Hyperactivity**	MELS+PROS	2	0.7778	0.7266	0.7604	0.6680	0.6957
**Hyperactivity**	MELS+PROS	3	0.7782	0.7377	0.8020	0.6277	0.6830
**Hyperactivity**	MELS+PROS	4	0.7771	0.7172	0.7584	0.6436	0.6803
**Hyperactivity**	MELS+PROS	5	0.7475	0.6906	0.7248	0.6257	0.6595
**Hyperactivity**	MELS+FORM	Mean ± Std	0.7507 ± 0.0096	0.6959 ± 0.0078	0.7363 ± 0.0171	0.6203 ± 0.0192	0.6600 ± 0.0084
**Hyperactivity**	MELS+FORM	1	0.7678	0.7077	0.7490	0.6317	0.6708
**Hyperactivity**	MELS+FORM	2	0.7434	0.6919	0.7248	0.6304	0.6618
**Hyperactivity**	MELS+FORM	3	0.7539	0.6994	0.7624	0.5822	0.6460
**Hyperactivity**	MELS+FORM	4	0.7472	0.6962	0.7307	0.6317	0.6649
**Hyperactivity**	MELS+FORM	5	0.7412	0.6844	0.7149	0.6257	0.6564
**Hyperactivity**	PROS+FORM	Mean ± Std	0.7703 ± 0.0262	0.7179 ± 0.0183	0.7633 ± 0.0136	0.6362 ± 0.0377	0.6778 ± 0.0246
**Hyperactivity**	PROS+FORM	1	0.7915	0.7257	0.7609	0.6634	0.6937
**Hyperactivity**	PROS+FORM	2	0.7907	0.7358	0.7723	0.6739	0.7027
**Hyperactivity**	PROS+FORM	3	0.7441	0.7011	0.7663	0.5802	0.6461
**Hyperactivity**	PROS+FORM	4	0.7921	0.7353	0.7782	0.6614	0.6968
**Hyperactivity**	PROS+FORM	5	0.7329	0.6914	0.7386	0.6020	0.6498
**Hyperactivity**	MFCC+MELS+PROS	Mean ± Std	0.7590 ± 0.0232	0.7037 ± 0.0185	0.7490 ± 0.0203	0.6199 ± 0.0322	0.6638 ± 0.0218
**Hyperactivity**	MFCC+MELS+PROS	1	0.7920	0.7224	0.7510	0.6713	0.6960
**Hyperactivity**	MFCC+MELS+PROS	2	0.7754	0.7111	0.7505	0.6403	0.6756
**Hyperactivity**	MFCC+MELS+PROS	3	0.7368	0.7049	0.7663	0.5921	0.6526
**Hyperactivity**	MFCC+MELS+PROS	4	0.7607	0.7114	0.7663	0.6119	0.6638
**Hyperactivity**	MFCC+MELS+PROS	5	0.7301	0.6685	0.7109	0.5842	0.6309
**Hyperactivity**	MFCC+MELS+FORM	Mean ± Std	0.7547 ± 0.0058	0.7038 ± 0.0094	0.7427 ± 0.0213	0.6326 ± 0.0181	0.6692 ± 0.0076
**Hyperactivity**	MFCC+MELS+FORM	1	0.7663	0.7067	0.7451	0.6356	0.6720
**Hyperactivity**	MFCC+MELS+FORM	2	0.7513	0.6988	0.7168	0.6660	0.6817
**Hyperactivity**	MFCC+MELS+FORM	3	0.7517	0.7203	0.7802	0.6139	0.6689
**Hyperactivity**	MFCC+MELS+FORM	4	0.7532	0.6924	0.7287	0.6238	0.6595
**Hyperactivity**	MFCC+MELS+FORM	5	0.7513	0.7009	0.7426	0.6238	0.6637
**Hyperactivity**	MFCC+PROS+FORM	Mean ± Std	0.7676 ± 0.0196	0.7063 ± 0.0116	0.7391 ± 0.0096	0.6461 ± 0.0257	0.6765 ± 0.0169
**Hyperactivity**	MFCC+PROS+FORM	1	0.7974	0.7247	0.7490	0.6812	0.7019
**Hyperactivity**	MFCC+PROS+FORM	2	0.7816	0.7096	0.7307	0.6719	0.6897
**Hyperactivity**	MFCC+PROS+FORM	3	0.7493	0.6922	0.7327	0.6158	0.6560
**Hyperactivity**	MFCC+PROS+FORM	4	0.7641	0.7096	0.7525	0.6317	0.6714
**Hyperactivity**	MFCC+PROS+FORM	5	0.7456	0.6956	0.7307	0.6297	0.6637
**Hyperactivity**	MELS+PROS+FORM	Mean ± Std	0.7550 ± 0.0191	0.7012 ± 0.0140	0.7371 ± 0.0181	0.6346 ± 0.0210	0.6687 ± 0.0144

**Hyperactivity**	MELS+PROS+FORM	1	0.7822	0.7249	0.7708	0.6436	0.6842
**Hyperactivity**	MELS+PROS+FORM	2	0.7612	0.7003	0.7287	0.6482	0.6740
**Hyperactivity**	MELS+PROS+FORM	3	0.7400	0.6889	0.7366	0.5980	0.6470
**Hyperactivity**	MELS+PROS+FORM	4	0.7637	0.7061	0.7327	0.6574	0.6814
**Hyperactivity**	MELS+PROS+FORM	5	0.7278	0.6856	0.7168	0.6257	0.6570
**Hyperactivity**	MFCC+MELS+PROS+	Mean ± Std	0.7553 ± 0.0197	0.6997 ± 0.0194	0.7355 ± 0.0247	0.6334 ± 0.0225	0.6674 ± 0.0178
**Hyperactivity**	MFCC+MELS+PROS+	1	0.7927	0.7368	0.7747	0.6713	0.7025
**Hyperactivity**	MFCC+MELS+PROS+	2	0.7551	0.6820	0.7030	0.6423	0.6623
**Hyperactivity**	MFCC+MELS+PROS+	3	0.7394	0.6999	0.7505	0.6059	0.6557
**Hyperactivity**	MFCC+MELS+PROS+	4	0.7499	0.6880	0.7248	0.6178	0.6547
**Hyperactivity**	MFCC+MELS+PROS+	5	0.7394	0.6919	0.7248	0.6297	0.6618
**Conduct Problems**	MFCC	Mean ± Std	0.6154 ± 0.0244	0.5933 ± 0.0230	0.6094 ± 0.0312	0.5558 ± 0.0185	0.5782 ± 0.0176
**Conduct Problems**	MFCC	1	0.6202	0.5911	0.5935	0.5865	0.5887
**Conduct Problems**	MFCC	2	0.5965	0.5764	0.5865	0.5501	0.5666
**Conduct Problems**	MFCC	3	0.5828	0.5642	0.5772	0.5312	0.5518
**Conduct Problems**	MFCC	4	0.6235	0.6042	0.6287	0.5474	0.5815
**Conduct Problems**	MFCC	5	0.6537	0.6305	0.6612	0.5637	0.6025
**Conduct Problems**	MELS	Mean ± Std	0.6023 ± 0.0228	0.5831 ± 0.0303	0.6051 ± 0.0422	0.5314 ± 0.0278	0.5633 ± 0.0232
**Conduct Problems**	MELS	1	0.5874	0.5565	0.5610	0.5459	0.5520
**Conduct Problems**	MELS	2	0.5952	0.5959	0.6297	0.5149	0.5655
**Conduct Problems**	MELS	3	0.5793	0.5469	0.5691	0.4878	0.5263
**Conduct Problems**	MELS	4	0.6052	0.5845	0.5908	0.5691	0.5782
**Conduct Problems**	MELS	5	0.6445	0.6320	0.6748	0.5393	0.5943
**Conduct Problems**	PROS	Mean ± Std	0.5898 ± 0.0187	0.5977 ± 0.0180	0.6300 ± 0.0285	0.5227 ± 0.0093	0.5687 ± 0.0099
**Conduct Problems**	PROS	1	0.5658	0.5673	0.5827	0.5297	0.5527
**Conduct Problems**	PROS	2	0.5700	0.5891	0.6162	0.5230	0.5644
**Conduct Problems**	PROS	3	0.5954	0.6068	0.6504	0.5068	0.5687
**Conduct Problems**	PROS	4	0.6111	0.6057	0.6369	0.5339	0.5774
**Conduct Problems**	PROS	5	0.6069	0.6195	0.6640	0.5203	0.5806
**Conduct Problems**	FORM	Mean ± Std	0.6117 ± 0.0250	0.6097 ± 0.0237	0.6463 ± 0.0321	0.5271 ± 0.0214	0.5773 ± 0.0185
**Conduct Problems**	FORM	1	0.6055	0.5982	0.6314	0.5216	0.5683
**Conduct Problems**	FORM	2	0.5911	0.5985	0.6405	0.4986	0.5616
**Conduct Problems**	FORM	3	0.5837	0.5816	0.5989	0.5393	0.5652
**Conduct Problems**	FORM	4	0.6529	0.6506	0.6938	0.5610	0.6124
**Conduct Problems**	FORM	5	0.6251	0.6196	0.6667	0.5149	0.5788
**Conduct Problems**	MFCC+MELS	Mean ± Std	0.6026 ± 0.0184	0.5876 ± 0.0125	0.6018 ± 0.0183	0.5536 ± 0.0136	0.5741 ± 0.0092
**Conduct Problems**	MFCC+MELS	1	0.5846	0.5730	0.5745	0.5703	0.5714
**Conduct Problems**	MFCC+MELS	2	0.5853	0.5785	0.5973	0.5312	0.5609
**Conduct Problems**	MFCC+MELS	3	0.5952	0.5839	0.5989	0.5474	0.5696
**Conduct Problems**	MFCC+MELS	4	0.6165	0.5942	0.6070	0.5637	0.5818
**Conduct Problems**	MFCC+MELS	5	0.6313	0.6084	0.6314	0.5556	0.5869
**Conduct Problems**	MFCC+PROS	Mean ± Std	0.6123 ± 0.0186	0.5911 ± 0.0163	0.6121 ± 0.0259	0.5417 ± 0.0140	0.5717 ± 0.0092
**Conduct Problems**	MFCC+PROS	1	0.5982	0.5629	0.5637	0.5622	0.5622
**Conduct Problems**	MFCC+PROS	2	0.5967	0.5893	0.6108	0.5366	0.5693
**Conduct Problems**	MFCC+PROS	3	0.6003	0.5891	0.6179	0.5203	0.5630
**Conduct Problems**	MFCC+PROS	4	0.6216	0.6104	0.6369	0.5501	0.5860
**Conduct Problems**	MFCC+PROS	5	0.6449	0.6036	0.6314	0.5393	0.5782
**Conduct Problems**	MFCC+FORM	Mean ± Std	0.6218 ± 0.0278	0.5994 ± 0.0201	0.6208 ± 0.0235	0.5493 ± 0.0281	0.5795 ± 0.0199
**Conduct Problems**	MFCC+FORM	1	0.6154	0.5955	0.6043	0.5757	0.5868
**Conduct Problems**	MFCC+FORM	2	0.5806	0.5703	0.5865	0.5285	0.5550
**Conduct Problems**	MFCC+FORM	3	0.6095	0.5882	0.6233	0.5041	0.5569
**Conduct Problems**	MFCC+FORM	4	0.6611	0.6268	0.6531	0.5691	0.6025
**Conduct Problems**	MFCC+FORM	5	0.6426	0.6160	0.6369	0.5691	0.5964
**Conduct Problems**	MELS+PROS	Mean ± Std	0.5963 ± 0.0130	0.5882 ± 0.0116	0.6143 ± 0.0124	0.5255 ± 0.0276	0.5645 ± 0.0153
**Conduct Problems**	MELS+PROS	1	0.5816	0.5783	0.6152	0.4892	0.5457
**Conduct Problems**	MELS+PROS	2	0.5878	0.5806	0.6135	0.4986	0.5510
**Conduct Problems**	MELS+PROS	3	0.5939	0.5812	0.6016	0.5312	0.5620

**Conduct Problems**	MELS+PROS	4	0.5987	0.5915	0.6043	0.5610	0.5792
**Conduct Problems**	MELS+PROS	5	0.6197	0.6096	0.6369	0.5474	0.5846
**Conduct Problems**	MELS+FORM	Mean ± Std	0.6066 ± 0.0244	0.5914 ± 0.0154	0.6105 ± 0.0190	0.5460 ± 0.0194	0.5736 ± 0.0142
**Conduct Problems**	MELS+FORM	1	0.5925	0.5971	0.6125	0.5622	0.5825
**Conduct Problems**	MELS+FORM	2	0.5774	0.5803	0.6054	0.5176	0.5572
**Conduct Problems**	MELS+FORM	3	0.5918	0.5680	0.5772	0.5447	0.5591
**Conduct Problems**	MELS+FORM	4	0.6319	0.6113	0.6287	0.5718	0.5949
**Conduct Problems**	MELS+FORM	5	0.6392	0.6003	0.6287	0.5339	0.5743
**Conduct Problems**	PROS+FORM	Mean ± Std	0.5994 ± 0.0266	0.5963 ± 0.0228	0.6279 ± 0.0206	0.5217 ± 0.0361	0.5680 ± 0.0258
**Conduct Problems**	PROS+FORM	1	0.5939	0.5881	0.6152	0.5243	0.5633
**Conduct Problems**	PROS+FORM	2	0.5720	0.5798	0.6135	0.4959	0.5496
**Conduct Problems**	PROS+FORM	3	0.5770	0.5751	0.6070	0.4959	0.5463
**Conduct Problems**	PROS+FORM	4	0.6460	0.6387	0.6612	0.5908	0.6177
**Conduct Problems**	PROS+FORM	5	0.6080	0.6000	0.6423	0.5014	0.5629
**Conduct Problems**	MFCC+MELS+PROS	Mean ± Std	0.5981 ± 0.0219	0.5944 ± 0.0179	0.6143 ± 0.0236	0.5477 ± 0.0133	0.5758 ± 0.0138
**Conduct Problems**	MFCC+MELS+PROS	1	0.5825	0.5672	0.5772	0.5432	0.5576
**Conduct Problems**	MFCC+MELS+PROS	2	0.5797	0.5899	0.6162	0.5257	0.5658
**Conduct Problems**	MFCC+MELS+PROS	3	0.5788	0.5865	0.6016	0.5501	0.5722
**Conduct Problems**	MFCC+MELS+PROS	4	0.6225	0.6115	0.6314	0.5664	0.5929
**Conduct Problems**	MFCC+MELS+PROS	5	0.6272	0.6166	0.6450	0.5528	0.5906
**Conduct Problems**	MFCC+MELS+FORM	Mean ± Std	0.6189 ± 0.0240	0.5991 ± 0.0166	0.6170 ± 0.0202	0.5574 ± 0.0125	0.5822 ± 0.0137
**Conduct Problems**	MFCC+MELS+FORM	1	0.6118	0.5896	0.6016	0.5622	0.5781
**Conduct Problems**	MFCC+MELS+FORM	2	0.5814	0.5752	0.5892	0.5393	0.5619
**Conduct Problems**	MFCC+MELS+FORM	3	0.6119	0.5950	0.6152	0.5474	0.5761
**Conduct Problems**	MFCC+MELS+FORM	4	0.6400	0.6176	0.6369	0.5745	0.5995
**Conduct Problems**	MFCC+MELS+FORM	5	0.6493	0.6180	0.6423	0.5637	0.5955
**Conduct Problems**	MFCC+PROS+FORM	Mean ± Std	0.6147 ± 0.0252	0.5958 ± 0.0206	0.6181 ± 0.0269	0.5439 ± 0.0219	0.5753 ± 0.0174
**Conduct Problems**	MFCC+PROS+FORM	1	0.6012	0.5787	0.5881	0.5568	0.5696
**Conduct Problems**	MFCC+PROS+FORM	2	0.5827	0.5789	0.5946	0.5393	0.5641
**Conduct Problems**	MFCC+PROS+FORM	3	0.6052	0.5977	0.6341	0.5122	0.5652
**Conduct Problems**	MFCC+PROS+FORM	4	0.6557	0.6346	0.6612	0.5772	0.6100
**Conduct Problems**	MFCC+PROS+FORM	5	0.6289	0.5893	0.6125	0.5339	0.5678
**Conduct Problems**	MELS+PROS+FORM	Mean ± Std	0.6117 ± 0.0272	0.5980 ± 0.0223	0.6159 ± 0.0256	0.5563 ± 0.0236	0.5813 ± 0.0206
**Conduct Problems**	MELS+PROS+FORM	1	0.5929	0.5718	0.5772	0.5595	0.5665
**Conduct Problems**	MELS+PROS+FORM	2	0.5820	0.5870	0.6108	0.5285	0.5650
**Conduct Problems**	MELS+PROS+FORM	3	0.5944	0.5838	0.6043	0.5339	0.5646
**Conduct Problems**	MELS+PROS+FORM	4	0.6465	0.6324	0.6504	0.5935	0.6154
**Conduct Problems**	MELS+PROS+FORM	5	0.6427	0.6152	0.6369	0.5664	0.5949
**Conduct Problems**	MFCC+MELS+PROS+	Mean ± Std	0.6097 ± 0.0252	0.5827 ± 0.0217	0.5937 ± 0.0226	0.5558 ± 0.0230	0.5720 ± 0.0213
**Conduct Problems**	MFCC+MELS+PROS+	1	0.6026	0.5829	0.5908	0.5649	0.5752
**Conduct Problems**	MFCC+MELS+PROS+	2	0.5883	0.5710	0.5811	0.5447	0.5614
**Conduct Problems**	MFCC+MELS+PROS+	3	0.5833	0.5547	0.5637	0.5312	0.5459
**Conduct Problems**	MFCC+MELS+PROS+	4	0.6525	0.6205	0.6314	0.5962	0.6099
**Conduct Problems**	MFCC+MELS+PROS+	5	0.6219	0.5842	0.6016	0.5420	0.5678
**Peer Problems**	MFCC	Mean ± Std	0.5511 ± 0.0074	0.5377 ± 0.0163	0.5412 ± 0.0238	0.5291 ± 0.0210	0.5347 ± 0.0132
**Peer Problems**	MFCC	1	0.5467	0.5590	0.5818	0.5015	0.5378
**Peer Problems**	MFCC	2	0.5623	0.5540	0.5515	0.5619	0.5566
**Peer Problems**	MFCC	3	0.5462	0.5152	0.5136	0.5182	0.5167
**Peer Problems**	MFCC	4	0.5573	0.5305	0.5257	0.5424	0.5354
**Peer Problems**	MFCC	5	0.5431	0.5301	0.5333	0.5212	0.5269
**Peer Problems**	MELS	Mean ± Std	0.5707 ± 0.0126	0.5342 ± 0.0190	0.5097 ± 0.0272	0.6023 ± 0.0297	0.5620 ± 0.0172
**Peer Problems**	MELS	1	0.5667	0.5262	0.5182	0.5498	0.5344
**Peer Problems**	MELS	2	0.5584	0.5363	0.5152	0.5952	0.5592
**Peer Problems**	MELS	3	0.5755	0.5348	0.4985	0.6333	0.5769
**Peer Problems**	MELS	4	0.5930	0.5661	0.5498	0.6061	0.5833
**Peer Problems**	MELS	5	0.5601	0.5074	0.4667	0.6273	0.5560
**Peer Problems**	PROS	Mean ± Std	0.5188 ± 0.0258	0.5093 ± 0.0173	0.5121 ± 0.0236	0.5018 ± 0.0201	0.5068 ± 0.0142

**Peer Problems**	PROS	1	0.4742	0.4793	0.4727	0.5015	0.4860
**Peer Problems**	PROS	2	0.5374	0.5260	0.5364	0.4985	0.5160
**Peer Problems**	PROS	3	0.5501	0.5184	0.5106	0.5394	0.5265
**Peer Problems**	PROS	4	0.5158	0.5007	0.5045	0.4879	0.4970
**Peer Problems**	PROS	5	0.5166	0.5221	0.5364	0.4818	0.5086
**Peer Problems**	FORM	Mean ± Std	0.5461 ± 0.0228	0.5150 ± 0.0207	0.5006 ± 0.0274	0.5575 ± 0.0263	0.5308 ± 0.0183
**Peer Problems**	FORM	1	0.5704	0.5311	0.5182	0.5680	0.5446
**Peer Problems**	FORM	2	0.5028	0.4837	0.4727	0.5196	0.4952
**Peer Problems**	FORM	3	0.5559	0.5182	0.4955	0.5818	0.5430
**Peer Problems**	FORM	4	0.5500	0.5414	0.5438	0.5333	0.5389
**Peer Problems**	FORM	5	0.5516	0.5008	0.4727	0.5848	0.5324
**Peer Problems**	MFCC+MELS	Mean ± Std	0.5602 ± 0.0104	0.5258 ± 0.0212	0.4970 ± 0.0267	0.6078 ± 0.0139	0.5586 ± 0.0157
**Peer Problems**	MFCC+MELS	1	0.5589	0.5158	0.4939	0.5801	0.5397
**Peer Problems**	MFCC+MELS	2	0.5577	0.5399	0.5121	0.6163	0.5709
**Peer Problems**	MFCC+MELS	3	0.5461	0.4984	0.4592	0.6152	0.5448
**Peer Problems**	MFCC+MELS	4	0.5784	0.5589	0.5378	0.6121	0.5817
**Peer Problems**	MFCC+MELS	5	0.5599	0.5162	0.4818	0.6152	0.5559
**Peer Problems**	MFCC+PROS	Mean ± Std	0.5571 ± 0.0103	0.5473 ± 0.0089	0.5508 ± 0.0097	0.5381 ± 0.0081	0.5439 ± 0.0086
**Peer Problems**	MFCC+PROS	1	0.5389	0.5419	0.5485	0.5257	0.5355
**Peer Problems**	MFCC+PROS	2	0.5649	0.5400	0.5424	0.5347	0.5375
**Peer Problems**	MFCC+PROS	3	0.5631	0.5599	0.5650	0.5455	0.5549
**Peer Problems**	MFCC+PROS	4	0.5660	0.5564	0.5589	0.5485	0.5539
**Peer Problems**	MFCC+PROS	5	0.5527	0.5386	0.5394	0.5364	0.5378
**Peer Problems**	MFCC+FORM	Mean ± Std	0.5696 ± 0.0159	0.5428 ± 0.0117	0.5345 ± 0.0112	0.5648 ± 0.0317	0.5517 ± 0.0184
**Peer Problems**	MFCC+FORM	1	0.5705	0.5495	0.5303	0.6012	0.5700
**Peer Problems**	MFCC+FORM	2	0.5438	0.5351	0.5424	0.5166	0.5280
**Peer Problems**	MFCC+FORM	3	0.5819	0.5452	0.5287	0.5879	0.5627
**Peer Problems**	MFCC+FORM	4	0.5624	0.5252	0.5196	0.5394	0.5309
**Peer Problems**	MFCC+FORM	5	0.5895	0.5591	0.5515	0.5788	0.5670
**Peer Problems**	MELS+PROS	Mean ± Std	0.5697 ± 0.0218	0.5350 ± 0.0218	0.5139 ± 0.0212	0.5927 ± 0.0340	0.5583 ± 0.0266
**Peer Problems**	MELS+PROS	1	0.5480	0.4976	0.4788	0.5559	0.5180
**Peer Problems**	MELS+PROS	2	0.5438	0.5299	0.5242	0.5468	0.5356
**Peer Problems**	MELS+PROS	3	0.5770	0.5440	0.5136	0.6242	0.5782
**Peer Problems**	MELS+PROS	4	0.6035	0.5643	0.5438	0.6152	0.5863
**Peer Problems**	MELS+PROS	5	0.5765	0.5393	0.5091	0.6212	0.5734
**Peer Problems**	MELS+FORM	Mean ± Std	0.5650 ± 0.0133	0.5120 ± 0.0163	0.4794 ± 0.0264	0.6078 ± 0.0271	0.5502 ± 0.0129
**Peer Problems**	MELS+FORM	1	0.5633	0.4976	0.4697	0.5831	0.5290
**Peer Problems**	MELS+FORM	2	0.5525	0.5379	0.5273	0.5680	0.5489
**Peer Problems**	MELS+FORM	3	0.5647	0.5074	0.4683	0.6212	0.5536
**Peer Problems**	MELS+FORM	4	0.5899	0.5229	0.4834	0.6333	0.5694
**Peer Problems**	MELS+FORM	5	0.5548	0.4942	0.4485	0.6333	0.5502
**Peer Problems**	PROS+FORM	Mean ± Std	0.5462 ± 0.0152	0.5206 ± 0.0071	0.5061 ± 0.0070	0.5618 ± 0.0239	0.5362 ± 0.0131
**Peer Problems**	PROS+FORM	1	0.5520	0.5182	0.4970	0.5801	0.5413
**Peer Problems**	PROS+FORM	2	0.5162	0.5114	0.5091	0.5196	0.5138
**Peer Problems**	PROS+FORM	3	0.5558	0.5329	0.5136	0.5848	0.5537
**Peer Problems**	PROS+FORM	4	0.5507	0.5181	0.4985	0.5727	0.5392
**Peer Problems**	PROS+FORM	5	0.5564	0.5224	0.5121	0.5515	0.5331
**Peer Problems**	MFCC+MELS+PROS	Mean ± Std	0.5616 ± 0.0201	0.5252 ± 0.0222	0.5048 ± 0.0243	0.5829 ± 0.0162	0.5474 ± 0.0203
**Peer Problems**	MFCC+MELS+PROS	1	0.5531	0.5055	0.4848	0.5680	0.5281
**Peer Problems**	MFCC+MELS+PROS	2	0.5763	0.5429	0.5273	0.5861	0.5595
**Peer Problems**	MFCC+MELS+PROS	3	0.5383	0.5086	0.4894	0.5636	0.5294
**Peer Problems**	MFCC+MELS+PROS	4	0.5931	0.5603	0.5408	0.6091	0.5812
**Peer Problems**	MFCC+MELS+PROS	5	0.5473	0.5088	0.4818	0.5879	0.5390
**Peer Problems**	MFCC+MELS+FORM	Mean ± Std	0.5611 ± 0.0182	0.5151 ± 0.0268	0.4873 ± 0.0333	0.5969 ± 0.0208	0.5469 ± 0.0210
**Peer Problems**	MFCC+MELS+FORM	1	0.5635	0.5188	0.5030	0.5650	0.5355
**Peer Problems**	MFCC+MELS+FORM	2	0.5614	0.5183	0.4939	0.5891	0.5452
**Peer Problems**	MFCC+MELS+FORM	3	0.5490	0.4895	0.4562	0.5909	0.5280

**Peer Problems**	MFCC+MELS+FORM	4	0.5928	0.5615	0.5378	0.6212	0.5875
**Peer Problems**	MFCC+MELS+FORM	5	0.5389	0.4876	0.4455	0.6182	0.5385
**Peer Problems**	MFCC+PROS+FORM	Mean ± Std	0.5679 ± 0.0066	0.5324 ± 0.0114	0.5187 ± 0.0205	0.5708 ± 0.0183	0.5473 ± 0.0051
**Peer Problems**	MFCC+PROS+FORM	1	0.5706	0.5215	0.4970	0.5921	0.5485
**Peer Problems**	MFCC+PROS+FORM	2	0.5557	0.5382	0.5333	0.5529	0.5432
**Peer Problems**	MFCC+PROS+FORM	3	0.5723	0.5510	0.5468	0.5606	0.5552
**Peer Problems**	MFCC+PROS+FORM	4	0.5743	0.5315	0.5227	0.5545	0.5406
**Peer Problems**	MFCC+PROS+FORM	5	0.5667	0.5199	0.4939	0.5939	0.5488
**Peer Problems**	MELS+PROS+FORM	Mean ± Std	0.5706 ± 0.0234	0.5216 ± 0.0201	0.4927 ± 0.0222	0.6035 ± 0.0316	0.5546 ± 0.0239
**Peer Problems**	MELS+PROS+FORM	1	0.5551	0.5189	0.5000	0.5740	0.5392
**Peer Problems**	MELS+PROS+FORM	2	0.5617	0.5329	0.5152	0.5831	0.5519
**Peer Problems**	MELS+PROS+FORM	3	0.5567	0.5040	0.4773	0.5818	0.5338
**Peer Problems**	MELS+PROS+FORM	4	0.6171	0.5537	0.5136	0.6576	0.6007
**Peer Problems**	MELS+PROS+FORM	5	0.5626	0.4983	0.4576	0.6212	0.5471
**Peer Problems**	MFCC+MELS+PROS+	Mean ± Std	0.5627 ± 0.0157	0.5145 ± 0.0194	0.4861 ± 0.0297	0.5981 ± 0.0272	0.5475 ± 0.0135
**Peer Problems**	MFCC+MELS+PROS+	1	0.5629	0.5135	0.4909	0.5801	0.5382
**Peer Problems**	MFCC+MELS+PROS+	2	0.5508	0.5255	0.5152	0.5559	0.5363
**Peer Problems**	MFCC+MELS+PROS+	3	0.5511	0.4860	0.4441	0.6152	0.5365
**Peer Problems**	MFCC+MELS+PROS+	4	0.5930	0.5434	0.5196	0.6061	0.5695
**Peer Problems**	MFCC+MELS+PROS+	5	0.5558	0.5041	0.4606	0.6333	0.5568

GBDT_Male_detailed_performance_results

Symptom	Subspace	Fold	AUC	F1	Sensitivity	Specificity	Precision
**Emotional Symptoms**	MFCC	Mean ± Std	0.7361 ± 0.0065	0.6847 ± 0.0099	0.7047 ± 0.0178	0.6467 ± 0.0114	0.6661 ± 0.0063
**Emotional Symptoms**	MFCC	1	0.7309	0.6722	0.6822	0.6531	0.6625
**Emotional Symptoms**	MFCC	2	0.7375	0.6782	0.6914	0.6531	0.6655
**Emotional Symptoms**	MFCC	3	0.7349	0.6805	0.7013	0.6394	0.6608
**Emotional Symptoms**	MFCC	4	0.7477	0.6968	0.7161	0.6599	0.6784
**Emotional Symptoms**	MFCC	5	0.7293	0.6961	0.7323	0.6283	0.6633
**Emotional Symptoms**	MELS	Mean ± Std	0.7241 ± 0.0161	0.6723 ± 0.0135	0.7114 ± 0.0256	0.5955 ± 0.0239	0.6377 ± 0.0118
**Emotional Symptoms**	MELS	1	0.7190	0.6560	0.6840	0.5993	0.6301
**Emotional Symptoms**	MELS	2	0.7445	0.6787	0.6989	0.6401	0.6596
**Emotional Symptoms**	MELS	3	0.7075	0.6608	0.6994	0.5818	0.6262
**Emotional Symptoms**	MELS	4	0.7078	0.6719	0.7161	0.5836	0.6328
**Emotional Symptoms**	MELS	5	0.7417	0.6939	0.7584	0.5725	0.6395
**Emotional Symptoms**	PROS	Mean ± Std	0.7187 ± 0.0160	0.6827 ± 0.0149	0.7277 ± 0.0176	0.5958 ± 0.0238	0.6431 ± 0.0158
**Emotional Symptoms**	PROS	1	0.7106	0.6731	0.7100	0.6011	0.6399
**Emotional Symptoms**	PROS	2	0.7406	0.7117	0.7546	0.6345	0.6733
**Emotional Symptoms**	PROS	3	0.7017	0.6730	0.7254	0.5688	0.6276
**Emotional Symptoms**	PROS	4	0.7054	0.6731	0.7087	0.6022	0.6409
**Emotional Symptoms**	PROS	5	0.7351	0.6827	0.7398	0.5725	0.6338
**Emotional Symptoms**	FORM	Mean ± Std	0.7116 ± 0.0124	0.6709 ± 0.0135	0.7233 ± 0.0164	0.5672 ± 0.0223	0.6258 ± 0.0143
**Emotional Symptoms**	FORM	1	0.7070	0.6510	0.6970	0.5566	0.6107
**Emotional Symptoms**	FORM	2	0.7222	0.6808	0.7454	0.5566	0.6266
**Emotional Symptoms**	FORM	3	0.6892	0.6587	0.7180	0.5372	0.6085
**Emotional Symptoms**	FORM	4	0.7202	0.6851	0.7347	0.5892	0.6418
**Emotional Symptoms**	FORM	5	0.7195	0.6789	0.7212	0.5967	0.6413
**Emotional Symptoms**	MFCC+MELS	Mean ± Std	0.7141 ± 0.0092	0.6667 ± 0.0065	0.6973 ± 0.0163	0.6055 ± 0.0351	0.6393 ± 0.0162
**Emotional Symptoms**	MFCC+MELS	1	0.7244	0.6722	0.6803	0.6568	0.6642
**Emotional Symptoms**	MFCC+MELS	2	0.7172	0.6685	0.6952	0.6160	0.6437
**Emotional Symptoms**	MFCC+MELS	3	0.7189	0.6571	0.6790	0.6115	0.6365
**Emotional Symptoms**	MFCC+MELS	4	0.7127	0.6743	0.7143	0.5948	0.6385
**Emotional Symptoms**	MFCC+MELS	5	0.6972	0.6615	0.7175	0.5483	0.6137
**Emotional Symptoms**	MFCC+PROS	Mean ± Std	0.7383 ± 0.0067	0.6865 ± 0.0097	0.7099 ± 0.0172	0.6419 ± 0.0122	0.6647 ± 0.0068
**Emotional Symptoms**	MFCC+PROS	1	0.7332	0.6745	0.6914	0.6419	0.6584
**Emotional Symptoms**	MFCC+PROS	2	0.7515	0.7002	0.7249	0.6549	0.6771
**Emotional Symptoms**	MFCC+PROS	3	0.7356	0.6764	0.6865	0.6561	0.6667
**Emotional Symptoms**	MFCC+PROS	4	0.7362	0.6897	0.7217	0.6283	0.6604

**Emotional Symptoms**	MFCC+PROS	5	0.7351	0.6915	0.7249	0.6283	0.6610
**Emotional Symptoms**	MFCC+FORM	Mean ± Std	0.7078 ± 0.0271	0.6630 ± 0.0244	0.7028 ± 0.0278	0.5828 ± 0.0288	0.6276 ± 0.0232
**Emotional Symptoms**	MFCC+FORM	1	0.6970	0.6471	0.6747	0.5900	0.6216
**Emotional Symptoms**	MFCC+FORM	2	0.7196	0.6776	0.7305	0.5751	0.6318
**Emotional Symptoms**	MFCC+FORM	3	0.6612	0.6254	0.6660	0.5353	0.5895
**Emotional Symptoms**	MFCC+FORM	4	0.7404	0.6954	0.7328	0.6245	0.6616
**Emotional Symptoms**	MFCC+FORM	5	0.7210	0.6696	0.7100	0.5892	0.6335
**Emotional Symptoms**	MELS+PROS	Mean ± Std	0.7321 ± 0.0146	0.6858 ± 0.0148	0.7288 ± 0.0249	0.6036 ± 0.0185	0.6478 ± 0.0109
**Emotional Symptoms**	MELS+PROS	1	0.7206	0.6739	0.6970	0.6289	0.6522
**Emotional Symptoms**	MELS+PROS	2	0.7560	0.7112	0.7621	0.6197	0.6667
**Emotional Symptoms**	MELS+PROS	3	0.7300	0.6720	0.7069	0.6022	0.6403
**Emotional Symptoms**	MELS+PROS	4	0.7144	0.6782	0.7273	0.5818	0.6353
**Emotional Symptoms**	MELS+PROS	5	0.7393	0.6936	0.7509	0.5855	0.6443
**Emotional Symptoms**	MELS+FORM	Mean ± Std	0.7084 ± 0.0258	0.6645 ± 0.0257	0.7077 ± 0.0311	0.5780 ± 0.0312	0.6266 ± 0.0245
**Emotional Symptoms**	MELS+FORM	1	0.7051	0.6626	0.7026	0.5826	0.6269
**Emotional Symptoms**	MELS+FORM	2	0.6972	0.6814	0.7435	0.5622	0.6289
**Emotional Symptoms**	MELS+FORM	3	0.6687	0.6163	0.6512	0.5372	0.5850
**Emotional Symptoms**	MELS+FORM	4	0.7437	0.6898	0.7199	0.6320	0.6621
**Emotional Symptoms**	MELS+FORM	5	0.7273	0.6724	0.7212	0.5762	0.6299
**Emotional Symptoms**	PROS+FORM	Mean ± Std	0.7320 ± 0.0265	0.6803 ± 0.0179	0.7136 ± 0.0258	0.6159 ± 0.0306	0.6504 ± 0.0191
**Emotional Symptoms**	PROS+FORM	1	0.7338	0.6793	0.6989	0.6419	0.6608
**Emotional Symptoms**	PROS+FORM	2	0.7533	0.6942	0.7509	0.5881	0.6454
**Emotional Symptoms**	PROS+FORM	3	0.6814	0.6467	0.6809	0.5743	0.6158
**Emotional Symptoms**	PROS+FORM	4	0.7383	0.6854	0.7013	0.6543	0.6702
**Emotional Symptoms**	PROS+FORM	5	0.7532	0.6960	0.7361	0.6208	0.6600
**Emotional Symptoms**	MFCC+MELS+PROS	Mean ± Std	0.7216 ± 0.0194	0.6766 ± 0.0096	0.7136 ± 0.0141	0.6040 ± 0.0287	0.6435 ± 0.0151
**Emotional Symptoms**	MFCC+MELS+PROS	1	0.7267	0.6841	0.7063	0.6419	0.6632
**Emotional Symptoms**	MFCC+MELS+PROS	2	0.7331	0.6784	0.7156	0.6067	0.6449
**Emotional Symptoms**	MFCC+MELS+PROS	3	0.7343	0.6714	0.6957	0.6227	0.6488
**Emotional Symptoms**	MFCC+MELS+PROS	4	0.7305	0.6880	0.7384	0.5911	0.6440
**Emotional Symptoms**	MFCC+MELS+PROS	5	0.6832	0.6609	0.7119	0.5576	0.6167
**Emotional Symptoms**	MFCC+MELS+FORM	Mean ± Std	0.7003 ± 0.0196	0.6558 ± 0.0177	0.6924 ± 0.0244	0.5810 ± 0.0184	0.6230 ± 0.0149
**Emotional Symptoms**	MFCC+MELS+FORM	1	0.6938	0.6371	0.6673	0.5733	0.6095
**Emotional Symptoms**	MFCC+MELS+FORM	2	0.6915	0.6551	0.6989	0.5659	0.6164
**Emotional Symptoms**	MFCC+MELS+FORM	3	0.6719	0.6352	0.6623	0.5762	0.6103
**Emotional Symptoms**	MFCC+MELS+FORM	4	0.7258	0.6767	0.7069	0.6171	0.6491
**Emotional Symptoms**	MFCC+MELS+FORM	5	0.7187	0.6747	0.7268	0.5725	0.6296
**Emotional Symptoms**	MFCC+PROS+FORM	Mean ± Std	0.7121 ± 0.0199	0.6659 ± 0.0138	0.7002 ± 0.0145	0.5970 ± 0.0255	0.6349 ± 0.0165
**Emotional Symptoms**	MFCC+PROS+FORM	1	0.7152	0.6655	0.6896	0.6178	0.6430
**Emotional Symptoms**	MFCC+PROS+FORM	2	0.7134	0.6690	0.7156	0.5770	0.6281
**Emotional Symptoms**	MFCC+PROS+FORM	3	0.6744	0.6440	0.6846	0.5576	0.6079
**Emotional Symptoms**	MFCC+PROS+FORM	4	0.7301	0.6873	0.7199	0.6245	0.6576
**Emotional Symptoms**	MFCC+PROS+FORM	5	0.7273	0.6637	0.6914	0.6078	0.6381
**Emotional Symptoms**	MELS+PROS+FORM	Mean ± Std	0.7109 ± 0.0247	0.6707 ± 0.0274	0.7095 ± 0.0353	0.5944 ± 0.0260	0.6361 ± 0.0234
**Emotional Symptoms**	MELS+PROS+FORM	1	0.7104	0.6590	0.6896	0.5974	0.6310
**Emotional Symptoms**	MELS+PROS+FORM	2	0.7051	0.6833	0.7361	0.5826	0.6377
**Emotional Symptoms**	MELS+PROS+FORM	3	0.6697	0.6228	0.6494	0.5632	0.5983
**Emotional Symptoms**	MELS+PROS+FORM	4	0.7447	0.6998	0.7310	0.6413	0.6712
**Emotional Symptoms**	MELS+PROS+FORM	5	0.7244	0.6885	0.7416	0.5874	0.6425
**Emotional Symptoms**	MFCC+MELS+PROS+	Mean ± Std	0.7014 ± 0.0246	0.6556 ± 0.0160	0.6921 ± 0.0206	0.5810 ± 0.0193	0.6229 ± 0.0147
**Emotional Symptoms**	MFCC+MELS+PROS+	1	0.7026	0.6584	0.6896	0.5955	0.6299
**Emotional Symptoms**	MFCC+MELS+PROS+	2	0.6907	0.6469	0.6877	0.5622	0.6106
**Emotional Symptoms**	MFCC+MELS+PROS+	3	0.6611	0.6302	0.6623	0.5595	0.6010
**Emotional Symptoms**	MFCC+MELS+PROS+	4	0.7310	0.6661	0.6939	0.6097	0.6404
**Emotional Symptoms**	MFCC+MELS+PROS+	5	0.7216	0.6765	0.7268	0.5781	0.6327
**Hyperactivity**	MFCC	Mean ± Std	0.7791 ± 0.0143	0.7181 ± 0.0122	0.7522 ± 0.0175	0.6572 ± 0.0193	0.6871 ± 0.0132
**Hyperactivity**	MFCC	1	0.7542	0.6954	0.7299	0.6314	0.6641

**Hyperactivity**	MFCC	2	0.7977	0.7206	0.7372	0.6906	0.7047
**Hyperactivity**	MFCC	3	0.7775	0.7216	0.7556	0.6615	0.6906
**Hyperactivity**	MFCC	4	0.7866	0.7325	0.7795	0.6513	0.6909
**Hyperactivity**	MFCC	5	0.7794	0.7202	0.7590	0.6513	0.6852
**Hyperactivity**	MELS	Mean ± Std	0.7649 ± 0.0117	0.7118 ± 0.0155	0.7519 ± 0.0273	0.6398 ± 0.0081	0.6760 ± 0.0072
**Hyperactivity**	MELS	1	0.7469	0.6941	0.7197	0.6468	0.6704
**Hyperactivity**	MELS	2	0.7571	0.6981	0.7338	0.6308	0.6656
**Hyperactivity**	MELS	3	0.7706	0.7085	0.7436	0.6444	0.6765
**Hyperactivity**	MELS	4	0.7697	0.7221	0.7641	0.6479	0.6845
**Hyperactivity**	MELS	5	0.7804	0.7360	0.7983	0.6291	0.6827
**Hyperactivity**	PROS	Mean ± Std	0.7725 ± 0.0159	0.7143 ± 0.0104	0.7485 ± 0.0113	0.6528 ± 0.0207	0.6833 ± 0.0136
**Hyperactivity**	PROS	1	0.7799	0.7195	0.7521	0.6621	0.6897
**Hyperactivity**	PROS	2	0.7965	0.7292	0.7560	0.6821	0.7043
**Hyperactivity**	PROS	3	0.7498	0.6984	0.7265	0.6462	0.6725
**Hyperactivity**	PROS	4	0.7743	0.7162	0.7504	0.6547	0.6849
**Hyperactivity**	PROS	5	0.7622	0.7082	0.7573	0.6188	0.6652
**Hyperactivity**	FORM	Mean ± Std	0.7618 ± 0.0082	0.7027 ± 0.0033	0.7433 ± 0.0102	0.6278 ± 0.0102	0.6664 ± 0.0032
**Hyperactivity**	FORM	1	0.7694	0.7035	0.7402	0.6365	0.6703
**Hyperactivity**	FORM	2	0.7596	0.7085	0.7611	0.6120	0.6627
**Hyperactivity**	FORM	3	0.7508	0.6997	0.7368	0.6308	0.6662
**Hyperactivity**	FORM	4	0.7563	0.7026	0.7470	0.6205	0.6631
**Hyperactivity**	FORM	5	0.7729	0.6993	0.7316	0.6393	0.6698
**Hyperactivity**	MFCC+MELS	Mean ± Std	0.7826 ± 0.0110	0.7250 ± 0.0101	0.7628 ± 0.0200	0.6586 ± 0.0210	0.6910 ± 0.0112
**Hyperactivity**	MFCC+MELS	1	0.7664	0.7138	0.7590	0.6331	0.6737
**Hyperactivity**	MFCC+MELS	2	0.7779	0.7138	0.7321	0.6803	0.6964
**Hyperactivity**	MFCC+MELS	3	0.7871	0.7356	0.7658	0.6838	0.7077
**Hyperactivity**	MFCC+MELS	4	0.7816	0.7246	0.7624	0.6581	0.6904
**Hyperactivity**	MFCC+MELS	5	0.8001	0.7369	0.7949	0.6376	0.6869
**Hyperactivity**	MFCC+PROS	Mean ± Std	0.7810 ± 0.0128	0.7240 ± 0.0139	0.7601 ± 0.0170	0.6606 ± 0.0107	0.6913 ± 0.0114
**Hyperactivity**	MFCC+PROS	1	0.7730	0.7256	0.7641	0.6587	0.6909
**Hyperactivity**	MFCC+PROS	2	0.8004	0.7411	0.7816	0.6718	0.7046
**Hyperactivity**	MFCC+PROS	3	0.7894	0.7301	0.7675	0.6650	0.6961
**Hyperactivity**	MFCC+PROS	4	0.7634	0.6989	0.7299	0.6410	0.6703
**Hyperactivity**	MFCC+PROS	5	0.7789	0.7244	0.7573	0.6667	0.6944
**Hyperactivity**	MFCC+FORM	Mean ± Std	0.7733 ± 0.0096	0.7137 ± 0.0103	0.7471 ± 0.0114	0.6535 ± 0.0124	0.6831 ± 0.0102
**Hyperactivity**	MFCC+FORM	1	0.7685	0.7039	0.7333	0.6502	0.6767
**Hyperactivity**	MFCC+FORM	2	0.7889	0.7233	0.7560	0.6650	0.6933
**Hyperactivity**	MFCC+FORM	3	0.7692	0.7137	0.7436	0.6598	0.6861
**Hyperactivity**	MFCC+FORM	4	0.7611	0.7007	0.7385	0.6308	0.6667
**Hyperactivity**	MFCC+FORM	5	0.7788	0.7268	0.7641	0.6615	0.6930
**Hyperactivity**	MELS+PROS	Mean ± Std	0.7733 ± 0.0060	0.7137 ± 0.0059	0.7492 ± 0.0166	0.6500 ± 0.0137	0.6817 ± 0.0043
**Hyperactivity**	MELS+PROS	1	0.7665	0.7126	0.7419	0.6604	0.6856
**Hyperactivity**	MELS+PROS	2	0.7711	0.7058	0.7287	0.6632	0.6843
**Hyperactivity**	MELS+PROS	3	0.7741	0.7144	0.7590	0.6342	0.6748
**Hyperactivity**	MELS+PROS	4	0.7709	0.7116	0.7402	0.6598	0.6851
**Hyperactivity**	MELS+PROS	5	0.7842	0.7241	0.7761	0.6325	0.6786
**Hyperactivity**	MELS+FORM	Mean ± Std	0.7715 ± 0.0085	0.7039 ± 0.0061	0.7303 ± 0.0058	0.6552 ± 0.0138	0.6794 ± 0.0089
**Hyperactivity**	MELS+FORM	1	0.7672	0.7133	0.7402	0.6655	0.6884
**Hyperactivity**	MELS+FORM	2	0.7747	0.7074	0.7304	0.6650	0.6859
**Hyperactivity**	MELS+FORM	3	0.7642	0.6967	0.7265	0.6410	0.6693
**Hyperactivity**	MELS+FORM	4	0.7648	0.6982	0.7316	0.6359	0.6677
**Hyperactivity**	MELS+FORM	5	0.7868	0.7038	0.7231	0.6684	0.6856
**Hyperactivity**	PROS+FORM	Mean ± Std	0.7743 ± 0.0084	0.7149 ± 0.0116	0.7512 ± 0.0197	0.6500 ± 0.0131	0.6822 ± 0.0085
**Hyperactivity**	PROS+FORM	1	0.7734	0.7137	0.7350	0.6758	0.6935
**Hyperactivity**	PROS+FORM	2	0.7860	0.7326	0.7833	0.6444	0.6882
**Hyperactivity**	PROS+FORM	3	0.7633	0.6962	0.7265	0.6393	0.6682
**Hyperactivity**	PROS+FORM	4	0.7676	0.7168	0.7573	0.6444	0.6805

**Hyperactivity**	PROS+FORM	5	0.7809	0.7153	0.7538	0.6462	0.6806
**Hyperactivity**	MFCC+MELS+PROS	Mean ± Std	0.7805 ± 0.0122	0.7232 ± 0.0081	0.7604 ± 0.0084	0.6576 ± 0.0133	0.6896 ± 0.0096
**Hyperactivity**	MFCC+MELS+PROS	1	0.7587	0.7086	0.7504	0.6331	0.6713
**Hyperactivity**	MFCC+MELS+PROS	2	0.7816	0.7219	0.7509	0.6701	0.6951
**Hyperactivity**	MFCC+MELS+PROS	3	0.7923	0.7313	0.7675	0.6684	0.6983
**Hyperactivity**	MFCC+MELS+PROS	4	0.7785	0.7240	0.7624	0.6564	0.6893
**Hyperactivity**	MFCC+MELS+PROS	5	0.7915	0.7304	0.7709	0.6598	0.6938
**Hyperactivity**	MFCC+MELS+FORM	Mean ± Std	0.7652 ± 0.0126	0.7036 ± 0.0133	0.7334 ± 0.0183	0.6487 ± 0.0228	0.6763 ± 0.0151
**Hyperactivity**	MFCC+MELS+FORM	1	0.7473	0.6806	0.6974	0.6485	0.6645
**Hyperactivity**	MFCC+MELS+FORM	2	0.7760	0.7217	0.7389	0.6906	0.7052
**Hyperactivity**	MFCC+MELS+FORM	3	0.7579	0.7021	0.7453	0.6222	0.6636
**Hyperactivity**	MFCC+MELS+FORM	4	0.7626	0.7053	0.7385	0.6444	0.6750
**Hyperactivity**	MFCC+MELS+FORM	5	0.7824	0.7083	0.7470	0.6376	0.6733
**Hyperactivity**	MFCC+PROS+FORM	Mean ± Std	0.7770 ± 0.0063	0.7113 ± 0.0105	0.7471 ± 0.0139	0.6466 ± 0.0096	0.6789 ± 0.0087
**Hyperactivity**	MFCC+PROS+FORM	1	0.7711	0.7095	0.7453	0.6451	0.6770
**Hyperactivity**	MFCC+PROS+FORM	2	0.7889	0.7296	0.7713	0.6564	0.6922
**Hyperactivity**	MFCC+PROS+FORM	3	0.7757	0.7131	0.7436	0.6581	0.6850
**Hyperactivity**	MFCC+PROS+FORM	4	0.7724	0.6978	0.7282	0.6410	0.6698
**Hyperactivity**	MFCC+PROS+FORM	5	0.7770	0.7065	0.7470	0.6325	0.6702
**Hyperactivity**	MELS+PROS+FORM	Mean ± Std	0.7720 ± 0.0101	0.7031 ± 0.0081	0.7262 ± 0.0122	0.6606 ± 0.0125	0.6816 ± 0.0081
**Hyperactivity**	MELS+PROS+FORM	1	0.7662	0.6942	0.7043	0.6758	0.6844
**Hyperactivity**	MELS+PROS+FORM	2	0.7772	0.7168	0.7406	0.6735	0.6944
**Hyperactivity**	MELS+PROS+FORM	3	0.7586	0.6968	0.7248	0.6444	0.6709
**Hyperactivity**	MELS+PROS+FORM	4	0.7696	0.7007	0.7282	0.6496	0.6751
**Hyperactivity**	MELS+PROS+FORM	5	0.7884	0.7073	0.7333	0.6598	0.6831
**Hyperactivity**	MFCC+MELS+PROS+	Mean ± Std	0.7625 ± 0.0103	0.7013 ± 0.0058	0.7303 ± 0.0087	0.6476 ± 0.0121	0.6746 ± 0.0074
**Hyperactivity**	MFCC+MELS+PROS+	1	0.7498	0.6949	0.7162	0.6553	0.6747
**Hyperactivity**	MFCC+MELS+PROS+	2	0.7690	0.7112	0.7355	0.6667	0.6885
**Hyperactivity**	MFCC+MELS+PROS+	3	0.7512	0.6962	0.7265	0.6393	0.6682
**Hyperactivity**	MFCC+MELS+PROS+	4	0.7666	0.7034	0.7419	0.6325	0.6687
**Hyperactivity**	MFCC+MELS+PROS+	5	0.7761	0.7011	0.7316	0.6444	0.6730
**Conduct Problems**	MFCC	Mean ± Std	0.5722 ± 0.0105	0.5743 ± 0.0203	0.5996 ± 0.0280	0.5119 ± 0.0312	0.5514 ± 0.0188
**Conduct Problems**	MFCC	1	0.5615	0.5680	0.5891	0.5149	0.5484
**Conduct Problems**	MFCC	2	0.5781	0.5797	0.5941	0.5434	0.5660
**Conduct Problems**	MFCC	3	0.5682	0.5589	0.5693	0.5310	0.5489
**Conduct Problems**	MFCC	4	0.5635	0.5539	0.5931	0.4530	0.5196
**Conduct Problems**	MFCC	5	0.5898	0.6109	0.6526	0.5173	0.5742
**Conduct Problems**	MELS	Mean ± Std	0.5632 ± 0.0142	0.5596 ± 0.0111	0.5778 ± 0.0131	0.5129 ± 0.0200	0.5427 ± 0.0121
**Conduct Problems**	MELS	1	0.5470	0.5444	0.5693	0.4777	0.5215
**Conduct Problems**	MELS	2	0.5755	0.5735	0.5891	0.5335	0.5587
**Conduct Problems**	MELS	3	0.5679	0.5519	0.5594	0.5310	0.5446
**Conduct Problems**	MELS	4	0.5460	0.5577	0.5757	0.5124	0.5408
**Conduct Problems**	MELS	5	0.5798	0.5707	0.5955	0.5099	0.5479
**Conduct Problems**	PROS	Mean ± Std	0.5974 ± 0.0116	0.5998 ± 0.0111	0.6353 ± 0.0149	0.5169 ± 0.0216	0.5682 ± 0.0124
**Conduct Problems**	PROS	1	0.6006	0.5974	0.6262	0.5297	0.5711
**Conduct Problems**	PROS	2	0.6170	0.6112	0.6361	0.5533	0.5881
**Conduct Problems**	PROS	3	0.5947	0.5986	0.6386	0.5037	0.5633
**Conduct Problems**	PROS	4	0.5937	0.6108	0.6600	0.5000	0.5684
**Conduct Problems**	PROS	5	0.5812	0.5808	0.6154	0.4975	0.5499
**Conduct Problems**	FORM	Mean ± Std	0.5897 ± 0.0111	0.5937 ± 0.0237	0.6388 ± 0.0434	0.4891 ± 0.0271	0.5554 ± 0.0121
**Conduct Problems**	FORM	1	0.5710	0.5502	0.5693	0.5000	0.5324
**Conduct Problems**	FORM	2	0.5987	0.5887	0.6163	0.5211	0.5633
**Conduct Problems**	FORM	3	0.5846	0.6016	0.6411	0.5087	0.5667
**Conduct Problems**	FORM	4	0.5920	0.6150	0.6898	0.4480	0.5549
**Conduct Problems**	FORM	5	0.6023	0.6128	0.6774	0.4678	0.5594
**Conduct Problems**	MFCC+MELS	Mean ± Std	0.5665 ± 0.0066	0.5602 ± 0.0120	0.5729 ± 0.0245	0.5288 ± 0.0211	0.5487 ± 0.0033
**Conduct Problems**	MFCC+MELS	1	0.5597	0.5653	0.5891	0.5050	0.5434

**Conduct Problems**	MFCC+MELS	2	0.5781	0.5620	0.5718	0.5360	0.5526
**Conduct Problems**	MFCC+MELS	3	0.5693	0.5379	0.5272	0.5658	0.5490
**Conduct Problems**	MFCC+MELS	4	0.5635	0.5738	0.5980	0.5149	0.5515
**Conduct Problems**	MFCC+MELS	5	0.5618	0.5621	0.5782	0.5223	0.5469
**Conduct Problems**	MFCC+PROS	Mean ± Std	0.5819 ± 0.0122	0.5699 ± 0.0083	0.5897 ± 0.0046	0.5198 ± 0.0240	0.5515 ± 0.0134
**Conduct Problems**	MFCC+PROS	1	0.5891	0.5673	0.5891	0.5124	0.5471
**Conduct Problems**	MFCC+PROS	2	0.5936	0.5735	0.5842	0.5459	0.5632
**Conduct Problems**	MFCC+PROS	3	0.5772	0.5814	0.5965	0.5434	0.5671
**Conduct Problems**	MFCC+PROS	4	0.5602	0.5559	0.5856	0.4802	0.5291
**Conduct Problems**	MFCC+PROS	5	0.5896	0.5711	0.5931	0.5173	0.5507
**Conduct Problems**	MFCC+FORM	Mean ± Std	0.5877 ± 0.0135	0.5764 ± 0.0236	0.6051 ± 0.0522	0.5090 ± 0.0517	0.5525 ± 0.0136
**Conduct Problems**	MFCC+FORM	1	0.5672	0.5498	0.5668	0.5050	0.5338
**Conduct Problems**	MFCC+FORM	2	0.5873	0.5590	0.5569	0.5633	0.5611
**Conduct Problems**	MFCC+FORM	3	0.6015	0.5658	0.5644	0.5682	0.5672
**Conduct Problems**	MFCC+FORM	4	0.5794	0.5940	0.6625	0.4332	0.5383
**Conduct Problems**	MFCC+FORM	5	0.6030	0.6133	0.6749	0.4752	0.5620
**Conduct Problems**	MELS+PROS	Mean ± Std	0.5696 ± 0.0124	0.5643 ± 0.0119	0.5808 ± 0.0125	0.5223 ± 0.0164	0.5488 ± 0.0124
**Conduct Problems**	MELS+PROS	1	0.5514	0.5500	0.5718	0.4926	0.5298
**Conduct Problems**	MELS+PROS	2	0.5847	0.5800	0.5965	0.5385	0.5644
**Conduct Problems**	MELS+PROS	3	0.5739	0.5614	0.5718	0.5335	0.5513
**Conduct Problems**	MELS+PROS	4	0.5594	0.5538	0.5682	0.5173	0.5401
**Conduct Problems**	MELS+PROS	5	0.5787	0.5762	0.5955	0.5297	0.5581
**Conduct Problems**	MELS+FORM	Mean ± Std	0.5905 ± 0.0174	0.5855 ± 0.0227	0.6170 ± 0.0389	0.5110 ± 0.0438	0.5583 ± 0.0209
**Conduct Problems**	MELS+FORM	1	0.5684	0.5524	0.5743	0.4950	0.5321
**Conduct Problems**	MELS+FORM	2	0.5836	0.5662	0.5767	0.5385	0.5561
**Conduct Problems**	MELS+FORM	3	0.6192	0.6024	0.6114	0.5806	0.5938
**Conduct Problems**	MELS+FORM	4	0.5821	0.5937	0.6526	0.4554	0.5445
**Conduct Problems**	MELS+FORM	5	0.5995	0.6129	0.6700	0.4851	0.5649
**Conduct Problems**	PROS+FORM	Mean ± Std	0.5876 ± 0.0144	0.5837 ± 0.0330	0.6215 ± 0.0531	0.4956 ± 0.0274	0.5513 ± 0.0200
**Conduct Problems**	PROS+FORM	1	0.5642	0.5220	0.5297	0.5000	0.5144
**Conduct Problems**	PROS+FORM	2	0.6001	0.5979	0.6238	0.5360	0.5740
**Conduct Problems**	PROS+FORM	3	0.5814	0.5785	0.6064	0.5087	0.5530
**Conduct Problems**	PROS+FORM	4	0.5877	0.6078	0.6749	0.4554	0.5528
**Conduct Problems**	PROS+FORM	5	0.6046	0.6124	0.6725	0.4777	0.5622
**Conduct Problems**	MFCC+MELS+PROS	Mean ± Std	0.5690 ± 0.0190	0.5605 ± 0.0096	0.5733 ± 0.0074	0.5273 ± 0.0288	0.5485 ± 0.0154
**Conduct Problems**	MFCC+MELS+PROS	1	0.5482	0.5519	0.5792	0.4802	0.5270
**Conduct Problems**	MFCC+MELS+PROS	2	0.5734	0.5638	0.5743	0.5360	0.5537
**Conduct Problems**	MFCC+MELS+PROS	3	0.5716	0.5633	0.5668	0.5533	0.5599
**Conduct Problems**	MFCC+MELS+PROS	4	0.5507	0.5483	0.5633	0.5099	0.5341
**Conduct Problems**	MFCC+MELS+PROS	5	0.6010	0.5753	0.5831	0.5569	0.5676
**Conduct Problems**	MFCC+MELS+FORM	Mean ± Std	0.5918 ± 0.0169	0.5748 ± 0.0126	0.5986 ± 0.0329	0.5169 ± 0.0467	0.5542 ± 0.0143
**Conduct Problems**	MFCC+MELS+FORM	1	0.5650	0.5602	0.5817	0.5050	0.5402
**Conduct Problems**	MFCC+MELS+FORM	2	0.6056	0.5878	0.6089	0.5360	0.5681
**Conduct Problems**	MFCC+MELS+FORM	3	0.6128	0.5591	0.5446	0.5955	0.5744
**Conduct Problems**	MFCC+MELS+FORM	4	0.5827	0.5864	0.6402	0.4579	0.5409
**Conduct Problems**	MFCC+MELS+FORM	5	0.5928	0.5804	0.6179	0.4901	0.5473
**Conduct Problems**	MFCC+PROS+FORM	Mean ± Std	0.5865 ± 0.0111	0.5784 ± 0.0201	0.6135 ± 0.0408	0.4941 ± 0.0442	0.5484 ± 0.0156
**Conduct Problems**	MFCC+PROS+FORM	1	0.5767	0.5424	0.5619	0.4901	0.5242
**Conduct Problems**	MFCC+PROS+FORM	2	0.5898	0.5763	0.5842	0.5558	0.5687
**Conduct Problems**	MFCC+PROS+FORM	3	0.5878	0.5786	0.6015	0.5211	0.5573
**Conduct Problems**	MFCC+PROS+FORM	4	0.5733	0.5976	0.6725	0.4233	0.5377
**Conduct Problems**	MFCC+PROS+FORM	5	0.6047	0.5973	0.6476	0.4802	0.5541
**Conduct Problems**	MELS+PROS+FORM	Mean ± Std	0.5906 ± 0.0175	0.5830 ± 0.0205	0.6105 ± 0.0331	0.5169 ± 0.0468	0.5590 ± 0.0229
**Conduct Problems**	MELS+PROS+FORM	1	0.5632	0.5437	0.5619	0.4950	0.5267
**Conduct Problems**	MELS+PROS+FORM	2	0.5857	0.5852	0.5990	0.5509	0.5721
**Conduct Problems**	MELS+PROS+FORM	3	0.6166	0.5943	0.5965	0.5881	0.5921
**Conduct Problems**	MELS+PROS+FORM	4	0.5881	0.5889	0.6452	0.4554	0.5417

**Conduct Problems**	MELS+PROS+FORM	5	0.5993	0.6030	0.6501	0.4950	0.5622
**Conduct Problems**	MFCC+MELS+PROS+	Mean ± Std	0.5895 ± 0.0203	0.5792 ± 0.0141	0.6001 ± 0.0294	0.5288 ± 0.0422	0.5608 ± 0.0165
**Conduct Problems**	MFCC+MELS+PROS+	1	0.5602	0.5541	0.5644	0.5272	0.5442
**Conduct Problems**	MFCC+MELS+PROS+	2	0.5944	0.5935	0.6089	0.5558	0.5788
**Conduct Problems**	MFCC+MELS+PROS+	3	0.6215	0.5747	0.5668	0.5931	0.5827
**Conduct Problems**	MFCC+MELS+PROS+	4	0.5939	0.5899	0.6352	0.4827	0.5505
**Conduct Problems**	MFCC+MELS+PROS+	5	0.5774	0.5840	0.6253	0.4851	0.5478
**Peer Problems**	MFCC	Mean ± Std	0.6035 ± 0.0185	0.5564 ± 0.0214	0.5354 ± 0.0284	0.6117 ± 0.0400	0.5803 ± 0.0244
**Peer Problems**	MFCC	1	0.5883	0.5721	0.5694	0.5778	0.5748
**Peer Problems**	MFCC	2	0.6044	0.5301	0.4871	0.6486	0.5815
**Peer Problems**	MFCC	3	0.6266	0.5774	0.5542	0.6353	0.6026
**Peer Problems**	MFCC	4	0.5778	0.5305	0.5236	0.5506	0.5375
**Peer Problems**	MFCC	5	0.6203	0.5721	0.5425	0.6462	0.6053
**Peer Problems**	MELS	Mean ± Std	0.6222 ± 0.0224	0.5748 ± 0.0249	0.5523 ± 0.0273	0.6310 ± 0.0212	0.5994 ± 0.0235
**Peer Problems**	MELS	1	0.6211	0.5899	0.5788	0.6156	0.6015
**Peer Problems**	MELS	2	0.6063	0.5506	0.5247	0.6179	0.5792
**Peer Problems**	MELS	3	0.6216	0.5690	0.5448	0.6306	0.5954
**Peer Problems**	MELS	4	0.5986	0.5495	0.5236	0.6188	0.5781
**Peer Problems**	MELS	5	0.6635	0.6150	0.5896	0.6722	0.6427
**Peer Problems**	PROS	Mean ± Std	0.5915 ± 0.0097	0.5510 ± 0.0168	0.5387 ± 0.0194	0.5839 ± 0.0139	0.5641 ± 0.0149
**Peer Problems**	PROS	1	0.5889	0.5608	0.5482	0.5920	0.5739
**Peer Problems**	PROS	2	0.5858	0.5239	0.5035	0.5802	0.5459
**Peer Problems**	PROS	3	0.5858	0.5560	0.5448	0.5859	0.5676
**Peer Problems**	PROS	4	0.5863	0.5418	0.5354	0.5600	0.5483
**Peer Problems**	PROS	5	0.6108	0.5728	0.5613	0.6014	0.5848
**Peer Problems**	FORM	Mean ± Std	0.6037 ± 0.0084	0.5486 ± 0.0071	0.5321 ± 0.0112	0.5924 ± 0.0196	0.5664 ± 0.0100
**Peer Problems**	FORM	1	0.6104	0.5515	0.5294	0.6085	0.5754
**Peer Problems**	FORM	2	0.5940	0.5369	0.5224	0.5755	0.5522
**Peer Problems**	FORM	3	0.5927	0.5443	0.5212	0.6071	0.5696
**Peer Problems**	FORM	4	0.6103	0.5545	0.5519	0.5624	0.5571
**Peer Problems**	FORM	5	0.6109	0.5557	0.5354	0.6085	0.5776
**Peer Problems**	MFCC+MELS	Mean ± Std	0.6094 ± 0.0154	0.5589 ± 0.0159	0.5288 ± 0.0172	0.6367 ± 0.0226	0.5929 ± 0.0183
**Peer Problems**	MFCC+MELS	1	0.6195	0.5657	0.5318	0.6509	0.6043
**Peer Problems**	MFCC+MELS	2	0.5907	0.5315	0.5059	0.6014	0.5599
**Peer Problems**	MFCC+MELS	3	0.6163	0.5802	0.5542	0.6447	0.6088
**Peer Problems**	MFCC+MELS	4	0.5918	0.5609	0.5377	0.6212	0.5861
**Peer Problems**	MFCC+MELS	5	0.6288	0.5561	0.5142	0.6651	0.6056
**Peer Problems**	MFCC+PROS	Mean ± Std	0.6084 ± 0.0166	0.5614 ± 0.0165	0.5410 ± 0.0197	0.6140 ± 0.0248	0.5839 ± 0.0180
**Peer Problems**	MFCC+PROS	1	0.5844	0.5636	0.5576	0.5778	0.5697
**Peer Problems**	MFCC+PROS	2	0.6133	0.5582	0.5247	0.6439	0.5963
**Peer Problems**	MFCC+PROS	3	0.6193	0.5836	0.5637	0.6329	0.6051
**Peer Problems**	MFCC+PROS	4	0.5949	0.5332	0.5118	0.5929	0.5564
**Peer Problems**	MFCC+PROS	5	0.6303	0.5686	0.5472	0.6226	0.5918
**Peer Problems**	MFCC+FORM	Mean ± Std	0.6153 ± 0.0143	0.5734 ± 0.0116	0.5603 ± 0.0157	0.6060 ± 0.0213	0.5874 ± 0.0134
**Peer Problems**	MFCC+FORM	1	0.5899	0.5639	0.5553	0.5849	0.5728
**Peer Problems**	MFCC+FORM	2	0.6166	0.5682	0.5388	0.6415	0.6010
**Peer Problems**	MFCC+FORM	3	0.6124	0.5639	0.5566	0.5835	0.5714
**Peer Problems**	MFCC+FORM	4	0.6263	0.5950	0.5873	0.6141	0.6029
**Peer Problems**	MFCC+FORM	5	0.6312	0.5759	0.5637	0.6061	0.5887
**Peer Problems**	MELS+PROS	Mean ± Std	0.6192 ± 0.0168	0.5673 ± 0.0165	0.5405 ± 0.0234	0.6357 ± 0.0120	0.5973 ± 0.0103
**Peer Problems**	MELS+PROS	1	0.6196	0.5487	0.5106	0.6486	0.5929
**Peer Problems**	MELS+PROS	2	0.6037	0.5714	0.5506	0.6226	0.5939
**Peer Problems**	MELS+PROS	3	0.6339	0.5771	0.5472	0.6518	0.6105
**Peer Problems**	MELS+PROS	4	0.5975	0.5486	0.5189	0.6282	0.5820
**Peer Problems**	MELS+PROS	5	0.6412	0.5908	0.5755	0.6274	0.6070
**Peer Problems**	MELS+FORM	Mean ± Std	0.6199 ± 0.0065	0.5702 ± 0.0151	0.5476 ± 0.0260	0.6277 ± 0.0195	0.5953 ± 0.0082
**Peer Problems**	MELS+FORM	1	0.6216	0.5582	0.5247	0.6439	0.5963

**Peer Problems**	MELS+FORM	2	0.6243	0.5599	0.5388	0.6132	0.5827
**Peer Problems**	MELS+FORM	3	0.6175	0.5586	0.5283	0.6376	0.5926
**Peer Problems**	MELS+FORM	4	0.6087	0.5774	0.5495	0.6471	0.6084
**Peer Problems**	MELS+FORM	5	0.6276	0.5967	0.5967	0.5967	0.5967
**Peer Problems**	PROS+FORM	Mean ± Std	0.6044 ± 0.0160	0.5569 ± 0.0270	0.5401 ± 0.0353	0.6018 ± 0.0185	0.5752 ± 0.0204
**Peer Problems**	PROS+FORM	1	0.5885	0.5245	0.5035	0.5825	0.5473
**Peer Problems**	PROS+FORM	2	0.6012	0.5536	0.5224	0.6344	0.5889
**Peer Problems**	PROS+FORM	3	0.5880	0.5307	0.5094	0.5906	0.5538
**Peer Problems**	PROS+FORM	4	0.6147	0.5868	0.5778	0.6094	0.5961
**Peer Problems**	PROS+FORM	5	0.6298	0.5887	0.5873	0.5920	0.5900
**Peer Problems**	MFCC+MELS+PROS	Mean ± Std	0.6258 ± 0.0183	0.5724 ± 0.0101	0.5448 ± 0.0129	0.6414 ± 0.0133	0.6031 ± 0.0102
**Peer Problems**	MFCC+MELS+PROS	1	0.6224	0.5757	0.5459	0.6486	0.6089
**Peer Problems**	MFCC+MELS+PROS	2	0.6333	0.5791	0.5553	0.6368	0.6051
**Peer Problems**	MFCC+MELS+PROS	3	0.6408	0.5718	0.5354	0.6635	0.6135
**Peer Problems**	MFCC+MELS+PROS	4	0.5918	0.5533	0.5259	0.6259	0.5838
**Peer Problems**	MFCC+MELS+PROS	5	0.6407	0.5819	0.5613	0.6321	0.6041
**Peer Problems**	MFCC+MELS+FORM	Mean ± Std	0.6257 ± 0.0150	0.5624 ± 0.0170	0.5344 ± 0.0297	0.6352 ± 0.0248	0.5945 ± 0.0110
**Peer Problems**	MFCC+MELS+FORM	1	0.6170	0.5558	0.5271	0.6297	0.5879
**Peer Problems**	MFCC+MELS+FORM	2	0.6362	0.5619	0.5176	0.6745	0.6145
**Peer Problems**	MFCC+MELS+FORM	3	0.6416	0.5633	0.5354	0.6353	0.5942
**Peer Problems**	MFCC+MELS+FORM	4	0.6006	0.5392	0.5024	0.6400	0.5820
**Peer Problems**	MFCC+MELS+FORM	5	0.6330	0.5917	0.5896	0.5967	0.5938
**Peer Problems**	MFCC+PROS+FORM	Mean ± Std	0.6180 ± 0.0127	0.5716 ± 0.0090	0.5523 ± 0.0175	0.6202 ± 0.0276	0.5930 ± 0.0132
**Peer Problems**	MFCC+PROS+FORM	1	0.6049	0.5788	0.5788	0.5778	0.5788
**Peer Problems**	MFCC+PROS+FORM	2	0.6309	0.5704	0.5341	0.6604	0.6119
**Peer Problems**	MFCC+PROS+FORM	3	0.6065	0.5631	0.5472	0.6047	0.5800
**Peer Problems**	MFCC+PROS+FORM	4	0.6124	0.5612	0.5354	0.6282	0.5896
**Peer Problems**	MFCC+PROS+FORM	5	0.6353	0.5847	0.5660	0.6297	0.6045
**Peer Problems**	MELS+PROS+FORM	Mean ± Std	0.6150 ± 0.0146	0.5581 ± 0.0197	0.5269 ± 0.0242	0.6395 ± 0.0081	0.5935 ± 0.0145
**Peer Problems**	MELS+PROS+FORM	1	0.6034	0.5632	0.5294	0.6486	0.6016
**Peer Problems**	MELS+PROS+FORM	2	0.6096	0.5377	0.5035	0.6297	0.5768
**Peer Problems**	MELS+PROS+FORM	3	0.6260	0.5711	0.5401	0.6494	0.6058
**Peer Problems**	MELS+PROS+FORM	4	0.5985	0.5335	0.4976	0.6329	0.5749
**Peer Problems**	MELS+PROS+FORM	5	0.6376	0.5851	0.5637	0.6368	0.6081
**Peer Problems**	MFCC+MELS+PROS+	Mean ± Std	0.6187 ± 0.0158	0.5564 ± 0.0202	0.5231 ± 0.0337	0.6447 ± 0.0233	0.5955 ± 0.0096
**Peer Problems**	MFCC+MELS+PROS+	1	0.6084	0.5586	0.5271	0.6392	0.5942
**Peer Problems**	MFCC+MELS+PROS+	2	0.6257	0.5459	0.5035	0.6580	0.5961
**Peer Problems**	MFCC+MELS+PROS+	3	0.6344	0.5583	0.5142	0.6729	0.6106
**Peer Problems**	MFCC+MELS+PROS+	4	0.5929	0.5289	0.4858	0.6494	0.5803
**Peer Problems**	MFCC+MELS+PROS+	5	0.6320	0.5905	0.5849	0.6038	0.5962
LR_Female_detailed_performance_results							
**Symptom**	Subspace	Fold	AUC	F1	Sensitivity	Specificity	Precision
**Emotional Symptoms**	MFCC	1	0.6042467321928	0.5905383360522	0.6094276094276	0.5454545454545	0.5727848101265
**Emotional Symptoms**	MFCC	2	0.5796460678615	0.5442176870748	0.5387205387205	0.5589225589225	0.5498281786941
**Emotional Symptoms**	MFCC	3	0.5936922536249	0.5876460767946	0.5925925925925	0.5757575757575	0.5827814569536
**Emotional Symptoms**	MFCC	4	0.6056738556738	0.5647058823529	0.5675675675675	0.5589225589225	0.5618729096989
**Emotional Symptoms**	MFCC	5	0.6080398580398	0.6042003231017	0.6296296296296	0.5439189189189	0.5807453416149
**Emotional Symptoms**	MFCC	Mean	0.5983 ± 0.0105	0.5783 ± 0.0212	0.5876 ± 0.0318	0.5566 ± 0.0115	0.5696 ± 0.0123
**Emotional Symptoms**	MELS	1	0.5572900724415	0.5664556962025	0.6026936026936	0.4747474747474	0.5343283582089
**Emotional Symptoms**	MELS	2	0.5865274518473	0.5676998368678	0.5858585858585	0.5218855218855	0.5506329113924
**Emotional Symptoms**	MELS	3	0.5270097155619	0.5424836601307	0.5589225589225	0.4983164983164	0.5269841269841
**Emotional Symptoms**	MELS	4	0.5674196924196	0.5533333333333	0.5608108108108	0.5353535353535	0.5460526315789
**Emotional Symptoms**	MELS	5	0.5531440531440	0.5589660743134	0.5824915824915	0.4966216216216	0.5372670807453
**Emotional Symptoms**	MELS	Mean	0.5583 ± 0.0194	0.5578 ± 0.0093	0.5782 ± 0.0164	0.5054 ± 0.0211	0.5391 ± 0.0084
**Emotional Symptoms**	PROS	1	0.5700892199208	0.5605306799336	0.5690235690235	0.5387205387205	0.5522875816993
**Emotional Symptoms**	PROS	2	0.5761997075128	0.5792	0.6094276094276	0.5050505050505	0.5518292682926
**Emotional Symptoms**	PROS	3	0.5179630196465	0.5641838351822	0.5993265993265	0.4747474747474	0.5329341317365

**Emotional Symptoms**	PROS	4	0.5463986713986	0.5686591276252	0.5945945945945	0.5050505050505	0.5448916408668
**Emotional Symptoms**	PROS	5	0.5180976430976	0.5249169435215	0.5319865319865	0.5033783783783	0.5180327868852
**Emotional Symptoms**	PROS	Mean	0.5457 ± 0.0247	0.5595 ± 0.0184	0.5809 ± 0.0278	0.5054 ± 0.0203	0.5400 ± 0.0130
**Emotional Symptoms**	FORM	1	0.5177702955480	0.5290322580645	0.5521885521885	0.4646464646464	0.5077399380804
**Emotional Symptoms**	FORM	2	0.5801902300218	0.5704809286898	0.5791245791245	0.5488215488215	0.5620915032679
**Emotional Symptoms**	FORM	3	0.5383917740820	0.5882352941176	0.6397306397306	0.4646464646464	0.5444126074498
**Emotional Symptoms**	FORM	4	0.5413367913367	0.5923664122137	0.6554054054054	0.4444444444444	0.5403899721448
**Emotional Symptoms**	FORM	5	0.5327486577486	0.5578446909667	0.5925925925925	0.4662162162162	0.5269461077844
**Emotional Symptoms**	FORM	Mean	0.5421 ± 0.0207	0.5676 ± 0.0229	0.6038 ± 0.0383	0.4778 ± 0.0364	0.5363 ± 0.0182
**Emotional Symptoms**	MFCC+MELS	1	0.5868562164858	0.5709624796084	0.5892255892255	0.5252525252525	0.5537974683544
**Emotional Symptoms**	MFCC+MELS	2	0.5929667040778	0.5597269624573	0.5521885521885	0.5791245791245	0.5674740484429
**Emotional Symptoms**	MFCC+MELS	3	0.5889875182804	0.5649350649350	0.5858585858585	0.5117845117845	0.5454545454545
**Emotional Symptoms**	MFCC+MELS	4	0.6088133588133	0.5771812080536	0.5810810810810	0.5690235690235	0.5733333333333
**Emotional Symptoms**	MFCC+MELS	5	0.5964828464828	0.5958132045088	0.6228956228956	0.5304054054054	0.5709876543209
**Emotional Symptoms**	MFCC+MELS	Mean	0.5948 ± 0.0077	0.5737 ± 0.0125	0.5862 ± 0.0225	0.5431 ± 0.0262	0.5622 ± 0.0108
**Emotional Symptoms**	MFCC+PROS	1	0.6014125542745	0.5899513776337	0.6127946127946	0.5353535353535	0.56875
**Emotional Symptoms**	MFCC+PROS	2	0.5876837964380	0.5622895622895	0.5622895622895	0.5622895622895	0.5622895622895
**Emotional Symptoms**	MFCC+PROS	3	0.5899851489077	0.5411764705882	0.5420875420875	0.5387205387205	0.5402684563758
**Emotional Symptoms**	MFCC+PROS	4	0.6099281099281	0.5866666666666	0.5945945945945	0.5690235690235	0.5789473684210
**Emotional Symptoms**	MFCC+PROS	5	0.5909204659204	0.5747508305647	0.5824915824915	0.5540540540540	0.5672131147540
**Emotional Symptoms**	MFCC+PROS	Mean	0.5960 ± 0.0084	0.5710 ± 0.0178	0.5789 ± 0.0247	0.5519 ± 0.0131	0.5635 ± 0.0128
**Emotional Symptoms**	MFCC+FORM	1	0.5913228808851	0.5958132045088	0.6228956228956	0.5319865319865	0.5709876543209
**Emotional Symptoms**	MFCC+FORM	2	0.5715970025734	0.5511265164644	0.5353535353535	0.5925925925925	0.5678571428571
**Emotional Symptoms**	MFCC+FORM	3	0.5951546894307	0.5719063545150	0.5757575757575	0.5622895622895	0.5681063122923
**Emotional Symptoms**	MFCC+FORM	4	0.6118163618163	0.5834683954619	0.6081081081081	0.5252525252525	0.5607476635514
**Emotional Symptoms**	MFCC+FORM	5	0.6053439803439	0.6032786885245	0.6195286195286	0.5641891891891	0.5878594249201
**Emotional Symptoms**	MFCC+FORM	Mean	0.5950 ± 0.0138	0.5811 ± 0.0184	0.5923 ± 0.0330	0.5553 ± 0.0244	0.5711 ± 0.0090
**Emotional Symptoms**	MELS+PROS	1	0.5491729868834	0.5555555555555	0.5723905723905	0.5117845117845	0.5396825396825
**Emotional Symptoms**	MELS+PROS	2	0.5672890521375	0.5366666666666	0.5420875420875	0.5218855218855	0.5313531353135
**Emotional Symptoms**	MELS+PROS	3	0.5488442222448	0.5768621236133	0.6127946127946	0.4882154882154	0.5449101796407
**Emotional Symptoms**	MELS+PROS	4	0.5855969605969	0.5955414012738	0.6317567567567	0.5117845117845	0.5632530120481
**Emotional Symptoms**	MELS+PROS	5	0.5195536445536	0.5339805825242	0.5555555555555	0.4729729729729	0.5140186915887
**Emotional Symptoms**	MELS+PROS	Mean	0.5541 ± 0.0220	0.5597 ± 0.0236	0.5829 ± 0.0341	0.5013 ± 0.0180	0.5386 ± 0.0162
**Emotional Symptoms**	MELS+FORM	1	0.5565871963178	0.5737179487179	0.6026936026936	0.5016835016835	0.5474006116207
**Emotional Symptoms**	MELS+FORM	2	0.5789091816027	0.5575959933222	0.5622895622895	0.5454545454545	0.5529801324503
**Emotional Symptoms**	MELS+FORM	3	0.5446156287907	0.5714285714285	0.6060606060606	0.4848484848484	0.5405405405405
**Emotional Symptoms**	MELS+FORM	4	0.5618004368004	0.5436573311367	0.5574324324324	0.5084175084175	0.5305466237942
**Emotional Symptoms**	MELS+FORM	5	0.5680225680225	0.5718954248366	0.5892255892255	0.5270270270270	0.5555555555555
**Emotional Symptoms**	MELS+FORM	Mean	0.5620 ± 0.0114	0.5637 ± 0.0115	0.5835 ± 0.0202	0.5135 ± 0.0209	0.5454 ± 0.0090
**Emotional Symptoms**	PROS+FORM	1	0.5344692718430	0.5425361155698	0.5690235690235	0.4713804713804	0.5184049079754
**Emotional Symptoms**	PROS+FORM	2	0.5830697547869	0.6019417475728	0.6262626262626	0.5454545454545	0.5794392523364
**Emotional Symptoms**	PROS+FORM	3	0.5250484644424	0.5669291338582	0.6060606060606	0.4680134680134	0.5325443786982
**Emotional Symptoms**	PROS+FORM	4	0.5585926835926	0.578125	0.625	0.4646464646464	0.5377906976744
**Emotional Symptoms**	PROS+FORM	5	0.5258554008554	0.5433715220949	0.5589225589225	0.5	0.5286624203821
**Emotional Symptoms**	PROS+FORM	Mean	0.5454 ± 0.0224	0.5666 ± 0.0224	0.5971 ± 0.0281	0.4899 ± 0.0305	0.5394 ± 0.0210
**Emotional Symptoms**	MFCC+MELS+PROS	1	0.6016732986429	0.5705024311183	0.5925925925925	0.5151515151515	0.55
**Emotional Symptoms**	MFCC+MELS+PROS	2	0.5991792220748	0.5852842809364	0.5892255892255	0.5757575757575	0.5813953488372
**Emotional Symptoms**	MFCC+MELS+PROS	3	0.5975807457288	0.5918699186991	0.6127946127946	0.5420875420875	0.5723270440251
**Emotional Symptoms**	MFCC+MELS+PROS	4	0.6245904995905	0.5996705107084	0.6148648648648	0.5656565656565	0.5852090032154
**Emotional Symptoms**	MFCC+MELS+PROS	5	0.6004186004186	0.5832012678288	0.6195286195286	0.4932432432432	0.5508982035928
**Emotional Symptoms**	MFCC+MELS+PROS	Mean	0.6047 ± 0.0100	0.5861 ± 0.0097	0.6058 ± 0.0124	0.5384 ± 0.0308	0.5680 ± 0.0149
**Emotional Symptoms**	MFCC+MELS+FORM	1	0.5895430171524	0.5829307568438	0.6094276094276	0.5185185185185	0.5586419753086
**Emotional Symptoms**	MFCC+MELS+FORM	2	0.5848949653663	0.5753424657534	0.5656565656565	0.5993265993265	0.5853658536585
**Emotional Symptoms**	MFCC+MELS+FORM	3	0.5797027514199	0.5566666666666	0.5622895622895	0.5420875420875	0.5511551155115
**Emotional Symptoms**	MFCC+MELS+FORM	4	0.6232709982709	0.5936981757877	0.6047297297297	0.5690235690235	0.5830618892508
**Emotional Symptoms**	MFCC+MELS+FORM	5	0.5938893438893	0.5652892561983	0.5757575757575	0.5371621621621	0.5551948051948
**Emotional Symptoms**	MFCC+MELS+FORM	Mean	0.5943 ± 0.0153	0.5748 ± 0.0130	0.5836 ± 0.0198	0.5532 ± 0.0281	0.5667 ± 0.0145

**Emotional Symptoms**	MFCC+PROS+FORM	1	0.5877858268430	0.5929487179487	0.6228956228956	0.5218855218855	0.5657492354740
**Emotional Symptoms**	MFCC+PROS+FORM	2	0.5795213640331	0.5423143350604	0.5286195286195	0.5791245791245	0.5567375886524
**Emotional Symptoms**	MFCC+PROS+FORM	3	0.5866068088290	0.5548172757475	0.5622895622895	0.5353535353535	0.5475409836065
**Emotional Symptoms**	MFCC+PROS+FORM	4	0.6088588588588	0.5859247135842	0.6047297297297	0.5420875420875	0.5682539682539
**Emotional Symptoms**	MFCC+PROS+FORM	5	0.6061516061516	0.5847176079734	0.5925925925925	0.5641891891891	0.5770491803278
**Emotional Symptoms**	MFCC+PROS+FORM	Mean	0.5938 ± 0.0116	0.5721 ± 0.0199	0.5822 ± 0.0333	0.5485 ± 0.0205	0.5631 ± 0.0101
**Emotional Symptoms**	MELS+PROS+FORM	1	0.5601129136482	0.5645161290322	0.5892255892255	0.5016835016835	0.5417956656346
**Emotional Symptoms**	MELS+PROS+FORM	2	0.6049836184510	0.5936981757877	0.6026936026936	0.5723905723905	0.5849673202614
**Emotional Symptoms**	MELS+PROS+FORM	3	0.5426543776712	0.5425361155698	0.5690235690235	0.4713804713804	0.5184049079754
**Emotional Symptoms**	MELS+PROS+FORM	4	0.5721630721630	0.5643564356435	0.5777027027027	0.5319865319865	0.5516129032258
**Emotional Symptoms**	MELS+PROS+FORM	5	0.5655541905541	0.5792	0.6094276094276	0.5033783783783	0.5518292682926
**Emotional Symptoms**	MELS+PROS+FORM	Mean	0.5691 ± 0.0204	0.5689 ± 0.0171	0.5896 ± 0.0150	0.5162 ± 0.0340	0.5497 ± 0.0214
**Emotional Symptoms**	MFCC+MELS+PROS+	1	0.5933634889863	0.5922330097087	0.6161616161616	0.5353535353535	0.5700934579439
**Emotional Symptoms**	MFCC+MELS+PROS+	2	0.5947465678105	0.5753899480069	0.5589225589225	0.6161616161616	0.5928571428571
**Emotional Symptoms**	MFCC+MELS+PROS+	3	0.5862667074788	0.5667215815486	0.5791245791245	0.5353535353535	0.5548387096774
**Emotional Symptoms**	MFCC+MELS+PROS+	4	0.6293338793338	0.6010016694490	0.6081081081081	0.5858585858585	0.5940594059405
**Emotional Symptoms**	MFCC+MELS+PROS+	5	0.6023978523978	0.5831960461285	0.5959595959595	0.5506756756756	0.5709677419354
**Emotional Symptoms**	MFCC+MELS+PROS+	Mean	0.6012 ± 0.0150	0.5837 ± 0.0121	0.5917 ± 0.0206	0.5647 ± 0.0317	0.5766 ± 0.0149
**Hyperactivity**	MFCC	1	0.6505872278806	0.6362484157160	0.6622691292875	0.5804749340369	0.6121951219512
**Hyperactivity**	MFCC	2	0.6273487374774	0.6185044359949	0.6437994722955	0.5620052770448	0.5951219512195
**Hyperactivity**	MFCC	3	0.6784274684804	0.6529562982005	0.6701846965699	0.6174142480211	0.6365914786967
**Hyperactivity**	MFCC	4	0.6174282084572	0.6102564102564	0.6296296296296	0.5672823218997	0.5920398009950
**Hyperactivity**	MFCC	5	0.6450908126369	0.6272493573264	0.6437994722955	0.5899470899470	0.6115288220551
**Hyperactivity**	MFCC	Mean	0.6438 ± 0.0210	0.6290 ± 0.0148	0.6499 ± 0.0145	0.5834 ± 0.0196	0.6095 ± 0.0159
**Hyperactivity**	MELS	1	0.6458949742761	0.6138107416879	0.6332453825857	0.5699208443271	0.5955334987593
**Hyperactivity**	MELS	2	0.6303075027325	0.5997392438070	0.6068601583113	0.5831134564643	0.5927835051546
**Hyperactivity**	MELS	3	0.6383762296280	0.6290115532734	0.6464379947229	0.5910290237467	0.6125
**Hyperactivity**	MELS	4	0.6477502757186	0.6559405940594	0.7010582010582	0.5646437994722	0.6162790697674
**Hyperactivity**	MELS	5	0.6294481439600	0.6223862238622	0.6675461741424	0.5211640211640	0.5829493087557
**Hyperactivity**	MELS	Mean	0.6384 ± 0.0076	0.6242 ± 0.0187	0.6510 ± 0.0318	0.5660 ± 0.0243	0.6000 ± 0.0125
**Hyperactivity**	PROS	1	0.6021957519092	0.5885286783042	0.6226912928759	0.5065963060686	0.5579196217494
**Hyperactivity**	PROS	2	0.6186464867273	0.6254728877679	0.6543535620052	0.5620052770448	0.5990338164251
**Hyperactivity**	PROS	3	0.5987705460140	0.6140567200986	0.6569920844327	0.5171503957783	0.5763888888888
**Hyperactivity**	PROS	4	0.5787508201756	0.6029411764705	0.6507936507936	0.4934036939313	0.5616438356164
**Hyperactivity**	PROS	5	0.6125699766860	0.6300366300366	0.6807387862796	0.5185185185185	0.5863636363636
**Hyperactivity**	PROS	Mean	0.6022 ± 0.0137	0.6122 ± 0.0151	0.6531 ± 0.0185	0.5195 ± 0.0231	0.5763 ± 0.0153
**Hyperactivity**	FORM	1	0.6034767232196	0.5987421383647	0.6279683377308	0.5303430079155	0.5721153846153
**Hyperactivity**	FORM	2	0.6385293892412	0.6354679802955	0.6807387862796	0.5382585751978	0.5958429561200
**Hyperactivity**	FORM	3	0.6295417046664	0.6221662468513	0.6517150395778	0.5567282321899	0.5951807228915
**Hyperactivity**	FORM	4	0.6488042886459	0.625	0.6746031746031	0.5171503957783	0.5821917808219
**Hyperactivity**	FORM	5	0.6285546760480	0.6331288343558	0.6807387862796	0.5291005291005	0.5917431192660
**Hyperactivity**	FORM	Mean	0.6298 ± 0.0150	0.6229 ± 0.0130	0.6632 ± 0.0206	0.5343 ± 0.0131	0.5874 ± 0.0091
**Hyperactivity**	MFCC+MELS	1	0.6908821297540	0.6352941176470	0.6411609498680	0.6226912928759	0.6295336787564
**Hyperactivity**	MFCC+MELS	2	0.6524947612450	0.6551724137931	0.7018469656992	0.5593667546174	0.6143187066974
**Hyperactivity**	MFCC+MELS	3	0.7008235810109	0.6692708333333	0.6781002638522	0.6517150395778	0.6606683804627
**Hyperactivity**	MFCC+MELS	4	0.6415797629517	0.6342710997442	0.6560846560846	0.5883905013192	0.6138613861386
**Hyperactivity**	MFCC+MELS	5	0.6620667029638	0.6448362720403	0.6754617414248	0.5793650793650	0.6168674698795
**Hyperactivity**	MFCC+MELS	Mean	0.6696 ± 0.0226	0.6478 ± 0.0131	0.6705 ± 0.0207	0.6003 ± 0.0329	0.6270 ± 0.0178
**Hyperactivity**	MFCC+PROS	1	0.6910840219714	0.6449086161879	0.6517150395778	0.6306068601583	0.6382428940568
**Hyperactivity**	MFCC+PROS	2	0.6463126823121	0.6318471337579	0.6543535620052	0.5831134564643	0.6108374384236
**Hyperactivity**	MFCC+PROS	3	0.6845956238121	0.6650185414091	0.7097625329815	0.5751978891820	0.6255813953488
**Hyperactivity**	MFCC+PROS	4	0.6586673367676	0.6398996235884	0.6746031746031	0.5672823218997	0.6085918854415
**Hyperactivity**	MFCC+PROS	5	0.6751476316120	0.6615186615186	0.6781002638522	0.6269841269841	0.6457286432160
**Hyperactivity**	MFCC+PROS	Mean	0.6712 ± 0.0165	0.6486 ± 0.0127	0.6737 ± 0.0209	0.5966 ± 0.0268	0.6258 ± 0.0146
**Hyperactivity**	MFCC+FORM	1	0.6539288921686	0.6267880364109	0.6358839050131	0.6068601583113	0.6179487179487
**Hyperactivity**	MFCC+FORM	2	0.6724751289673	0.6575	0.6939313984168	0.5831134564643	0.6247030878859
**Hyperactivity**	MFCC+FORM	3	0.7132434332815	0.6846153846153	0.7044854881266	0.6464379947229	0.6658354114713

**Hyperactivity**	MFCC+FORM	4	0.679056553726	0.656097560975	0.711640211640	0.543535620052	0.608597285067
**Hyperactivity**	MFCC+FORM	5	0.665877902025	0.637419354838	0.651715039577	0.605820105820	0.623737373737
**Hyperactivity**	MFCC+FORM	Mean	0.6769 ± 0.0200	0.6525 ± 0.0198	0.6795 ± 0.0301	0.5972 ± 0.0337	0.6282 ± 0.0197
**Hyperactivity**	MELS+PROS	1	0.630794828774	0.587615283267	0.588390501319	0.585751978891	0.586842105263
**Hyperactivity**	MELS+PROS	2	0.654478874416	0.627700127064	0.651715039577	0.575197889182	0.605392156862
**Hyperactivity**	MELS+PROS	3	0.657138282245	0.638461538461	0.656992084432	0.598944591029	0.620947630922
**Hyperactivity**	MELS+PROS	4	0.647345423071	0.636363636363	0.685185185185	0.532981530343	0.594036697247
**Hyperactivity**	MELS+PROS	5	0.639736985383	0.629072681704	0.662269129287	0.555555555555	0.599045346062
**Hyperactivity**	MELS+PROS	Mean	0.6459 ± 0.0097	0.6238 ± 0.0186	0.6489 ± 0.0323	0.5697 ± 0.0232	0.6013 ± 0.0116
**Hyperactivity**	MELS+FORM	1	0.692706121511	0.659898477157	0.686015831134	0.606860158311	0.635696821515
**Hyperactivity**	MELS+FORM	2	0.664114006446	0.629677419354	0.643799472295	0.598944591029	0.616161616161
**Hyperactivity**	MELS+FORM	3	0.676749674535	0.640726329442	0.651715039577	0.617414248021	0.630102040816
**Hyperactivity**	MELS+FORM	4	0.673004704666	0.654275092936	0.698412698412	0.564643799472	0.615384615384
**Hyperactivity**	MELS+FORM	5	0.670652371180	0.659147869674	0.693931398416	0.587301587301	0.627684964200
**Hyperactivity**	MELS+FORM	Mean	0.6754 ± 0.0096	0.6487 ± 0.0118	0.6748 ± 0.0226	0.5950 ± 0.0181	0.6250 ± 0.0080
**Hyperactivity**	PROS+FORM	1	0.629757520485	0.630517023959	0.659630606860	0.567282321899	0.603864734299
**Hyperactivity**	PROS+FORM	2	0.671827681511	0.635204081632	0.656992084432	0.588390501319	0.614814814814
**Hyperactivity**	PROS+FORM	3	0.647461379411	0.622617534942	0.646437994722	0.569920844327	0.600490196078
**Hyperactivity**	PROS+FORM	4	0.649928103753	0.632352941176	0.682539682539	0.525065963060	0.589041095890
**Hyperactivity**	PROS+FORM	5	0.640323323700	0.626716604244	0.662269129287	0.547619047619	0.594786729857
**Hyperactivity**	PROS+FORM	Mean	0.6479 ± 0.0139	0.6295 ± 0.0044	0.6616 ± 0.0118	0.5597 ± 0.0216	0.6006 ± 0.0087
**Hyperactivity**	MFCC+MELS+PROS	1	0.705752535835	0.657105606258	0.664907651715	0.641160949868	0.649484536082
**Hyperactivity**	MFCC+MELS+PROS	2	0.662074198870	0.676470588235	0.728232189973	0.575197889182	0.631578947368
**Hyperactivity**	MFCC+MELS+PROS	3	0.679576165579	0.653944020356	0.678100263852	0.604221635883	0.631449631449
**Hyperactivity**	MFCC+MELS+PROS	4	0.662813586296	0.637279596977	0.669312169312	0.569920844327	0.608173076923
**Hyperactivity**	MFCC+MELS+PROS	5	0.684780332537	0.647571606475	0.686015831134	0.566137566137	0.613207547169
**Hyperactivity**	MFCC+MELS+PROS	Mean	0.6790 ± 0.0161	0.6545 ± 0.0129	0.6853 ± 0.0227	0.5913 ± 0.0283	0.6268 ± 0.0148
**Hyperactivity**	MFCC+MELS+FORM	1	0.702557069360	0.658974358974	0.678100263852	0.620052770448	0.640897755610
**Hyperactivity**	MFCC+MELS+FORM	2	0.673707367673	0.633766233766	0.643799472295	0.612137203166	0.624040920716
**Hyperactivity**	MFCC+MELS+FORM	3	0.718102770100	0.679487179487	0.699208443271	0.641160949868	0.660847880299
**Hyperactivity**	MFCC+MELS+FORM	4	0.699222403707	0.667503136762	0.703703703703	0.596306068601	0.634844868735
**Hyperactivity**	MFCC+MELS+FORM	5	0.694796945456	0.669987546699	0.709762532981	0.589947089947	0.634433962264
**Hyperactivity**	MFCC+MELS+FORM	Mean	0.6977 ± 0.0143	0.6619 ± 0.0155	0.6869 ± 0.0240	0.6119 ± 0.0182	0.6390 ± 0.0122
**Hyperactivity**	MFCC+PROS+FORM	1	0.670379626986	0.648578811369	0.662269129287	0.620052770448	0.635443037974
**Hyperactivity**	MFCC+PROS+FORM	2	0.683029218677	0.671717171717	0.701846965699	0.612137203166	0.644067796610
**Hyperactivity**	MFCC+PROS+FORM	3	0.718701484952	0.688442211055	0.722955145118	0.622691292875	0.657074340527
**Hyperactivity**	MFCC+PROS+FORM	4	0.689687425835	0.681927710843	0.748677248677	0.554089709762	0.626106194690
**Hyperactivity**	MFCC+PROS+FORM	5	0.681541511356	0.643320363164	0.654353562005	0.619047619047	0.632653061224
**Hyperactivity**	MFCC+PROS+FORM	Mean	0.6887 ± 0.0162	0.6668 ± 0.0179	0.6980 ± 0.0357	0.6056 ± 0.0260	0.6391 ± 0.0107
**Hyperactivity**	MELS+PROS+FORM	1	0.695546536156	0.680203045685	0.707124010554	0.627968337730	0.655256723716
**Hyperactivity**	MELS+PROS+FORM	2	0.668743603845	0.645326504481	0.664907651715	0.604221635883	0.626865671641
**Hyperactivity**	MELS+PROS+FORM	3	0.684324113588	0.638020833333	0.646437994722	0.620052770448	0.629820051413
**Hyperactivity**	MELS+PROS+FORM	4	0.682169730982	0.661710037174	0.706349206349	0.572559366754	0.622377622377
**Hyperactivity**	MELS+PROS+FORM	5	0.684256816182	0.664179104477	0.704485488126	0.582010582010	0.628235294117
**Hyperactivity**	MELS+PROS+FORM	Mean	0.6830 ± 0.0085	0.6579 ± 0.0149	0.6859 ± 0.0253	0.6014 ± 0.0213	0.6325 ± 0.0116
**Hyperactivity**	MFCC+MELS+PROS+	1	0.715770566899	0.677083333333	0.686015831134	0.659630606860	0.668380462724
**Hyperactivity**	MFCC+MELS+PROS+	2	0.683231110894	0.653061224489	0.675461741424	0.606860158311	0.632098765432
**Hyperactivity**	MFCC+MELS+PROS+	3	0.721138115162	0.686868686868	0.717678100263	0.627968337730	0.658595641646
**Hyperactivity**	MFCC+MELS+PROS+	4	0.711577389677	0.689740420271	0.738095238095	0.598944591029	0.647331786542
**Hyperactivity**	MFCC+MELS+PROS+	5	0.702377462271	0.673366834170	0.707124010554	0.605820105820	0.642685851318
**Hyperactivity**	MFCC+MELS+PROS+	Mean	0.7068 ± 0.0133	0.6760 ± 0.0130	0.7049 ± 0.0223	0.6198 ± 0.0221	0.6498 ± 0.0126
**Conduct Problems**	MFCC	1	0.510380690482	0.522522522522	0.523465703971	0.519855595667	0.521582733812
**Conduct Problems**	MFCC	2	0.550808690325	0.544802867383	0.548736462093	0.534296028880	0.540925266903
**Conduct Problems**	MFCC	3	0.527909916719	0.560934891485	0.606498194945	0.444043321299	0.521739130434
**Conduct Problems**	MFCC	4	0.602600324386	0.592057761732	0.592057761732	0.590579710144	0.592057761732
**Conduct Problems**	MFCC	5	0.574451943703	0.584775086505	0.612318840579	0.519855595667	0.559602649006
**Conduct Problems**	MFCC	Mean	0.5532 ± 0.0328	0.5610 ± 0.0256	0.5766 ± 0.0347	0.5217 ± 0.0468	0.5472 ± 0.0265

**Conduct Problems**	MELS	1	0.5221493828930	0.5331010452961	0.5523465703971	0.4801444043321	0.5151515151515
**Conduct Problems**	MELS	2	0.5021830077284	0.5035460992907	0.5126353790613	0.4765342960288	0.4947735191637
**Conduct Problems**	MELS	3	0.5254727677931	0.5719008264462	0.6245487364620	0.4404332129963	0.5274390243902
**Conduct Problems**	MELS	4	0.5150682781353	0.5255474452554	0.5198555956678	0.5398550724637	0.5313653136531
**Conduct Problems**	MELS	5	0.5417516873332	0.5435168738898	0.5543478260869	0.5162454873646	0.5331010452961
**Conduct Problems**	MELS	Mean	0.5213 ± 0.0130	0.5355 ± 0.0224	0.5527 ± 0.0396	0.4906 ± 0.0344	0.5204 ± 0.0143
**Conduct Problems**	PROS	1	0.5415292783693	0.5525423728813	0.5884476534296	0.4584837545126	0.5207667731629
**Conduct Problems**	PROS	2	0.5275840946708	0.5130890052356	0.5306859205776	0.4620938628158	0.4966216216216
**Conduct Problems**	PROS	3	0.5118273403797	0.53125	0.5523465703971	0.4729241877256	0.5117056856187
**Conduct Problems**	PROS	4	0.5743865431904	0.5525846702317	0.5595667870036	0.5326086956521	0.5457746478873
**Conduct Problems**	PROS	5	0.5248260346361	0.5212765957446	0.5326086956521	0.4909747292418	0.5104166666666
**Conduct Problems**	PROS	Mean	0.5360 ± 0.0214	0.5341 ± 0.0161	0.5527 ± 0.0210	0.4834 ± 0.0271	0.5171 ± 0.0163
**Conduct Problems**	FORM	1	0.5447353673317	0.5457627118644	0.5812274368231	0.4512635379061	0.5143769968051
**Conduct Problems**	FORM	2	0.5690025935435	0.58	0.6281588447653	0.4620938628158	0.5386996904024
**Conduct Problems**	FORM	3	0.5452697154921	0.5808580858085	0.6353790613718	0.4476534296028	0.5349544072948
**Conduct Problems**	FORM	4	0.5884214932245	0.5949579831932	0.6389891696750	0.4891304347826	0.5566037735849
**Conduct Problems**	FORM	5	0.5979699680845	0.6182432432432	0.6630434782608	0.5198555956678	0.5791139240506
**Conduct Problems**	FORM	Mean	0.5691 ± 0.0218	0.5840 ± 0.0236	0.6294 ± 0.0268	0.4740 ± 0.0271	0.5447 ± 0.0218
**Conduct Problems**	MFCC+MELS	1	0.5284181991163	0.5285714285714	0.5342960288808	0.5126353790613	0.5229681978798
**Conduct Problems**	MFCC+MELS	2	0.5336052861369	0.5203539823008	0.5306859205776	0.4909747292418	0.5104166666666
**Conduct Problems**	MFCC+MELS	3	0.5201553519529	0.5536912751677	0.5956678700361	0.4440433212996	0.5172413793103
**Conduct Problems**	MFCC+MELS	4	0.5806649924135	0.5714285714285	0.5776173285198	0.5543478260869	0.5653710247349
**Conduct Problems**	MFCC+MELS	5	0.5486710615811	0.5574912891986	0.5797101449275	0.5018050541516	0.5369127516778
**Conduct Problems**	MFCC+MELS	Mean	0.5423 ± 0.0213	0.5463 ± 0.0190	0.5636 ± 0.0262	0.5008 ± 0.0356	0.5306 ± 0.0195
**Conduct Problems**	MFCC+PROS	1	0.5466772667439	0.5531914893617	0.5631768953068	0.5270758122743	0.5435540069686
**Conduct Problems**	MFCC+PROS	2	0.5642846902735	0.5402504472271	0.5451263537906	0.5270758122743	0.5354609929078
**Conduct Problems**	MFCC+PROS	3	0.5569471777294	0.5767284991568	0.6173285198555	0.4765342960288	0.5411392405063
**Conduct Problems**	MFCC+PROS	4	0.6279103228169	0.5996409335727	0.6028880866425	0.5905797101449	0.5964285714285
**Conduct Problems**	MFCC+PROS	5	0.5961518338303	0.5955249569707	0.6268115942028	0.5234657039711	0.5672131147540
**Conduct Problems**	MFCC+PROS	Mean	0.5784 ± 0.0298	0.5731 ± 0.0232	0.5911 ± 0.0316	0.5289 ± 0.0363	0.5568 ± 0.0226
**Conduct Problems**	MFCC+FORM	1	0.5405127135763	0.5483304042179	0.5631768953068	0.5090252707581	0.5342465753424
**Conduct Problems**	MFCC+FORM	2	0.5707620326082	0.5496453900709	0.5595667870036	0.5234657039711	0.5400696864111
**Conduct Problems**	MFCC+FORM	3	0.5600099049902	0.5699658703071	0.6028880866425	0.4873646209386	0.5404530744336
**Conduct Problems**	MFCC+FORM	4	0.6219196358499	0.5971731448763	0.6101083032490	0.5652173913043	0.5847750865051
**Conduct Problems**	MFCC+FORM	5	0.6288520902003	0.6078098471986	0.6485507246376	0.5162454873646	0.5718849840255
**Conduct Problems**	MFCC+FORM	Mean	0.5844 ± 0.0349	0.5746 ± 0.0243	0.5969 ± 0.0329	0.5203 ± 0.0255	0.5543 ± 0.0202
**Conduct Problems**	MELS+PROS	1	0.5549661796713	0.5540069686411	0.5740072202166	0.5018050541516	0.5353535353535
**Conduct Problems**	MELS+PROS	2	0.5344654563463	0.4963503649635	0.4909747292418	0.5126353790613	0.5018450184501
**Conduct Problems**	MELS+PROS	3	0.5344263577004	0.5266781411359	0.5523465703971	0.4548736462093	0.5032894736842
**Conduct Problems**	MELS+PROS	4	0.5623920891539	0.5101663585951	0.4981949458483	0.5434782608695	0.5227272727272
**Conduct Problems**	MELS+PROS	5	0.5600245905927	0.5553539019963	0.5543478260869	0.5595667870036	0.5563636363636
**Conduct Problems**	MELS+PROS	Mean	0.5493 ± 0.0123	0.5285 ± 0.0234	0.5340 ± 0.0331	0.5145 ± 0.0363	0.5239 ± 0.0205
**Conduct Problems**	MELS+FORM	1	0.5527375568559	0.5415929203539	0.5523465703971	0.5126353790613	0.53125
**Conduct Problems**	MELS+FORM	2	0.5398741023602	0.5225225225225	0.5234657039711	0.5198555956678	0.5215827338129
**Conduct Problems**	MELS+FORM	3	0.5406300095140	0.5323992994746	0.5487364620938	0.4873646209386	0.5170068027210
**Conduct Problems**	MELS+FORM	4	0.5908805525035	0.5856353591160	0.5740072202166	0.6123188405797	0.5977443609022
**Conduct Problems**	MELS+FORM	5	0.5848637053314	0.5849387040280	0.6050724637681	0.5379061371841	0.5661016949152
**Conduct Problems**	MELS+FORM	Mean	0.5618 ± 0.0219	0.5534 ± 0.0267	0.5607 ± 0.0274	0.5340 ± 0.0424	0.5467 ± 0.0308
**Conduct Problems**	PROS+FORM	1	0.5546273247403	0.5680272108843	0.6028880866425	0.4801444043321	0.5369774919614
**Conduct Problems**	PROS+FORM	2	0.5752323111209	0.5876288659793	0.6173285198555	0.5162454873646	0.5606557377049
**Conduct Problems**	PROS+FORM	3	0.5532979707802	0.5641025641025	0.5956678700361	0.4837545126353	0.5357142857142
**Conduct Problems**	PROS+FORM	4	0.6121749594516	0.5940246045694	0.6101083032490	0.5543478260869	0.5787671232876
**Conduct Problems**	PROS+FORM	5	0.5820907235912	0.5935919055649	0.6376811594202	0.4909747292418	0.5552050473186
**Conduct Problems**	PROS+FORM	Mean	0.5755 ± 0.0215	0.5815 ± 0.0128	0.6127 ± 0.0144	0.5051 ± 0.0277	0.5535 ± 0.0160
**Conduct Problems**	MFCC+MELS+PROS	1	0.5652882221845	0.5818815331010	0.6028880866425	0.5306859205776	0.5622895622895
**Conduct Problems**	MFCC+MELS+PROS	2	0.5641413285719	0.5613382899628	0.5451263537906	0.6028880866425	0.5785440613026
**Conduct Problems**	MFCC+MELS+PROS	3	0.5494532706017	0.5619834710743	0.6137184115523	0.4296028880866	0.5182926829268

**Conduct Problems**	MFCC+MELS+PROS	4	0.598506252289	0.569872958257	0.566787003610	0.576086956521	0.572992700729
**Conduct Problems**	MFCC+MELS+PROS	5	0.568265055198	0.559859154929	0.576086956521	0.519855595667	0.544520547945
**Conduct Problems**	MFCC+MELS+PROS	Mean	0.5691 ± 0.0161	0.5670 ± 0.0082	0.5809 ± 0.0247	0.5318 ± 0.0593	0.5553 ± 0.0219
**Conduct Problems**	MFCC+MELS+FORM	1	0.545791030770	0.525939177101	0.530685920577	0.512635379061	0.521276595744
**Conduct Problems**	MFCC+MELS+FORM	2	0.559267030718	0.542495479204	0.541516245487	0.545126353790	0.543478260869
**Conduct Problems**	MFCC+MELS+FORM	3	0.568676771494	0.594871794871	0.628158844765	0.516245487364	0.564935064935
**Conduct Problems**	MFCC+MELS+FORM	4	0.603672892795	0.576642335766	0.570397111913	0.590579710144	0.583025830258
**Conduct Problems**	MFCC+MELS+FORM	5	0.591639198451	0.6	0.641304347826	0.505415162454	0.563694267515
**Conduct Problems**	MFCC+MELS+FORM	Mean	0.5738 ± 0.0211	0.5680 ± 0.0291	0.5824 ± 0.0448	0.5340 ± 0.0313	0.5553 ± 0.0211
**Conduct Problems**	MFCC+PROS+FORM	1	0.553193707724	0.558464223385	0.577617328519	0.509025270758	0.540540540540
**Conduct Problems**	MFCC+PROS+FORM	2	0.580693088662	0.559714795008	0.566787003610	0.541516245487	0.552816901408
**Conduct Problems**	MFCC+PROS+FORM	3	0.558823912731	0.567062818336	0.602888086642	0.476534296028	0.535256410256
**Conduct Problems**	MFCC+PROS+FORM	4	0.639473133469	0.602150537634	0.606498194945	0.590579710144	0.597864768683
**Conduct Problems**	MFCC+PROS+FORM	5	0.625555904358	0.620102214650	0.659420289855	0.534296028880	0.585209003215
**Conduct Problems**	MFCC+PROS+FORM	Mean	0.5915 ± 0.0350	0.5815 ± 0.0250	0.6026 ± 0.0321	0.5304 ± 0.0377	0.5623 ± 0.0248
**Conduct Problems**	MELS+PROS+FORM	1	0.554796752205	0.551126516464	0.574007220216	0.490974729241	0.53
**Conduct Problems**	MELS+PROS+FORM	2	0.554510028802	0.533094812164	0.537906137184	0.519855595667	0.528368794326
**Conduct Problems**	MELS+PROS+FORM	3	0.555630856651	0.543554006968	0.563176895306	0.490974729241	0.525252525252
**Conduct Problems**	MELS+PROS+FORM	4	0.601658557003	0.593128390596	0.592057761732	0.594202898550	0.594202898550
**Conduct Problems**	MELS+PROS+FORM	5	0.575066708522	0.573451327433	0.586956521739	0.541516245487	0.560553633217
**Conduct Problems**	MELS+PROS+FORM	Mean	0.5683 ± 0.0184	0.5589 ± 0.0217	0.5708 ± 0.0193	0.5275 ± 0.0384	0.5477 ± 0.0265
**Conduct Problems**	MFCC+MELS+PROS+	1	0.557650953355	0.529100529100	0.541516245487	0.494584837545	0.517241379310
**Conduct Problems**	MFCC+MELS+PROS+	2	0.570514407851	0.538599640933	0.541516245487	0.530685920577	0.535714285714
**Conduct Problems**	MFCC+MELS+PROS+	3	0.564493216384	0.565972222222	0.588447653429	0.509025270758	0.545150501672
**Conduct Problems**	MFCC+MELS+PROS+	4	0.614490137602	0.588447653429	0.588447653429	0.586956521739	0.588447653429
**Conduct Problems**	MFCC+MELS+PROS+	5	0.599761942133	0.610455311973	0.655797101449	0.509025270758	0.570977917981
**Conduct Problems**	MFCC+MELS+PROS+	Mean	0.5814 ± 0.0219	0.5665 ± 0.0303	0.5831 ± 0.0420	0.5261 ± 0.0326	0.5515 ± 0.0253
**Peer Problems**	MFCC	1	0.503284339229	0.526522593320	0.540322580645	0.487903225806	0.513409961685
**Peer Problems**	MFCC	2	0.603846904266	0.561886051080	0.576612903225	0.524193548387	0.547892720306
**Peer Problems**	MFCC	3	0.495333636836	0.496062992125	0.508064516129	0.459677419354	0.484615384615
**Peer Problems**	MFCC	4	0.546460754864	0.562015503875	0.584677419354	0.502024291497	0.541044776119
**Peer Problems**	MFCC	5	0.508260415306	0.503042596348	0.502024291497	0.508064516129	0.504065040650
**Peer Problems**	MFCC	Mean	0.5314 ± 0.0403	0.5299 ± 0.0280	0.5423 ± 0.0340	0.4964 ± 0.0217	0.5182 ± 0.0235
**Peer Problems**	MELS	1	0.543460587929	0.531958762886	0.520161290322	0.564516129032	0.544303797468
**Peer Problems**	MELS	2	0.569800338189	0.551440329218	0.540322580645	0.580645161290	0.563025210084
**Peer Problems**	MELS	3	0.532957206035	0.519114688128	0.520161290322	0.516129032258	0.518072289156
**Peer Problems**	MELS	4	0.539441034347	0.538775510204	0.532258064516	0.554655870445	0.545454545454
**Peer Problems**	MELS	5	0.539636933524	0.536489151873	0.550607287449	0.5	0.523076923076
**Peer Problems**	MELS	Mean	0.5451 ± 0.0128	0.5356 ± 0.0105	0.5327 ± 0.0118	0.5432 ± 0.0303	0.5388 ± 0.0164
**Peer Problems**	PROS	1	0.482830385015	0.463366336633	0.471774193548	0.435483870967	0.455252918287
**Peer Problems**	PROS	2	0.541168054110	0.579964850615	0.665322580645	0.370967741935	0.514018691588
**Peer Problems**	PROS	3	0.520047476586	0.556363636363	0.616935483870	0.399193548387	0.506622516556
**Peer Problems**	PROS	4	0.528552305080	0.544776119402	0.588709677419	0.425101214574	0.506944444444
**Peer Problems**	PROS	5	0.490302990727	0.524528301886	0.562753036437	0.419354838709	0.491166077738
**Peer Problems**	PROS	Mean	0.5126 ± 0.0224	0.5338 ± 0.0395	0.5811 ± 0.0644	0.4100 ± 0.0228	0.4948 ± 0.0211
**Peer Problems**	FORM	1	0.556045135275	0.537254901960	0.552419354838	0.495967741935	0.522900763358
**Peer Problems**	FORM	2	0.564174687825	0.557312252964	0.568548387096	0.528225806451	0.546511627906
**Peer Problems**	FORM	3	0.553882674297	0.540540540540	0.524193548387	0.584677419354	0.557939914163
**Peer Problems**	FORM	4	0.481830351312	0.506930693069	0.516129032258	0.477732793522	0.498054474708
**Peer Problems**	FORM	5	0.498187932610	0.506876227897	0.522267206477	0.463709677419	0.492366412213
**Peer Problems**	FORM	Mean	0.5308 ± 0.0339	0.5298 ± 0.0199	0.5367 ± 0.0202	0.5101 ± 0.0431	0.5236 ± 0.0258
**Peer Problems**	MFCC+MELS	1	0.523006633714	0.515463917525	0.504032258064	0.548387096774	0.527426160337
**Peer Problems**	MFCC+MELS	2	0.583035249739	0.552915766738	0.516129032258	0.649193548387	0.595348837209
**Peer Problems**	MFCC+MELS	3	0.510096904266	0.511156186612	0.508064516129	0.520161290322	0.514285714285
**Peer Problems**	MFCC+MELS	4	0.540094031605	0.531187122736	0.532258064516	0.526315789473	0.530120481927
**Peer Problems**	MFCC+MELS	5	0.541563275434	0.541501976284	0.554655870445	0.508064516129	0.528957528957
**Peer Problems**	MFCC+MELS	Mean	0.5396 ± 0.0246	0.5304 ± 0.0156	0.5230 ± 0.0185	0.5504 ± 0.0511	0.5392 ± 0.0286

**Peer Problems**	MFCC+PROS	1	0.4813345473465	0.5176470588235	0.5322580645161	0.4758064516129	0.5038167938931
**Peer Problems**	MFCC+PROS	2	0.5803362382934	0.5753424657534	0.5927419354838	0.5322580645161	0.5589353612167
**Peer Problems**	MFCC+PROS	3	0.4831555671175	0.4950099800399	0.5	0.4798387096774	0.4901185770750
**Peer Problems**	MFCC+PROS	4	0.5332212354708	0.5493230174081	0.5725806451612	0.4858299595141	0.5278810408921
**Peer Problems**	MFCC+PROS	5	0.4948576465978	0.4777327935222	0.4777327935222	0.4798387096774	0.4777327935222
**Peer Problems**	MFCC+PROS	Mean	0.5146 ± 0.0378	0.5230 ± 0.0355	0.5351 ± 0.0430	0.4907 ± 0.0210	0.5117 ± 0.0289
**Peer Problems**	MFCC+FORM	1	0.5231041883454	0.5288270377733	0.5362903225806	0.5080645161290	0.5215686274509
**Peer Problems**	MFCC+FORM	2	0.6036355359001	0.576	0.5806451612903	0.5645161290322	0.5714285714285
**Peer Problems**	MFCC+FORM	3	0.5102432362122	0.4929292929292	0.4919354838709	0.4959677419354	0.4939271255060
**Peer Problems**	MFCC+FORM	4	0.4989388794567	0.5168986083499	0.5241935483870	0.4939271255060	0.5098039215686
**Peer Problems**	MFCC+FORM	5	0.5044403813503	0.4869215291750	0.4898785425101	0.4798387096774	0.484
**Peer Problems**	MFCC+FORM	Mean	0.5281 ± 0.0386	0.5203 ± 0.0318	0.5246 ± 0.0333	0.5085 ± 0.0294	0.5161 ± 0.0305
**Peer Problems**	MELS+PROS	1	0.5342091571279	0.5104602510460	0.4919354838709	0.5645161290322	0.5304347826086
**Peer Problems**	MELS+PROS	2	0.5685809053069	0.5528455284552	0.5483870967741	0.5645161290322	0.5573770491803
**Peer Problems**	MELS+PROS	3	0.5288436524453	0.5069860279441	0.5120967741935	0.4919354838709	0.5019762845849
**Peer Problems**	MELS+PROS	4	0.5415306255713	0.5418326693227	0.5483870967741	0.5222672064777	0.5354330708661
**Peer Problems**	MELS+PROS	5	0.5414816507770	0.5553398058252	0.5789473684210	0.4959677419354	0.5335820895522
**Peer Problems**	MELS+PROS	Mean	0.5429 ± 0.0137	0.5335 ± 0.0208	0.5360 ± 0.0305	0.5278 ± 0.0317	0.5318 ± 0.0177
**Peer Problems**	MELS+FORM	1	0.5253804630593	0.5189620758483	0.5241935483870	0.5040322580645	0.5138339920948
**Peer Problems**	MELS+FORM	2	0.5709872528610	0.5432098765432	0.5322580645161	0.5725806451612	0.5546218487394
**Peer Problems**	MELS+FORM	3	0.5343554890738	0.5265306122448	0.5201612903225	0.5443548387096	0.5330578512396
**Peer Problems**	MELS+FORM	4	0.5115580514561	0.5140562248995	0.5161290322580	0.5060728744939	0.512
**Peer Problems**	MELS+FORM	5	0.5412367768055	0.5447154471544	0.5425101214574	0.5524193548387	0.5469387755102
**Peer Problems**	MELS+FORM	Mean	0.5367 ± 0.0198	0.5295 ± 0.0125	0.5271 ± 0.0094	0.5359 ± 0.0268	0.5321 ± 0.0171
**Peer Problems**	PROS+FORM	1	0.5175435744016	0.5060240963855	0.5080645161290	0.5	0.504
**Peer Problems**	PROS+FORM	2	0.5475741415192	0.5444015444015	0.5685483870967	0.4798387096774	0.5222222222222
**Peer Problems**	PROS+FORM	3	0.5505820759625	0.5289256198347	0.5161290322580	0.5645161290322	0.5423728813559
**Peer Problems**	PROS+FORM	4	0.4848831134909	0.488	0.4919354838709	0.4736842105263	0.4841269841269
**Peer Problems**	PROS+FORM	5	0.5065952722998	0.4989775051124	0.4939271255060	0.5161290322580	0.5041322314049
**Peer Problems**	PROS+FORM	Mean	0.5214 ± 0.0249	0.5133 ± 0.0205	0.5157 ± 0.0279	0.5068 ± 0.0325	0.5114 ± 0.0196
**Peer Problems**	MFCC+MELS+PROS	1	0.5147470083240	0.4876033057851	0.4758064516129	0.5241935483870	0.5
**Peer Problems**	MFCC+MELS+PROS	2	0.5912135796045	0.5358649789029	0.5120967741935	0.6008064516129	0.5619469026548
**Peer Problems**	MFCC+MELS+PROS	3	0.5298029396462	0.5132382892057	0.5080645161290	0.5282258064516	0.5185185185185
**Peer Problems**	MFCC+MELS+PROS	4	0.5391961603761	0.5422396856581	0.5564516129032	0.5020242914979	0.5287356321839
**Peer Problems**	MFCC+MELS+PROS	5	0.5569740107091	0.5469061876247	0.5546558704453	0.5282258064516	0.5393700787401
**Peer Problems**	MFCC+MELS+PROS	Mean	0.5464 ± 0.0263	0.5252 ± 0.0221	0.5214 ± 0.0306	0.5367 ± 0.0335	0.5297 ± 0.0207
**Peer Problems**	MFCC+MELS+FORM	1	0.5084059573361	0.5121951219512	0.5080645161290	0.5241935483870	0.5163934426229
**Peer Problems**	MFCC+MELS+FORM	2	0.5838644640998	0.5446985446985	0.5282258064516	0.5887096774193	0.5622317596566
**Peer Problems**	MFCC+MELS+FORM	3	0.5151372268470	0.4863731656184	0.4677419354838	0.5443548387096	0.5065502183406
**Peer Problems**	MFCC+MELS+FORM	4	0.5088317879064	0.5070993914807	0.5040322580645	0.5141700404858	0.5102040816326
**Peer Problems**	MFCC+MELS+FORM	5	0.5444201384354	0.5617529880478	0.5708502024291	0.5403225806451	0.5529411764705
**Peer Problems**	MFCC+MELS+FORM	Mean	0.5321 ± 0.0291	0.5224 ± 0.0271	0.5158 ± 0.0337	0.5424 ± 0.0256	0.5297 ± 0.0232
**Peer Problems**	MFCC+PROS+FORM	1	0.5071702653485	0.5182186234817	0.5161290322580	0.5241935483870	0.5203252032520
**Peer Problems**	MFCC+PROS+FORM	2	0.5902868106139	0.5579567779960	0.5725806451612	0.5201612903225	0.5440613026819
**Peer Problems**	MFCC+PROS+FORM	3	0.5112513007284	0.5061224489795	0.5	0.5241935483870	0.5123966942148
**Peer Problems**	MFCC+PROS+FORM	4	0.5038690087501	0.5267326732673	0.5362903225806	0.4979757085020	0.5175097276264
**Peer Problems**	MFCC+PROS+FORM	5	0.5043914065560	0.4989858012170	0.4979757085020	0.5040322580645	0.5
**Peer Problems**	MFCC+PROS+FORM	Mean	0.5234 ± 0.0335	0.5216 ± 0.0205	0.5246 ± 0.0277	0.5141 ± 0.0110	0.5189 ± 0.0144
**Peer Problems**	MELS+PROS+FORM	1	0.5133162070759	0.5	0.4919354838709	0.5241935483870	0.5083333333333
**Peer Problems**	MELS+PROS+FORM	2	0.5732797866805	0.5390946502057	0.5282258064516	0.5685483870967	0.5504201680672
**Peer Problems**	MELS+PROS+FORM	3	0.5280957336108	0.5195876288659	0.5080645161290	0.5524193548387	0.5316455696202
**Peer Problems**	MELS+PROS+FORM	4	0.5087011884550	0.4969199178644	0.4879032258064	0.5222672064777	0.5062761506276
**Peer Problems**	MELS+PROS+FORM	5	0.5413673762570	0.5439672801635	0.5384615384615	0.5604838709677	0.5495867768595
**Peer Problems**	MELS+PROS+FORM	Mean	0.5330 ± 0.0232	0.5199 ± 0.0193	0.5109 ± 0.0198	0.5456 ± 0.0190	0.5293 ± 0.0191
**Peer Problems**	MFCC+MELS+PROS+	1	0.5037395941727	0.4865979381443	0.4758064516129	0.5201612903225	0.4978902953586
**Peer Problems**	MFCC+MELS+PROS+	2	0.5940589229968	0.5443037974683	0.5201612903225	0.6088709677419	0.5707964601769
**Peer Problems**	MFCC+MELS+PROS+	3	0.5275104058272	0.5104602510460	0.4919354838709	0.5645161290322	0.5304347826086

**Peer Problems**	MFCC+MELS+PROS+	4	0.5156066344521	0.4878048780487	0.4838709677419	0.4979757085020	0.4918032786885
**Peer Problems**	MFCC+MELS+PROS+	5	0.5406164294109	0.54	0.5465587044534	0.5241935483870	0.5335968379446
**Peer Problems**	MFCC+MELS+PROS+	Mean	0.5363 ± 0.0314	0.5138 ± 0.0247	0.5037 ± 0.0261	0.5431 ± 0.0393	0.5249 ± 0.0284
LR_Male_detailed_performance_results							
**Symptom**	Subspace	Fold	AUC	F1	Sensitivity	Specificity	Precision
**Emotional Symptoms**	MFCC	1	0.5667336535633	0.5666266506602	0.5841584158415	0.5222772277227	0.5501165501165
**Emotional Symptoms**	MFCC	2	0.5644728457994	0.5610640870610	0.5742574257425	0.5272277227722	0.5484633569739
**Emotional Symptoms**	MFCC	3	0.5943412410547	0.5848375451263	0.6014851485148	0.5445544554455	0.5690866510538
**Emotional Symptoms**	MFCC	4	0.5882858757339	0.5696969696969	0.5831265508684	0.5371287128712	0.5568720379146
**Emotional Symptoms**	MFCC	5	0.5698412893398	0.5619631901840	0.5668316831683	0.5483870967741	0.5571776155717
**Emotional Symptoms**	MFCC	Mean	0.5767 ± 0.0122	0.5688 ± 0.0086	0.5820 ± 0.0116	0.5359 ± 0.0099	0.5563 ± 0.0073
**Emotional Symptoms**	MELS	1	0.5770819037349	0.5506883604505	0.5445544554455	0.5668316831683	0.5569620253164
**Emotional Symptoms**	MELS	2	0.5690312224291	0.5814771395076	0.6138613861386	0.5024752475247	0.5523385300668
**Emotional Symptoms**	MELS	3	0.5886922850700	0.6062801932367	0.6212871287128	0.5717821782178	0.5919811320754
**Emotional Symptoms**	MELS	4	0.5922167899172	0.5738498789346	0.5880893300248	0.5396039603960	0.5602836879432
**Emotional Symptoms**	MELS	5	0.5884210009090	0.5495608531994	0.5420792079207	0.5682382133995	0.5572519083969
**Emotional Symptoms**	MELS	Mean	0.5831 ± 0.0087	0.5724 ± 0.0211	0.5820 ± 0.0334	0.5498 ± 0.0263	0.5638 ± 0.0143
**Emotional Symptoms**	PROS	1	0.5224916674835	0.5574136008918	0.6188118811881	0.3985148514851	0.5070993914807
**Emotional Symptoms**	PROS	2	0.5613175178904	0.5856980703745	0.6386138613861	0.4579207920792	0.5408805031446
**Emotional Symptoms**	PROS	3	0.5849549063817	0.5971896955503	0.6311881188118	0.5173267326732	0.5666666666666
**Emotional Symptoms**	PROS	4	0.5446281600864	0.5734265734265	0.6104218362282	0.4826732673267	0.5406593406593
**Emotional Symptoms**	PROS	5	0.5672984792275	0.5704307334109	0.6064356435643	0.4789081885856	0.5384615384615
**Emotional Symptoms**	PROS	Mean	0.5561 ± 0.0212	0.5768 ± 0.0136	0.6211 ± 0.0122	0.4671 ± 0.0392	0.5388 ± 0.0189
**Emotional Symptoms**	FORM	1	0.5537263503578	0.5663924794359	0.5965346534653	0.4900990099009	0.5391498881431
**Emotional Symptoms**	FORM	2	0.5854144201548	0.5853658536585	0.6237623762376	0.4925742574257	0.5514223194748
**Emotional Symptoms**	FORM	3	0.5566733653563	0.5461077844311	0.5643564356435	0.4975247524752	0.5290023201856
**Emotional Symptoms**	FORM	4	0.6270790850797	0.6404494382022	0.7071960297766	0.5	0.5852156057494
**Emotional Symptoms**	FORM	5	0.5315271601601	0.5632839224629	0.6113861386138	0.4392059553349	0.5221987315010
**Emotional Symptoms**	FORM	Mean	0.5709 ± 0.0329	0.5803 ± 0.0325	0.6206 ± 0.0476	0.4839 ± 0.0226	0.5454 ± 0.0222
**Emotional Symptoms**	MFCC+MELS	1	0.5917863444760	0.5707317073170	0.5792079207920	0.5495049504950	0.5625
**Emotional Symptoms**	MFCC+MELS	2	0.6159445152436	0.5988304093567	0.6336633663366	0.5173267326732	0.5676274944567
**Emotional Symptoms**	MFCC+MELS	3	0.6409053034016	0.6125907990314	0.6262376237623	0.5816831683168	0.5995260663507
**Emotional Symptoms**	MFCC+MELS	4	0.6289401272633	0.6131025957972	0.6153846153846	0.6089108910891	0.6108374384236
**Emotional Symptoms**	MFCC+MELS	5	0.6350883227280	0.5889724310776	0.5816831683168	0.6054590570719	0.5964467005076
**Emotional Symptoms**	MFCC+MELS	Mean	0.6225 ± 0.0175	0.5968 ± 0.0159	0.6072 ± 0.0226	0.5726 ± 0.0348	0.5874 ± 0.0189
**Emotional Symptoms**	MFCC+PROS	1	0.5667459072639	0.5579710144927	0.5717821782178	0.5222772277227	0.5448113207547
**Emotional Symptoms**	MFCC+PROS	2	0.5759300558768	0.5704057279236	0.5915841584158	0.5173267326732	0.5506912442396
**Emotional Symptoms**	MFCC+PROS	3	0.6050693559454	0.5907990314769	0.6039603960396	0.5594059405940	0.5781990521327
**Emotional Symptoms**	MFCC+PROS	4	0.5895634228435	0.5728155339805	0.5856079404466	0.5420792079207	0.5605700712589
**Emotional Symptoms**	MFCC+PROS	5	0.5966022160528	0.5886610373944	0.6039603960396	0.5508684863523	0.5741176470588
**Emotional Symptoms**	MFCC+PROS	Mean	0.5868 ± 0.0138	0.5761 ± 0.0122	0.5914 ± 0.0121	0.5384 ± 0.0162	0.5617 ± 0.0129
**Emotional Symptoms**	MFCC+FORM	1	0.5993530046073	0.5903614457831	0.6064356435643	0.5519801980198	0.5751173708920
**Emotional Symptoms**	MFCC+FORM	2	0.5876139594157	0.5797101449275	0.5940594059405	0.5445544554455	0.5660377358490
**Emotional Symptoms**	MFCC+FORM	3	0.6046956180760	0.5847255369928	0.6064356435643	0.5321782178217	0.5645161290322
**Emotional Symptoms**	MFCC+FORM	4	0.6439328796403	0.6368977673325	0.6724565756823	0.5618811881188	0.6049107142857
**Emotional Symptoms**	MFCC+FORM	5	0.5993538559811	0.6263345195729	0.6534653465346	0.5657568238213	0.6013667425968
**Emotional Symptoms**	MFCC+FORM	Mean	0.6070 ± 0.0193	0.6036 ± 0.0234	0.6266 ± 0.0306	0.5513 ± 0.0121	0.5824 ± 0.0174
**Emotional Symptoms**	MELS+PROS	1	0.6072566415057	0.5953827460510	0.6064356435643	0.5693069306930	0.5847255369928
**Emotional Symptoms**	MELS+PROS	2	0.6085187726693	0.5945303210463	0.6188118811881	0.5371287128712	0.5720823798627
**Emotional Symptoms**	MELS+PROS	3	0.6269667189491	0.6038186157517	0.6262376237623	0.5519801980198	0.5829493087557
**Emotional Symptoms**	MELS+PROS	4	0.5944095029850	0.5826193390452	0.5905707196029	0.5643564356435	0.5748792270531
**Emotional Symptoms**	MELS+PROS	5	0.6264833058988	0.6002490660024	0.5965346534653	0.6079404466501	0.6040100250626
**Emotional Symptoms**	MELS+PROS	Mean	0.6127 ± 0.0125	0.5953 ± 0.0072	0.6077 ± 0.0133	0.5661 ± 0.0237	0.5837 ± 0.0112
**Emotional Symptoms**	MELS+FORM	1	0.6039113812371	0.5943970767356	0.6039603960396	0.5717821782178	0.5851318944844
**Emotional Symptoms**	MELS+FORM	2	0.6237072345848	0.6077738515901	0.6386138613861	0.5371287128712	0.5797752808988
**Emotional Symptoms**	MELS+FORM	3	0.6148968238408	0.5941320293398	0.6014851485148	0.5767326732673	0.5869565217391
**Emotional Symptoms**	MELS+FORM	4	0.6232894381249	0.5956416464891	0.6104218362282	0.5618811881188	0.5815602836879

Emotional Symptoms	MELS+FORM	5	0.614665995135	0.563591022443	0.559405940594	0.573200992555	0.567839195979
Emotional Symptoms	MELS+FORM	Mean	0.6161 ± 0.0072	0.5911 ± 0.0147	0.6028 ± 0.0254	0.5641 ± 0.0144	0.5803 ± 0.0067
Emotional Symptoms	PROS+FORM	1	0.562665424958	0.555423122765	0.576732673267	0.5	0.535632183908
Emotional Symptoms	PROS+FORM	2	0.600400696010	0.589073634204	0.613861386138	0.529702970297	0.566210045662
Emotional Symptoms	PROS+FORM	3	0.579403980001	0.583535108958	0.596534653465	0.551980198019	0.571090047393
Emotional Symptoms	PROS+FORM	4	0.619266393140	0.621809744779	0.665012406947	0.527227722772	0.583877995642
Emotional Symptoms	PROS+FORM	5	0.546464634056	0.558030480656	0.589108910891	0.476426799007	0.530066815144
Emotional Symptoms	PROS+FORM	Mean	0.5816 ± 0.0260	0.5816 ± 0.0242	0.6083 ± 0.0308	0.5171 ± 0.0262	0.5574 ± 0.0209
Emotional Symptoms	MFCC+MELS+PROS	1	0.601417753161	0.584951456310	0.596534653465	0.556930693069	0.573809523809
Emotional Symptoms	MFCC+MELS+PROS	2	0.623063915302	0.627634660421	0.663366336633	0.549504950495	0.595555555555
Emotional Symptoms	MFCC+MELS+PROS	3	0.638307518870	0.608272506082	0.618811881188	0.584158415841	0.598086124401
Emotional Symptoms	MFCC+MELS+PROS	4	0.635481414146	0.609223300970	0.622828784119	0.579207920792	0.596199524940
Emotional Symptoms	MFCC+MELS+PROS	5	0.649024641918	0.632850241545	0.648514851485	0.598014888337	0.617924528301
Emotional Symptoms	MFCC+MELS+PROS	Mean	0.6295 ± 0.0163	0.6126 ± 0.0169	0.6300 ± 0.0235	0.5736 ± 0.0179	0.5963 ± 0.0140
Emotional Symptoms	MFCC+MELS+FORM	1	0.627168905009	0.586929716399	0.589108910891	0.581683168316	0.584766584766
Emotional Symptoms	MFCC+MELS+FORM	2	0.617543623174	0.610250297973	0.633663366336	0.556930693069	0.588505747126
Emotional Symptoms	MFCC+MELS+FORM	3	0.641328056072	0.620192307692	0.638613861386	0.579207920792	0.602803738317
Emotional Symptoms	MFCC+MELS+FORM	4	0.651880696754	0.629453681710	0.657568238213	0.569306930693	0.603644646924
Emotional Symptoms	MFCC+MELS+FORM	5	0.644411959806	0.633495145631	0.646039603960	0.605459057071	0.621428571428
Emotional Symptoms	MFCC+MELS+FORM	Mean	0.6365 ± 0.0124	0.6161 ± 0.0166	0.6330 ± 0.0234	0.5785 ± 0.0160	0.6002 ± 0.0130
Emotional Symptoms	MFCC+PROS+FORM	1	0.602575727869	0.595382746051	0.606435643564	0.569306930693	0.584725536992
Emotional Symptoms	MFCC+PROS+FORM	2	0.590799921576	0.580335731414	0.599009900990	0.534653465346	0.562790697674
Emotional Symptoms	MFCC+PROS+FORM	3	0.604756886579	0.594724220623	0.613861386138	0.549504950495	0.576744186046
Emotional Symptoms	MFCC+PROS+FORM	4	0.642544775569	0.636792452830	0.669975186104	0.566831683168	0.606741573033
Emotional Symptoms	MFCC+PROS+FORM	5	0.591430607080	0.587951807228	0.603960396039	0.548387096774	0.572769953051
Emotional Symptoms	MFCC+PROS+FORM	Mean	0.6064 ± 0.0189	0.5990 ± 0.0196	0.6186 ± 0.0261	0.5537 ± 0.0128	0.5808 ± 0.0148
Emotional Symptoms	MELS+PROS+FORM	1	0.617598764826	0.593865030674	0.599009900990	0.581683168316	0.588807785888
Emotional Symptoms	MELS+PROS+FORM	2	0.639110136261	0.613207547169	0.643564356435	0.544554455445	0.585585585585
Emotional Symptoms	MELS+PROS+FORM	3	0.614020684246	0.601212121212	0.613861386138	0.571782178217	0.589073634204
Emotional Symptoms	MELS+PROS+FORM	4	0.620881753187	0.607866507747	0.632754342431	0.551980198019	0.584862385321
Emotional Symptoms	MELS+PROS+FORM	5	0.622951625187	0.599508599508	0.603960396039	0.588089330024	0.595121951219
Emotional Symptoms	MELS+PROS+FORM	Mean	0.6229 ± 0.0086	0.6031 ± 0.0067	0.6186 ± 0.0170	0.5676 ± 0.0168	0.5887 ± 0.0036
Emotional Symptoms	MFCC+MELS+PROS+	1	0.629674786785	0.596273291925	0.594059405940	0.601485148514	0.598503740648
Emotional Symptoms	MFCC+MELS+PROS+	2	0.619957602195	0.616647127784	0.650990099009	0.539603960396	0.585746102449
Emotional Symptoms	MFCC+MELS+PROS+	3	0.634018723654	0.598787878787	0.611386138613	0.569306930693	0.586698337292
Emotional Symptoms	MFCC+MELS+PROS+	4	0.646954769918	0.616844602609	0.645161290322	0.554455445544	0.590909090909
Emotional Symptoms	MFCC+MELS+PROS+	5	0.636464142692	0.621787025703	0.628712871287	0.605459057071	0.615012106537
Emotional Symptoms	MFCC+MELS+PROS+	Mean	0.6334 ± 0.0088	0.6101 ± 0.0104	0.6261 ± 0.0211	0.5741 ± 0.0258	0.5954 ± 0.0108
Hyperactivity	MFCC	1	0.613207694024	0.564814814814	0.555808656036	0.587699316628	0.574117647058
Hyperactivity	MFCC	2	0.614162442079	0.585635359116	0.603644646924	0.542141230068	0.568669527896
Hyperactivity	MFCC	3	0.639966583818	0.611607142857	0.624145785876	0.583143507972	0.599562363238
Hyperactivity	MFCC	4	0.592697184343	0.609913793103	0.646118721461	0.528473804100	0.577551020408
Hyperactivity	MFCC	5	0.596394878355	0.555304740406	0.560364464692	0.541095890410	0.550335570469
Hyperactivity	MFCC	Mean	0.6113 ± 0.0167	0.5855 ± 0.0229	0.5980 ± 0.0353	0.5565 ± 0.0241	0.5740 ± 0.0158
Hyperactivity	MELS	1	0.681980687107	0.631341600901	0.637813211845	0.617312072892	0.625
Hyperactivity	MELS	2	0.630792700328	0.608315098468	0.633257403189	0.551252847380	0.585263157894
Hyperactivity	MELS	3	0.640604812137	0.611111111111	0.626423690205	0.576309794988	0.596529284164
Hyperactivity	MELS	4	0.640236735627	0.617777777777	0.634703196347	0.580865603644	0.601731601731
Hyperactivity	MELS	5	0.636388221466	0.602892102335	0.617312072892	0.568493150684	0.589130434782
Hyperactivity	MELS	Mean	0.6460 ± 0.0183	0.6143 ± 0.0098	0.6299 ± 0.0073	0.5788 ± 0.0217	0.5995 ± 0.0140
Hyperactivity	PROS	1	0.608454709139	0.576388888888	0.567198177676	0.599088838268	0.585882352941
Hyperactivity	PROS	2	0.619506955650	0.624598930481	0.665148063781	0.535307517084	0.588709677419
Hyperactivity	PROS	3	0.591295188381	0.603613177470	0.646924829157	0.503416856492	0.565737051792
Hyperactivity	PROS	4	0.558747048605	0.599580712788	0.652968036529	0.476082004555	0.554263565891
Hyperactivity	PROS	5	0.586627973497	0.593147751605	0.630979498861	0.502283105022	0.559595959595
Hyperactivity	PROS	Mean	0.5929 ± 0.0208	0.5995 ± 0.0156	0.6326 ± 0.0345	0.5232 ± 0.0423	0.5708 ± 0.0139
Hyperactivity	FORM	1	0.606270204077	0.585526315789	0.608200455580	0.530751708428	0.564482029598

**Hyperactivity**	FORM	2	0.6002407625531	0.6078639744952	0.6514806378132	0.5079726651480	0.5697211155378
**Hyperactivity**	FORM	3	0.6411236969505	0.6293558606124	0.6788154897494	0.5216400911161	0.5866141732283
**Hyperactivity**	FORM	4	0.5908821418541	0.5993623804463	0.6438356164383	0.4965831435079	0.5606361829025
**Hyperactivity**	FORM	5	0.6285923799419	0.6281512605042	0.6810933940774	0.5114155251141	0.5828460038986
**Hyperactivity**	FORM	Mean	0.6134 ± 0.0186	0.6101 ± 0.0169	0.6527 ± 0.0266	0.5137 ± 0.0117	0.5729 ± 0.0102
**Hyperactivity**	MFCC+MELS	1	0.6896394269436	0.6418918918918	0.6492027334851	0.6264236902050	0.6347438752783
**Hyperactivity**	MFCC+MELS	2	0.6931885990628	0.6607538802660	0.6788154897494	0.6241457858769	0.6436285097192
**Hyperactivity**	MFCC+MELS	3	0.6808287628229	0.6558441558441	0.6902050113895	0.5854214123006	0.6247422680412
**Hyperactivity**	MFCC+MELS	4	0.6393006105615	0.6116611661166	0.6347031963470	0.5603644646924	0.5902335456475
**Hyperactivity**	MFCC+MELS	5	0.6756690693876	0.6379690949227	0.6583143507972	0.5936073059360	0.6188436830835
**Hyperactivity**	MFCC+MELS	Mean	0.6757 ± 0.0192	0.6416 ± 0.0172	0.6622 ± 0.0200	0.5980 ± 0.0248	0.6224 ± 0.0182
**Hyperactivity**	MFCC+PROS	1	0.6529750260739	0.608	0.6059225512528	0.6127562642369	0.6100917431192
**Hyperactivity**	MFCC+PROS	2	0.6619154113978	0.6126126126126	0.6195899772209	0.5968109339407	0.6057906458797
**Hyperactivity**	MFCC+PROS	3	0.6516466809532	0.6075388026607	0.6241457858769	0.5694760820045	0.5917926565874
**Hyperactivity**	MFCC+PROS	4	0.6167191936842	0.6032786885245	0.6301369863013	0.5421412300683	0.5786163522012
**Hyperactivity**	MFCC+PROS	5	0.6392121987497	0.6191536748329	0.6332574031890	0.5867579908675	0.6056644880174
**Hyperactivity**	MFCC+PROS	Mean	0.6445 ± 0.0157	0.6101 ± 0.0054	0.6226 ± 0.0096	0.5816 ± 0.0242	0.5984 ± 0.0117
**Hyperactivity**	MFCC+FORM	1	0.6699166152105	0.6300114547537	0.6264236902050	0.6378132118451	0.6336405529953
**Hyperactivity**	MFCC+FORM	2	0.6555953943784	0.6303162486368	0.6583143507972	0.5694760820045	0.6046025104602
**Hyperactivity**	MFCC+FORM	3	0.6888403443319	0.6467331118493	0.6651480637813	0.6082004555808	0.6293103448275
**Hyperactivity**	MFCC+FORM	4	0.6511737968192	0.6202393906420	0.6506849315068	0.5535307517084	0.5925155925155
**Hyperactivity**	MFCC+FORM	5	0.6486930653935	0.6377440347071	0.6697038724373	0.5684931506849	0.6086956521739
**Hyperactivity**	MFCC+FORM	Mean	0.6628 ± 0.0149	0.6330 ± 0.0088	0.6541 ± 0.0152	0.5875 ± 0.0310	0.6138 ± 0.0155
**Hyperactivity**	MELS+PROS	1	0.6946155322979	0.6401816118047	0.6423690205011	0.6355353075170	0.6380090497737
**Hyperactivity**	MELS+PROS	2	0.6478847660607	0.6233480176211	0.6446469248291	0.5763097949886	0.6034115138592
**Hyperactivity**	MELS+PROS	3	0.6598969494761	0.6301969365426	0.6560364464692	0.5740318906605	0.6063157894736
**Hyperactivity**	MELS+PROS	4	0.6515742503198	0.6329670329670	0.6575342465753	0.5808656036446	0.6101694915254
**Hyperactivity**	MELS+PROS	5	0.6707076065362	0.6356413166855	0.6378132118451	0.6301369863013	0.6334841628959
**Hyperactivity**	MELS+PROS	Mean	0.6649 ± 0.0168	0.6325 ± 0.0056	0.6477 ± 0.0078	0.5994 ± 0.0275	0.6183 ± 0.0145
**Hyperactivity**	MELS+FORM	1	0.7017501984734	0.6569178852643	0.6651480637813	0.6400911161731	0.6488888888888
**Hyperactivity**	MELS+FORM	2	0.6518438571821	0.6377440347071	0.6697038724373	0.5694760820045	0.6086956521739
**Hyperactivity**	MELS+FORM	3	0.7070324458673	0.6759956942949	0.7152619589977	0.5990888382687	0.6408163265306
**Hyperactivity**	MELS+FORM	4	0.6663390228934	0.6440306681270	0.6712328767123	0.5876993166287	0.6189473684210
**Hyperactivity**	MELS+FORM	5	0.6872562174306	0.6502793296089	0.6628701594533	0.6232876712328	0.6381578947368
**Hyperactivity**	MELS+FORM	Mean	0.6828 ± 0.0210	0.6530 ± 0.0132	0.6768 ± 0.0194	0.6039 ± 0.0251	0.6311 ± 0.0149
**Hyperactivity**	PROS+FORM	1	0.6377561345156	0.6174496644295	0.6287015945330	0.5922551252847	0.6065934065934
**Hyperactivity**	PROS+FORM	2	0.6176182149324	0.5989071038251	0.6241457858769	0.5398633257403	0.5756302521008
**Hyperactivity**	PROS+FORM	3	0.6510759076592	0.6400839454354	0.6947608200455	0.5239179954441	0.5933852140077
**Hyperactivity**	PROS+FORM	4	0.6236621212594	0.6097297297297	0.6438356164383	0.5330296127562	0.5790554414784
**Hyperactivity**	PROS+FORM	5	0.6303034085353	0.6314677930306	0.6810933940774	0.5228310502283	0.5885826771653
**Hyperactivity**	PROS+FORM	Mean	0.6321 ± 0.0116	0.6195 ± 0.0148	0.6545 ± 0.0284	0.5424 ± 0.0257	0.5886 ± 0.0110
**Hyperactivity**	MFCC+MELS+PROS	1	0.6987458554075	0.6508474576271	0.6560364464692	0.6400911161731	0.6457399103139
**Hyperactivity**	MFCC+MELS+PROS	2	0.6851925840982	0.6520787746170	0.6788154897494	0.5968109339407	0.6273684210526
**Hyperactivity**	MFCC+MELS+PROS	3	0.6924984822619	0.6585903083700	0.6810933940774	0.6127562642369	0.6375266524520
**Hyperactivity**	MFCC+MELS+PROS	4	0.6631509969731	0.6413043478260	0.6735159817351	0.5740318906605	0.6120331950207
**Hyperactivity**	MFCC+MELS+PROS	5	0.6885407890494	0.6425438596491	0.6674259681093	0.5890410958904	0.6194503171247
**Hyperactivity**	MFCC+MELS+PROS	Mean	0.6856 ± 0.0121	0.6491 ± 0.0064	0.6714 ± 0.0090	0.6025 ± 0.0225	0.6284 ± 0.0121
**Hyperactivity**	MFCC+MELS+FORM	1	0.7308648253174	0.6772366930917	0.6810933940774	0.6697038724373	0.6734234234234
**Hyperactivity**	MFCC+MELS+FORM	2	0.6929706674415	0.6716738197424	0.7129840546697	0.5899772209567	0.6348884381338
**Hyperactivity**	MFCC+MELS+FORM	3	0.7279746369103	0.6865342163355	0.7084282460136	0.6446469248291	0.6659528907922
**Hyperactivity**	MFCC+MELS+FORM	4	0.6984273098886	0.6773847802786	0.7214611872146	0.5922551252847	0.6383838383838
**Hyperactivity**	MFCC+MELS+FORM	5	0.7010952663275	0.6762749445676	0.6947608200455	0.6392694063926	0.6587473002159
**Hyperactivity**	MFCC+MELS+FORM	Mean	0.7103 ± 0.0159	0.6778 ± 0.0048	0.7037 ± 0.0142	0.6272 ± 0.0312	0.6543 ± 0.0152
**Hyperactivity**	MFCC+PROS+FORM	1	0.6764130530663	0.6362599771949	0.6355353075170	0.6378132118451	0.6369863013698
**Hyperactivity**	MFCC+PROS+FORM	2	0.6694911296641	0.6220735785953	0.6355353075170	0.5922551252847	0.6091703056768
**Hyperactivity**	MFCC+PROS+FORM	3	0.6884356141780	0.6482910694597	0.6697038724373	0.6036446469248	0.6282051282051
**Hyperactivity**	MFCC+PROS+FORM	4	0.6603166183002	0.6398258977149	0.6712328767123	0.5740318906605	0.6112266112266

**Hyperactivity**	MFCC+PROS+FORM	5	0.656171664534З	0.6406926406926	0.674259681093З	0.5684931506849	0.6103092783505
**Hyperactivity**	MFCC+PROS+FORM	Mean	0.6702 ± 0.0115	0.6374 ± 0.0086	0.6573 ± 0.0178	0.5952 ± 0.0247	0.6192 ± 0.0113
**Hyperactivity**	MELS+PROS+FORM	1	0.7119929846773	0.6725860155382	0.6902050113895	0.6378132118451	0.6558441558441
**Hyperactivity**	MELS+PROS+FORM	2	0.6621904203485	0.6480086114101	0.6856492027334	0.5694760820045	0.6142857142857
**Hyperactivity**	MELS+PROS+FORM	3	0.7182766797598	0.6725082146768	0.6993166287015	0.6195899772209	0.6476793248945
**Hyperactivity**	MELS+PROS+FORM	4	0.6823831663910	0.6558089033659	0.6894977168949	0.5876993166287	0.6252587991718
**Hyperactivity**	MELS+PROS+FORM	5	0.6954473117608	0.6320541760722	0.6378132118451	0.6187214611872	0.6263982102908
**Hyperactivity**	MELS+PROS+FORM	Mean	0.6941 ± 0.0203	0.6562 ± 0.0154	0.6805 ± 0.0218	0.6067 ± 0.0246	0.6339 ± 0.0154
**Hyperactivity**	MFCC+MELS+PROS+	1	0.7423788793125	0.7085201793721	0.7198177676537	0.6879271070615	0.6975717439293
**Hyperactivity**	MFCC+MELS+PROS+	2	0.6923116837293	0.6471226927252	0.6788154897494	0.5808656036446	0.6182572614107
**Hyperactivity**	MFCC+MELS+PROS+	3	0.7425604889970	0.7032967032967	0.7289293849658	0.6560364464692	0.6794055201698
**Hyperactivity**	MFCC+MELS+PROS+	4	0.7083658376759	0.6911447084233	0.7305936073059	0.6173120728929	0.6557377049180
**Hyperactivity**	MFCC+MELS+PROS+	5	0.7156052048553	0.6784922394678	0.6970387243735	0.6415525114155	0.6609071274298
**Hyperactivity**	MFCC+MELS+PROS+	Mean	0.7202 ± 0.0197	0.6857 ± 0.0219	0.7110 ± 0.0201	0.6367 ± 0.0361	0.6624 ± 0.0265
**Conduct Problems**	MFCC	1	0.5622978139398	0.5838709677419	0.5973597359735	0.5511551155115	0.5709779179810
**Conduct Problems**	MFCC	2	0.5388608397263	0.5425361155698	0.5596026490066	0.4983498349834	0.5264797507788
**Conduct Problems**	MFCC	3	0.5401722291434	0.5221674876847	0.5264900662251	0.5115511551155	0.5179153094462
**Conduct Problems**	MFCC	4	0.5386313465783	0.5442834138486	0.5577557755775	0.5066225165562	0.5314465408805
**Conduct Problems**	MFCC	5	0.5299433916901	0.5285481239804	0.5346534653465	0.5099337748344	0.5225806451612
**Conduct Problems**	MFCC	Mean	0.5420 ± 0.0108	0.5443 ± 0.0215	0.5552 ± 0.0247	0.5155 ± 0.0184	0.5339 ± 0.0191
**Conduct Problems**	MELS	1	0.5175418531952	0.5161290322580	0.5280528052805	0.4818481848184	0.5047318611987
**Conduct Problems**	MELS	2	0.5210040871636	0.5276872964169	0.5364238410596	0.5049504950495	0.5192307692307
**Conduct Problems**	MELS	3	0.4919895963106	0.5016501650165	0.5033112582781	0.4983498349834	0.5
**Conduct Problems**	MELS	4	0.4936725460625	0.5064102564102	0.5214521452145	0.4602649006622	0.4922118380062
**Conduct Problems**	MELS	5	0.4904487137455	0.5362776025236	0.5610561056105	0.4668874172185	0.5135951661631
**Conduct Problems**	MELS	Mean	0.5029 ± 0.0134	0.5176 ± 0.0129	0.5301 ± 0.0189	0.4825 ± 0.0173	0.5060 ± 0.0096
**Conduct Problems**	PROS	1	0.5517324009628	0.5673981191222	0.5973597359735	0.4917491749174	0.5402985074626
**Conduct Problems**	PROS	2	0.5235394400367	0.5543644716692	0.5993377483443	0.4389438943894	0.5156695156695
**Conduct Problems**	PROS	3	0.5322273949249	0.5454545454545	0.5761589403973	0.4653465346534	0.5178571428571
**Conduct Problems**	PROS	4	0.5415164032959	0.5589660743134	0.5709570957095	0.5264900662251	0.5474683544303
**Conduct Problems**	PROS	5	0.5381067908115	0.5476923076923	0.5874587458745	0.4403973509933	0.5129682997118
**Conduct Problems**	PROS	Mean	0.5374 ± 0.0094	0.5548 ± 0.0079	0.5863 ± 0.0112	0.4726 ± 0.0331	0.5269 ± 0.0142
**Conduct Problems**	FORM	1	0.5464170179394	0.5869894099848	0.6402640264026	0.4587458745874	0.5418994413407
**Conduct Problems**	FORM	2	0.4979236334229	0.5382262996941	0.5827814569536	0.4191419141914	0.5
**Conduct Problems**	FORM	3	0.5442703210718	0.5765765765765	0.6357615894039	0.4323432343234	0.5274725274725
**Conduct Problems**	FORM	4	0.5727602561580	0.5891238670694	0.6435643564356	0.4569536423841	0.5431754874651
**Conduct Problems**	FORM	5	0.5597119314580	0.6041055718475	0.6798679867986	0.4271523178807	0.5435356200527
**Conduct Problems**	FORM	Mean	0.5442 ± 0.0253	0.5790 ± 0.0222	0.6364 ± 0.0311	0.4389 ± 0.0161	0.5312 ± 0.0167
**Conduct Problems**	MFCC+MELS	1	0.5279220991406	0.5256410256410	0.5412541254125	0.4818481848184	0.5109034267912
**Conduct Problems**	MFCC+MELS	2	0.5123489170109	0.5033112582781	0.5033112582781	0.5049504950495	0.5033112582781
**Conduct Problems**	MFCC+MELS	3	0.5189495770769	0.5164473684210	0.5198675496688	0.5082508250825	0.5130718954248
**Conduct Problems**	MFCC+MELS	4	0.5240639958035	0.5388272583201	0.5610561056105	0.4768211920529	0.5182926829268
**Conduct Problems**	MFCC+MELS	5	0.5004589862959	0.4875621890547	0.4851485148514	0.4933774834437	0.49
**Conduct Problems**	MFCC+MELS	Mean	0.5167 ± 0.0097	0.5144 ± 0.0177	0.5221 ± 0.0269	0.4930 ± 0.0123	0.5071 ± 0.0098
**Conduct Problems**	MFCC+PROS	1	0.5638771797971	0.5727848101265	0.5973597359735	0.5115511551155	0.5501519756838
**Conduct Problems**	MFCC+PROS	2	0.5322711079054	0.5356576862123	0.5596026490066	0.4719471947194	0.5136778115501
**Conduct Problems**	MFCC+PROS	3	0.5436583393438	0.5397350993377	0.5397350993377	0.5412541254125	0.5397350993377
**Conduct Problems**	MFCC+PROS	4	0.5380193648503	0.5481727574750	0.5445544554455	0.5562913907284	0.5518394648829
**Conduct Problems**	MFCC+PROS	5	0.5173649815312	0.5024958402662	0.4983498349834	0.5132450331125	0.5067114093959
**Conduct Problems**	MFCC+PROS	Mean	0.5390 ± 0.0152	0.5398 ± 0.0227	0.5479 ± 0.0320	0.5189 ± 0.0289	0.5324 ± 0.0187
**Conduct Problems**	MFCC+FORM	1	0.5720898822555	0.5741626794258	0.5940594059405	0.5247524752475	0.5555555555555
**Conduct Problems**	MFCC+FORM	2	0.5008961161016	0.5008130081300	0.5099337748344	0.4752475247524	0.4920127795527
**Conduct Problems**	MFCC+FORM	3	0.5381832885275	0.5322580645161	0.5463576158940	0.4950495049504	0.5188679245283
**Conduct Problems**	MFCC+FORM	4	0.5614823071711	0.5493421052631	0.5511551155115	0.5430463576158	0.5475409836065
**Conduct Problems**	MFCC+FORM	5	0.5408060673617	0.5487012987012	0.5577557755775	0.5231788079470	0.5399361022364
**Conduct Problems**	MFCC+FORM	Mean	0.5427 ± 0.0244	0.5411 ± 0.0242	0.5519 ± 0.0268	0.5123 ± 0.0240	0.5308 ± 0.0229
**Conduct Problems**	MELS+PROS	1	0.5200906229236	0.5252854812398	0.5313531353135	0.5082508250825	0.5193548387096

Conduct Problems	MELS+PROS	2	0.520840163486	0.508361204013	0.503311258278	0.524752475247	0.513513513513
Conduct Problems	MELS+PROS	3	0.502262146744	0.500840336134	0.493377483443	0.524752475247	0.508532423208
Conduct Problems	MELS+PROS	4	0.523583153017	0.535885167464	0.554455445544	0.483443708609	0.518518518518
Conduct Problems	MELS+PROS	5	0.519638056520	0.548837209302	0.584158415841	0.453642384105	0.517543859649
Conduct Problems	MELS+PROS	Mean	0.5173 ± 0.0076	0.5238 ± 0.0176	0.5333 ± 0.0333	0.4990 ± 0.0272	0.5155 ± 0.0040
Conduct Problems	MELS+FORM	1	0.517008136457	0.494271685761	0.498349834983	0.481848184818	0.490259740259
Conduct Problems	MELS+FORM	2	0.516457937184	0.519736842105	0.523178807947	0.511551155115	0.516339869281
Conduct Problems	MELS+FORM	3	0.506862937949	0.521327014218	0.546357615894	0.452145214521	0.498489425981
Conduct Problems	MELS+FORM	4	0.526074792909	0.522022838499	0.528052805280	0.503311258278	0.516129032258
Conduct Problems	MELS+FORM	5	0.518501519026	0.560975609756	0.607260726072	0.440397350993	0.521246458923
Conduct Problems	MELS+FORM	Mean	0.5170 ± 0.0061	0.5237 ± 0.0214	0.5406 ± 0.0367	0.4779 ± 0.0278	0.5085 ± 0.0120
Conduct Problems	PROS+FORM	1	0.546569508435	0.561128526645	0.590759075907	0.485148514851	0.534328358208
Conduct Problems	PROS+FORM	2	0.511310733722	0.561456752655	0.612582781456	0.432343234323	0.518207282913
Conduct Problems	PROS+FORM	3	0.557165650339	0.592933947772	0.639072847682	0.485148514851	0.553008595988
Conduct Problems	PROS+FORM	4	0.563165256923	0.575471698113	0.603960396039	0.503311258278	0.549549549549
Conduct Problems	PROS+FORM	5	0.551362752169	0.586259541984	0.633663366336	0.470198675496	0.545454545454
Conduct Problems	PROS+FORM	Mean	0.5459 ± 0.0182	0.5755 ± 0.0128	0.6160 ± 0.0181	0.4752 ± 0.0239	0.5401 ± 0.0126
Conduct Problems	MFCC+MELS+PROS	1	0.523728610484	0.5344	0.551155115511	0.488448844884	0.518633540372
Conduct Problems	MFCC+MELS+PROS	2	0.537199746464	0.516339869281	0.523178807947	0.498349834983	0.509677419354
Conduct Problems	MFCC+MELS+PROS	3	0.541319694883	0.523489932885	0.516556291390	0.544554455445	0.530612244897
Conduct Problems	MFCC+MELS+PROS	4	0.520097042816	0.545741324921	0.570957095709	0.476821192052	0.522658610271
Conduct Problems	MFCC+MELS+PROS	5	0.505737328699	0.480672268907	0.471947194719	0.506622516556	0.489726027397
Conduct Problems	MFCC+MELS+PROS	Mean	0.5256 ± 0.0127	0.5201 ± 0.0221	0.5268 ± 0.0337	0.5030 ± 0.0231	0.5143 ± 0.0140
Conduct Problems	MFCC+MELS+FORM	1	0.541580890762	0.512077294685	0.524752475247	0.475247524752	0.5
Conduct Problems	MFCC+MELS+FORM	2	0.525298887504	0.516129032258	0.529801324503	0.478547854785	0.503144654088
Conduct Problems	MFCC+MELS+FORM	3	0.540674928419	0.563694267515	0.586092715231	0.508250825082	0.542944785276
Conduct Problems	MFCC+MELS+FORM	4	0.526424496754	0.530546623794	0.544554455445	0.490066225165	0.517241379310
Conduct Problems	MFCC+MELS+FORM	5	0.513911656066	0.537842190016	0.551155115511	0.5	0.525157232704
Conduct Problems	MFCC+MELS+FORM	Mean	0.5296 ± 0.0104	0.5321 ± 0.0184	0.5473 ± 0.0216	0.4904 ± 0.0125	0.5177 ± 0.0156
Conduct Problems	MFCC+PROS+FORM	1	0.563485061377	0.5632	0.580858085808	0.518151815181	0.546583850931
Conduct Problems	MFCC+PROS+FORM	2	0.513791445369	0.532051282051	0.549668874172	0.485148514851	0.515527950310
Conduct Problems	MFCC+PROS+FORM	3	0.549942080300	0.543089430894	0.552980132450	0.518151815181	0.533546325878
Conduct Problems	MFCC+PROS+FORM	4	0.552029375122	0.532445923460	0.528052805280	0.543046357615	0.536912751677
Conduct Problems	MFCC+PROS+FORM	5	0.528992634362	0.550161812297	0.561056105610	0.519867549668	0.539682539682
Conduct Problems	MFCC+PROS+FORM	Mean	0.5416 ± 0.0178	0.5442 ± 0.0117	0.5545 ± 0.0171	0.5169 ± 0.0185	0.5345 ± 0.0104
Conduct Problems	MELS+PROS+FORM	1	0.517171519132	0.504885993485	0.511551155115	0.485148514851	0.498392282958
Conduct Problems	MELS+PROS+FORM	2	0.520457674906	0.525285481239	0.533112582781	0.504950495049	0.517684887459
Conduct Problems	MELS+PROS+FORM	3	0.510993814613	0.5248	0.543046357615	0.475247524752	0.507739938080
Conduct Problems	MELS+PROS+FORM	4	0.532380390356	0.537216828478	0.547854785478	0.506622516556	0.526984126984
Conduct Problems	MELS+PROS+FORM	5	0.523812646165	0.583458646616	0.640264026402	0.443708609271	0.535911602209
Conduct Problems	MELS+PROS+FORM	Mean	0.5210 ± 0.0071	0.5351 ± 0.0263	0.5552 ± 0.0443	0.4831 ± 0.0230	0.5173 ± 0.0133
Conduct Problems	MFCC+MELS+PROS+	1	0.541406615909	0.529505582137	0.547854785478	0.478547854785	0.512345679012
Conduct Problems	MFCC+MELS+PROS+	2	0.528599217537	0.511400651465	0.519867549668	0.488448844884	0.503205128205
Conduct Problems	MFCC+MELS+PROS+	3	0.546445041855	0.558441558441	0.569536423841	0.531353135313	0.547770700636
Conduct Problems	MFCC+MELS+PROS+	4	0.527976307564	0.535885167464	0.554455445544	0.483443708609	0.518518518518
Conduct Problems	MFCC+MELS+PROS+	5	0.516556291390	0.543689320388	0.554455445544	0.513245033112	0.533333333333
Conduct Problems	MFCC+MELS+PROS+	Mean	0.5322 ± 0.0106	0.5358 ± 0.0156	0.5492 ± 0.0163	0.4990 ± 0.0201	0.5230 ± 0.0158
Peer Problems	MFCC	1	0.532590051457	0.546282245827	0.566037735849	0.495297805642	0.527859237536
Peer Problems	MFCC	2	0.495652688235	0.496815286624	0.489028213166	0.518867924528	0.504854368932
Peer Problems	MFCC	3	0.576421027649	0.546325878594	0.537735849056	0.569182389937	0.555194805194
Peer Problems	MFCC	4	0.534413195680	0.502446982055	0.484276729559	0.556603773584	0.522033898305
Peer Problems	MFCC	5	0.568163838455	0.562009419152	0.562893081761	0.559748427672	0.561128526645
Peer Problems	MFCC	Mean	0.5414 ± 0.0289	0.5308 ± 0.0261	0.5280 ± 0.0352	0.5399 ± 0.0282	0.5342 ± 0.0210
Peer Problems	MELS	1	0.509226947418	0.488745980707	0.477987421383	0.523510971786	0.5
Peer Problems	MELS	2	0.552276177520	0.521035598705	0.504702194357	0.566037735849	0.538461538461
Peer Problems	MELS	3	0.536618409081	0.513643659711	0.503144654088	0.544025157232	0.524590163934
Peer Problems	MELS	4	0.554438115580	0.527821939586	0.522012578616	0.544025157232	0.533762057877

**Peer Problems**	MELS	5	0.6050690241683	0.5682539682539	0.5628930817610	0.5817610062893	0.5737179487179
**Peer Problems**	MELS	Mean	0.5515 ± 0.0313	0.5239 ± 0.0258	0.5141 ± 0.0281	0.5519 ± 0.0201	0.5341 ± 0.0238
**Peer Problems**	PROS	1	0.5006801916366	0.4802527646129	0.4779874213836	0.4890282131661	0.4825396825396
**Peer Problems**	PROS	2	0.5179511444963	0.4562607204116	0.4169278996865	0.5880503144654	0.5037878787878
**Peer Problems**	PROS	3	0.5418496103793	0.5370370370370	0.5471698113207	0.5094339622641	0.5272727272727
**Peer Problems**	PROS	4	0.5348581939005	0.5440494590417	0.5534591194968	0.5188679245283	0.5349544072948
**Peer Problems**	PROS	5	0.5527669000435	0.5142857142857	0.4811320754716	0.6100628930817	0.5523465703971
**Peer Problems**	PROS	Mean	0.5296 ± 0.0184	0.5064 ± 0.0335	0.4953 ± 0.0504	0.5431 ± 0.0472	0.5202 ± 0.0244
**Peer Problems**	FORM	1	0.5180595808442	0.5491923641703	0.5880503144654	0.4482758620689	0.5151515151515
**Peer Problems**	FORM	2	0.5372133830168	0.5558912386706	0.5768025078369	0.5	0.5364431486880
**Peer Problems**	FORM	3	0.5570685495035	0.5826086956521	0.6320754716981	0.4622641509433	0.5403225806451
**Peer Problems**	FORM	4	0.5850935485146	0.5636363636363	0.5849056603773	0.5094339622641	0.5438596491228
**Peer Problems**	FORM	5	0.5555357778568	0.5875542691751	0.6383647798742	0.4654088050314	0.5442359249329
**Peer Problems**	FORM	Mean	0.5506 ± 0.0223	0.5678 ± 0.0149	0.6040 ± 0.0258	0.4771 ± 0.0235	0.5360 ± 0.0108
**Peer Problems**	MFCC+MELS	1	0.5193509591687	0.5	0.4874213836477	0.5391849529780	0.5132450331125
**Peer Problems**	MFCC+MELS	2	0.5382878886457	0.5089722675367	0.4890282131661	0.5660377358490	0.5306122448979
**Peer Problems**	MFCC+MELS	3	0.5566729955302	0.5153970826580	0.5	0.5597484276729	0.5317725752508
**Peer Problems**	MFCC+MELS	4	0.5412167240219	0.5283018867924	0.5283018867924	0.5283018867924	0.5283018867924
**Peer Problems**	MFCC+MELS	5	0.6087674538190	0.5818759936407	0.5754716981132	0.5974842767295	0.5884244372990
**Peer Problems**	MFCC+MELS	Mean	0.5529 ± 0.0304	0.5269 ± 0.0290	0.5160 ± 0.0331	0.5582 ± 0.0239	0.5385 ± 0.0259
**Peer Problems**	MFCC+PROS	1	0.5399440074131	0.5528700906344	0.5754716981132	0.4952978056426	0.5319767441860
**Peer Problems**	MFCC+PROS	2	0.5113956743755	0.5071315372424	0.5015673981191	0.5220125786163	0.5128205128205
**Peer Problems**	MFCC+PROS	3	0.5650092955183	0.5518341307814	0.5440251572327	0.5723270440251	0.5598705501618
**Peer Problems**	MFCC+PROS	4	0.5499683556821	0.5320512820512	0.5220125786163	0.5597484276729	0.5424836601307
**Peer Problems**	MFCC+PROS	5	0.5659981804517	0.5381026438569	0.5440251572327	0.5220125786163	0.5323076923076
**Peer Problems**	MFCC+PROS	Mean	0.5465 ± 0.0201	0.5364 ± 0.0167	0.5374 ± 0.0247	0.5343 ± 0.0280	0.5359 ± 0.0154
**Peer Problems**	MFCC+FORM	1	0.5430985193509	0.5426829268292	0.5597484276729	0.4984326018808	0.5266272189349
**Peer Problems**	MFCC+FORM	2	0.5378344275546	0.5254777070063	0.5172413793103	0.5471698113207	0.5339805825242
**Peer Problems**	MFCC+FORM	3	0.5936276254894	0.5779816513761	0.5943396226415	0.5377358490566	0.5625
**Peer Problems**	MFCC+FORM	4	0.5437383806020	0.5471698113207	0.5471698113207	0.5471698113207	0.5471698113207
**Peer Problems**	MFCC+FORM	5	0.5531624540168	0.5631659056316	0.5817610062893	0.5157232704402	0.5457227138643
**Peer Problems**	MFCC+FORM	Mean	0.5543 ± 0.0203	0.5513 ± 0.0179	0.5601 ± 0.0270	0.5292 ± 0.0192	0.5432 ± 0.0123
**Peer Problems**	MELS+PROS	1	0.5094438201139	0.4951140065146	0.4779874213836	0.5485893416927	0.5135135135135
**Peer Problems**	MELS+PROS	2	0.5531436683030	0.5244299674267	0.5047021943573	0.5786163522012	0.5457627118644
**Peer Problems**	MELS+PROS	3	0.5394070645939	0.5154975530179	0.4968553459119	0.5691823899371	0.5355932203389
**Peer Problems**	MELS+PROS	4	0.5450535975633	0.5263157894736	0.5345911949685	0.5031446540880	0.5182926829268
**Peer Problems**	MELS+PROS	5	0.5857264348720	0.5705521472392	0.5849056603773	0.5345911949685	0.5568862275449
**Peer Problems**	MELS+PROS	Mean	0.5466 ± 0.0245	0.5264 ± 0.0247	0.5198 ± 0.0373	0.5468 ± 0.0267	0.5340 ± 0.0163
**Peer Problems**	MELS+FORM	1	0.5357741369452	0.5253456221198	0.5377358490566	0.4921630094043	0.5135135135135
**Peer Problems**	MELS+FORM	2	0.5480077285542	0.5041186161449	0.4796238244514	0.5754716981132	0.53125
**Peer Problems**	MELS+FORM	3	0.5625271943356	0.5372670807453	0.5440251572327	0.5188679245283	0.5306748466257
**Peer Problems**	MELS+FORM	4	0.5673232862624	0.5616224648985	0.5660377358490	0.5503144654088	0.5572755417956
**Peer Problems**	MELS+FORM	5	0.5967623907282	0.6101190476190	0.6446540880503	0.5314465408805	0.5790960451977
**Peer Problems**	MELS+FORM	Mean	0.5621 ± 0.0206	0.5477 ± 0.0363	0.5544 ± 0.0534	0.5337 ± 0.0282	0.5424 ± 0.0231
**Peer Problems**	PROS+FORM	1	0.5303326038524	0.5497076023391	0.5911949685534	0.4420062695924	0.5136612021857
**Peer Problems**	PROS+FORM	2	0.5518720056781	0.5426829268292	0.5579937304075	0.5	0.5281899109792
**Peer Problems**	PROS+FORM	3	0.5525691230568	0.5672082717872	0.6037735849056	0.4748427672955	0.5348189415041
**Peer Problems**	PROS+FORM	4	0.5643071872156	0.5574272588055	0.5723270440251	0.5188679245283	0.5432835820895
**Peer Problems**	PROS+FORM	5	0.5753826984692	0.6081871345029	0.6540880503144	0.5031446540880	0.5683060109289
**Peer Problems**	PROS+FORM	Mean	0.5549 ± 0.0150	0.5650 ± 0.0231	0.5959 ± 0.0331	0.4878 ± 0.0269	0.5377 ± 0.0181
**Peer Problems**	MFCC+MELS+PROS	1	0.5230180792965	0.5080385852090	0.4968553459119	0.5423197492163	0.5197368421052
**Peer Problems**	MFCC+MELS+PROS	2	0.5475739831627	0.5296950240770	0.5172413793103	0.5628930817610	0.5427631578947
**Peer Problems**	MFCC+MELS+PROS	3	0.5545370040742	0.5273311897106	0.5157232704402	0.5597484276729	0.5394736842105
**Peer Problems**	MFCC+MELS+PROS	4	0.5494739132154	0.5271317829457	0.5345911949685	0.5062893081761	0.5198776758409
**Peer Problems**	MFCC+MELS+PROS	5	0.5925398520628	0.5548589341692	0.5566037735849	0.5503144654088	0.553125
**Peer Problems**	MFCC+MELS+PROS	Mean	0.5534 ± 0.0224	0.5294 ± 0.0149	0.5242 ± 0.0201	0.5443 ± 0.0203	0.5350 ± 0.0132
**Peer Problems**	MFCC+MELS+FORM	1	0.5471008063721	0.5454545454545	0.5471698113207	0.5423197492163	0.54375

**Peer Problems**	MFCC+MELS+FORM	2	0.5506200587527	0.5298869143786	0.5141065830721	0.5723270440251	0.5466666666666
**Peer Problems**	MFCC+MELS+FORM	3	0.5847869941853	0.5821596244131	0.5849056603773	0.5754716981132	0.5794392523364
**Peer Problems**	MFCC+MELS+FORM	4	0.5524306791661	0.5541795665634	0.5628930817610	0.5314465408805	0.5457317073170
**Peer Problems**	MFCC+MELS+FORM	5	0.5918575214588	0.5674418604651	0.5754716981132	0.5471698113207	0.5596330275229
**Peer Problems**	MFCC+MELS+FORM	Mean	0.5654 ± 0.0190	0.5558 ± 0.0180	0.5569 ± 0.0249	0.5537 ± 0.0173	0.5550 ± 0.0134
**Peer Problems**	MFCC+PROS+FORM	1	0.5297411328641	0.5374045801526	0.5534591194968	0.4952978056426	0.5222551928783
**Peer Problems**	MFCC+PROS+FORM	2	0.5464699039845	0.5171849427168	0.4952978056426	0.5786163522012	0.5410958904109
**Peer Problems**	MFCC+PROS+FORM	3	0.5890985324947	0.5924812030075	0.6194968553459	0.5283018867924	0.5677233429394
**Peer Problems**	MFCC+PROS+FORM	4	0.5400201732526	0.5625	0.5660377358490	0.5534591194968	0.5590062111801
**Peer Problems**	MFCC+PROS+FORM	5	0.5512736837941	0.5864661654135	0.6132075471698	0.5220125786163	0.5619596541786
**Peer Problems**	MFCC+PROS+FORM	Mean	0.5513 ± 0.0202	0.5592 ± 0.0286	0.5695 ± 0.0451	0.5355 ± 0.0284	0.5504 ± 0.0167
**Peer Problems**	MELS+PROS+FORM	1	0.5311705210859	0.5321100917431	0.5471698113207	0.4921630094043	0.5178571428571
**Peer Problems**	MELS+PROS+FORM	2	0.5537449971412	0.5363489499192	0.5203761755485	0.5786163522012	0.5533333333333
**Peer Problems**	MELS+PROS+FORM	3	0.5606186464142	0.5515151515151	0.5723270440251	0.4968553459119	0.5321637426900
**Peer Problems**	MELS+PROS+FORM	4	0.5598967604129	0.5462962962962	0.5566037735849	0.5188679245283	0.5363636363636
**Peer Problems**	MELS+PROS+FORM	5	0.5913531901427	0.5800604229607	0.6037735849056	0.5220125786163	0.5581395348837
**Peer Problems**	MELS+PROS+FORM	Mean	0.5594 ± 0.0192	0.5493 ± 0.0169	0.5601 ± 0.0276	0.5217 ± 0.0308	0.5396 ± 0.0146
**Peer Problems**	MFCC+MELS+PROS+	1	0.5427042053587	0.5142857142857	0.5094339622641	0.5297805642633	0.5192307692307
**Peer Problems**	MFCC+MELS+PROS+	2	0.5441237357307	0.5286624203821	0.5203761755485	0.5503144654088	0.5372168284789
**Peer Problems**	MFCC+MELS+PROS+	3	0.5841046635813	0.5664556962025	0.5628930817610	0.5754716981132	0.5700636942675
**Peer Problems**	MFCC+MELS+PROS+	4	0.5487124718167	0.5507692307692	0.5628930817610	0.5188679245283	0.5391566265060
**Peer Problems**	MFCC+MELS+PROS+	5	0.597316662908	0.5871559633027	0.6037735849056	0.5471698113207	0.5714285714285
**Peer Problems**	MFCC+MELS+PROS+	Mean	0.5634 ± 0.0228	0.5495 ± 0.0260	0.5519 ± 0.0339	0.5443 ± 0.0194	0.5474 ± 0.0203
SVM_Female_detailed_performance_results							
**Emotional Symptoms**	MFCC	1	0.6041220283644	0.5911330049261	0.6060606060606	0.5555555555555	0.5769230769230
**Emotional Symptoms**	MFCC	2	0.5801675565985	0.5482233502538	0.5454545454545	0.5555555555555	0.5510204081632
**Emotional Symptoms**	MFCC	3	0.5947238943871	0.5925925925925	0.5925925925925	0.5925925925925	0.5925925925925
**Emotional Symptoms**	MFCC	4	0.6038538538538	0.5690235690235	0.5709459459459	0.5656565656565	0.5671140939597
**Emotional Symptoms**	MFCC	5	0.6066748566748	0.6109324758842	0.6397306397306	0.5439189189189	0.5846153846153
**Emotional Symptoms**	MFCC	Mean	0.5979 ± 0.0098	0.5824 ± 0.0216	0.5910 ± 0.0319	0.5627 ± 0.0165	0.5745 ± 0.0144
**Emotional Symptoms**	MELS	1	0.5536056411477	0.5480769230769	0.5757575757575	0.4747474747474	0.5229357798165
**Emotional Symptoms**	MELS	2	0.5854844747333	0.5681444991789	0.5824915824915	0.5319865319865	0.5544871794871
**Emotional Symptoms**	MELS	3	0.5259780747990	0.5400981996726	0.5555555555555	0.4983164983164	0.5254777070063
**Emotional Symptoms**	MELS	4	0.5708776958776	0.5469798657718	0.5506756756756	0.5387205387205	0.5433333333333
**Emotional Symptoms**	MELS	5	0.5531213031213	0.5672609400324	0.5892255892255	0.5101351351351	0.546875
**Emotional Symptoms**	MELS	Mean	0.5578 ± 0.0199	0.5541 ± 0.0114	0.5707 ± 0.0151	0.5108 ± 0.0232	0.5386 ± 0.0123
**Emotional Symptoms**	PROS	1	0.5695904046072	0.5582922824302	0.5723905723905	0.5218855218855	0.5448717948717
**Emotional Symptoms**	PROS	2	0.5743744969334	0.5778491171749	0.6060606060606	0.5084175084175	0.5521472392638
**Emotional Symptoms**	PROS	3	0.5180083664932	0.5668789808917	0.5993265993265	0.4848484848484	0.5377643504531
**Emotional Symptoms**	PROS	4	0.5469446719446	0.5663430420711	0.5912162162162	0.5050505050505	0.5434782608695
**Emotional Symptoms**	PROS	5	0.5188142688142	0.5166666666666	0.5218855218855	0.5	0.5115511551155
**Emotional Symptoms**	PROS	Mean	0.5455 ± 0.0240	0.5572 ± 0.0212	0.5782 ± 0.0303	0.5040 ± 0.0120	0.5380 ± 0.0140
**Emotional Symptoms**	FORM	1	0.5139724971374	0.5162337662337	0.5353535353535	0.4612794612794	0.4984326018808
**Emotional Symptoms**	FORM	2	0.5803716174086	0.5737976782752	0.5824915824915	0.5521885521885	0.5653594771241
**Emotional Symptoms**	FORM	3	0.5398655465995	0.5867895545314	0.6430976430976	0.4511784511784	0.5395480225988
**Emotional Symptoms**	FORM	4	0.5429975429975	0.5902140672782	0.6520270270270	0.4444444444444	0.5391061452513
**Emotional Symptoms**	FORM	5	0.5334880334880	0.5555555555555	0.5892255892255	0.4662162162162	0.5255255255255
**Emotional Symptoms**	FORM	Mean	0.5421 ± 0.0216	0.5645 ± 0.0270	0.6004 ± 0.0428	0.4751 ± 0.0393	0.5336 ± 0.0218
**Emotional Symptoms**	MFCC+MELS	1	0.5858359124352	0.5657894736842	0.5791245791245	0.5319865319865	0.5530546623794
**Emotional Symptoms**	MFCC+MELS	2	0.5889081612987	0.5563139931740	0.5488215488215	0.5757575757575	0.5640138408304
**Emotional Symptoms**	MFCC+MELS	3	0.5878311736897	0.5662847790507	0.5824915824915	0.5252525252525	0.5509554140127
**Emotional Symptoms**	MFCC+MELS	4	0.6061174811174	0.5762144053601	0.5810810810810	0.5656565656565	0.5714285714285
**Emotional Symptoms**	MFCC+MELS	5	0.5921034671034	0.5958132045088	0.6228956228956	0.5304054054054	0.5709876543209
**Emotional Symptoms**	MFCC+MELS	Mean	0.5922 ± 0.0073	0.5721 ± 0.0134	0.5829 ± 0.0236	0.5458 ± 0.0207	0.5621 ± 0.0087
**Emotional Symptoms**	MFCC+PROS	1	0.6008570554025	0.5882352941176	0.6060606060606	0.5454545454545	0.5714285714285
**Emotional Symptoms**	MFCC+PROS	2	0.5881372649049	0.5622895622895	0.5622895622895	0.5622895622895	0.5622895622895
**Emotional Symptoms**	MFCC+PROS	3	0.5904726275090	0.5460636515912	0.5488215488215	0.5387205387205	0.5433333333333

**Emotional Symptoms**	MFCC+PROS	4	0.6072549822549	0.5799011532125	0.5945945945945	0.5454545454545	0.5659163987138
**Emotional Symptoms**	MFCC+PROS	5	0.5909090909090	0.5666666666666	0.5723905723905	0.5506756756756	0.5610561056105
**Emotional Symptoms**	MFCC+PROS	Mean	0.5955 ± 0.0073	0.5686 ± 0.0146	0.5768 ± 0.0209	0.5485 ± 0.0079	0.5608 ± 0.0095
**Emotional Symptoms**	MFCC+FORM	1	0.5878991939598	0.5958132045088	0.6228956228956	0.5319865319865	0.5709876543209
**Emotional Symptoms**	MFCC+FORM	2	0.5702932807309	0.5501730103806	0.5353535353535	0.5892255892255	0.5658362989323
**Emotional Symptoms**	MFCC+FORM	3	0.5949279551973	0.5666104553119	0.5656565656565	0.5690235690235	0.5675675675675
**Emotional Symptoms**	MFCC+FORM	4	0.6111566111566	0.5830618892508	0.6047297297297	0.5319865319865	0.5628930817610
**Emotional Symptoms**	MFCC+FORM	5	0.6035353535353	0.6032786885245	0.6195286195286	0.5641891891891	0.5878594249201
**Emotional Symptoms**	MFCC+FORM	Mean	0.5936 ± 0.0140	0.5798 ± 0.0193	0.5896 ± 0.0339	0.5573 ± 0.0223	0.5710 ± 0.0088
**Emotional Symptoms**	MELS+PROS	1	0.5471890623405	0.5460526315789	0.5589225589225	0.5117845117845	0.5337620578778
**Emotional Symptoms**	MELS+PROS	2	0.5640694260222	0.5344537815126	0.5353535353535	0.5319865319865	0.5335570469798
**Emotional Symptoms**	MELS+PROS	3	0.5413960026754	0.5594855305466	0.5858585858585	0.4915824915824	0.5353846153846
**Emotional Symptoms**	MELS+PROS	4	0.5851988351988	0.5787781350482	0.6081081081081	0.5084175084175	0.5521472392638
**Emotional Symptoms**	MELS+PROS	5	0.5186891436891	0.5285481239804	0.5454545454545	0.4797297297297	0.5126582278481
**Emotional Symptoms**	MELS+PROS	Mean	0.5513 ± 0.0223	0.5495 ± 0.0181	0.5667 ± 0.0267	0.5047 ± 0.0179	0.5335 ± 0.0125
**Emotional Symptoms**	MELS+FORM	1	0.5564058089310	0.5806451612903	0.6060606060606	0.5185185185185	0.5572755417956
**Emotional Symptoms**	MELS+FORM	2	0.5780362548039	0.5695142378559	0.5723905723905	0.5622895622895	0.5666666666666
**Emotional Symptoms**	MELS+FORM	3	0.5449784035642	0.5582137161084	0.5892255892255	0.4781144781144	0.5303030303030
**Emotional Symptoms**	MELS+FORM	4	0.5618345618345	0.5520661157024	0.5641891891891	0.5218855218855	0.5404530744336
**Emotional Symptoms**	MELS+FORM	5	0.5672604422604	0.5868852459016	0.6026936026936	0.5472972972972	0.5718849840255
**Emotional Symptoms**	MELS+FORM	Mean	0.5617 ± 0.0110	0.5695 ± 0.0131	0.5869 ± 0.0164	0.5256 ± 0.0288	0.5533 ± 0.0157
**Emotional Symptoms**	PROS+FORM	1	0.5332449069822	0.5619047619047	0.5959595959595	0.4747474747474	0.5315315315315
**Emotional Symptoms**	PROS+FORM	2	0.5754401478307	0.5769854132901	0.5993265993265	0.5218855218855	0.55625
**Emotional Symptoms**	PROS+FORM	3	0.5286648754662	0.5610142630744	0.5959595959595	0.4713804713804	0.5299401197604
**Emotional Symptoms**	PROS+FORM	4	0.5618231868231	0.5795275590551	0.6216216216216	0.4781144781144	0.5427728613569
**Emotional Symptoms**	PROS+FORM	5	0.5308149058149	0.5422077922077	0.5622895622895	0.4864864864864	0.5235109717868
**Emotional Symptoms**	PROS+FORM	Mean	0.5460 ± 0.0190	0.5643 ± 0.0134	0.5950 ± 0.0190	0.4865 ± 0.0184	0.5368 ± 0.0115
**Emotional Symptoms**	MFCC+MELS+PROS	1	0.5987257536079	0.5728155339805	0.5959595959595	0.5151515151515	0.5514018691588
**Emotional Symptoms**	MFCC+MELS+PROS	2	0.5970025734335	0.5834738617200	0.5824915824915	0.5858585858585	0.5844594594594
**Emotional Symptoms**	MFCC+MELS+PROS	3	0.5967191556417	0.5836065573770	0.5993265993265	0.5454545454545	0.5686900958466
**Emotional Symptoms**	MFCC+MELS+PROS	4	0.6202452452452	0.5933333333333	0.6013513513513	0.5757575757575	0.5855263157894
**Emotional Symptoms**	MFCC+MELS+PROS	5	0.5990990990990	0.5863708399366	0.6228956228956	0.4966216216216	0.5538922155688
**Emotional Symptoms**	MFCC+MELS+PROS	Mean	0.6024 ± 0.0090	0.5839 ± 0.0066	0.6004 ± 0.0130	0.5438 ± 0.0342	0.5688 ± 0.0145
**Emotional Symptoms**	MFCC+MELS+FORM	1	0.5863687378838	0.5751633986928	0.5925925925925	0.5319865319865	0.5587301587301
**Emotional Symptoms**	MFCC+MELS+FORM	2	0.5800995363284	0.5724020442930	0.5656565656565	0.5892255892255	0.5793103448275
**Emotional Symptoms**	MFCC+MELS+FORM	3	0.5773673888152	0.55	0.5555555555555	0.5353535353535	0.5445544554455
**Emotional Symptoms**	MFCC+MELS+FORM	4	0.6202566202566	0.5872483221476	0.5912162162162	0.5791245791245	0.5833333333333
**Emotional Symptoms**	MFCC+MELS+FORM	5	0.5928087178087	0.5601317957166	0.5723905723905	0.5270270270270	0.5483870967741
**Emotional Symptoms**	MFCC+MELS+FORM	Mean	0.5914 ± 0.0154	0.5690 ± 0.0128	0.5755 ± 0.0144	0.5525 ± 0.0262	0.5629 ± 0.0158
**Emotional Symptoms**	MFCC+PROS+FORM	1	0.5851443730231	0.5939004815409	0.6228956228956	0.5252525252525	0.5674846625766
**Emotional Symptoms**	MFCC+PROS+FORM	2	0.5769139203482	0.5473321858864	0.5353535353535	0.5791245791245	0.5598591549295
**Emotional Symptoms**	MFCC+PROS+FORM	3	0.5882052851749	0.5732899022801	0.5925925925925	0.5252525252525	0.5552050473186
**Emotional Symptoms**	MFCC+PROS+FORM	4	0.6098371098371	0.5836065573770	0.6013513513513	0.5420875420875	0.5668789808917
**Emotional Symptoms**	MFCC+PROS+FORM	5	0.6056738556738	0.5884297520661	0.5993265993265	0.5608108108108	0.5779220779220
**Emotional Symptoms**	MFCC+PROS+FORM	Mean	0.5932 ± 0.0125	0.5773 ± 0.0165	0.5903 ± 0.0293	0.5465 ± 0.0209	0.5655 ± 0.0077
**Emotional Symptoms**	MELS+PROS+FORM	1	0.5598861794147	0.5603864734299	0.5858585858585	0.4949494949494	0.5370370370370
**Emotional Symptoms**	MELS+PROS+FORM	2	0.6019907265698	0.5884297520661	0.5993265993265	0.5622895622895	0.5779220779220
**Emotional Symptoms**	MELS+PROS+FORM	3	0.5436520082984	0.5512820512820	0.5791245791245	0.4781144781144	0.5259938837920
**Emotional Symptoms**	MELS+PROS+FORM	4	0.5726294476294	0.5625	0.5777027027027	0.5252525252525	0.5480769230769
**Emotional Symptoms**	MELS+PROS+FORM	5	0.5649513149513	0.5700325732899	0.5892255892255	0.5202702702702	0.5520504731861
**Emotional Symptoms**	MELS+PROS+FORM	Mean	0.5686 ± 0.0192	0.5665 ± 0.0125	0.5862 ± 0.0078	0.5162 ± 0.0287	0.5482 ± 0.0174
**Emotional Symptoms**	MFCC+MELS+PROS+	1	0.5921617975490	0.5774877650897	0.5959595959595	0.5319865319865	0.5601265822784
**Emotional Symptoms**	MFCC+MELS+PROS+	2	0.5930007142128	0.5724137931034	0.5589225589225	0.6060606060606	0.5865724381625
**Emotional Symptoms**	MFCC+MELS+PROS+	3	0.5846002108628	0.5568369028006	0.5690235690235	0.5252525252525	0.5451612903225
**Emotional Symptoms**	MFCC+MELS+PROS+	4	0.6278210028210	0.5996649916247	0.6047297297297	0.5892255892255	0.5946843853820
**Emotional Symptoms**	MFCC+MELS+PROS+	5	0.5996223496223	0.5847176079734	0.5925925925925	0.5641891891891	0.5770491803278
**Emotional Symptoms**	MFCC+MELS+PROS+	Mean	0.5994 ± 0.0150	0.5782 ± 0.0141	0.5842 ± 0.0173	0.5633 ± 0.0314	0.5727 ± 0.0180

**Hyperactivity**	MFCC	1	0.6517707339826	0.628205128205	0.6464379947229	0.5883905013192	0.6109725685785
**Hyperactivity**	MFCC	2	0.630913179384	0.6177215189873	0.6437994722955	0.5593667546174	0.5936739659367
**Hyperactivity**	MFCC	3	0.6801609568298	0.6529562982005	0.6701846965699	0.617414248021	0.6365914786967
**Hyperactivity**	MFCC	4	0.618496181820	0.6161745827984	0.6349206349206	0.5751978891820	0.5985037406483
**Hyperactivity**	MFCC	5	0.6429548659100	0.6211340206185	0.635883905013	0.5873015873015	0.6070528967254
**Hyperactivity**	MFCC	Mean	0.6449 ± 0.0209	0.6272 ± 0.0135	0.6462 ± 0.0128	0.5855 ± 0.0191	0.6094 ± 0.0149
**Hyperactivity**	MELS	1	0.643110254036	0.6090322580645	0.6226912928759	0.5778364116094	0.5959595959595
**Hyperactivity**	MELS	2	0.630105610515	0.5916230366492	0.5963060686015	0.5804749340369	0.5870129870129
**Hyperactivity**	MELS	3	0.6376034697614	0.6222791293213	0.6411609498680	0.5804749340369	0.6044776119402
**Hyperactivity**	MELS	4	0.6485879018860	0.6534653465346	0.6984126984126	0.5620052770448	0.6139534883720
**Hyperactivity**	MELS	5	0.625518281191	0.6212871287128	0.6622691292875	0.5291005291005	0.5850815850815
**Hyperactivity**	MELS	Mean	0.6370 ± 0.0084	0.6195 ± 0.0203	0.6442 ± 0.0347	0.5660 ± 0.0197	0.5973 ± 0.0108
**Hyperactivity**	PROS	1	0.6026343453470	0.5861635220125	0.6147757255936	0.5171503957783	0.560096153846
**Hyperactivity**	PROS	2	0.618980653156	0.629861982434	0.6622691292875	0.5593667546174	0.6004784688995
**Hyperactivity**	PROS	3	0.5991882540500	0.6105527638190	0.6411609498680	0.5408970976253	0.5827338129496
**Hyperactivity**	PROS	4	0.5802655274950	0.5883807169344	0.6296296296296	0.4907651715039	0.552204176334
**Hyperactivity**	PROS	5	0.6112856165620	0.6231527093596	0.6675461741424	0.5238095238095	0.5842956120092
**Hyperactivity**	PROS	Mean	0.6025 ± 0.0131	0.6076 ± 0.0177	0.6431 ± 0.0198	0.5264 ± 0.0231	0.5760 ± 0.0175
**Hyperactivity**	FORM	1	0.606672189695	0.6108998732572	0.635883905013	0.5540897097625	0.587804878048
**Hyperactivity**	FORM	2	0.6427412780604	0.642857142857	0.6886543535620	0.5461741424802	0.6027713625866
**Hyperactivity**	FORM	3	0.6271050744564	0.6216560509554	0.6437994722955	0.5725593667546	0.6009852216748
**Hyperactivity**	FORM	4	0.6510588990800	0.6296296296296	0.674603174603	0.5329815303430	0.590277777777
**Hyperactivity**	FORM	5	0.6285058145210	0.6328800988875	0.6754617414248	0.5396825396825	0.5953488372093
**Hyperactivity**	FORM	Mean	0.6312 ± 0.0152	0.6276 ± 0.0108	0.6637 ± 0.0202	0.5491 ± 0.0137	0.5954 ± 0.0058
**Hyperactivity**	MFCC+MELS	1	0.6929010519280	0.633507853403	0.6385224274406	0.6226912928759	0.6285714285714
**Hyperactivity**	MFCC+MELS	2	0.6529681636858	0.659231722428	0.7018469656992	0.5725593667546	0.6214953271028
**Hyperactivity**	MFCC+MELS	3	0.6985888430183	0.6701164294954	0.683377308707	0.6437994722955	0.6573604060913
**Hyperactivity**	MFCC+MELS	4	0.6411958509583	0.6338383838383	0.664021164021	0.569920844327	0.606280193236
**Hyperactivity**	MFCC+MELS	5	0.6623389314682	0.648030495552	0.6728232189973	0.5952380952380	0.625
**Hyperactivity**	MFCC+MELS	Mean	0.6696 ± 0.0224	0.6489 ± 0.0143	0.6721 ± 0.0210	0.6008 ± 0.0287	0.6277 ± 0.0166
**Hyperactivity**	MFCC+PROS	1	0.6917453930284	0.6439790575916	0.6490765171503	0.6332453825857	0.6389610389610
**Hyperactivity**	MFCC+PROS	2	0.6492157531623	0.626598465473	0.6464379947229	0.5831134564643	0.6079404466501
**Hyperactivity**	MFCC+PROS	3	0.6854449634853	0.6625	0.699208443271	0.5883905013192	0.6294536817102
**Hyperactivity**	MFCC+PROS	4	0.6605729362985	0.6358974358974	0.6560846560846	0.593667546174	0.6169154228855
**Hyperactivity**	MFCC+PROS	5	0.674219262609	0.654639175257	0.6701846965699	0.621693121693	0.6397984886649
**Hyperactivity**	MFCC+PROS	Mean	0.6722 ± 0.0157	0.6447 ± 0.0128	0.6642 ± 0.0193	0.6040 ± 0.0198	0.6266 ± 0.0125
**Hyperactivity**	MFCC+FORM	1	0.6546459576304	0.6332046332046	0.6490765171503	0.5989445910290	0.6180904522613
**Hyperactivity**	MFCC+FORM	2	0.6761718450860	0.6507537688442	0.683377308707	0.5831134564643	0.6211031175059
**Hyperactivity**	MFCC+FORM	3	0.7137237975229	0.6777920410783	0.6965699208443	0.6411609498680	0.66
**Hyperactivity**	MFCC+FORM	4	0.6781770462509	0.650246305418	0.6984126984126	0.5514511873350	0.6082949308755
**Hyperactivity**	MFCC+FORM	5	0.6655219109044	0.6381322957198	0.6490765171503	0.6137566137566	0.627551020408
**Hyperactivity**	MFCC+FORM	Mean	0.6776 ± 0.0199	0.6500 ± 0.0155	0.6753 ± 0.0220	0.5977 ± 0.0300	0.6270 ± 0.0176
**Hyperactivity**	MELS+PROS	1	0.6269310294414	0.5879265091863	0.591029023746	0.5804749340369	0.5848563968668
**Hyperactivity**	MELS+PROS	2	0.657827500504	0.6218274111675	0.6464379947229	0.567282321899	0.5990220048899
**Hyperactivity**	MELS+PROS	3	0.6579736983173	0.637419354838	0.6517150395778	0.6068601583113	0.6237373737373
**Hyperactivity**	MELS+PROS	4	0.6478898800798	0.6461159062885	0.6931216931216	0.5488126649076	0.605080831408
**Hyperactivity**	MELS+PROS	5	0.6394507964428	0.6267166042446	0.6622691292875	0.5476190476190	0.5947867298578
**Hyperactivity**	MELS+PROS	Mean	0.6460 ± 0.0118	0.6240 ± 0.0199	0.6489 ± 0.0332	0.5702 ± 0.0220	0.6015 ± 0.0129
**Hyperactivity**	MELS+FORM	1	0.6934231869730	0.6700379266750	0.699208443271	0.6121372031662	0.643203883495
**Hyperactivity**	MELS+FORM	2	0.6665506366560	0.6329442282749	0.6437994722955	0.609498680738	0.6224489795918
**Hyperactivity**	MELS+FORM	3	0.6771256117682	0.6467532467532	0.656992084432	0.6253298153034	0.6368286445012
**Hyperactivity**	MELS+FORM	4	0.670505786600	0.6516853932584	0.690476190476	0.5725593667546	0.617021276595
**Hyperactivity**	MELS+FORM	5	0.6721600982814	0.6616541353383	0.6965699208443	0.5899470899470	0.6300715990453
**Hyperactivity**	MELS+FORM	Mean	0.6760 ± 0.0094	0.6526 ± 0.0127	0.6774 ± 0.0226	0.6019 ± 0.0185	0.6299 ± 0.0094
**Hyperactivity**	PROS+FORM	1	0.633064375770	0.6138107416879	0.6332453825857	0.569920844327	0.5955334987593
**Hyperactivity**	PROS+FORM	2	0.6753225054128	0.6292993630573	0.6517150395778	0.5804749340369	0.6083743842364
**Hyperactivity**	PROS+FORM	3	0.6477259278339	0.6235741444866	0.6490765171503	0.567282321899	0.6

**Hyperactivity**	PROS+FORM	4	0.6433457581214	0.6305418719211	0.6772486772486	0.5303430079155	0.5898617511520
**Hyperactivity**	PROS+FORM	5	0.6401557984671	0.6289308176100	0.6596306068601	0.5608465608465	0.6009615384615
**Hyperactivity**	PROS+FORM	Mean	0.6479 ± 0.0145	0.6252 ± 0.0062	0.6542 ± 0.0144	0.5618 ± 0.0169	0.5989 ± 0.0061
**Hyperactivity**	MFCC+MELS+PROS	1	0.7048475017578	0.6563307493540	0.6701846965699	0.6279683377308	0.6430379746835
**Hyperactivity**	MFCC+MELS+PROS	2	0.6627494935290	0.6724351050679	0.7176781002638	0.5831134564643	0.6325581395348
**Hyperactivity**	MFCC+MELS+PROS	3	0.6789635271266	0.6547770700636	0.6781002638522	0.6068601583113	0.6330049261083
**Hyperactivity**	MFCC+MELS+PROS	4	0.6587790202565	0.6345177664974	0.6613756613756	0.5778364116094	0.6097560975609
**Hyperactivity**	MFCC+MELS+PROS	5	0.6838938448437	0.6489898989898	0.6781002638522	0.5873015873015	0.6222760290556
**Hyperactivity**	MFCC+MELS+PROS	Mean	0.6778 ± 0.0165	0.6534 ± 0.0122	0.6811 ± 0.0193	0.5966 ± 0.0185	0.6281 ± 0.0113
**Hyperactivity**	MFCC+MELS+FORM	1	0.7019513927082	0.6683738796414	0.6886543535620	0.6279683377308	0.6492537313432
**Hyperactivity**	MFCC+MELS+FORM	2	0.6757750224518	0.6354166666666	0.6437994722955	0.6174142480211	0.6272493573264
**Hyperactivity**	MFCC+MELS+FORM	3	0.7155338656790	0.6743886743886	0.6912928759894	0.6411609498680	0.6582914572864
**Hyperactivity**	MFCC+MELS+FORM	4	0.6964652175733	0.6691635455680	0.7089947089947	0.5910290237467	0.6335697399527
**Hyperactivity**	MFCC+MELS+FORM	5	0.6957811562033	0.6757763975155	0.7176781002638	0.5925925925925	0.6384976525821
**Hyperactivity**	MFCC+MELS+FORM	Mean	0.6971 ± 0.0128	0.6646 ± 0.0149	0.6901 ± 0.0256	0.6140 ± 0.0196	0.6414 ± 0.0111
**Hyperactivity**	MFCC+PROS+FORM	1	0.6721131153361	0.6391752577319	0.6543535620052	0.6068601583113	0.6246851385390
**Hyperactivity**	MFCC+PROS+FORM	2	0.6877214722815	0.6675126903553	0.6939313984168	0.6147757255936	0.6430317848410
**Hyperactivity**	MFCC+PROS+FORM	3	0.7193767796102	0.6801007556675	0.7124010554089	0.6174142480211	0.6506024096385
**Hyperactivity**	MFCC+PROS+FORM	4	0.6889684633747	0.6666666666666	0.7195767195767	0.5620052770448	0.6210045662100
**Hyperactivity**	MFCC+PROS+FORM	5	0.6805852214823	0.6354166666666	0.6437994722955	0.6164021164021	0.6272493573264
**Hyperactivity**	MFCC+PROS+FORM	Mean	0.6898 ± 0.0160	0.6578 ± 0.0174	0.6848 ± 0.0305	0.6035 ± 0.0211	0.6333 ± 0.0115
**Hyperactivity**	MELS+PROS+FORM	1	0.6956440013645	0.6750948166877	0.7044854881266	0.6174142480211	0.6480582524271
**Hyperactivity**	MELS+PROS+FORM	2	0.6732687742357	0.6348387096774	0.6490765171503	0.6042216358839	0.6212121212121
**Hyperactivity**	MELS+PROS+FORM	3	0.6833355379035	0.6407263294422	0.6517150395778	0.6174142480211	0.6301020408163
**Hyperactivity**	MELS+PROS+FORM	4	0.6800058633831	0.6558197747183	0.6931216931216	0.5804749340369	0.6223277909738
**Hyperactivity**	MELS+PROS+FORM	5	0.6864416244363	0.6708385481852	0.7071240105540	0.5978835978835	0.6380952380952
**Hyperactivity**	MELS+PROS+FORM	Mean	0.6837 ± 0.0074	0.6555 ± 0.0159	0.6811 ± 0.0255	0.6035 ± 0.0138	0.6320 ± 0.0101
**Hyperactivity**	MFCC+MELS+PROS+	1	0.7171002708140	0.6831168831168	0.6939313984168	0.6622691292875	0.6726342710997
**Hyperactivity**	MFCC+MELS+PROS+	2	0.6890372525950	0.6572890025575	0.6781002638522	0.6147757255936	0.6377171215880
**Hyperactivity**	MFCC+MELS+PROS+	3	0.7184717455322	0.6828025477707	0.7071240105540	0.6358839050131	0.6600985221674
**Hyperactivity**	MFCC+MELS+PROS+	4	0.7117449149111	0.6840796019900	0.7275132275132	0.6015831134564	0.6455399061032
**Hyperactivity**	MFCC+MELS+PROS+	5	0.7035291982521	0.6757425742574	0.7203166226912	0.5873015873015	0.6363636363636
**Hyperactivity**	MFCC+MELS+PROS+	Mean	0.7080 ± 0.0108	0.6766 ± 0.0101	0.7054 ± 0.0178	0.6204 ± 0.0264	0.6505 ± 0.0139
**Conduct Problems**	MFCC	1	0.5088819090565	0.5244122965641	0.5234657039711	0.5270758122743	0.5253623188405
**Conduct Problems**	MFCC	2	0.5485409688644	0.5314183123877	0.5342960288808	0.5234657039711	0.5285714285714
**Conduct Problems**	MFCC	3	0.5292653364438	0.5594639865996	0.6028880866425	0.4476534296028	0.521875
**Conduct Problems**	MFCC	4	0.5999843038769	0.5971223021582	0.5992779783393	0.5905797101449	0.5949820788530
**Conduct Problems**	MFCC	5	0.5736932977554	0.5807560137457	0.6123188405797	0.5054151624548	0.5522875816993
**Conduct Problems**	MFCC	Mean	0.5521 ± 0.0321	0.5586 ± 0.0279	0.5744 ± 0.0376	0.5188 ± 0.0458	0.5446 ± 0.0274
**Conduct Problems**	MELS	1	0.5192821488615	0.5375218150087	0.5559566787003	0.4873646209386	0.5202702702702
**Conduct Problems**	MELS	2	0.5001629110245	0.4982206405693	0.5054151624548	0.4765342960288	0.4912280701754
**Conduct Problems**	MELS	3	0.5216150347326	0.5667215815486	0.6209386281588	0.4296028880866	0.5212121212121
**Conduct Problems**	MELS	4	0.5120598545492	0.5291970802919	0.5234657039711	0.5434782608695	0.5350553505535
**Conduct Problems**	MELS	5	0.5414770051797	0.5379061371841	0.5398550724637	0.5342960288808	0.5359712230215
**Conduct Problems**	MELS	Mean	0.5189 ± 0.0135	0.5339 ± 0.0219	0.5491 ± 0.0396	0.4943 ± 0.0414	0.5207 ± 0.0162
**Conduct Problems**	PROS	1	0.5417508373626	0.5491525423728	0.5848375451263	0.4548736462093	0.5175718849840
**Conduct Problems**	PROS	2	0.5258246556060	0.5199306759098	0.5415162454873	0.4584837545126	0.5
**Conduct Problems**	PROS	3	0.5104328220099	0.5349650349650	0.5523465703971	0.4873646209386	0.5186440677966
**Conduct Problems**	PROS	4	0.5724899283210	0.5516014234875	0.5595667870036	0.5289855072463	0.5438596491228
**Conduct Problems**	PROS	5	0.5217652906398	0.5177304964539	0.5289855072463	0.4873646209386	0.5069444444444
**Conduct Problems**	PROS	Mean	0.5345 ± 0.0215	0.5347 ± 0.0141	0.5535 ± 0.0188	0.4834 ± 0.0266	0.5174 ± 0.0149
**Conduct Problems**	FORM	1	0.5385056497543	0.5494880546075	0.5812274368231	0.4657039711191	0.5210355987055
**Conduct Problems**	FORM	2	0.5666306090265	0.5699831365935	0.6101083032490	0.4693140794223	0.5348101265822
**Conduct Problems**	FORM	3	0.5480066207040	0.5776293823038	0.6245487364620	0.4620938628158	0.5372670807453
**Conduct Problems**	FORM	4	0.5814497985664	0.5778546712802	0.6028880866425	0.5144927536231	0.5548172757475
**Conduct Problems**	FORM	5	0.6013315544393	0.5841924398625	0.6159420289855	0.5090252707581	0.5555555555555
**Conduct Problems**	FORM	Mean	0.5672 ± 0.0226	0.5718 ± 0.0120	0.6069 ± 0.0147	0.4841 ± 0.0227	0.5407 ± 0.0131

**Conduct Problems**	MFCC+MELS	1	0.5234787368530	0.5197132616487	0.5234657039711	0.5090252707581	0.5160142348754
**Conduct Problems**	MFCC+MELS	2	0.5396916420127	0.5203539823008	0.5306859205776	0.4909747292418	0.5104166666666
**Conduct Problems**	MFCC+MELS	3	0.5209633906345	0.5442176870748	0.5776173285198	0.4548736462093	0.5144694533762
**Conduct Problems**	MFCC+MELS	4	0.5778396902631	0.5719360568383	0.5812274368231	0.5471014492753	0.5629370629370
**Conduct Problems**	MFCC+MELS	5	0.5474153717365	0.5594405594405	0.5797101449275	0.5090252707581	0.5405405405405
**Conduct Problems**	MFCC+MELS	Mean	0.5419 ± 0.0205	0.5431 ± 0.0208	0.5585 ± 0.0258	0.5022 ± 0.0299	0.5289 ± 0.0200
**Conduct Problems**	MFCC+PROS	1	0.5470552203208	0.5604203152364	0.5776173285198	0.5162454873646	0.5442176870748
**Conduct Problems**	MFCC+PROS	2	0.5720522879224	0.5366726296958	0.5415162454873	0.5234657039711	0.5319148936170
**Conduct Problems**	MFCC+PROS	3	0.5610916341930	0.5878378378378	0.6281588447653	0.4909747292418	0.5523809523809
**Conduct Problems**	MFCC+PROS	4	0.6301470203526	0.5992779783393	0.5992779783393	0.5978260869565	0.5992779783393
**Conduct Problems**	MFCC+PROS	5	0.5969628001883	0.5982905982905	0.6340579710144	0.5162454873646	0.5663430420711
**Conduct Problems**	MFCC+PROS	Mean	0.5815 ± 0.0293	0.5765 ± 0.0244	0.5961 ± 0.0341	0.5290 ± 0.0362	0.5588 ± 0.0231
**Conduct Problems**	MFCC+FORM	1	0.5353516923197	0.5283687943262	0.5379061371841	0.5018050541516	0.5191637630662
**Conduct Problems**	MFCC+FORM	2	0.5742157463279	0.5679012345679	0.5812274368231	0.5342960288808	0.5551724137931
**Conduct Problems**	MFCC+FORM	3	0.5562955336313	0.5640138408304	0.5884476534296	0.5018050541516	0.5415282392026
**Conduct Problems**	MFCC+FORM	4	0.6198398995448	0.6	0.6064981949458	0.5833333333333	0.5936395759717
**Conduct Problems**	MFCC+FORM	5	0.6291398524564	0.6148531951640	0.6449275362318	0.5487364620938	0.5874587458745
**Conduct Problems**	MFCC+FORM	Mean	0.5830 ± 0.0362	0.5750 ± 0.0302	0.5918 ± 0.0348	0.5340 ± 0.0307	0.5594 ± 0.0280
**Conduct Problems**	MELS+PROS	1	0.5530373131410	0.5441941074523	0.5667870036101	0.4837545126353	0.5233333333333
**Conduct Problems**	MELS+PROS	2	0.5339571739498	0.5045703839122	0.4981949458483	0.5234657039711	0.5111111111111
**Conduct Problems**	MELS+PROS	3	0.5356123499589	0.5365025466893	0.5703971119133	0.4440433212996	0.5064102564102
**Conduct Problems**	MELS+PROS	4	0.5618688850520	0.5064695009242	0.4945848375451	0.5398550724637	0.5189393939393
**Conduct Problems**	MELS+PROS	5	0.5616596034112	0.5569620253164	0.5579710144927	0.5559566787003	0.5559566787003
**Conduct Problems**	MELS+PROS	Mean	0.5492 ± 0.0122	0.5297 ± 0.0208	0.5376 ± 0.0339	0.5094 ± 0.0406	0.5232 ± 0.0174
**Conduct Problems**	MELS+FORM	1	0.5505349998045	0.5563380281690	0.5703971119133	0.5198555956678	0.5429553264604
**Conduct Problems**	MELS+FORM	2	0.5445920056302	0.5215827338129	0.5234657039711	0.5162454873646	0.5197132616487
**Conduct Problems**	MELS+FORM	3	0.5433929804897	0.5403508771929	0.5559566787003	0.4981949458483	0.5255972696245
**Conduct Problems**	MELS+FORM	4	0.5837257364097	0.5787545787545	0.5703971119133	0.5978260869565	0.5873605947955
**Conduct Problems**	MELS+FORM	5	0.5886830952754	0.5923344947735	0.6159420289855	0.5379061371841	0.5704697986577
**Conduct Problems**	MELS+FORM	Mean	0.5622 ± 0.0198	0.5579 ± 0.0255	0.5672 ± 0.0298	0.5340 ± 0.0343	0.5492 ± 0.0260
**Conduct Problems**	PROS+FORM	1	0.5512909069582	0.5680272108843	0.6028880866425	0.4801444043321	0.5369774919614
**Conduct Problems**	PROS+FORM	2	0.5775260983461	0.5778546712802	0.6028880866425	0.5162454873646	0.5548172757475
**Conduct Problems**	PROS+FORM	3	0.5570775065490	0.5586206896551	0.5848375451263	0.4909747292418	0.5346534653465
**Conduct Problems**	PROS+FORM	4	0.6060534714592	0.5804195804195	0.5992779783393	0.5326086956521	0.5627118644067
**Conduct Problems**	PROS+FORM	5	0.5834118139486	0.5962521294718	0.6340579710144	0.5090252707581	0.5627009646302
**Conduct Problems**	PROS+FORM	Mean	0.5751 ± 0.0196	0.5762 ± 0.0126	0.6048 ± 0.0161	0.5058 ± 0.0185	0.5504 ± 0.0123
**Conduct Problems**	MFCC+MELS+PROS	1	0.5648581370798	0.5639229422066	0.5812274368231	0.5198555956678	0.5476190476190
**Conduct Problems**	MFCC+MELS+PROS	2	0.5737726283413	0.5553470919324	0.5342960288808	0.6101083032490	0.578125
**Conduct Problems**	MFCC+MELS+PROS	3	0.5477068644189	0.5614617940199	0.6101083032490	0.4368231046931	0.52
**Conduct Problems**	MFCC+MELS+PROS	4	0.6002066656202	0.5688405797101	0.5667870036101	0.5724637681159	0.5709090909090
**Conduct Problems**	MFCC+MELS+PROS	5	0.5732616543713	0.5709342560553	0.5978260869565	0.5054151624548	0.5463576158940
**Conduct Problems**	MFCC+MELS+PROS	Mean	0.5720 ± 0.0170	0.5641 ± 0.0055	0.5780 ± 0.0263	0.5289 ± 0.0593	0.5526 ± 0.0206
**Conduct Problems**	MFCC+MELS+FORM	1	0.5424676458705	0.5270758122743	0.5270758122743	0.5270758122743	0.5270758122743
**Conduct Problems**	MFCC+MELS+FORM	2	0.5655097811779	0.5464480874316	0.5415162454873	0.5595667870036	0.5514705882352
**Conduct Problems**	MFCC+MELS+FORM	3	0.5661353595120	0.5787671232876	0.6101083032490	0.5018050541516	0.5504885993485
**Conduct Problems**	MFCC+MELS+FORM	4	0.6022602417202	0.5735027223230	0.5703971119133	0.5797101449275	0.5766423357664
**Conduct Problems**	MFCC+MELS+FORM	5	0.5938366556793	0.5884353741496	0.6268115942028	0.4981949458483	0.5544871794871
**Conduct Problems**	MFCC+MELS+FORM	Mean	0.5740 ± 0.0215	0.5628 ± 0.0227	0.5752 ± 0.0384	0.5333 ± 0.0320	0.5520 ± 0.0157
**Conduct Problems**	MFCC+PROS+FORM	1	0.5516167290072	0.5486725663716	0.5595667870036	0.5198555956678	0.5381944444444
**Conduct Problems**	MFCC+PROS+FORM	2	0.5863884580797	0.5621621621621	0.5631768953068	0.5595667870036	0.5611510791366
**Conduct Problems**	MFCC+PROS+FORM	3	0.5558784814085	0.5494880546075	0.5812274368231	0.4657039711191	0.5210355987055
**Conduct Problems**	MFCC+PROS+FORM	4	0.6415921100821	0.6131907308377	0.6209386281588	0.5942028985507	0.6056338028169
**Conduct Problems**	MFCC+PROS+FORM	5	0.6293491340972	0.6166950596252	0.6557971014492	0.5306859205776	0.5819935691318
**Conduct Problems**	MFCC+PROS+FORM	Mean	0.5930 ± 0.0369	0.5780 ± 0.0305	0.5961 ± 0.0369	0.5340 ± 0.0428	0.5616 ± 0.0302
**Conduct Problems**	MELS+PROS+FORM	1	0.5523987019249	0.5505226480836	0.5703971119133	0.4981949458483	0.5319865319865
**Conduct Problems**	MELS+PROS+FORM	2	0.5593843266561	0.5330948121645	0.5379061371841	0.5198555956678	0.5283687943262
**Conduct Problems**	MELS+PROS+FORM	3	0.5597883459969	0.5406360424028	0.5523465703971	0.5090252707581	0.5294117647058

**Conduct Problems**	MELS+PROS+FORM	4	0.5974467639826	0.5787545787545	0.5703971119133	0.5978260869565	0.5873605947955
**Conduct Problems**	MELS+PROS+FORM	5	0.5814105582587	0.5709219858156	0.5833333333333	0.5415162454873	0.5590277777777
**Conduct Problems**	MELS+PROS+FORM	Mean	0.5701 ± 0.0168	0.5548 ± 0.0175	0.5629 ± 0.0159	0.5333 ± 0.0353	0.5472 ± 0.0230
**Conduct Problems**	MFCC+MELS+PROS+	1	0.5582244001616	0.5451327433628	0.5559566787003	0.5162454873646	0.5347222222222
**Conduct Problems**	MFCC+MELS+PROS+	2	0.5791682414732	0.5464480874316	0.5415162454873	0.5595667870036	0.5514705882352
**Conduct Problems**	MFCC+MELS+PROS+	3	0.5617302454091	0.5625	0.5848375451263	0.5054151624548	0.5418060200668
**Conduct Problems**	MFCC+MELS+PROS+	4	0.6175508815989	0.5843920145190	0.5812274368231	0.5905797101449	0.5875912408759
**Conduct Problems**	MFCC+MELS+PROS+	5	0.6031889290012	0.6125211505922	0.6557971014492	0.5162454873646	0.5746031746031
**Conduct Problems**	MFCC+MELS+PROS+	Mean	0.5840 ± 0.0231	0.5702 ± 0.0255	0.5839 ± 0.0394	0.5376 ± 0.0324	0.5580 ± 0.0200
**Peer Problems**	MFCC	1	0.5021787200832	0.5304518664047	0.5443548387096	0.4919354838709	0.5172413793103
**Peer Problems**	MFCC	2	0.6034566857440	0.5611222444889	0.5645161290322	0.5524193548387	0.5577689243027
**Peer Problems**	MFCC	3	0.4925370707596	0.476	0.4798387096774	0.4637096774193	0.4722222222222
**Peer Problems**	MFCC	4	0.5466729789734	0.5575048732943	0.5766129032258	0.5060728744939	0.5396226415094
**Peer Problems**	MFCC	5	0.5091093117408	0.4989775051124	0.4939271255060	0.5161290322580	0.5041322314049
**Peer Problems**	MFCC	Mean	0.5308 ± 0.0407	0.5248 ± 0.0331	0.5318 ± 0.0384	0.5061 ± 0.0291	0.5182 ± 0.0295
**Peer Problems**	MELS	1	0.5385665972944	0.5271966527196	0.5080645161290	0.5806451612903	0.5478260869565
**Peer Problems**	MELS	2	0.5649551248699	0.5518672199170	0.5362903225806	0.5927419354838	0.5683760683760
**Peer Problems**	MELS	3	0.5302256763787	0.5241935483870	0.5241935483870	0.5241935483870	0.5241935483870
**Peer Problems**	MELS	4	0.5389023116102	0.5365853658536	0.5322580645161	0.5465587044534	0.5409836065573
**Peer Problems**	MELS	5	0.5374820425754	0.5335968379446	0.5465587044534	0.5	0.5212355212355
**Peer Problems**	MELS	Mean	0.5420 ± 0.0119	0.5347 ± 0.0097	0.5295 ± 0.0129	0.5488 ± 0.0345	0.5405 ± 0.0171
**Peer Problems**	PROS	1	0.4828466441207	0.4788732394366	0.4798387096774	0.4758064516129	0.4779116465863
**Peer Problems**	PROS	2	0.5411517950052	0.5892857142857	0.6653225806451	0.4072580645161	0.5288461538461
**Peer Problems**	PROS	3	0.5172021331945	0.5454545454545	0.5927419354838	0.4193548387096	0.5051546391752
**Peer Problems**	PROS	4	0.5257770667363	0.5358490566037	0.5725806451612	0.4331983805668	0.5035460992907
**Peer Problems**	PROS	5	0.4897152931957	0.5087378640776	0.5303643724696	0.4475806451612	0.4888059701492
**Peer Problems**	PROS	Mean	0.5113 ± 0.0220	0.5316 ± 0.0370	0.5682 ± 0.0621	0.4366 ± 0.0238	0.5009 ± 0.0172
**Peer Problems**	FORM	1	0.5637844693028	0.5458089668615	0.5645161290322	0.4959677419354	0.5283018867924
**Peer Problems**	FORM	2	0.5644023152965	0.5573122529644	0.5685483870967	0.5282258064516	0.5465116279069
**Peer Problems**	FORM	3	0.5465660770031	0.5302713987473	0.5120967741935	0.5806451612903	0.5497835497835
**Peer Problems**	FORM	4	0.4695540028731	0.4900398406374	0.4959677419354	0.4696356275303	0.4842519685039
**Peer Problems**	FORM	5	0.4965391145357	0.4980079681274	0.5060728744939	0.4758064516129	0.4901960784313
**Peer Problems**	FORM	Mean	0.5282 ± 0.0384	0.5243 ± 0.0263	0.5294 ± 0.0307	0.5101 ± 0.0408	0.5198 ± 0.0277
**Peer Problems**	MFCC+MELS	1	0.5194784079084	0.5041666666666	0.4879032258064	0.5524193548387	0.5215517241379
**Peer Problems**	MFCC+MELS	2	0.5797183922996	0.5524625267665	0.5201612903225	0.6370967741935	0.5890410958904
**Peer Problems**	MFCC+MELS	3	0.5050403225806	0.5121951219512	0.5080645161290	0.5241935483870	0.5163934426229
**Peer Problems**	MFCC+MELS	4	0.5431631187149	0.5154639175257	0.5040322580645	0.5465587044534	0.5274261603375
**Peer Problems**	MFCC+MELS	5	0.5367637455922	0.5443786982248	0.5587044534412	0.5080645161290	0.5307692307692
**Peer Problems**	MFCC+MELS	Mean	0.5368 ± 0.0253	0.5257 ± 0.0191	0.5158 ± 0.0238	0.5537 ± 0.0446	0.5370 ± 0.0265
**Peer Problems**	MFCC+PROS	1	0.4783916493236	0.5069306930693	0.5161290322580	0.4798387096774	0.4980544747081
**Peer Problems**	MFCC+PROS	2	0.5813280437044	0.5702970297029	0.5806451612903	0.5443548387096	0.5603112840466
**Peer Problems**	MFCC+PROS	3	0.4790257544224	0.4729458917835	0.4758064516129	0.4637096774193	0.4701195219123
**Peer Problems**	MFCC+PROS	4	0.5325682382134	0.5475728155339	0.5685483870967	0.4898785425101	0.5280898876404
**Peer Problems**	MFCC+PROS	5	0.4948576465978	0.4804928131416	0.4736842105263	0.5040322580645	0.4875
**Peer Problems**	MFCC+PROS	Mean	0.5132 ± 0.0393	0.5156 ± 0.0378	0.5230 ± 0.0450	0.4964 ± 0.0274	0.5088 ± 0.0319
**Peer Problems**	MFCC+FORM	1	0.5261121227887	0.528	0.5322580645161	0.5161290322580	0.5238095238095
**Peer Problems**	MFCC+FORM	2	0.6013917793964	0.572	0.5766129032258	0.5604838709677	0.5674603174603
**Peer Problems**	MFCC+FORM	3	0.5055931321540	0.4847250509164	0.4798387096774	0.5	0.4897119341563
**Peer Problems**	MFCC+FORM	4	0.4920007835967	0.5128205128205	0.5241935483870	0.4777327935222	0.5019305019305
**Peer Problems**	MFCC+FORM	5	0.5045220060075	0.4837398373983	0.4817813765182	0.4919354838709	0.4857142857142
**Peer Problems**	MFCC+FORM	Mean	0.5259 ± 0.0393	0.5163 ± 0.0326	0.5189 ± 0.0359	0.5093 ± 0.0285	0.5137 ± 0.0300
**Peer Problems**	MELS+PROS	1	0.5337701612903	0.5063291139240	0.4838709677419	0.5725806451612	0.5309734513274
**Peer Problems**	MELS+PROS	2	0.5654103798126	0.5308641975308	0.5201612903225	0.5604838709677	0.5420168067226
**Peer Problems**	MELS+PROS	3	0.5270063735691	0.4960317460317	0.5040322580645	0.4717741935483	0.48828125
**Peer Problems**	MELS+PROS	4	0.5447466370641	0.5298804780876	0.5362903225806	0.5101214574898	0.5236220472440
**Peer Problems**	MELS+PROS	5	0.5390982107875	0.5570599613152	0.5829959514170	0.4919354838709	0.5333333333333
**Peer Problems**	MELS+PROS	Mean	0.5420 ± 0.0131	0.5240 ± 0.0213	0.5255 ± 0.0336	0.5214 ± 0.0390	0.5236 ± 0.0186

**Peer Problems**	MELS+FORM	1	0.5272502601456	0.5201612903225	0.5201612903225	0.5201612903225	0.5201612903225
**Peer Problems**	MELS+FORM	2	0.5693776014568	0.5626283367556	0.5524193548387	0.5887096774193	0.5732217573221
**Peer Problems**	MELS+FORM	3	0.5285347294484	0.5186721991701	0.5040322580645	0.5604838709677	0.5341880341880
**Peer Problems**	MELS+FORM	4	0.5084726394149	0.4969450101832	0.4919354838709	0.5101214574898	0.5020576131687
**Peer Problems**	MELS+FORM	5	0.5441915893953	0.5327868852459	0.5263157894736	0.5524193548387	0.5394190871369
**Peer Problems**	MELS+FORM	Mean	0.5356 ± 0.0203	0.5262 ± 0.0215	0.5190 ± 0.0206	0.5464 ± 0.0284	0.5338 ± 0.0236
**Peer Problems**	PROS+FORM	1	0.5199499219562	0.5150905432595	0.5161290322580	0.5120967741935	0.5140562248995
**Peer Problems**	PROS+FORM	2	0.5470863683662	0.5426356589147	0.5645161290322	0.4838709677419	0.5223880597014
**Peer Problems**	PROS+FORM	3	0.5427289281997	0.5300207039337	0.5161290322580	0.5685483870967	0.5446808510638
**Peer Problems**	PROS+FORM	4	0.4719864176570	0.4790419161676	0.4838709677419	0.4615384615384	0.4743083003952
**Peer Problems**	PROS+FORM	5	0.5048974794305	0.4959016393442	0.4898785425101	0.5161290322580	0.5020746887966
**Peer Problems**	PROS+FORM	Mean	0.5173 ± 0.0274	0.5125 ± 0.0229	0.5141 ± 0.0285	0.5084 ± 0.0360	0.5115 ± 0.0232
**Peer Problems**	MFCC+MELS+PROS	1	0.5105033818938	0.4810126582278	0.4596774193548	0.5483870967741	0.5044247787610
**Peer Problems**	MFCC+MELS+PROS	2	0.5909696930280	0.5415778251599	0.5120967741935	0.6209677419354	0.5746606334841
**Peer Problems**	MFCC+MELS+PROS	3	0.5242260665972	0.5	0.4919354838709	0.5241935483870	0.5083333333333
**Peer Problems**	MFCC+MELS+PROS	4	0.5425264463889	0.536	0.5403225806451	0.5222672064777	0.5317460317460
**Peer Problems**	MFCC+MELS+PROS	5	0.5515867833355	0.5392354124748	0.5425101214574	0.5322580645161	0.536
**Peer Problems**	MFCC+MELS+PROS	Mean	0.5440 ± 0.0275	0.5196 ± 0.0245	0.5093 ± 0.0311	0.5496 ± 0.0368	0.5310 ± 0.0251
**Peer Problems**	MFCC+MELS+FORM	1	0.5102757544224	0.5060728744939	0.5040322580645	0.5120967741935	0.5081300813008
**Peer Problems**	MFCC+MELS+FORM	2	0.5852790062434	0.5454545454545	0.5322580645161	0.5806451612903	0.5593220338983
**Peer Problems**	MFCC+MELS+FORM	3	0.5089262486992	0.4745762711864	0.4516129032258	0.5483870967741	0.5
**Peer Problems**	MFCC+MELS+FORM	4	0.5048158547734	0.4969450101832	0.4919354838709	0.5101214574898	0.5020576131687
**Peer Problems**	MFCC+MELS+FORM	5	0.5425917461146	0.56	0.5668016194331	0.5443548387096	0.5533596837944
**Peer Problems**	MFCC+MELS+FORM	Mean	0.5304 ± 0.0306	0.5166 ± 0.0316	0.5093 ± 0.0387	0.5391 ± 0.0261	0.5246 ± 0.0261
**Peer Problems**	MFCC+PROS+FORM	1	0.5086661030176	0.5111561866125	0.5080645161290	0.5201612903225	0.5142857142857
**Peer Problems**	MFCC+PROS+FORM	2	0.5887747138397	0.5588822355289	0.5645161290322	0.5443548387096	0.5533596837944
**Peer Problems**	MFCC+PROS+FORM	3	0.5062272372528	0.5010266940451	0.4919354838709	0.5282258064516	0.5104602510460
**Peer Problems**	MFCC+PROS+FORM	4	0.4942536241347	0.5119047619047	0.5201612903225	0.4858299595141	0.50390625
**Peer Problems**	MFCC+PROS+FORM	5	0.5039996082016	0.4939271255060	0.4939271255060	0.4959677419354	0.4939271255060
**Peer Problems**	MFCC+PROS+FORM	Mean	0.5204 ± 0.0345	0.5154 ± 0.0228	0.5157 ± 0.0265	0.5149 ± 0.0213	0.5152 ± 0.0203
**Peer Problems**	MELS+PROS+FORM	1	0.5154624089490	0.4917355371900	0.4798387096774	0.5282258064516	0.5042372881355
**Peer Problems**	MELS+PROS+FORM	2	0.5713774713839	0.5562372188139	0.5483870967741	0.5766129032258	0.5643153526970
**Peer Problems**	MELS+PROS+FORM	3	0.5254780176899	0.5030674846625	0.4959677419354	0.5241935483870	0.5103734439834
**Peer Problems**	MELS+PROS+FORM	4	0.5051586783335	0.5140562248995	0.5161290322580	0.5060728744939	0.512
**Peer Problems**	MELS+PROS+FORM	5	0.5458893822645	0.5269709543568	0.5141700404858	0.5645161290322	0.5404255319148
**Peer Problems**	MELS+PROS+FORM	Mean	0.5327 ± 0.0236	0.5184 ± 0.0222	0.5109 ± 0.0229	0.5399 ± 0.0264	0.5263 ± 0.0228
**Peer Problems**	MFCC+MELS+PROS+	1	0.5049265088449	0.4836065573770	0.4758064516129	0.5080645161290	0.4916666666666
**Peer Problems**	MFCC+MELS+PROS+	2	0.5978798126951	0.5672877846790	0.5524193548387	0.6048387096774	0.5829787234042
**Peer Problems**	MFCC+MELS+PROS+	3	0.5197385535900	0.5	0.4798387096774	0.5604838709677	0.5219298245614
**Peer Problems**	MFCC+MELS+PROS+	4	0.5126681467937	0.4897959183673	0.4838709677419	0.5060728744939	0.4958677685950
**Peer Problems**	MFCC+MELS+PROS+	5	0.5386900875016	0.5362903225806	0.5384615384615	0.5322580645161	0.5341365461847
**Peer Problems**	MFCC+MELS+PROS+	Mean	0.5348 ± 0.0335	0.5154 ± 0.0317	0.5061 ± 0.0325	0.5423 ± 0.0370	0.5253 ± 0.0329

SVM_Male_detailed_performance_results

**Symptom**	Subspace	Fold	AUC	F1	Sensitivity	Specificity	Precision
**Emotional Symptoms**	MFCC	1	0.5685839623566	0.5755395683453	0.5940594059405	0.5297029702970	0.5581395348837
**Emotional Symptoms**	MFCC	2	0.5658146260170	0.5472392638036	0.5519801980198	0.5346534653465	0.5425790754257
**Emotional Symptoms**	MFCC	3	0.5960199980394	0.5909090909090	0.6113861386138	0.5420792079207	0.5717592592592
**Emotional Symptoms**	MFCC	4	0.5895388546298	0.5741857659831	0.5905707196029	0.5346534653465	0.5586854460093
**Emotional Symptoms**	MFCC	5	0.5681030882244	0.5693430656934	0.5792079207920	0.5434243176178	0.5598086124401
**Emotional Symptoms**	MFCC	Mean	0.5776 ± 0.0126	0.5714 ± 0.0141	0.5854 ± 0.0197	0.5369 ± 0.0051	0.5582 ± 0.0093
**Emotional Symptoms**	MELS	1	0.5768123223213	0.5563909774436	0.5495049504950	0.5742574257425	0.5634517766497
**Emotional Symptoms**	MELS	2	0.5692824232918	0.5814771395076	0.6138613861386	0.5024752475247	0.5523385300668
**Emotional Symptoms**	MELS	3	0.5861312616410	0.6021765417170	0.6163366336633	0.5693069306930	0.5886524822695
**Emotional Symptoms**	MELS	4	0.5913569024396	0.5735294117647	0.5806451612903	0.5569306930693	0.5665859564164
**Emotional Symptoms**	MELS	5	0.5900486450630	0.5493670886075	0.5371287128712	0.5806451612903	0.5621761658031
**Emotional Symptoms**	MELS	Mean	0.5827 ± 0.0084	0.5726 ± 0.0187	0.5795 ± 0.0324	0.5567 ± 0.0282	0.5666 ± 0.0120
**Emotional Symptoms**	PROS	1	0.5280977355161	0.5462857142857	0.5915841584158	0.4257425742574	0.5074309978768

**Emotional Symptoms**	PROS	2	0.564448338398	0.583621683967	0.626237623762	0.480198019801	0.546436285097
**Emotional Symptoms**	PROS	3	0.582142682089	0.582750582750	0.618811881188	0.495049504950	0.550660792951
**Emotional Symptoms**	PROS	4	0.541305309190	0.547393364928	0.573200992555	0.480198019801	0.523809523809
**Emotional Symptoms**	PROS	5	0.567058939144	0.570414201183	0.596534653465	0.503722084367	0.546485260770
**Emotional Symptoms**	PROS	Mean	0.5566 ± 0.0193	0.5661 ± 0.0164	0.6013 ± 0.0192	0.4770 ± 0.0272	0.5350 ± 0.0167
**Emotional Symptoms**	FORM	1	0.552758308009	0.558083832335	0.576732673267	0.509900990099	0.540603248259
**Emotional Symptoms**	FORM	2	0.584434124105	0.584400465657	0.621287128712	0.495049504950	0.551648351648
**Emotional Symptoms**	FORM	3	0.559503970199	0.55	0.571782178217	0.492574257425	0.529816513761
**Emotional Symptoms**	FORM	4	0.626642999287	0.631698973774	0.687344913151	0.512376237623	0.584388185654
**Emotional Symptoms**	FORM	5	0.534463061690	0.565864833906	0.611386138613	0.449131513647	0.526652452025
**Emotional Symptoms**	FORM	Mean	0.5716 ± 0.0319	0.5780 ± 0.0292	0.6137 ± 0.0415	0.4918 ± 0.0227	0.5466 ± 0.0208
**Emotional Symptoms**	MFCC+MELS	1	0.595156112145	0.578754578754	0.586633663366	0.559405940594	0.571084337349
**Emotional Symptoms**	MFCC+MELS	2	0.616698117831	0.608796296296	0.650990099009	0.512376237623	0.571739130434
**Emotional Symptoms**	MFCC+MELS	3	0.639097882560	0.605103280680	0.616336633663	0.579207920792	0.594272076372
**Emotional Symptoms**	MFCC+MELS	4	0.625740117436	0.610086100861	0.615384615384	0.599009900990	0.604878048780
**Emotional Symptoms**	MFCC+MELS	5	0.639307913421	0.591760299625	0.586633663366	0.602977667493	0.596977329974
**Emotional Symptoms**	MFCC+MELS	Mean	0.6232 ± 0.0164	0.5989 ± 0.0120	0.6112 ± 0.0238	0.5706 ± 0.0330	0.5878 ± 0.0138
**Emotional Symptoms**	MFCC+PROS	1	0.569453975100	0.557457212713	0.564356435643	0.539603960396	0.550724637681
**Emotional Symptoms**	MFCC+PROS	2	0.580996961082	0.562801932367	0.576732673267	0.527227722772	0.549528301886
**Emotional Symptoms**	MFCC+PROS	3	0.603825605332	0.589090909090	0.601485148514	0.559405940594	0.577197149643
**Emotional Symptoms**	MFCC+PROS	4	0.585184138761	0.561743341404	0.575682382133	0.527227722772	0.548463356973
**Emotional Symptoms**	MFCC+PROS	5	0.596903176670	0.590361445783	0.606435643564	0.550868486352	0.575117370892
**Emotional Symptoms**	MFCC+PROS	Mean	0.5873 ± 0.0121	0.5723 ± 0.0144	0.5849 ± 0.0162	0.5409 ± 0.0128	0.5602 ± 0.0131
**Emotional Symptoms**	MFCC+FORM	1	0.598813841780	0.583232077764	0.594059405940	0.556930693069	0.572792362768
**Emotional Symptoms**	MFCC+FORM	2	0.586204783844	0.580097087378	0.591584158415	0.551980198019	0.569047619047
**Emotional Symptoms**	MFCC+FORM	3	0.606680717576	0.586002372479	0.611386138613	0.524752475247	0.562642369020
**Emotional Symptoms**	MFCC+FORM	4	0.645775495663	0.639053254437	0.669975186104	0.574257425742	0.610859728506
**Emotional Symptoms**	MFCC+FORM	5	0.604138515588	0.628571428571	0.653465346534	0.573200992555	0.605504587155
**Emotional Symptoms**	MFCC+FORM	Mean	0.6083 ± 0.0200	0.6034 ± 0.0251	0.6241 ± 0.0319	0.5562 ± 0.0180	0.5842 ± 0.0199
**Emotional Symptoms**	MELS+PROS	1	0.612047838447	0.595898673100	0.611386138613	0.559405940594	0.581176470588
**Emotional Symptoms**	MELS+PROS	2	0.606974806391	0.586124401913	0.606435643564	0.537128712871	0.567129629629
**Emotional Symptoms**	MELS+PROS	3	0.628333006567	0.588942307692	0.606435643564	0.547029702970	0.572429906542
**Emotional Symptoms**	MELS+PROS	4	0.594305088077	0.582309582309	0.588089330024	0.569306930693	0.576642335766
**Emotional Symptoms**	MELS+PROS	5	0.632158563250	0.584070796460	0.571782178217	0.612903225806	0.596899224806
**Emotional Symptoms**	MELS+PROS	Mean	0.6148 ± 0.0140	0.5875 ± 0.0048	0.5968 ± 0.0148	0.5652 ± 0.0262	0.5789 ± 0.0101
**Emotional Symptoms**	MELS+FORM	1	0.596559160866	0.589242053789	0.596534653465	0.571782178217	0.582125603864
**Emotional Symptoms**	MELS+FORM	2	0.624901970395	0.618436406067	0.655940594059	0.534653465346	0.584988962472
**Emotional Symptoms**	MELS+FORM	3	0.614725272032	0.592411260709	0.599009900990	0.576732673267	0.585956416464
**Emotional Symptoms**	MELS+FORM	4	0.623891359359	0.596363636363	0.610421836228	0.564356435643	0.582938388625
**Emotional Symptoms**	MELS+FORM	5	0.615317052797	0.578491965389	0.579207920792	0.575682382133	0.577777777777
**Emotional Symptoms**	MELS+FORM	Mean	0.6151 ± 0.0102	0.5950 ± 0.0131	0.6082 ± 0.0259	0.5646 ± 0.0156	0.5828 ± 0.0028
**Emotional Symptoms**	PROS+FORM	1	0.566580482305	0.551390568319	0.564356435643	0.517326732673	0.539007092198
**Emotional Symptoms**	PROS+FORM	2	0.602624742672	0.588796185935	0.611386138613	0.534653465346	0.567816091954
**Emotional Symptoms**	PROS+FORM	3	0.583239388295	0.589371980676	0.603960396039	0.554455445544	0.575471698113
**Emotional Symptoms**	PROS+FORM	4	0.614776552096	0.608695652173	0.642679900744	0.532178217821	0.578125
**Emotional Symptoms**	PROS+FORM	5	0.543848119303	0.543735224586	0.569306930693	0.473945409429	0.520361990950
**Emotional Symptoms**	PROS+FORM	Mean	0.5822 ± 0.0253	0.5764 ± 0.0247	0.5983 ± 0.0289	0.5225 ± 0.0270	0.5562 ± 0.0227
**Emotional Symptoms**	MFCC+MELS+PROS	1	0.607360797960	0.584951456310	0.596534653465	0.556930693069	0.573809523809
**Emotional Symptoms**	MFCC+MELS+PROS	2	0.624944858347	0.626025791324	0.660891089108	0.549504950495	0.594654788418
**Emotional Symptoms**	MFCC+MELS+PROS	3	0.633675620037	0.609223300970	0.621287128712	0.581683168316	0.597619047619
**Emotional Symptoms**	MFCC+MELS+PROS	4	0.635751664496	0.612590799031	0.627791563275	0.579207920792	0.598108747044
**Emotional Symptoms**	MFCC+MELS+PROS	5	0.648306021669	0.618932038834	0.631188118811	0.590570719602	0.607142857142
**Emotional Symptoms**	MFCC+MELS+PROS	Mean	0.6300 ± 0.0136	0.6103 ± 0.0139	0.6275 ± 0.0206	0.5716 ± 0.0156	0.5943 ± 0.0110
**Emotional Symptoms**	MFCC+MELS+FORM	1	0.622739192236	0.585908529048	0.586633663366	0.584158415841	0.585185185185
**Emotional Symptoms**	MFCC+MELS+FORM	2	0.618572934026	0.603592814371	0.623762376237	0.556930693069	0.584686774941
**Emotional Symptoms**	MFCC+MELS+FORM	3	0.640445789628	0.622596153846	0.641089108910	0.581683168316	0.605140186915
**Emotional Symptoms**	MFCC+MELS+FORM	4	0.651573594083	0.620359281437	0.642679900744	0.571782178217	0.599537037037

**Emotional Symptoms**	MFCC+MELS+FORM	5	0.6457570695034	0.6248462484624	0.6287128712871	0.6153846153846	0.6210268948655
**Emotional Symptoms**	MFCC+MELS+FORM	Mean	0.6358 ± 0.0129	0.6115 ± 0.0148	0.6246 ± 0.0203	0.5820 ± 0.0192	0.5991 ± 0.0136
**Emotional Symptoms**	MFCC+PROS+FORM	1	0.6024409371630	0.5916870415647	0.5990099009900	0.5742574257425	0.5845410628019
**Emotional Symptoms**	MFCC+PROS+FORM	2	0.5894275071071	0.5686746987951	0.5841584158415	0.5297029702970	0.5539906103286
**Emotional Symptoms**	MFCC+PROS+FORM	3	0.6074894618174	0.5966183574879	0.6113861386138	0.5618811881188	0.5825471698113
**Emotional Symptoms**	MFCC+PROS+FORM	4	0.6435520723288	0.6460807600950	0.6749379652605	0.5866336633663	0.6195899772209
**Emotional Symptoms**	MFCC+PROS+FORM	5	0.5920509544751	0.5920577617328	0.6089108910891	0.5508684863523	0.5761124121779
**Emotional Symptoms**	MFCC+PROS+FORM	Mean	0.6070 ± 0.0194	0.5990 ± 0.0255	0.6157 ± 0.0311	0.5607 ± 0.0196	0.5834 ± 0.0211
**Emotional Symptoms**	MELS+PROS+FORM	1	0.6145843544750	0.5958485958485	0.6039603960396	0.5767326732673	0.5879518072289
**Emotional Symptoms**	MELS+PROS+FORM	2	0.6419652485050	0.6150234741784	0.6485148514851	0.5396039603960	0.5848214285714
**Emotional Symptoms**	MELS+PROS+FORM	3	0.6169309381433	0.6007237635705	0.6163366336633	0.5643564356435	0.5858823529411
**Emotional Symptoms**	MELS+PROS+FORM	4	0.6230928924157	0.6109785202863	0.6352357320099	0.5569306930693	0.5885057471264
**Emotional Symptoms**	MELS+PROS+FORM	5	0.6253040316438	0.6066584463625	0.6089108910891	0.6004962779156	0.6044226044226
**Emotional Symptoms**	MELS+PROS+FORM	Mean	0.6244 ± 0.0096	0.6058 ± 0.0069	0.6226 ± 0.0168	0.5676 ± 0.0204	0.5903 ± 0.0072
**Emotional Symptoms**	MFCC+MELS+PROS+	1	0.6255452896774	0.5928659286592	0.5965346534653	0.5841584158415	0.5892420537897
**Emotional Symptoms**	MFCC+MELS+PROS+	2	0.6217466424860	0.6263096623981	0.6658415841584	0.5396039603960	0.5912087912087
**Emotional Symptoms**	MFCC+MELS+PROS+	3	0.6326524360356	0.6040914560770	0.6212871287128	0.5643564356435	0.5878220140515
**Emotional Symptoms**	MFCC+MELS+PROS+	4	0.6474829865120	0.6115107913669	0.6327543424317	0.5643564356435	0.5916473317865
**Emotional Symptoms**	MFCC+MELS+PROS+	5	0.6378768149767	0.6200980392156	0.6262376237623	0.6054590570719	0.6140776699029
**Emotional Symptoms**	MFCC+MELS+PROS+	Mean	0.6331 ± 0.0091	0.6110 ± 0.0118	0.6285 ± 0.0223	0.5716 ± 0.0221	0.5948 ± 0.0097
**Hyperactivity**	MFCC	1	0.6127977750219	0.5590643274853	0.5444191343963	0.5968109339407	0.5745192307692
**Hyperactivity**	MFCC	2	0.6120765251321	0.5834254143646	0.6013667425968	0.5398633257403	0.5665236051502
**Hyperactivity**	MFCC	3	0.6396863860191	0.6086956521739	0.6218678815489	0.5785876993166	0.5960698689956
**Hyperactivity**	MFCC	4	0.5945954379505	0.6094420600858	0.6484018264840	0.5216400911161	0.5748987854251
**Hyperactivity**	MFCC	5	0.5962440582061	0.5604519774011	0.5649202733485	0.5479452054794	0.5560538116591
**Hyperactivity**	MFCC	Mean	0.6111 ± 0.0162	0.5842 ± 0.0221	0.5962 ± 0.0376	0.5570 ± 0.0271	0.5736 ± 0.0132
**Hyperactivity**	MELS	1	0.6823283399318	0.6312217194570	0.6355353075170	0.6218678815489	0.6269662921348
**Hyperactivity**	MELS	2	0.6327281406800	0.6083150984682	0.6332574031890	0.5512528473804	0.5852631578947
**Hyperactivity**	MELS	3	0.6381504869733	0.6114790286975	0.6309794988610	0.5671981776765	0.5931477516059
**Hyperactivity**	MELS	4	0.6402003307641	0.6105499438832	0.6210045662100	0.5876993166287	0.6004415011037
**Hyperactivity**	MELS	5	0.6344067567427	0.5995525727069	0.6104783599088	0.5730593607305	0.5890109890109
**Hyperactivity**	MELS	Mean	0.6456 ± 0.0186	0.6122 ± 0.0104	0.6263 ± 0.0093	0.5802 ± 0.0239	0.5990 ± 0.0149
**Hyperactivity**	PROS	1	0.6090462378256	0.5773672055427	0.5694760820045	0.5968109339407	0.5854800936768
**Hyperactivity**	PROS	2	0.6201555616668	0.6188436830835	0.6583143507972	0.5307517084282	0.5838383838383
**Hyperactivity**	PROS	3	0.5905479942507	0.6006389776357	0.6423690205011	0.5034168564920	0.564
**Hyperactivity**	PROS	4	0.5567395804079	0.5972660357518	0.6484018264840	0.4783599088838	0.5536062378167
**Hyperactivity**	PROS	5	0.5849221455986	0.5993555316863	0.6355353075170	0.5136986301369	0.5670731707317
**Hyperactivity**	PROS	Mean	0.5923 ± 0.0218	0.5987 ± 0.0132	0.6308 ± 0.0316	0.5246 ± 0.0399	0.5708 ± 0.0122
**Hyperactivity**	FORM	1	0.6075051499317	0.5923497267759	0.6173120728929	0.5330296127562	0.5693277310924
**Hyperactivity**	FORM	2	0.5995350792077	0.6051502145922	0.6423690205011	0.5193621867881	0.5720081135902
**Hyperactivity**	FORM	3	0.6404854686308	0.6297872340425	0.6742596810933	0.5330296127562	0.5908183632734
**Hyperactivity**	FORM	4	0.5853850074369	0.6057385759829	0.6506849315068	0.5034168564920	0.5666003976143
**Hyperactivity**	FORM	5	0.6292632695728	0.6240681576144	0.6674259681093	0.5273972602739	0.586
**Hyperactivity**	FORM	Mean	0.6124 ± 0.0200	0.6114 ± 0.0137	0.6504 ± 0.0201	0.5232 ± 0.0111	0.5770 ± 0.0096
**Hyperactivity**	MFCC+MELS	1	0.6900182128569	0.6442199775533	0.6537585421412	0.6241457858769	0.6349557522123
**Hyperactivity**	MFCC+MELS	2	0.6944391114616	0.6570477247502	0.6742596810933	0.6218678815489	0.6406926406926
**Hyperactivity**	MFCC+MELS	3	0.6801905345032	0.6492942453854	0.6810933940774	0.5831435079726	0.6203319502074
**Hyperactivity**	MFCC+MELS	4	0.6397062647569	0.6121546961325	0.6324200913242	0.5671981776765	0.5931477516059
**Hyperactivity**	MFCC+MELS	5	0.6749409721138	0.6383928571428	0.6514806378132	0.6095890410958	0.6258205689277
**Hyperactivity**	MFCC+MELS	Mean	0.6759 ± 0.0194	0.6402 ± 0.0153	0.6586 ± 0.0174	0.6012 ± 0.0224	0.6230 ± 0.0165
**Hyperactivity**	MFCC+PROS	1	0.6531255026696	0.6134549600912	0.6127562642369	0.6150341685649	0.6141552511415
**Hyperactivity**	MFCC+PROS	2	0.6594714639297	0.6099773242630	0.6127562642369	0.6036446469248	0.6072234762979
**Hyperactivity**	MFCC+PROS	3	0.6497164294498	0.5979843225083	0.6082004555808	0.5740318906605	0.5881057268722
**Hyperactivity**	MFCC+PROS	4	0.6203596800532	0.6108695652173	0.6415525114155	0.5421412300683	0.5829875518672
**Hyperactivity**	MFCC+PROS	5	0.6373711527860	0.6225165562913	0.6423690205011	0.5776255707762	0.6038543897216
**Hyperactivity**	MFCC+PROS	Mean	0.6440 ± 0.0138	0.6110 ± 0.0079	0.6235 ± 0.0151	0.5825 ± 0.0254	0.5993 ± 0.0118
**Hyperactivity**	MFCC+FORM	1	0.6740625048645	0.6287703016241	0.6173120728929	0.6537585421412	0.6406619385342

**Hyperactivity**	MFCC+FORM	2	0.6567473186627	0.6170921198668	0.6332574031890	0.5808656036446	0.6017316017316
**Hyperactivity**	MFCC+FORM	3	0.6910923044193	0.6585365853658	0.6765375854214	0.6218678815489	0.6414686825054
**Hyperactivity**	MFCC+FORM	4	0.6519226968723	0.6199342825848	0.6461187214611	0.5626423690205	0.5957894736842
**Hyperactivity**	MFCC+FORM	5	0.6486046535817	0.6244541484716	0.6514806378132	0.5639269406392	0.5995807127882
**Hyperactivity**	MFCC+FORM	Mean	0.6645 ± 0.0159	0.6298 ± 0.0149	0.6449 ± 0.0197	0.5966 ± 0.0357	0.6158 ± 0.0207
**Hyperactivity**	MELS+PROS	1	0.6934220972280	0.6453819840364	0.6446469248291	0.6469248291571	0.6461187214611
**Hyperactivity**	MELS+PROS	2	0.6456328059734	0.6233480176211	0.6446469248291	0.5763097949886	0.6034115138592
**Hyperactivity**	MELS+PROS	3	0.6542047830802	0.6281352235550	0.6560364464692	0.5671981776765	0.6025104602510
**Hyperactivity**	MELS+PROS	4	0.6509449662474	0.6301969365426	0.6575342465753	0.5717539863325	0.6050420168067
**Hyperactivity**	MELS+PROS	5	0.6692046057353	0.6361556064073	0.6332574031890	0.6415525114155	0.6390804597701
**Hyperactivity**	MELS+PROS	Mean	0.6627 ± 0.0172	0.6326 ± 0.0076	0.6472 ± 0.0089	0.6007 ± 0.0357	0.6192 ± 0.0192
**Hyperactivity**	MELS+FORM	1	0.6995968265004	0.6629588431590	0.6788154897494	0.6309794988610	0.6478260869565
**Hyperactivity**	MELS+FORM	2	0.6541269503582	0.6400862068965	0.6765375854214	0.5626423690205	0.6073619631901
**Hyperactivity**	MELS+FORM	3	0.7074371760212	0.6753246753246	0.7107061503416	0.6059225512528	0.6432989690721
**Hyperactivity**	MELS+FORM	4	0.6654757075545	0.6438053097345	0.6643835616438	0.6013667425968	0.6244635193133
**Hyperactivity**	MELS+FORM	5	0.6874434424439	0.6405375139977	0.6514806378132	0.6164383561643	0.6299559471365
**Hyperactivity**	MELS+FORM	Mean	0.6828 ± 0.0202	0.6525 ± 0.0142	0.6764 ± 0.0197	0.6035 ± 0.0228	0.6306 ± 0.0144
**Hyperactivity**	PROS+FORM	1	0.6376990571862	0.6219239373601	0.6332574031890	0.5968109339407	0.6109890109890
**Hyperactivity**	PROS+FORM	2	0.6189465600531	0.5884955752212	0.6059225512528	0.5466970387243	0.5720430107526
**Hyperactivity**	PROS+FORM	3	0.6488447029643	0.6391534391534	0.6879271070615	0.5353075170842	0.5968379446640
**Hyperactivity**	PROS+FORM	4	0.6246918588323	0.6145494028230	0.6461187214611	0.5444191343963	0.5859213250517
**Hyperactivity**	PROS+FORM	5	0.6306362530034	0.6226012793176	0.6651480637813	0.5273972602739	0.5851703406813
**Hyperactivity**	PROS+FORM	Mean	0.6322 ± 0.0104	0.6173 ± 0.0165	0.6477 ± 0.0279	0.5501 ± 0.0243	0.5902 ± 0.0130
**Hyperactivity**	MFCC+MELS+PROS	1	0.6988963320032	0.6508474576271	0.6560364464692	0.6400911161731	0.6457399103139
**Hyperactivity**	MFCC+MELS+PROS	2	0.6835996077230	0.6507072905331	0.6810933940774	0.5876993166287	0.6229166666666
**Hyperactivity**	MFCC+MELS+PROS	3	0.6908536174054	0.6548672566371	0.6742596810933	0.6150341685649	0.6365591397849
**Hyperactivity**	MFCC+MELS+PROS	4	0.6646695998585	0.6427795874049	0.6757990867579	0.5740318906605	0.6128364389233
**Hyperactivity**	MFCC+MELS+PROS	5	0.6881195327695	0.6425438596491	0.6674259681093	0.5890410958904	0.6194503171247
**Hyperactivity**	MFCC+MELS+PROS	Mean	0.6852 ± 0.0114	0.6483 ± 0.0049	0.6709 ± 0.0086	0.6012 ± 0.0235	0.6275 ± 0.0120
**Hyperactivity**	MFCC+MELS+FORM	1	0.7300553650095	0.6853932584269	0.6947608200455	0.6674259681093	0.6762749445676
**Hyperactivity**	MFCC+MELS+FORM	2	0.6966288053714	0.6637837837837	0.6993166287015	0.5922551252847	0.6316872427983
**Hyperactivity**	MFCC+MELS+FORM	3	0.7292562823978	0.6936542669584	0.7220956719817	0.6400911161731	0.6673684210526
**Hyperactivity**	MFCC+MELS+FORM	4	0.7015113219126	0.6745689655172	0.7146118721461	0.5968109339407	0.6387755102040
**Hyperactivity**	MFCC+MELS+FORM	5	0.7018389656858	0.6805708013172	0.7061503416856	0.6301369863013	0.6567796610169
**Hyperactivity**	MFCC+MELS+FORM	Mean	0.7119 ± 0.0147	0.6796 ± 0.0101	0.7074 ± 0.0099	0.6253 ± 0.0280	0.6542 ± 0.0168
**Hyperactivity**	MFCC+PROS+FORM	1	0.6771343029560	0.6431014823261	0.6423690205011	0.6446469248291	0.6438356164383
**Hyperactivity**	MFCC+PROS+FORM	2	0.6711619387612	0.6165919282511	0.6264236902050	0.5945330296127	0.6070640176600
**Hyperactivity**	MFCC+PROS+FORM	3	0.6891672417640	0.6473214285714	0.6605922551252	0.6195899772209	0.6345733041575
**Hyperactivity**	MFCC+PROS+FORM	4	0.6604778398394	0.6462882096069	0.6757990867579	0.5854214123006	0.6192468619246
**Hyperactivity**	MFCC+PROS+FORM	5	0.6509241634682	0.6407766990291	0.6765375854214	0.5639269406392	0.6086065573770
**Hyperactivity**	MFCC+PROS+FORM	Mean	0.6698 ± 0.0132	0.6388 ± 0.0114	0.6563 ± 0.0195	0.6016 ± 0.0280	0.6227 ± 0.0144
**Hyperactivity**	MELS+PROS+FORM	1	0.7099537673631	0.6711409395973	0.6833712984054	0.6469248291571	0.6593406593406
**Hyperactivity**	MELS+PROS+FORM	2	0.6602913019338	0.6368593238822	0.6651480637813	0.5763097949886	0.6108786610878
**Hyperactivity**	MELS+PROS+FORM	3	0.7166162483590	0.6769230769230	0.7015945330296	0.6287015945330	0.6539278131634
**Hyperactivity**	MELS+PROS+FORM	4	0.6826536025212	0.6483516483516	0.6735159817351	0.5968109339407	0.625
**Hyperactivity**	MELS+PROS+FORM	5	0.6987705557462	0.6312217194570	0.6355353075170	0.6210045662100	0.6269662921348
**Hyperactivity**	MELS+PROS+FORM	Mean	0.6937 ± 0.0203	0.6529 ± 0.0182	0.6718 ± 0.0218	0.6140 ± 0.0248	0.6352 ± 0.0184
**Hyperactivity**	MFCC+MELS+PROS+	1	0.7431416399873	0.7138121546961	0.7357630979498	0.6742596810933	0.6931330472103
**Hyperactivity**	MFCC+MELS+PROS+	2	0.6953990483652	0.6579234972677	0.6856492027334	0.6013667425968	0.6323529411764
**Hyperactivity**	MFCC+MELS+PROS+	3	0.7413826204720	0.7098901098901	0.7357630979498	0.6628701594533	0.6857749469214
**Hyperactivity**	MFCC+MELS+PROS+	4	0.7113406351088	0.6828729281767	0.7054794520547	0.6400911161731	0.6616702355460
**Hyperactivity**	MFCC+MELS+PROS+	5	0.7159536514078	0.6813186813186	0.7061503416856	0.6324200913242	0.6581740976645
**Hyperactivity**	MFCC+MELS+PROS+	Mean	0.7214 ± 0.0183	0.6892 ± 0.0206	0.7138 ± 0.0194	0.6422 ± 0.0254	0.6662 ± 0.0216
**Conduct Problems**	MFCC	1	0.5626136871112	0.5806451612903	0.5940594059405	0.5478547854785	0.5678233438485
**Conduct Problems**	MFCC	2	0.5389810504229	0.5316045380875	0.5430463576158	0.5016501650165	0.5206349206349
**Conduct Problems**	MFCC	3	0.5401831573885	0.5280528052805	0.5298013245033	0.5247524752475	0.5263157894736
**Conduct Problems**	MFCC	4	0.5374183113675	0.5359477124183	0.5412541254125	0.5198675496688	0.5307443365695

**Conduct Problems**	MFCC	5	0.5288177824404	0.5245901639344	0.5280528052805	0.5132450331125	0.5211726384364
**Conduct Problems**	MFCC	Mean	0.5416 ± 0.0112	0.5402 ± 0.0206	0.5472 ± 0.0242	0.5215 ± 0.0153	0.5333 ± 0.0176
**Conduct Problems**	MELS	1	0.5149712991101	0.5186385737439	0.5280528052805	0.4917491749174	0.5095541401273
**Conduct Problems**	MELS	2	0.5211133696151	0.5115511551155	0.5132450331125	0.5082508250825	0.5098684210526
**Conduct Problems**	MELS	3	0.4882193517364	0.5008183306055	0.5066225165562	0.4851485148514	0.4951456310679
**Conduct Problems**	MELS	4	0.4901645793718	0.4959481361426	0.5049504950495	0.4668874172185	0.4872611464968
**Conduct Problems**	MELS	5	0.4888204052193	0.5306122448979	0.5577557755775	0.4536423841059	0.5059880239520
**Conduct Problems**	MELS	Mean	0.5007 ± 0.0143	0.5115 ± 0.0124	0.5221 ± 0.0196	0.4811 ± 0.0191	0.5016 ± 0.0089
**Conduct Problems**	PROS	1	0.5520373819560	0.5637795275590	0.5907590759075	0.4950495049504	0.5391566265060
**Conduct Problems**	PROS	2	0.5230039560247	0.5485362095531	0.5894039735099	0.4422442244224	0.5129682997118
**Conduct Problems**	PROS	3	0.5300417458964	0.5417322834645	0.5695364238410	0.4686468646864	0.5165165165165
**Conduct Problems**	PROS	4	0.5414726903153	0.5589660743134	0.5709570957095	0.5264900662251	0.5474683544303
**Conduct Problems**	PROS	5	0.5350031691910	0.534375	0.5643564356435	0.4503311258278	0.5074183976261
**Conduct Problems**	PROS	Mean	0.5363 ± 0.0099	0.5495 ± 0.0108	0.5770 ± 0.0109	0.4766 ± 0.0309	0.5247 ± 0.0157
**Conduct Problems**	FORM	1	0.5400450936182	0.5705521472392	0.6138613861386	0.4620462046204	0.5329512893982
**Conduct Problems**	FORM	2	0.4965466745350	0.5359877488514	0.5794701986754	0.4191419141914	0.4985754985754
**Conduct Problems**	FORM	3	0.5399536642405	0.5749235474006	0.6225165562913	0.4587458745874	0.5340909090909
**Conduct Problems**	FORM	4	0.5724324088037	0.5891238670694	0.6435643564356	0.4569536423841	0.5431754874651
**Conduct Problems**	FORM	5	0.5571984350752	0.6005917159763	0.6699669966996	0.4370860927152	0.5442359249329
**Conduct Problems**	FORM	Mean	0.5412 ± 0.0254	0.5742 ± 0.0219	0.6259 ± 0.0302	0.4468 ± 0.0164	0.5306 ± 0.0167
**Conduct Problems**	MFCC+MELS	1	0.5283251097386	0.5318107667210	0.5379537953795	0.5148514851485	0.5258064516129
**Conduct Problems**	MFCC+MELS	2	0.5101195550018	0.4983388704318	0.4966887417218	0.5049504950495	0.5
**Conduct Problems**	MFCC+MELS	3	0.5125237689331	0.5099337748344	0.5099337748344	0.5115511551155	0.5099337748344
**Conduct Problems**	MFCC+MELS	4	0.5201735405328	0.5358851674641	0.5544554455445	0.4834437086092	0.5185185185185
**Conduct Problems**	MFCC+MELS	5	0.4967433829475	0.4782608695652	0.4719471947194	0.4966887417218	0.4847457627118
**Conduct Problems**	MFCC+MELS	Mean	0.5136 ± 0.0106	0.5108 ± 0.0214	0.5142 ± 0.0293	0.5023 ± 0.0113	0.5078 ± 0.0144
**Conduct Problems**	MFCC+PROS	1	0.5640296702937	0.5677830940988	0.5874587458745	0.5181518151815	0.5493827160493
**Conduct Problems**	MFCC+PROS	2	0.5311782833912	0.5263157894736	0.5463576158940	0.4719471947194	0.5076923076923
**Conduct Problems**	MFCC+PROS	3	0.5372325312001	0.5256198347107	0.5264900662251	0.5247524752475	0.5247524752475
**Conduct Problems**	MFCC+PROS	4	0.5374073831224	0.5348837209302	0.5313531353135	0.5430463576158	0.5384615384615
**Conduct Problems**	MFCC+PROS	5	0.5131903918868	0.4991680532445	0.4950495049504	0.5099337748344	0.5033557046979
**Conduct Problems**	MFCC+PROS	Mean	0.5366 ± 0.0163	0.5308 ± 0.0221	0.5373 ± 0.0301	0.5136 ± 0.0235	0.5247 ± 0.0176
**Conduct Problems**	MFCC+FORM	1	0.5695193281704	0.5691573926868	0.5907590759075	0.5148514851485	0.5490797546012
**Conduct Problems**	MFCC+FORM	2	0.4991475968788	0.5032467532467	0.5132450331125	0.4752475247524	0.4936305732484
**Conduct Problems**	MFCC+FORM	3	0.5338994164317	0.5322580645161	0.5463576158940	0.4950495049504	0.5188679245283
**Conduct Problems**	MFCC+FORM	4	0.5582475466089	0.5526315789473	0.5544554455445	0.5463576158940	0.5508196721311
**Conduct Problems**	MFCC+FORM	5	0.5378007999475	0.5454545454545	0.5544554455445	0.5198675496688	0.5367412140575
**Conduct Problems**	MFCC+FORM	Mean	0.5397 ± 0.0241	0.5405 ± 0.0221	0.5519 ± 0.0247	0.5103 ± 0.0240	0.5298 ± 0.0214
**Conduct Problems**	MELS+PROS	1	0.5202540055985	0.5220228384991	0.5280528052805	0.5049504950495	0.5161290322580
**Conduct Problems**	MELS+PROS	2	0.5218346337945	0.5075125208681	0.5033112582781	0.5214521452145	0.5117845117845
**Conduct Problems**	MELS+PROS	3	0.4995956549297	0.4908180300500	0.4867549668874	0.5049504950495	0.4949494949494
**Conduct Problems**	MELS+PROS	4	0.5188949358512	0.5382165605095	0.5577557755775	0.4834437086092	0.52
**Conduct Problems**	MELS+PROS	5	0.5182173846523	0.5548589341692	0.5841584158415	0.4768211920529	0.5283582089552
**Conduct Problems**	MELS+PROS	Mean	0.5158 ± 0.0082	0.5227 ± 0.0225	0.5320 ± 0.0354	0.4983 ± 0.0162	0.5142 ± 0.0111
**Conduct Problems**	MELS+FORM	1	0.5129453539413	0.4867549668874	0.4851485148514	0.4917491749174	0.4883720930232
**Conduct Problems**	MELS+FORM	2	0.5149170546193	0.5175292153589	0.5132450331125	0.5313531353135	0.5218855218855
**Conduct Problems**	MELS+FORM	3	0.4996502961554	0.5182829888712	0.5397350993377	0.4587458745874	0.4984709480122
**Conduct Problems**	MELS+FORM	4	0.5195943435403	0.5145631067961	0.5247524752475	0.4834437086092	0.5047619047619
**Conduct Problems**	MELS+FORM	5	0.5151028347867	0.5546995377503	0.5940594059405	0.4503311258278	0.5202312138728
**Conduct Problems**	MELS+FORM	Mean	0.5124 ± 0.0068	0.5184 ± 0.0216	0.5314 ± 0.0361	0.4831 ± 0.0285	0.5067 ± 0.0128
**Conduct Problems**	PROS+FORM	1	0.5375507847814	0.5492063492063	0.5709570957095	0.4917491749174	0.5290519877675
**Conduct Problems**	PROS+FORM	2	0.5080104036893	0.5535168195718	0.5993377483443	0.4356435643564	0.5142045454545
**Conduct Problems**	PROS+FORM	3	0.5420081743273	0.5709876543209	0.6125827814569	0.4686468646864	0.5346820809248
**Conduct Problems**	PROS+FORM	4	0.5626625576464	0.5628930817610	0.5907590759075	0.4900662251655	0.5375375375375
**Conduct Problems**	PROS+FORM	5	0.5458002753917	0.5837173579109	0.6270627062706	0.4768211920529	0.5459770114942
**Conduct Problems**	PROS+FORM	Mean	0.5392 ± 0.0178	0.5641 ± 0.0124	0.6001 ± 0.0191	0.4726 ± 0.0204	0.5323 ± 0.0106
**Conduct Problems**	MFCC+MELS+PROS	1	0.5248178283174	0.5248	0.5412541254125	0.4785478547854	0.5093167701863

**Conduct Problems**	MFCC+MELS+PROS	2	0.536401984569:	0.524590163934	0.529801324503:	0.511551155115	0.519480519480
**Conduct Problems**	MFCC+MELS+PROS	3	0.535975783008	0.516020236087	0.506622516556	0.544554455445	0.525773195876
**Conduct Problems**	MFCC+MELS+PROS	4	0.515070050051	0.538827258320	0.561056105610	0.476821192052	0.518292682926
**Conduct Problems**	MFCC+MELS+PROS	5	0.501519026074	0.479865771812	0.471947194719	0.503311258278	0.488054607508
**Conduct Problems**	MFCC+MELS+PROS	Mean	0.5228 ± 0.0132	0.5168 ± 0.0199	0.5221 ± 0.0307	0.5030 ± 0.0248	0.5122 ± 0.0132
**Conduct Problems**	MFCC+MELS+FORM	1	0.541885871755	0.513731825525	0.524752475247	0.481848184818	0.503164556962
**Conduct Problems**	MFCC+MELS+FORM	2	0.526522850960	0.512077294685	0.526490066225	0.471947194719	0.498432601880
**Conduct Problems**	MFCC+MELS+FORM	3	0.533790133980	0.546325878594	0.566225165562	0.495049504950	0.527777777777
**Conduct Problems**	MFCC+MELS+FORM	4	0.520151684042	0.530351437699	0.547854785478	0.480132450331	0.513931888544
**Conduct Problems**	MFCC+MELS+FORM	5	0.513441741525	0.536977491961	0.551155115511	0.496688741721	0.523510971786
**Conduct Problems**	MFCC+MELS+FORM	Mean	0.5272 ± 0.0100	0.5279 ± 0.0133	0.5433 ± 0.0157	0.4851 ± 0.0094	0.5134 ± 0.0113
**Conduct Problems**	MFCC+PROS+FORM	1	0.559661906784	0.549273021001	0.561056105610	0.518151815181	0.537974683544
**Conduct Problems**	MFCC+PROS+FORM	2	0.511081240574	0.528662420382	0.549668874172	0.471947194719	0.509202453987
**Conduct Problems**	MFCC+PROS+FORM	3	0.539276113041	0.542483660130	0.549668874172	0.524752475247	0.535483870967
**Conduct Problems**	MFCC+PROS+FORM	4	0.548947609992	0.528239202657	0.524752475247	0.536423841059	0.531772575250
**Conduct Problems**	MFCC+PROS+FORM	5	0.525266102769	0.528363047001	0.537953795379	0.5	0.519108280254
**Conduct Problems**	MFCC+PROS+FORM	Mean	0.5368 ± 0.0172	0.5354 ± 0.0088	0.5446 ± 0.0123	0.5103 ± 0.0225	0.5267 ± 0.0109
**Conduct Problems**	MELS+PROS+FORM	1	0.513598884640	0.502446982055	0.508250825082	0.485148514851	0.496774193548
**Conduct Problems**	MELS+PROS+FORM	2	0.519386706882	0.515599343185	0.519867549668	0.504950495049	0.511400651465
**Conduct Problems**	MELS+PROS+FORM	3	0.500032784735	0.517571884984	0.536423841059	0.465346534653	0.5
**Conduct Problems**	MELS+PROS+FORM	4	0.526435424999	0.535714285714	0.544554455445	0.509933774834	0.527156549520
**Conduct Problems**	MELS+PROS+FORM	5	0.520020545100	0.561832061068	0.607260726072	0.443708609271	0.522727272727
**Conduct Problems**	MELS+PROS+FORM	Mean	0.5159 ± 0.0089	0.5266 ± 0.0205	0.5433 ± 0.0344	0.4818 ± 0.0247	0.5116 ± 0.0120
**Conduct Problems**	MFCC+MELS+PROS+	1	0.542147284035	0.534181240063	0.554455445544	0.478547854785	0.515337423312
**Conduct Problems**	MFCC+MELS+PROS+	2	0.527080191462	0.510638297872	0.516556291390	0.495049504950	0.504854368932
**Conduct Problems**	MFCC+MELS+PROS+	3	0.534172622560	0.545161290322	0.559602649006	0.508250825082	0.531446540880
**Conduct Problems**	MFCC+MELS+PROS+	4	0.521823705549	0.522151898734	0.544554455445	0.456953642384	0.501519756838
**Conduct Problems**	MFCC+MELS+PROS+	5	0.513168535396	0.536115569823	0.551155115511	0.493377483443	0.521875
**Conduct Problems**	MFCC+MELS+PROS+	Mean	0.5277 ± 0.0100	0.5296 ± 0.0120	0.5453 ± 0.0152	0.4864 ± 0.0175	0.5150 ± 0.0110
**Peer Problems**	MFCC	1	0.531771849924	0.535987748851	0.550314465408	0.498432601880	0.522388059701
**Peer Problems**	MFCC	2	0.494243015713	0.495207667731	0.485893416927	0.522012578616	0.504885993485
**Peer Problems**	MFCC	3	0.574997033345	0.543408360128	0.531446540880	0.575471698113	0.555921052631
**Peer Problems**	MFCC	4	0.533997864008	0.508143322475	0.490566037735	0.559748427672	0.527027027027
**Peer Problems**	MFCC	5	0.567600174043	0.561514195583	0.559748427672	0.566037735849	0.563291139240
**Peer Problems**	MFCC	Mean	0.5405 ± 0.0289	0.5289 ± 0.0240	0.5236 ± 0.0303	0.5443 ± 0.0293	0.5347 ± 0.0218
**Peer Problems**	MELS	1	0.507245519607	0.487883683360	0.474842767295	0.529780564263	0.501661129568
**Peer Problems**	MELS	2	0.549900435716	0.529695024077	0.517241379310	0.562893081761	0.542763157894
**Peer Problems**	MELS	3	0.533602310035	0.5	0.484276729559	0.547169811320	0.516778523489
**Peer Problems**	MELS	4	0.553815118072	0.507223113964	0.496855345911	0.537735849056	0.518032786885
**Peer Problems**	MELS	5	0.606572129267	0.576433121019	0.569182389937	0.594339622641	0.583870967741
**Peer Problems**	MELS	Mean	0.5502 ± 0.0326	0.5202 ± 0.0312	0.5085 ± 0.0335	0.5544 ± 0.0228	0.5326 ± 0.0288
**Peer Problems**	PROS	1	0.501035074229	0.475590551181	0.474842767295	0.479623824451	0.476340694006
**Peer Problems**	PROS	2	0.517951144496	0.444444444444	0.401253918495	0.594339622641	0.498054474708
**Peer Problems**	PROS	3	0.540465171472	0.538940809968	0.544025157232	0.525157232704	0.533950617283
**Peer Problems**	PROS	4	0.533048534472	0.533541341653	0.537735849056	0.522012578616	0.529411764705
**Peer Problems**	PROS	5	0.550492464696	0.503401360544	0.465408805031	0.616352201257	0.548148148148
**Peer Problems**	PROS	Mean	0.5286 ± 0.0174	0.4992 ± 0.0356	0.4847 ± 0.0525	0.5475 ± 0.0504	0.5172 ± 0.0262
**Peer Problems**	FORM	1	0.518069438694	0.539027982326	0.575471698113	0.442006269592	0.506925207756
**Peer Problems**	FORM	2	0.537459829262	0.558912386706	0.579937304075	0.503144654088	0.539358600583
**Peer Problems**	FORM	3	0.556712550927	0.586357039187	0.635220125786	0.468553459119	0.544474393530
**Peer Problems**	FORM	4	0.580495233574	0.560121765601	0.578616352201	0.512578616352	0.542772861356
**Peer Problems**	FORM	5	0.549127803488	0.582972582972	0.635220125786	0.455974842767	0.538666666666
**Peer Problems**	FORM	Mean	0.5484 ± 0.0207	0.5655 ± 0.0174	0.6009 ± 0.0281	0.4765 ± 0.0272	0.5344 ± 0.0139
**Peer Problems**	MFCC+MELS	1	0.521647838173	0.500807754442	0.487421383647	0.542319749216	0.514950166112
**Peer Problems**	MFCC+MELS	2	0.526823209321	0.495921696574	0.476489028213	0.553459119496	0.517006802721
**Peer Problems**	MFCC+MELS	3	0.554596337170	0.5	0.477987421383	0.566037735849	0.524137931034
**Peer Problems**	MFCC+MELS	4	0.539149954511	0.529968454258	0.528301886792	0.534591194968	0.531645569620

**Peer Problems**	MFCC+MELS	5	0.6105474466991	0.5755166931637	0.5691823899371	0.5911949685534	0.5819935691318
**Peer Problems**	MFCC+MELS	Mean	0.5506 ± 0.0321	0.5204 ± 0.0301	0.5079 ± 0.0360	0.5575 ± 0.0199	0.5339 ± 0.0247
**Peer Problems**	MFCC+PROS	1	0.5389976538317	0.5449010654490	0.5628930817610	0.4984326018808	0.5280235988200
**Peer Problems**	MFCC+PROS	2	0.5066343329193	0.4968553459119	0.4952978056426	0.5	0.4984227129337
**Peer Problems**	MFCC+PROS	3	0.5628139709663	0.5489566613162	0.5377358490566	0.5786163522012	0.5606557377049
**Peer Problems**	MFCC+PROS	4	0.5483861397887	0.5362776025236	0.5345911949685	0.5408805031446	0.5379746835443
**Peer Problems**	MFCC+PROS	5	0.5663344013290	0.5426356589147	0.5503144654088	0.5220125786163	0.5351681957186
**Peer Problems**	MFCC+PROS	Mean	0.5446 ± 0.0214	0.5339 ± 0.0190	0.5362 ± 0.0228	0.5280 ± 0.0297	0.5320 ± 0.0201
**Peer Problems**	MFCC+FORM	1	0.5419352930738	0.5382262996941	0.5534591194968	0.4984326018808	0.5238095238095
**Peer Problems**	MFCC+FORM	2	0.5360107253405	0.5200642054574	0.5078369905956	0.5534591194968	0.5328947368421
**Peer Problems**	MFCC+FORM	3	0.5924409635694	0.5798449612403	0.5880503144654	0.5597484276729	0.5718654434250
**Peer Problems**	MFCC+FORM	4	0.5424528301886	0.5425867507886	0.5408805031446	0.5471698113207	0.5443037974683
**Peer Problems**	MFCC+FORM	5	0.5499386891341	0.5631659056316	0.5817610062893	0.5157232704402	0.5457227138643
**Peer Problems**	MFCC+FORM	Mean	0.5526 ± 0.0204	0.5488 ± 0.0207	0.5544 ± 0.0291	0.5349 ± 0.0237	0.5437 ± 0.0162
**Peer Problems**	MELS+PROS	1	0.5084876086827	0.4861337683523	0.4685534591194	0.5423197492163	0.5050847457627
**Peer Problems**	MELS+PROS	2	0.5474655468149	0.5154975530179	0.4952978056426	0.5723270440251	0.5374149659863
**Peer Problems**	MELS+PROS	3	0.5379929591392	0.5065789473684	0.4842767295597	0.5723270440251	0.5310344827586
**Peer Problems**	MELS+PROS	4	0.5426209406273	0.5339506172839	0.5440251572327	0.5062893081761	0.5242424242424
**Peer Problems**	MELS+PROS	5	0.5852319924053	0.5595054095826	0.5691823899371	0.5345911949685	0.5501519756838
**Peer Problems**	MELS+PROS	Mean	0.5444 ± 0.0245	0.5203 ± 0.0249	0.5123 ± 0.0380	0.5456 ± 0.0249	0.5296 ± 0.0149
**Peer Problems**	MELS+FORM	1	0.5353601072534	0.5243902439024	0.5408805031446	0.4796238244514	0.5088757396449
**Peer Problems**	MELS+FORM	2	0.5480471599534	0.5049504950495	0.4796238244514	0.5786163522012	0.5331010452961
**Peer Problems**	MELS+FORM	3	0.5599264269609	0.5287713841368	0.5345911949685	0.5125786163522	0.5230769230769
**Peer Problems**	MELS+FORM	4	0.5623788615956	0.553125	0.5566037735849	0.5440251572327	0.5496894409937
**Peer Problems**	MELS+FORM	5	0.5917388552668	0.6074074074074	0.6446540880503	0.5220125786163	0.5742296918767
**Peer Problems**	MELS+FORM	Mean	0.5595 ± 0.0188	0.5437 ± 0.0353	0.5513 ± 0.0534	0.5274 ± 0.0330	0.5378 ± 0.0226
**Peer Problems**	PROS+FORM	1	0.5311606632361	0.5443786982248	0.5786163522012	0.4545454545454	0.5139664804469
**Peer Problems**	PROS+FORM	2	0.5522663196703	0.5435114503816	0.5579937304075	0.5031446540880	0.5297619047619
**Peer Problems**	PROS+FORM	3	0.5506605751354	0.5582089552238	0.5880503144654	0.4811320754716	0.53125
**Peer Problems**	PROS+FORM	4	0.5597583165222	0.5488372093023	0.5566037735849	0.5283018867924	0.5412844036697
**Peer Problems**	PROS+FORM	5	0.5693702780744	0.6046511627906	0.6540880503144	0.4905660377358	0.5621621621621
**Peer Problems**	PROS+FORM	Mean	0.5526 ± 0.0126	0.5599 ± 0.0230	0.5871 ± 0.0356	0.4915 ± 0.0243	0.5357 ± 0.0159
**Peer Problems**	MFCC+MELS+PROS	1	0.5229096429486	0.5137318255250	0.5	0.5548589341692	0.5282392026578
**Peer Problems**	MFCC+MELS+PROS	2	0.5400820173103	0.5191082802547	0.5109717868338	0.5408805031446	0.5275080906148
**Peer Problems**	MFCC+MELS+PROS	3	0.5512934614928	0.5154975530179	0.4968553459119	0.5691823899371	0.5355932203389
**Peer Problems**	MFCC+MELS+PROS	4	0.5480400300621	0.5318818040435	0.5377358490566	0.5157232704402	0.5261538461538
**Peer Problems**	MFCC+MELS+PROS	5	0.5927574067481	0.54375	0.5471698113207	0.5345911949685	0.5403726708074
**Peer Problems**	MFCC+MELS+PROS	Mean	0.5510 ± 0.0231	0.5248 ± 0.0114	0.5185 ± 0.0203	0.5430 ± 0.0181	0.5316 ± 0.0055
**Peer Problems**	MFCC+MELS+FORM	1	0.5457601387985	0.5448818897637	0.5440251572327	0.5485893416927	0.5457413249211
**Peer Problems**	MFCC+MELS+FORM	2	0.5478105715581	0.5307443365695	0.5141065830721	0.5754716981132	0.5484949832775
**Peer Problems**	MFCC+MELS+FORM	3	0.5833728887306	0.5691823899371	0.5691823899371	0.5691823899371	0.5691823899371
**Peer Problems**	MFCC+MELS+FORM	4	0.5512143506981	0.5527950310559	0.5597484276729	0.5345911949685	0.5460122699386
**Peer Problems**	MFCC+MELS+FORM	5	0.5880799810134	0.5665634674922	0.5754716981132	0.5440251572327	0.5579268292682
**Peer Problems**	MFCC+MELS+FORM	Mean	0.5632 ± 0.0185	0.5528 ± 0.0142	0.5525 ± 0.0219	0.5544 ± 0.0155	0.5535 ± 0.0090
**Peer Problems**	MFCC+PROS+FORM	1	0.5288342106819	0.5432473444613	0.5628930817610	0.4921630094043	0.5249266862170
**Peer Problems**	MFCC+PROS+FORM	2	0.5450898050117	0.5211726384364	0.5015673981191	0.5754716981132	0.5423728813559
**Peer Problems**	MFCC+PROS+FORM	3	0.5889304220560	0.5930408472012	0.6163522012578	0.5377358490566	0.5714285714285
**Peer Problems**	MFCC+PROS+FORM	4	0.5388137336339	0.5548589341692	0.5566037735849	0.5503144654088	0.553125
**Peer Problems**	MFCC+PROS+FORM	5	0.5485740279261	0.5757121439280	0.6037735849056	0.5062893081761	0.5501432664756
**Peer Problems**	MFCC+PROS+FORM	Mean	0.5500 ± 0.0206	0.5576 ± 0.0250	0.5682 ± 0.0405	0.5324 ± 0.0300	0.5484 ± 0.0151
**Peer Problems**	MELS+PROS+FORM	1	0.5299777212594	0.5274390243902	0.5440251572327	0.4827586206896	0.5118343195266
**Peer Problems**	MELS+PROS+FORM	2	0.5529169377575	0.5352564102564	0.5235109717868	0.5660377358490	0.5475409836065
**Peer Problems**	MELS+PROS+FORM	3	0.5579288793955	0.5396341463414	0.5566037735849	0.4937106918238	0.5236686390532
**Peer Problems**	MELS+PROS+FORM	4	0.5556346663502	0.5426356589147	0.5503144654088	0.5220125786163	0.5351681957186
**Peer Problems**	MELS+PROS+FORM	5	0.5854594359400	0.5804511278195	0.6069182389937	0.5157232704402	0.5561959654178
**Peer Problems**	MELS+PROS+FORM	Mean	0.5564 ± 0.0177	0.5451 ± 0.0184	0.5563 ± 0.0277	0.5160 ± 0.0288	0.5349 ± 0.0159
**Peer Problems**	MFCC+MELS+PROS+	1	0.5419057195244	0.5306122448979	0.5314465408805	0.5297805642633	0.5297805642633

**Peer Problems**	MFCC+MELS+PROS+	2	0.54111709153	0.51373182552	0.49843260188	0.55660377358	0.53
**Peer Problems**	MFCC+MELS+PROS+	3	0.58186978363	0.56597774244	0.55974842767	0.58176100628	0.57234726688
**Peer Problems**	MFCC+MELS+PROS+	4	0.54863336102	0.54601226993	0.55974842767	0.50943396226	0.53293413173
**Peer Problems**	MFCC+MELS+PROS+	5	0.59156085597	0.58409785932	0.60062893081	0.54402515723	0.56845238095
**Peer Problems**	MFCC+MELS+PROS+	Mean	0.5610 ± 0.0214	0.5481 ± 0.0249	0.5500 ± 0.0339	0.5443 ± 0.0244	0.5467 ± 0.0194
