# Supplementary figures and images for: Using Speech Features and Machine Learning Models to Predict Emotional and Behavioral Problems in Chinese Adolescents
Source: Depress Anxiety. 2025 Jun 16;2025:5734107. doi: 10.1155/da/5734107 (PMC12185205; doi:10.1155/da/5734107)

Male

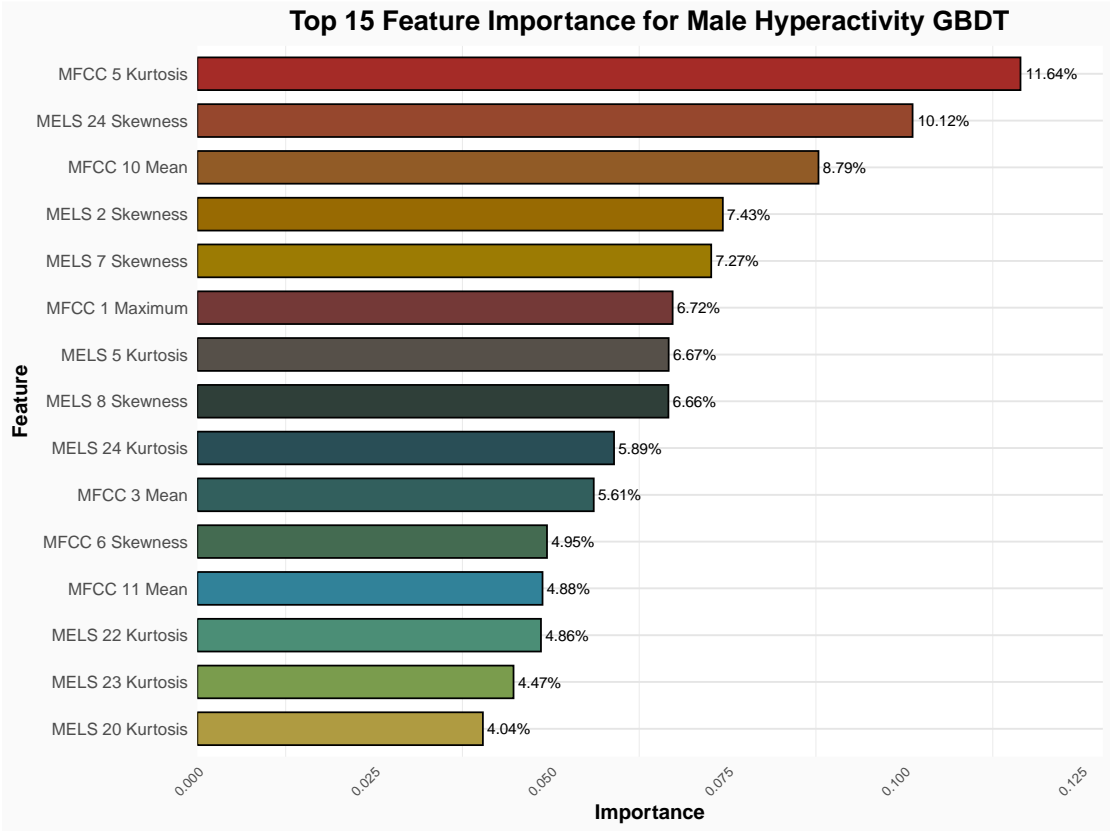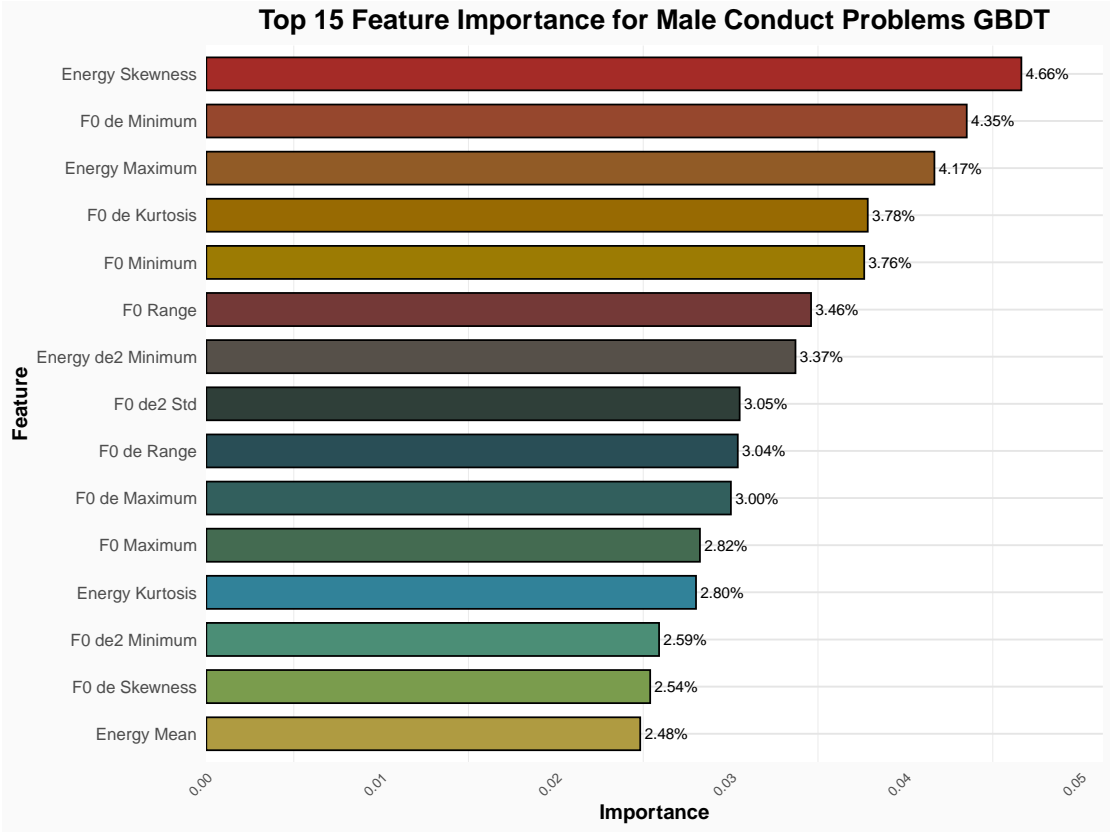

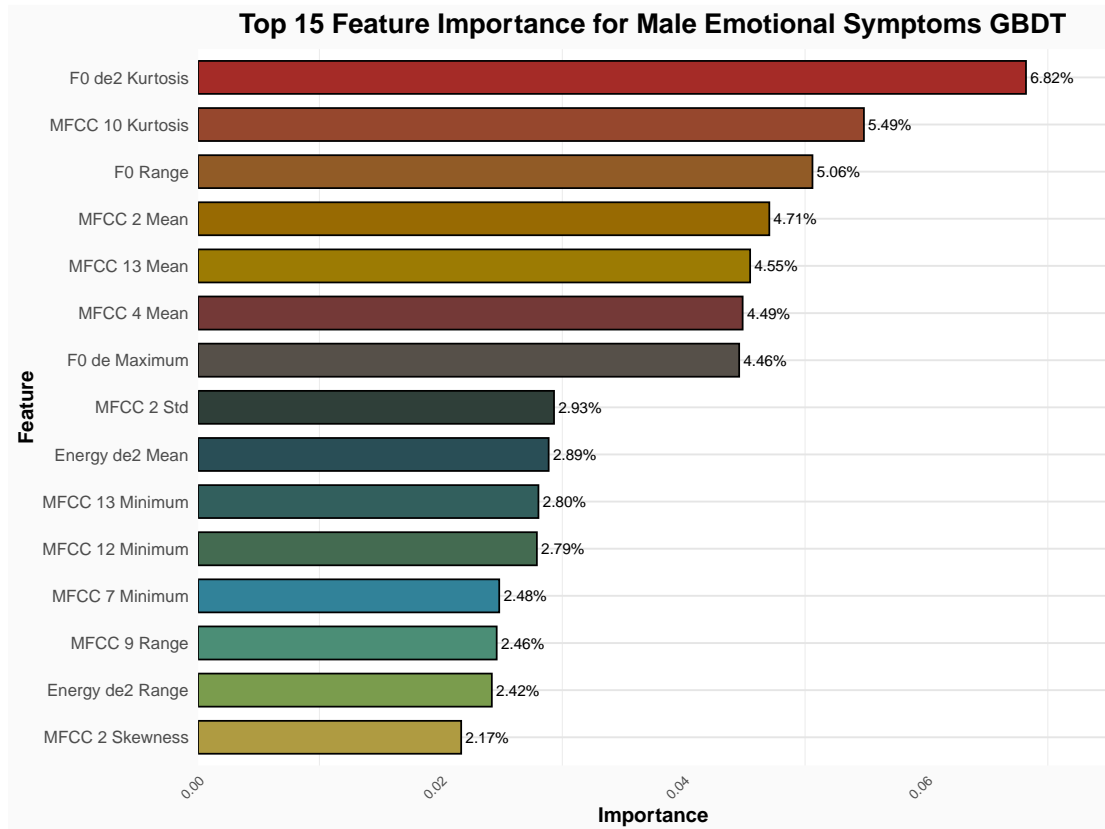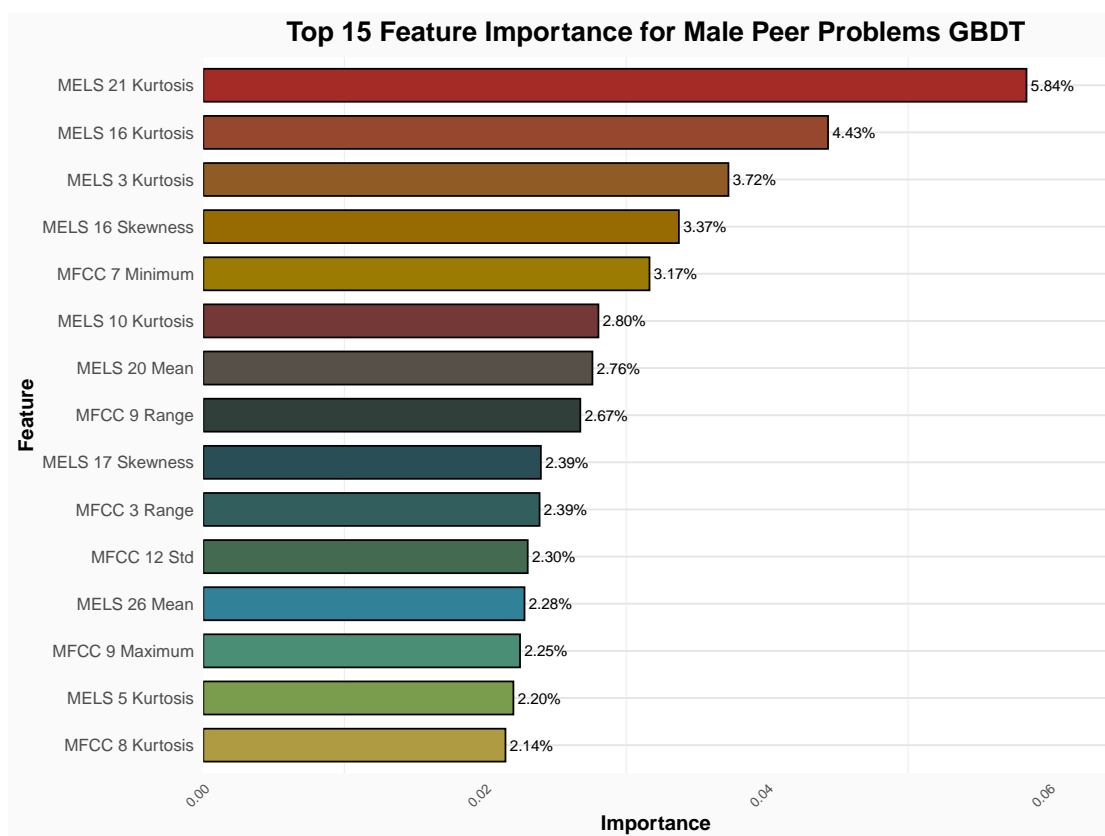

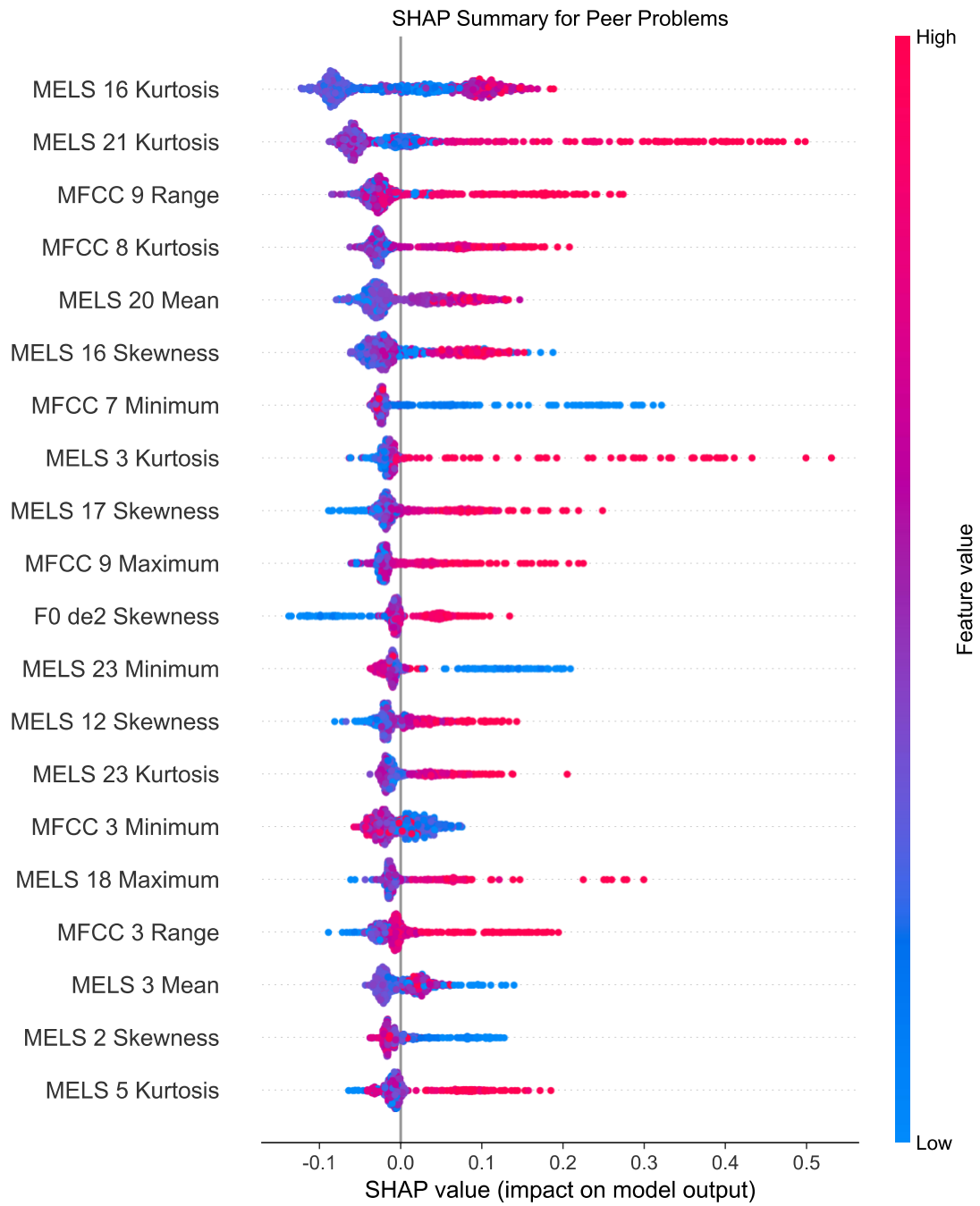

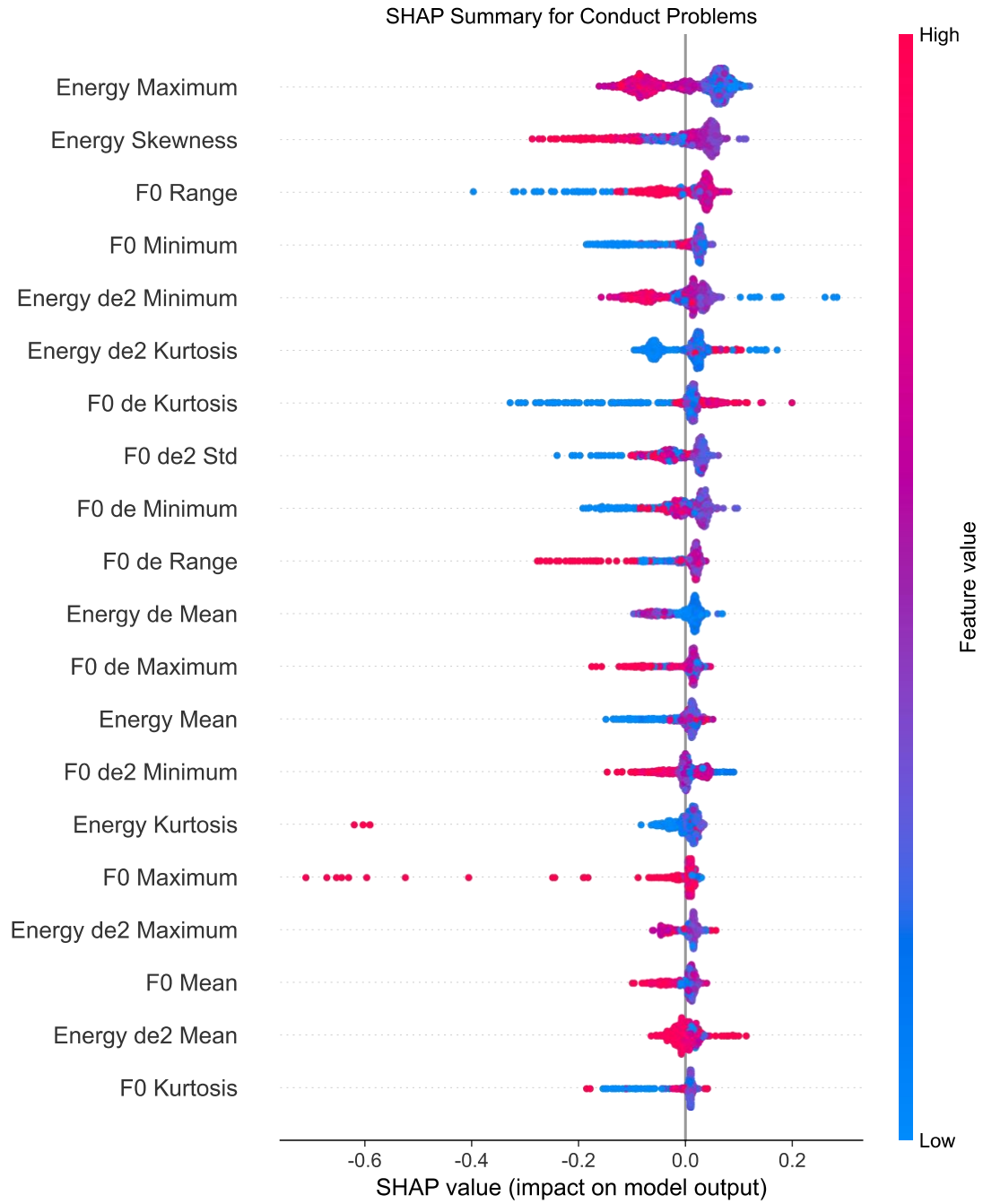

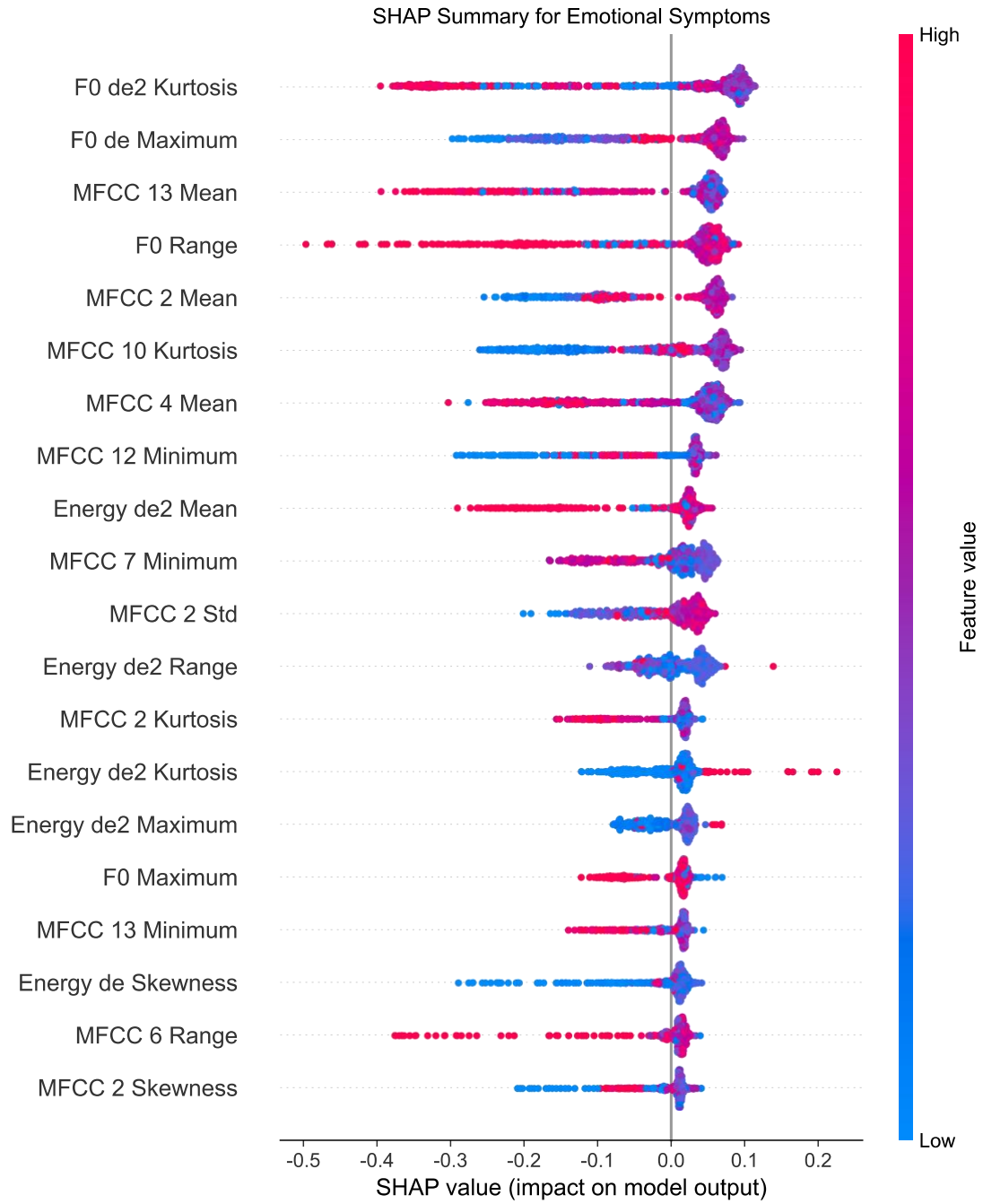

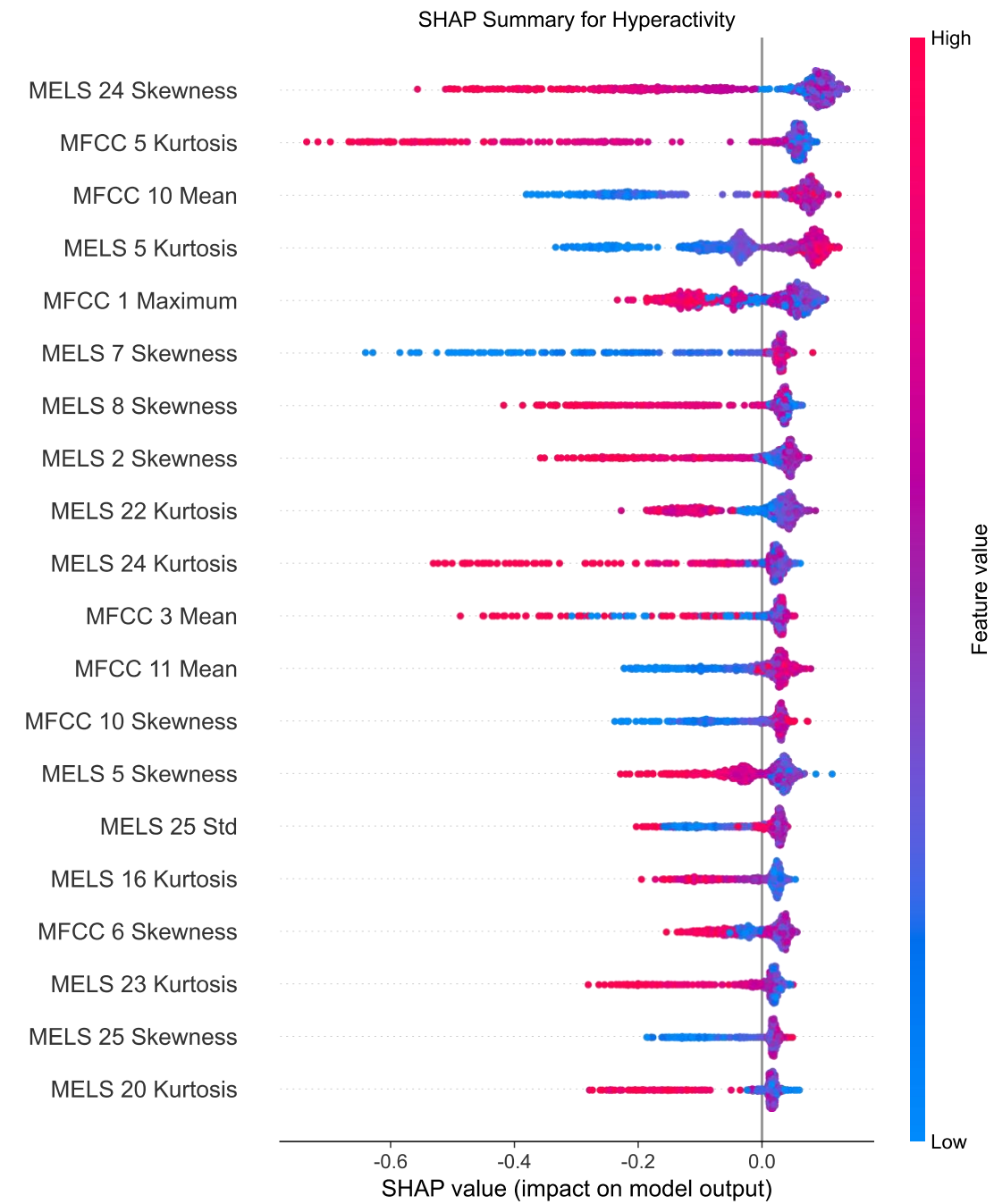

**Female**

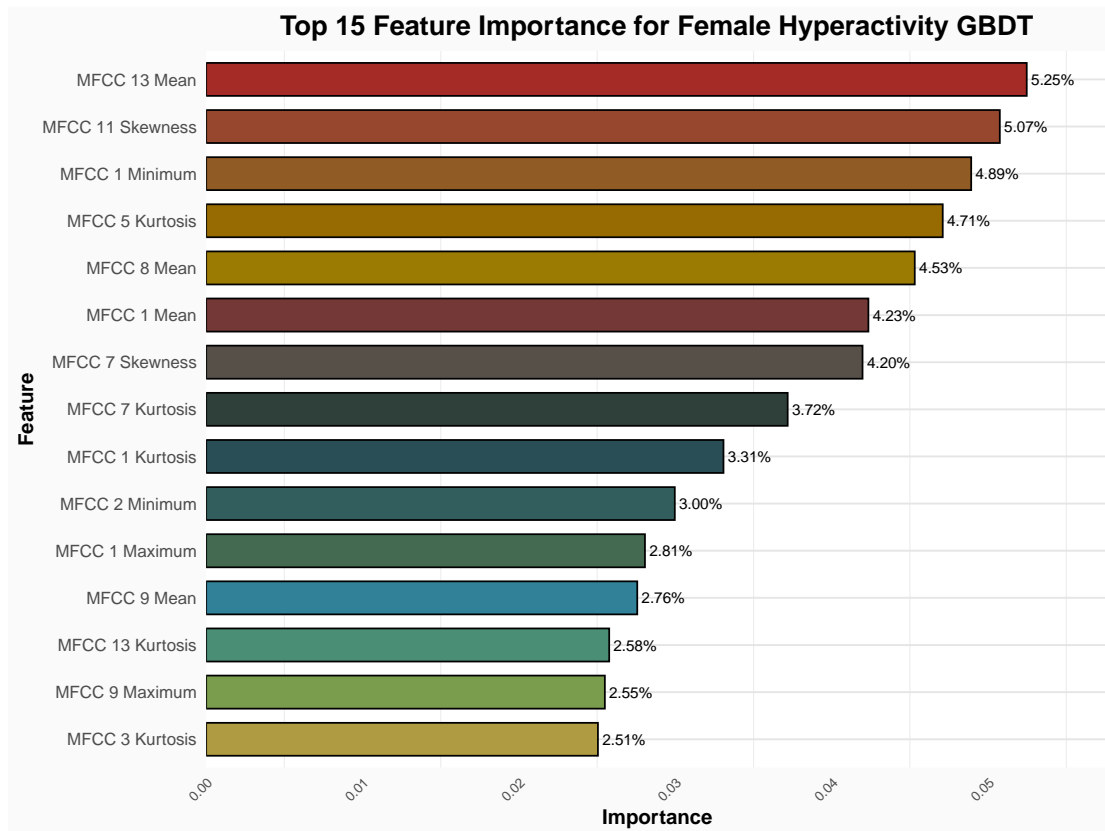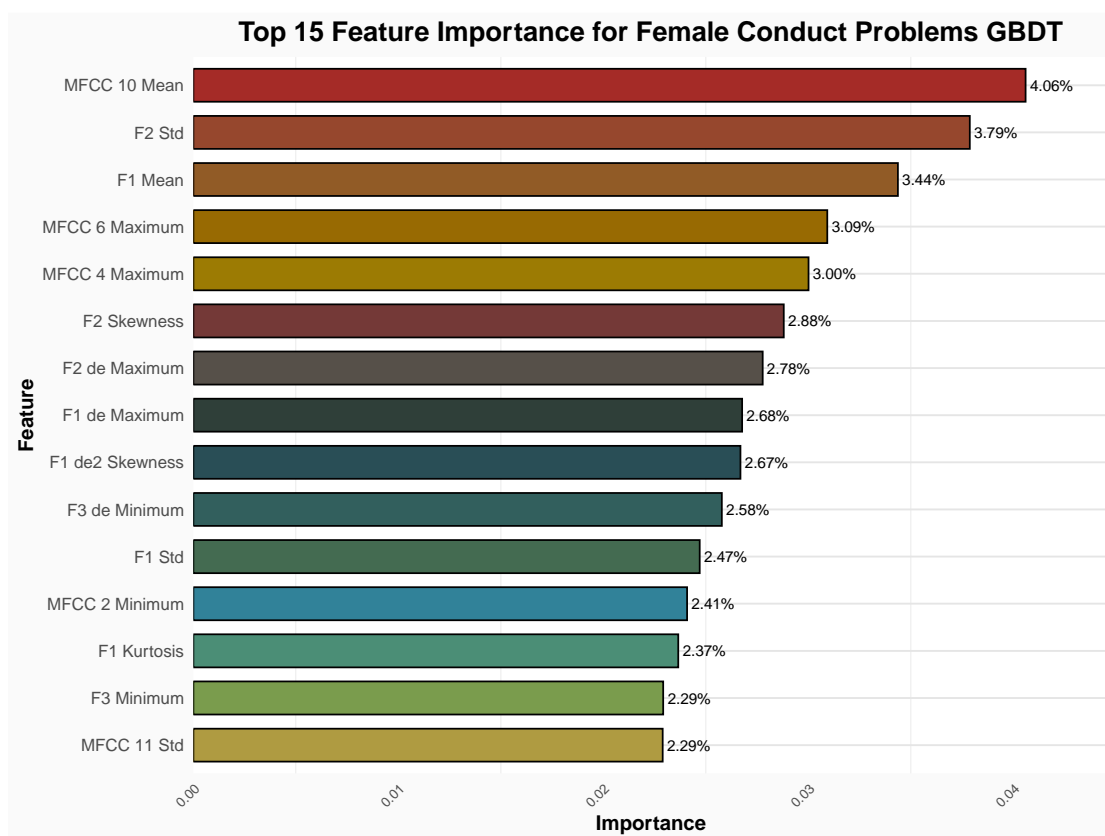

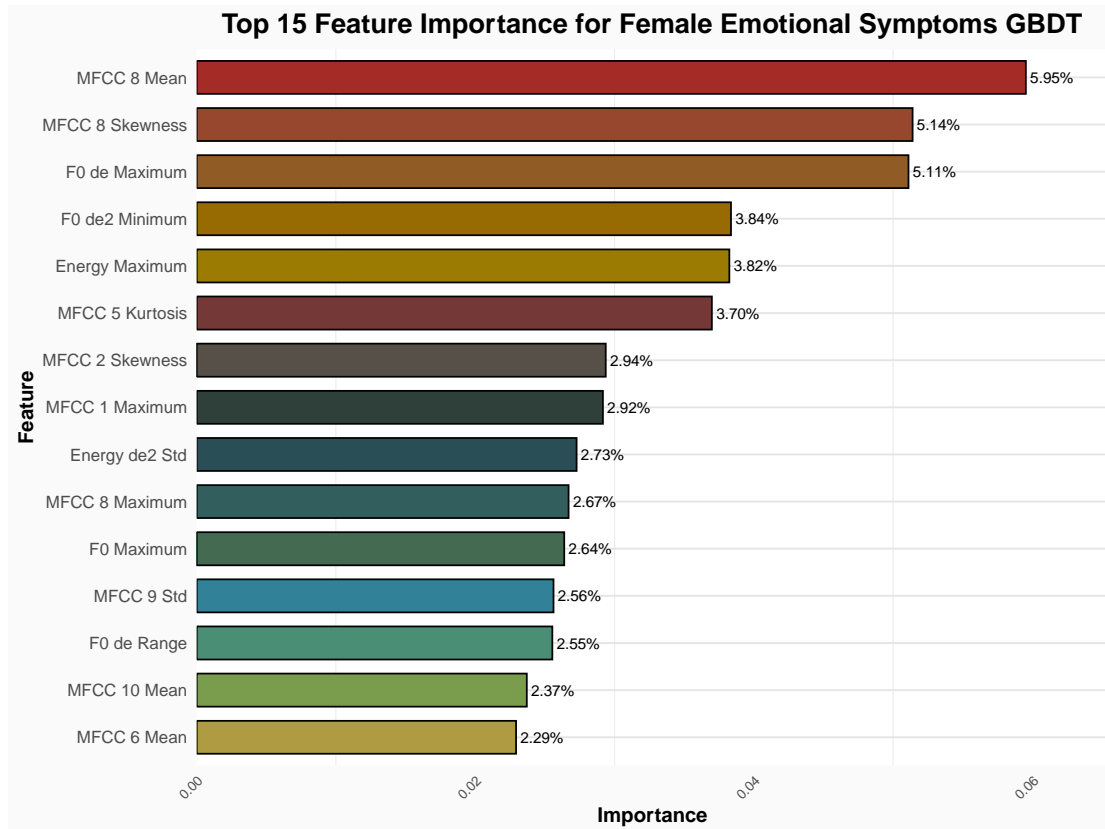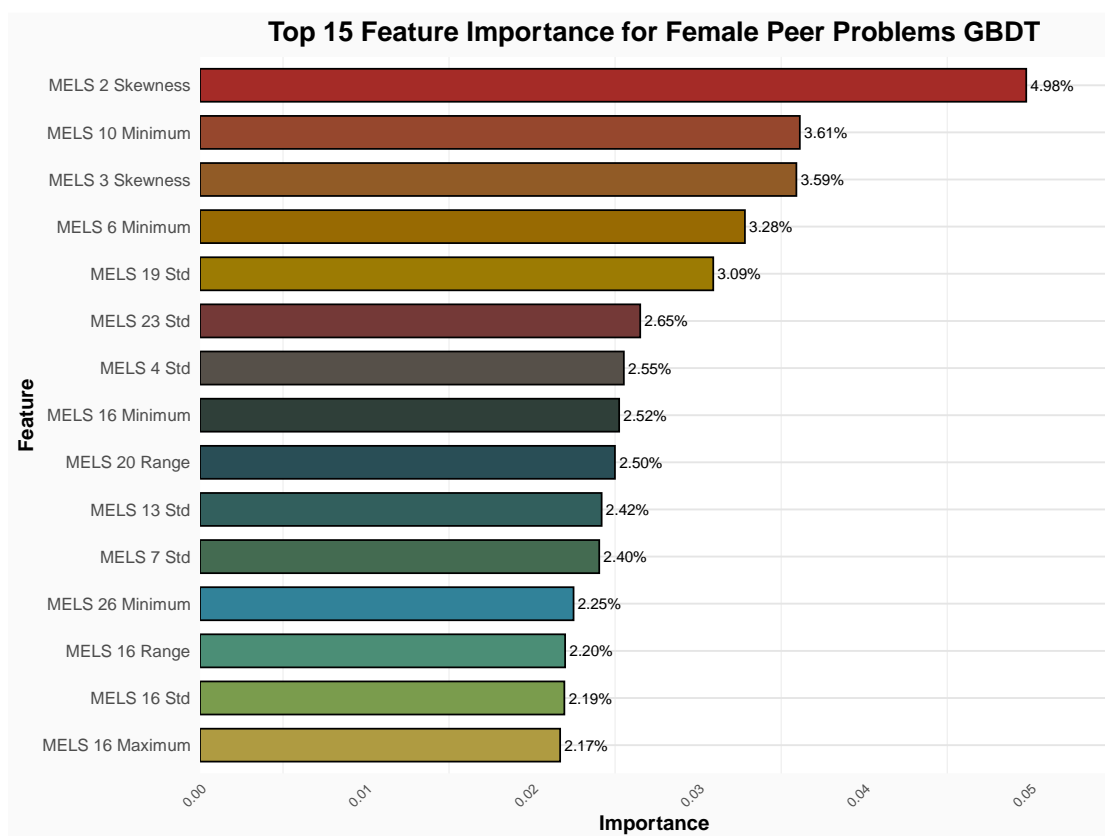

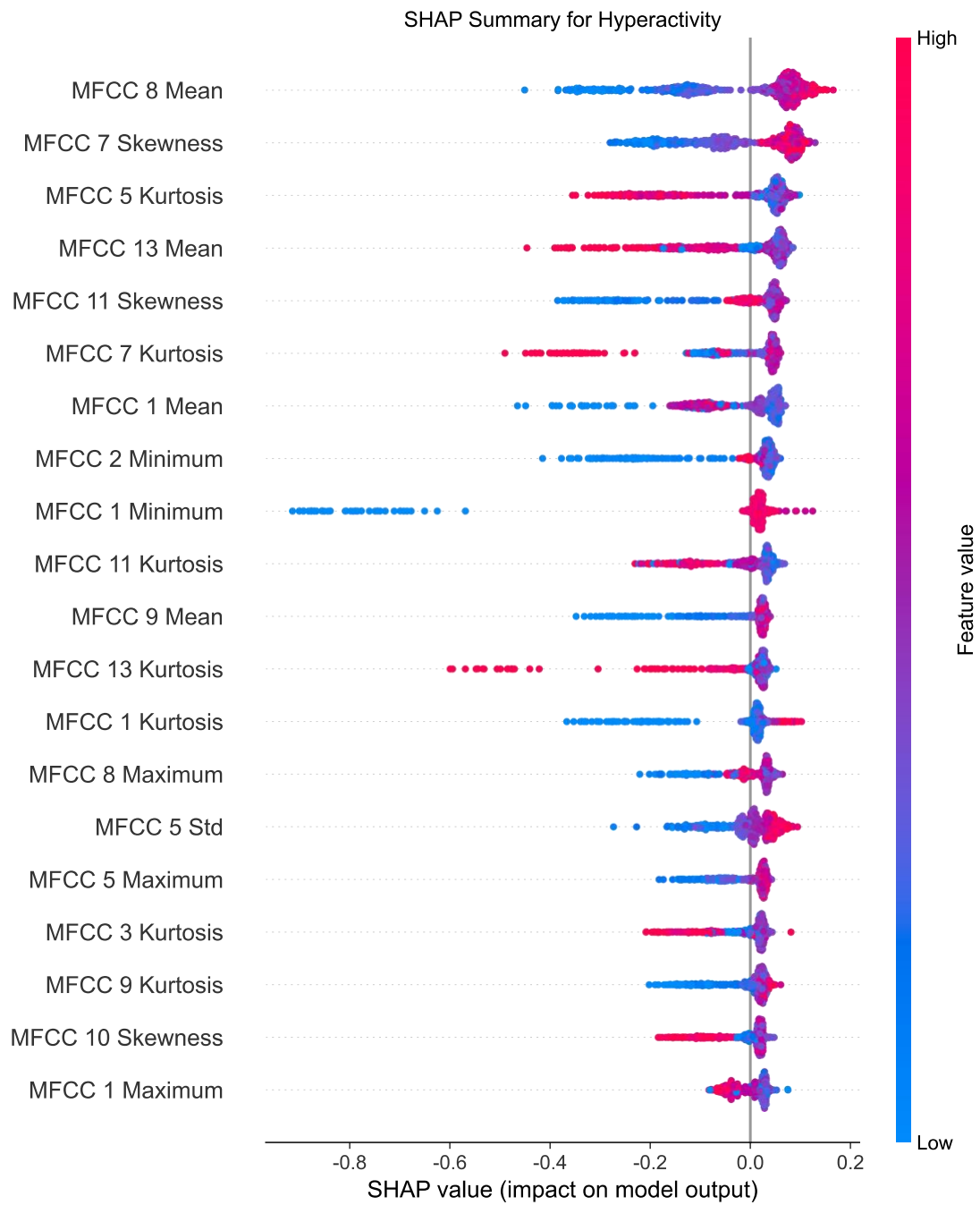

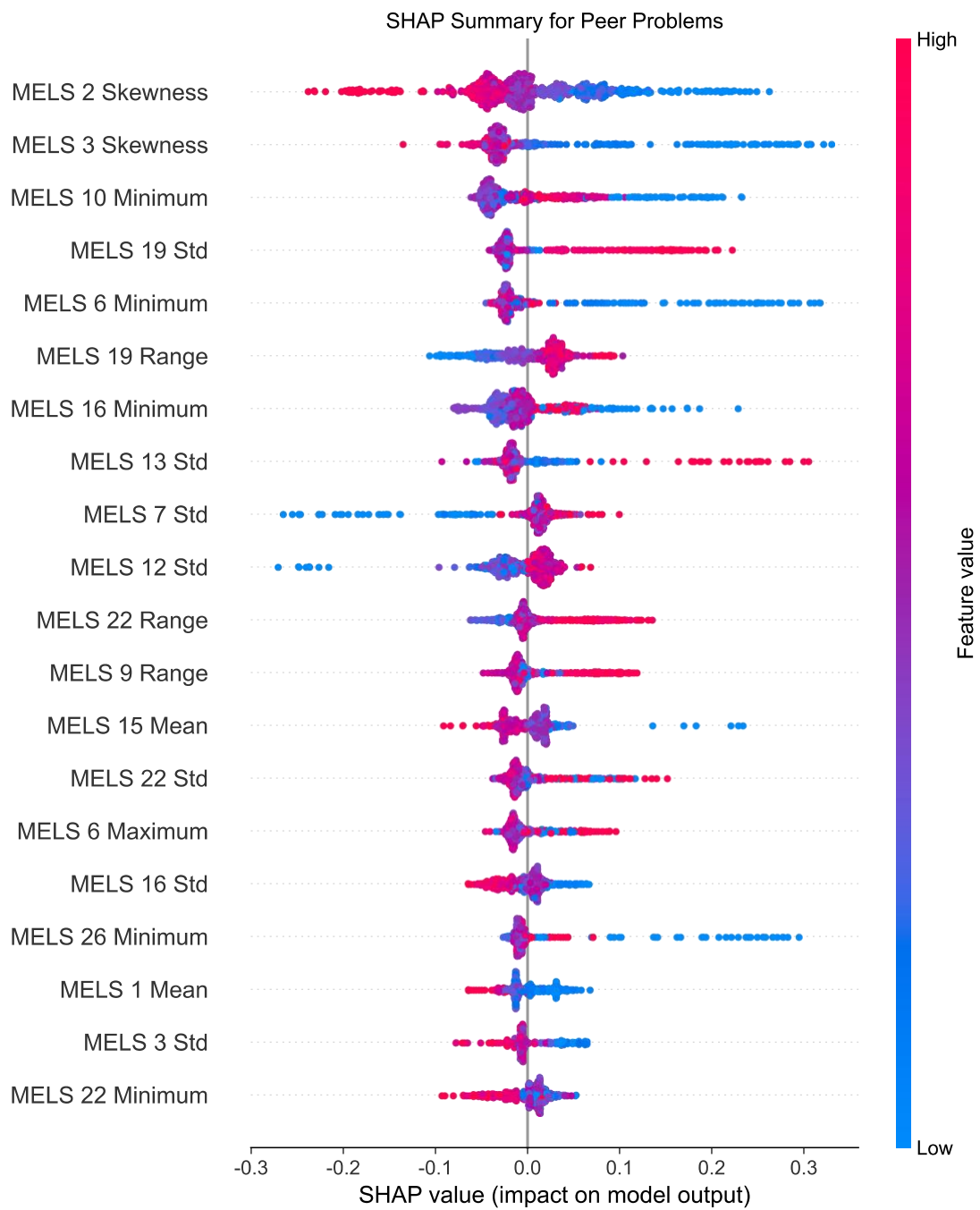

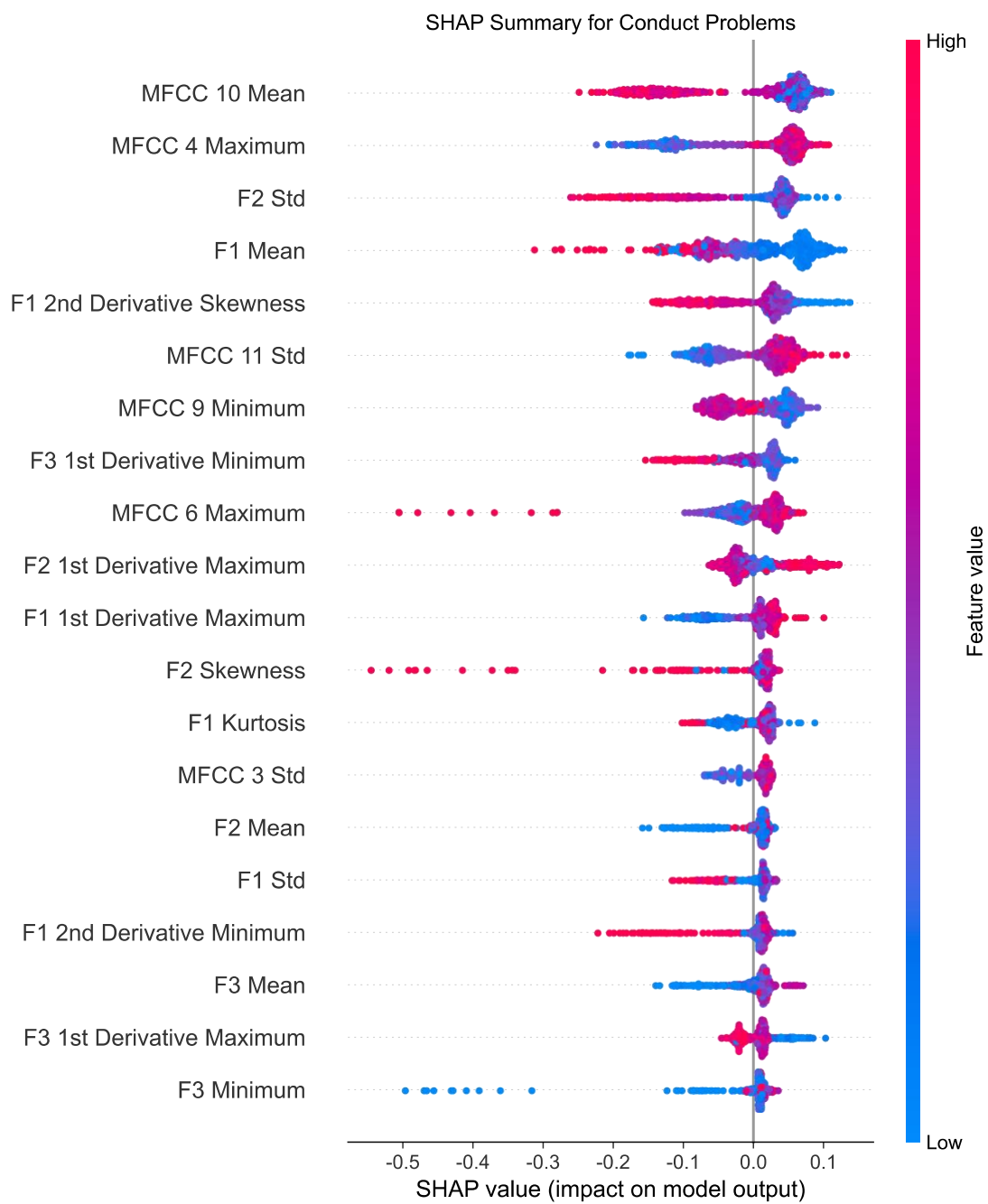

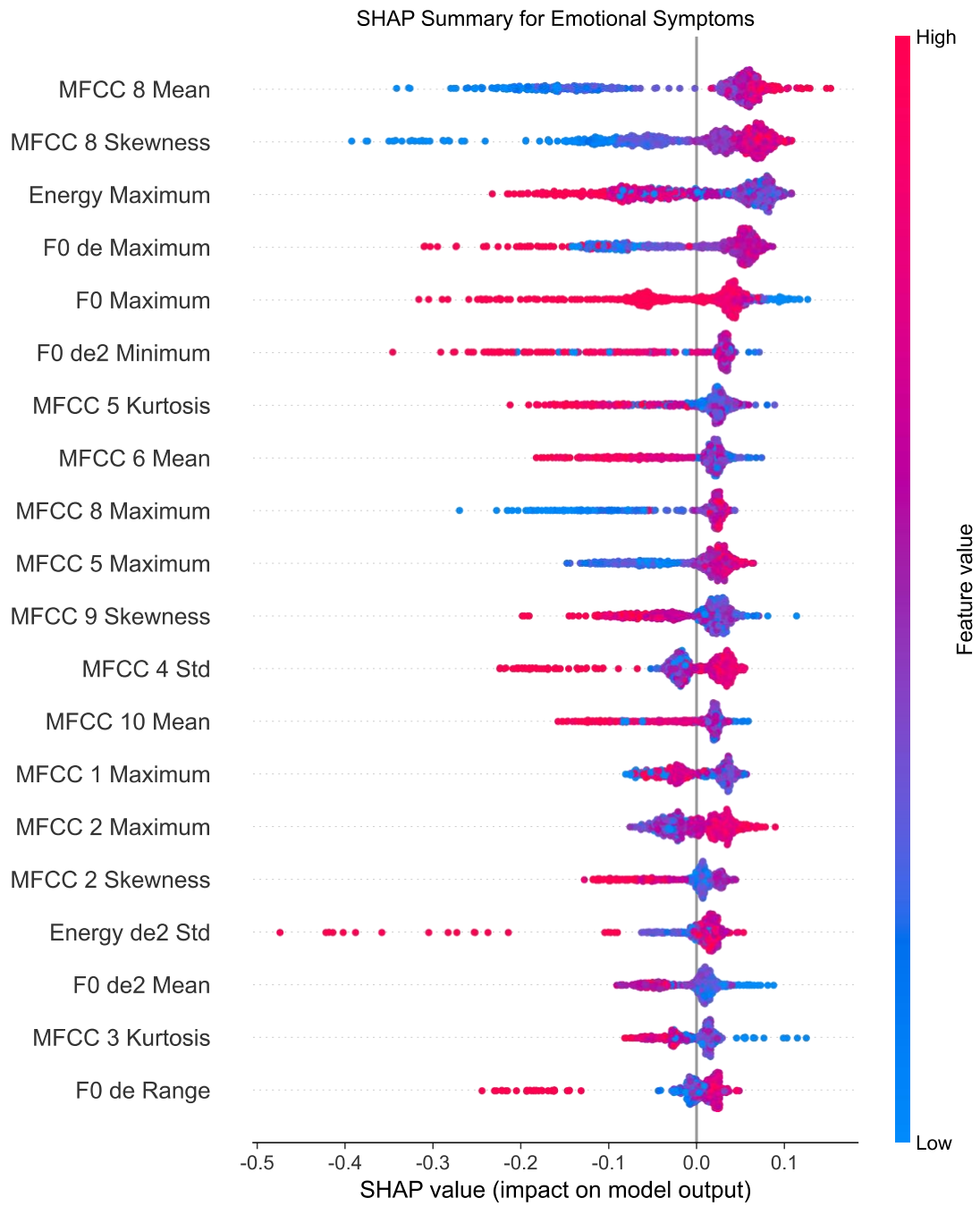

Supplement: Supporting Information 1 — The supporting information file includes all additional figures and tables referenced in the main text as Appendix A–G. [file 5734107.f1.zip › Appendix F.pdf]

Male

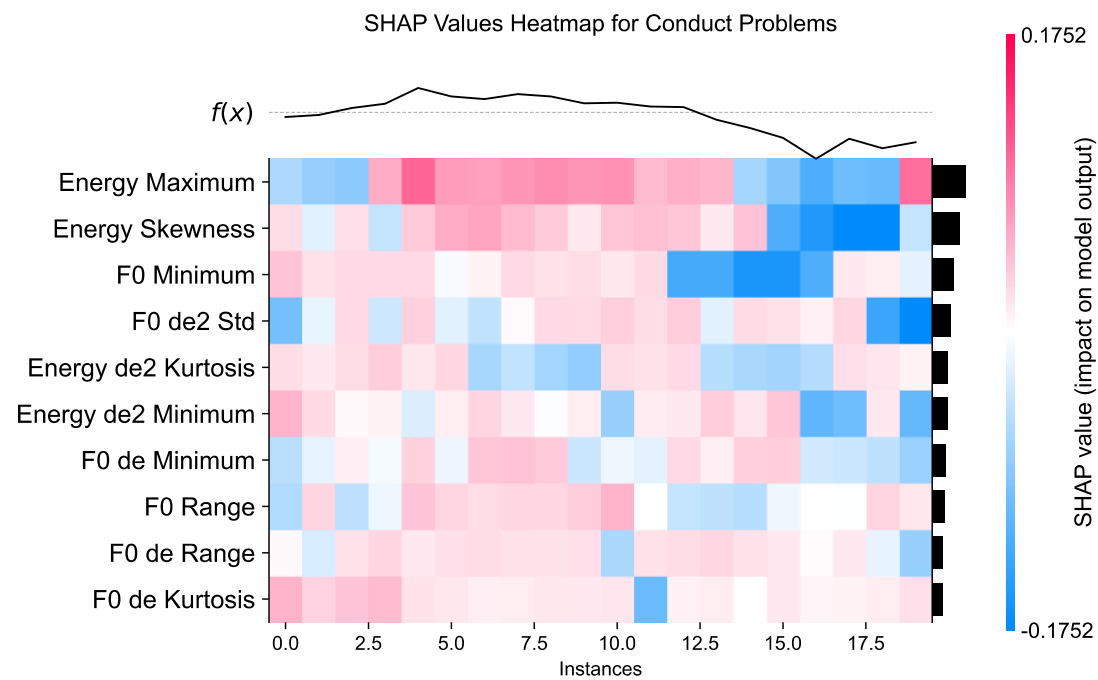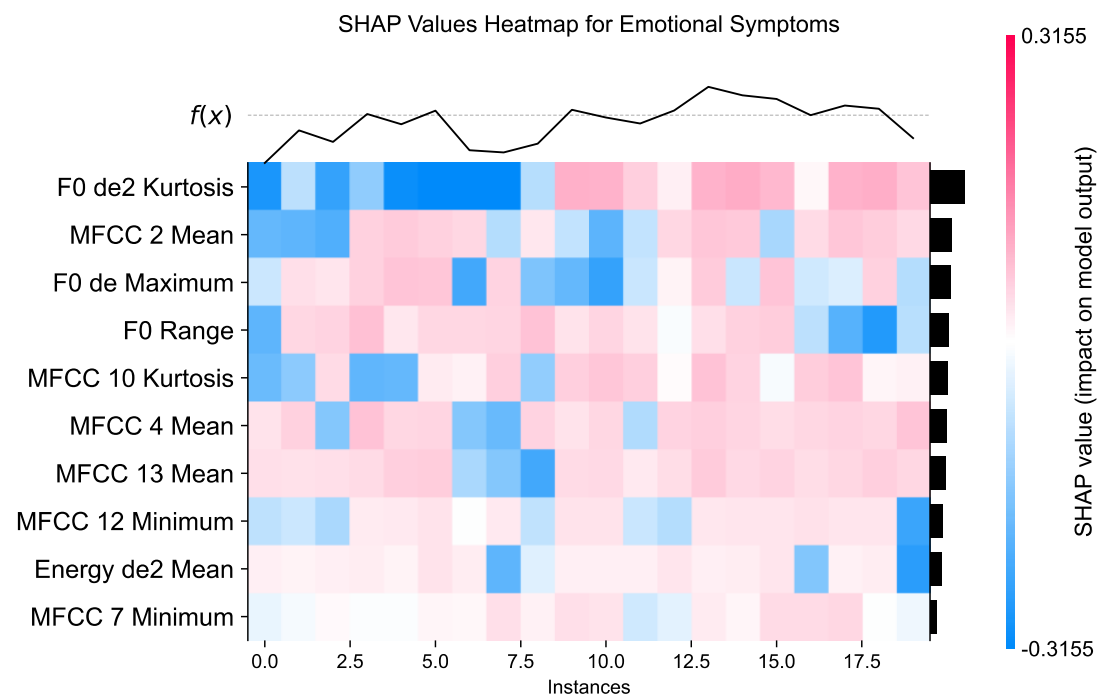

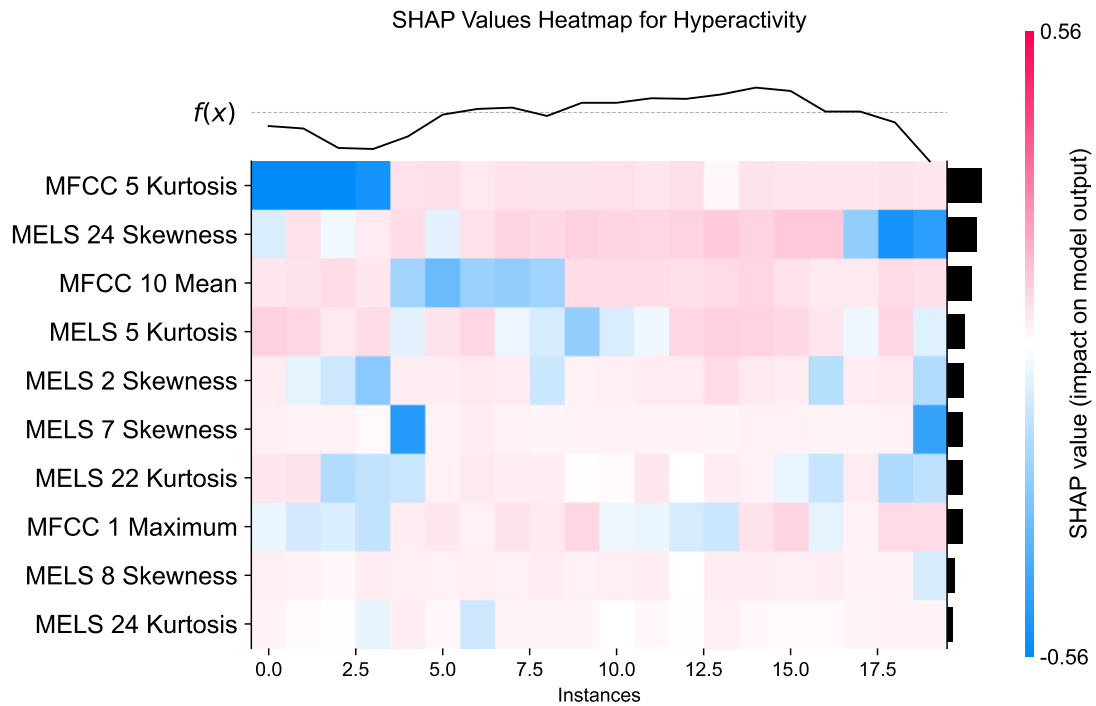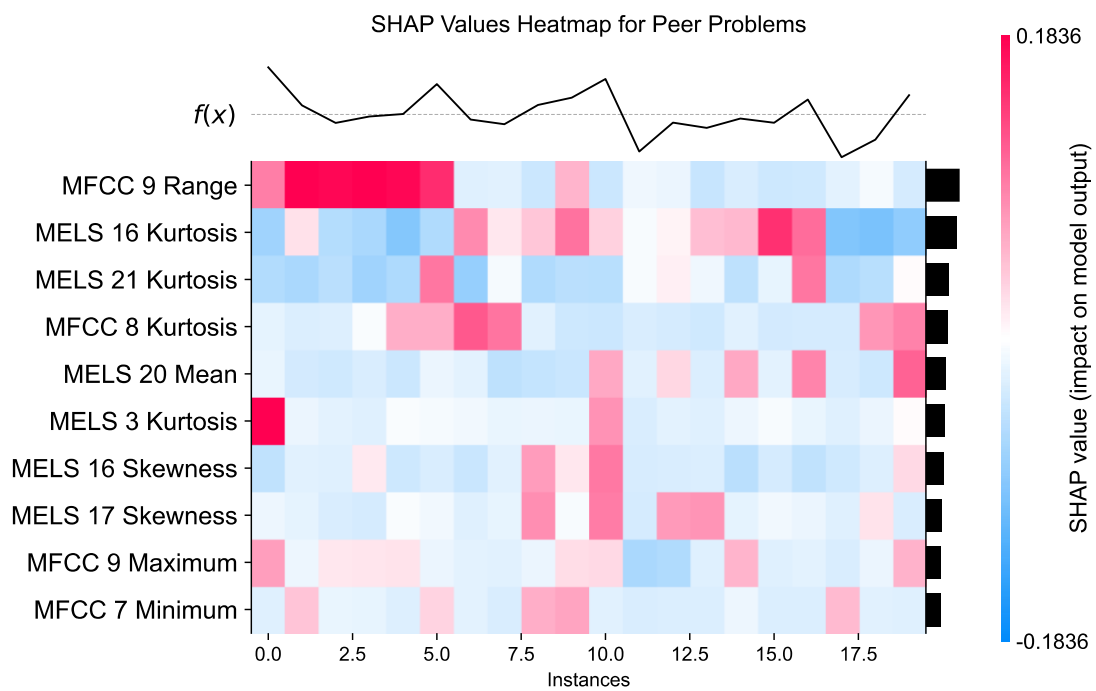

**Female**

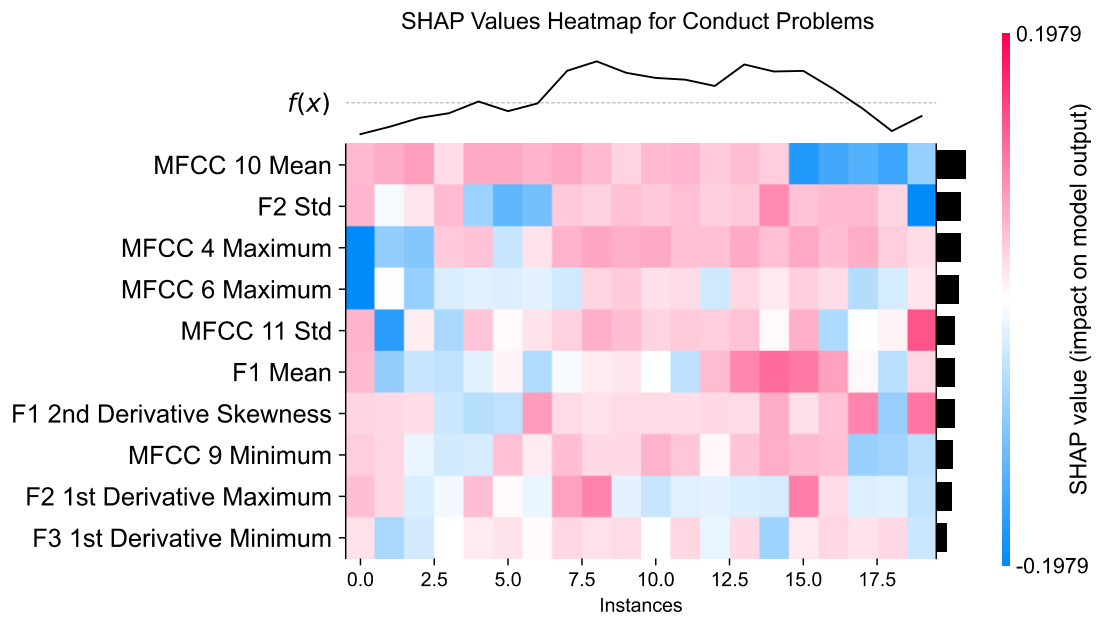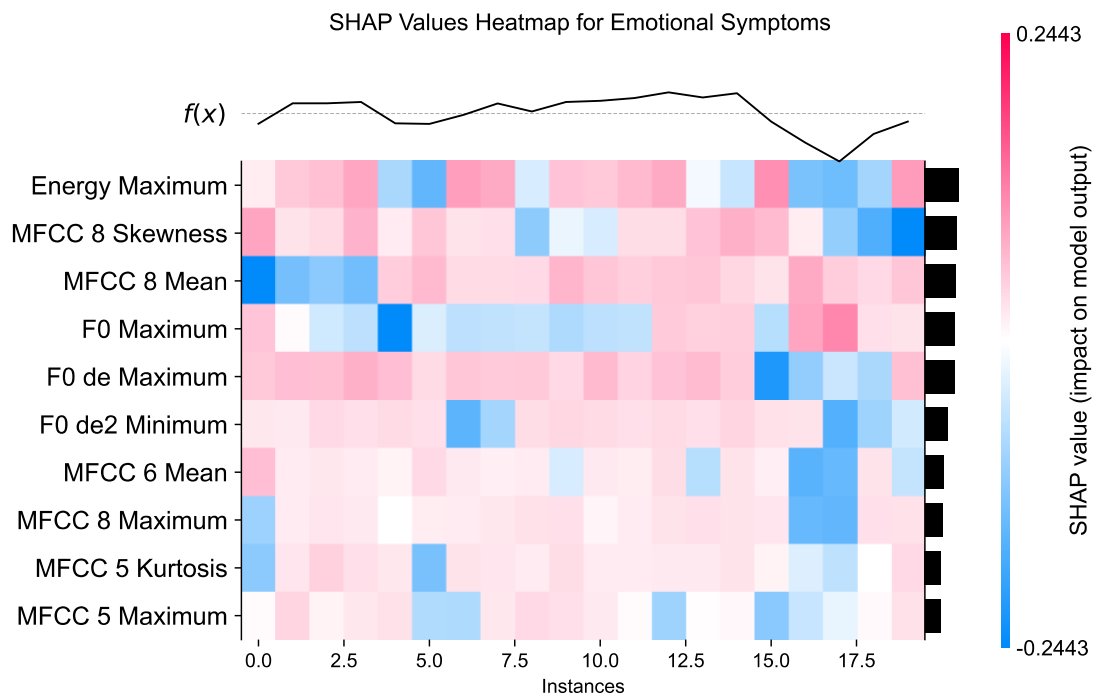

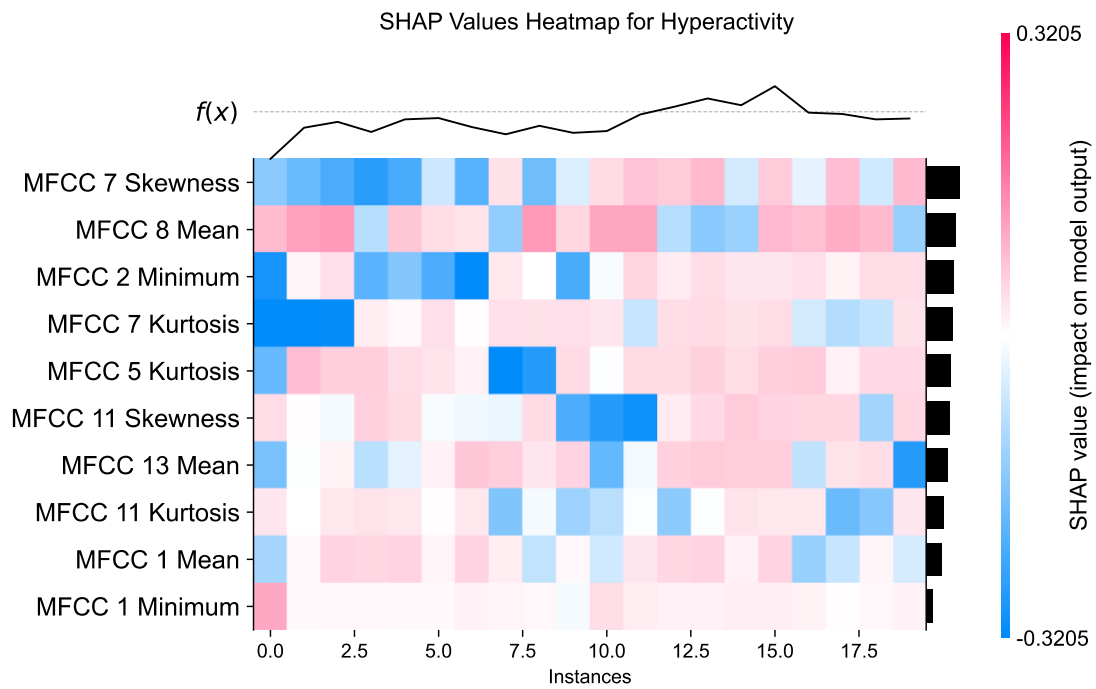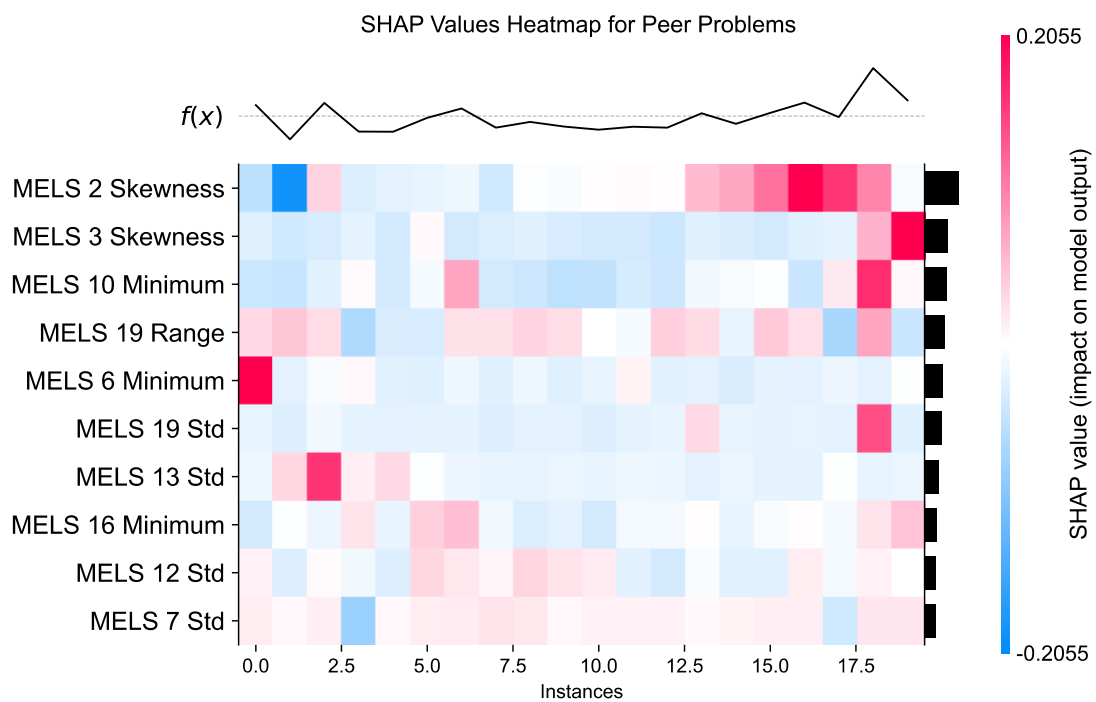

Supplement: Supporting Information 1 — The supporting information file includes all additional figures and tables referenced in the main text as Appendix A–G. [file 5734107.f1.zip › Appendix G.pdf]
